# Aryne‐Enabled C−N Arylation of Anilines

**DOI:** 10.1002/anie.202310583

**Published:** 2023-11-06

**Authors:** Thomas Sephton, Anastasios Charitou, Cristina Trujillo, Jonathan M. Large, Sam Butterworth, Michael F. Greaney

**Affiliations:** ^1^ School of Chemistry University of Manchester Manchester M13 9PL UK; ^2^ LifeArc, Accelerator Building Open Innovation Campus Stevenage SG1 2FX UK; ^3^ Division of Pharmacy and Optometry, School of Health Sciences, Manchester Academic Health Sciences Centre University of Manchester Manchester M13 9PL UK

**Keywords:** aryne, aniline, biaryl synthesis, C−N arylation, Smiles rearrangement

## Abstract

Anilines are potentially high‐value arylating agents, but are limited by the low reactivity of the strong C−N bond. We show that the reactive intermediate benzyne can be used to both activate anilines, and set‐up an aryl transfer reaction in a single step. The reaction does not require any transition metal catalysts or stoichiometric organometallics, and establishes a metal‐free route to valuable biaryl products by functionalizing the aniline C−N bond.

## Graphical abstract

A transition metal‐free C−N arylation of easily accessible tertiary anilines enabled by bifunctional arynes is described. The reaction proceeds via a tandem nucleophilic attack of anilines to arynes, followed by a Smiles‐Truce rearrangement to furnish synthetically valuable and densely substituted biaryls. This protocol was further utilized for heterocyclic scaffolds, providing access to medium size azaheterocycles via n+2 carbon insertion.

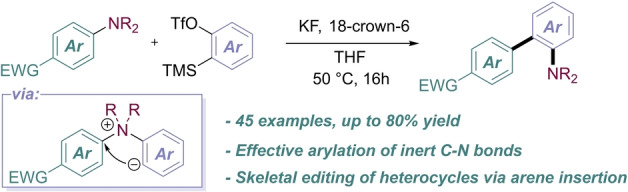

## Introduction

**Figure 1:**
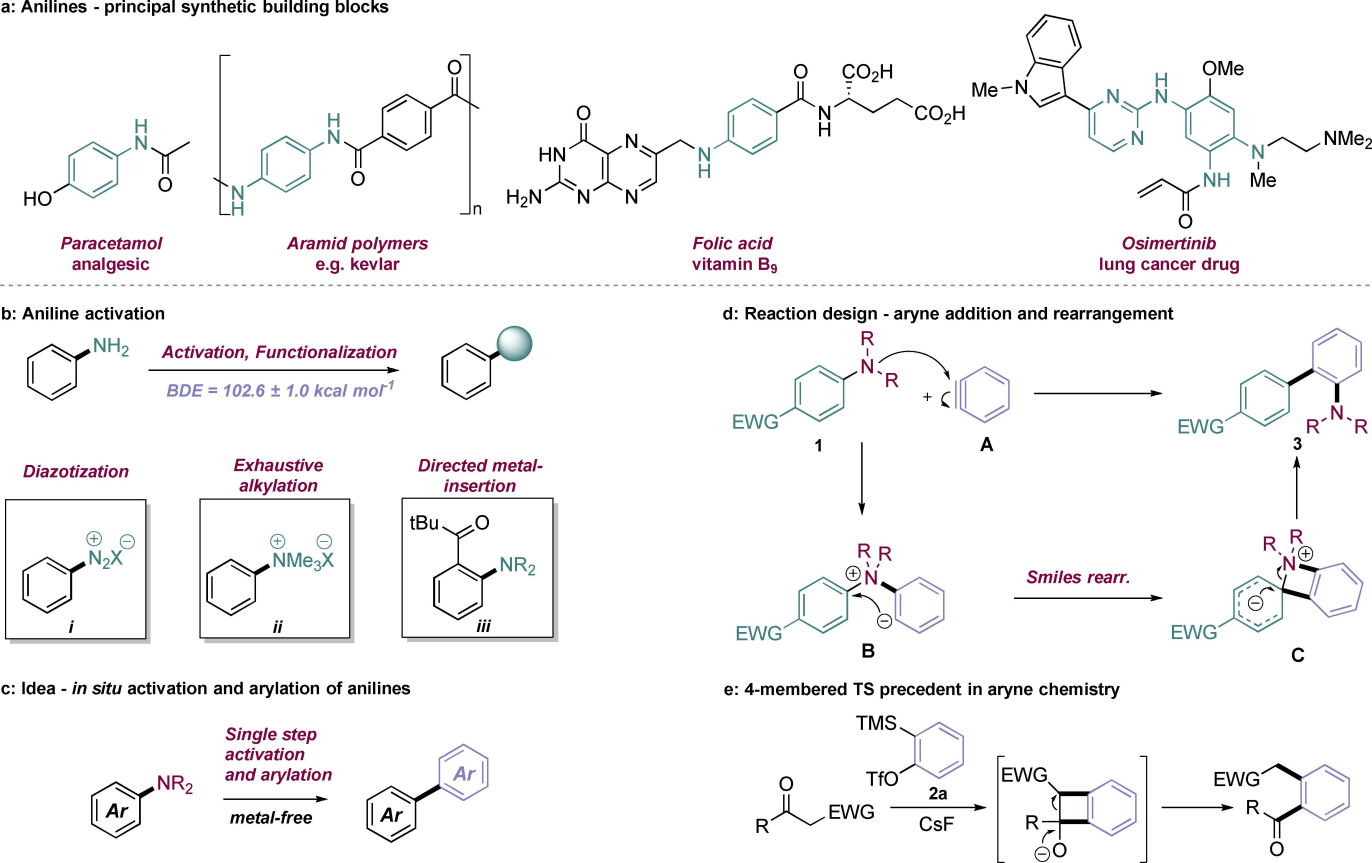
(a) Examples of pharmaceutically and industrially important aniline‐containing molecules. (b) Pre‐functionalization strategies for C−N bond functionalization. (c) Prospective in situ arylation of aniline C−N bond. (d) Aryne‐enabled C−N functionalization of electron‐poor anilines. (d) Precedent for four membered transition state in aryne *σ*‐insertion chemistry.

Anilines are one of the principal building blocks of chemistry, embedded in numerous man‐made molecules and natural products.^[1]^ First exploited in dye and pigment synthesis, anilines were formative in the development of the chemical industry and are fundamental motifs in a myriad of pharmaceuticals, polymers and biologically active compounds (Figure 1a).^[2]^ The tremendous versatility of aniline reactivity lies in methods for N‐functionalization, which are legion, and C‐functionalization through electrophilic aromatic substitution of the electron rich benzene ring. Direct manipulation of the C_(aryl)_−N bond represents a more difficult proposition, especially for C−C bond formation. The bond is strong (BDE=103 kcal mol^−1^) and requires high energy chemistry for activation, with diazonium salt formation being by far the most common method (Figure 1b). Strong mineral acids are used to generate a diazonium salt ***i***, which is highly explosive, but can be decomposed in a variety of ways to form new arene structures. This route is a text‐book method for simple aromatic C‐heteroatom transformations,^[3]^ and can also be applied to some metal‐catalyzed C−C bond formations.^[4]^ However, application to more complex and/or sensitive substrates is precluded due to the safety concerns and harshly acidic reaction conditions associated with diazonium formation. An alternative approach is to pre‐functionalize anilines as quaternary ammonium salts (***ii***) for metal‐catalyzed cross‐coupling. Wenkert and Macmillan demonstrated that salt ***ii*** can undergo Ni‐catalyzed cross‐coupling with Grignards and boronic acids, respectively, for C−C bond formation and access to biaryls.^[5]^ Whilst effective, the pre‐synthesis of ***ii*** requires an excess of methyl triflate, a highly toxic alkylating agent, which restricts application in terms of substrate scope and scale. Arylation of the aniline C−N bond is also feasible through direct insertion of transition metals, if a suitable chelating group is installed in the *ortho*‐position. Kakiuchi first showed that keto‐anilines (***iii***) could undergo Ru‐catalyzed C−C bond formation, although high temperatures and heavy catalyst loadings were required due to the inertness of the C−N bond.^[6]^ Despite these milestone advances, a mild and direct method for unlocking anilines as arylating agents remains elusive.

Our design approach to this problem is set out in Figure 1c. We wanted to develop a method that achieves aniline arylation in a single step, with no pre‐functionalization or isolation of an activated aniline intermediate. Our plan was to react a dialkylaniline **1** with the reactive intermediate benzyne **A**, creating the charged intermediate **B** which could then potentially undergo Smiles‐Truce rearrangement in situ to the biaryl product **3** (Figure 1d). Benzyne arylation pathways often forego the need for transition‐metal catalysis,[^7^, ^8^] and have emerged as synthetically viable routes to biaryls through the introduction of Kobayashi precursors **2**.[^9^, ^10^] Likewise, Smiles‐Truce transformations have received renewed attention in the recent literature, as they can enable common functional groups such as aryl sulfonamides, sulfinamides, and ureas to be used as C‐arylating agents under mild conditions.[^11^, ^12^]

Aniline addition to benzyne is a known reaction,^[13]^ whereas the postulated rearrangement step through a strained four‐membered, spirocyclic transition state has very little precedent. There are, however, many examples of analogous 4‐membered transition state aryne rearrangements onto carbonyl groups (e.g. Figure 1e).^[14]^ Whilst a C=C arene π‐bond has quite different reactivity to a C=O π‐bond, the demonstration of a viable benzocyclobutane transition state geometry was encouraging for the proposed arene transfer. If the electronic character of the aniline could be controlled such that it initially acted both as an N‐nucleophile with benzyne, and as a C‐electrophile for the subsequent Smiles rearrangement, then overall aniline arylation could be possible in a single step. We were further encouraged to find a single example of the desired reactivity in the literature, with Hosoya, Yoshida and co‐workers describing the arylation of N,N‐dimethylaniline with an unusually strained polycyclic aryne intermediate generated through a hexa‐dehydro Diels–Alder reaction.^[15]^ If this reactivity could be captured for benzyne, it would present an attractive methodology for the direct arylation of the inert C_(aryl)_−N bond, unmasking tertiary anilines as hidden functional handles.

## Results and Discussion

We initially selected the Kobayashi precursor **2 a** and N,N‐dimethyl‐4‐nitroaniline **1 a** as substrates ‐ the nitro group to activate the aniline ring for intramolecular S_N_Ar chemistry, and the tertiary aniline group to create a charged anilinium intermediate on aryne addition. Charge‐quenching is well known to accelerate rearrangement chemistry,^[16]^ and could assist the proposed arene transfer step via the strained four‐membered transition state.

Pleasingly, upon treatment of **1 a** and **2 a** with KF/18‐crown‐6 we obtained biaryl product **3 a** in a very low, but encouraging 7 % yield (Table 1, entry 1). Alongside the desired product, we obtained diarylamine **4 a** in 30 % yield, ostensibly arising from tandem benzyne protoamination/demethylation.^[17]^ Extensive screening established that eliminating adventitious water was key, and could be accomplished by drying KF and 18‐crown‐6 and using anhydrous THF under oxygen‐free conditions (see Supporting Information for additional information). Substantial improvement in efficiency was observed when excess aniline was used, resulting in a 35 % yield (entry 3). However, subsequent attempts to improve the yield were largely unsuccessful (see Supporting Information for additional information). Most solvents trialed exhibited trace reactivity, except for THF and dioxane (entry 5). Among the various fluoride sources screened, CsF and KF proved to be optimal, the latter retained thanks to its lower hygroscopic character and thus higher reliability (entry 6). Carrying out the reaction at 50 °C was found to be optimal, affording 38 % of the desired product (entry 7). As large quantities of unreacted aniline were consistently recovered, we surmised that the main hurdle to overcome was the attenuated nucleophilicity of the electron‐deficient aniline. We therefore looked to increase the availability of the nitrogen lone pair by exploring the effect of *ortho*‐substitution on the system, as the strategic placement of *ortho*‐groups is known to increase aniline basicity through steric inhibition of resonance effects.[^18^, ^19^]

**Table 1:** Reaction optimization. All reactions were carried out at 0.1 mmol scale under nitrogen.

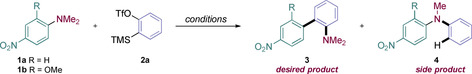							
Entry	R	1 [equiv]	2a [equiv]	Fluoride source [equiv]	Solvent, T [°C]	3 [%]^[a]^	**4** [%]^[a]^
**1**	H	1	2	KF (3), 18‐c‐6 (3)	THF, reflux	7	**30**
**2**	H	1	1	KF (3), 18‐c‐6 (3)	THF, 40	17	**<5**
**3**	H	3	1	KF (3), 18‐c‐6 (3)	THF, rt	35	**<5**
**4**	H	3	1	CsF (3)	1,4‐Dioxane, reflux	11	**<5**
**5**	H	3	1	KF (3), 18‐c‐6 (3)	THF, 50	30	**9**
**6**	H	3	1	CsF (3)	THF, 50	37	**6**
**7**	H	3	1	KF (3), 18‐c‐6 (3)	THF, 50	38 (36)	**<5**
**8**	**OMe**	**3**	**1**	**KF (3), 18‐c‐6(3)**	**THF, 50**	**70 (68)**	**<5**

[a] NMR yields using 1,3,5‐trimethoxybenzene as internal standard, isolated yields in parentheses.

This strategy proved successful, with the placement of an *o*‐methoxy group in the starting aniline affording 68 % of the desired biaryl product **3 b**, with very small amounts (<5%) of simple diarylamine side product (entry 8).

**Figure 2:**
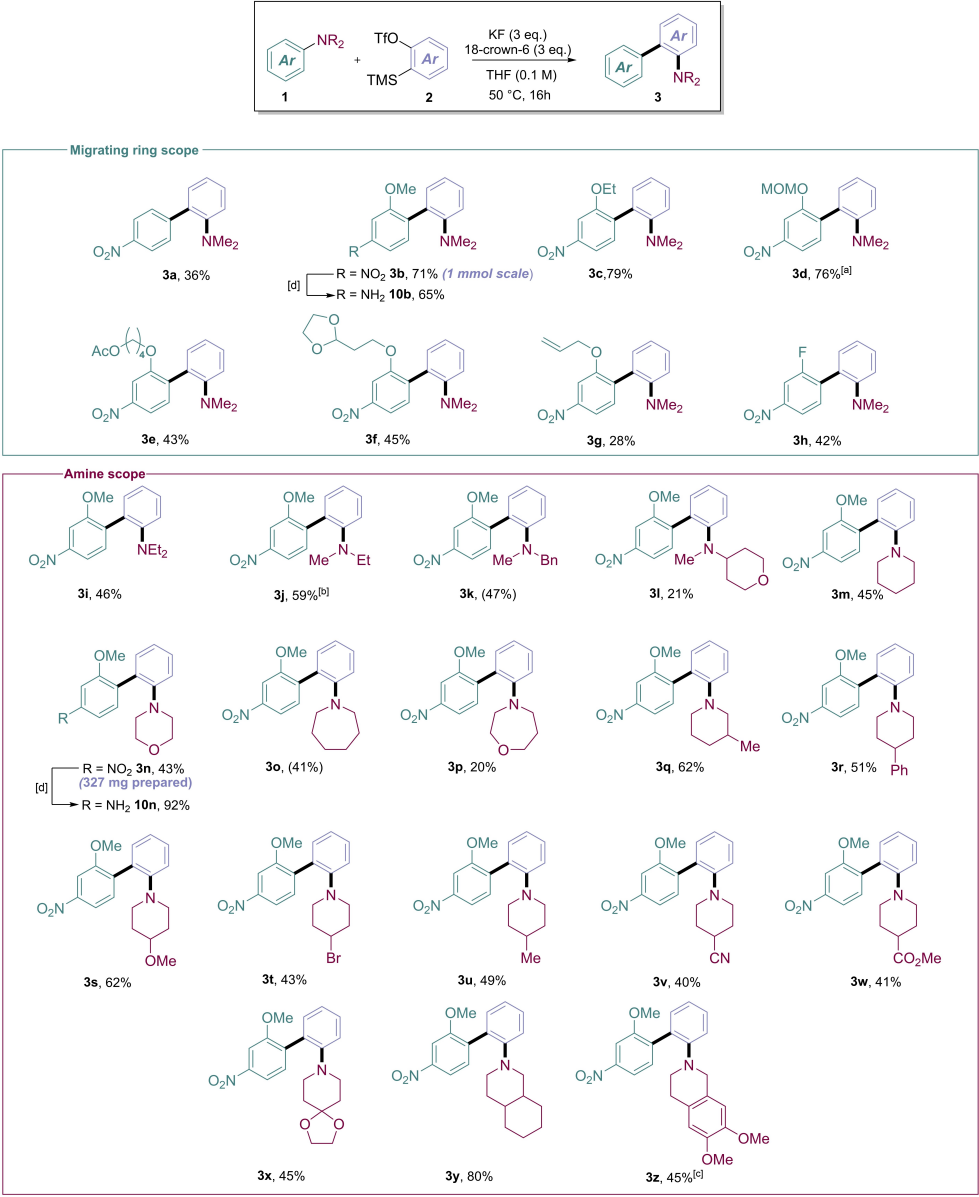
Scope of the transformation. Reactions performed on a 0.2 mmol scale, using 3.0 equivalents of aniline, isolated yields. NMR yields (nitromethane as internal standard) in parentheses. [a] 0.1 mmol scale, [b] 1.0 eq. aniline, [c] 2.0 eq. aniline. [d] Conditions: Fe_(s)_, NH_4_Cl (aq), MeOH, 50 °C, 16 h.

With the optimized reaction conditions in hand, we examined the scope of the system beginning with the migrating aniline ring (Figure 2). In addition to our two parent substrates (**3 a** and **3 b**), the reaction proceeded smoothly with various *o*‐alkoxyanilines, including a readily cleavable MOM ether **3 d**, masked aldehyde (**3 f**) and a masked alcohol (**3 g**). Additionally, *o*‐fluoro substitution was tolerated, affording biaryl **3 h** in moderate yield. The reaction required a strongly electron‐deficient *p*‐nitro group to proceed, and attempts to replace it with other electron‐withdrawing groups were mainly unsuccessful. The requirement of a *p*‐nitro group is, however, mitigated by its preeminent role as an amine surrogate in multi‐step organic synthesis. We demonstrated reduction of the nitro group using iron for **3 b** and **3 n**, enabling facile conversion to the electron rich primary anilines **10 b** and **10 n**.

After sufficient exploration of the migrating ring scope, we then examined the effect of *N*‐substitution. Pleasingly, simple N‐alkyl substitution was tolerated, furnishing a wide variety of N‐functionalized biaryls **3 i**–**z** in good yields, including **3 k** containing a readily cleavable benzyl protecting group, which could allow the synthesis of a secondary aniline and further N‐functionalization. Furthermore, the scope encompassed various common cyclic amines of varying ring size, including morpholine, piperidine, azepane and oxazepane, generating products **3 m**–**p** in moderate to good yields. Due to the success and ease of purification of the piperidine substituted example, it was employed as a scaffold for exploring the distal functional group tolerance of the system. Using our protocol, we synthesized biaryls **3 q**–**3 z**, providing access to N‐arylpiperidine derivatives, a prevalent motif in lead compounds and drugs.^[20]^ Importantly, C−N arylation occurred readily even in the presence of nitriles, esters, and acetals and generating substituted biaryls **3 v**–**3 x**, exemplifying the functional group tolerance of this approach. Interestingly, the reaction performed exceptionally on N‐arylperhydroisoquinoline, furnishing the corresponding biaryl product **3 y** in 80 % yield. The apparently high nucleophilicity of related isoquinoline‐derived substrates enabled the synthesis of a related product (**3 z**) using a lower excess of aniline. Some amount of starting material was consistently recovered throughout our substrate scope investigation, alongside small amounts of benzyne degradation products.

**Figure 3:**
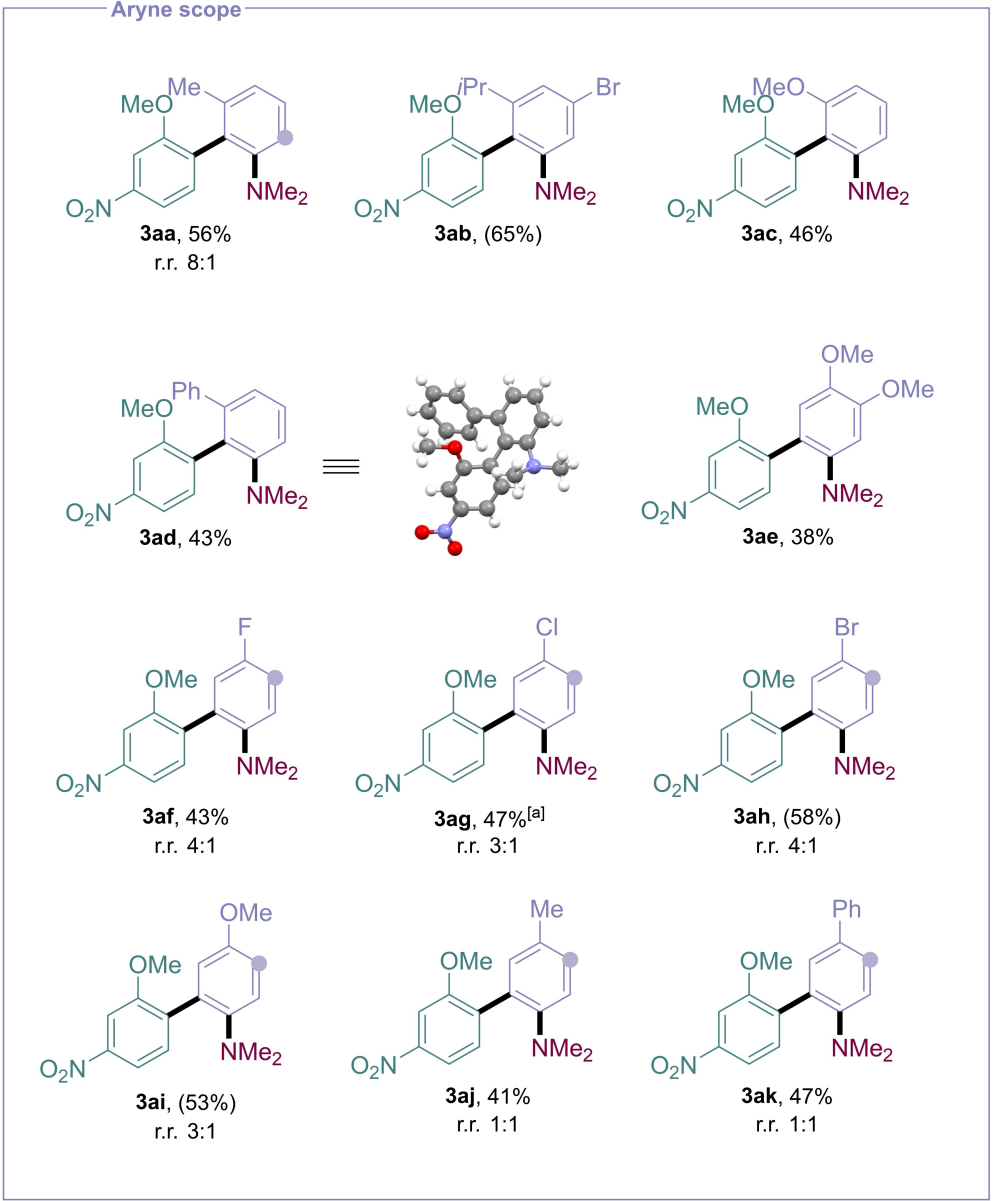
Scope of the aryne. Reactions performed on a 0.2 mmol scale, using 3 equivalents of aniline, isolated yields. NMR yields in parentheses. Filled circles represent site of substituent in minor regiosiomer. [a] 0.1 mmol scale.

Subsequently, we explored the scope of the aryne precursor (Figure 3). Nucleophilic addition to arynes substituted at the 3‐position can be highly selective,^[21]^ providing useful routes to hindered biaryls.^[22]^ We were therefore pleased to observe successful, regioselective reaction for several arynes containing *o*‐Me, *i*Pr, OMe, and Ph substituents, allowing for the facile synthesis of densely functionalized and sterically hindered biaryls **3 aa**–**3 ad**, the structure of **3 ad** being confirmed by X‐ray crystallographic analysis. Hindered biaryls containing three *ortho*‐substituents such as **3 aa**, **3 ab**, **3 ac**, and **3 ad** are challenging to access using conventional transition metal cross‐coupling protocols, requiring bespoke phosphine ligands and stoichiometric organometallics.^[23]^ *m*‐Substituted arynes could also be employed in the reaction, accessing biaryls with halogen, alkoxy, phenyl and methyl groups. The complementarity of the aryne approach is further emphasized by the tolerance to aryl bromides, which are untouched in the transformation and can be used for further synthetic manipulations (e.g. **3 ab** and **3 ah**).

**Figure 4:**
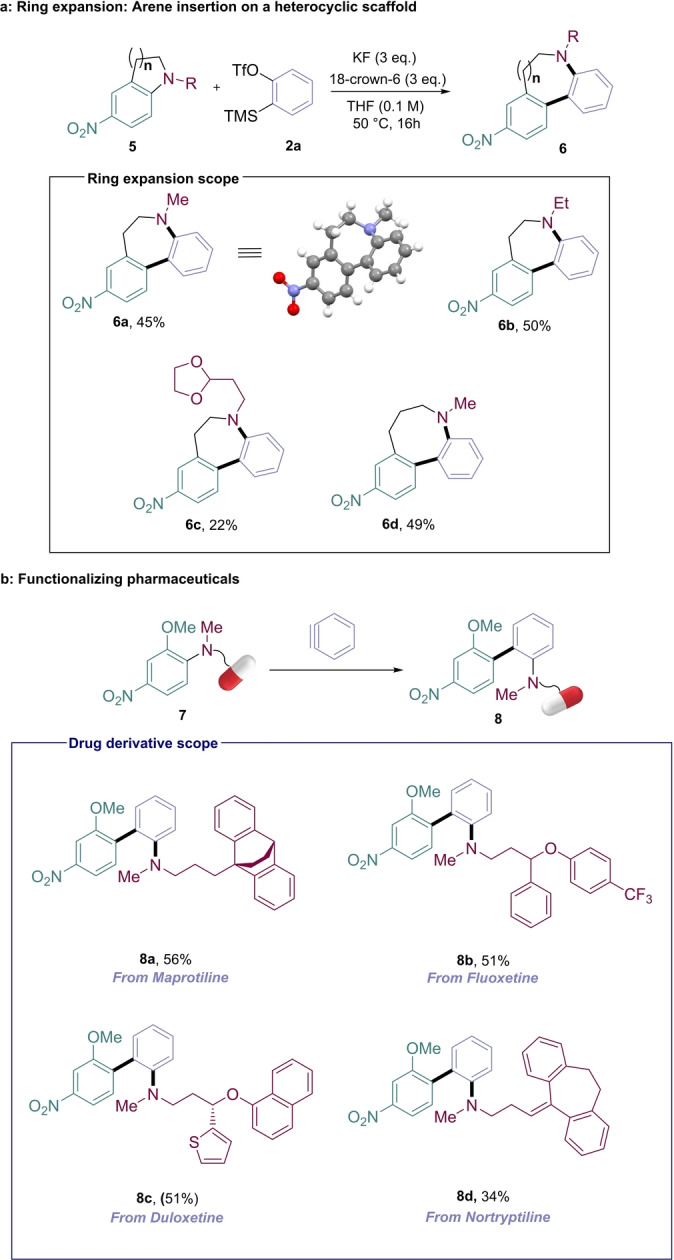
^a^ Aryne‐enabled ring expansion. ^b^ C−N arylation of drug derivatives. Reactions performed on a 0.2 mmol scale, using 3 equivalents of aniline, isolated yields. NMR yields (nitromethane as internal standard) in parentheses.

Upon completion of aniline substrate scope examination, we turned our attention to anilines embedded in cyclic structures. Insertion of benzyne into the inert C_(aryl)_−N bond of heterocycles **5** opens up the possibility of achieving n+2 ring expansion via arene insertion (Figure 4a), a novel transformation that is difficult to envisage in a single step using conventional methods. Gratifyingly, we found that when subjected to the standard reaction conditions, indoline **5 a** underwent ring expansion with reasonable success, affording tricyclic biaryl **6 a**, out‐competing side products arising from tandem protoamination/demethylation and Hoffmann elimination (see Supporting Information for additional information). The N‐substituent could be varied to access the dibenzazepanes **6 b** and **6 c**, and we could extend the process to a tetrahydroquinoline substrate to access the 8‐membered dibenzazocane **6 d**. To further explore the scope of the reaction with biologically‐active molecules, we functionalized several secondary amine‐containing anti‐depressant pharmaceuticals with the OMeN‐arene (*o*‐methoxy‐*p*‐nitro) unit using S_N_Ar chemistry (see SI) (Figure 4b). In each case, benzyne insertion into the aniline C−N bond proceeded smoothly under the standard fluoride reaction conditions, affording biaryl derivatives **8 a**–**8 d**.

**Figure 5:**
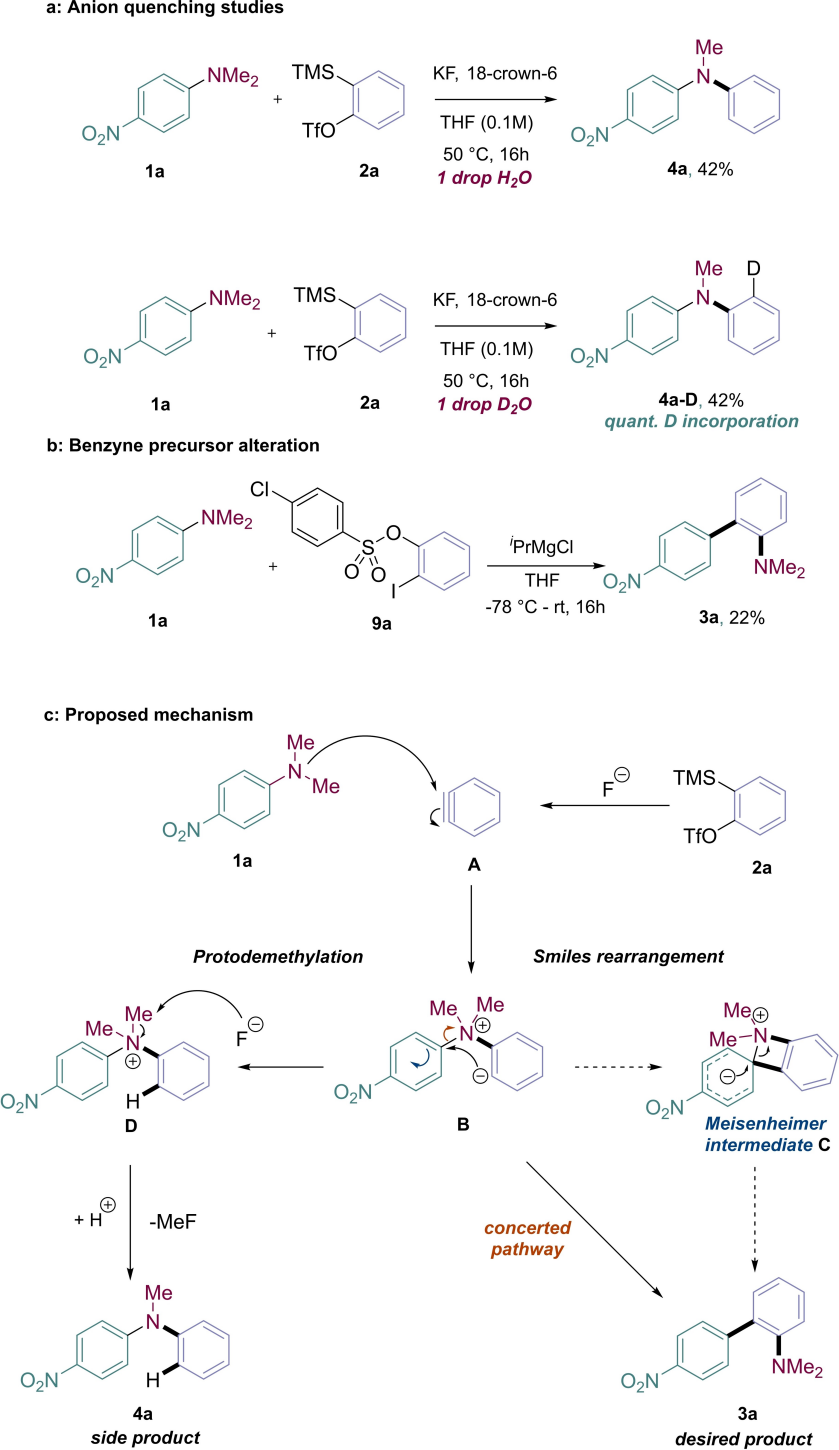
Mechanistic investigations. (a) Probing the protodemethylation pathway. (b) Benzyne precursor alteration (NMR yield, using 1,3,5‐trimethoxybenzene as internal standard). (c) Proposed mechanism.

To address the mechanism of the transformation, we focused first on investigating our hypothesis regarding side product formation (Figure 5a). We added a drop of H_2_O to our otherwise anhydrous system, which resulted in full conversion to diarylamine **4 a**, without any biaryl **3 a** detected, indicating that trace water is responsible for side product formation. When H_2_O was replaced with D_2_O, full conversion to diarylamine was again observed, this time with quantitative deuterium incorporation, providing further support that the proton in **4 a** originates from adventitious water. We then looked to confirm the role of a benzyne intermediate by altering the benzyne precursor used (Figure 5b). We substituted the Kobayashi precursor **2 a** for the Knochel‐type aryne precursor **9 a**, a well‐described Grignard system used in a variety of benzyne arylations.^[24]^ From this reaction, we observed the desired biaryl product **3 a** in a modest yield, strongly indicating that nucleophilic capture of benzyne by the aniline starting material is the first step in the mechanism. The subsequent Smiles‐Truce rearrangement step conventionally involves a Meisenheimer intermediate formed from carbanion attack onto the electron‐poor arene, which would involve a highly strained four‐membered azetidinium structure for this transformation (intermediate **C**, blue arrows). We were interested in examining the feasibility of this pathway and conducted a DFT study (wb97‐xD) to investigate the transformation (see Supporting Information S6 for details). Our analysis revealed an asynchronous concerted transition state situated between zwitterion **B** and product **3 a**, where the C−N bond undergoes cleavage and once it is nearly broken the C−C biaryl bond is formed. A discrete Meisenheimer intermediate was not located on the free energy profile. Accordingly, we propose the mechanism shown in Figure 5c for the overall transformation. First, aniline **1 a** attacks the highly electrophilic benzyne **A**, generating zwitterionic intermediate **B**. This then undergoes a concerted Smiles rearrangement (orange arrow) to generate biaryl **3 a**.^[25]^ In the presence of water, the aryl anion of intermediate **B** is quenched, forming intermediate **D**, which undergoes previously reported fluoride‐induced demethylation to produce diarylamine **4 a**.^[17]^

## Conclusion

In conclusion, we have developed a method for harnessing the aniline C−N bond for intermolecular arylation. In contrast to literature methods which pre‐functionalize anilines for transition‐metal catalyzed cross‐coupling, this reaction proceeds in a single step under mild, metal‐free conditions. The transformation can be applied to the synthesis of sterically‐encumbered biaryls using substituted aryne precursors, and preliminary experiments have established an arene‐insertion pathway for heterocycle expansion. Further applications of these transformations are underway in our laboratory.

## Supporting Information

The Supporting Information is available free of charge at http://pubs.acs.org. Synthetic methods, optimization studies, preparative procedures, mechanistic studies and ^1^H NMR, ^13^C NMR, ^19^F NMR spectra, HRMS, melting points and crystallographic data are available in the Supporting Information.^[26]^

## Conflict of interest

The authors declare no conflict of interest.

1

## Supporting information

As a service to our authors and readers, this journal provides supporting information supplied by the authors. Such materials are peer reviewed and may be re‐organized for online delivery, but are not copy‐edited or typeset. Technical support issues arising from supporting information (other than missing files) should be addressed to the authors.

Supporting Information

Supporting Information

Supporting Information

Supporting Information

Supporting Information

## Data Availability

The data that support the findings of this study are available in the supplementary material of this article.
