# Supplementary material for: Aryne‐Enabled C−N Arylation of Anilines
Source: Angew Chem Int Ed Engl. 2023 Nov 6;62(49):e202310583. doi: 10.1002/anie.202310583 (PMC10952162; doi:10.1002/anie.202310583)
Supplement: Supplementary file 5 — Supporting Information [file ANIE-62-0-s003.pdf]

Angewandte Chemie
Eine Zeitschrift der Gesellschaft Deutscher Chemiker

Supporting Information

Aryne-Enabled C–N Arylation of Anilines

T. Sephton, A. Charitou, C. Trujillo, J. M. Large, S. Butterworth, M. F. Greaney**

Table of Contents

1. General Remarks 3

2. Synthetic Procedures 5

3. Optimisation 9

3. Limitations 16

4. Substituted aryne regioselectivity 17

5. Mechanistic Experiments 23

6. Computational Study 26

7. X-ray Crystallography 35

8. Compound Characterisation 44

9. References 90

10. NMR Spectra 92

1. General Remarks

All air and/or moisture sensitive reactions were performed under an atmosphere of dry nitrogen using anhydrous solvents and standard Schlenk techniques. The glassware used for such reactions was oven-dried. Reagents and solvents were purchased from commercial sources at the highest available grade and used as supplied unless otherwise noted. Distilled THF, where used, was obtained from a Na/benzophenone still kindly provided by the group of David J. Procter. Small amounts of liquids were handled using 50, 100 and 250 µL Hamilton® 700 Series, 800 Series and GASTIGHT PTFE Luer-lock 1700 Series microsyringes, fit with Luer-lock needles.

Thin layer chromatography (TLC) was carried out using aluminium TLC plates coated with Silica gel 60 F_{254} from Sigma-Aldrich® cut using a guillotine, and spots were illuminated by a Spectroline® UV light lamp (365 nm). Flash column chromatography was performed using re-used 10g, 25g or 50g Biotage® Snap Ultra or Biotage Sfär Silica cartridges on a Biotage Isolera Four automated column, using 35-70 µm, 60 Å silica gel for chromatography from ThermoFisher Scientific® or 40-63 µm 60 Å silica gel from Sigma-Aldrich. Preparative thin layer chromatography was carried out using Uniplate™ Silica gel GF UV254 (20×20 cm, 2000 micron) plates from Analtech®.

^{1}H, ^{13}C and ^{19}F NMR spectroscopy were recorded on either 500 MHz (Bruker® AVII+ 500, Bruker AVIII HD 500) or 400 MHz (Bruker AVIII HD 400, Bruker AVIII 400) NMR spectrometers. Chemical shifts (δ) are reported in parts per million (ppm) and multiplicities are reported as singlets (s), broad singlets (brs), doublets (d), triplets (t), quartets (q), pentets (p), sextets (sx), heptets (h), combinations thereof (dt meaning a doublet of triplets), or multiplets (m). Coupling constants (J) are reported in Hertz (Hz). All ^{1}H NMR and ^{13}C NMR shifts were referenced to the residual solvent peak of $CDCl_3$ (^{1}H referenced to 7.26 ppm and ^{13}C referenced to 77.16 ppm). All ^{19}F chemical shifts were unadjusted from raw data. 2D heteronuclear single quantum coherence (HSQC), heteronuclear multiple bond correlation (HMBC), homonuclear correlation (COSY) and nuclear Overhauser effect (NOE) NMR spectroscopy were used to assist the assignment of signals and regiochemical assignments. NMR yields were calculated from ^{1}H NMR spectra using nitromethane and 1,3,5-trimethoxybenzene as internal standards on the basis of convenience. The NMR yields of scope examples were calculated from quantitative ^{1}H NMR spectra (^{1}H qNMR) using nitromethane as internal standard.

Mass spectrometry measurements were carried out by the Mass Spectrometry Service in the Department of Chemistry at the University of Manchester (experiments and analysis was carried out by Gareth Smith, Emma Enston, Otis Leahair and Carole Webb). High resolution mass spectrometry (HRMS) was recorded on ThermoFisher Scientific Q-Exactive™, Thermo Scientific Exactive plus EMR and Agilent 6530 Q-TOF instruments, using either electrospray ionisation (ESI), atmospheric-pressure chemical ionisation (APCI) - in some cases APCI was carried out using an atmospheric solids analysis probe (ASAP) - as ionisation methods in the positive and negative mode. Low resolution APCI was recorder on Agilent® 6120 Quadrupole LC/MS or a ThermoFisher Scientific Q-Exactive instruments in the positive and negative mode. Melting points (mp) were recorded on a Griffin melting point apparatus to the nearest degree and are uncorrected.

Compound names are those generated by ChemDraw® (PerkinElmer®) following International Union of Pure and Applied Chemistry (IUPAC) nomenclature. Novel compounds are labelled in italics.

2. Synthetic Procedures

General Procedure A: Synthesis of *N,N*-Dimethyl-4-nitroanilines

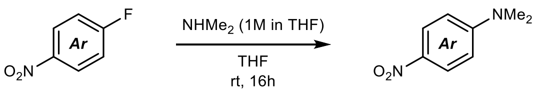

1a-b, 1h

A 2-5 mL oven-dried microwave vial was charged with the corresponding aryl fluoride (1.0 eq.), sealed, evacuated under vacuum and backfilled with nitrogen (×3). Dimethylamine (1 M in THF, 3.0-5.0 eq.) was added to the vial slowly *via* syringe. After stirring at room temperature for 16 hours, the resulting suspension was quenched with water and the aqueous layer was extracted with EtOAc (×3). The phases were separated and the combined organic layers were dried over $MgSO_4$, filtered, and concentrated *in vacuo*. If necessary, the crude product was then purified by flash column chromatography.

General Procedure B: *O*-alkylation of 1sm

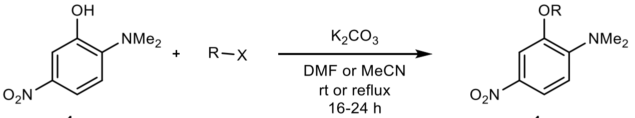

1sm **1c-g**

A 2-5 mL oven-dried microwave vial was charged with phenol **1sm** (1.0 eq.) and K_2CO_3 (1.0-1.5 eq.), sealed, evacuated and backfilled with nitrogen (×3). Anhydrous DMF (or MeCN) (0.3-1.0 M) and alkyl halide (1.1-1.5 eq.) were added *via* syringe. The resulting suspension was stirred at room temperature (at reflux for MeCN) for 16-24 hours. The crude mixture was cooled down to room temperature (if required), quenched with water and diluted with EtOAc. The phases were separated and the organic layer was washed with 10% aq. LiCl solution (×3) (for DMF). The organic layer was then dried over $MgSO_4$, filtered, and concentrated *in vacuo*. The crude product was purified by flash column chromatography**.**

General Procedure C: Synthesis of *N*-Substituted Anilines

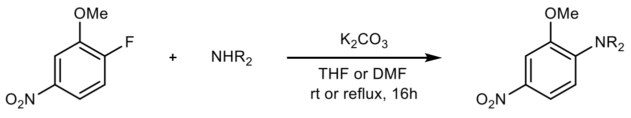

1i-1z, 7

An appropriately-sized oven-dried microwave vial was charged with 1-fluoro-2-methoxy-4-nitrobenzene (1.0 eq.), K_2CO_3 (2.0 eq.) and the corresponding amine (1.2-5.0 eq.) (if solid), sealed, evacuated, and backfilled with nitrogen (×3). Anhydrous DMF or THF (0.5 M) and the corresponding amine (1.2-5.0 eq.) (if liquid) were added *via* syringe and the mixture was stirred at room temperature or at reflux for 16 hours. The crude mixture was cooled down to room temperature (if required), quenched with water and diluted with EtOAc. The phases were separated and the organic layer was washed with 10% aq. LiCl solution (x3) (for DMF) and with 1 M aq. HCl solution. The organic phase was dried over $MgSO_4$, filtered, and concentrated *in vacuo*. If necessary, the crude product was then purified by flash column chromatography.

General Procedure D: *N*-Alkylation of Azaheterocycles

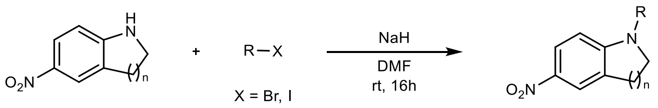

5

An appropriately-sized oven-dried microwave vial or round-bottom flask was charged with the corresponding heterocycle (1.0 eq.) and NaH (1.5 eq., 60% in mineral oil), sealed, evacuated and backfilled with nitrogen (×3). Anhydrous DMF (0.4 M) and the alkyl halide (1.2 eq.) were added *via* syringe. The resulting solution was stirred at room temperature for 16 hours. The crude mixture was quenched with water and diluted with EtOAc. The phases were separated and the organic layer was washed with 10% aq. LiCl solution (x3) and with 1M aq. HCl solution. The combined organic layers were washed with brine, dried over $MgSO_4$, filtered, and concentrated *in vacuo*. If necessary, the crude product was then purified by flash column chromatography.

General Procedure E: Metal-Free Aryne-Enabled *C-N* Arylation of Anilines

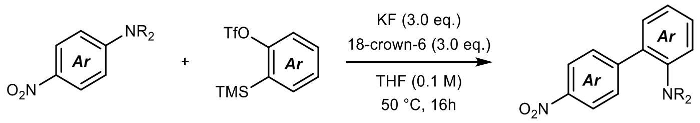

1 2 3

A 2-5 mL oven-dried microwave vial was charged with the corresponding nitroaniline **1** (3.0 eq.), dried KF (3.0 eq.), dried 18-crown-6 (3.0 eq.) and aryne precursor **2** (if solid). The vial was sealed, evacuated, and backfilled with nitrogen (×3). Distilled or anhydrous THF (0.1 M) was added *via* syringe, followed by aryne precursor **2** (if liquid) *via* microsyringe and the resulting suspension was stirred at 50 °C for 16 hours. The reaction mixture was cooled to room temperature, quenched with water, and diluted with EtOAc. The phases were separated and the aqueous layer was extracted with EtOAc (×3). The combined organic layers were washed with brine, dried over $MgSO_4$, filtered, and concentrated *in vacuo*. The crude product was then purified by flash column chromatography, and if necessary, preparative thin layer chromatography (10% EtOAc in hexane). For comments on the drying of the reagents and the purification of products, see notes below.

Notes:

1. As protonation of zwitterionic species **B** is very facile, the presence of adventitious water is deleterious for the desired Truce-Smiles rearrangement. To exclude any water from the reaction vessel, KF was dried for 2-3 h in a round-bottom flask at 140 °C under high vacuum, stored under nitrogen, sealed with a Suba-Seal® and secured with Parafilm®. 18-Crown-6 was dried under high vacuum in a round-bottom flask at room temperature for 2-3 h, and then stored as described above. Before each reaction, KF, 18-crown-6 and anilines **1** were kept under high vacuum at room temperature for at least 1 h. Still THF was used for optimal results; extra dry THF over molecular sieves purchased from Sigma-Aldrich can also be used, provided that the bottle is relatively new and kept dry.

2. Separation was challenging in most cases, due to the high lipophilic character of the products and the presence of small amounts of side products **4**. In most cases, removal of the remaining starting materials could be achieved by column chromatography using 0-5% EtOAc in hexane as eluent, followed by preparative thin layer chromatography using 10% EtOAc in hexane to remove **4**. Note that anilines **1** and side products **4** are highly UV-active, as are desired products **3**.

General Procedure F: Reduction of Nitrobiaryls

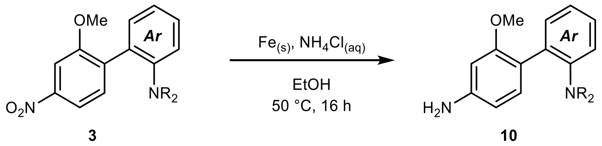

A 2-5 mL oven-dried microwave vial was charged with the corresponding nitrobiaryl **3** (1.0 eq.), ammonium chloride (0.7 eq.), Fe filings (5.0 eq.) and water (0.33 M). EtOH (0.5 M) was added *via* syringe and the resulting suspension was stirred at 50 °C for 16 h. The reaction mixture was cooled to room temperature and filtered. Water was added and the aqueous layer was extracted with EtOAc (x3). The combined organic layers were washed with brine, dried over $MgSO_4$, filtered, and concentrated *in vacuo*. The crude product was then purified by flash column chromatography.

3. Optimisation

Fluoride Source

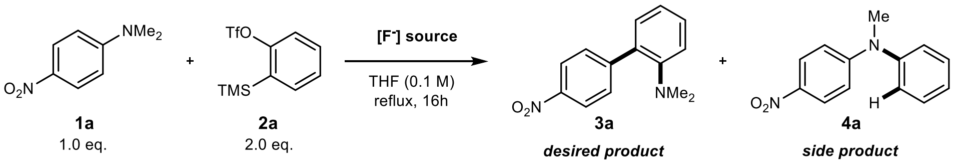

Entry	[F$^-$] source	Recovery of 1a (%)[a]	3 (%)[a]	4 (%)[a]
**1**	**KF (6.0 eq.), 18-crown-6 (6.0 eq.)**	**35**	**22**	**18**
2	CsF (6.0 eq.)	23	10	19
3	TBAF (2.0 eq.)	45	trace	13
4	TBAT (6.0 eq.)	45	0	36
5	TASF (3.0 eq.)	65	13	trace

Table S1. Screening of fluoride sources. All reactions were carried out on a 0.1 mmol scale. (a) Determined by NMR yield using 1,3,5-trimethoxybenzene or nitromethane as internal standard. TBAF = tetrabutylammonium fluoride, TBAT = tetrabutylammonium difluorotriphenylsilicate, TASF = tris(dimethylamino)sulfonium difluorotrimethylsilicate.

Temperature

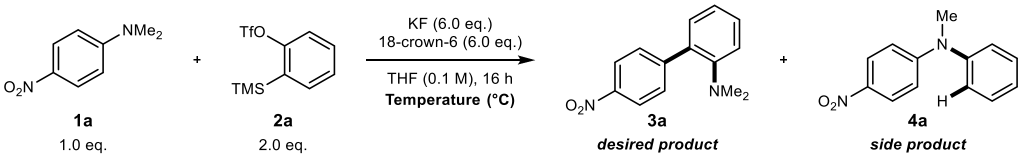

Entry	Temperature (°C)	Recovery of 1a (%)[a]	3 (%)[a]	4 (%)[a]
1[b]	-78 to rt	86	13	1
2	rt	55	15	0
**3**	**40**	**33**	**24**	**<5**
4	50	32	24	11
5	60	30	17	8
6	reflux	35	22	18

Table S2. Temperature optimisation. All reactions were carried out on a 0.1 mmol scale. (a) Determined by NMR yield using 1,3,5-trimethoxybenzene or nitromethane as internal standard. (b) Carried out with 3.0 eq. of KF and 18-crown-6 with a stoichiometry of **1a**:**2a** 3:1.

Reagent stoichiometry

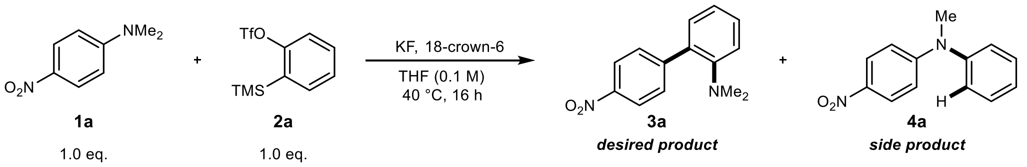

Entry	KF, 18-crown-6 (eq.)	Recovery of 1a (%)[a]	3 (%)[a]	4 (%)[a]
1	1.0	58	10	4
2	2.0	60	15	3
**3**	**3.0**	**50**	**18**	**4**
4	6.0	42	20	4

Table S3. Optimisation of fluoride source equivalents. All reactions were carried out on a 0.1 mmol scale. (a) Determined by NMR yield using 1,3,5-trimethoxybenzene or nitromethane as internal standard.

Additive

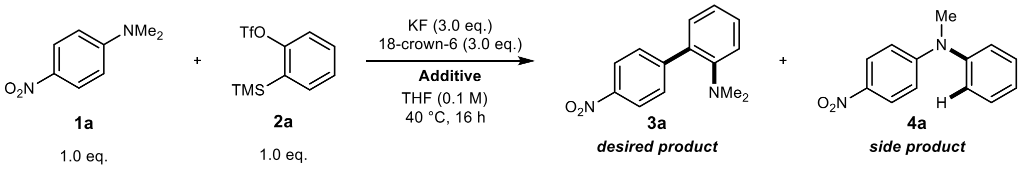

Entry	Additive (eq.)	Recovery of 1a (%)[a]	3 (%)[a]	4 (%)[a]
1	no additive	43	17	<5
2	AuCl (0.2)	52	12	8
3[b]	$Pd(OAc)_2$ (0.2)	75	3	1
4[b]	CuI (0.2)	85	trace	0
5[c,d]	LiBr (1.5)	90	0	0
**6**[d]	**no additive**	**nd**	**35**	**5**
7[d]	K_2CO_3 (1.0)	63	19	17
8[d]	$MgBr_2$ (1.0)	nd	0	0

Table S4. Screening of additives. All reactions were carried out on a 0.1 mmol scale. (a) Determined by NMR yield using 1,3,5-trimethoxybenzene or nitromethane as internal standard. (b) Small amounts (<15%) of triphenylene detected. (c) Carried out at -78 °C to rt. (d) Carried out under reflux with a stoichiometry of **1a**:**2a** 3:1.

Reactant stoichiometry

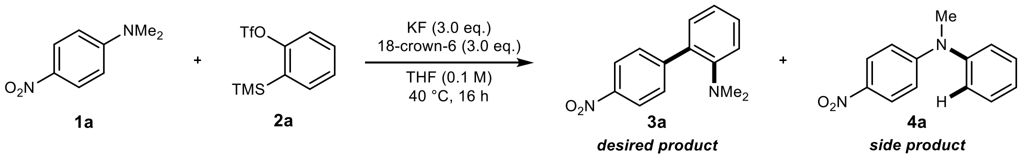

Entry	1a : 2a (eq.)	Recovery of 1a (%)[a]	3 (%)[a]	4 (%)[a]
1[b]	1:2	41	23	<5
2	1:1	43	17	<5
3	2:1	65	30	5
**4**	**3:1**	**nd**	**35**	**5**
5	5:1	75	33	10

Table S5. Optimisation of reactant stoichiometry. All reactions were carried out on a 0.1 mmol scale. (a) Determined by NMR yield using 1,3,5-trimethoxybenzene or nitromethane as internal standard. (b) 6.0 eq. KF and 18-crown-6 used, 0.2 M THF.

Solvent

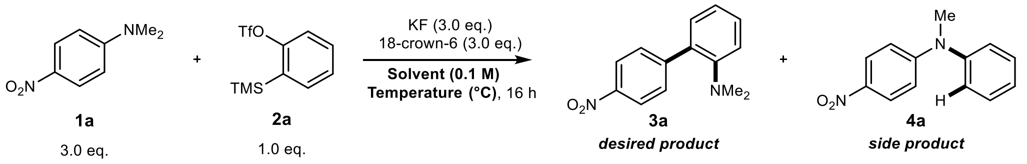

Entry	Solvent, Temperature (°C)	Recovery of 1a (%)[a]	3 (%)[a]	4 (%)[a]
1	1,4-dioxane, reflux	77	30	9
**2**	**THF, reflux**	**71**	**35**	**9**
3	DMF, 150	37	0	0
4	DMSO, 150	17	0	0
5[b,c]	PhMe/MeCN, reflux (120 °C)	55	<5	<5
6[c,d]	THF, rt	58	14	1
7[c,d]	MeCN, rt	15	3	6

Table S6. Screening of solvents. All reactions were carried out on a 0.1 mmol scale. (a) Determined by NMR yield using 1,3,5-trimethoxybenzene or nitromethane as internal standard. (b) 2.0 eq. of KF and 18-crown-6 used. (c) **1a**:**2a** 1.0:2.0 eq. (d) 6.0 eq. of CsF used.

Dryness

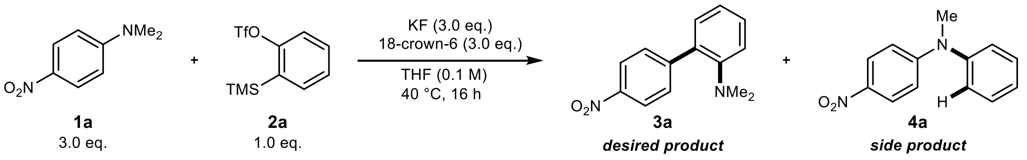

Entry	Drying conditions	Recovery of 1a (%)[a]	3 (%)[a]	4 (%)[a]
1[b]	KF, 18-crown-6 as purchased<br>Anhydrous THF	77	7	3
2[c]	KF and 18-crown-6 dried<br>Anhydrous THF	75	38	5
3[c,d]	KF and 18-crown-6 dried<br>Distilled THF<br>Molecular sieves (4Å)	39	0	0
**4**[c,d]	**KF and 18-crown-6 dried**<br>**Distilled THF**	**79**	**42**	**7**
5[c]	KF and 18-crown-6 dried<br>Anhydrous THF<br>glovebox	74	23	6
6[c,d,e]	KF and 18-crown-6 dried<br>Distilled THF<br>1 drop of water added	nd	0	42
7[c]	KF and 18-crown-6 dried<br>Distilled THF<br>under air	75	23	10

Table S7. Investigations on reaction sensitivity to air and water. All reactions were carried out on a 0.1 mmol scale. (a) Determined by NMR yield using 1,3,5-trimethoxybenzene or nitromethane as internal standard. (b) Carried out at reflux with a stoichiometry of **1a**:**2a** 1.0:2.0. (c) KF was drying by heating at 140 °C for a few hours, then stored in a vacuum oven at 100 °C or kept under N_2 in a round-bottom flask sealed with a suba seal and secured with Parafilm. (d) Distilled THF from a Na/benzophenone still. (e) Carried out at 50 °C.

Reaction time

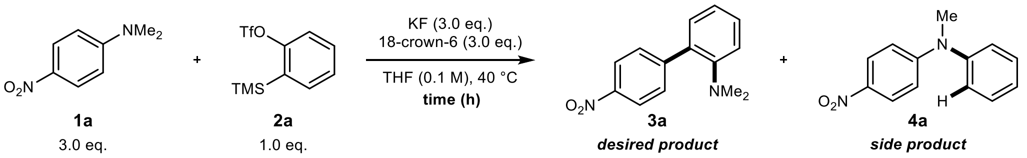

Entry	Reaction time (h)	Recovery of 1a (%)[a]	3 (%)[a]	4 (%)[a]
1	2	nd	12	0
2[b]	4	71	25	11
**3**	**16**	**nd**	**35**	**<5**
4	48	62	34	7

Table S8. Reaction time optimisation. All reactions were carried out on a 0.1 mmol scale. (a) Determined by NMR yield using 1,3,5-trimethoxybenzene or nitromethane as internal standard. (b) Carried out under reflux.

Concentration

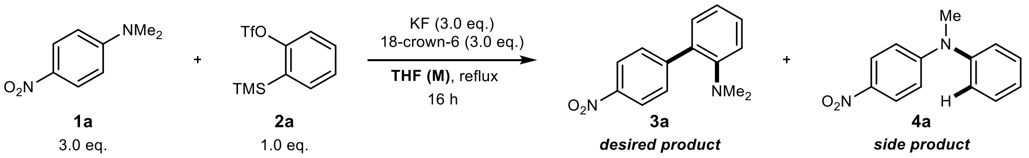

Entry	Concentration (M)	Recovery of 1a (%)[a]	3 (%)[a]	4 (%)[a]
1	0.03	76	0	0
**2**	**0.1**	**61**	**33**	**<5**
3	0.2	76	36	13
4[b]	0.4	79	29	15

Table S9. Concentration optimisation. All reactions were carried out on a 0.1 mmol scale. (a) Determined by NMR yield using 1,3,5-trimethoxybenzene or nitromethane as internal standard. (b) Carried out at 60 °C.

Cross-optimisation

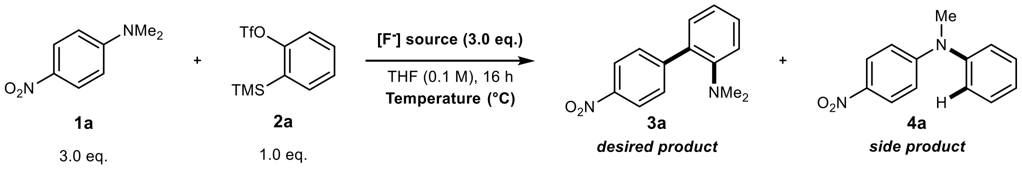

Entry	[F-] source	Temperature (°C)	Recovery of 1a (%)[a]	3 (%)[a]	4 (%)[a]
1	KF, 18-crown-6	40	79	42	7
2	KF (2.0 eq.),<br>18-crown-6 (2.0 eq.)	40	84	32	3
3	KF, 18-crown-6<br>0.2 M (on the basis of **2**)	40	nd	36	13
4	CsF	40	nd	33	<5
5	CsF	50	nd	37	6
**6**	**KF, 18-crown-6**	**50**	**54**	**38**	**<5**
7	CsF<br>glovebox	reflux	56	36	11

Table S10. Cross-optimisation. All reactions were carried out on a 0.1 mmol scale. (a) Determined by NMR yield using 1,3,5-trimethoxybenzene or nitromethane as internal standard.

Substrate optimisation

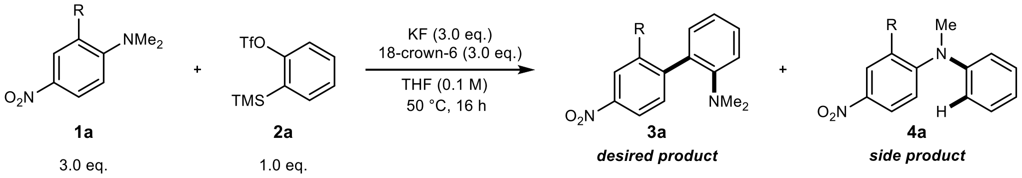

Entry	R	Recovery of 1a (%)[a]	3 (%)[a]	4 (%)[a]
1	H	54	38	5
2[b]	Me	39	13	34
**3**	**OMe**	**63**	**70**	**<5**

Table S11. Substrate optimisation. All reactions were carried out on a 0.1 mmol scale. (a) Determined by NMR yield using 1,3,5-trimethoxybenzene or nitromethane as internal standard. (b) Carried out at 40 °C using 2.0 eq. of **1a**.

3. Limitations

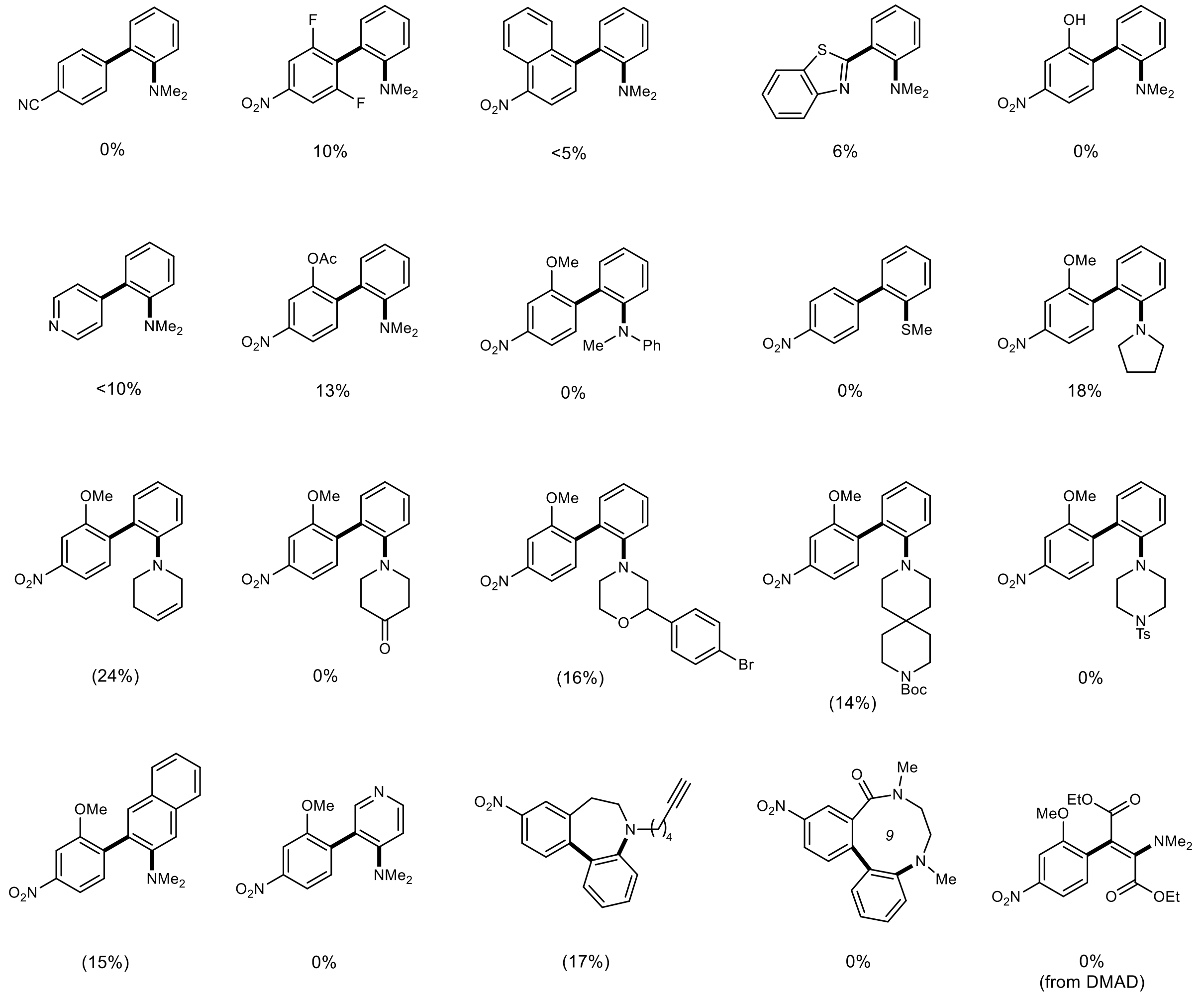

Figure S1. Low-yielding and unsuccessful scope examples. NMR yields, isolated yields in parentheses.

4. Substituted aryne regioselectivity

Addition to *meta*-substituted arynes (products **3af** – **3ak**) gave mixtures of regio-isomers, which were inseparable in most cases, co-eluting after several columns. The ^{1}H NMR spectra of the crude mixtures of the examples resulting in mixtures of regioisomers are shown below. Note that due to partial co-elution of products **3af-3ai** with side-products **4**, lower regioisomeric ratios were often obtained after column chromatography. Regiochemical assignments for compounds **3af-3ak** were made on the basis of ^{1}H-^{1}H NOE spectra of purified **3ah-major** and **3ah-minor** pictured below.

^{1}H-^{1}H NOESY of **3ah-major**

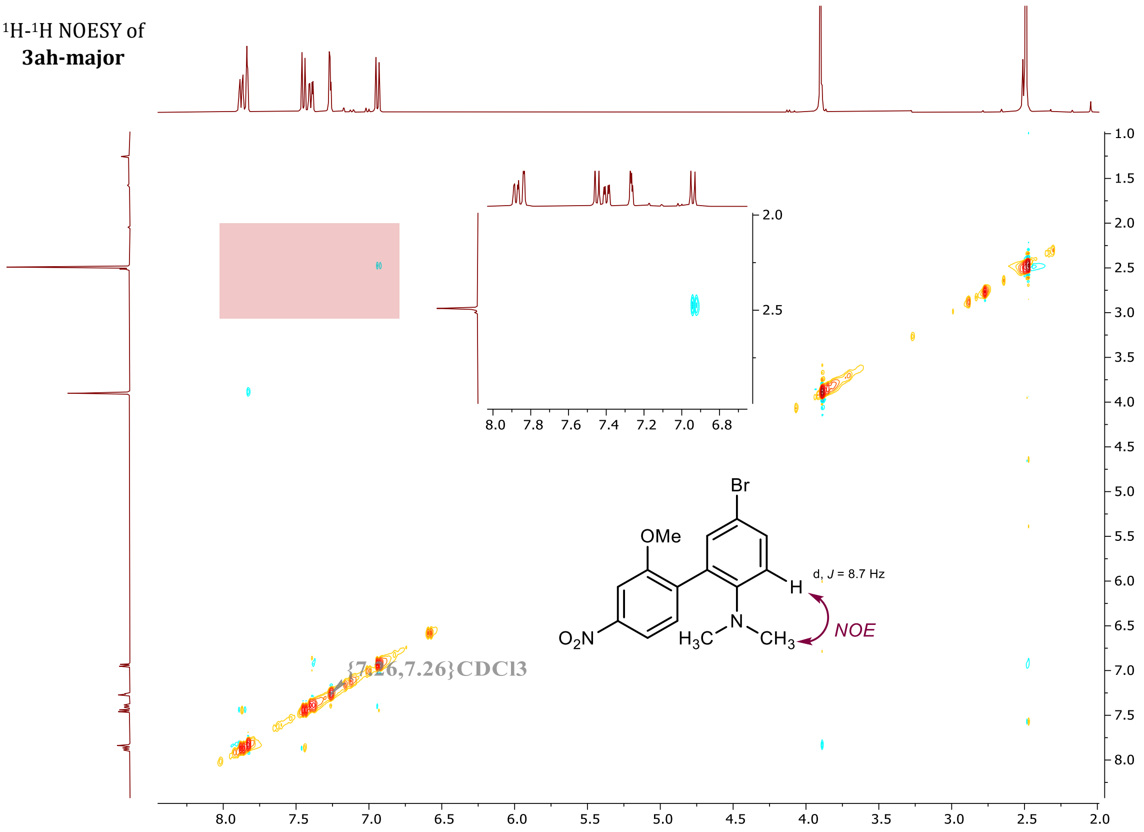

^{1}H-^{1}H NOESY of
3ah-minor

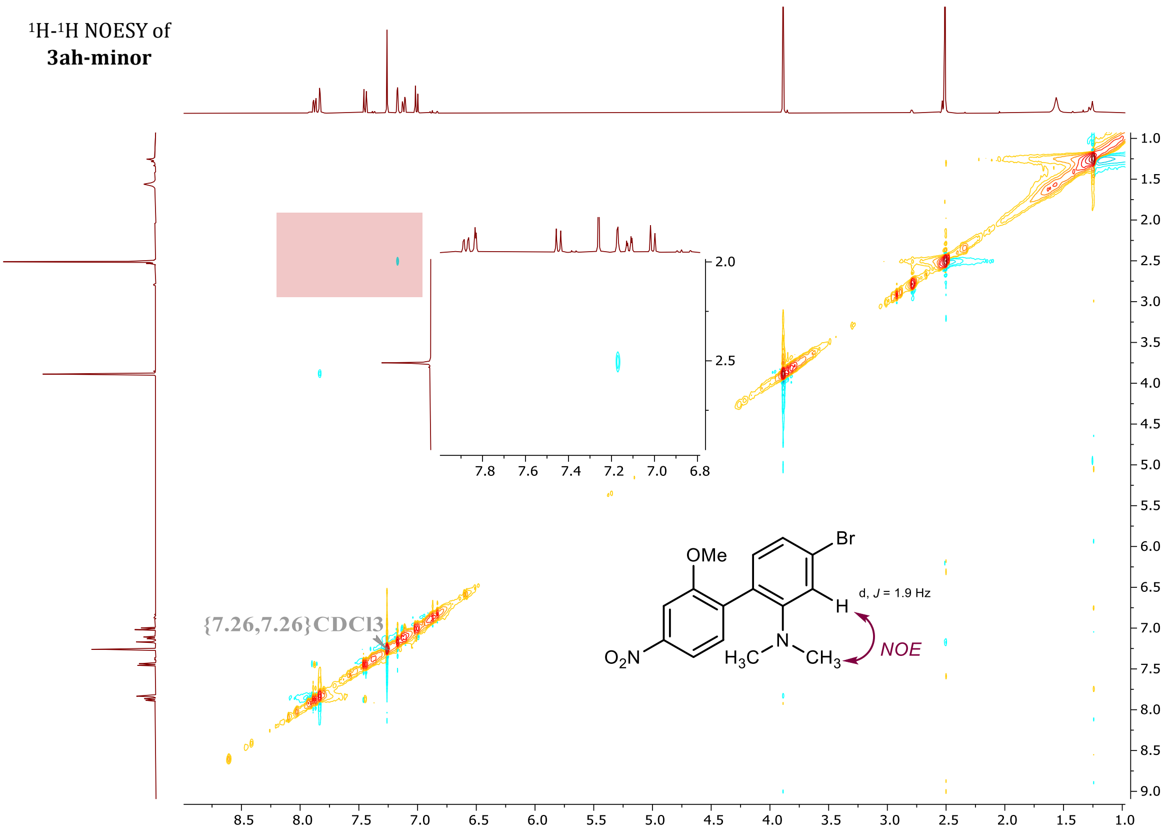

400 MHz, CDCl$_3$

Crude of **3aa**

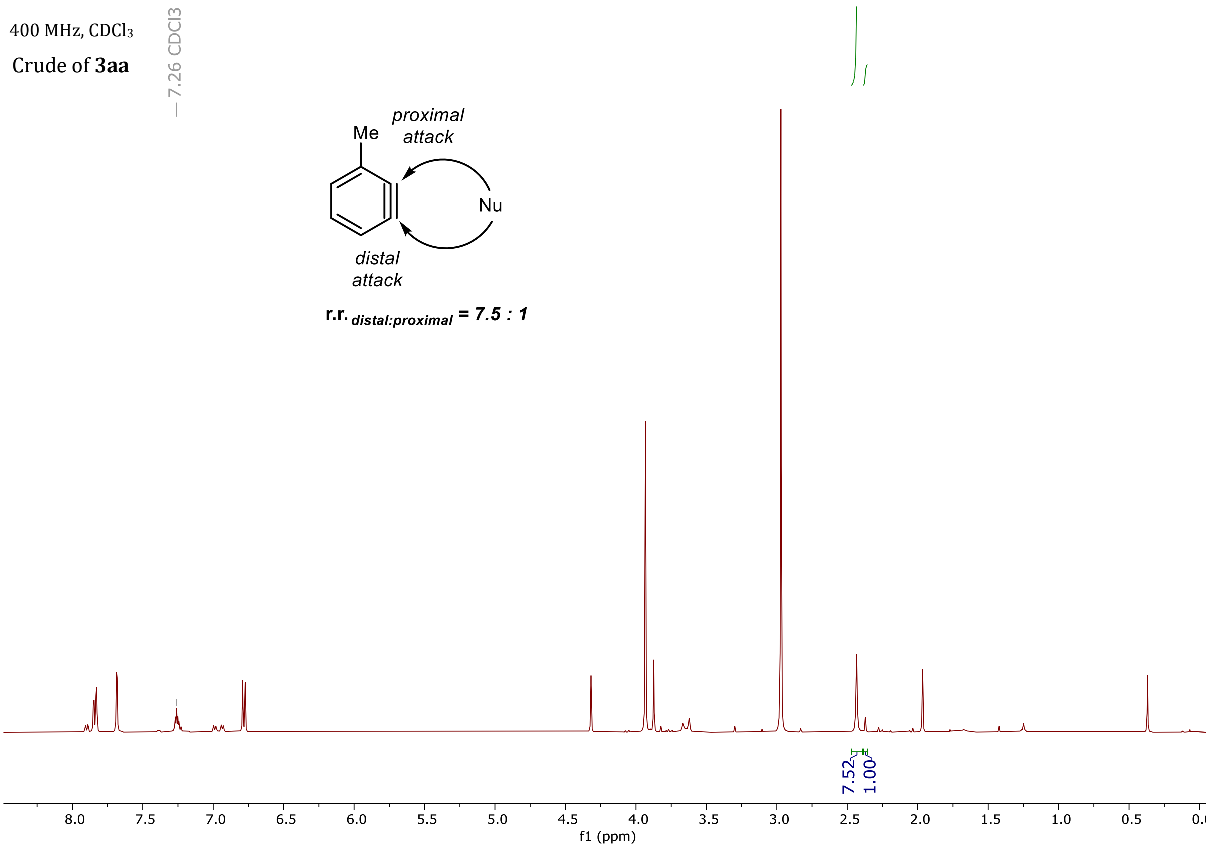

400 MHz, CDCl$_3$

Crude of **3af**

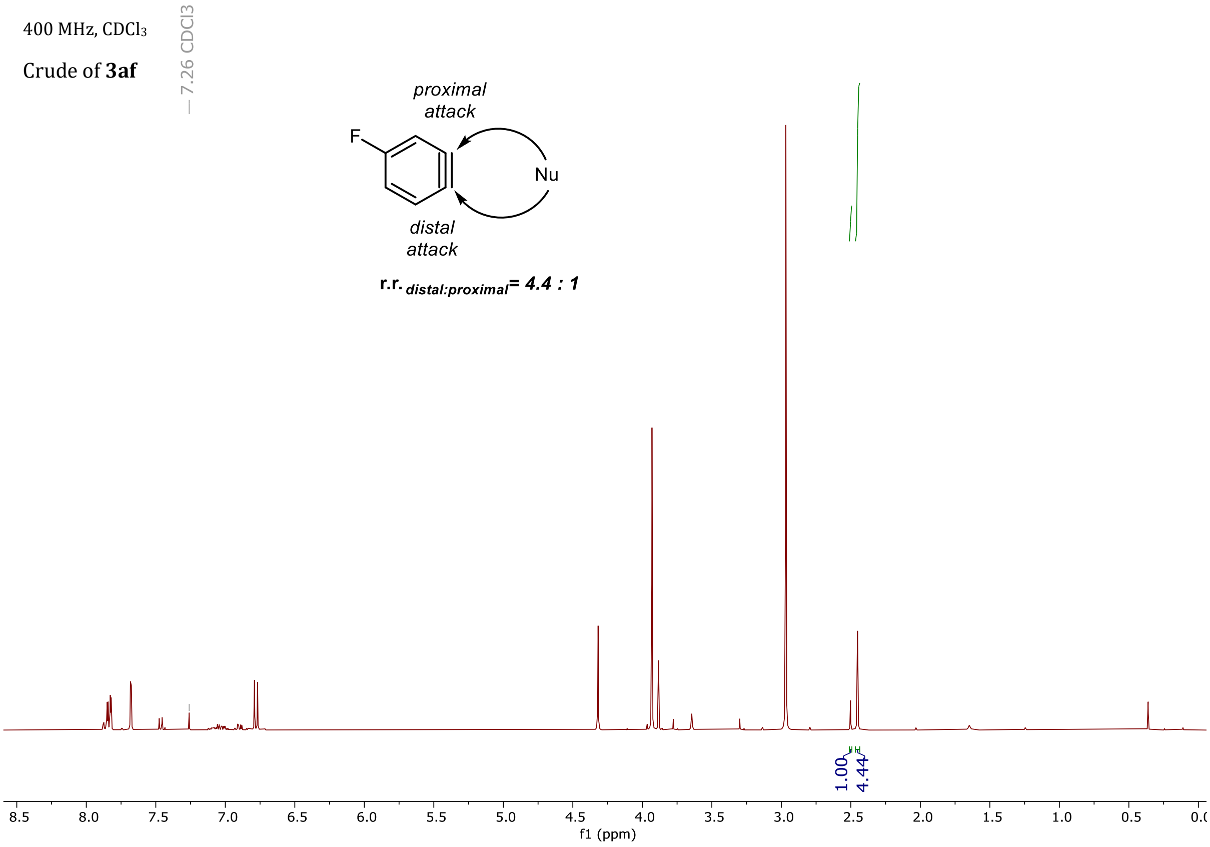

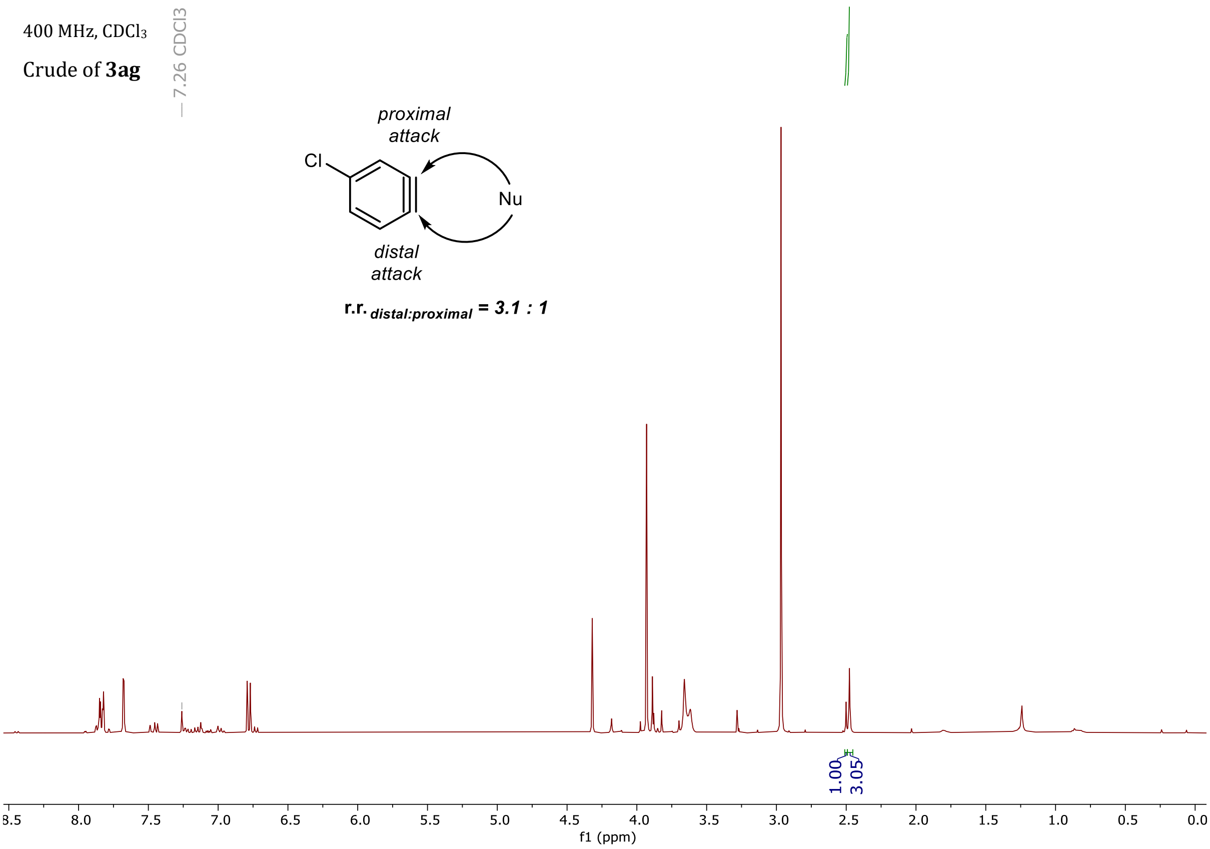

400 MHz, CDCl3
Crude of 3ag
7.26 CDCl3
proximal attack
Cl
Nu
distal attack
r.r. distal:proximal = 3.1 : 1
1.00
3.05
f1 (ppm)

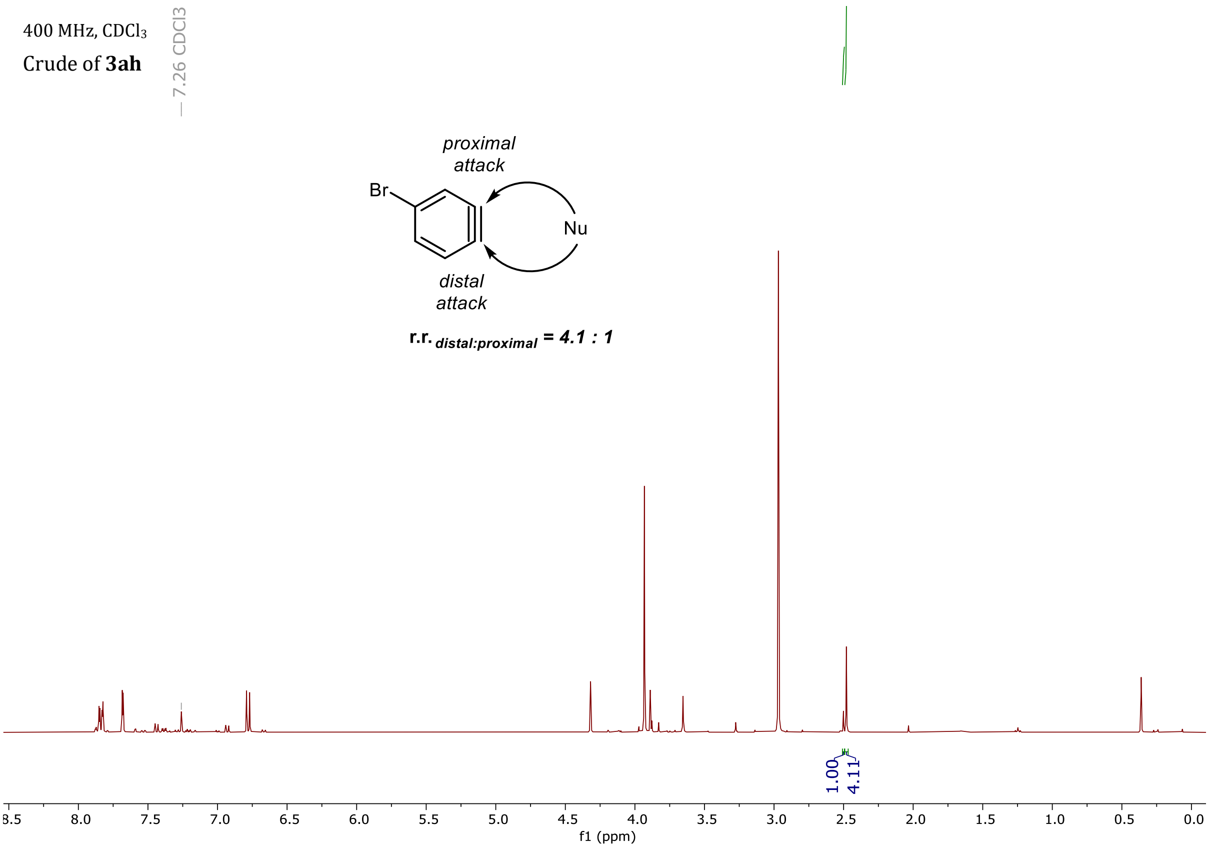

400 MHz, CDCl3
Crude of 3ah
7.26 CDCl3
proximal attack
Br
Nu
distal attack
r.r. distal:proximal = 4.1 : 1
1.00
4.11
f1 (ppm)

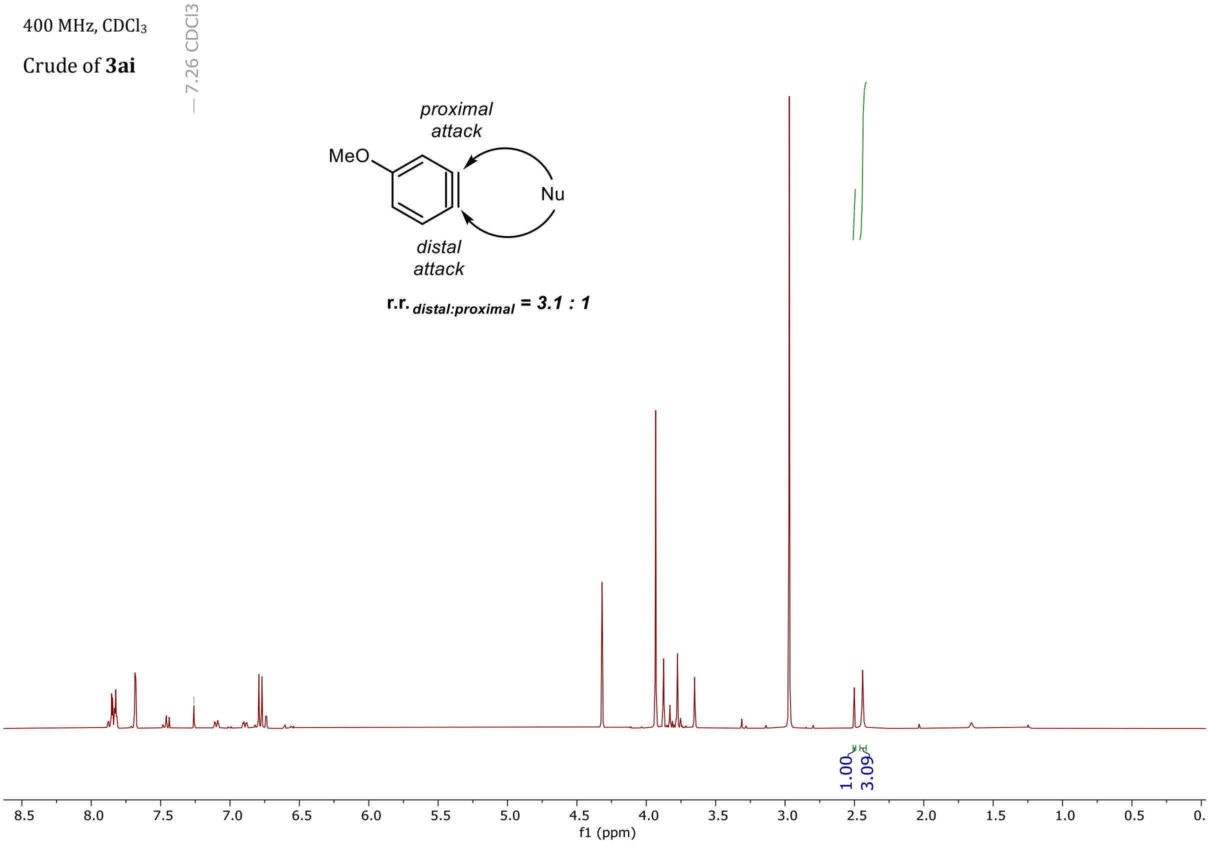
400 MHz, CDCl3
Crude of 3ai
7.26 CDCl3
proximal attack
MeO
Nu
distal attack
r.r. distal:proximal = 3.1 : 1
1.00
3.09
8.5
8.0
7.5
7.0
6.5
6.0
5.5
5.0
4.5
4.0
3.5
3.0
2.5
2.0
1.5
1.0
0.5
f1 (ppm)

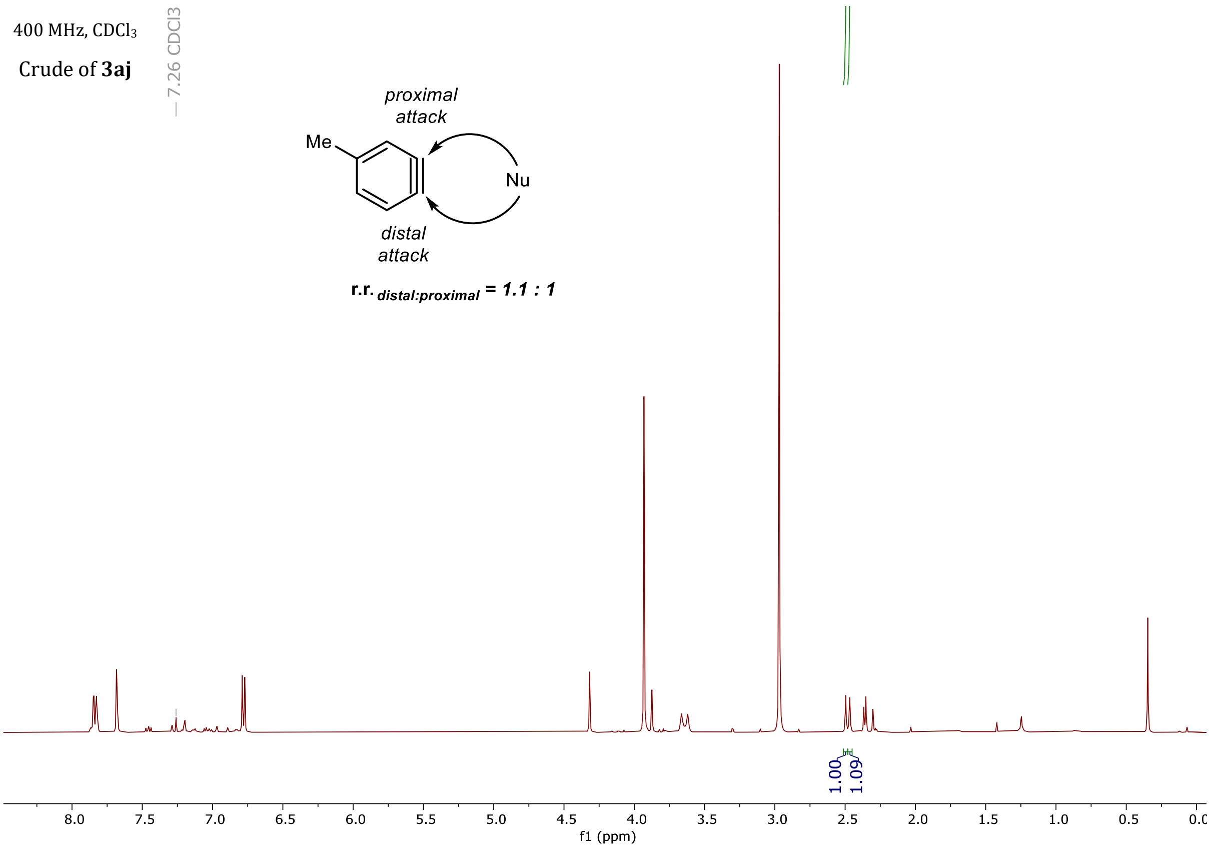
400 MHz, CDCl3
Crude of 3aj
7.26 CDCl3
proximal attack
Me
Nu
distal attack
r.r. distal:proximal = 1.1 : 1
1.00
1.09
8.0
7.5
7.0
6.5
6.0
5.5
5.0
4.5
4.0
3.5
3.0
2.5
2.0
1.5
1.0
0.5
f1 (ppm)

400 MHz, $CDCl_3$

Crude of **3ak**

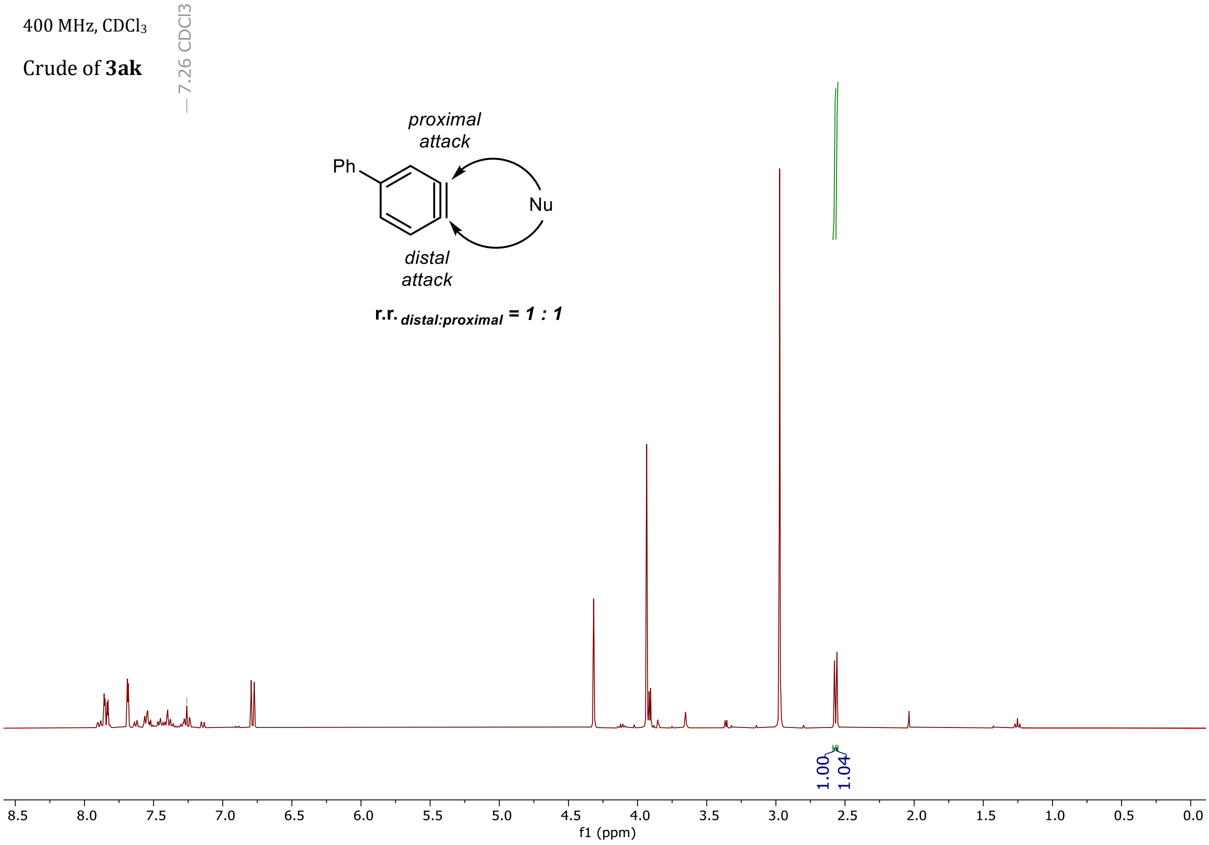

5. Mechanistic Experiments

Anion Quenching Studies

Addition of one drop of H_2O

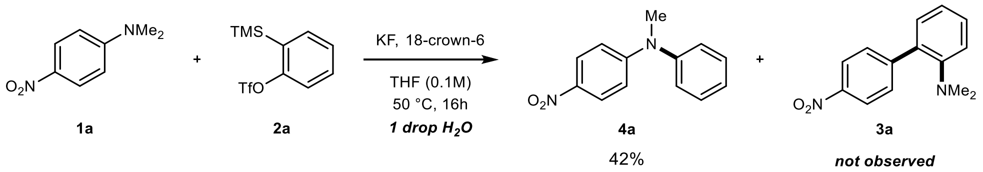

A 2-5 mL microwave vial was charged with *N*,*N*-dimethyl-4-nitroaniline **1a** (0.6 mmol, 102 mg), KF (0.6 mmol, 35 mg) and 18-crown-6 (0.6 mmol, 158 mg). The vial was sealed, evacuated and backfilled with nitrogen. Anhydrous THF (2.0 mL) was added *via* syringe, followed by aryne precursor **2a** (0.2 mmol, 49 µL) *via* microsyringe. A drop of H_2O was then added *via* syringe, and the reaction was left to stir at 50 °C for 16 hours. The crude mixture was cooled to room temperature and quenched with water. The aqueous layer was extracted with EtOAc (2 x 10 mL), and the combined organic phases were dried over $MgSO_4$, filtered and concentrated *in vacuo*. The crude product was then purified by flash column chromatography (column conditions: 0-15% EtOAc in hexane), affording **4a** as a yellow gum (19 mg, 42% yield). **3a** was not observed, indicating that in the presence of water, zwitterionic species **B** is protonated instead of undergoing the desired Smiles rearrangement.

***N*-Methyl-4-nitro-*N*-phenylaniline (4a)**

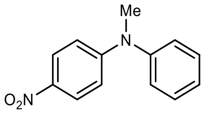

^{1}H NMR (400 MHz, CDCl$_3$) δ 8.09 – 8.04 (m, 2H), 7.51 – 7.40 (m, 2H), 7.36 – 7.29 (m, 1H), 7.27 – 7.19 (m, 2H), 6.70 – 6.64 (m, 2H), 3.41 (s, 3H).

^{13}C NMR (101 MHz, CDCl$_3$) δ 153.9, 146.6, 138.4, 130.4, 126.94, 126.85, 125.9, 112.6, 40.7.

HRMS (ESI) Calculated for $[C_{13}H_{12}O_2N_2+Na]^+$ 251.0791, found 251.0799.

The data are in accordance with the literature.[1]

Addition of one drop of D_2O

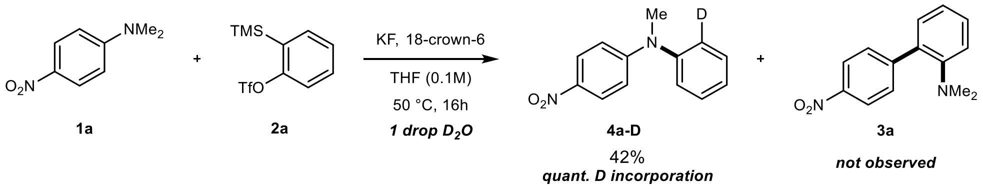

A 2-5 mL microwave vial was charged with *N*,*N*-dimethyl-4-nitroaniline **1a** (0.6 mmol, 102 mg), KF (0.6 mmol, 35 mg) and 18-crown-6 (0.6 mmol, 158 mg). The vial was sealed, evacuated and backfilled with nitrogen. Anhydrous THF (2.0 mL) was added *via* syringe, followed by aryne precursor **2a** (0.2 mmol, 49 μL) *via* microsyringe. A drop of D_2O was then added *via* syringe, and the reaction was left to stir at 50 °C for 16 hours. The crude mixture was cooled to room temperature and quenched with water. The aqueous layer was extracted with EtOAc (2 x 10 mL), and the combined organic phases were dried over $MgSO_4$, filtered and concentrated *in vacuo*. The crude product was then purified by flash column chromatography (column conditions: 0-15% EtOAc in hexane), affording **4a-D** as a yellow gum (19 mg, 42% yield). Quantitative deuterium incorporation in **4a-D** confirms that the source of protons is adventitious water.

N-Methyl-N-(4-nitrophenyl)aniline-2-D (4a-D)

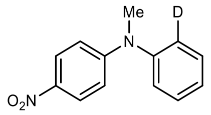

^{1}H NMR (400 MHz, $CDCl_3$) δ 8.11 – 8.02 (m, 2H), 7.50 – 7.41 (m, 2H), 7.30 (t, J = 7.5 Hz, 1H), 7.23 (d, J = 8.3 Hz, 1H), 6.72 – 6.63 (m, 2H), 3.41 (s, 3H).

^{13}C NMR (101 MHz, $CDCl_3$) δ 153.9, 146.5, 138.4, 130.4, 130.2, 126.9, 126.8, 126.6 (t, J = 24.0 Hz), 125.9, 112.6, 40.7.

HRMS (ESI) Calculated for $[C_{13}H_{11}DO_2N_2+Na]^+$ 252.0854, found 252.0862.

Benzyne precursor alteration experiment

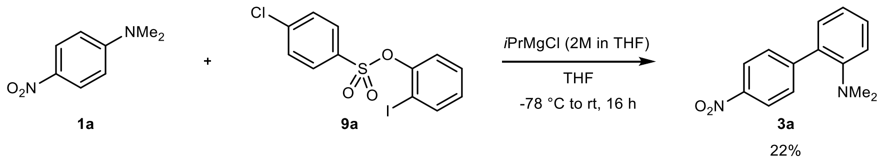

A 2-5 mL microwave vial was charged with **9a** (0.2 mmol, 80 mg), sealed, evacuated and backfilled with nitrogen (×3). Anhydrous THF was added (2.0 mL) *via* syringe and the vial was cooled to -78 °C. *i*PrMgCl (2 M in THF, 0.22 mmol, 0.11 mL) was added dropwise *via* syringe, and the mixture was stirred for 30 minutes. In a separate vial, **1a** (0.6 mmol, 100 mg) was dissolved in anhydrous THF (1.0 mL), and added to the reaction mixture. The resulting solution was left to warm up to room temperature. After stirring for 16 hours, the reaction mixture was quenched with sat. aq. NH_4Cl solution. The aqueous phase was extracted with EtOAc (×3) and the combined organic layers were washed with sat. aq. $NaHCO_3$ solution, dried over $MgSO_4$, filtered and concentrated *in vacuo*. The crude product was purified by flash column chromatography (0-30% EtOAc in hexane) to afford **3a** as a yellow solid (5 mg, 10% isolated yield, 22% NMR yield). The formation of desired product **3a**, albeit in a lower yield, confirms the presence of benzyne as an intermediate in the reaction.

6. Computational Study

Computational Details

The geometries of the structures in the reaction pathways were optimised using the density functional theory (DFT) method, wb97XD[2] functional, which is a long-range corrected hybrid density functional including empirical atom-atom dispersion correction, with the DFT standard split valence (SV) def2SVP basis sets.[3] Vibrational frequency calculations were performed to confirm that the optimised structures corresponded to a minimum in the free-energy profile or alternatively to a transition state. Single point energy calculations of optimised structures were obtained to increase accuracy of the free energy values by using the higher level, triple zeta valence (TZV) def2TZVP basis set.[4] All calculations were carried out with the Gaussian16 program[5] at 323 K temperature. Solvent effects, using THF as the solvent for the reaction were included in the optimisation and single point calculations, by means of the solvation model based on the density approach (SMD) implemented in Gaussian16.[6] Finally, intrinsic reaction coordinate (IRC) calculations were performed.

Scheme S1. *p*-Nitro(dimethyl)aniline (**1a**) + benzyne

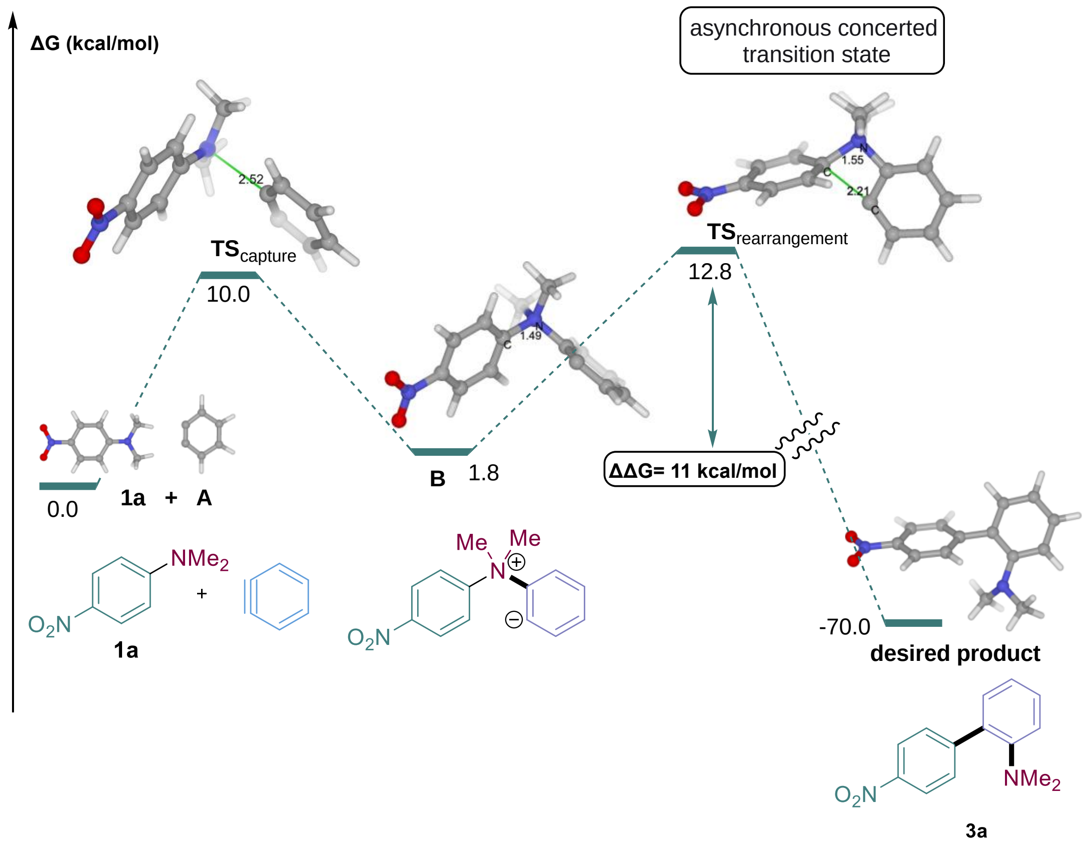

Scheme S2. Dimethylaniline + benzyne

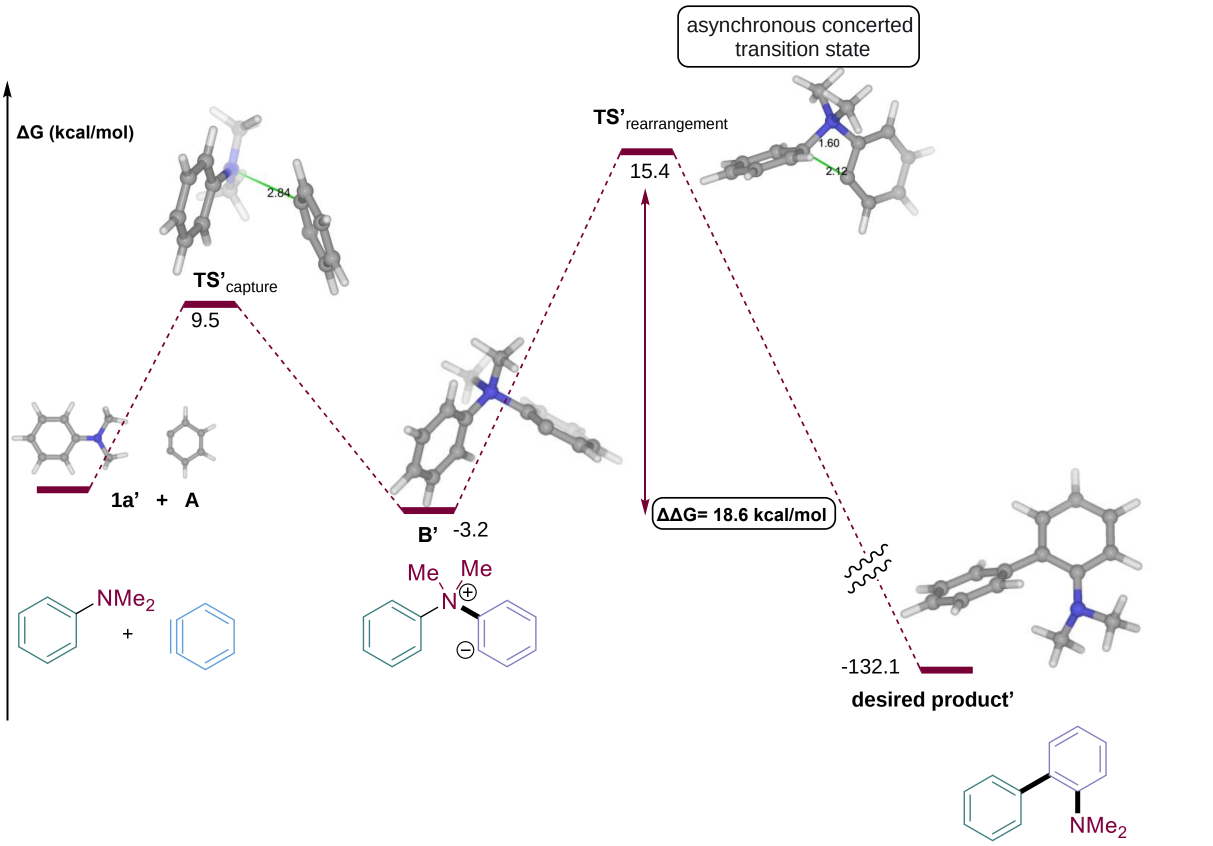

Calculations were carried out for the parent transformation of *p*-nitro(dimethyl)aniline **1a** with benzyne (Scheme S1), along with dimethylaniline and benzyne as a reference transformation (Scheme S2). Initial capture of benzyne was slightly less favourable for **1a**, in line with the *p*-NO_2 group deactivating nucleophilic addition, to form adduct **B**. For the key aryl transfer step, a transition state was identified between both minima, wherein the C-N bond undergoes cleavage, and once it is nearly broken, the new C-C bond is formed, resulting in the desired product **3a**. For the substrate lacking the NO_2 substituent the barrier is higher, nearly double, which aligns with the experimental results.

Optimised coordinates
1a

0 1
C 1.497407 -0.000002 -0.000159
C 0.803358 -1.211814 0.007657
C -0.578635 -1.215768 0.007117
C -1.317458 -0.000085 -0.000549
C -0.578718 1.215586 -0.008243
C 0.803320 1.211718 -0.008353

H 1.357675 -2.150301 0.015164
H -1.093357 -2.175090 0.015862
H -1.093534 2.174854 -0.017493
H 1.357548 2.150259 -0.015815
N -2.677118 -0.000002 -0.000514
C -3.406732 1.251495 0.020545
H -3.207123 1.859413 -0.878409
H -4.481641 1.041440 0.053972
H -3.154056 1.856499 0.907525
N 2.946857 0.000019 0.000240
O 3.523675 -1.072880 0.008606
O 3.523653 1.072928 -0.007764
C -3.407174 -1.251310 -0.019312
H -3.154892 -1.857691 -0.905393
H -3.207615 -1.857885 0.880567
H -4.482014 -1.040925 -0.053021

1a_noNO2

0 1
C 2.655162 0.000019 0.000161
C 1.939562 -1.197647 0.009213
C 0.547657 -1.208912 0.009152
C -0.190113 -0.000051 -0.000306
C 0.547597 1.208840 -0.009432
C 1.939502 1.197648 -0.009079
H 2.473101 -2.152126 0.017505
H 0.032296 -2.169142 0.018889
H 0.032170 2.169040 -0.019231
H 2.472991 2.152157 -0.017146
N -1.564757 -0.000009 -0.000579
C -2.287973 1.249820 0.016814
H -2.087200 1.862420 -0.880744
H -3.364922 1.045613 0.047104
H -2.039348 1.861061 0.902680
C -2.288165 -1.249751 -0.016334
H -2.039318 -1.862259 -0.901230
H -2.087871 -1.861141 0.882183
H -3.365066 -1.045399 -0.047431
H 3.747101 0.000047 0.000338

A

0 1
C 0.704373 1.054167 0.000040
C 1.464892 -0.132410 -0.000177
C 0.624513 -1.235449 0.000114
C -0.624527 -1.235450 0.000111
C -1.464891 -0.132398 -0.000180
C -0.704364 1.054172 0.000044
H 1.231166 2.012551 0.000202

H 2.556007 -0.130463 -0.000063
H -2.556006 -0.130440 -0.000060
H -1.231148 2.012561 0.000212

TS1a_A_noNO2

0 1
C 2.002682 -2.487214 0.342308
C 2.252536 -1.793463 -0.840803
C 1.975366 -0.432812 -0.951266
C 1.422001 0.284407 0.132494
C 1.191065 -0.424488 1.332905
C 1.472397 -1.783967 1.424613
H 2.677585 -2.316282 -1.701876
H 2.190274 0.069239 -1.894306
H 0.768005 0.083509 2.199141
H 1.270193 -2.301584 2.366156
N 1.085817 1.622038 0.019185
C 0.838403 2.387124 1.219802
H 1.683242 2.339495 1.931438
H 0.675166 3.437972 0.951531
H -0.070503 2.039037 1.740241
C -1.603118 0.839580 -0.437206
C -1.536302 -0.150147 -1.394806
C -2.325479 0.955154 0.580077
C -2.489360 -1.144157 -1.096073
H -0.873957 -0.205198 -2.259222
C -3.298078 0.016702 0.926146
C -3.337420 -1.064094 0.025644
H -2.568404 -2.008150 -1.761844
H -3.961788 0.066203 1.792441
H -4.048346 -1.875263 0.205730
C 1.423247 2.338383 -1.190492
H 2.515474 2.430322 -1.346122
H 0.995330 1.846129 -2.077382
H 0.997913 3.348359 -1.142327
H 2.221447 -3.554107 0.422035

TS1a_A

0 1
C -2.652092 -0.193397 -0.067943
C -2.147052 0.240942 1.155680
C -0.957565 0.949541 1.193693
C -0.239535 1.230758 0.007769
C -0.788656 0.790439 -1.220156
C -1.977292 0.083113 -1.256473
H -2.693431 0.027036 2.074123
H -0.590394 1.288212 2.160725
H -0.274163 0.989091 -2.158633
H -2.388157 -0.260080 -2.205736

N 0.975920 1.889942 0.040425
C 1.454691 2.512088 -1.181226
H 0.711838 3.209587 -1.604941
H 2.373110 3.068183 -0.960008
H 1.709823 1.754294 -1.938229
C 2.411464 -0.183976 -0.000771
C 2.186280 -0.893531 1.154124
C 3.102403 -0.385249 -1.037338
C 2.936100 -2.084891 1.106839
H 1.540795 -0.627462 1.991960
C 3.861558 -1.563353 -1.113768
C 3.749680 -2.407377 0.004310
H 2.876753 -2.775896 1.952218
H 4.496132 -1.840642 -1.960626
H 4.306847 -3.348105 0.019573
N -3.902454 -0.937782 -0.106223
O -4.460856 -1.175060 0.948161
O -4.328630 -1.287615 -1.190374
C 1.411177 2.500084 1.285656
H 0.758410 3.338727 1.588366
H 1.434829 1.764416 2.100780
H 2.432268 2.877771 1.156346

TS_B_product

0 1
C -2.742019 -0.169902 0.017427
C -1.899329 -0.510645 1.086929
C -0.565620 -0.184264 1.038899
C -0.000629 0.436970 -0.114084
C -0.892143 0.860726 -1.130681
C -2.232747 0.525444 -1.079805
H -2.309177 -1.030993 1.952433
H 0.081102 -0.484909 1.863671
H -0.527736 1.387758 -2.010163
H -2.900231 0.809235 -1.893572
N 1.302507 1.235336 0.120595
C 1.580016 2.219053 -0.966798
H 0.817985 3.007276 -0.955873
H 2.569343 2.649448 -0.774231
H 1.594840 1.695520 -1.928005
C 2.287391 0.135214 0.029765
C 3.587169 0.175260 0.516489
C 1.671683 -0.905214 -0.634837
C 4.346361 -0.980205 0.312542
H 4.006896 1.043369 1.031361
C 2.466861 -2.046235 -0.819122
C 3.787371 -2.079990 -0.348771
H 5.376142 -1.023368 0.674850
H 2.074836 -2.933980 -1.330274
H 4.395316 -2.977796 -0.496080

N -4.142629 -0.509468 0.067831
O -4.557905 -1.107897 1.046840
O -4.852708 -0.185221 -0.870045
C 1.290261 1.968780 1.418373
H 0.409863 2.621607 1.444827
H 1.265194 1.258778 2.249804
H 2.203624 2.571973 1.479939

TS_B_product_noNO2

0 1
C 3.652085 -0.926441 0.152282
C 3.159275 -0.441689 -1.058307
C 1.860708 0.046714 -1.179485
C 0.957625 0.004404 -0.086616
C 1.497305 -0.422525 1.161527
C 2.794042 -0.900735 1.258509
H 3.799995 -0.433418 -1.945036
H 1.523079 0.380723 -2.160186
H 0.854232 -0.461529 2.042218
H 3.143487 -1.262503 2.229933
N -0.151682 1.159752 0.002859
C 0.002341 2.008941 1.211537
H 1.009266 2.443085 1.213773
H -0.748010 2.808128 1.173388
H -0.152553 1.409183 2.112661
C -1.343960 0.301623 0.004415
C -2.646971 0.689114 0.290032
C -0.908915 -0.957720 -0.347527
C -3.609552 -0.321904 0.220501
H -2.921046 1.712896 0.557587
C -1.902229 -1.946059 -0.412042
C -3.237139 -1.626573 -0.126227
H -4.654820 -0.091654 0.440298
H -1.658880 -2.981250 -0.680396
H -4.005646 -2.404319 -0.171647
C -0.156570 2.041737 -1.194788
H 0.796542 2.579951 -1.258597
H -0.321954 1.436734 -2.091701
H -0.983960 2.753481 -1.083696
H 4.673765 -1.301086 0.238514

desired_product

0 1
C -2.806137 -0.149776 -0.089705
C -2.263153 -1.091463 0.777323
C -0.883834 -1.268593 0.789774
C -0.049573 -0.515511 -0.049604
C -0.633536 0.425280 -0.914421
C -2.008334 0.612023 -0.941006

H -2.913461 -1.669384 1.433298
H -0.445930 -1.995977 1.476528
H 0.002974 1.020319 -1.569967
H -2.466754 1.335465 -1.614549
N 1.923326 1.640646 0.186894
C 2.811297 2.707274 -0.214157
H 2.236496 3.644032 -0.275083
H 3.647549 2.877563 0.495025
H 3.234726 2.505973 -1.208562
C 2.359181 0.317996 0.010716
C 3.723401 0.010715 -0.128188
C 1.421517 -0.746948 -0.056867
C 4.159297 -1.301626 -0.299216
H 4.462854 0.810886 -0.082188
C 1.885518 -2.059854 -0.197585
C 3.243151 -2.349056 -0.320307
H 5.228669 -1.502150 -0.402039
H 1.154928 -2.870942 -0.254977
H 3.576092 -3.381147 -0.447481
N -4.260138 0.047152 -0.108692
O -4.935890 -0.629274 0.639383
O -4.711842 0.876360 -0.871635
C 1.185911 1.957336 1.397192
H 0.533032 2.828114 1.226829
H 0.551854 1.120812 1.712824
H 1.868280 2.198376 2.237260

desired_product_no_NO2

0 1
C -3.812515 0.021330 -0.201254
C -3.294944 -0.874291 0.732836
C -1.921021 -1.111768 0.789029
C -1.041971 -0.456896 -0.084216
C -1.575324 0.440790 -1.021808
C -2.946604 0.675809 -1.079997
H -3.962665 -1.390621 1.426866
H -1.521370 -1.807045 1.531905
H -0.902925 0.961643 -1.705833
H -3.344371 1.374574 -1.820149
N 1.017314 1.631769 0.098773
C 1.965792 2.635709 -0.323040
H 1.438042 3.593619 -0.448920
H 2.787765 2.804777 0.403657
H 2.410601 2.366154 -1.291726
C 1.401586 0.282183 0.005028
C 2.756092 -0.085031 -0.067085
C 0.421185 -0.745108 -0.043998
C 3.145178 -1.420397 -0.158719
H 3.525980 0.686662 -0.032241
C 0.838598 -2.079097 -0.108035

C 2.187097 -2.429016 -0.165511
H 4.208510 -1.667379 -0.211813
H 0.077078 -2.862095 -0.153833
H 2.480378 -3.478911 -0.231698
C 0.271568 2.034754 1.277180
H -0.300531 2.951878 1.065465
H -0.441336 1.260810 1.584304
H 0.943108 2.239525 2.136200
H -4.887773 0.210208 -0.246061

int_B.gjf

0 1
C 2.732534 -0.192666 0.040725
C 2.389698 0.852506 -0.804108
C 1.082628 1.333442 -0.790482
C 0.147846 0.755463 0.061252
C 0.507426 -0.297372 0.906294
C 1.806876 -0.778010 0.900456
H 3.133844 1.285770 -1.471273
H 0.822171 2.146823 -1.463938
H -0.233906 -0.761770 1.557375
H 2.101305 -1.603041 1.547627
N -1.243727 1.265410 0.160197
C -1.378203 1.942230 1.488004
H -0.613714 2.726013 1.546760
H -2.374264 2.392542 1.557426
H -1.229960 1.219761 2.296512
C -2.209586 0.076501 -0.060357
C -3.173919 -0.179472 0.916172
C -2.007725 -0.610371 -1.251070
C -4.046938 -1.245167 0.689502
H -3.279612 0.400224 1.833997
C -2.925716 -1.671673 -1.412253
C -3.920524 -1.994032 -0.481110
H -4.817053 -1.481927 1.427795
H -2.875386 -2.297739 -2.315387
H -4.601644 -2.831461 -0.663966
N 4.112429 -0.699799 0.025427
O 4.898736 -0.178427 -0.736543
O 4.388010 -1.610138 0.777818
C -1.571806 2.279337 -0.887098
H -0.940610 3.166214 -0.758507
H -1.437095 1.813072 -1.867949
H -2.624502 2.550457 -0.751598

int_B_no_NO2.gjf

0 1
C 3.465295 -1.355168 0.179643
C 3.380137 -0.359583 -0.787726

C 2.251359 0.459485 -0.868871
C 1.206309 0.268856 0.027126
C 1.280436 -0.729076 1.000122
C 2.409167 -1.536862 1.074575
H 4.194888 -0.212365 -1.499788
H 2.207832 1.221018 -1.645055
H 0.448181 -0.890422 1.687659
H 2.458176 -2.320634 1.833328
N -0.000573 1.141859 0.027541
C 0.056234 1.977150 1.265916
H 0.996954 2.539755 1.250961
H -0.789886 2.672910 1.270660
H 0.027525 1.335562 2.151861
C -1.256658 0.245298 -0.082121
C -2.229514 0.353917 0.913802
C -1.284663 -0.581971 -1.199370
C -3.364391 -0.451278 0.797949
H -2.150027 1.025594 1.769291
C -2.459685 -1.364131 -1.250800
C -3.478328 -1.315585 -0.290905
H -4.147898 -0.397271 1.557984
H -2.607030 -2.063127 -2.087704
H -4.364362 -1.951510 -0.388137
C -0.042984 2.079651 -1.133890
H 0.812877 2.763611 -1.092712
H -0.052067 1.485657 -2.052950
H -0.976303 2.648499 -1.055559
H 4.348866 -1.994516 0.234673

7. X-ray Crystallography

All data collections, crystal structure determinations and refinements were done by the X-ray crystallography service (George F. S. Whitehead and Iñigo J. Vitorica-Yrezabal) at The University of Manchester.

Data collection

X-ray data was collected at a temperature of 100 K on a Rigaku® FR-X DW rotating anode diffractometer using CuKα radiation, (λ = 1.54184 Å) with an AFC-11 RINC goniometer and a Rigaku Hypix 6000 HE photon counting detector. The diffractometer was equipped with an Oxford Cryosystems® Cryostream 800 plus nitrogen flow gas system.

Crystal structure determinations and refinements

X-ray data were processed and reduced using CrysAlis^{Pro} suite of programs. The crystal structures were solved and refined against all F2 values using the SHELX and Olex 2 suite of programs. All the non-hydrogen atoms were refined anisotropically. Hydrogen atoms were placed in a calculated position refined using idealised geometries (riding model) and assigned fixed isotropic displacement parameters.

Data availability

Crystallographic data for **3ad** and **6a** have been deposited in the Cambridge Crystallographic Data Centre, with deposition numbers CCDC 2253673 (**1a**), 2253675 (**1b**), 2244918 (**3ad**), and 2244917 (**6a**), and are available free of charge via https://www.ccdc.cam.ac.uk/structures/.

X-ray structure of *N,N-dimethyl-4-nitroaniline (1a)*

CCDC Deposition Number: 2253673

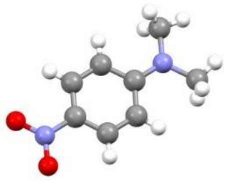

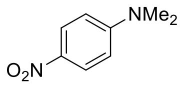

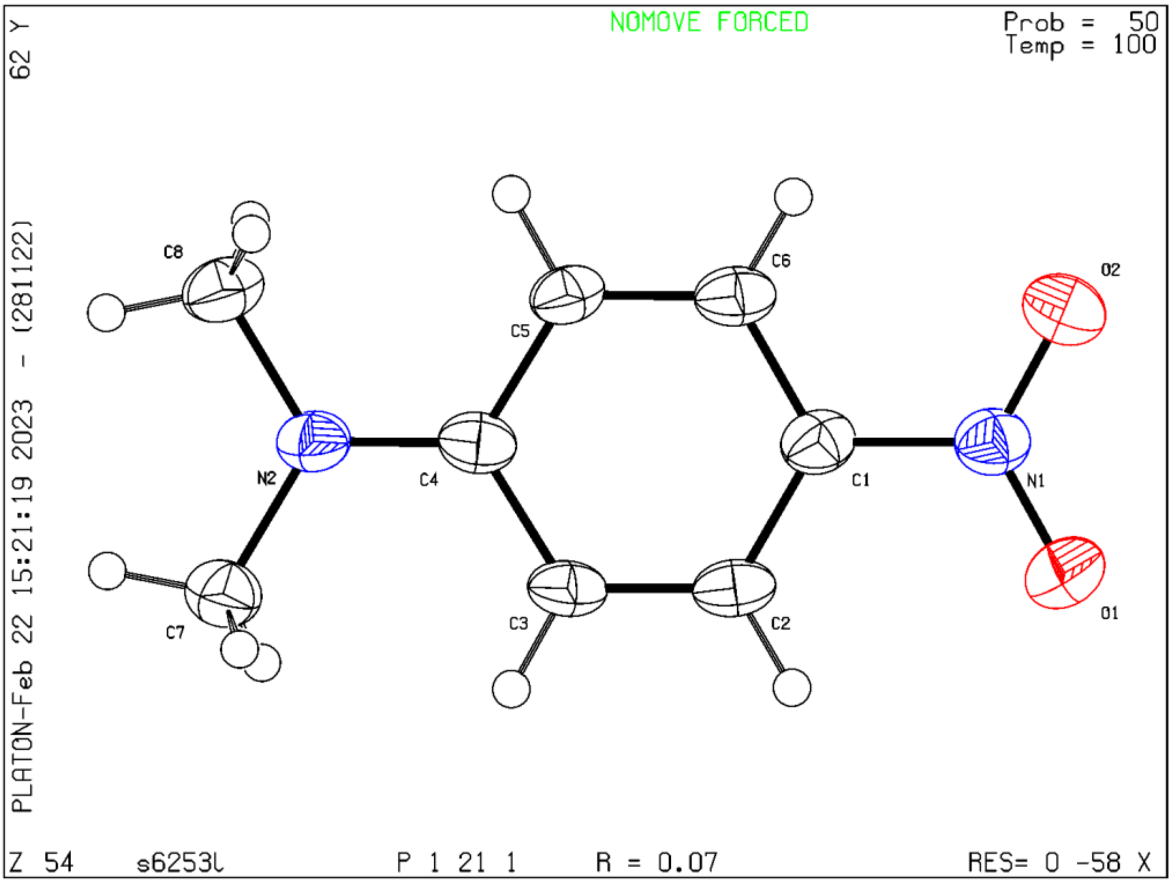

Empirical formula	$C_8H_{10}N_2O_2$
Formula weight	166.18
Temperature (K)	100
Space group	P 1 21 1
a (Å)	3.8776(4)
b (Å)	10.4790(8)
c (Å)	9.6055(8)

α (°)	90
β (°)	90.139(8)
γ (°)	90
Volume (Å^3)	390.30(6)
Z	2
P_{calc} (g/cm^3)	1.414
μ (mm^{-1})	0.861
F(000)	176.0
Radiation (Å)	CuKα (λ = 1.54184)
Independent reflections	1382
Data/parameters	1382/112
Goodness-of-fit on F^2	1.058
Final R indexes [I>=2σ (I)]	wR_2 = 0.2418
Final R indexes [all data]	R_1 = 0.0749

Table S12. Crystal data and structure refinement for 1a.

X-ray structure of *2-methoxy-N,N-dimethyl-4-nitroaniline (1b)*

CCDC Deposition Number: 2253675

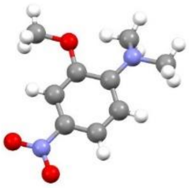

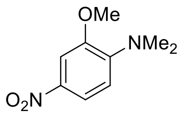

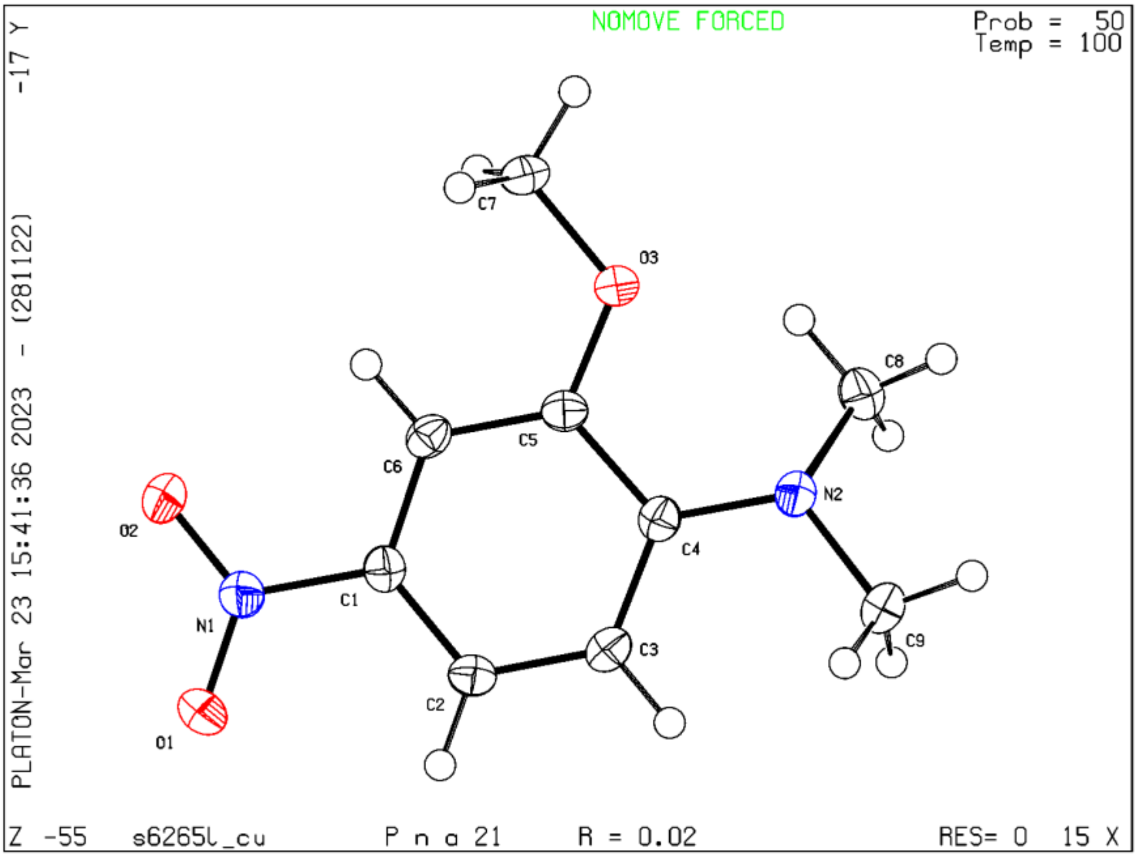

Empirical formula	$C_9H_{12}N_2O_3$
Formula weight	196.21
Temperature (K)	100
Space group	P n a 21
a (Å)	14.2446(3)
b (Å)	16.7350(3)
c (Å)	3.84405(9)
α (°)	90

β (°)	90
γ (°)	90
Volume (Å^3)	916.36(3)
Z	4
P_{calc} (g/cm^3)	1.422
μ (mm^{-1})	0.906
F(000)	416.0
Radiation (Å)	CuKα (λ = 1.54184)
Independent reflections	1583
Data/parameters	1583/131
Goodness-of-fit on F^2	1.085
Final R indexes [I>=2σ (I)]	wR_2 = 0.0683
Final R indexes [all data]	R_1 = 0.0246

Table S13. Crystal data and structure refinement for 1b.

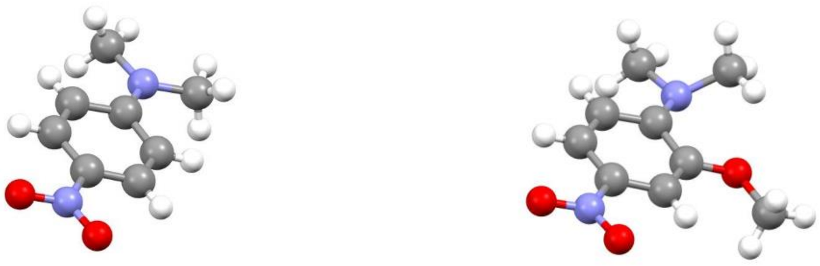

Figure S2. Comparison of the crystal structures of 1a and 1b. The presence of an o-methoxy substituent in **1b** forces the nitrogen lone pair out of conjugation with the aromatic ring, rendering **1b** more nucleophilic as observed.

X-ray structure of *2''-Methoxy-N,N-dimethyl-4''-nitro-[1,1':2',1''-terphenyl]-3'-amine (3ad)*

CCDC Deposition Number: 2244918

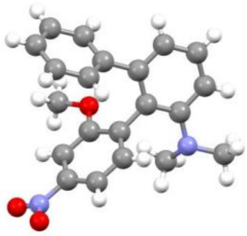

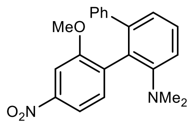

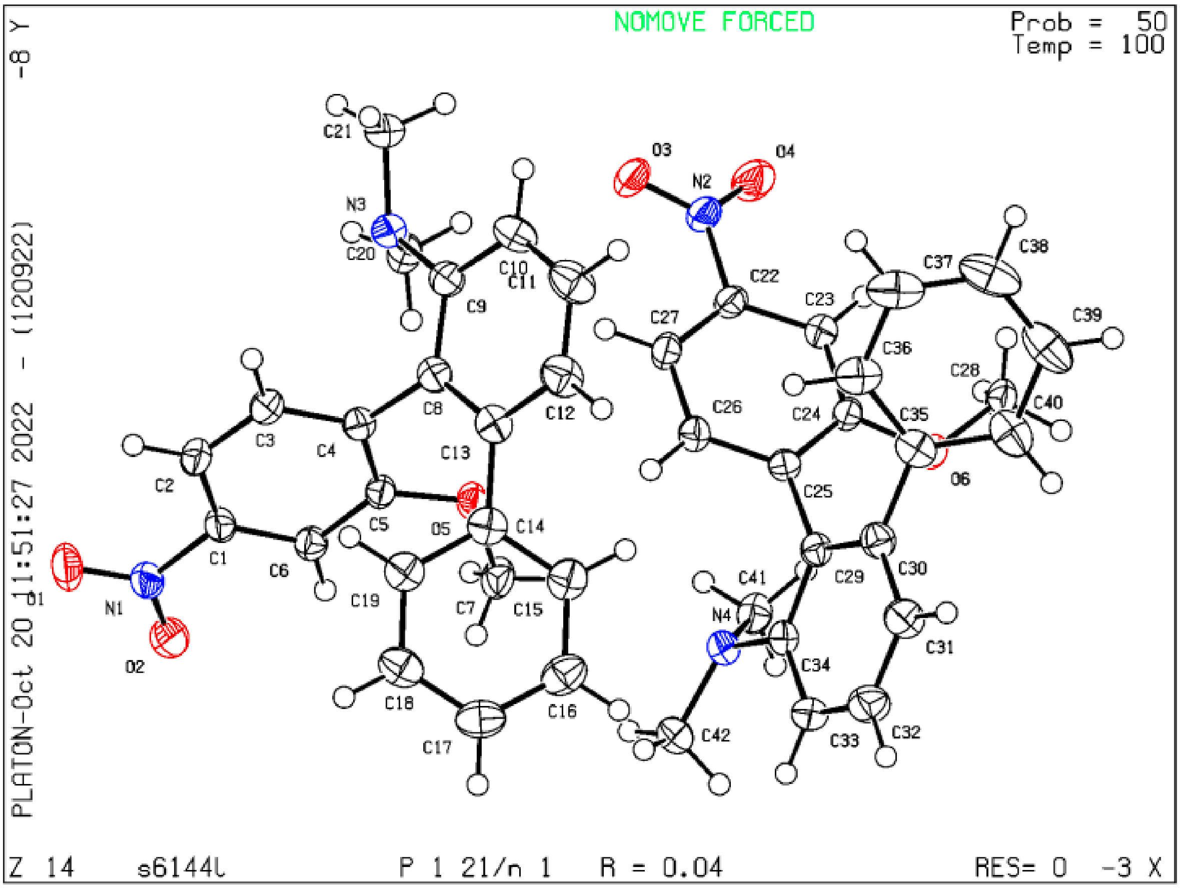

Empirical formula	$C_{21}H_{20}N_2O_3$
Formula weight	348.39
Temperature (K)	100
Space group	P 1 21/n 1
a (Å)	12.7014(2)
b (Å)	19.2279(4)
c (Å)	14.9543(3)
α (°)	90

β (°)	95.179(1)
γ (°)	90
Volume (Å^3)	3637.25(12)
Z	8
P_{calc} (g/cm^3)	1.272
μ (mm^{-1})	0.695
F(000)	1472.0
Radiation (Å)	CuKα (λ = 1.54184)
Independent reflections	7333
Data/parameters	7333/475
Goodness-of-fit on F^2	1.085
Final R indexes [I>=2σ (I)]	wR_2 = 0.1218
Final R indexes [all data]	R_1 = 0.0434

Table S14. Crystal data and structure refinement for 3ad.

X-ray structure of *5-Methyl-9-nitro-6,7-dihydro-5H-dibenzo[b,d]azepine (6a)*

CCDC Deposition Number: 2244917

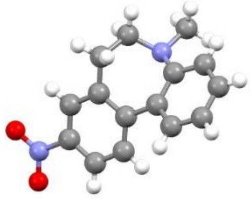

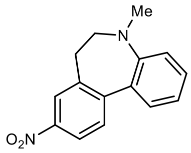

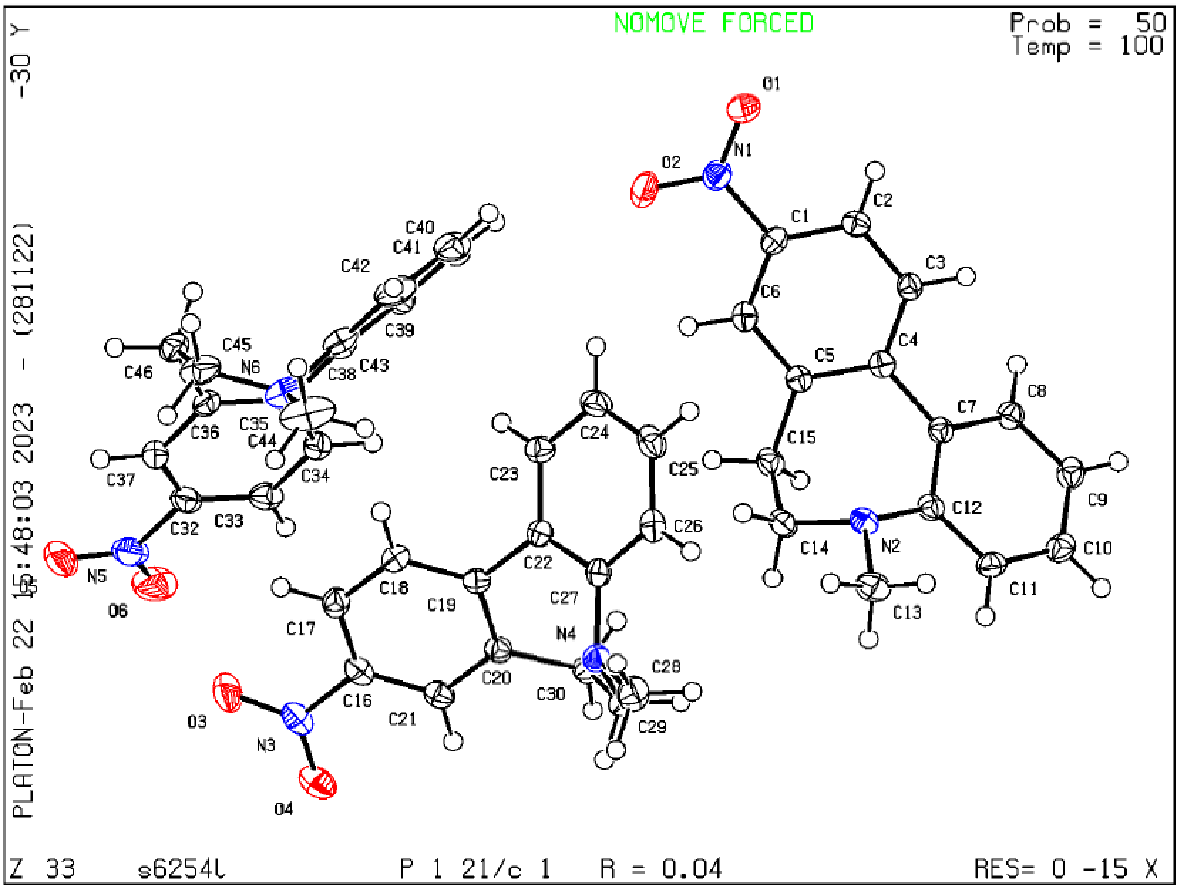

Empirical formula	$C_{15}H_{14}N_2O_2$
Formula weight	254.28
Temperature (K)	100
Space group	P 1 21/c 1
a (Å)	11.31591(18)
b (Å)	46.7564(6)
c (Å)	7.03327(10)

α (°)	90
β (°)	92.9757(13)
γ (°)	90
Volume (Å^3)	3716.22(9)
Z	12
P_{calc} (g/cm^3)	1.363
μ (mm^{-1})	0.746
F(000)	1608.0
Radiation (Å)	CuKα (λ = 1.54184)
Independent reflections	7699
Data/parameters	7699/518
Goodness-of-fit on F^2	1.059
Final R indexes [I>=2σ (I)]	wR_2 = 0.1149
Final R indexes [all data]	R_1 = 0.0428

Table S15. Crystal data and structure refinement for 6a.

8. Compound Characterisation

2-Methoxy-*N*,*N*-dimethyl-4-nitroaniline (1b)

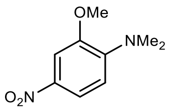

Prepared according to general procedure A (1.0 mmol scale). No column chromatography required. The pure product **1b** was afforded as an orange solid (170 mg, 87% yield).

^{1}H NMR (400 MHz, $CDCl_3$) δ 7.85 (dd, *J* = 8.9, 2.5 Hz, 1H), 7.69 (d, *J* = 2.5 Hz, 1H), 6.79 (d, *J* = 8.9 Hz, 1H), 3.94 (s, 3H), 2.97 (s, 6H).

^{13}C NMR (126 MHz, $CDCl_3$) δ 150.4, 148.4, 140.7, 118.3, 115.5, 106.8, 56.0, 42.7.

HRMS (ESI) Calculated for $[C_9H_{12}O_3N_2+Na]^+$ 219.0740, found 219.0737.

XRD: Recrystallised from acetonitrile as yellow-orange needles. Single crystal analysis confirms the structure drawn (CCDC deposition number: 2253675).

The data are in accordance with the literature.[7]

2-Ethoxy-N,N-dimethyl-4-nitroaniline (1c)

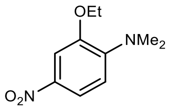

Prepared according to General Procedure B (2.5 mmol scale). Purified by column chromatography (0-15% EtOAc in hexane). The pure product **1c** was afforded as a yellow solid (378 mg, 71% yield).

^{1}H NMR ($CDCl_3$, 400 MHz) δ 7.83 (dd, *J* = 8.9, 2.6 Hz, 1H), 7.68 (d, *J* = 2.6 Hz, 1H), 6.77 (d, *J* = 8.9 Hz, 1H), 4.14 (q, *J* = 7.0 Hz, 2H), 3.00 (s, 6H), 1.51 (t, *J* = 7.0 Hz, 3H).

^{13}C NMR ($CDCl_3$, 101 MHz) δ 149.4, 148.4, 140.3, 118.1, 115.2, 107.6, 64.6, 42.6, 14.7.

HRMS (APCI) Calculated for $[C_{10}H_{14}O_3N_2]^+$ 210.0999, found 210.0999.

mp: 69-72 °C.

2-(Dimethylamino)-5-nitrophenol (1sm)

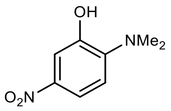

Prepared according to general procedure A (2.0 mmol scale). Purified by column chromatography (0-40% EtOAc in hexane). The pure product **1sm** was afforded as a red solid (153 mg, 42% yield).

^{1}H NMR (500 MHz, $CDCl_3$) δ 7.79 (dd, J = 8.6, 2.6 Hz, 1H), 7.76 (d, J = 2.6 Hz, 1H), 7.15 (d, J = 8.6 Hz, 1H), 6.46 (brs, 1H), 2.75 (s, 6H).

^{13}C NMR (126 MHz, $CDCl_3$) δ 150.7, 147.3, 144.8, 119.8, 116.4, 110.2, 44.2.

HRMS (ESI) Calculated for $[C_8H_{10}O_3N_2+Na]^+$ 205.0584, found 205.0593.

The data are in accordance with the literature.[8]

2-(Methoxymethoxy)-N,N-dimethyl-4-nitroaniline (1d)

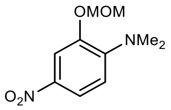

Prepared according to general procedure B (0.6 mmol scale). Purified by column chromatography (0-30% EtOAc in hexane). The pure product **1d** was afforded as a yellow oil (104 mg, 77% yield).

^{1}H NMR (400 MHz, $CDCl_3$) δ 7.95 (d, J = 2.6 Hz, 1H), 7.88 (dd, J = 9.0, 2.6 Hz, 1H), 6.81 (d, J = 9.0 Hz, 1H), 5.26 (s, 2H), 3.54 (s, 3H), 3.00 (s, 6H).

^{13}C NMR (101 MHz, $CDCl_3$) δ 149.0, 147.6, 140.6, 119.3, 115.8, 111.6, 95.5, 56.7, 42.7.

HRMS (ESI) Calculated for $[C_{10}H_{14}O_4N_2+Na]$ 249.0846, found 249.0851.

4-(2-(Dimethylamino)-5-nitrophenoxy)butyl acetate (1e)

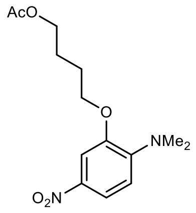

Prepared according to general procedure B (1.0 mmol scale). Purified by column chromatography (0-30% EtOAc in hexane). The pure product **1e** was afforded as an orange solid (189 mg, 64% yield).

^{1}H NMR (400 MHz, $CDCl_3$) δ 7.84 (dd, *J* = 8.9, 2.5 Hz, 1H), 7.67 (d, *J* = 2.5 Hz, 1H), 6.76 (d, *J* = 8.9 Hz, 1H), 4.16 (t, *J* = 6.3 Hz, 2H), 4.09 (t, *J* = 6.2 Hz, 2H), 2.99 (s, 6H), 2.06 (s, 3H), 2.00 – 1.91 (m, 2H), 1.91 – 1.82 (m, 2H).

^{13}C NMR (101 MHz, $CDCl_3$) δ 171.3, 149.4, 148.4, 140.4, 118.4, 115.3, 107.7, 68.6, 64.1, 42.7, 26.0, 25.7, 21.1.

HRMS (APCI) Calculated for $[C_{14}H_{20}O_5N_2]^+$ 297.1445, found 297.1441.

mp: 56-58 °C

2-(2-(1,3-Dioxolan-2-yl)ethoxy)-N,N-dimethyl-4-nitroaniline (1f)

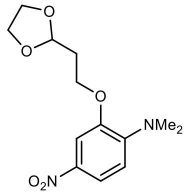

Prepared according to general procedure B (1.0 mmol scale). Purified by column chromatography (0-40% EtOAc in hexane). The pure product **1f** was afforded as an orange oil (178 mg, 63% yield).

^{1}H NMR (400 MHz, $CDCl_3$) δ 7.84 (dd, *J* = 8.9, 2.5 Hz, 1H), 7.71 (d, *J* = 2.5 Hz, 1H), 6.75 (d, *J* = 8.9 Hz, 1H), 5.12 (t, *J* = 4.7 Hz, 1H), 4.21 (t, *J* = 6.6 Hz, 2H), 4.08 – 3.95 (m, 2H), 3.95 – 3.82 (m, 2H), 2.99 (s, 6H), 2.30 – 2.14 (m, 2H).

^{13}C NMR (101 MHz, $CDCl_3$) δ 149.3, 148.4, 140.4, 118.4, 115.3, 107.9, 102.1, 65.2, 64.8, 42.7, 33.8.

HRMS (APCI) Calculated for $[C_{13}H_{18}O_5N_2+H]^+$ 283.1288, found 283.1285.

2-(Allyloxy)-N,N-dimethyl-4-nitroaniline (1g)

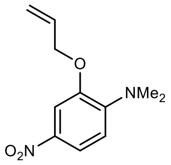

Prepared according to General Procedure B (2.0 mmol scale). Purified by column chromatography (0-35% EtOAc in hexane). The pure product **1g** was afforded as an orange solid (266 mg, 60% yield).

^{1}H NMR ($CDCl_3$, 400 MHz) δ 7.85 (dd, *J* = 8.9, 2.5 Hz, 1H), 7.69 (d, *J* = 2.5 Hz, 1H), 6.78 (d, *J* = 8.9 Hz, 1H), 6.10 (ddt, *J* = 17.1, 10.5, 5.3 Hz, 1H), 5.47 (dq, *J* = 17.2, 1.6 Hz, 1H), 5.34 (dq, *J* = 10.5, 1.4 Hz, 1H), 4.64 (dt, *J* = 5.4, 1.5 Hz, 2H), 3.00 (s, 6H).

^{13}C NMR ($CDCl_3$, 101 MHz) δ 149.0, 148.6, 140.4, 132.4, 118.54, 118.50, 115.5, 108.4, 69.9, 42.7.

HRMS (APCI) Calculated for $[C_{11}H_{14}O_3N_2+H]^+$ 223.1077, found 223.1079.

2-Fluoro-*N*,*N*-dimethyl-4-nitroaniline (1h)

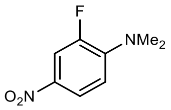

Prepared according to general procedure A (2.0 mmol scale). No column chromatography required. The pure product **1h** was afforded as a yellow solid (431 mg, quant. yield).

^{1}H NMR (500 MHz, $CDCl_3$) δ 7.96 – 7.90 (m, 1H), 7.86 (dd, *J* = 14.2, 2.6 Hz, 1H), 6.71 (t, *J* = 9.1 Hz, 1H), 3.09 (d, *J* = 2.2 Hz, 6H).

^{13}C NMR (126 MHz, $CDCl_3$) δ 151.0 (d, *J* = 246.5 Hz), 145.5 (d, *J* = 7.7 Hz), 138.1 (d, *J* = 8.4 Hz), 121.5 (d, *J* = 2.4 Hz), 114.8 (d, *J* = 4.9 Hz), 112.9 (d, *J* = 26.8 Hz), 42.3 (d, *J* = 6.4 Hz).

^{19}F NMR (471 MHz, $CDCl_3$) δ -121.8 – -122.0 (m).

HRMS (ESI) Calculated for $[C_8H_9O_2N_2F+Na]^+$ 207.0540, found 207.0539.

The data are in accordance with the literature.[9]

N,N-Diethyl-2-methoxy-4-nitroaniline (1i)

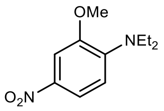

Prepared according to general procedure C (2 mmol scale). Purified by column chromatography (0-30% EtOAc in hexane). The pure product **1i** was afforded as a yellow oil (200 mg, 45% yield).

^{1}H NMR (400 MHz, $CDCl_3$) δ 7.82 (dd, *J* = 9.0, 2.6 Hz, 1H), 7.67 (d, *J* = 2.6 Hz, 1H), 6.75 (d, *J* = 9.0 Hz, 1H), 3.90 (s, 3H), 3.37 (q, *J* = 7.1 Hz, 4H), 1.15 (t, *J* = 7.0 Hz, 6H).

^{13}C NMR (101 MHz, $CDCl_3$) δ 150.2, 146.4, 139.7, 118.3, 115.9, 107.1, 56.0, 46.0, 12.9.

HRMS (ESI) calculated for $[C_{11}H_{16}O_3N_{2+}Na]^+$ 247.1053, found 247.1056.

***N*-Ethyl-2-methoxy-*N*-methyl-4-nitroaniline (1j)**

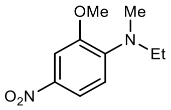

Prepared according to general procedure C (2.0 mmol scale). Purified by column chromatography (0-30% EtOAc in hexane). The pure product **1j** was afforded as a yellow oil (53 mg, 13% yield).

^{1}H NMR (500 MHz, $CDCl_3$) δ 7.85 (dd, *J* = 8.9, 2.6 Hz, 1H), 7.69 (d, *J* = 2.5 Hz, 1H), 6.77 (d, *J* = 8.9 Hz, 1H), 3.93 (s, 3H), 3.37 (q, *J* = 7.1 Hz, 2H), 2.93 (s, 3H), 1.16 (t, *J* = 7.1 Hz, 3H).

^{13}C NMR (126 MHz, $CDCl_3$) δ 150.3, 147.8, 140.3, 118.4, 115.7, 106.9, 56.1, 49.2, 39.3, 12.7.

HRMS (ESI) Calculated for $[C_{10}H_{14}O_3N_2+Na]^+$ 233.0897, found 233.0891.

The data are in accordance with the literature.[10]

N-Benzyl-2-methoxy-N-methyl-4-nitroaniline (1k)

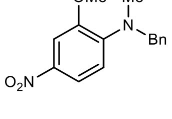

Prepared according to General Procedure C (2.0 mmol scale). Purified by column chromatography (0-20% EtOAc in hexane). The pure product **1k** was afforded as an orange solid (486 mg, 71% yield).

^{1}H NMR (CDCl$_3$, 400 MHz) δ 7.83 (dd, J = 8.8, 2.5 Hz, 1H), 7.72 (d, J = 2.5 Hz, 1H), 7.34 – 7.28 (m, 2H), 7.28 – 7.22 (m, 3H), 6.76 (d, J = 8.9 Hz, 1H), 4.50 (s, 2H), 3.92 (s, 3H), 2.85 (s, 3H).

^{13}C NMR (CDCl$_3$, 126 MHz) δ 150.3, 147.6, 140.7, 138.2, 128.6, 128.1, 127.4, 118.4, 116.1, 106.9, 58.4, 56.1, 39.5.

HRMS (APCI) Calculated for $[C_{15}H_{16}O_2N_2+H]^+$ 273.1234, found 273.1234.

mp: 54-57 °C.

N-(2-Methoxy-4-nitrophenyl)-N-methyltetrahydro-2H-pyran-4-amine (1l)

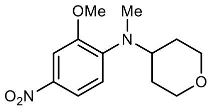

Prepared according to General Procedure C (2.0 mmol scale). Purified by column chromatography (0-20% EtOAc in hexane). The pure product **1l** was afforded as a yellow-orange solid (274 mg, 51% yield).

^{1}H NMR (CDCl$_3$, 400 MHz) δ 7.81 (dd, J = 8.9, 2.5 Hz, 1H), 7.68 (d, J = 2.5 Hz, 1H), 6.82 (d, J = 8.9 Hz, 1H), 4.03 (dd, J = 11.3, 4.4 Hz, 2H), 3.91 (s, 3H), 3.79 (tt, J = 11.8, 4.0 Hz, 1H), 3.39 (td, J = 11.9, 2.0 Hz, 2H), 2.82 (s, 3H), 1.89 (qd, J = 12.2, 4.6 Hz, 2H), 1.64 (qqd, J = 12.3, 4.1, 1.9 Hz, 2H).

^{13}C NMR (CDCl$_3$, 101 MHz) δ 150.8, 147.9, 140.8, 118.0, 117.1, 106.9, 67.8, 58.1, 56.1, 33.4, 30.2.

mp: 97-100 °C.

HRMS (APCI) Calculated for $[C_{13}H_{18}O_4N_2+H]^+$ 267.1339, found 267.1330.

1-(2-Methoxy-4-nitrophenyl)piperidine (1m)

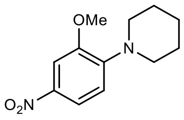

Prepared according to general procedure C (2.0 mmol scale). No column chromatography required. The pure product **1m** was afforded as a yellow solid (412 mg, 87% yield).

^{1}H NMR (400 MHz, CDCl$_3$) δ 7.85 (dd, J = 8.8, 2.5 Hz, 1H), 7.69 (d, J = 2.5 Hz, 1H), 6.88 (d, J = 8.9 Hz, 1H), 3.94 (s, 3H), 3.20 – 3.13 (m, 4H), 1.79 – 1.69 (m, 4H), 1.67 – 1.57 (m, 2H).

^{13}C NMR (101 MHz, CDCl$_3$) δ 151.3, 148.7, 141.7, 118.0, 116.8, 106.6, 56.1, 51.6, 26.1, 24.4.

HRMS (APCI) Calculated for $[C_{12}H_{16}O_3N_2+H]^+$ 237.1234, found 237.1226.

The data are in accordance with the literature.[11]

4-(2-Methoxy-4-nitrophenyl)morpholine (1n)

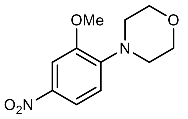

Prepared according to General Procedure C (2.0 mmol scale). Purified by column chromatography (5-30% EtOAc in hexane). The pure product **1n** was afforded as a yellow solid (468 mg, 96% yield).

^{1}H NMR (CDCl$_3$, 400 MHz) δ 7.86 (dd, J = 8.8, 2.5 Hz, 1H), 7.71 (d, J = 2.5 Hz, 1H), 6.88 (d, J = 8.8 Hz, 1H), 3.95 (s, 3H), 3.91 – 3.84 (m, 4H), 3.26 – 3.18 (m, 4H).

^{13}C NMR (CDCl$_3$, 101 MHz) δ 151.4, 147.2, 142.4, 117.9, 116.6, 106.7, 66.9, 56.1, 50.5.

HRMS (APCI) Calculated for $[C_{11}H_{14}O_2N_2+H]^+$ 239.1026, found 239.1030.

The data are in accordance with the literature.[12]

1-(2-Methoxy-4-nitrophenyl)azepane (1o)

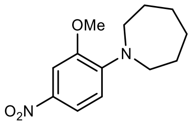

Prepared according to General Procedure C (2.0 mmol scale). No column chromatography required. The pure product **1o** was afforded as a brown oil (503 mg, 93% yield).

^{1}H NMR (CDCl$_3$, 500 MHz) δ 7.85 – 7.78 (m, 1H), 7.66 (d, J = 3.2 Hz, 1H), 6.71 (d, J = 9.1 Hz, 1H), 3.88 (s, 3H), 3.51 (t, J = 5.8 Hz, 4H), 1.83 (t, J = 6.5 Hz, 4H), 1.67 – 1.55 (m, 4H).

^{13}C NMR (CDCl$_3$, 126 MHz) δ 148.9, 147.8, 138.5, 119.0, 113.8, 107.4, 56.0, 52.7, 29.0, 27.2.

HRMS (APCI) Calculated for $[C_{13}H_{18}O_3N_2+H]^+$ 251.1390, found 251.1379.

4-(2-Methoxy-4-nitrophenyl)-1,4-oxazepane (1p)

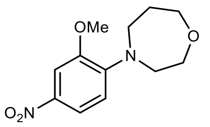

Prepared according to general procedure C (2.0 mmol scale). Purified by column chromatography (0-40% EtOAc in petroleum ether). The pure product **1p** was afforded as a brown oil (382 mg, 76% yield).

^{1}H NMR (500 MHz, CDCl$_3$) δ 7.82 (dd, *J* = 8.9, 2.5 Hz, 1H), 7.68 (d, *J* = 2.6 Hz, 1H), 6.77 (d, *J* = 9.0 Hz, 1H), 3.89 (s, 3H), 3.88 – 3.85 (m, 2H), 3.84 – 3.79 (m, 2H), 3.63 – 3.58 (m, 2H), 3.56 (t, *J* = 6.2 Hz, 2H), 2.10 – 2.04 (m, 2H).

^{13}C NMR (126 MHz, CDCl$_3$) δ 149.6, 147.5, 139.8, 118.5, 115.1, 107.2, 71.0, 69.6, 56.0, 55.0, 51.2, 30.6.

HRMS (APCI) Calculated for $[C_{12}H_{16}O_4N_2+H]^+$ 253.1183, found 253.1183.

1-(2-Methoxy-4-nitrophenyl)-3-methylpiperidine (1q)

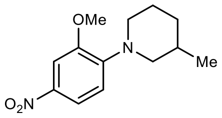

Prepared according to General Procedure C (3.0 mmol scale). No column chromatography required. The pure product **1p** was afforded as a yellow solid (735 mg, 98% yield).

^{1}H NMR (CDCl$_3$, 400 MHz) δ 7.84 (dd, *J* = 8.8, 2.5 Hz, 1H), 7.69 (d, *J* = 2.5 Hz, 1H), 6.87 (d, *J* = 8.9 Hz, 1H), 3.93 (s, 3H), 3.66 – 3.58 (m, 1H), 3.54 (ddt, *J* = 11.7, 3.7, 1.9 Hz, 1H), 2.73 – 2.58 (m, 1H), 2.37 (dd, *J* = 11.8 Hz, 10.1, 1H), 1.91 – 1.66 (m, 4H), 1.17 – 1.01 (m, 1H), 0.94 (d, *J* = 6.4 Hz, 3H).

^{13}C NMR (CDCl$_3$, 101 MHz) δ 151.3, 148.5, 141.6, 118.1, 116.9, 106.7, 58.4, 56.1, 51.0, 33.0, 31.3, 25.6, 19.5.

HRMS (APCI) Calculated for $[C_{13}H_{18}O_3N_2+H]^+$ 251.1390, found 251.1393.

mp: 100-103 °C.

1-(2-Methoxy-4-nitrophenyl)-4-phenylpiperidine (1r)

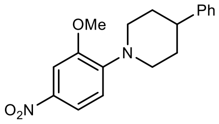

Prepared according to General Procedure C (2.0 mmol scale). No column chromatography required. The pure product **1r** was afforded as a yellow-orange solid (628 mg, quant. yield).

^{1}H NMR ($CDCl_3$, 400 MHz) δ 7.85 (dd, J = 8.8, 2.5 Hz, 1H), 7.70 (d, J = 2.5 Hz, 1H), 7.35 – 7.27 (m, 2H), 7.27 – 2.23 (m, 2H), 7.21 (tt, J = 7.2, 1.4 Hz, 1H), 6.94 (d, J = 8.8 Hz, 1H), 3.97 (s, 3H), 3.86 – 3.78 (m, 2H), 2.84 (ddt, J = 14.3, 10.8, 7.1 Hz, 2H), 2.76 – 2.64 (m, 1H), 2.04 – 1.92 (m, 4H).

^{13}C NMR ($CDCl_3$, 101 MHz) δ 151.4, 148.2, 145.9, 142.0, 128.7, 127.0, 126.5, 118.0, 117.0, 106.6, 56.1, 51.3, 42.6, 33.5.

HRMS (HESI) Calculated for $[C_{18}H_{20}O_3N_2+H]^+$ 313.1533, found 313.1543.

mp: 128-130 °C.

4-Methoxy-1-(2-methoxy-4-nitrophenyl)piperidine (1s)

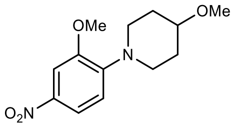

Prepared according to General Procedure C (2.0 mmol scale). No column chromatography required. The pure product **1s** was afforded as a yellow solid (509 mg, 96% yield).

^{1}H NMR ($CDCl_3$, 400 MHz) δ 7.84 (dd, J = 8.8, 2.5 Hz, 1H), 7.69 (d, J = 2.5 Hz, 1H), 6.89 (d, J = 8.8 Hz, 1H), 3.94 (s, 3H), 3.54 – 3.43 (m, 2H), 3.42 (p, J = 4.0 Hz, 1H), 3.38 (s, 3H), 3.04 – 2.92 (m, 2H), 2.10 – 1.96 (m, 2H), 1.77 (dtd, J = 12.5, 8.4, 3.6 Hz, 2H).

^{13}C NMR ($CDCl_3$, 101 MHz) δ 151.1, 147.8, 141.6, 117.7, 116.7, 106.4, 75.4, 55.9, 55.6, 47.6, 30.7.

HRMS (APCI) Calculated for $[C_{13}H_{18}O_4N_2+H]^+$ 267.1339, found 267.1347.

mp: 85-87 °C.

4-Bromo-1-(2-methoxy-4-nitrophenyl)piperidine (1t)

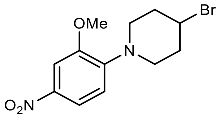

Prepared according to General Procedure C (2.0 mmol scale). Purified by column chromatography (0-10% EtOAc in hexane). The pure product **1t** was afforded as an orange solid (443 mg, 70% yield).

^{1}H NMR ($CDCl_3$, 400 MHz) δ 7.77 (dd, J = 8.8, 2.5 Hz, 1H), 7.63 (d, J = 2.6 Hz, 1H), 6.84 (dd, J = 8.8 Hz, 1H), 4.35 (tt, J = 7.9, 3.9 Hz, 1H), 3.90 (s, 3H), 3.43 (ddd, J = 11.3, 6.8, 3.5 Hz, 2H), 3.05 (ddd, J = 11.9, 8.0, 3.3 Hz, 2H), 2.32 – 2.21 (m, 2H), 2.18 – 2.05 (m, 2H).

^{13}C NMR ($CDCl_3$, 101 MHz) δ 151.0, 147.3, 141.8, 117.6, 116.8, 106.3, 55.9, 49.4, 48.6, 35.9.

HRMS (ESI) Calculated for $[C_{12}H_{15}O_3N_2Br+H]^+$ 315.0339, found 315.0344.

mp: 93-96 °C.

1-(2-Methoxy-4-nitrophenyl)-4-methylpiperidine (1u)

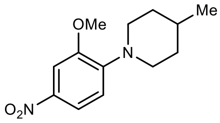

Prepared according to General Procedure C (2.0 mmol scale). Purified by column chromatography (0-10% EtOAc in hexane). The pure product **1u** was afforded as a yellow-orange solid (481 mg, 96% yield).

^{1}H NMR ($CDCl_3$, 400 MHz) δ 7.84 (ddd, J = 8.8, 2.5, 1.1 Hz, 1H), 7.68 (dd, J = 2.6, 1.0 Hz, 1H), 6.88 (d, J = 8.8 Hz, 1H), 3.94 (s, 3H), 3.70 – 3.59 (m, 2H), 2.70 (td, J = 12.0, 2.4 Hz, 2H), 1.79-1.70 (dd, J = 12.9, 3.0 Hz, 2H), 1.63 – 1.49 (m, 1H), 1.41 (qd, J = 12.0, 3.9 Hz, 2H), 0.99 (d, J = 6.5 Hz, 3H).

^{13}C NMR ($CDCl_3$, 101 MHz) δ 151.3, 148.5, 141.6, 118.0, 116.9, 106.6, 56.1, 50.9, 34.3, 30.8, 22.0.

HRMS (ESI) Calculated for $[C_{13}H_{18}O_3N_2+H]^+$ 275.1396, found 251.1399.

mp: 64-67 °C.

1-(2-Methoxy-4-nitrophenyl)piperidine-4-carbonitrile (1v)

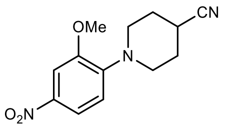

Prepared according to General Procedure C (2.0 mmol scale). Purified by column chromatography (0-50% EtOAc in hexane). The pure product **1v** was afforded as a yellow solid (307 mg, 58% yield).

^{1}H NMR ($CDCl_3$, 500 MHz) δ 7.86 (dd, J = 8.8, 2.5 Hz, 1H), 7.72 (d, J = 2.5 Hz, 1H), 6.90 (d, J = 8.8 Hz, 1H), 3.95 (s, 3H), 3.46 – 3.32 (m, 2H), 3.18 – 3.05 (m, 2H), 2.86 (tt, J = 7.9, 4.3 Hz, 1H), 2.20 – 1.98 (m, 4H).

^{13}C NMR ($CDCl_3$, 126 MHz) δ 151.5, 147.3, 142.6, 121.4, 117.8, 117.3, 106.7, 56.2, 48.5, 28.9, 26.2.

HRMS (APCI) Calculated for $[C_{13}H_{15}O_3N_3+H]^+$ 262.1186, found 262.1181.

mp: 110-113 °C.

Methyl 1-(2-methoxy-4-nitrophenyl)piperidine-4-carboxylate (1w)

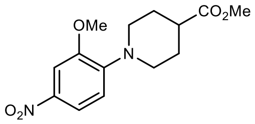

Prepared according to general procedure C (2.0 mmol scale). No column chromatography required. The pure product **1w** was afforded as a yellow solid (499 mg, 85% yield).

^{1}H NMR (500 MHz, $CDCl_3$) δ 7.83 (dd, J = 8.8, 2.5 Hz, 1H), 7.68 (d, J = 2.5 Hz, 1H), 6.86 (d, J = 8.8 Hz, 1H), 3.93 (s, 3H), 3.70 (s, 3H), 3.66 – 3.58 (m, 2H), 2.85 – 2.76 (m, 2H), 2.55 – 2.45 (m, 1H), 2.11 – 1.99 (m, 2H), 1.98 – 1.88 (m, 2H).

^{13}C NMR (126 MHz, $CDCl_3$) δ 175.2, 151.4, 147.9, 142.0, 117.9, 117.0, 106.7, 56.1, 51.9, 49.8, 40.8, 28.3.

HRMS (ESI) Calculated for $[C_{14}H_{18}O_5N_2+Na]^+$ 317.1108, found 317.1101.

mp: 82-84 °C.

8-(2-Methoxy-4-nitrophenyl)-1,4-dioxa-8-azaspiro[4.5]decane (1x)

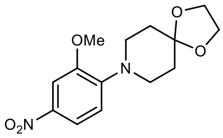

Prepared according to general procedure C (2.0 mmol scale). No column chromatography required. The pure product **1x** was afforded as a yellow solid (532 mg, 86% yield).

^{1}H NMR (500 MHz, CDCl$_3$) δ 7.84 (dd, J = 8.8, 2.6 Hz, 1H), 7.70 (d, J = 2.5 Hz, 1H), 6.90 (d, J = 8.8 Hz, 1H), 4.00 (s, 4H), 3.95 (s, 3H), 3.34 – 3.28 (m, 4H), 1.92 – 1.86 (m, 4H).

^{13}C NMR (126 MHz, CDCl$_3$) δ 151.3, 147.7, 142.0, 117.9, 117.2, 106.8, 106.7, 64.5, 56.1, 48.5, 35.2.

HRMS (APCI) Calculated for [$C_{14}H_{18}O_5N_2$+H] 295.1288, found 295.1285.

mp: 134-136 °C.

Cis- and trans-2-(2-Methoxy-4-nitrophenyl)decahydroisoquinoline (1y)

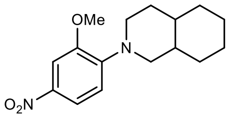

Prepared according to General Procedure C (2.0 mmol scale). Purified by column chromatography (0-5% EtOAc in hexane). The inseparable mixture of **cis-** and **trans-1x** was afforded as a yellow solid (428 mg, 74% yield).

^{1}H NMR (CDCl$_3$, 400 MHz) δ 7.85 (dd, J = 8.8, 2.5 Hz, 1H), 7.69 (d, J = 2.5 Hz, 1H), 6.89 (d, J = 4.7 Hz, 1H), 3.94 (s, 3H), 3.73 (ddq, J = 11.9, 4.5, 2.3 Hz, 1H), 3.52 (ddd, J = 11.7, 3.8, 2.2 Hz, 1H), 2.73 (t, J = 11.8 Hz, 1H), 2.39 (t, J = 11.3 Hz, 1H), 1.84 – 1.72 (m, 2H), 1.72 – 1.57 (m, 3H), 1.53 – 1.24 (m, 4H), 1.17 – 0.93 (m, 3H).

^{13}C NMR (CDCl$_3$, 101 MHz) δ 151.2, 148.4, 141.5, 118.1, 116.9, 106.7, 57.0, 56.1, 51.4, 42.1, 42.0, 33.1, 33.0, 30.4, 26.5, 26.1.

HRMS (ESI) Calculated for [$C_{16}H_{22}O_3N_2$+H]$^+$ 291.1703, found 291.1702.

mp: 130-131 °C.

6,7-Dimethoxy-2-(2-methoxy-4-nitrophenyl)-1,2,3,4-tetrahydroisoquinoline (1z)

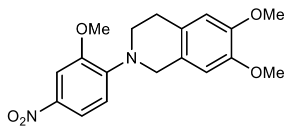

Prepared according to General Procedure C (2.0 mmol scale). No column chromatography required. The pure product **1z** was afforded as an orange-yellow solid (193 mg, 28% yield).

^{1}H NMR (CDCl$_3$, 400 MHz) δ 7.85 (dd, J = 8.9, 2.5 Hz, 1H), 7.73 (d, J = 2.5 Hz, 1H), 6.92 (d, J = 8.9 Hz, 1H), 6.62 (d, J = 7.0 Hz, 2H), 4.37 (s, 2H), 3.97 (s, 3H), 3.87 (s, 3H), 3.86 (s, 3H), 3.60 (t, J = 5.8 Hz, 2H), 2.89 (t, J = 5.8 Hz, 2H).

^{13}C NMR (CDCl$_3$, 101 MHz) δ 151.1, 147.8, 147.6, 147.1, 141.6, 126.2, 125.6, 117.9, 116.7, 111.6, 109.1, 106.6, 56.1, 56.00, 55.95, 51.9, 48.4, 28.3.

HRMS (APCI) Calculated for $[C_{18}H_{20}O_5N_2+H]^+$ 345.1445, found 345.1450.

mp: 136-139 °C.

4-Bromo-2-isopropyl-6-(trimethylsilyl)phenyl trifluoromethanesulfonate (2ab)

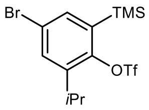

Prepared according to a literature procedure.[8] Isolated as a colourless solid.

^{1}H NMR (CDCl$_3$, 400 MHz) δ 7.46 (d, J = 2.6 Hz, 1H), 7.49 (d, J = 2.6 Hz, 1H), 3.25 (hept, J = 6.8 Hz, 1H), 1.23 (d, J = 6.8 Hz, 6H), 0.38 (s, 9H).

^{13}C NMR (CDCl$_3$, 101 MHz) δ 147.9, 144.6, 138.2, 137.1, 132.3, 123.0, 118.7 (q, J = 320.5 Hz), 27.5, 23.5, 0.25.

^{19}F NMR (CDCl$_3$, 376 MHz) δ -73.4.

HRMS (APCI) Calculated for $[C_{13}H_{18}O_3F_3BrSSi-Me]^+$ 402.9641, found 402.9632.

The data are in accordance with the literature.[13]

3-(Trimethylsilyl)-[1,1'-biphenyl]-2-yl trifluoromethanesulfonate (2ad)

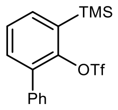

Prepared according to a literature procedure.[9] Isolated as a colourless solid.

^{1}H NMR (CDCl$_3$, 400 MHz) δ 7.58 – 7.53 (m, 1H), 7.45 – 7.35 (m, 7H), 0.45 (s, 9H).

^{13}C NMR (CDCl$_3$, 101 MHz) δ 149.9, 137.0, 136.4, 136.00, 135.95, 133.7, 129.8, 128.5, 128.2, 128.1, 118.1 (q, *J* = 320.6 Hz), 0.36.

^{19}F NMR (CDCl$_3$, 376 MHz) δ -73.9.

HRMS (APCI) Calculated for [$C_{16}H_{17}O_3F_3SSi$-Me]$^+$ 359.0380, found 359.0368.

The data are in accordance with the literature.[14]

4-Fluoro-2-(trimethylsilyl)phenyl trifluoromethanesulfonate (2af)

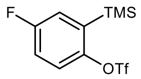

Prepared according to a literature procedure.[8] Isolated as a colourless oil.

^{1}H NMR (CDCl$_3$, 400 MHz) δ 7.31 (dd, *J* = 9.0, 4.0 Hz, 1H), 7.19 (d, *J* = 8.1, 3.2 Hz, 1H), 7.10 (ddd, *J* = 9.0, 7.3, 3.2 Hz, 1H), 0.37 (s, 9H).

^{13}C NMR (CDCl$_3$, 101 MHz) δ 161.2 (d, *J* = 249.7 Hz), 150.3 (d, *J* = 2.5 Hz), 136.1 (d, *J* = 4.7 Hz), 122.5 (d, *J* = 22.0 Hz), 121.6 (dq, *J* = 8.3, 1.5 Hz), 118.6 (q, *J* = 320.7 Hz), 117.9 (d, *J* = 24.2 Hz), -0.9.

^{19}F NMR (CDCl$_3$, 376 MHz) δ -73.9 (s), -114.4 (td, *J* = 7.7, 4.0 Hz).

HRMS (APCI) Calculated for [$C_{10}H_{12}O_3F_4SSi$-Me]$^+$ 300.9972, 300.9971.

The data are in accordance with the literature.[15]

4-Chloro-2-(trimethylsilyl)phenyl trifluoromethanesulfonate (2ag)

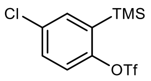

Prepared according to a literature procedure.[8] Isolated as a colourless oil.

^{1}H NMR (CDCl$_3$, 500 MHz) δ 7.43 (d, J = 2.7 Hz, 1H), 7.37 (dd, J = 8.8, 2.7 Hz, 1H), 7.24 (d, J = 3.5 Hz, 1H), 0.35 (s, 9H).

^{13}C NMR (CDCl$_3$, 101 MHz) δ 153.2, 136.0, 135.5, 133.7, 131.1, 121.1 (q, J = 1.4 Hz), 118.6 (q, J = 318.1 Hz), -0.9.

^{19}F NMR (CDCl$_3$, 376 MHz) δ -73.8.

HRMS (ESI, APCI) not observed.

The data are in accordance with the literature.[16]

4-Bromo-2-(trimethylsilyl)phenyl trifluoromethanesulfonate (2ah)

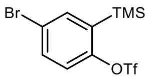

Prepared according to a literature procedure.[8] Isolated as a colourless oil.

^{1}H NMR (CDCl$_3$, 400 MHz) δ 7.60 (d, J = 2.5 Hz, 1H), 7.54 (dd, J = 8.8, 2.6 Hz, 1H), 7.22 (d, J = 8.9 Hz, 1H), 0.37 (s, 9H).

^{13}C NMR (CDCl$_3$, 101 MHz) δ 153.9, 139.0, 135.9, 134.2, 121.8, 121.5 (q, J = 1.5 Hz), 118.6 (q, J = 320.6 Hz), -0.9.

^{19}F NMR (CDCl$_3$, 376 MHz) δ -73.8.

HRMS (ESI) Calculated for $[C_{10}H_{13}O_3F_3BrSSi\text{-}Me]^+$ 360.9172, found 360.9162.

The data are in accordance with the literature.[17]

5-Methyl-2-(trimethylsilyl)phenyl trifluoromethanesulfonate (2aj)

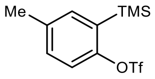

Prepared according to a literature procedure.[13] Isolated as a colourless oil.

^{1}H NMR ($CDCl_3$, 400 MHz) δ 7.31 – 7.29 (m, 1H), 7.23 – 7.20 (m, 2H), 2.37 (s, 3H), 0.36 (s, 9H).

^{13}C NMR ($CDCl_3$, 101 MHz) δ 153.2, 137.4, 136.8, 132.4, 131.8, 119.4 (q, J = 1.5 Hz), 118.7 (q, J = 230.4 Hz), 21.0, -0.7.

^{19}F NMR ($CDCl_3$, 376 MHz) δ -74.0.

HRMS (APCI) Calculated for $[C_{11}H_{15}O_3F_3SSi\text{-}Me]^+$ 297.0223, found 297.0224.

The data are in accordance with the literature.[16]

4-(Trimethylsilyl)-[1,1'-biphenyl]-3-yl trifluoromethanesulfonate (2ak)

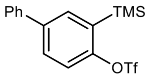

Prepared according to a literature procedure.[10] Isolated as a colourless solid.

^{1}H NMR ($CDCl_3$, 400 MHz) δ 7.69 (d, J = 2.4 Hz, 1H), 7.62 (dd, J = 8.6, 2.5 Hz, 1H), 7.56 – 7.52 (m, 2H), 7.50 – 7.44 (m, 2H), 7.42 – 7.36 (m, 2H), 0.41 (s, 9H).

^{13}C NMR ($CDCl_3$, 101 MHz) δ 154.6, 140.7, 139.9, 135.1, 133.1, 130.1, 129.1, 128.0, 127.4, 119.9 (q, J = 1.5 Hz), 118.7 (q, J = 320.2 Hz), -0.6.

^{19}F NMR ($CDCl_3$, 376 MHz) δ -73.9.

HRMS (APCI) Calculated for $[C_{16}H_{17}O_3F_3SSi]^+$ 374.0614, found 374.0598.

The data are in accordance with the literature.[15]

N,N-Dimethyl-4'-nitro-[1,1'-biphenyl]-2-amine (3a)

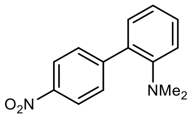

Prepared according to general procedure E (0.2 mmol scale). Purified by column chromatography (0-5% EtOAc in hexane). The pure product **3a** was afforded as a yellow oil (18 mg, 36% yield).

^{1}H NMR (400 MHz, $CDCl_3$) δ 8.29 – 8.22 (m, 2H), 7.81 – 7.73 (m, 2H), 7.38 – 7.31 (m, 1H), 7.23 (dd, J = 7.6, 1.7 Hz, 1H), 7.11 – 6.99 (m, 2H), 2.55 (s, 6H).

^{13}C NMR (101 MHz, $CDCl_3$) δ 151.5, 149.1, 146.6, 131.8, 131.5, 129.7, 129.5, 123.9, 122.0, 118.3, 43.7.

HRMS (ESI) Calculated for $[C_{14}H_{14}O_2N_2+H]^+$ 243.1128, found 243.1126.

2'-Methoxy-N,N-dimethyl-4'-nitro-[1,1'-biphenyl]-2-amine (3b)

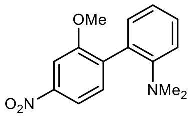

A 20 mL microwave vial was charged with **1b** (585 mg, 3.0 mmol), KF (174 mg, 3.0 mmol) and 18-crown-6 (792 mg, 3.0 mmol), evacuated and backfilled with nitrogen (×3). Anhydrous THF (10 mL) was added *via* syringe, followed by the kobayashi precursor **2a** (243 µL, 1.0 mmol) *via* microsyringe. The reaction was stirred at 50 °C for 16 hours. The reaction mixture was cooled to room temperature, quenched with water and the phases were separated. The aqueous layer was extracted with EtOAc (2 x 10 mL) and the combined organic phases were dried with $MgSO_4$, filtered, and concentrated *in vacuo*. The crude product was then purified by flash column chromatography (column conditions: 0-5% EtOAc in hexane), affording **3b** as a red solid (192 mg, 71% yield).

^{1}H NMR (400 MHz, CDCl$_3$) δ 7.88 (dd, J = 8.4, 2.2 Hz, 1H), 7.84 (d, J = 2.2 Hz, 1H), 7.49 (d, J = 8.3 Hz, 1H), 7.37 – 7.29 (m, 1H), 7.17 (dd, J = 7.6, 1.7 Hz, 1H), 7.10 (dd, J = 8.2, 1.2 Hz, 1H), 7.02 (t, J = 7.5 Hz, 1H), 3.89 (s, 3H), 2.52 (s, 6H).

^{13}C NMR (101 MHz, CDCl$_3$) δ 157.0, 152.0, 147.9, 138.1, 131.74, 131.66, 129.3, 129.1, 121.3, 118.3, 116.0, 106.4, 56.2, 43.6.

HRMS (ESI) Calculated for $[C_{15}H_{16}O_3N_2+H]^+$ 273.1234, found 273.1228.

mp: 71-73 °C.

2'-Ethoxy-N,N-dimethyl-4'-nitro-[1,1'-biphenyl]-2-amine (3c)

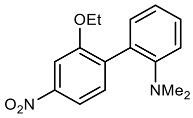

Prepared according to General Procedure E (0.2 mmol scale). Purified by column chromatography (0-5% EtOAc in hexane). The pure product **3c** was afforded as an orange oil (47 mg, 79% yield).

^{1}**H NMR (CDCl$_3$, 500 MHz)** δ 7.86 (dd, J = 8.4, 2.2 Hz, 1H), 7.81 (d, J = 2.2 Hz, 1H), 7.48 (d, J = 8.3 Hz, 1H), 7.32 (td, J = 8.2, 1.7 Hz, 1H), 7.17 (dd, J = 7.6, 1.7 Hz, 1H), 7.08 (d, J = 8.2 Hz, 1H), 7.00 (t, J = 7.4 Hz, 1H), 4.14 (q, J = 7.0 Hz, 2H), 2.52 (s, 6H), 1.34 (t, J = 7.0 Hz, 3H).

^{13}**C NMR (CDCl$_3$, 126 MHz)** δ 156.2, 152.0, 147.8, 138.4, 131.77, 131.75, 129.3, 129.1, 121.1, 118.0, 115.8, 107.2, 64.5, 43.6, 14.6.

HRMS (ESI) Calculated for $[C_{16}H_{18}O_3N_2+H]^+$ 287.1396, found 287.1393.

2'-(Methoxymethoxy)-N,N-dimethyl-4'-nitro-[1,1'-biphenyl]-2-amine (3d)

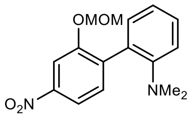

Prepared according to general procedure E (0.1 mmol scale). Purified by column chromatography (0-10% EtOAc in hexane). The pure product **3d** was afforded as a yellow oil (23 mg, 76% yield).

^{1}**H NMR (400 MHz, CDCl$_3$)** δ 8.11 (d, J = 2.2 Hz, 1H), 7.93 (dd, J = 8.4, 2.2 Hz, 1H), 7.50 (d, J = 8.4 Hz, 1H), 7.38 – 7.29 (m, 1H), 7.17 (dd, J = 7.6, 1.7 Hz, 1H), 7.09 (dd, J = 8.2, 1.2 Hz, 1H), 7.02 (td, J = 7.4, 1.2 Hz, 1H), 5.20 (s, 2H), 3.42 (s, 3H), 2.53 (s, 6H).

^{13}**C NMR (101 MHz, CDCl$_3$)** δ 154.7, 152.0, 147.8, 138.9, 131.9, 131.7, 129.4, 129.3, 121.3, 118.2, 117.0, 110.6, 95.1, 56.4, 43.7.

HRMS (ESI) Calculated for $[C_{16}H_{18}O_4N_2+H]^+$ 303.1339, found 303.1354.

4-((2'-(Dimethylamino)-4-nitro-[1,1'-biphenyl]-2-yl)oxy)butyl acetate (3e)

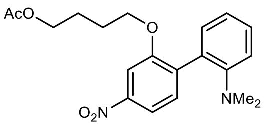

Prepared according to general procedure E (0.2 mmol scale). Purified by column chromatography (0-10% EtOAc in hexane). The pure product **3e** was isolated as an orange oil (32 mg, 43% yield).

^{1}**H NMR (400 MHz, CDCl$_3$)** δ 7.87 (dt, J = 8.3, 1.6 Hz, 1H), 7.81 (s, 1H), 7.50 (d, J = 8.3 Hz, 1H), 7.35 – 7.28 (m, 1H), 7.14 (d, J = 7.5 Hz, 1H), 7.06 (d, J = 8.3 Hz, 1H), 6.97 (t, J = 7.5 Hz, 1H), 4.07 (t, J = 6.1 Hz, 2H), 4.01 (t, J = 6.4 Hz, 2H), 2.51 (d, J = 1.0 Hz, 6H), 2.02 (s, 3H), 1.82 – 1.72 (m, 2H), 1.70 – 1.61 (m, 2H).

^{13}C NMR (101 MHz, CDCl$_3$) δ 171.2, 156.2, 151.9, 147.7, 138.4, 131.8, 131.7, 129.1, 129.0, 121.0, 117.9, 116.0, 107.2, 68.3, 64.0, 43.6, 25.7, 25.3, 21.1.

HRMS (APCI) Calculated for $[C_{20}H_{24}O_5N_2+H]^+$ 373.1758, found 373.1753.

2'-(2-(1,3-Dioxolan-2-yl)ethoxy)-N,N-dimethyl-4'-nitro-[1,1'-biphenyl]-2-amine (3f)

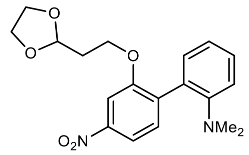

Prepared according to general procedure E (0.2 mmol scale). Purified by column chromatography (0-15% EtOAc in hexane). The pure product **3f** was afforded as a red oil (32 mg, 45% yield).

^{1}H NMR (400 MHz, CDCl$_3$) δ 7.91 – 7.82 (m, 2H), 7.49 (d, J = 8.1 Hz, 1H), 7.33 – 7.27 (m, 1H), 7.14 (dd, J = 7.6, 1.7 Hz, 1H), 7.06 (d, J = 8.1 Hz, 1H), 6.98 (t, J = 7.4 Hz, 1H), 4.90 (t, J = 4.8 Hz, 1H), 4.21 (t, J = 6.6 Hz, 2H), 4.01 – 3.91 (m, 2H), 3.88 – 3.80 (m, 2H), 2.51 (s, 6H), 2.06 (q, J = 6.2 Hz, 2H).

^{13}C NMR (101 MHz, CDCl$_3$) δ 156.2, 152.0, 147.8, 138.4, 131.8 (2C), 129.08, 129.05, 121.0, 117.9, 116.0, 107.3, 101.8, 65.0, 64.7, 43.6, 33.6.

HRMS (APCI) Calculated for $[C_{19}H_{22}O_5N_2+H]^+$ 359.1601, found 359.1594.

2'-(Allyloxy)-N,N-dimethyl-4'-nitro-[1,1'-biphenyl]-2-amine (3g)

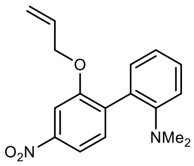

Prepared according to General Procedure E (0.2 mmol scale). Purified by column chromatography (0-5% EtOAc in hexane), followed by preparative thin layer chromatography (10% EtOAc in hexane). The pure product **3g** was afforded as an orange oil (17 mg, 28% yield).

^{1}H NMR (CDCl$_3$, 500 MHz) δ 7.88 (dd, J = 8.3, 2.2 Hz, 1H), 7.81 (d, J = 2.2 Hz, 1H), 7.50 (d, J = 8.3 Hz, 1H), 7.33 (td, J = 8.1, 1.6 Hz, 1H), 7.18 (dd, J = 7.6, 1.8 Hz, 1H), 7.09 (d, J = 8.2 Hz, 1H), 7.02 (t, J = 7.5 Hz, 1H), 5.94 (ddt, J = 17.3, 10.3, 4.9 Hz, 1H), 5.29 (dq, J = 17.3, 1.7 Hz, 1H), 5.23 (dq, J = 10.6, 1.6 Hz, 1H), 4.64 (dt, J = 4.9, 1.7 Hz, 2H), 2.52 (s, 6H).

^{13}C NMR (CDCl$_3$, 126 MHz) δ 155.8, 152.1, 147.7, 138.5, 132.4, 131.8, 131.7, 129.3, 129.2, 121.2, 118.1, 117.7, 116.1, 107.7, 69.4, 43.7.

HRMS (APCI) Calculated for $[C_{17}H_{18}O_3N_2]^+$ 298.1312, found 298.1313.

2'-Fluoro-N,N-dimethyl-4'-nitro-[1,1'-biphenyl]-2-amine (3h)

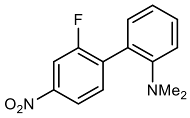

Prepared according to general procedure E (0.2 mmol scale). Purified by column chromatography (0-20% EtOAc in hexane). The pure product **3h** was afforded as a yellow oil (22 mg, 42% yield).

^{1}H NMR (400 MHz, CDCl$_3$) δ 8.11 – 7.99 (m, 2H), 7.68 (dd, J = 8.5, 7.1 Hz, 1H), 7.42 – 7.34 (m, 1H), 7.21 (dt, J = 7.6, 1.6 Hz, 1H), 7.14 (dd, J = 8.3, 1.2 Hz, 1H), 7.11 – 7.02 (m, 1H), 2.55 (s, 6H).

^{13}C NMR (101 MHz, CDCl$_3$) δ 159.2 (d, J = 251.2 Hz), 152.3, 147.5 (d, J = 8.7 Hz), 136.7 (d, J = 15.9 Hz), 132.4 (d, J = 3.9 Hz), 131.6 (d, J = 2.1 Hz), 130.2, 126.7 (d, J = 1.7 Hz), 121.9, 119.3 (d, J = 3.8 Hz), 118.7, 112.0 (d, J = 28.2 Hz), 43.8.

^{19}F NMR (376 MHz, CDCl$_3$) δ -110.4 (t, J = 8.3 Hz).

HRMS (APCI) calculated for $[C_{14}H_{13}O_2N_2F+H]^+$ 261.1034, found 261.1022.

N,N-Diethyl-2'-methoxy-4'-nitro-[1,1'-biphenyl]-2-amine (3i)

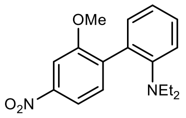

Prepared according to general procedure E (0.2 mmol scale). Purified by column chromatography (0-10% EtOAc in petroleum ether). The pure product **3i** was isolated as a yellow oil (27 mg, 46% yield).

^{1}H NMR (400 MHz, CDCl$_3$) δ 7.88 (dd, J = 8.3, 2.2 Hz, 1H), 7.81 (d, J = 2.1 Hz, 1H), 7.43 (d, J = 8.3 Hz, 1H), 7.37 – 7.27 (m, 1H), 7.18 – 7.11 (m, 2H), 7.06 (td, J = 7.4, 1.2 Hz, 1H), 3.86 (s, 3H), 2.82 (q, J = 7.1 Hz, 4H), 0.84 (t, J = 7.0 Hz, 6H).

^{13}C NMR (101 MHz, CDCl$_3$) δ 157.2, 150.0, 147.9, 138.2, 132.5, 132.1, 131.4, 128.8, 122.1, 121.5, 115.7, 106.0, 56.0, 46.8, 12.5.

HRMS (ESI) Calculated for $[C_{17}H_{20}O_3N_2+H]^+$ 301.1547, found 301.1548.

N-Ethyl-2'-methoxy-N-methyl-4'-nitro-[1,1'-biphenyl]-2-amine (3j)

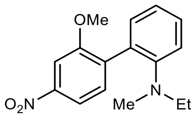

Prepared according to general procedure E (0.2 mmol scale, 1.0 eq. of **1j**). Purified by column chromatography (0-10% EtOAc in hexane). The pure product **3j** was afforded as a red oil (34 mg, 59% yield).

^{1}H NMR (400 MHz, $CDCl_3$) δ 7.88 (dd, J = 8.3, 2.2 Hz, 1H), 7.83 (d, J = 2.2 Hz, 1H), 7.45 (d, J = 8.3 Hz, 1H), 7.33 (ddd, J = 8.0, 7.3, 1.8 Hz, 1H), 7.14 (ddd, J = 14.7, 7.9, 1.4 Hz, 2H), 7.04 (td, J = 7.4, 1.2 Hz, 1H), 3.88 (s, 3H), 2.74 (q, J = 7.1 Hz, 2H), 2.54 (s, 3H), 0.79 (t, J = 7.1 Hz, 3H).

^{13}C NMR (101 MHz, $CDCl_3$) δ 157.2, 151.8, 147.9, 138.2, 131.9, 131.4, 130.7, 129.1, 121.7, 119.7, 115.9, 106.2, 56.2, 50.5, 40.2, 12.5.

HRMS (ESI) Calculated for $[C_{16}H_{18}O_3N_2+H]^+$ 287.1390, found 287.1385.

N-Benzyl-2'-methoxy-N-methyl-4'-nitro-[1,1'-biphenyl]-2-amine (3k)

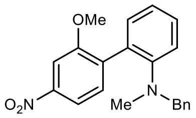

Prepared according to General Procedure E (0.2 mmol scale). Purified by column chromatography (0-10% EtOAc in hexane). The pure product **3k** was afforded as an orange gum (17 mg, 24% yield).

^{1}H NMR ($CDCl_3$, 400 MHz) δ 7.89 (dd, J = 8.3, 2.2 Hz, 1H), 7.79 (d, J = 2.2 Hz, 1H), 7.46 (d, J = 8.3 Hz, 1H), 7.36 (ddd, J = 8.7, 7.3, 1.8 Hz, 1H), 7.22 – 7.15 (m, 5H), 7.10 (td, J = 7.4, 1.2 Hz, 1H), 6.92 (dd, J = 7.3, 2.2 Hz, 2H), 3.86 (s, 2H), 3.85 (s, 3H), 2.47 (s, 3H).

^{13}C NMR ($CDCl_3$, 101 MHz) δ 157.3, 152.0, 148.1, 138.5, 137.8, 132.1, 131.5, 130.9, 129.3, 128.3, 128.1, 127.1, 122.4, 120.3, 115.9, 106.1, 61.0, 56.2, 40.4.

HRMS (ESI) Calculated for $[C_{21}H_{20}O_3N_2+H]^+$ 349.1547, found 349.1566.

N-(2'-Methoxy-4'-nitro-[1,1'-biphenyl]-2-yl)-N-methyltetrahydro-2H-pyran-4-amine (3l)

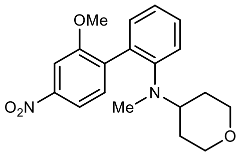

Prepared according to General Procedure E (0.2 mmol scale). Purified by column chromatography (0-10% EtOAc in hexane). The pure product **3l** was afforded as an orange oil (15 mg, 21% yield).

^{1}H NMR ($CDCl_3$, 500 MHz) δ 7.88 (dd, J = 8.3, 2.2 Hz, 1H), 7.83 (d, J = 2.2 Hz, 1H), 7.40 (d, J = 8.3 Hz, 1H), 7.34 (ddd, J = 9.0, 7.3, 1.8 Hz, 1H), 7.17 (dd, J = 7.4, 1.7 Hz, 2H), 7.09 (td, J = 7.4, 1.2 Hz, 1H), 3.89 (s, 3H), 3.82 (dd, J = 11.5, 4.4 Hz, 2H), 3.01 (td, J = 11.9, 2.0 Hz, 2H), 2.82 – 2.71 (m, 1H), 2.60 (s, 3H), 1.53 (qd, J = 12.1, 4.5 Hz, 2H), 1.16 (dd, J = 12.8, 2.3 Hz, 2H).

^{13}C NMR ($CDCl_3$, 126 MHz) δ 157.2, 151.2, 147.9, 138.0, 132.04, 132.01, 131.3, 129.0, 122.5, 121.6, 115.8, 105.9, 67.8, 60.3, 56.1, 34.4, 29.6.

HRMS (ESI) Calculated for $[C_{19}H_{22}O_4N_2+H]^+$ 343.1652, found 343.1661.

1-(2'-Methoxy-4'-nitro-[1,1'-biphenyl]-2-yl)piperidine (3m)

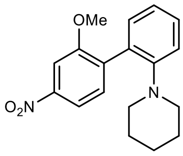

Prepared according to general procedure E (0.2 mmol scale). Purified by column chromatography (0-5% EtOAc in hexane). The pure product **3m** was afforded as an orange oil (28 mg, 45% yield).

^{1}H NMR (400 MHz, $CDCl_3$) δ 7.89 (dd, J = 8.3, 2.2 Hz, 1H), 7.83 (d, J = 2.2 Hz, 1H), 7.54 (d, J = 8.4 Hz, 1H), 7.34 (ddd, J = 8.1, 7.3, 1.7 Hz, 1H), 7.20 (dd, J = 7.5, 1.7 Hz, 1H), 7.13 – 7.02 (m, 2H), 3.89 (s, 3H), 2.76 – 2.69 (m, 4H), 1.43 – 1.26 (m, 6H).

^{13}C NMR (101 MHz, $CDCl_3$) δ 157.1, 152.5, 147.8, 137.6, 131.9, 131.2, 130.9, 129.4, 122.3, 119.5, 115.7, 106.1, 56.1, 53.1, 26.3, 24.2.

HRMS (APCI) Calculated for $[C_{18}H_{20}O_3N_2+H]^+$ 313.1547, found 313.1540.

4-(2'-Methoxy-4'-nitro-[1,1'-biphenyl]-2-yl)morpholine (3n)

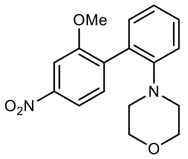

Prepared according to General Procedure E (0.2 mmol scale). Purified by column chromatography (5-25% EtOAc in hexane). The pure product **3n** was afforded as a yellow-orange solid (29 mg, 45% yield).

^{1}H NMR ($CDCl_3$, 400 MHz) δ 7.90 (dd, J = 8.3, 2.2 Hz, 1H), 7.84 (d, J = 2.2 Hz, 1H), 7.53 (d, J = 8.3 Hz, 1H), 7.38 (td, J = 7.8, 1.7 Hz, 1H), 7.22 (dd, J = 7.9, 1.8 Hz, 1H), 7.13 (t, J = 6.9 Hz, 2H), 3.89 (s, 3H), 3.54 – 3.45 (m, 4H), 2.83 – 2.75 (m, 4H).

^{13}C NMR ($CDCl_3$, 101 MHz) δ 157.1, 150.9, 148.1, 137.1, 131.8, 131.5, 131.0, 129.6, 123.2, 119.3, 115.8, 106.1, 67.2, 56.1, 52.0.

HRMS (APCI) Calculated for $[C_{17}H_{18}O_4N_2+H]^+$ 315.1339, found 315.1338.

mp: 143-144 °C.

1-(2'-Methoxy-4'-nitro-[1,1'-biphenyl]-2-yl)azepane (3o)

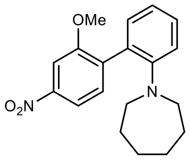

Prepared according to General Procedure E (0.2 mmol scale). Purified by column chromatography (0-5% EtOAc in hexane). The pure product **3o** was afforded as an orange gum (11 mg, 17% yield).

^{1}H NMR ($CDCl_3$, 400 MHz) δ 7.89 (dd, J = 8.3, 2.2 Hz, 1H), 7.81 (d, J = 2.2 Hz, 1H), 7.41 (d, J = 8.3 Hz, 1H), 7.30 (ddd, J = 8.2, 7.2, 1.8 Hz, 1H), 7.17 – 7.08 (m, 2H), 6.99 (td, J = 7.4, 1.2 Hz, 1H), 3.87 (s, 3H), 3.01 – 2.95 (m, 4H), 1.49 – 1.41 (m, 4H), 1.40 – 1.32 (m, 4H).

^{13}C NMR ($CDCl_3$, 101 MHz) δ 157.2, 153.7, 147.8, 138.8, 132.0, 131.4, 129.8, 129.1, 121.0, 120.1, 115.8, 105.9, 56.1, 55.2, 29.2, 27.3.

HRMS (ESI) Calculated for $[C_{19}H_{22}O_3N_2+H]^+$ 327.1709, found 327.1711.

4-(2'-Methoxy-4'-nitro-[1,1'-biphenyl]-2-yl)-1,4-oxazepane (3p)

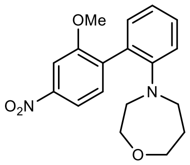

Prepared according to general procedure E (0.2 mmol scale). Purified by column chromatography (0-20% EtOAc in hexane). The pure product **3p** was afforded as a red solid (13 mg, 20% yield).

^{1}H NMR (400 MHz, CDCl$_3$) δ 7.91 (dd, J = 8.3, 2.2 Hz, 1H), 7.83 (d, J = 2.2 Hz, 1H), 7.43 (d, J = 8.3 Hz, 1H), 7.34 (ddd, J = 8.7, 7.3, 1.8 Hz, 1H), 7.16 (ddd, J = 7.6, 3.7, 1.5 Hz, 2H), 7.06 (td, J = 7.4, 1.2 Hz, 1H), 3.88 (s, 3H), 3.63 (t, J = 6.0 Hz, 2H), 3.48 – 3.41 (m, 2H), 3.11 – 3.03 (m, 4H), 1.53 (p, J = 6.2 Hz, 2H).

^{13}C NMR (101 MHz, CDCl$_3$) δ 157.3, 153.0, 148.0, 138.1, 132.0, 131.3, 130.7, 129.3, 122.3, 120.5, 115.8, 105.9, 71.0, 69.6, 57.6, 56.1, 54.0, 30.7.

HRMS (ESI) Calculated for $[C_{18}H_{20}O_4N_2+Na]^+$ 351.1315, found 351.1314.

mp: 108-110 °C.

1-(2'-Methoxy-4'-nitro-[1,1'-biphenyl]-2-yl)-3-methylpiperidine (3q)

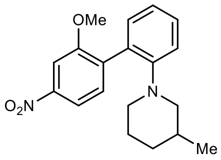

Prepared according to General Procedure E (0.2 mmol scale). Purified by column chromatography (0-5% EtOAc in hexane). The pure product **3q** was afforded as an orange oil (40 mg, 62% yield).

^{1}H NMR (CDCl$_3$, 400 MHz) δ 7.88 (dd, J = 8.3, 2.2 Hz, 1H), 7.83 (d, J = 2.2 Hz, 1H), 7.51 (d, J = 8.4 Hz, 1H), 7.34 (ddd, J = 8.1, 7.3, 1.7 Hz, 1H), 7.19 (dd, J = 7.5, 1.7 Hz, 1H), 7.10 (dd, J = 8.2, 1.2 Hz, 1H), 7.07 (td, J = 7.4, 1.2 Hz, 1H), 3.88 (s, 3H), 2.93 – 2.82 (m, 2H), 2.48 (td, J = 11.3, 2.7 Hz, 1H), 2.21 (dd, J = 11.4, 9.7 Hz, 1H), 1.67 – 1.57 (m, 1H), 1.50 – 1.41 (m, 1H), 1.41 – 1.28 (m, 1H), 1.28 – 1.12 (m, 1H), 0.95 – 0.79 (m, 1H), 0.71 (d, J = 6.7 Hz, 3H).

^{13}C NMR (CDCl$_3$, 101 MHz) δ 157.1, 152.3, 147.8, 137.7, 132.0, 131.2, 131.0, 129.4, 122.3, 119.6, 115.7, 106.0, 60.2, 56.1, 52.7, 32.7, 31.4, 25.6, 19.4.

HRMS (ESI) Calculated for $[C_{19}H_{22}O_3N_2+Na]^+$ 349.1523, found 349.1525.

1-(2'-Methoxy-4'-nitro-[1,1'-biphenyl]-2-yl)-4-phenylpiperidine (3r)

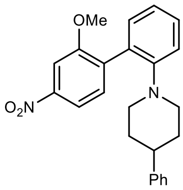

Prepared according to General Procedure E (0.2 mmol scale). Purified by column chromatography (0-5% EtOAc in hexane). The pure product **3r** was afforded as an orange solid (40 mg, 51% yield).

^{1}H NMR (CDCl$_3$, 400 MHz) δ 7.93 (dd, J = 8.3, 2.2 Hz, 1H), 7.86 (d, J = 2.2 Hz, 1H), 7.58 (d, J = 8.3 Hz, 1H), 7.38 (td, J = 8.1, 1.7 Hz, 1H), 7.33 – 7.27 (m, 2H), 7.25 – 7.08 (m, 6H), 3.91 (s, 3H), 3.11 (d, J = 12.1 Hz, 2H), 2.73 (td, J = 11.9, 2.3 Hz, 2H), 2.50 (tt, J = 12.2, 3.7 Hz, 1H), 1.73 (d, J = 11.8 Hz, 2H), 1.43 (qd, J = 12.3, 3.9 Hz, 2H).

^{13}C NMR (CDCl$_3$, 101 MHz) δ 157.1, 152.0, 148.0, 146.1, 137.5, 131.9, 131.4, 131.0, 129.5, 128.6, 126.8, 126.4, 122.5, 119.6, 115.8, 106.1, 56.2, 52.8, 42.2, 33.8.

HRMS (ESI) Calculated for $[C_{24}H_{24}O_3N_2+H]^+$ 411.1679, found 411.1684.

mp: 138-140 °C.

4-Methoxy-1-(2'-methoxy-4'-nitro-[1,1'-biphenyl]-2-yl)piperidine (3s)

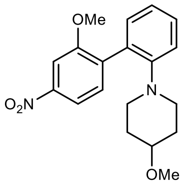

Prepared according to General Procedure E (0.2 mmol scale). Purified by column chromatography (0-5% EtOAc in hexane). The pure product **3s** was afforded as an orange solid (42 mg, 62% yield).

^{1}H NMR (CDCl$_3$, 500 MHz) δ 7.89 (dd, J = 8.3, 2.2 Hz, 1H), 7.83 (d, J = 2.2 Hz, 1H), 7.53 (d, J = 8.3 Hz, 1H), 7.34 (td, J = 7.6, 1.7 Hz, 1H), 7.20 (dd, J = 7.5, 1.7 Hz, 1H), 7.13 – 7.06 (m, 2H), 3.88 (s, 3H), 3.29 (s, 3H), 3.17 (tt, J = 8.4, 3.9 Hz, 1H), 3.02 – 2.91 (m, 2H), 2.61 (ddd, J = 12.2, 9.6, 2.9 Hz, 2H), 1.77 – 1.67 (m, 2H), 1.35 – 1.22 (m, 2H).

^{13}C NMR (CDCl$_3$, 126 MHz) δ 157.0, 151.6, 147.9, 137.3, 131.8, 131.2, 131.0, 129.4, 122.6, 119.5, 115.8, 106.0, 76.2, 56.1, 55.6, 49.7, 31.4.

HRMS (ESI) Calculated for $[C_{19}H_{22}O_4N_2+H]^+$ 343.1652, found 343.1655.

mp: decomp.

4-Bromo-1-(2'-methoxy-4'-nitro-[1,1'-biphenyl]-2-yl)piperidine (3t)

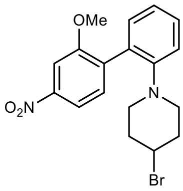

Prepared according to General Procedure E (0.2 mmol scale). Purified by column chromatography (0-5% EtOAc in hexane), followed by preparative thin layer chromatography (10% EtOAc in hexane). The pure product **3t** was afforded as a dark orange solid (33 mg, 43% yield).

^{1}H NMR (CDCl$_3$, 400 MHz) δ 7.91 (dd, *J* = 8.3, 2.2 Hz, 1H), 7.84 (d, *J* = 2.2 Hz, 1H), 7.50 (d, *J* = 8.3 Hz, 1H), 7.36 (ddd, *J* = 8.8, 7.3, 1.8 Hz, 1H), 7.20 (dd, *J* = 7.5, 1.9 Hz, 1H), 7.16 – 7.07 (m, 2H), 4.17 (tt, *J* = 8.7, 4.0 Hz, 1H), 3.89 (s, 3H), 3.07 – 2.98 (m, 2H), 2.70 (ddd, *J* = 11.9, 8.3, 3.2 Hz, 2H), 2.00 – 1.88 (m, 2H), 1.84 – 1.69 (m, 2H).

^{13}C NMR (CDCl$_3$, 101 MHz) δ 157.1, 151.2, 148.0, 137.1, 131.7, 131.4, 131.1, 129.5, 123.1, 119.7, 115.8, 106.1, 56.1, 50.7, 49.7, 36.6.

HRMS (APCI) Calculated for $[C_{18}H_{19}O_3N_2Br]^+$ 390.0574, found 390.0581.

mp: decomp.

1-(2'-Methoxy-4'-nitro-[1,1'-biphenyl]-2-yl)-4-methylpiperidine (3u)

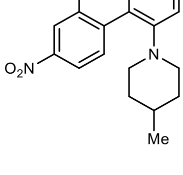

Prepared according to General Procedure E (0.2 mmol scale). Purified by column chromatography (0-5% EtOAc in hexane), followed by preparative thin layer chromatography

(10% EtOAc in hexane). The pure product **3u** was afforded as an orange-brown solid (32 mg, 49% yield).

^{1}H NMR ($CDCl_3$, 400 MHz) *δ* 7.89 (dd, *J* = 8.3, 2.2 Hz, 1H), 7.83 (d, *J* = 2.2 Hz, 1H), 7.54 (d, *J* = 8.3 Hz, 1H), 7.34 (td, *J* = 7.9, 1.7 Hz, 1H), 7.19 (dd, *J* = 7.6, 1.7 Hz, 1H), 7.11 (d, *J* = 8.1 Hz, 1H), 7.06 (t, *J* = 7.5 Hz, 1H), 3.88 (s, 3H), 2.94 (dt, *J* = 11.9, 3.8 Hz, 2H), 2.55 (td, *J* = 11.8, 2.4 Hz, 2H), 1.47 (d, *J* = 12.3 Hz, 2H), 1.38 – 1.22 (m, 2H), 0.96 – 0.79 (m, 4H).

^{13}C NMR ($CDCl_3$, 101 MHz) *δ* 157.0, 152.2, 147.8, 137.6, 131.9, 131.3, 130.8, 129.4, 122.2, 119.5, 115.7, 106.1, 56.1, 52.4, 34.7, 30.7, 22.0.

HRMS (ESI) Calculated for $[C_{19}H_{22}O_3N_2+H]^+$ 327.1703, found 327.1696.

mp: 83-85 °C.

1-(2'-Methoxy-4'-nitro-[1,1'-biphenyl]-2-yl)piperidine-4-carbonitrile (3v)

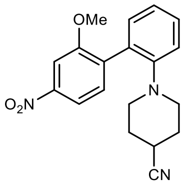

Prepared according to General Procedure E (0.2 mmol scale). Purified by column chromatography (0-25% EtOAc in hexane). The pure product **3v** was afforded as a yellow-orange solid (33 mg, 48% yield).

^{1}H NMR ($CDCl_3$, 400 MHz) *δ* 7.90 (dd, *J* = 8.3, 2.2 Hz, 1H), 7.84 (d, *J* = 2.2 Hz, 1H), 7.47 (d, *J* = 8.3 Hz, 1H), 7.37 (td, *J* = 7.5, 1.8 Hz, 1H), 7.21 (dd, *J* = 7.9, 1.7 Hz, 1H), 7.18 – 7.10 (m, 2H), 3.88 (s, 3H), 3.08 – 2.88 (m, 2H), 2.73 (ddd, *J* = 11.7, 7.5, 3.3 Hz, 2H), 2.63 (tt, *J* = 7.9, 4.5 Hz, 1H), 1.75 – 1.54 (m, 4H).

^{13}C NMR ($CDCl_3$, 126 MHz) *δ* 157.1, 151.1, 148.1, 136.9, 131.7, 131.5, 131.4, 129.6, 123.5, 121.6, 119.9, 115.8, 106.0, 56.1, 50.2, 29.1, 26.0.

HRMS (APCI) Calculated for $[C_{19}H_{19}O_3N_3+H]^+$ 338.1499, found 338.1503.

mp: 136-138 °C.

Methyl 1-(2'-methoxy-4'-nitro-[1,1'-biphenyl]-2-yl)piperidine-4-carboxylate (3w)

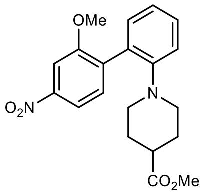

Prepared according to general procedure E (0.2 mmol scale). Purified by column chromatography (0-10% EtOAc in hexane). The pure product **3w** was afforded as a yellow solid (30 mg, 41% yield).

^{1}H NMR (400 MHz, $CDCl_3$) δ 7.89 (dd, J = 8.3, 2.2 Hz, 1H), 7.83 (d, J = 2.2 Hz, 1H), 7.50 (d, J = 8.3 Hz, 1H), 7.38 – 7.31 (m, 1H), 7.20 (dd, J = 8.0, 1.7 Hz, 1H), 7.13 – 7.07 (m, 2H), 3.88 (s, 3H), 3.65 (s, 3H), 3.03 – 2.97 (m, 2H), 2.59 (td, J = 11.6, 2.5 Hz, 2H), 2.31 – 2.21 (m, 1H), 1.80 – 1.71 (m, 2H), 1.46 – 1.35 (m, 2H).

^{13}C NMR (101 MHz, $CDCl_3$) δ 175.5, 157.1, 151.7, 148.0, 137.2, 131.8, 131.4, 131.1, 129.5, 122.8, 119.5, 115.8, 106.1, 56.1, 51.8, 51.6, 40.8, 28.7.

HRMS (ESI) Calculated for $[C_{20}H_{22}O_5N_2+Na]^+$ 393.1421, found 393.1414.

mp: 114-116 °C.

8-(2'-Methoxy-4'-nitro-[1,1'-biphenyl]-2-yl)-1,4-dioxa-8-azaspiro[4.5]decane (3x)

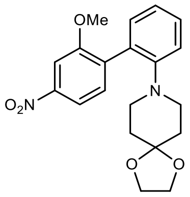

Prepared according to general procedure E (0.2 mmol scale). Purified by column chromatography (0-15% EtOAc in hexane). The pure product **3x** was afforded as an orange solid (33 mg, 45% yield).

^{1}H NMR (400 MHz, $CDCl_3$) δ 7.89 (dd, J = 8.3, 2.2 Hz, 1H), 7.82 (d, J = 2.2 Hz, 1H), 7.52 (d, J = 8.3 Hz, 1H), 7.34 (ddd, J = 8.0, 7.3, 1.7 Hz, 1H), 7.20 (dd, J = 7.6, 1.7 Hz, 1H), 7.14 – 7.07 (m, 2H), 3.91 (s, 4H), 3.88 (s, 3H), 2.90 – 2.85 (m, 4H), 1.54 – 1.46 (m, 4H).

^{13}C NMR (101 MHz, $CDCl_3$) δ 157.1, 151.4, 147.9, 137.3, 131.7, 131.2, 131.1, 129.5, 122.8, 119.8, 115.8, 106.9, 106.1, 64.4, 56.1, 50.0, 35.4.

HRMS (ESI) Calculated for $[C_{20}H_{22}O_5N_2+Na]^+$ 393.1421, found 393.1411.

mp: 130-132 °C.

Cis- and trans-2-(2'-methoxy-4'-nitro-[1,1'-biphenyl]-2-yl)decahydroisoquinoline (3y)

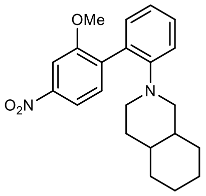

Prepared according to General Procedure E (0.2 mmol scale). Purified by column chromatography (0-2% EtOAc in hexane). The inseparable mixture of **cis-** and **trans-3y** was afforded as an orange oil (59 mg, 80% yield).

^{1}H NMR ($CDCl_3$, 500 MHz) δ 7.89 (dd, J = 8.3, 2.2 Hz, 1H), 7.83 (d, J = 2.2 Hz, 1H), 7.53 (d, J = 8.3 Hz, 1H), 7.33 (td, J = 7.7, 1.8 Hz, 1H), 7.19 (dd, J = 7.5, 1.7 Hz, 1H), 7.10 (d, J = 7.0 Hz, 1H), 7.06 (td, J = 7.4, 1.2 Hz, 1H), 3.88 (s, 3H), 2.93 (d, J = 10.7 Hz, 1H), 2.85 (d, J = 11.0 Hz, 1H), 2.54 (td, J = 11.6, 2.4 Hz, 1H), 2.27 (t, J = 10.3 Hz, 1H), 1.73 – 1.62 (m, 2H), 1.55 (d, J = 10.8 Hz, 1H), 1.42 – 1.28 (m, 2H), 1.25 – 1.13 (m, 2H), 0.98 – 0.79 (m, 5H).

^{13}C NMR ($CDCl_3$, 126 MHz) δ 157.1, 152.1, 147.8, 137.6, 131.9, 131.3, 130.7, 129.4, 122.1, 119.5, 115.7, 106.1, 58.6, 56.1, 53.2, 42.4, 41.8, 33.4, 33.0, 30.4, 26.5, 26.1.

HRMS (ESI) Calculated for $[C_{22}H_{26}O_3N_2+Na]^+$ 389.1836, found 389.1839.

6,7-Dimethoxy-2-(2'-methoxy-4'-nitro-[1,1'-biphenyl]-2-yl)-1,2,3,4-tetrahydroisoquinoline (3z)

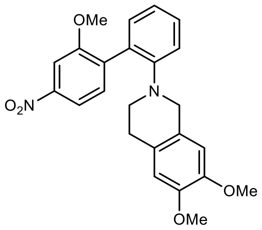

Prepared according to General Procedure E (0.2 mmol scale, 2.0 eq. of **1z**). Purified by column chromatography (0-10% EtOAc in hexane). The pure product **3z** was afforded as an orange gum (38 mg, 45% yield).

^{1}H NMR ($CDCl_3$, 500 MHz) δ 7.86 (dd, J = 8.3, 2.2 Hz, 1H), 7.83 (d, J = 2.2 Hz, 1H), 7.53 (d, J = 8.3 Hz, 1H), 7.38 (td, J = 7.7, 1.7 Hz, 1H), 7.23 (dd, J = 7.5, 1.7 Hz, 1H), 7.20 (dd, J = 8.2, 1.2 Hz, 1H),

7.11 (td, *J* = 7.4, 1.2 Hz, 1H), 6.52 (d, *J* = 3.4 Hz, 2H), 4.07 (s, 2H), 3.84 (s, 6H), 3.83 (s, 3H), 2.96 (t, *J* = 5.7 Hz, 2H), 2.34 (t, *J* = 5.7 Hz, 2H).

^{13}C NMR ($CDCl_3$, 126 MHz) δ 157.1, 150.9, 147.9, 147.6, 147.4, 137.5, 131.9, 131.6, 130.4, 129.4, 126.7, 126.4, 122.4, 119.1, 115.9, 111.6, 109.3, 106.3, 56.2, 56.1, 56.0, 52.4, 51.0, 28.8.

HRMS (ESI) Calculated for $[C_{24}H_{24}O_5N_2+Na]^+$ 443.1577, found 443.1571.

2'-Methoxy-N,N,6-trimethyl-4'-nitro-[1,1'-biphenyl]-2-amine (3aa)

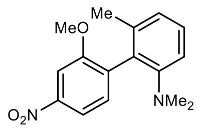

Prepared according to General Procedure E (0.2 mmol scale). Purified by column chromatography (0-1% EtOAc in hexane). The pure product **3aa** was afforded as a yellow-orange solid (32 mg, 56% yield, single regioisomer).

^{1}H NMR ($CDCl_3$, 400 MHz) δ 7.91 (dd, *J* = 8.3, 2.2 Hz, 1H), 7.84 (d, *J* = 2.2 Hz, 1H), 7.29 – 7.22 (m, 2H), 7.00 (d, *J* = 8.1 Hz, 1H), 6.95 (d, *J* = 7.5 Hz, 1H), 3.88 (s, 3H), 2.44 (s, 6H), 1.98 (s, 3H).

^{13}C NMR ($CDCl_3$, 101 MHz) δ 157.5, 152.7, 148.1, 137.7, 137.1, 132.2, 130.8, 128.8, 124.1, 116.5, 116.2, 105.8, 56.0, 44.1, 20.2.

HRMS (ESI) Calculated for $[C_{16}H_{18}O_3N_2+H]^+$ 287.1396, found 287.1394.

mp: 87-81 °C.

4-Bromo-6-isopropyl-2'-methoxy-N,N-dimethyl-4'-nitro-[1,1'-biphenyl]-2-amine (3ab)

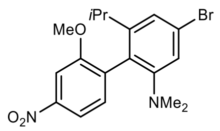

Prepared according to General Procedure E (0.2 mmol scale). Purified by column chromatography (0-2% EtOAc in hexane), followed by preparative thin layer chromatography (10% EtOAc in hexane). The pure product **3ab** was afforded as an orange gum (10 mg, 13% yield, single regioisomer).

^{1}H NMR (CDCl$_3$, 400 MHz) *δ* 7.91 (dd, *J* = 8.2, 2.1 Hz, 1H), 7.82 (d, *J* = 2.2 Hz, 1H), 7.22 (d, *J* = 8.2 Hz, 1H), 7.17 (d, *J* = 1.9 Hz, 1H), 7.09 (d, *J* = 1.9 Hz, 1H), 3.87 (s, 3H), 2.44 (hept, *J* = 6.8 Hz, 1H), 2.41 (s, 6H), 1.13 (d, *J* = 6.8 Hz, 3H), 0.96 (d, *J* = 6.8 Hz, 3H).

^{13}C NMR (CDCl$_3$, 101 MHz) *δ* 157.7, 154.0, 150.3, 148.4, 135.8, 132.4, 128.6, 123.2, 123.0, 120.4, 116.1, 105.7, 56.0, 44.0, 30.6, 24.4, 23.2.

HRMS (ESI) Calculated for $[C_{18}H_{21}O_3N_2Br+H]^+$ 393.0808, found 393.0795.

2',6-Dimethoxy-N,N-dimethyl-4'-nitro-[1,1'-biphenyl]-2-amine (3ac)

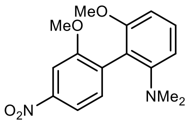

Prepared according to general procedure E (0.2 mmol scale). Purified by column chromatography (0-5% EtOAc in hexane). The pure product **3ac** was afforded as a red oil (28 mg, 46% yield, single regioisomer).

^{1}H NMR (400 MHz, CDCl$_3$) *δ* 7.83 (dd, *J* = 8.3, 2.2 Hz, 1H), 7.78 – 7.73 (m, 1H), 7.32 – 7.17 (m, 2H), 6.71 (d, *J* = 8.2 Hz, 1H), 6.59 (d, *J* = 8.3 Hz, 1H), 3.81 (s, 3H), 3.63 (s, 3H), 2.40 (s, 6H).

^{13}C NMR (101 MHz, CDCl$_3$) *δ* 158.2, 157.7, 153.6, 148.1, 134.6, 132.4, 129.7, 118.9, 116.0, 111.4, 106.0, 105.0, 56.2, 56.0, 43.8.

HRMS (ESI) Calculated for $[C_{16}H_{18}O_4N_2+Na]^+$ 325.1159, found 325.1154.

2''-Methoxy-N,N-dimethyl-4''-nitro-[1,1':2',1''-terphenyl]-3'-amine (3ad)

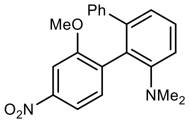

Prepared according to general procedure E (0.2 mmol scale). Purified by column chromatography (0-5% EtoAc in hexane). The pure product **3ad** was afforded as an orange solid (30 mg, 43% yield, single regioisomer).

^{1}H NMR (400 MHz, CDCl$_3$) *δ* 7.80 (dd, *J* = 8.3, 2.2 Hz, 1H), 7.50 (d, *J* = 2.2 Hz, 1H), 7.40 (t, *J* = 7.9 Hz, 1H), 7.36 (d, *J* = 8.3 Hz, 1H), 7.18 (d, *J* = 8.2 Hz, 1H), 7.14 – 7.09 (m, 3H), 7.07 (d, *J* = 7.6 Hz, 1H), 7.00 – 6.95 (m, 2H), 3.46 (s, 3H), 2.48 (s, 6H).

^{13}C NMR (101 MHz, CDCl$_3$) δ 157.2, 152.7, 147.9, 143.2, 141.8, 136.6, 133.0, 129.2, 129.1. 129.0, 127.4, 126.6, 124.3, 118.2, 115.6, 105.9, 55.6, 44.1.

HRMS (ESI) Calculated for $[C_{21}H_{20}O_3N_2]^+$ 349.1547, found 349.1555.

XRD: Recrystallised from chloroform as yellow crystals. Single crystal analysis confirms the structure drawn (CCDC deposition number: 2244918).

mp: 82-84 °C.

2',4,5-Trimethoxy-N,N-dimethyl-4'-nitro-[1,1'-biphenyl]-2-amine (3ae)

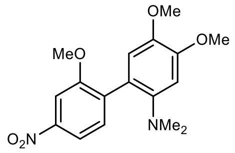

Prepared according to general procedure E (0.2 mmol scale). Purified by column chromatography (0-20% EtOAc in hexane). The pure product **3ae** was afforded as a red oil (25 mg, 38% yield, single regioisomer).

^{1}H NMR (400 MHz, CDCl$_3$) δ 7.88 (dd, J = 8.3, 2.2 Hz, 1H), 7.83 (d, J = 2.2 Hz, 1H), 7.48 (d, J = 8.3 Hz, 1H), 6.72 (d, J = 2.9 Hz, 2H), 3.93 (s, 3H), 3.89 (s, 3H), 3.83 (s, 3H), 2.48 (s, 6H).

^{13}C NMR (101 MHz, CDCl$_3$) δ 157.2, 149.5, 147.7, 146.2, 144.3, 137.6, 132.1, 122.2, 115.9, 114.6, 106.3, 103.5, 56.4, 56.2, 56.1, 44.4.

HRMS (ESI) Calculated for $[C_{17}H_{20}O_5N_2+Na]^+$ 355.1264, found 355.1278.

5-Fluoro-2'-methoxy-N,N-dimethyl-4'-nitro-[1,1'-biphenyl]-2-amine (major) and 4-fluoro-2'-methoxy-N,N-dimethyl-4'-nitro-[1,1'-biphenyl]-2-amine (minor) (3af)

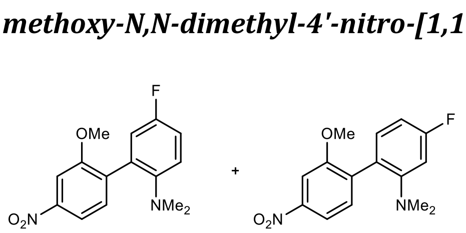

Prepared according to general procedure E (0.2 mmol scale). Purified by column chromatography (0-5% EtOAc in hexane). The inseparable mixture of regioisomers **3af** was afforded as a yellow oil (25 mg, 43% yield, r.r. 3.5:1).

^{1}H NMR (400 MHz, CDCl$_3$) δ 7.91 – 7.83 (m, 4H), 7.49 – 7.43 (m, 2H), 7.14 – 6.98 (m, 3H), 6.91 (dd, J = 9.1, 3.0 Hz, 1H), 6.75 (dd, J = 11.5, 2.5 Hz, 1H), 6.68 (td, J = 8.1, 2.6 Hz, 1H), 3.90 (s, 6H), 2.52 (s, 6H), 2.46 (s, 6H).

^{13}C NMR (101 MHz, CDCl$_3$) δ 163.4 (d, J = 247.1 Hz), 158.0 (d, J = 241.3 Hz), 157.0, 156.9, 153.7 (d, J = 8.8 Hz), 148.5 (d, J = 2.4 Hz), 148.2, 147.9, 137.3, 136.4 (d, J = 1.7 Hz), 133.0 (d, J = 9.8 Hz), 131.8, 131.7, 131.6 (d, J = 7.9 Hz), 123.9 (d, J = 3.1 Hz), 120.0 (d, J = 8.3 Hz), 118.1 (d, J = 22.9 Hz), 116.1, 115.9, 115.5 (d, J = 21.6 Hz), 107.4 (d, J = 21.8 Hz), 106.42, 106.39, 105.2 (d, J = 23.5 Hz), 56.3 (2C), 44.2, 43.3.

^{19}F NMR (376 MHz, CDCl$_3$) δ -112.07 – -112.16 (m), -122.31 – -122.39 (m).

HRMS (ESI) Calculated for $[C_{15}H_{15}O_3N_2F+H]^+$ 291.1139, found 291.1147.

5-Chloro-2'-methoxy-N,N-dimethyl-4'-nitro-[1,1'-biphenyl]-2-amine (major) and 4-chloro-2'-methoxy-N,N-dimethyl-4'-nitro-[1,1'-biphenyl]-2-amine (minor) (3ag)

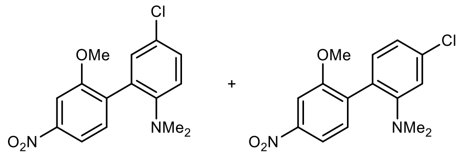

Prepared according to General Procedure E (0.1 mmol scale). Purified by column chromatography (0-5% EtOAc in hexane), followed by preparative thin layer chromatography (10% EtOAc in hexane). The inseparable mixture of regioisomers **3ag** was afforded as an orange solid (15 mg, 47% yield, r.r. 2.4:1).

^{1}H NMR (CDCl$_3$, 400 MHz) δ 7.88 (dd, J = 8.3, 2.2 Hz, 2H), 7.84 (d, J = 2.1 Hz, 2H), 7.45 (dd, J = 8.3, 2.2 Hz, 2H), 7.26 (dd, J = 8.3, 2.2 Hz, 1H), 7.14 (d, J = 2.6 Hz, 1H), 7.08 (d, J = 8.2 Hz, 1H), 7.02 (d, J = 2.1 Hz, 1H), 7.00 (d, J = 8.7 Hz, 1H), 6.96 (dd, J = 8.2, 2.1 Hz, 1H), 3.90 (s, 3H), 3.89 (s, 3H), 2.51 (s, 6H), 2.49 (s, 6H).

^{13}C NMR (CDCl$_3$, 101 MHz) δ 156.8 (2C), 153.0, 150.7, 148.1, 148.0, 136.9, 136.5, 134.8, 132.8, 131.62, 131.57, 131.3, 130.4, 129.0, 126.7, 126.2, 120.9, 119.6, 118.4, 116.1, 116.0, 106.4, 56.29, 56.26, 43.6, 43.3.

HRMS (ESI) Calculated for $[C_{15}H_{15}O_3N_2Cl+H]^+$ 307.0844, found 307.0834.

5-Bromo-2'-methoxy-N,N-dimethyl-4'-nitro-[1,1'-biphenyl]-2-amine (3ah-major)

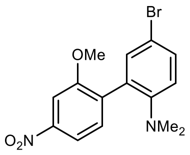

Prepared according to General Procedure E (0.2 mmol scale). Purified by column chromatography (0-5% EtOAc in hexane), followed by preparative thin layer chromatography (10% EtOAc in hexane). The pure product **3ah-major** was afforded as an orange solid (24 mg, 34% yield).

^{1}H NMR ($CDCl_3$, 400 MHz) δ 7.88 (dd, J = 8.4, 2.1 Hz, 1H), 7.84 (d, J = 2.2 Hz, 1H), 7.45 (d, J = 8.3 Hz, 1H), 7.40 (dd, J = 8.7, 2.4 Hz, 1H), 7.27 (d, J = 2.5 Hz, 1H), 6.94 (d, J = 8.7 Hz, 1H), 3.90 (s, 3H), 2.49 (s, 6H).

^{13}C NMR ($CDCl_3$, 101 MHz) δ 156.9, 151.1, 148.2, 136.5, 134.1, 131.9, 131.6, 130.7, 119.9, 116.0, 113.6, 106.4, 56.3, 43.5.

HRMS (ESI) Calculated for $[C_{15}H_{15}O_3N_2Br+H]^+$ 351.0344, found 351.0338.

mp: 112-115 °C.

4-Bromo-2'-methoxy-N,N-dimethyl-4'-nitro-[1,1'-biphenyl]-2-amine (3ah-minor)

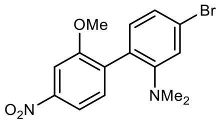

Prepared according to General Procedure E (0.2 mmol scale). Purified by column chromatography (0-5% EtOAc in hexane), followed by preparative thin layer chromatography (10% EtOAc in hexane). The pure product **3ah-minor** was afforded as an orange solid (9 mg, 13% yield).

^{1}H NMR ($CDCl_3$, 400 MHz) δ 7.88 (d, J = 8.3, 2.2 Hz, 1H), 7.83 (d, J = 2.2 Hz, 1H), 7.45 (d, J = 8.4 Hz, 1H), 7.17 (d, J = 1.9 Hz, 1H), 7.12 (dd, J = 8.2, 1.9 Hz, 1H), 7.01 (d, J = 8.2 Hz, 1H), 3.89 (s, 3H), 2.51 (s, 6H).

^{13}C NMR ($CDCl_3$, 101 MHz) δ 156.9, 153.2, 148.1, 137.0, 133.0, 131.5, 127.3, 123.9, 123.0, 121.5, 116.1, 106.4, 56.3, 43.3.

HRMS (ESI) Calculated for $[C_{15}H_{15}O_3N_2Br+H]^+$ 351.0344, found 351.0336.

mp: decomp.

2',5-Dimethoxy-N,N-dimethyl-4'-nitro-[1,1'-biphenyl]-2-amine (major) and 2',4-dimethoxy-N,N-dimethyl-4'-nitro-[1,1'-biphenyl]-2-amine (minor) (3ai)

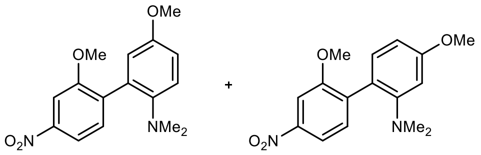

Prepared according to general procedure E (0.2 mmol scale). Purified by column chromatography (0-5% EtOAc in hexane). The inseparable mixture of regioisomers **3ai** was afforded as a red oil (14 mg, 23% yield, r.r 1.8:1).

^{1}H NMR (400 MHz, CDCl$_3$) δ 7.87 (ddd, J = 8.7, 6.5, 2.2 Hz, 2H), 7.83 (t, J = 2.1 Hz, 2H), 7.47 (dd, J = 10.2, 8.3 Hz, 2H), 7.11 (dd, J = 8.6, 1.4 Hz, 2H), 6.90 (dd, J = 8.8, 3.1 Hz, 1H), 6.75 (d, J = 3.1 Hz, 1H), 6.62 (d, J = 2.5 Hz, 1H), 6.56 (dd, J = 8.4, 2.5 Hz, 1H), 3.89 (d, J = 2.1 Hz, 6H), 3.84 (s, 3H), 3.79 (s, 3H), 2.51 (s, 6H), 2.45 (s, 6H).

^{13}C NMR (101 MHz, CDCl$_3$) δ 160.5, 157.2, 157.0, 154.9, 153.4, 148.0, 147.6, 146.0, 138.0, 137.5, 132.6, 132.1, 131.9, 131.8, 121.2, 120.2, 116.9, 116.0, 115.9, 114.3, 106.4, 106.3, 105.5, 104.9, 56.2 (2C), 55.7, 55.4, 44.6, 43.5.

HRMS (ESI) Calculated for $[C_{16}H_{18}O_4N_2+H]^+$ 303.1339, found 303.1350.

2'-Methoxy-N,N,5-trimethyl-4'-nitro-[1,1'-biphenyl]-2-amine (major) and 2'-methoxy-N,N,4-trimethyl-4'-nitro-[1,1'-biphenyl]-2-amine (minor) (3aj)

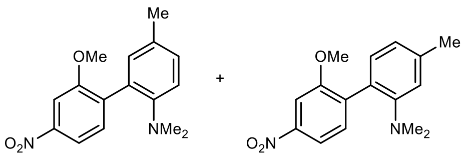

Prepared according to General Procedure E (0.2 mmol scale). Purified by column chromatography (0-5% EtOAc in hexane), followed by preparative thin layer chromatography (10% EtOAc in hexane). The inseparable mixture of regioisomers **3aj** was afforded as an orange solid (32 mg, 56% yield, r.r 1.1:1).

^{1}H NMR (CDCl$_3$, 400 MHz) δ 7.88 (dt, J = 8.4, 2.4 Hz, 2H), 7.83 (d, J = 2.2 Hz, 2H), 7.47 (t, J = 8.4 Hz, 2H), 7.15 (dd, J = 8.3, 2.2 Hz, 1H), 7.07 (d, J = 7.7 Hz, 1H), 7.03 (d, J = 8.2 Hz, 1H), 6.98 (d, J =

2.2 Hz, 1H), 6.91 (s, 1H), 6.85 (d, *J* = 7.7 Hz, 1H), 3.89 (s, 6H), 2.51 (s, 6H), 2.48 (s, 6H), 2.38 (s, 3H), 2.32 (s, 3H).

^{13}C NMR ($CDCl_3$, 126 MHz) *δ* 157.1, 157.0, 151.9, 149.8, 147.8, 147.7, 139.3, 138.14, 138.08, 132.0, 131.81, 131.78, 131.5, 131.1, 129.9, 129.8, 126.2, 122.1, 119.1, 118.6, 116.0, 115.9, 106.34, 106.30, 56.24, 56.21, 44.0, 43.6, 21.8, 20.7.

HRMS (APCI) Calculated for $[C_{16}H_{18}O_3N_2+H]^+$ 287.1390, found 287.1390.

2''-Methoxy-N,N-dimethyl-4''-nitro-[1,1':3',1''-terphenyl]-4'-amine and 2-methoxy-N,N-dimethyl-4-nitro-[1,1':4',1''-terphenyl]-2'-amine (3ak)

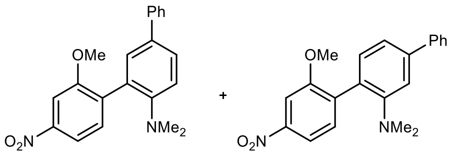

Prepared according to general procedure E (0.2 mmol scale). Purified by column chromatography (0-5% EtOAc in hexane). The inseparable mixture of regioisomers **3ak** was afforded as a red gum (33 mg, 47% yield, r.r. 1:1).

^{1}H NMR (400 MHz, $CDCl_3$) *δ* 7.92 – 7.85 (m, 4H), 7.67 – 7.60 (m, 2H), 7.59 – 7.24 (m, 15H), 7.17 – 7.13 (m, 1H), 3.92 (s, 3H), 3.91 (s, 3H), 2.58 (s, 6H), 2.56 (s, 6H).

^{13}C NMR (101 MHz, $CDCl_3$) *δ* 157.1, 157.0, 152.3, 151.3, 148.0, 147.9, 142.4, 142.3, 141.4, 140.7, 138.0, 137.7, 134.0, 132.2, 131.80, 131.78, 130.5, 129.0, 128.89, 128.85, 127.8, 127.6, 127.4, 126.88, 126.85, 120.1, 119.9, 118.5, 117.5, 117.2, 116.1, 106.5, 56.32, 56.30, 43.64, 43.59.

HRMS (ESI) Calculated for $[C_{21}H_{20}O_3N_2+H]^+$ 349.1547, found 349.1559.

1-Methyl-5-nitroindoline (5a)

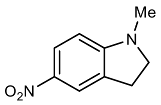

Prepared according to General Procedure D (3.0 mmol scale). No column chromatography required. The pure product **5a** was afforded as a yellow solid (567 mg, quant. yield).

^{1}H NMR ($CDCl_3$, 500 MHz) *δ* 8.06 (dd, *J* = 8.8, 2.3 Hz, 1H), 7.87 (dt, *J* = 2.6, 1.3 Hz, 1H), 6.25 (d, *J* = 8.8 Hz, 1H), 3.62 (t, *J* = 8.6 Hz, 2H), 3.06 (t, *J* = 8.6 Hz, 2H), 2.91 (s, 3H).

^{13}C NMR ($CDCl_3$, 126 MHz) δ 157.9, 138.1, 130.0, 126.9, 120.7, 103.5, 55.0, 34.0, 27.3.

HRMS (APCI) Calculated for $[C_9H_{10}O_2N_2+H]^+$ 179.0815, found 179.0817.

The data are in accordance with the literature.[18]

1-Ethyl-5-nitroindoline (5b)

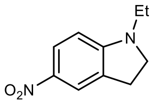

Prepared according to literature procedure D (2.0 mmol scale). No column chromatography required. The pure product **5b** was afforded as a brown solid (327 mg, 85% yield).

^{1}H NMR (400 MHz, $CDCl_3$) δ 8.03 (dd, *J* = 8.9, 2.3 Hz, 1H), 7.86 (dt, *J* = 2.5, 1.4 Hz, 1H), 6.24 (d, *J* = 8.9 Hz, 1H), 3.65 (t, *J* = 8.7 Hz, 2H), 3.30 (q, *J* = 7.2 Hz, 2H), 3.05 (t, *J* = 8.6 Hz, 2H), 1.20 (t, *J* = 7.2 Hz, 3H).

^{13}C NMR (101 MHz, $CDCl_3$) δ 157.0, 137.7, 130.0, 126.9, 120.9, 103.3, 51.5, 41.3, 27.1, 11.8.

HRMS (ESI) Calculated for $[C_{10}H_{12}O_2N_2+H]^+$ 193.0972, found 193.0970.

mp: 88-90 °C.

1-(2-(1,3-Dioxolan-2-yl)ethyl)-5-nitroindoline (5c)

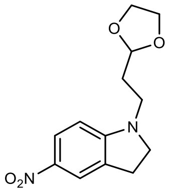

Prepared according to general procedure D (2.0 mmol scale). Purified by column chromatography (0-40% EtOAc in hexane). The pure product **5c** was afforded as a yellow solid (324 mg, 61% yield).

^{1}H NMR (500 MHz, $CDCl_3$) δ 8.03 (dd, *J* = 8.8, 2.3 Hz, 1H), 7.86 (s, 1H), 6.31 (d, *J* = 8.8 Hz, 1H), 4.93 (t, *J* = 4.4 Hz, 1H), 4.03 – 3.92 (m, 2H), 3.92 – 3.83 (m, 2H), 3.66 (t, *J* = 8.7 Hz, 2H), 3.43 – 3.37 (m, 2H), 3.10 – 3.03 (m, 2H), 2.01 – 1.94 (m, 2H).

^{13}C NMR (126 MHz, $CDCl_3$) δ 157.1, 137.9, 129.8, 126.9, 120.8, 103.5, 102.4, 65.2, 52.3, 42.1, 31.0, 27.2.

HRMS (ESI) Calculated for $[C_{13}H_{16}O_4N_2+Na]^+$ 287.1002, found 287.1008.

mp: 54-56 °C.

1-Methyl-6-nitro-1,2,3,4-tetrahydroquinoline (5d)

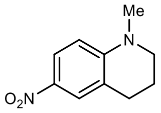

Prepared according to General Procedure D (3.0 mmol scale). Purified by column chromatography (0-10% EtOAc in hexane). The pure product **5d** was afforded as an orange solid (384 mg, 66% yield).

^{1}H NMR (CDCl$_3$, 400 MHz) δ 7.97 (dd, J = 9.2, 2.7 Hz, 1H), 7.83 (dd, J = 2.6, 1.3 Hz, 1H), 6.45 (d, J = 9.2 Hz, 1H), 3.44 – 3.37 (m, 2H), 3.03 (s, 3H), 2.77 (t, J = 6.3 Hz, 2H), 2.03 – 1.92 (m, 2H).

^{13}C NMR (CDCl$_3$, 101 MHz) δ 151.3, 136.5, 124.9, 124.8, 121.6, 108.8, 51.3, 39.1, 27.8, 21.5.

HRMS (ESI) Calculated for $[C_{10}H_{12}O_2N_2+H]^+$ 193.0972, found 193.0979.

The data are in accordance with the literature.[19]

5-Methyl-9-nitro-6,7-dihydro-5H-dibenzo[b,d]azepine (6a)

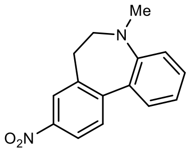

Prepared according to General Procedure E (0.2 mmol scale). Purified by column chromatography (0-5% EtOAc in hexane). The pure product **6a** was afforded as a yellow-orange solid (23 mg, 45% yield).

^{1}H NMR (CDCl$_3$, 400 MHz) δ 8.20 (dd, J = 8.4, 2.4 Hz, 1H), 8.13 (d, J = 2.4 Hz, 1H), 7.49 (d, J = 8.4 Hz, 1H), 7.42 (td, J = 7.7, 1.7 Hz, 1H), 7.35 (dd, J = 7.6, 1.7 Hz, 1H), 7.14 (td, J = 7.5, 1.2 Hz, 1H), 7.09 (d, J = 8.0 Hz, 1H), 3.48 (t, J = 6.6 Hz, 2H), 2.83 (t, J = 6.6 Hz, 2H), 2.78 (s, 3H).

^{13}C NMR (CDCl$_3$, 101 MHz) δ 148.8, 148.2, 146.9, 140.7, 133.7, 130.3, 129.4, 129.1, 122.7, 122.4, 122.2, 119.3, 63.7, 41.5, 33.0.

HRMS (ESI) Calculated for $[C_{15}H_{14}O_2N_2+Na]^+$ 277.0947, found 277.0946.

XRD: Recrystallised from MeCN as yellow-brown needles. Single crystal analysis confirms the structure drawn (CCDC deposition number: 2244917).

mp: 128-130 °C.

5-Ethyl-9-nitro-6,7-dihydro-5H-dibenzo[b,d]azepine (6b)

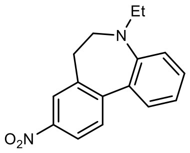

Prepared according to general procedure E (0.2 mmol scale). Purified by column chromatography (0-5% EtOAc in hexane). The pure product **6b** was afforded as a red oil (27 mg, 50% yield).

^{1}H NMR (400 MHz, CDCl$_3$) δ 8.20 (dd, J = 8.4, 2.4 Hz, 1H), 8.11 (d, J = 2.4 Hz, 1H), 7.48 (d, J = 8.4 Hz, 1H), 7.44 – 7.36 (m, 1H), 7.33 (dd, J = 7.5, 1.7 Hz, 1H), 7.17 – 7.06 (m, 2H), 3.46 (t, J = 6.6 Hz, 2H), 3.10 (q, J = 7.1 Hz, 2H), 2.80 (t, J = 6.6 Hz, 2H), 1.03 (t, J = 7.0 Hz, 3H).

^{13}C NMR (101 MHz, CDCl$_3$) δ 148.4, 147.9, 146.9, 140.9, 134.9, 129.9, 129.4, 128.7, 122.7, 122.3, 122.1, 120.4, 61.1, 47.3, 32.9, 13.3.

HRMS (ESI) Calculated for $[C_{16}H_{16}O_2N_2+H]^+$ 269.1285, found 269.1294.

5-(2-(1,3-Dioxolan-2-yl)ethyl)-9-nitro-6,7-dihydro-5H-dibenzo[b,d]azepine (6c)

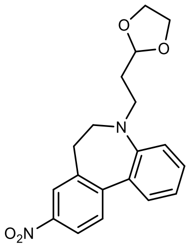

Prepared according to general procedure E (0.2 mmol scale). Purified by column chromatography (0-15% EtOAc in hexane). The pure product **6c** was afforded as an orange oil (15 mg, 22% yield).

^{1}H NMR (400 MHz, CDCl$_3$) δ 8.19 (dd, J = 8.4, 2.4 Hz, 1H), 8.10 (d, J = 2.4 Hz, 1H), 7.46 (d, J = 8.3 Hz, 1H), 7.40 (td, J = 7.7, 1.7 Hz, 1H), 7.32 (dd, J = 7.5, 1.7 Hz, 1H), 7.13 (t, J = 8.1, 7.6 Hz, 2H), 4.70 (t, J = 4.7 Hz, 1H), 3.93 – 3.81 (m, 2H), 3.79 – 3.67 (m, 2H), 3.47 (t, J = 6.6 Hz, 2H), 3.24 – 3.16 (m, 2H), 2.79 (t, J = 6.6 Hz, 2H), 1.81 – 1.72 (m, 2H).

^{13}C NMR (101 MHz, CDCl$_3$) δ 148.4, 147.4, 146.9, 140.8, 135.2, 130.0, 129.4, 128.6, 123.1, 122.3, 122.1, 120.6, 103.0, 64.9, 61.7, 47.8, 32.9, 32.1.

HRMS (ESI) Calculated for $[C_{19}H_{20}O_4N_2+H]^+$ 341.1496, found 341.1494.

5-Methyl-10-nitro-5,6,7,8-tetrahydrodibenzo[b,d]azocine (6d)

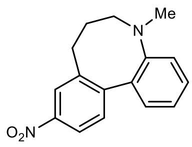

Prepared according to General Procedure E (0.2 mmol scale). Purified by column chromatography (0-5% EtOAc in hexane), followed by preparative thin layer chromatography. The pure product **6d** was afforded as a red gum (28 mg, 49% yield).

^{1}H NMR (CDCl$_3$, 400 MHz) δ 8.12 (dd, J = 8.5, 2.5 Hz, 1H), 7.96 (d, J = 2.4 Hz, 1H), 7.53 (d, J = 8.5 Hz, 1H), 7.29 – 7.22 (m, 1H), 6.99 (dd, J = 7.7, 1.8 Hz, 1H), 6.80 – 6.70 (m, 2H), 3.45 (ddd, J = 14.9, 11.8, 2.7 Hz, 1H), 2.99 (s, 3H), 2.94 – 2.83 (m, 2H), 2.78 (td, J = 12.8, 4.5 Hz, 1H), 2.19 – 2.06 (m, 1H), 1.33 – 1.22 (m, 1H).

^{13}C NMR (CDCl$_3$, 101 MHz) δ 151.2. 149.4, 147.1, 140.3, 135.5, 130.7, 129.7, 123.7, 122.9, 121.7, 116.9, 114.2, 49.4, 41.0, 31.0, 25.6.

HRMS (APCI) Calculated for $[C_{16}H_{16}O_2N_2+H]^+$ 269.1285, found 269.1273.

N-(3-(9,10-Ethanoanthracen-9(10H)-yl)propyl)-2-methoxy-N-methyl-4-nitroaniline (7a)

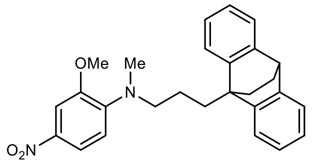

Prepared according to general procedure C (2.0 mmol scale). Purified by column chromatography (0-70% DCM in hexane). The pure product **7a** was afforded as a red solid (398 mg, 46% yield).

^{1}H NMR (400 MHz, CDCl$_3$) δ 7.89 (dd, J = 8.9, 2.5 Hz, 1H), 7.75 (d, J = 2.5 Hz, 1H), 7.30 – 7.23 (m, 2H), 7.20 – 7.15 (m, 2H), 7.13 – 7.05 (m, 4H), 6.85 (d, J = 8.9 Hz, 1H), 4.28 (t, J = 2.7 Hz, 1H), 4.00 (s, 3H), 3.64 (t, J = 7.6 Hz, 2H), 3.07 (s, 3H), 2.46 – 2.45 (m, 2H), 2.14 – 2.01 (m, 2H), 1.87 – 1.78 (m, 2H), 1.60 – 1.53 (m, 2H).

^{13}C NMR (101 MHz, CDCl$_3$) δ 150.2, 147.7, 145.4, 145.1, 140.4, 125.48, 125.47, 123.4, 121.1, 118.5, 115.7, 107.0, 56.2, 55.7, 44.9, 44.6, 40.5, 29.8, 28.4, 27.7, 23.4.

HRMS (APCI) Calculated for $[C_{27}H_{28}O_3N_2+H]^+$ 429.2173, found 429.2171.

mp: 161-164 °C.

2-Methoxy-N-methyl-4-nitro-N-(3-phenyl-3-(4-(trifluoromethyl)phenoxy)propyl)aniline (7b)

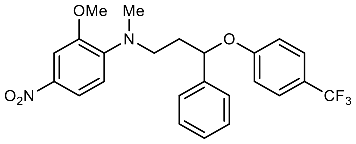

Prepared according to general procedure C (2.0 mmol scale). Purified by column chromatography (0-20% EtOAc in hexane). The pure product **7b** was afforded as a yellow gum (563 mg, 62% yield).

^{1}H NMR (400 MHz, CDCl$_3$) δ 7.78 (dd, J = 8.9, 2.5 Hz, 1H), 7.57 (d, J = 2.5 Hz, 1H), 7.41 – 7.35 (m, 2H), 7.34 – 7.28 (m, 2H), 7.27 – 7.21 (m, 3H), 6.81 (d, J = 8.6 Hz, 2H), 6.73 (d, J = 9.0 Hz, 1H), 5.13 (dd, J = 8.0, 4.5 Hz, 1H), 3.75 (s, 3H), 3.61 – 3.51 (m, 1H), 3.50 – 3.41 (m, 1H), 2.92 (s, 3H), 2.30 – 2.08 (m, 2H).

^{13}C NMR (101 MHz, CDCl$_3$) δ 160.4, 150.1, 147.3, 140.7, 140.4, 129.0, 128.1, 126.9 (q, J = 3.7 Hz), 125.8, 124.4 (q, J = 272.0 Hz), 123.08 (q, J = 32.8 Hz), 118.3, 115.71, 115.66, 106.9, 78.2, 55.8, 51.0, 40.2, 36.7.

^{19}F NMR (CDCl$_3$, 376 MHz) δ -61.6.

HRMS (APCI) Calculated for $[C_{24}H_{23}O_4N_2F_3]^+$ 461.1683, found 461.1682.

(S)-2-Methoxy-N-methyl-N-(3-(naphthalen-1-yloxy)-3-(thiophen-2-yl)propyl)-4-nitroaniline (7c)

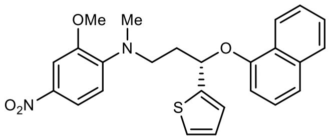

Prepared according to general procedure C (2.0 mmol scale). Purified by column chromatography (0-15% EtOAc in hexane). The pure product **7c** was isolated as an orange gum (513 mg, 57% yield).

^{1}H NMR (400 MHz, CDCl$_3$) δ 8.35 – 8.26 (m, 1H), 7.83 – 7.74 (m, 2H), 7.54 – 7.45 (m, 2H), 7.43 (d, J = 2.5 Hz, 1H), 7.37 (d, J = 8.2 Hz, 1H), 7.23 – 7.15 (m, 2H), 7.02 (dt, J = 3.5, 0.9 Hz, 1H), 6.94 (dd, J

= 5.0, 3.5 Hz, 1H), 6.77 (d, *J* = 8.9 Hz, 1H), 6.67 (d, *J* = 7.6 Hz, 1H), 5.61 (dd, *J* = 7.9, 4.8 Hz, 1H), 3.85 – 3.74 (m, 1H), 3.65 (s, 3H), 3.63 – 3.51 (m, 1H), 2.98 (s, 3H), 2.55 – 2.34 (m, 2H).

^{13}C NMR (101 MHz, CDCl$_3$) δ 153.2, 150.1, 147.2, 144.9, 140.5, 134.7, 127.8, 126.8, 126.5, 126.0, 125.7, 125.4, 124.9, 124.6, 122.0, 120.9, 118.2, 115.8, 106.7, 106.7, 74.1, 55.7, 50.8, 40.1, 36.9.

HRMS (APCI) Calculated for [$C_{25}H_{24}O_4N_2S$+H]$^+$ 449.1530, found 449.1526.

***N*-(3-(10,11-Dihydro-5*H*-dibenzo[a,d][7]annulen-5-ylidene)propyl)-2-methoxy-*N*-methyl-4-nitroaniline (7d)**

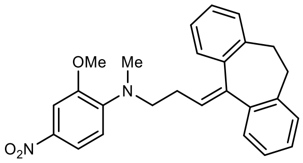

Prepared according to General Procedure C (2.5 mmol scale). Purified by column chromatography (0-10% EtOAc in hexane). The pure product **7d** was afforded as a yellow solid (229 mg, 22% yield).

^{1}H NMR (CDCl$_3$, 400 MHz) δ 7.78 (dd, *J* = 8.9, 2.5 Hz, 1H), 7.63 (d, *J* = 2.5 Hz, 1H), 7.23 – 7.18 (m, 3H), 7.17 – 7.08 (m, 4H), 7.08 – 7.01 (m, 1H), 6.69 (d, *J* = 9.0 Hz, 1H), 5.82 (t, *J* = 7.7 Hz, 1H), 3.79 (s, 3H), 3.46 – 3.22 (m, 4H), 3.06 – 3.90 (m, 1H), 2.89 (s, 3H), 2.89 – 2.69 (m, 1H), 2.53 – 2.31 (m, 2H).

^{13}C NMR (CDCl$_3$, 101 MHz) δ 149.8, 147.3, 144.6, 141.0, 140.0, 139.8, 139.4, 137.0, 130.1, 128.5, 128.1 (2C), 128.0, 127.6, 127.2, 126.1, 125.8, 118.3, 115.3, 106.7, 55.7, 54.4, 40.0, 33.8, 32.0, 27.9.

HRMS (ESI) Calculated for [$C_{26}H_{26}O_3N_2$+Na]$^+$ 437.1836, found 437.1824.

mp: 115-118 °C.

***N*-(3-(9,10-Ethanoanthracen-9(10*H*)-yl)propyl)-2'-methoxy-*N*-methyl-4'-nitro-[1,1'-biphenyl]-2-amine (8a)**

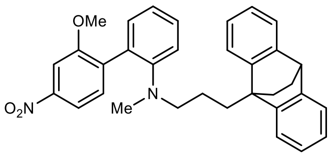

Prepared according to general procedure E (0.2 mmol scale). Purified by column chromatography (0-5% EtOAc in hexane). The pure product **8a** was isolated as a yellow solid (57 mg, 56% yield).

^{1}H NMR (400 MHz, CDCl$_3$) δ 7.90 (dd, J = 8.3, 2.2 Hz, 1H), 7.84 (d, J = 2.2 Hz, 1H), 7.52 (d, J = 8.3 Hz, 1H), 7.40 (ddd, J = 9.0, 7.3, 1.8 Hz, 1H), 7.28 – 7.23 (m, 2H), 7.21 (dd, J = 7.6, 1.8 Hz, 1H), 7.14 – 7.09 (m, 1H), 7.09 – 7.00 (m, 6H), 4.25 (t, J = 2.7 Hz, 1H), 3.84 (s, 3H), 3.02 (t, J = 7.3 Hz, 2H), 2.68 (s, 3H), 2.22 – 2.14 (m, 2H), 1.83 – 1.73 (m, 4H), 1.47 – 1.38 (m, 2H).

^{13}C NMR (101 MHz, CDCl$_3$) δ 157.2, 152.0, 148.0, 145.4, 145.1, 138.1, 132.0, 131.6, 131.0, 129.3, 125.4, 125.3, 123.5, 122.2, 121.2, 120.1, 115.9, 106.2, 57.5, 56.2, 44.8, 44.6, 41.3, 29.8, 28.6, 27.7, 22.8.

HRMS (APCI) Calculated for [$C_{33}H_{32}O_3N_2$+H]$^+$ 505.2486. Found 505.2485.

mp: 61-63 °C.

2'-Methoxy-N-methyl-4'-nitro-N-(3-phenyl-3-(4-(trifluoromethyl)phenoxy)propyl)-[1,1'-biphenyl]-2-amine (8b)

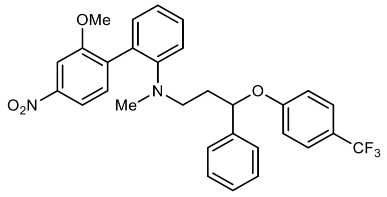

Prepared according to general procedure E (0.2 mmol scale). Purified by column chromatography (0-5% EtOAc in hexane). The pure product **8b** was afforded as a red gum (55 mg, 51% yield).

^{1}H NMR (400 MHz, CDCl$_3$) δ 7.75 (d, J = 8.3 Hz, 1H), 7.68 (d, J = 2.2 Hz, 1H), 7.35 – 7.13 (m, 7H), 7.09 – 7.01 (m, 4H), 6.97 (t, J = 7.4 Hz, 1H), 6.67 (d, J = 8.5 Hz, 2H), 4.84 – 4.76 (m, 1H), 3.69 (s, 3H), 2.89 – 2.72 (m, 2H), 2.52 (s, 3H), 1.91 – 1.78 (m, 1H), 1.72 – 1.59 (m, 1H).

^{13}C NMR (101 MHz, CDCl$_3$) δ 160.5, 157.1, 151.6, 147.9, 140.9, 137.8, 131.9, 131.6, 130.7, 129.3, 128.9, 128.0, 126.8 (q, J = 3.8 Hz), 125.7, 124.5 (q, J = 271.4 Hz), 123.0 (q, J = 27.2 Hz), 122.2, 119.9, 115.8 (2C), 106.3, 78.7, 56.1, 52.9, 41.2, 36.6.

^{19}F NMR (CDCl$_3$, 376 MHz) δ -61.6.

HRMS (APCI) Calculated for [$C_{30}H_{27}O_4N_2F_3$]$^+$ 537.1996, found 537.1996.

(S)-2'-Methoxy-N-methyl-N-(3-(naphthalen-1-yloxy)-3-(thiophen-2-yl)propyl)-4'-nitro-[1,1'-biphenyl]-2-amine (8c)

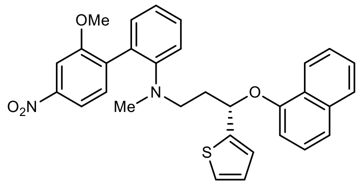

Prepared according to general procedure E (0.2 mmol scale). Purified by column chromatography (0-5% EtOAc in hexane). The pure product **8c** was afforded as a yellow gum (9 mg, 9% yield).

^{1}H NMR (400 MHz, CDCl$_3$) δ 8.23 – 8.17 (m, 1H), 7.83 – 7.73 (m, 2H), 7.70 (d, J = 2.2 Hz, 1H), 7.52 – 7.43 (m, 2H), 7.40 – 7.32 (m, 3H), 7.24 – 7.14 (m, 3H), 7.12 (dd, J = 7.6, 1.8 Hz, 1H), 7.05 (t, J = 7.5 Hz, 1H), 6.89 (dd, J = 5.0, 3.5 Hz, 1H), 6.87 – 6.82 (m, 1H), 6.61 (d, J = 7.7 Hz, 1H), 5.39 – 5.34 (m, 1H), 3.68 (s, 3H), 3.03 – 2.92 (m, 2H), 2.60 (s, 3H), 2.24 – 2.14 (m, 1H), 2.03 – 1.90 (m, 1H).

^{13}C NMR (101 MHz, CDCl$_3$) δ 157.0, 153.3, 151.5, 147.9, 145.0, 137.7, 134.6, 131.9, 131.6, 130.9, 129.3, 127.6, 126.7, 126.5, 126.1, 125.7, 125.4, 124.9, 124.5, 122.3, 122.1, 120.8, 120.0, 115.8, 107.0, 106.2, 74.6, 56.0, 52.8, 41.5, 36.6.

HRMS (APCI) Calculated for $[C_{31}H_{28}O_4N_2S]^+$ 525.1843, found 525.1845.

N-(3-(10,11-Dihydro-5H-dibenzo[a,d][7]annulen-5-ylidene)propyl)-2'-methoxy-N-methyl-4'-nitro-[1,1'-biphenyl]-2-amine (8d)

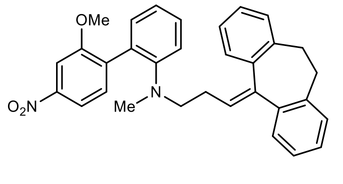

Prepared according to General Procedure E (0.1 mmol scale, 0.26 eq. aniline). Purified by column chromatography (0-10% EtOAc in hexane), followed by preparative thin layer chromatography (10% EtOAc in hexane). The pure product **8d** was afforded as an orange gum (17 mg, 34% yield).

^{1}H NMR (CDCl$_3$, 400 MHz) δ 7.68 – 7.61 (m, 2H), 7.37 – 7.28 (m, 2H), 7.18 – 7.02 (m, 10H), 6.98 (d, J = 7.5 Hz, 1H), 5.49 (t, J = 7.3 Hz, 1H), 3.72 (s, 3H), 3.30 – 3.10 (m, 2H), 2.96 – 2.63(m, 4H), 2.41 (s, 3H), 2.18 – 2.04 (m, 2H).

^{13}C NMR (CDCl$_3$, 101 MHz) δ 157.0, 152.1, 147.8, 143.5, 141.2, 140.0, 139.4, 137.9, 137.0, 131.9, 131.5, 131.0, 130.1, 129.2, 129.1, 128.6, 128.4, 128.1, 127.6, 127.3, 126.2, 125.7, 122.1, 119.9, 115.8, 106.0, 56.0, 55.8, 40.6, 33.9, 32.1, 27.9.

HRMS (ESI) Calculated for $[C_{32}H_{30}O_3N_2+H]^+$ 491.2329, found 491.2316.

2-Iodophenyl 4-chlorobenzenesulfonate (9a)

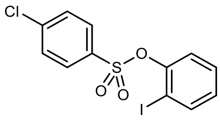

Prepared according to a literature procedure (3.0 mmol scale).[20] The pure product **9a** was afforded as a white solid (868 mg, 73% yield).

^{1}H NMR (500 MHz, CDCl$_3$) δ 7.90 – 7.83 (m, 2H), 7.78 – 7.75 (m, 1H), 7.55 – 7.49 (m, 2H), 7.39 – 7.34 (m, 2H), 7.04 – 6.97 (m, 1H).

^{13}C NMR (101 MHz, CDCl$_3$) δ 149.9, 141.5, 140.3, 134.3, 130.4, 129.8, 129.7, 128.8, 123.2, 90.1.

LRMS (APCI) Calculated for $[C_{12}H_8O_3ClIS+H]^+$ 394.9, found 394.9.

The data are in accordance with the literature.[21]

2'-Methoxy-N2,N2-dimethyl-[1,1'-biphenyl]-2,4'-diamine (10b)

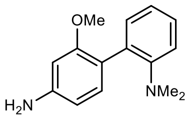

Prepared according to General Procedure F (0.1 mmol scale). Purified by column chromatography (0-50% EtOAc in hexane). The pure product **10b** was afforded as a grey solid (16 mg, 65%).

^{1}H NMR (400 MHz, CDCl$_3$) δ 7.23 (ddd, *J* = 8.0, 7.2, 1.8 Hz, 1H), 7.17 (dd, *J* = 7.5, 1.7 Hz, 1H), 7.11 –7.07 (m, 1H), 7.02 (dd, *J* = 8.1, 1.2 Hz, 1H), 6.95 (td, *J* = 7.4, 1.2 Hz, 1H), 6.37 – 6.32 (m, 2H), 3.74 (s, 3H), 3.71 (brs, 2H), 2.55 (s, 6H).

^{13}C NMR (101 MHz, CDCl$_3$) δ 157.4, 147.0, 132.9, 131.9, 131.4, 128.8, 127.8, 118.1, 112.8, 107.5, 99.1, 55.6, 43.6.

HRMS (ESI) Calculated for $[C_{15}H_{18}ON_2+H]^+$ 243.1492, found 243.1497.

2-Methoxy-2'-morpholino-[1,1'-biphenyl]-4-amine (10n)

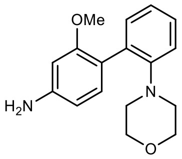

Prepared according to General Procedure F (0.1 mmol scale). Purified by column chromatography (0-50% EtOAc in hexane). The pure product **10n** was afforded as a yellow oil (26 mg, 93%).

^{1}H NMR (400 MHz, $CDCl_3$) δ 7.29 – 7.24 (m, 1H), 7.22 (dd, *J* = 7.5, 1.7 Hz, 1H), 7.19 – 7.14 (m, 1H), 6.42 – 6.34 (m, 2H), 7.08 – 6.99 (m, 2H), 3.74 (s, 3H), 3.55 (dd, *J* = 5.5, 3.5 Hz, 4H), 2.82 (dd, *J* = 5.6, 3.5 Hz, 4H).

^{13}C NMR (101 MHz, $CDCl_3$) δ 157.4, 150.9, 146.4, 132.6, 132.1, 128.0, 122.5, 120.4, 118.5, 107.5, 99.1, 67.4, 55.5, 51.5.

HRMS (APCI) Calculated for $[C_{17}H_{20}O_2N_2+H]^+$ 285.1598, found 285.1594.

N-Methyl-4-nitro-N-phenyl-2-vinylaniline (11)

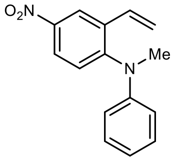

Isolated as a side product following General Procedure E for the synthesis of **6a** (0.2 mmol scale). Purified by column chromatography (0-5% EtOAc in hexane). The pure product **11** was afforded as a brown gum (9 mg, 17% yield).

^{1}H NMR ($CDCl_3$, 400 MHz) δ 8.43 (d, *J* = 2.7 Hz, 1H), 8.10 (dd, *J* = 8.8, 2.7 Hz, 1H), 7.27 – 7.19 (m, 3H), 6.89 (tt, *J* = 7.3, 1.1 Hz, 1H), 6.67 (dt, *J* = 7.9, 1.1 Hz, 2H), 6.70 (dd, *J* = 17.6, 11.0 Hz, 1H), 5.81 (dd, *J* = 17.6, 0.8 Hz, 1H), 5.31 (dd, *J* = 10.9, 0.8 Hz, 1H), 3.27 (s, 3H).

^{13}C NMR ($CDCl_3$, 101 MHz) δ 152.8, 148.6, 144.7, 135.5, 132.4, 129.4, 126.7, 124.1, 122.9, 120.5, 117.4, 117.1, 40.7.

HRMS (HESI) Calculated for $[C_{15}H_{14}O_2N_2+Na]^+$ 277.0947, found 277.0946.

9. References

1. S. Sil, A. S. Bhaskaran, S. Chakraborty, B. Singh, R. Kuniyil and S. K. Mandal, *J. Am. Chem. Soc.* **2022**, *144*, 22611-22621.

2. J.-D. Chai and M. Head-Gordon, *Phys. Chem. Chem. Phys*., **2008**, *10*, 6615-6620.

3. F. Weigend and R. Ahlrichs, *Phys. Chem. Chem. Phys*., **2005**, *7*, 3297–3305.

4. M. Bursch, J.-M. Mewes, A. Hansen and S. Grimme, Angew. Chem. Int. Ed., 2022, 61, e202205735.

5. Gaussian 16, Revision C.01, M. J. Frisch, G. W. Trucks, H. B. Schlegel, G. E. Scuseria, M. A. Robb, J. R. Cheeseman, G. Scalmani, V. Barone, G. A. Petersson, H. Nakatsuji, X. Li, M. Caricato, A. V. Marenich, J. Bloino, B. G. Janesko, R. Gomperts, B. Mennucci, H. P. Hratchian, J. V. Ortiz, A. F. Izmaylov, J. L. Sonnenberg, D. Williams-Young, F. Ding, F. Lipparini, F. Egidi, J. Goings, B. Peng, A. Petrone, T. Henderson, D. Ranasinghe, V. G. Zakrzewski, J. Gao, N. Rega, G. Zheng, W. Liang, M. Hada, M. Ehara, K. Toyota, R. Fukuda, J. Hasegawa, M. Ishida, T. Nakajima, Y. Honda, O. Kitao, H. Nakai, T. Vreven, K. Throssell, J. A. Montgomery, Jr., J. E. Peralta, F. Ogliaro, M. J. Bearpark, J. J. Heyd, E. N. Brothers, K. N. Kudin, V. N. Staroverov, T. A. Keith, R. Kobayashi, J. Normand, K. Raghavachari, A. P. Rendell, J. C. Burant, S. S. Iyengar, J. Tomasi, M. Cossi, J. M. Millam, M. Klene, C. Adamo, R. Cammi, J. W. Ochterski, R. L. Martin, K. Morokuma, O. Farkas, J. B. Foresman, and D. J. Fox, Gaussian, Inc., Wallingford CT, **2016**.

6. A. V. Marenich, C. J. Cramer and D. G. Truhlar, *J. Phys. Chem. B*, **2009**, *113*, 6378-6396.

7. A. Buchynskyy, J. R. Gillespie, Z. M. Herbst, R. M. Ranade, F. S. Buckner and M. H. Gelb, *ACS Med. Chem. Lett.* **2017**, *8*, 886-891.

8. A. Banerjee, C. Grewer, L. Ramakrishnan, J. Jäger, A. Gameiro, H.-G. A. Breitinger, K. R. Gee, B. K. Carpenter and G. P. Hess, *J. Org. Chem.* **2003**, *68*, 8361-8367.

9. *Int. Pat.*, WO2012151525A1, **2012**.

10. S. A. Gamage, D. P. Figgitt, S. J. Wojcik, R. K. Ralph, A. Ransijn, J. Mauel, V. Yardley, D. Snowdon, S. L. Croft and W. A. Denny, *J. Med. Chem.* **1997**, *40*, 2634-2642.

11. *US Pat.*, US2008227784A1, **2008**.

12. *Int. Pat.*, WO2020190912A1, **2020**.

13. T. Pirali, F. Zhang, A. H. Miller, J. L. Head, D. McAusland and M. F. Greaney, *Angew. Chem. Int. Ed.* **2012**, *51*, 1006-1009.

14. B. V. Moreira, A. C. A. Muraca, C. Raminelli, *Synthesis* **2017**, *49*, 1093-1102.

15. H. Jiang, Y. Zhang, W. Xiong, J. Cen, L. Wang, R. Cheng, C. Qi and W. Wu, *Org. Lett.* **2019**, *21*, 345-349.

16. B. Michel and M. F. Greaney, *Org. Lett.* **2014**, *16*, 2684-2687.

17. R. K. Hallani, M. Moser, H. Bristow, M. V. C. Jenart, H. Faber, M. Neophytou, E. Yarali, A. F. Paterson, T. D. Anthopoulos and I. McCulloch, *J. Org. Chem.* **2020**, *85*, 277-283.

18. *Int. Pat.*, WO2018171575A1, **2018**.

19. *Int. Pat.*, WO2009042907A1, **2009**.

20. J.-A. García-López, M. Çetin and M. F. Greaney, *Angew. Chem. Int. Ed.* **2015**, *54*, 2156-2159.

21. Y. Ueta, K. Mikami and S. Ito, *Angew. Chem. Int. Ed.* **2016**, *55*, 7525-7529.

10. NMR Spectra

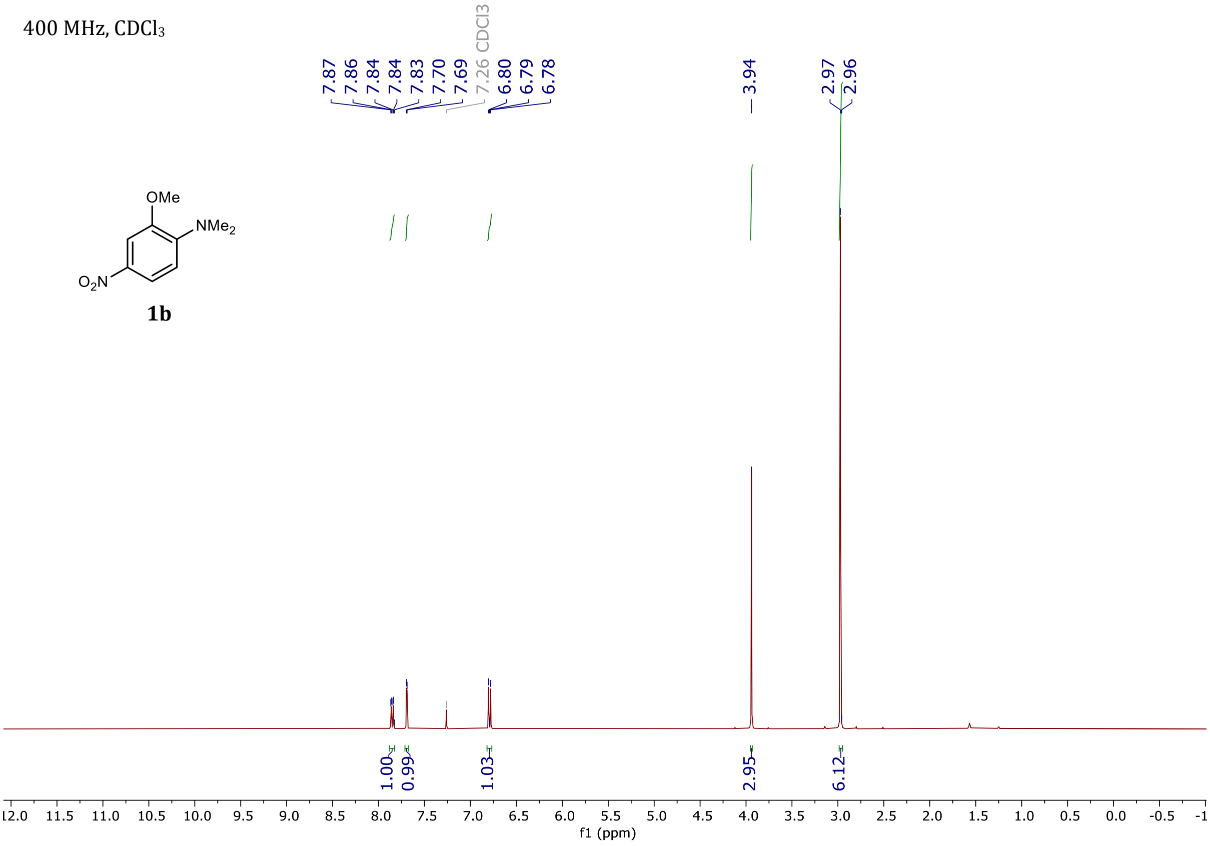

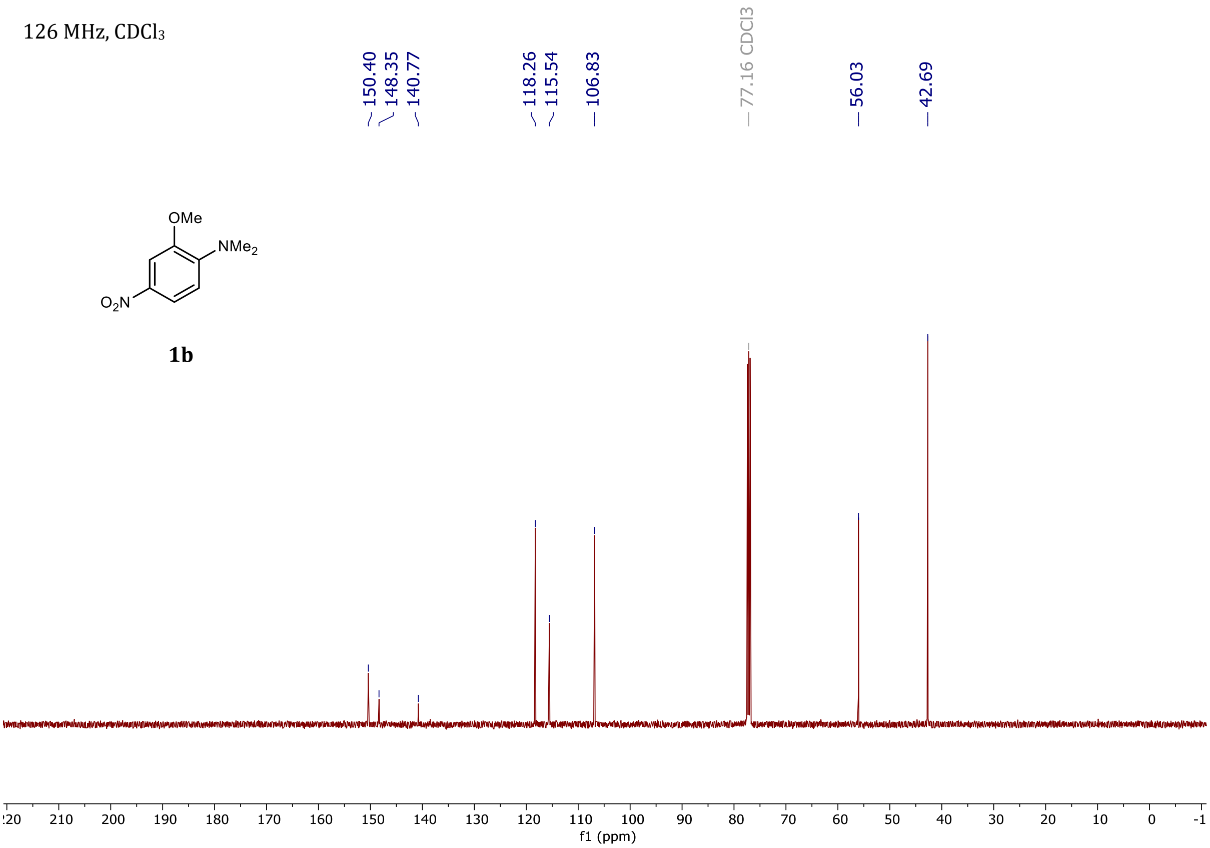

400 MHz, $CDCl_3$

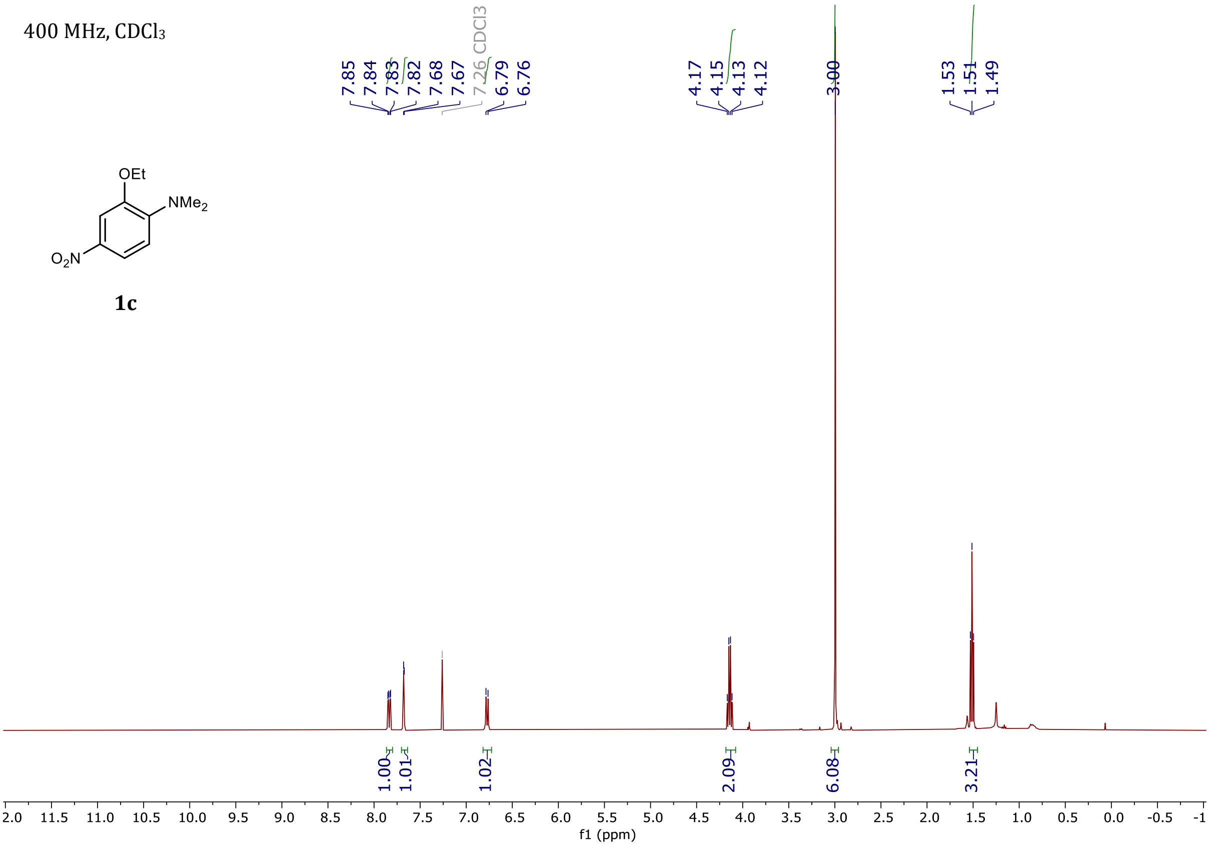

1c

101 MHz, $CDCl_3$

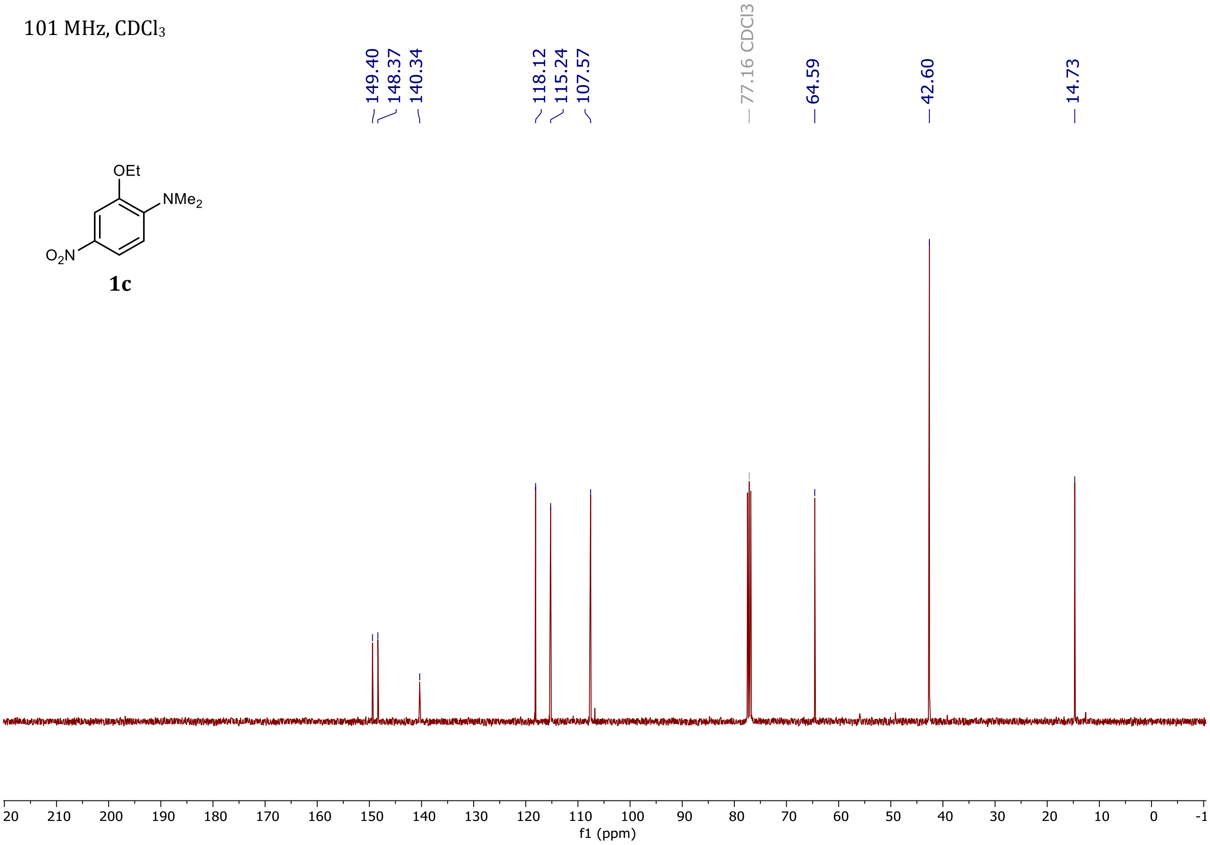

1c

500 MHz, $CDCl_3$

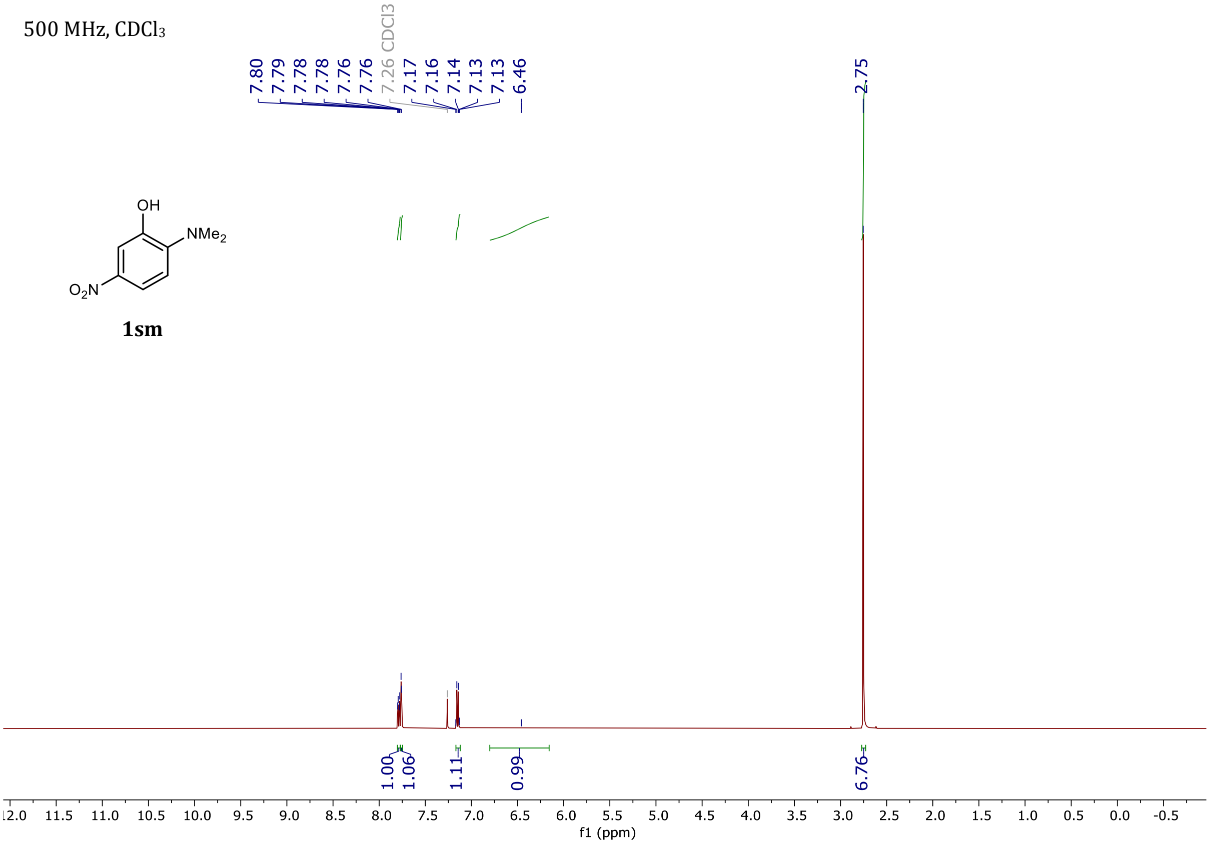

126 MHz, $CDCl_3$

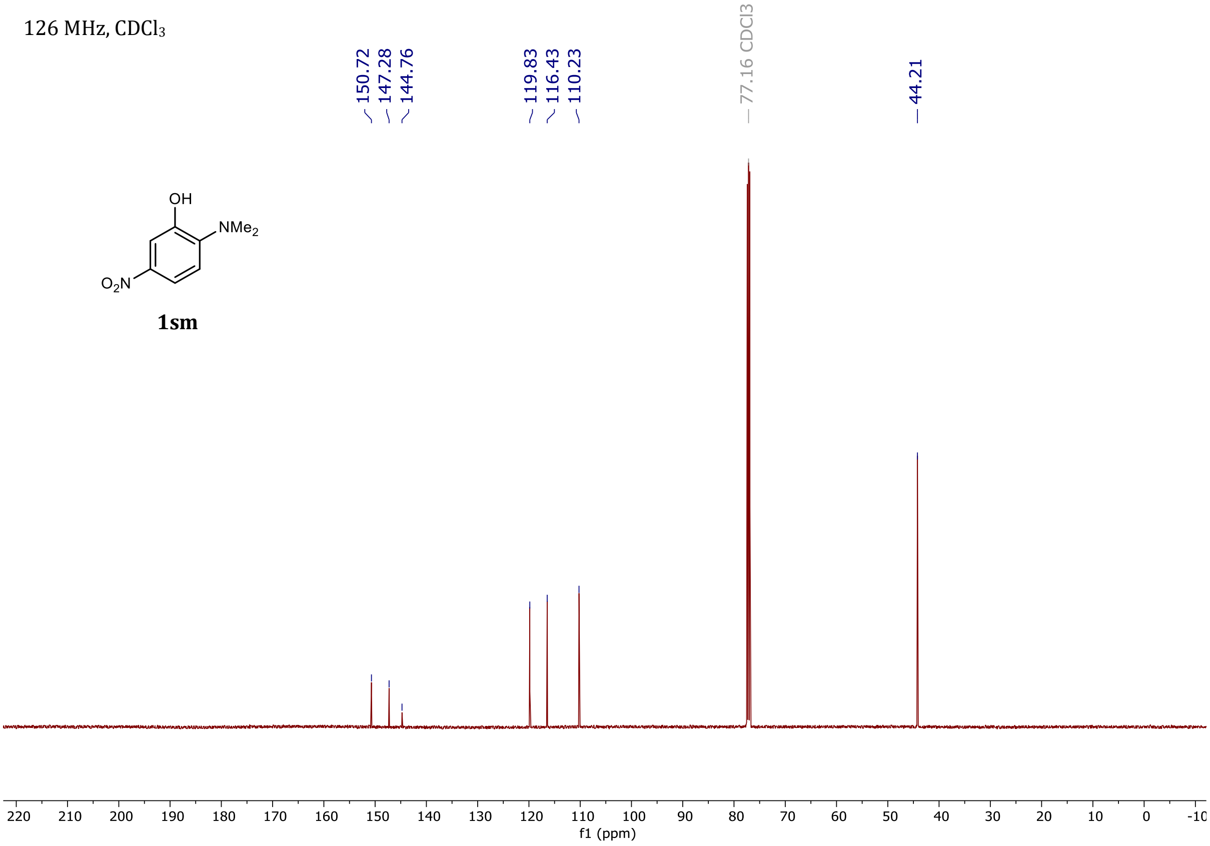

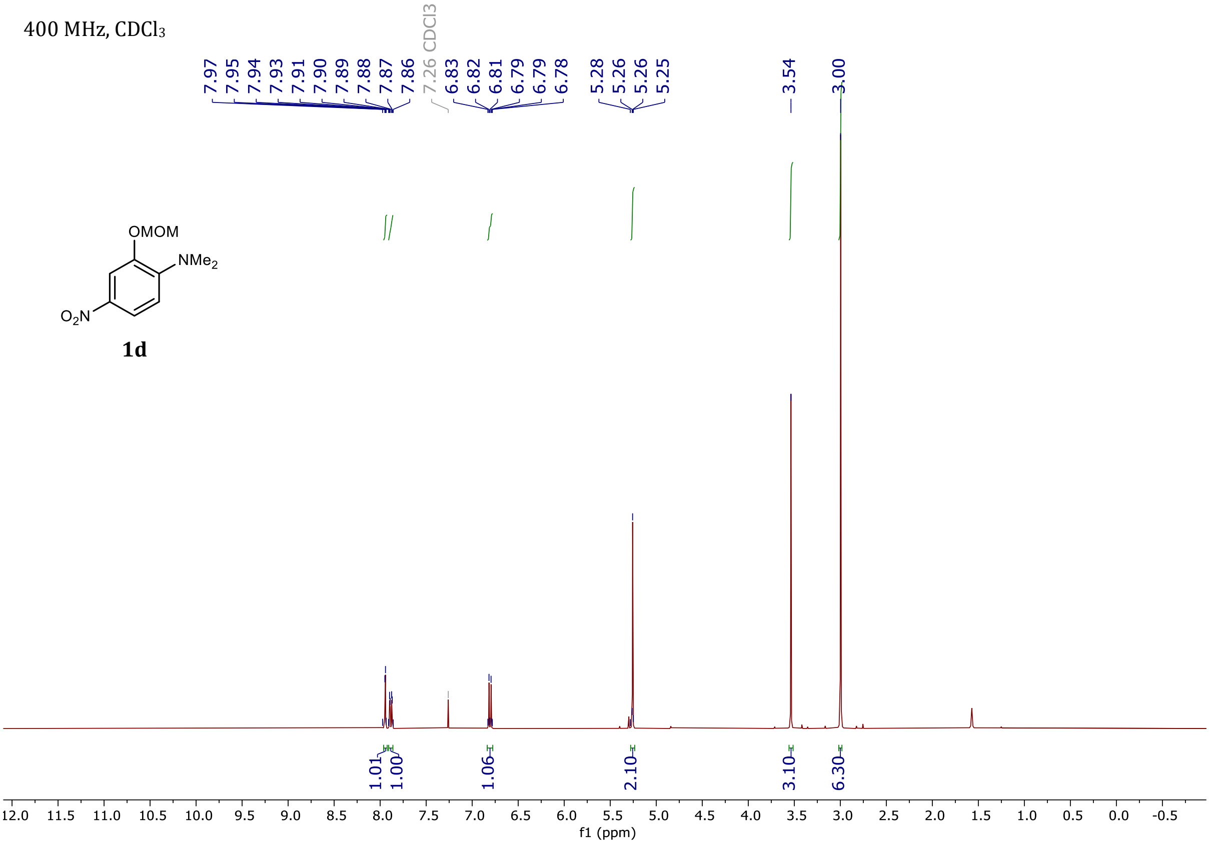
400 MHz, CDCl3
7.97
7.95
7.94
7.93
7.91
7.90
7.89
7.88
7.87
7.86
7.26 CDCl3
6.83
6.82
6.81
6.79
6.79
6.78
5.28
5.26
5.26
5.25
3.54
3.00
OMOM
NMe2
O2N
1d
1.01
1.00
1.06
2.10
3.10
6.30
f1 (ppm)

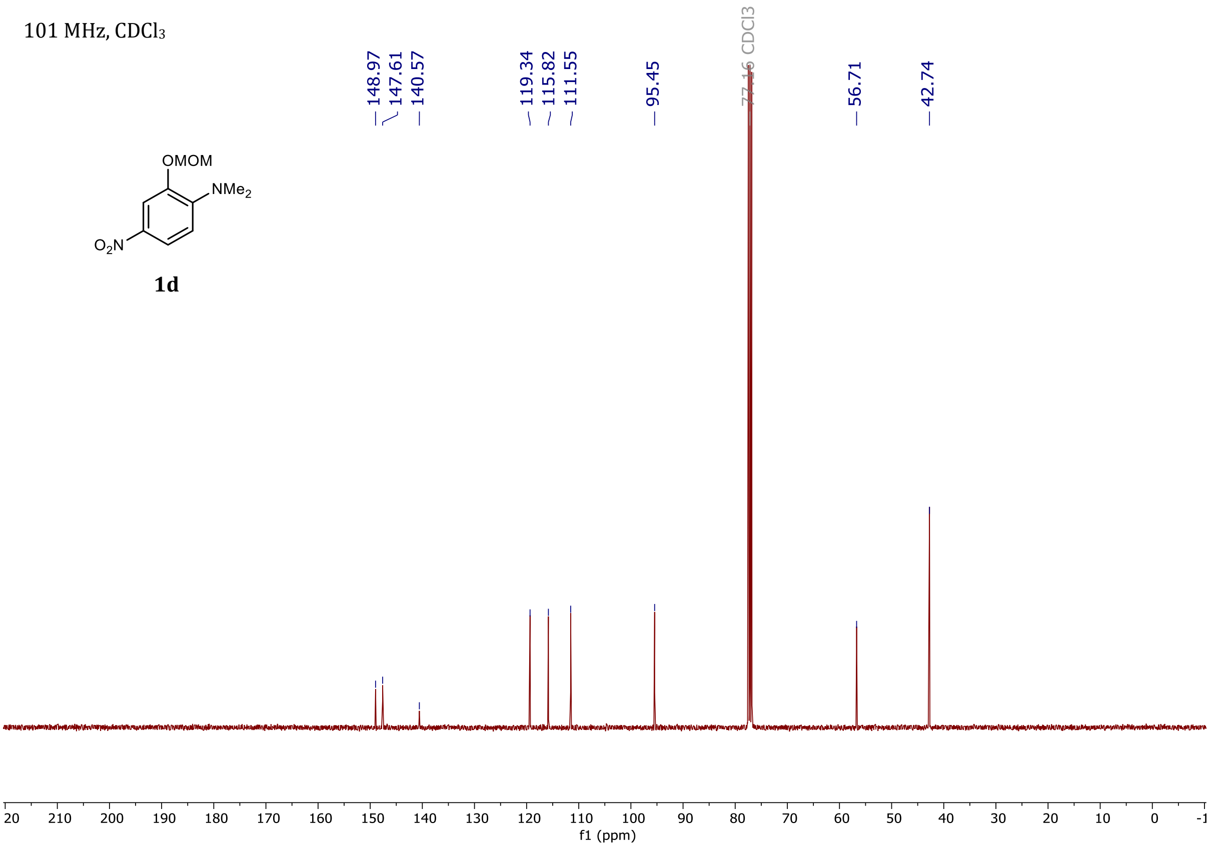
101 MHz, CDCl3
148.97
147.61
140.57
119.34
115.82
111.55
95.45
77.16 CDCl3
56.71
42.74
OMOM
NMe2
O2N
1d
f1 (ppm)

400 MHz, $CDCl_3$

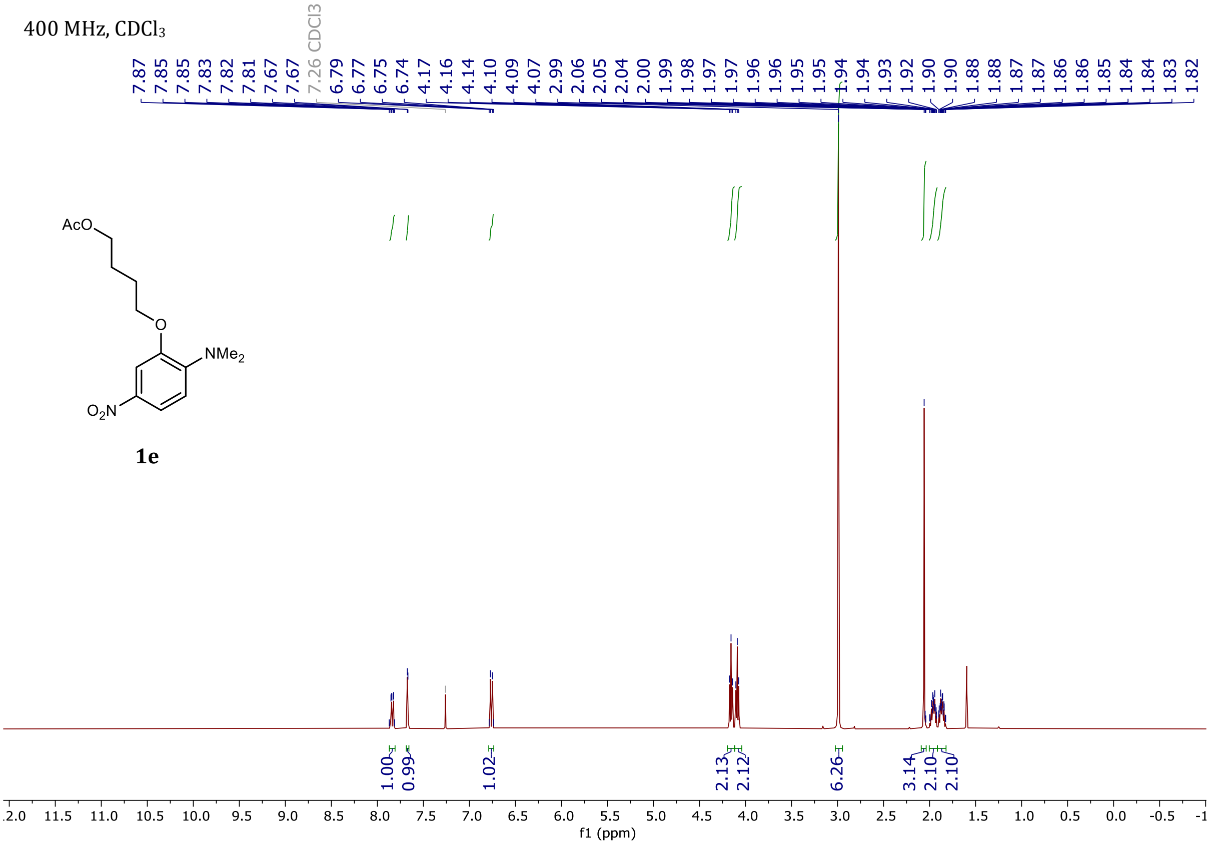

101 MHz, $CDCl_3$

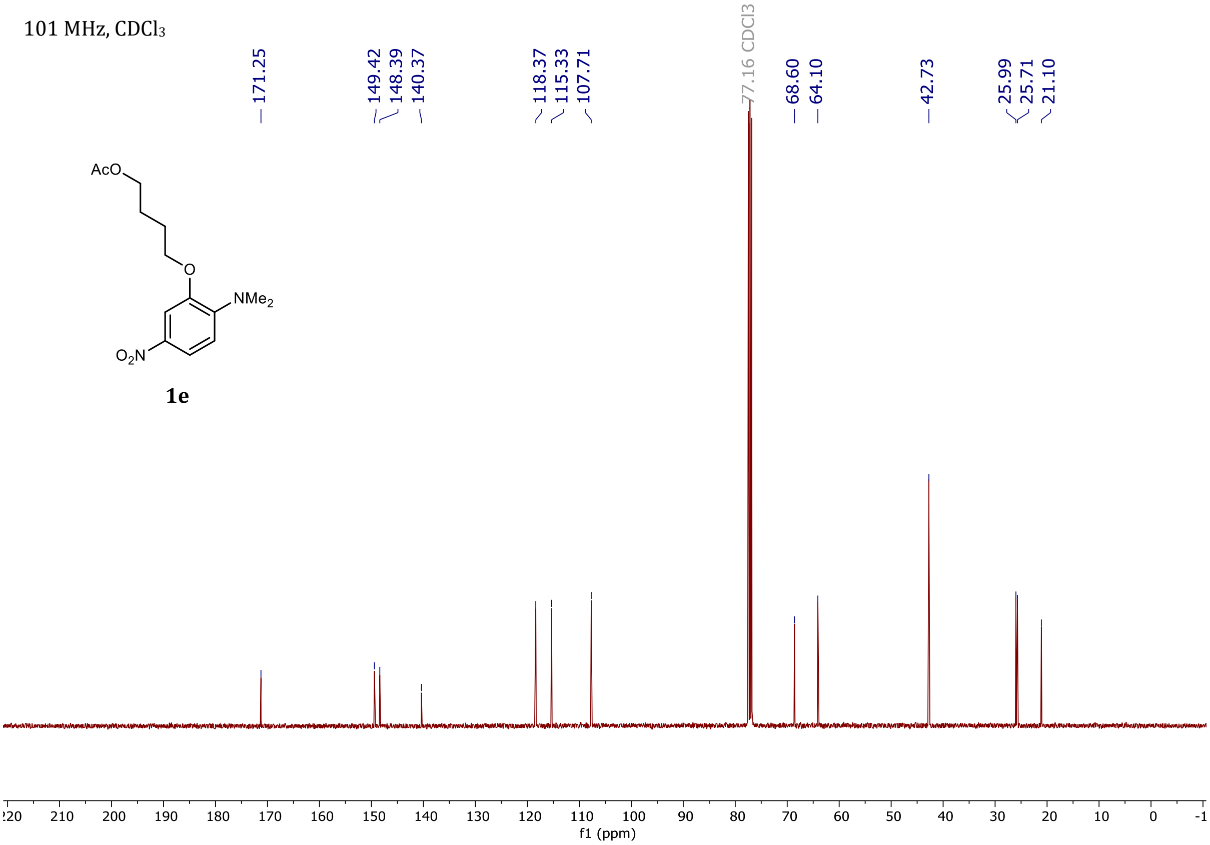

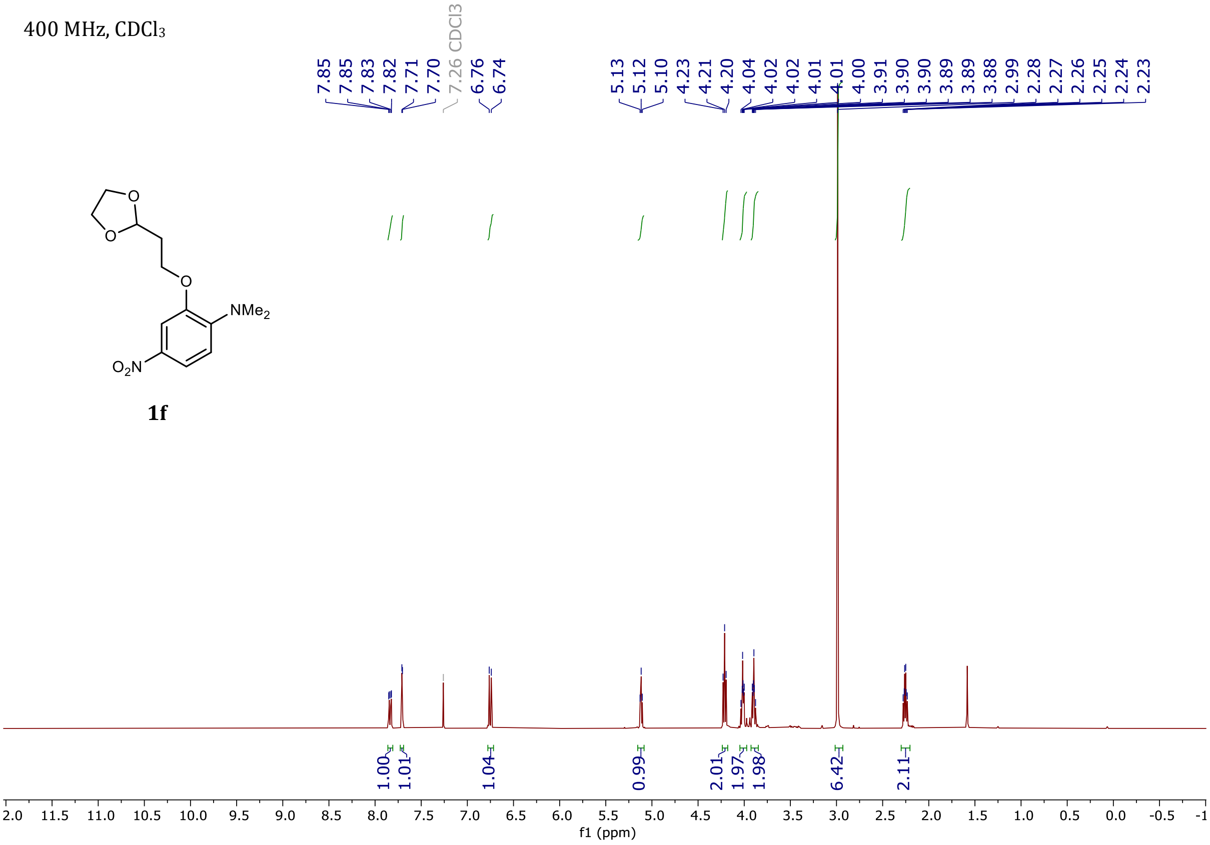
400 MHz, CDCl3
7.85 7.85 7.83 7.82 7.71 7.70 7.26 CDCl3 6.76 6.74 5.13 5.12 5.10 4.23 4.21 4.20 4.04 4.02 4.01 4.01 4.00 3.91 3.90 3.90 3.89 3.89 3.88 2.99 2.28 2.27 2.26 2.25 2.24 2.23
NMe2
O2N
1f
1.00 1.01 1.04 0.99 2.01 1.97 1.98 6.42 2.11
f1 (ppm)

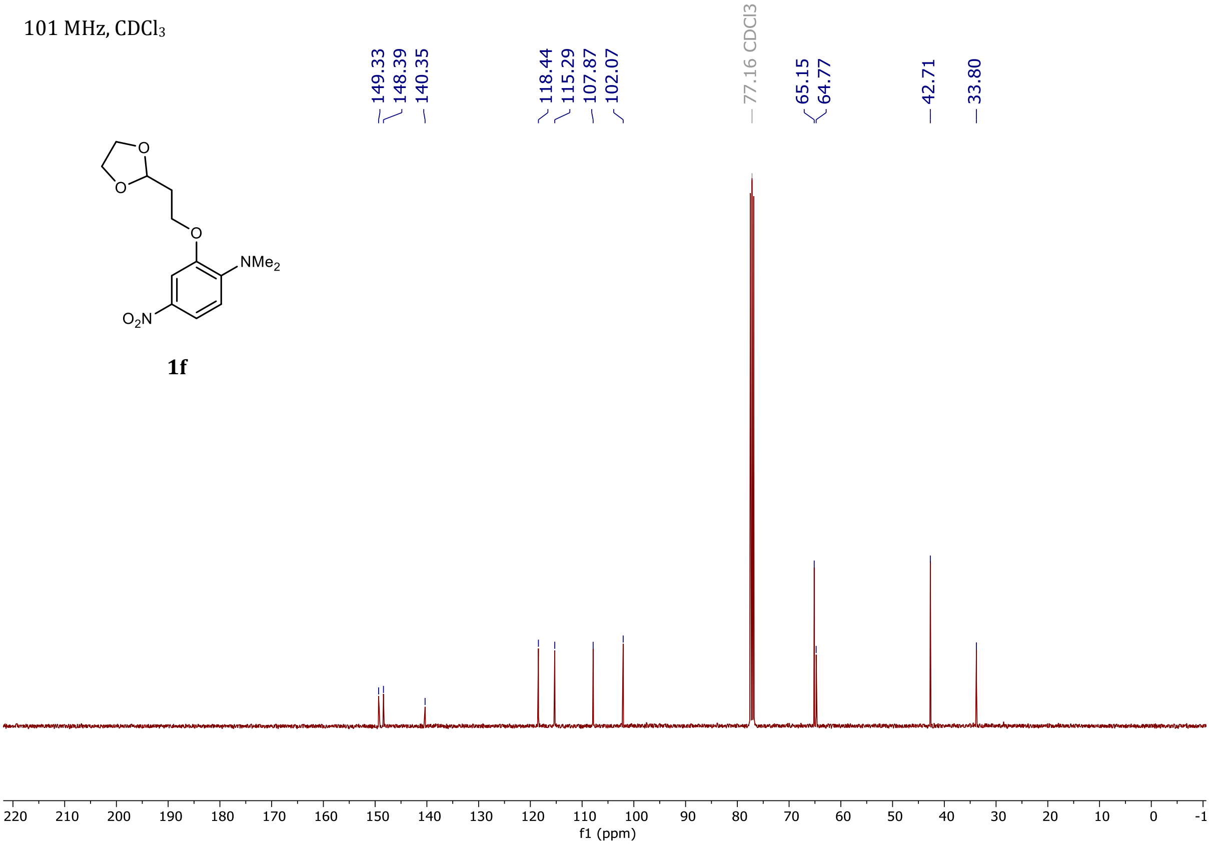
101 MHz, CDCl3
149.33 148.39 140.35 118.44 115.29 107.87 102.07 77.16 CDCl3 65.15 64.77 42.71 33.80
NMe2
O2N
1f
f1 (ppm)

400 MHz, $CDCl_3$

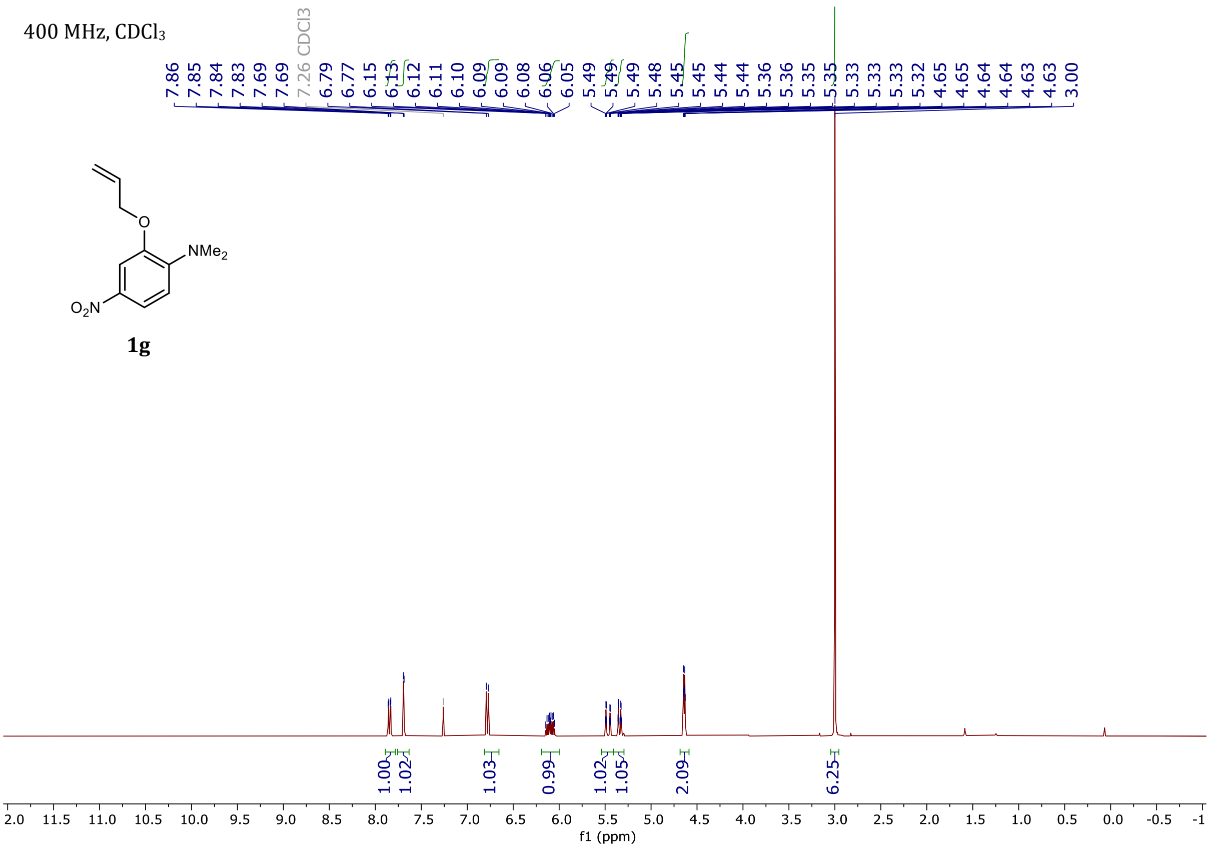

101 MHz, $CDCl_3$

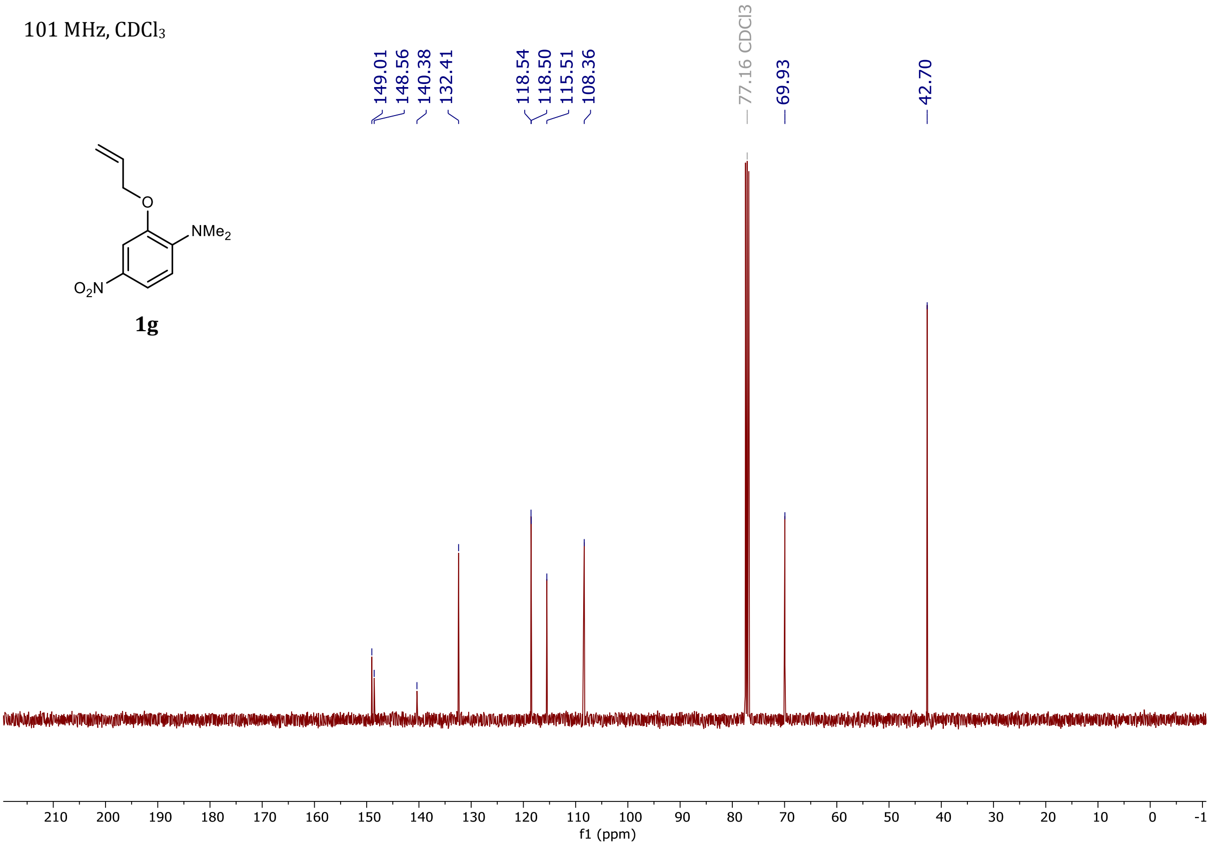

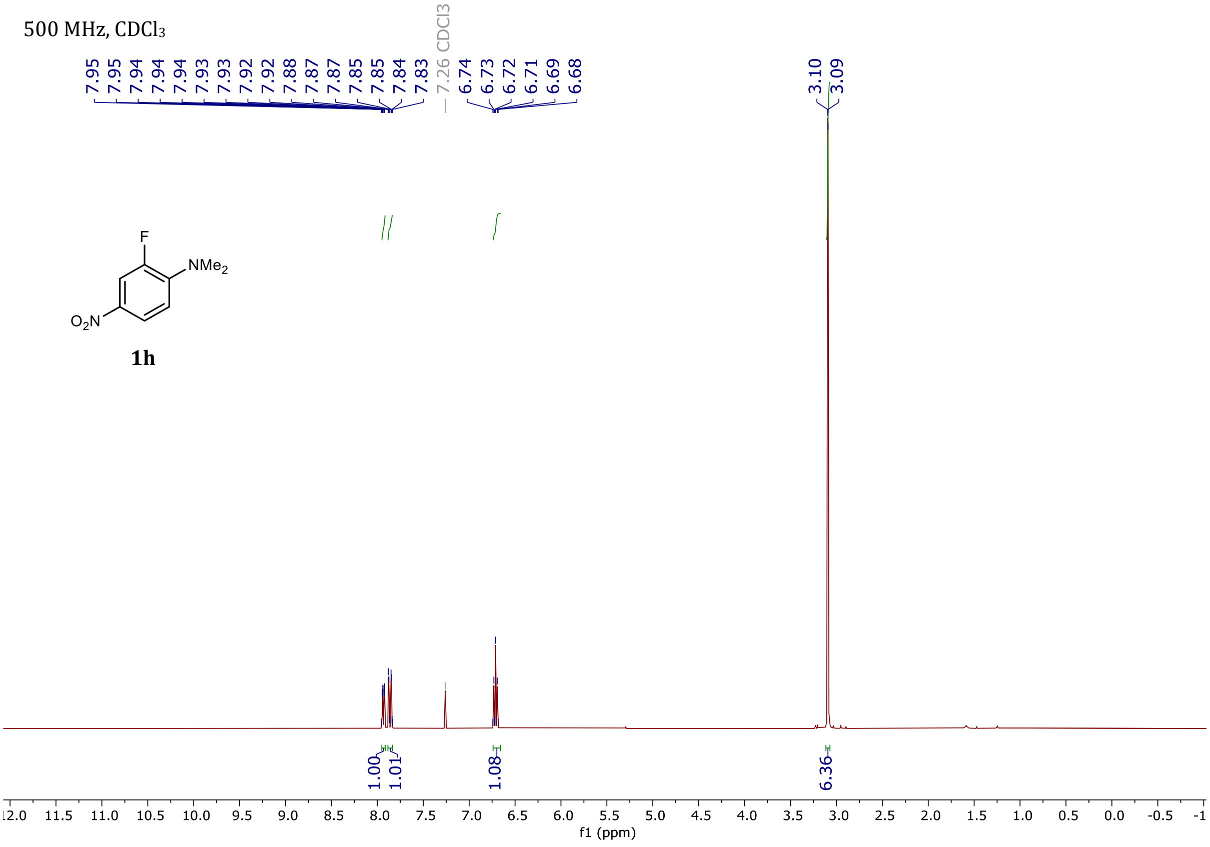
500 MHz, CDCl3
7.95
7.95
7.94
7.94
7.94
7.93
7.93
7.92
7.92
7.88
7.87
7.87
7.85
7.85
7.84
7.83
7.26 CDCl3
6.74
6.73
6.72
6.71
6.69
6.68
3.10
3.09
F
NMe2
O2N
1h
1.00
1.01
1.08
6.36
f1 (ppm)

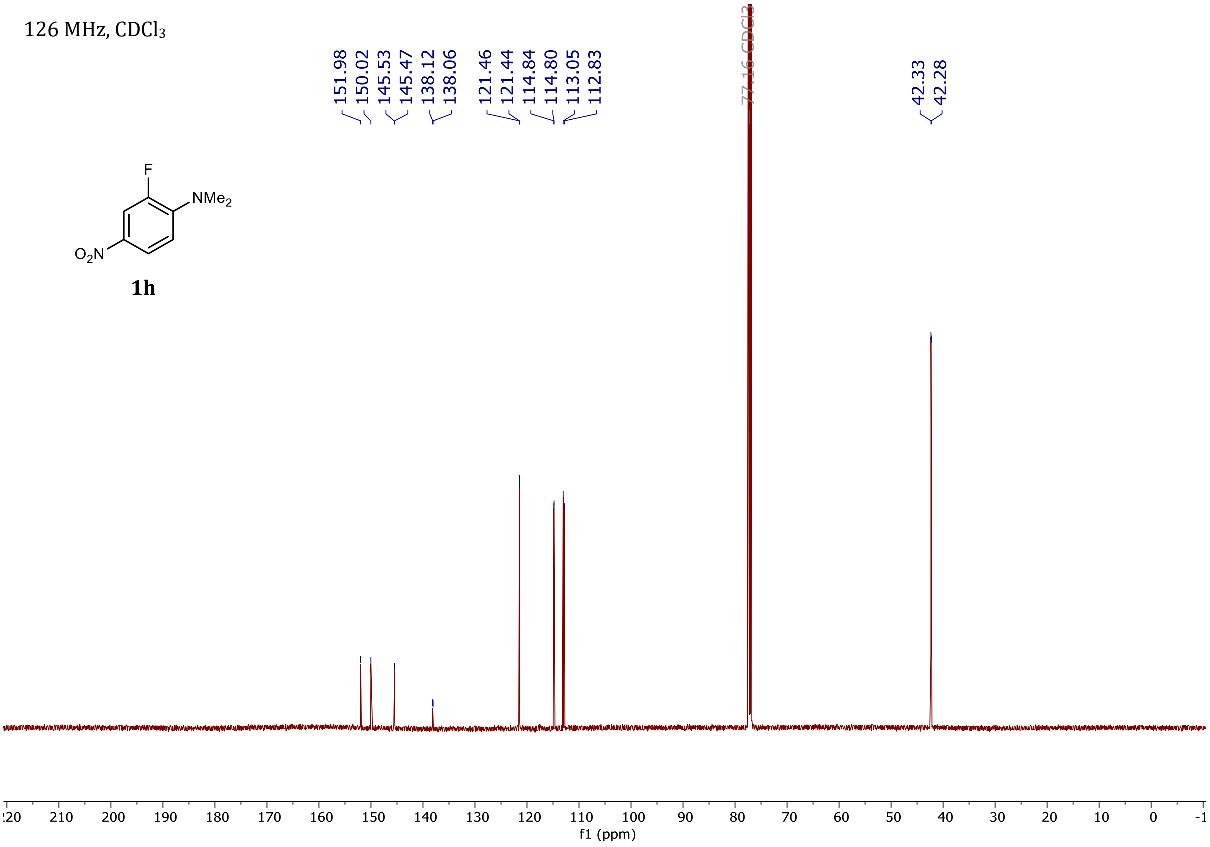
126 MHz, CDCl3
151.98
150.02
145.53
145.47
138.12
138.06
121.46
121.44
114.84
114.80
113.05
112.83
77.16 CDCl3
42.33
42.28
F
NMe2
O2N
1h
f1 (ppm)

471 MHz, $CDCl_3$

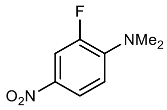

1h

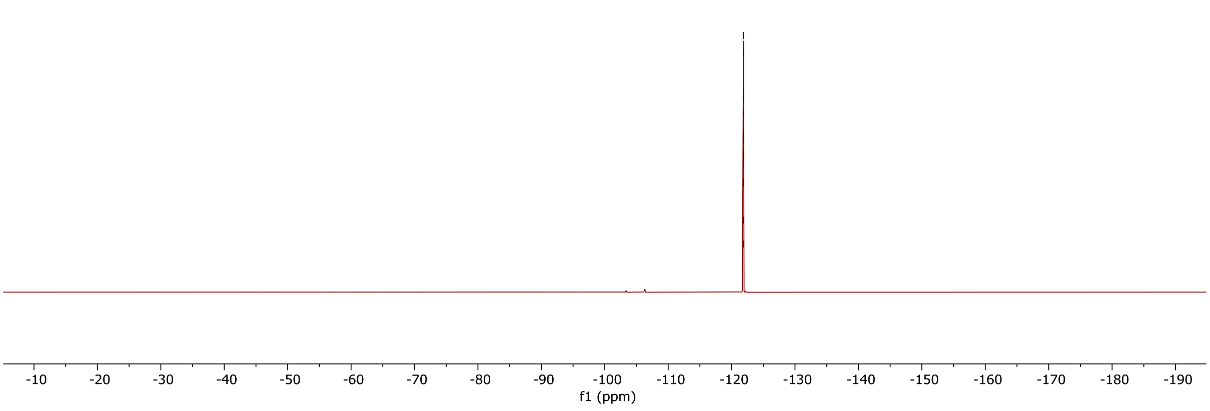

400 MHz, $CDCl_3$

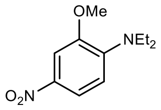

1i

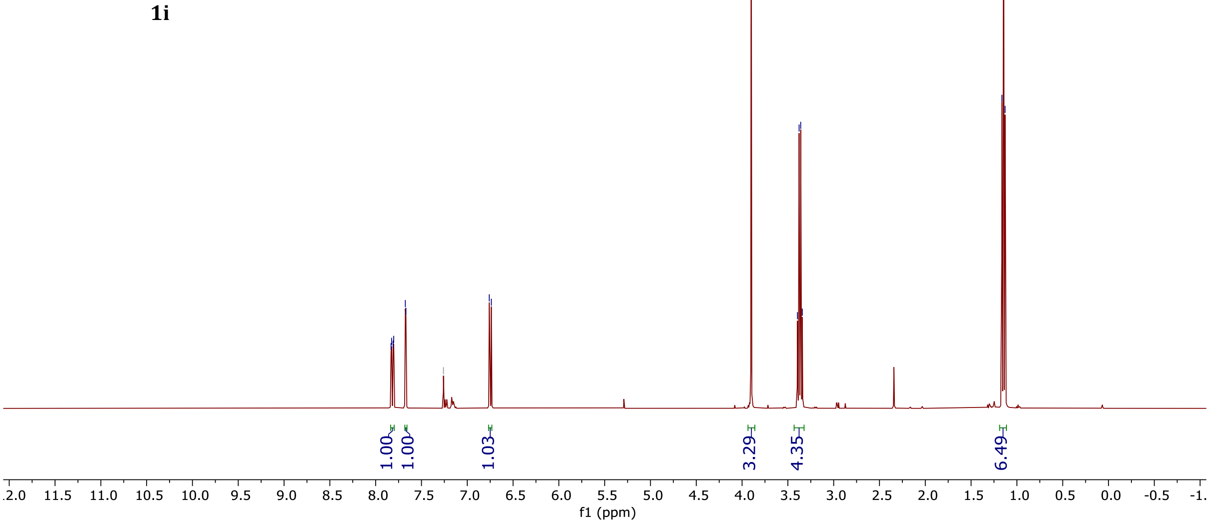

101 MHz, $CDCl_3$

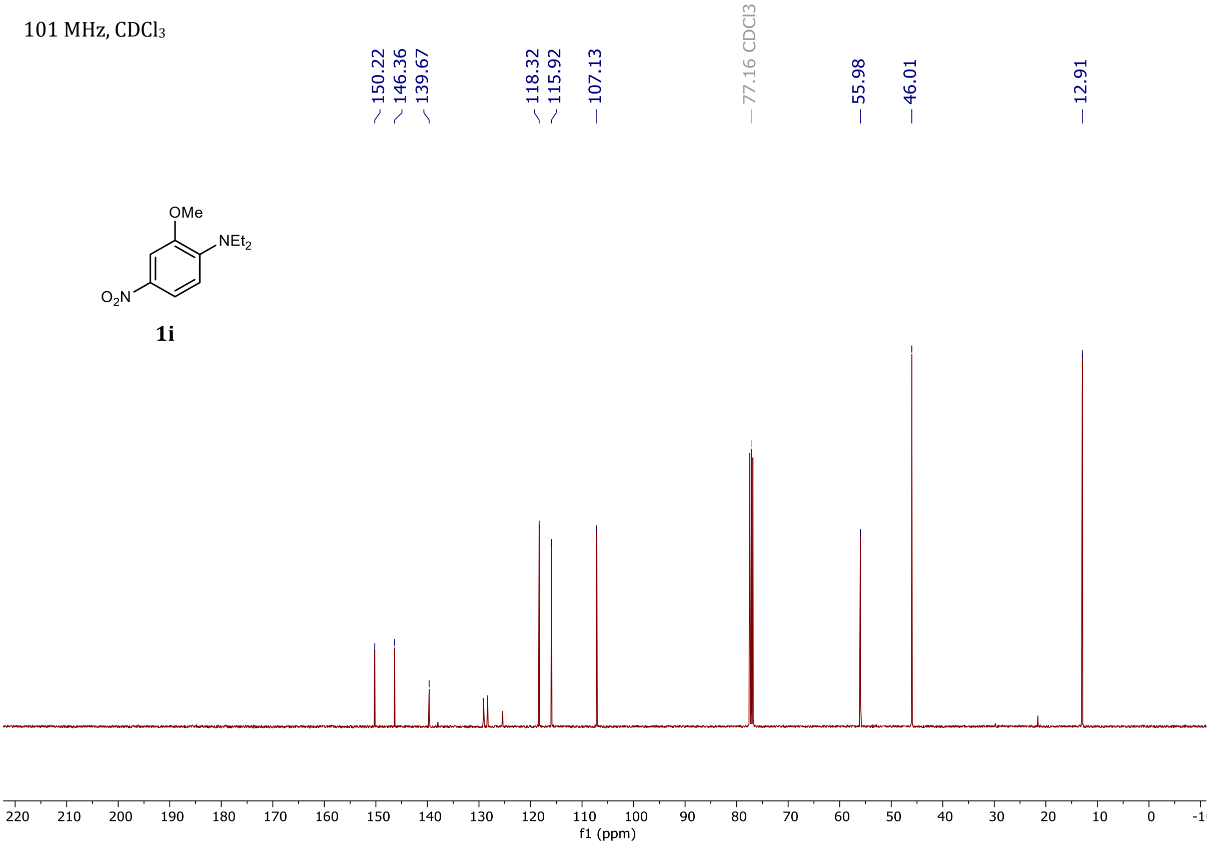

1i

500 MHz, $CDCl_3$

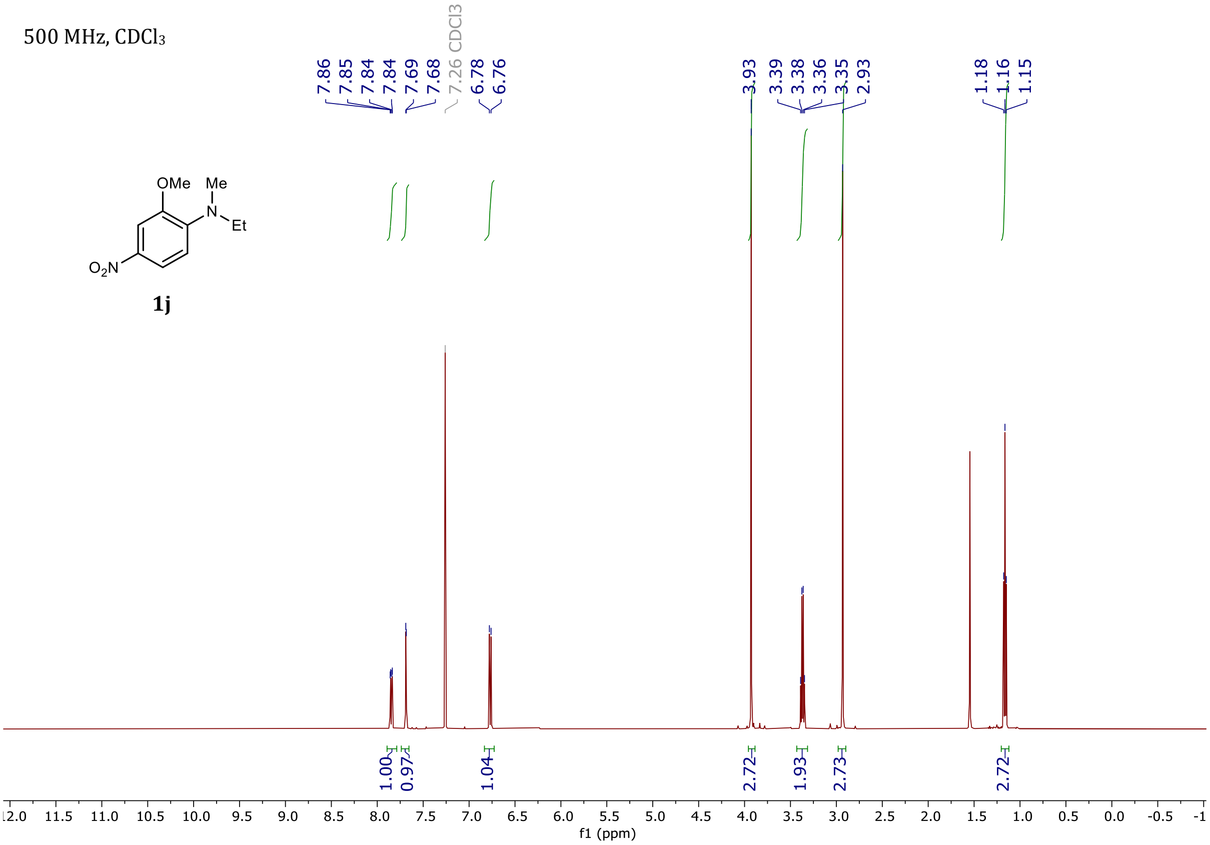

1j

126 MHz, $CDCl_3$

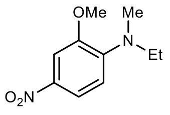

1j

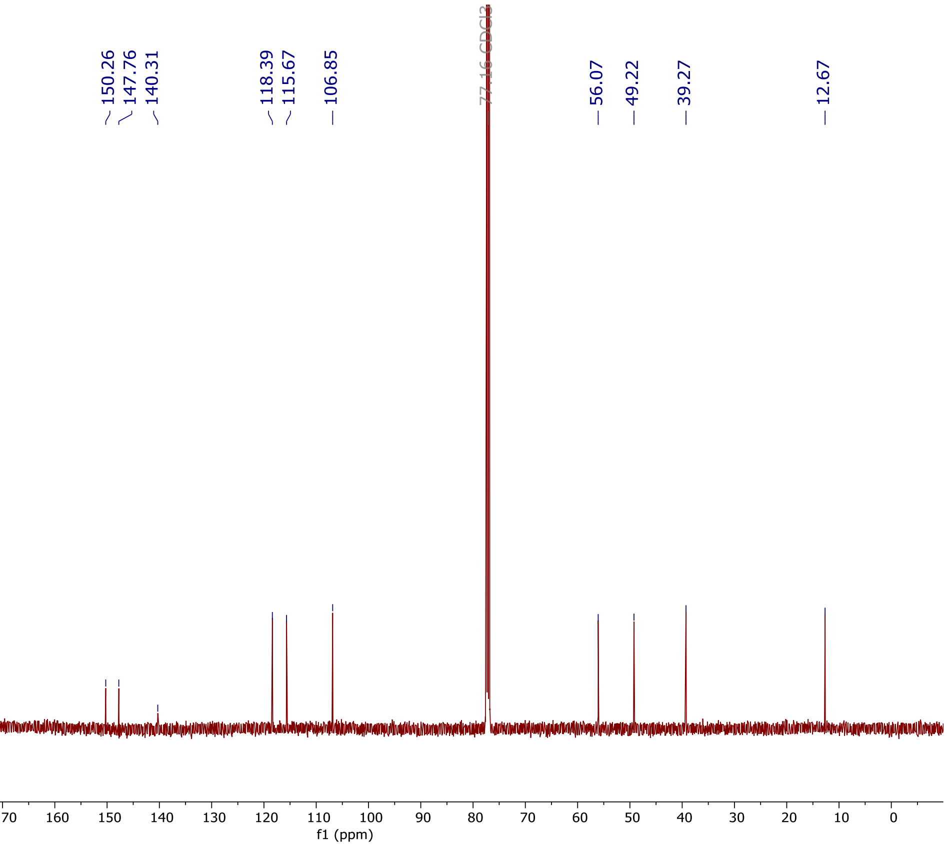

400 MHz, $CDCl_3$

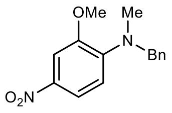

1k

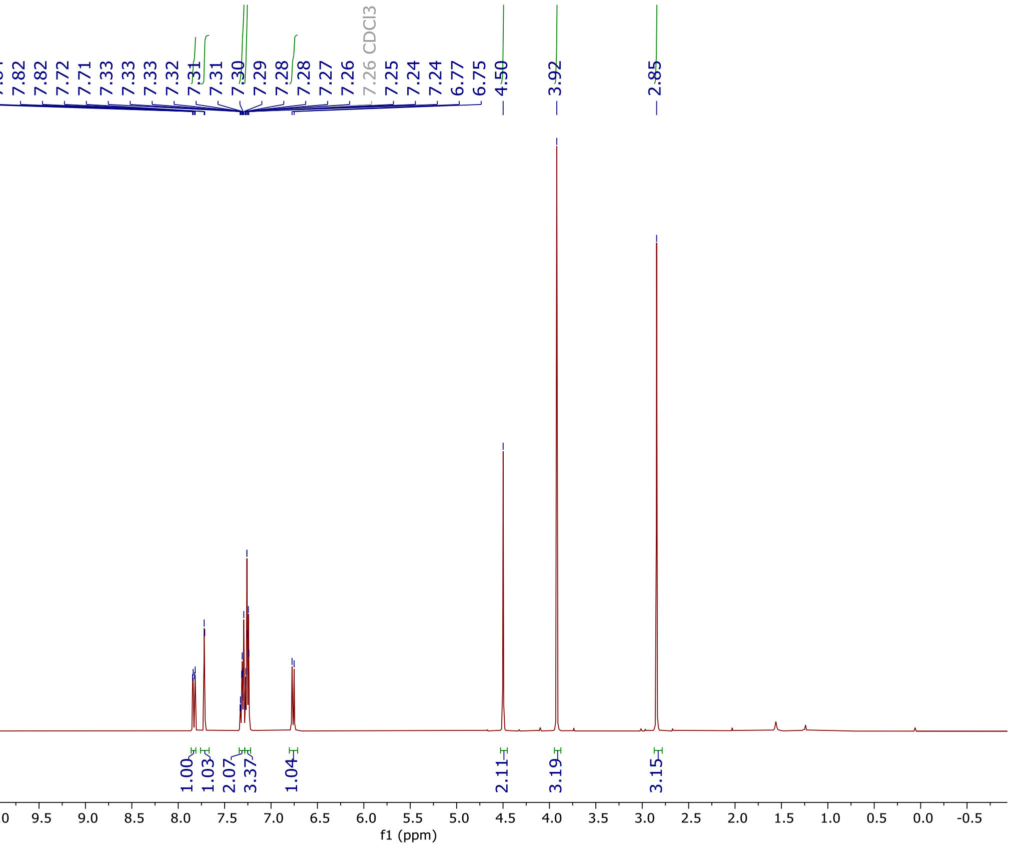

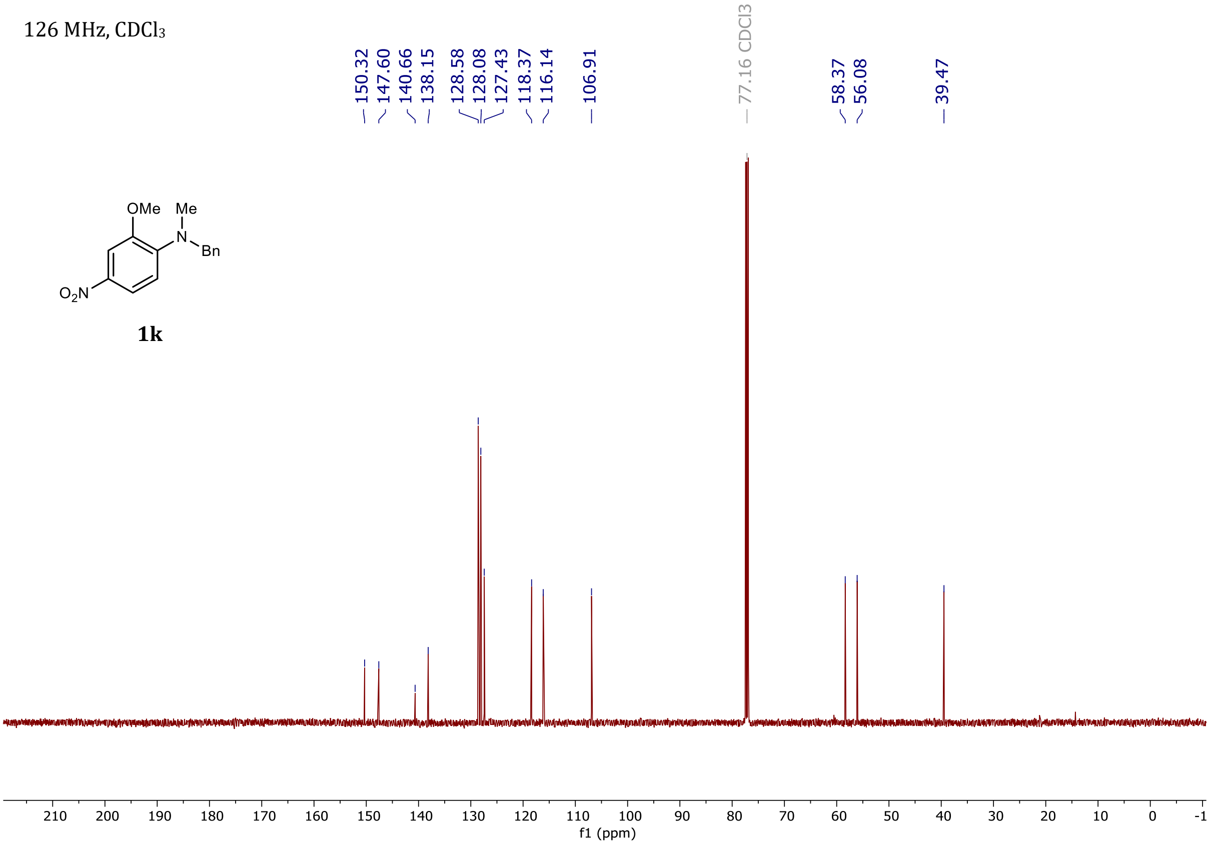
126 MHz, CDCl3
150.32
147.60
140.66
138.15
128.58
128.08
127.43
118.37
116.14
106.91
77.16 CDCl3
58.37
56.08
39.47
OMe
Me
N
Bn
O2N
1k
210 200 190 180 170 160 150 140 130 120 110 100 90 80 70 60 50 40 30 20 10 0 -1
f1 (ppm)

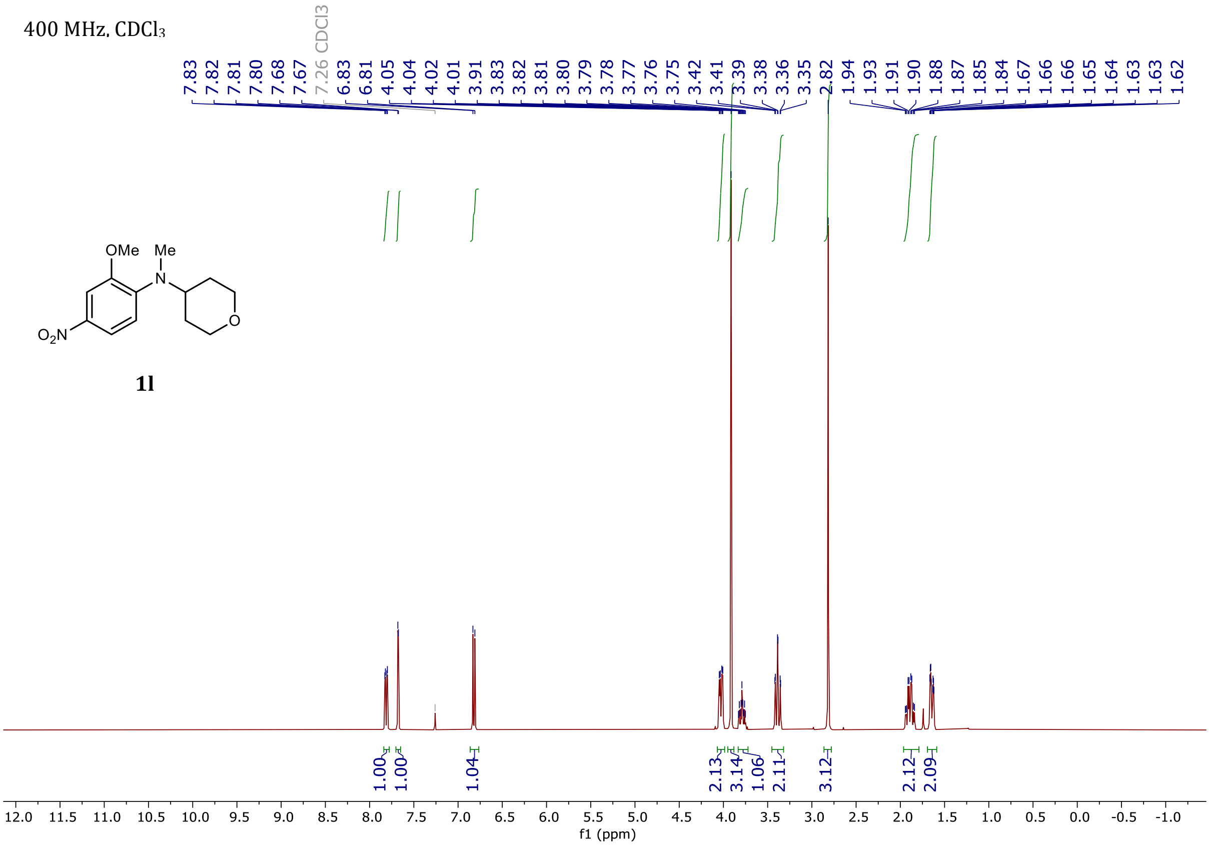
400 MHz, CDCl3
7.83
7.82
7.81
7.80
7.68
7.67
7.26 CDCl3
6.83
6.81
4.05
4.04
4.02
4.01
3.91
3.83
3.82
3.81
3.80
3.79
3.78
3.77
3.76
3.75
3.42
3.41
3.39
3.38
3.36
3.35
2.82
1.94
1.93
1.91
1.90
1.88
1.87
1.85
1.84
1.67
1.66
1.66
1.65
1.64
1.63
1.63
1.62
OMe
Me
N
O
O2N
1l
1.00
1.00
1.04
2.13
3.14
1.06
2.11
3.12
2.12
2.09
12.0 11.5 11.0 10.5 10.0 9.5 9.0 8.5 8.0 7.5 7.0 6.5 6.0 5.5 5.0 4.5 4.0 3.5 3.0 2.5 2.0 1.5 1.0 0.5 0.0 -0.5 -1.0
f1 (ppm)

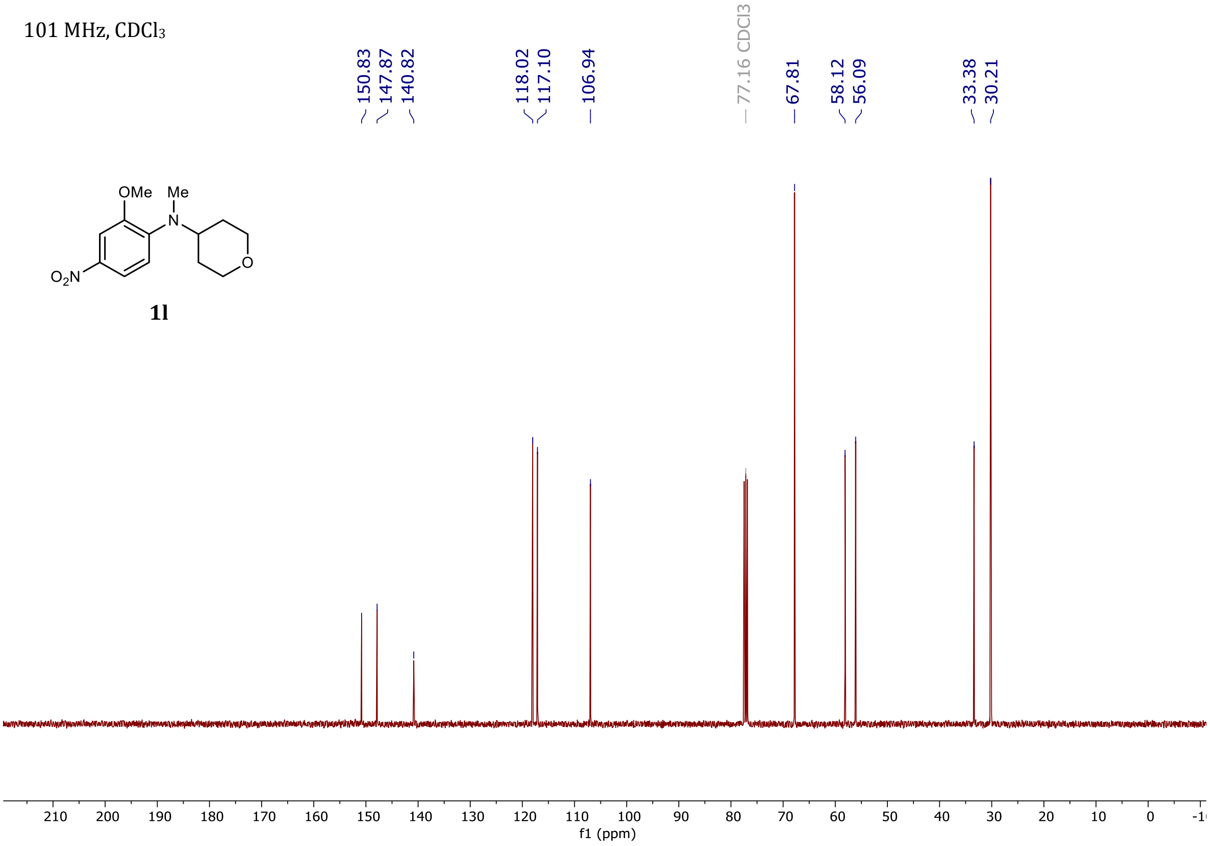
101 MHz, CDCl$_3$
150.83
147.87
140.82
118.02
117.10
106.94
77.16 CDCl3
67.81
58.12
56.09
33.38
30.21
OMe
Me
N
O
O_2N
1l
f1 (ppm)

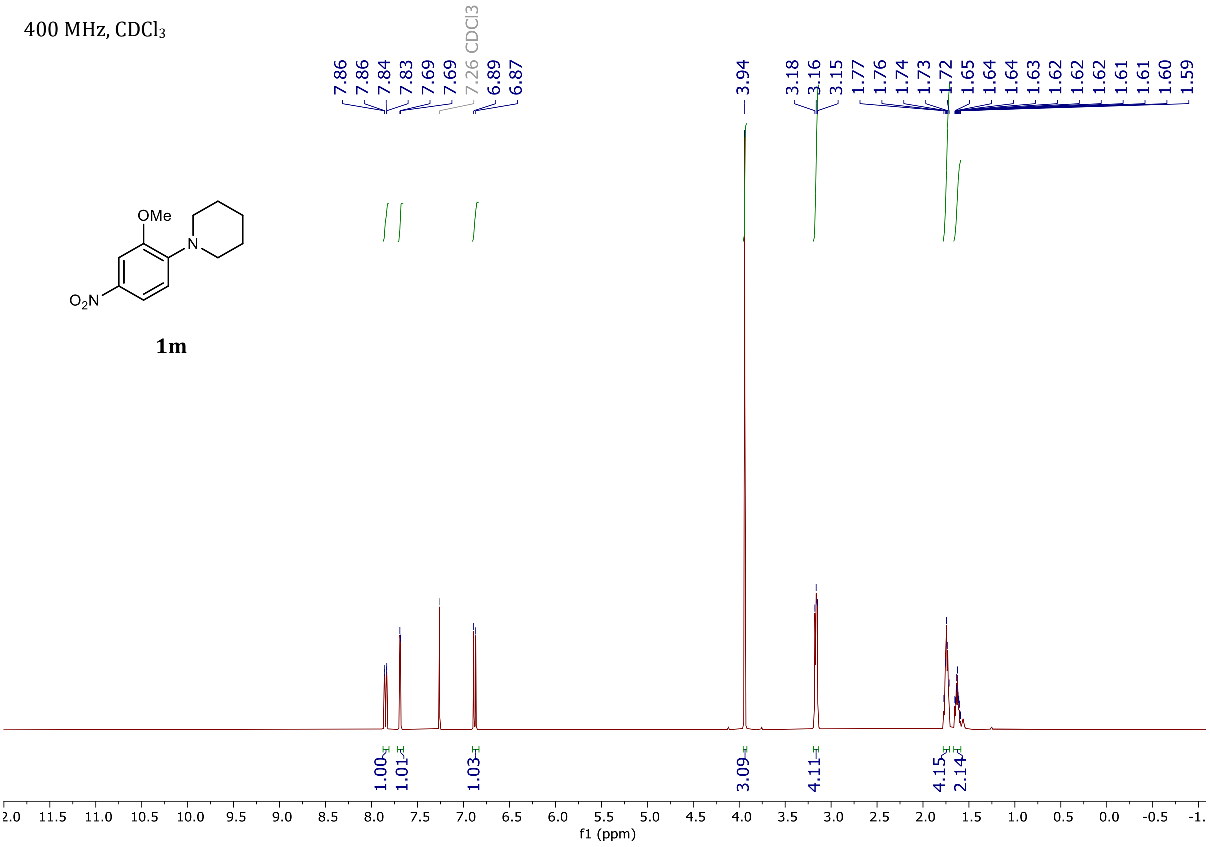
400 MHz, CDCl$_3$
7.86
7.86
7.84
7.83
7.69
7.69
7.26 CDCl3
6.89
6.87
3.94
3.18
3.16
3.15
1.77
1.76
1.74
1.73
1.72
1.65
1.64
1.64
1.63
1.62
1.62
1.62
1.61
1.61
1.60
1.59
OMe
N
O_2N
1m
1.00
1.01
1.03
3.09
4.11
4.15
2.14
f1 (ppm)

101 MHz, CDCl$_3$

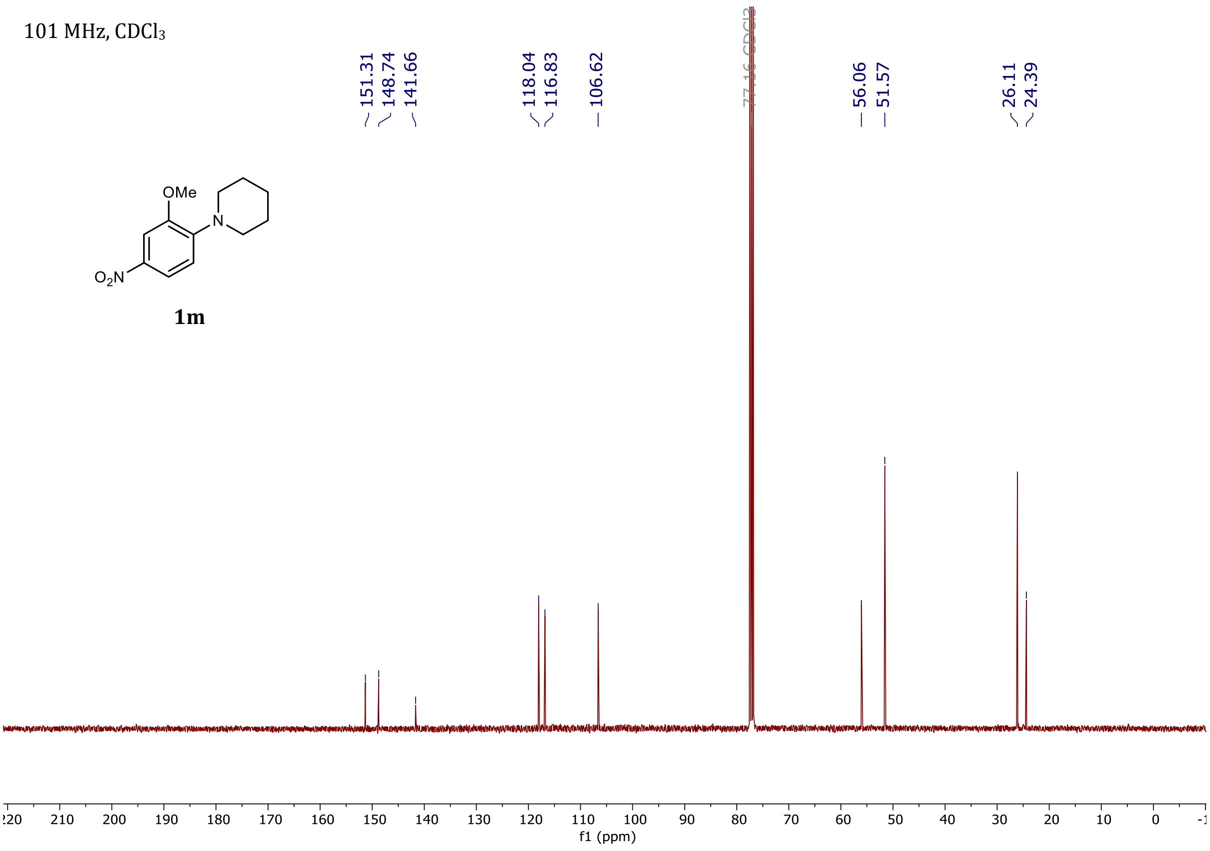

400 MHz, CDCl$_3$

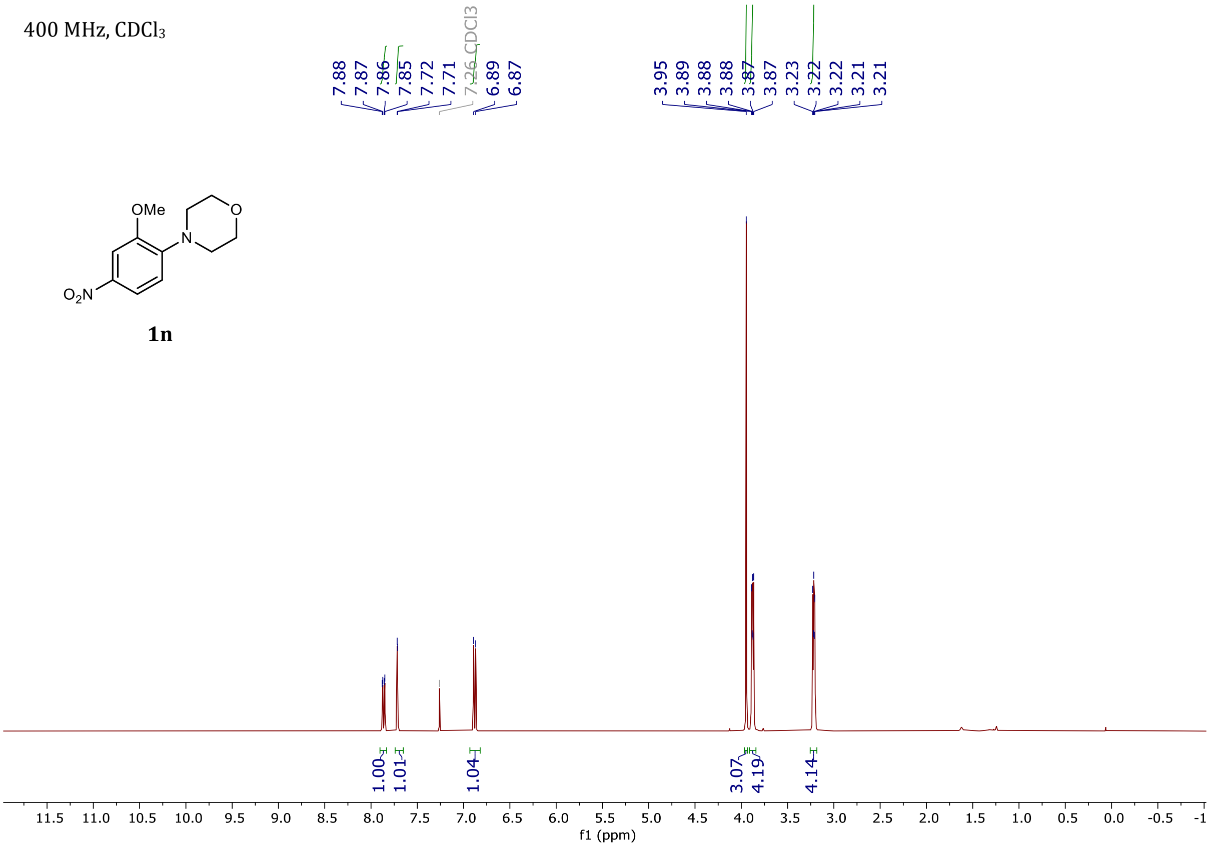

101 MHz, $CDCl_3$

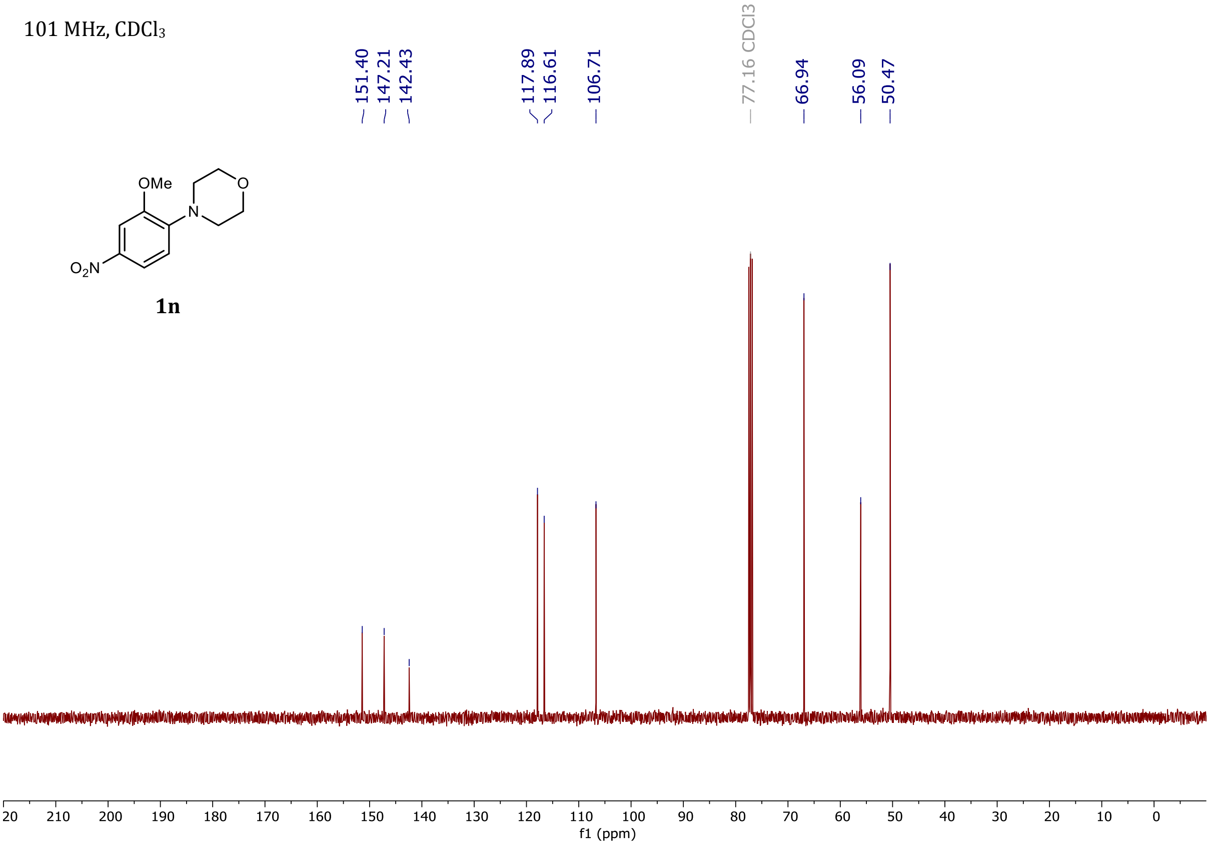

500 MHz, $CDCl_3$

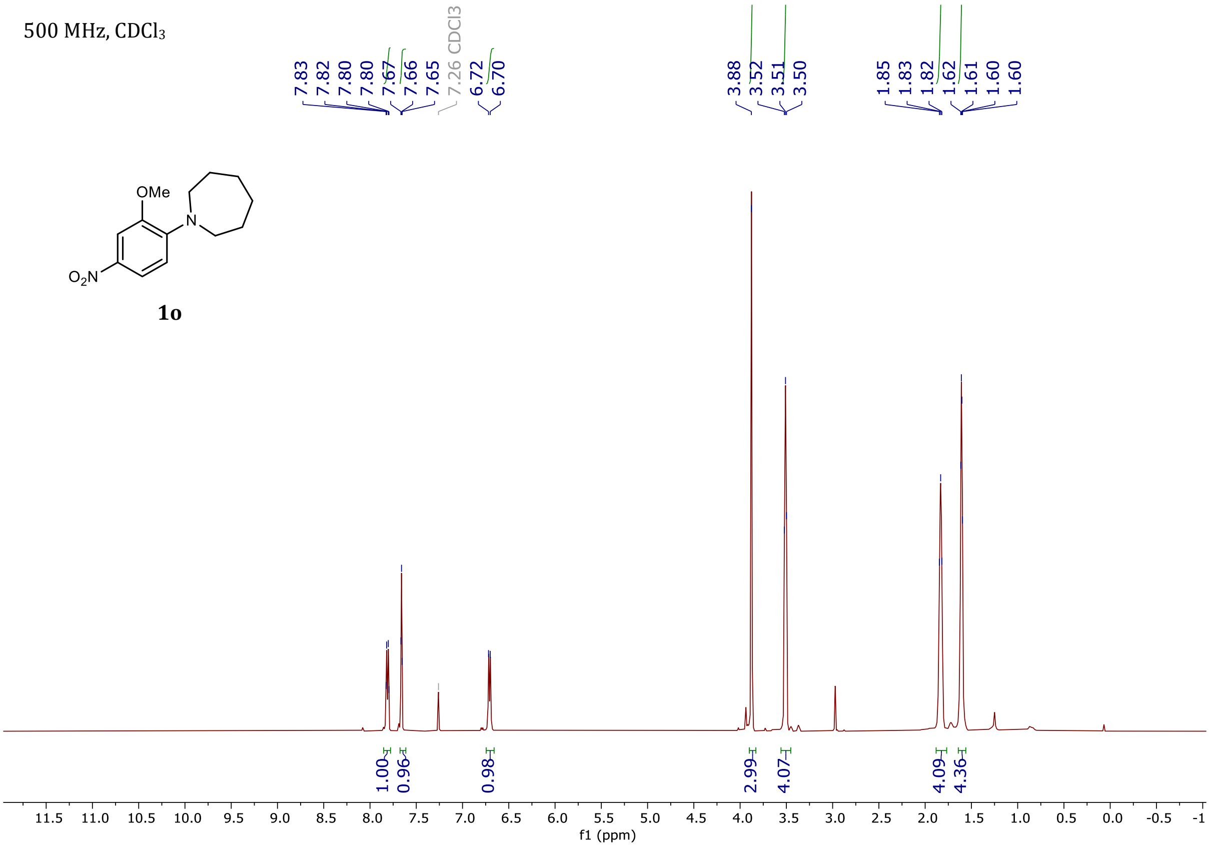

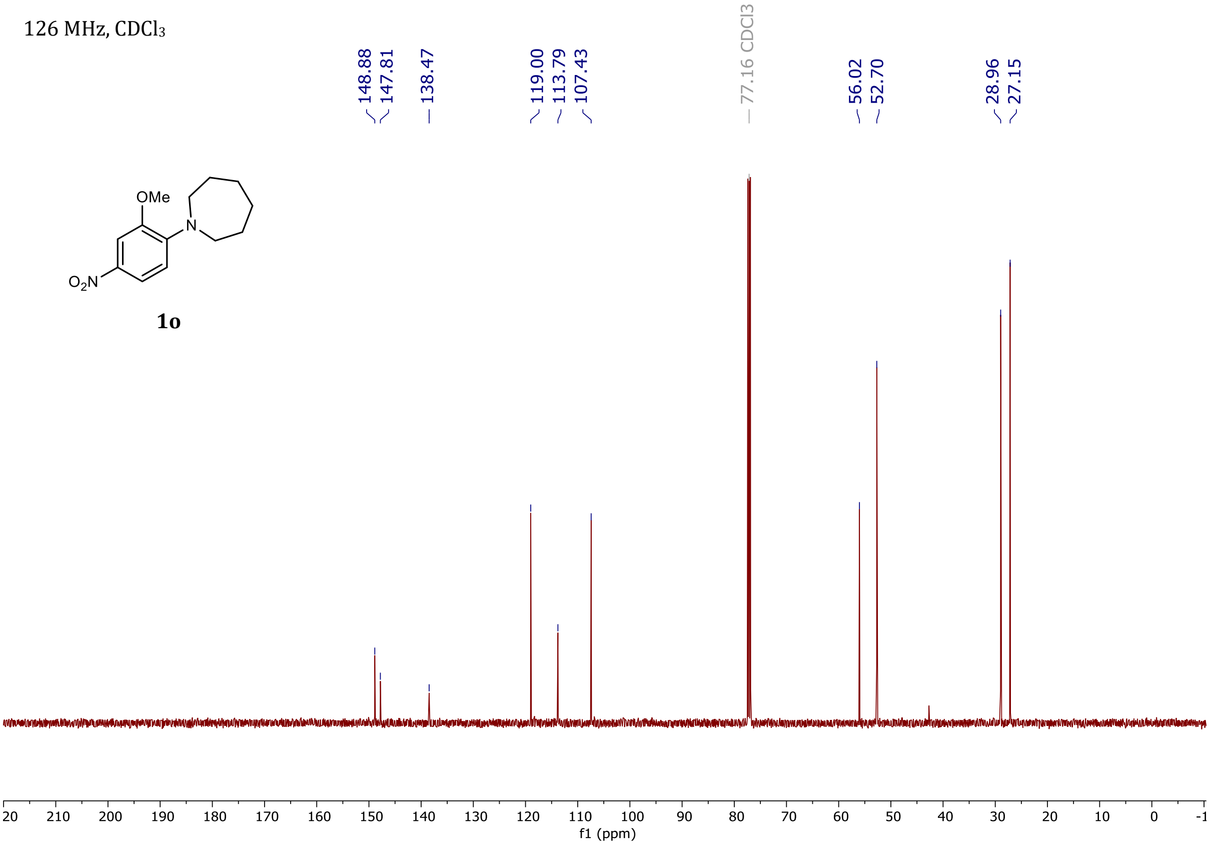
126 MHz, CDCl3
148.88
147.81
138.47
119.00
113.79
107.43
77.16 CDCl3
56.02
52.70
28.96
27.15
OMe
N
O2N
1o
f1 (ppm)

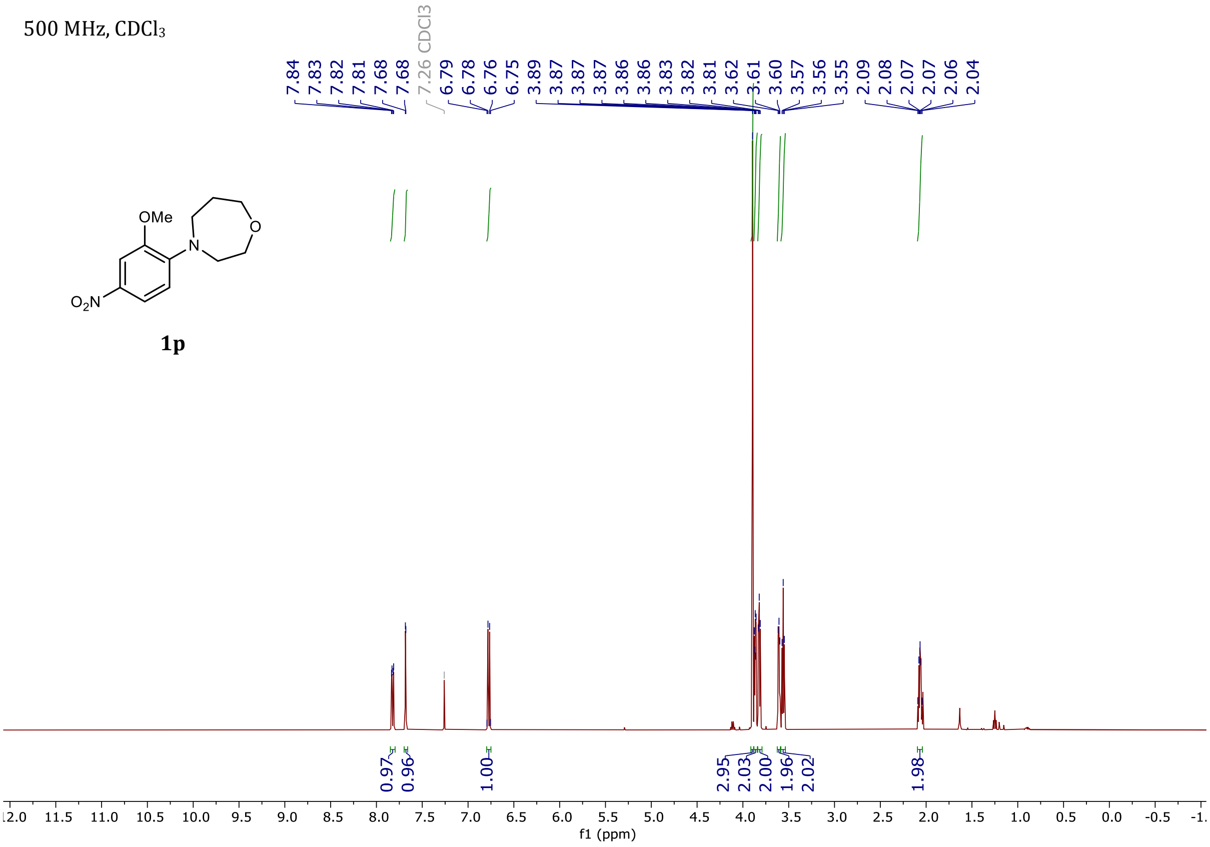
500 MHz, CDCl3
7.84
7.83
7.82
7.81
7.68
7.68
7.26 CDCl3
6.79
6.78
6.76
6.75
3.89
3.87
3.87
3.86
3.86
3.83
3.82
3.81
3.62
3.61
3.60
3.57
3.56
3.55
2.09
2.08
2.07
2.07
2.06
2.04
OMe
N
O
O2N
1p
0.97
0.96
1.00
2.95
2.03
2.00
1.96
2.02
1.98
f1 (ppm)

126 MHz, CDCl₃

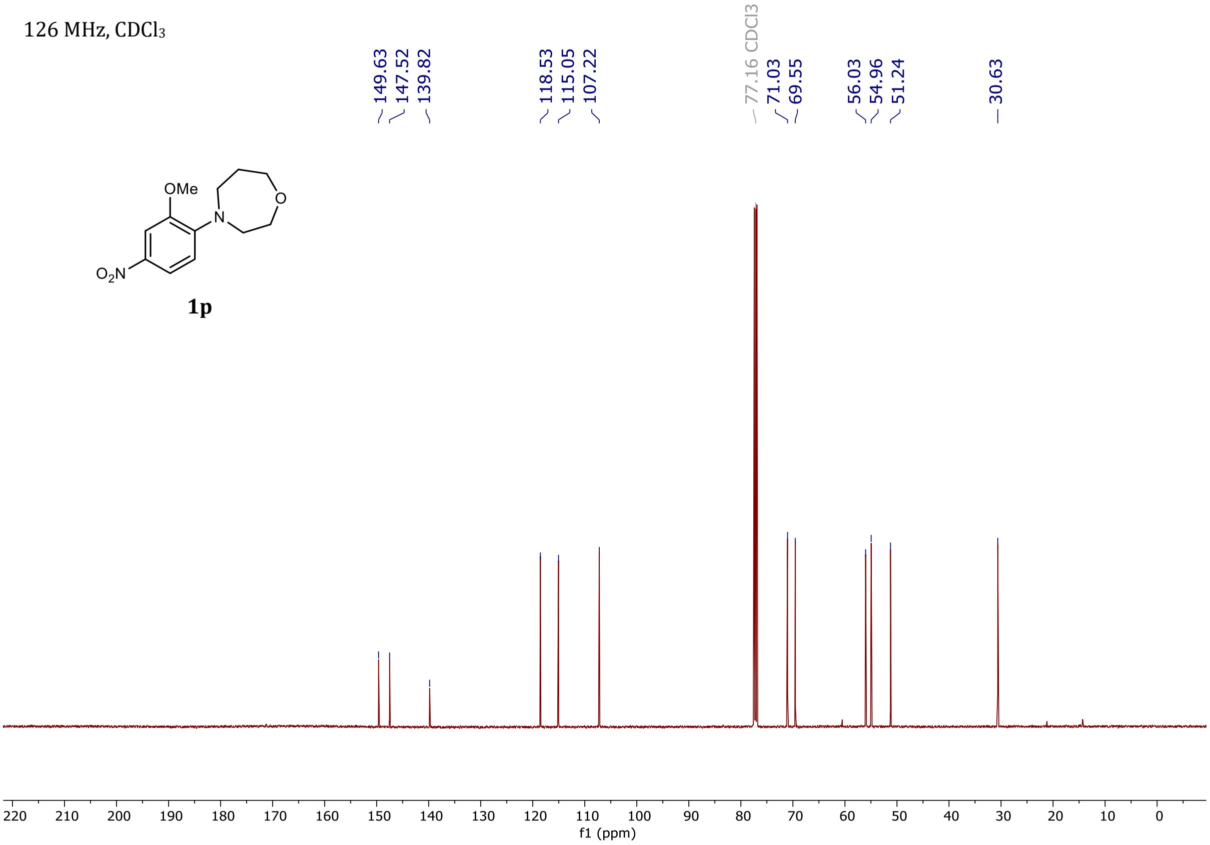

1p

400 MHz, CDCl₃

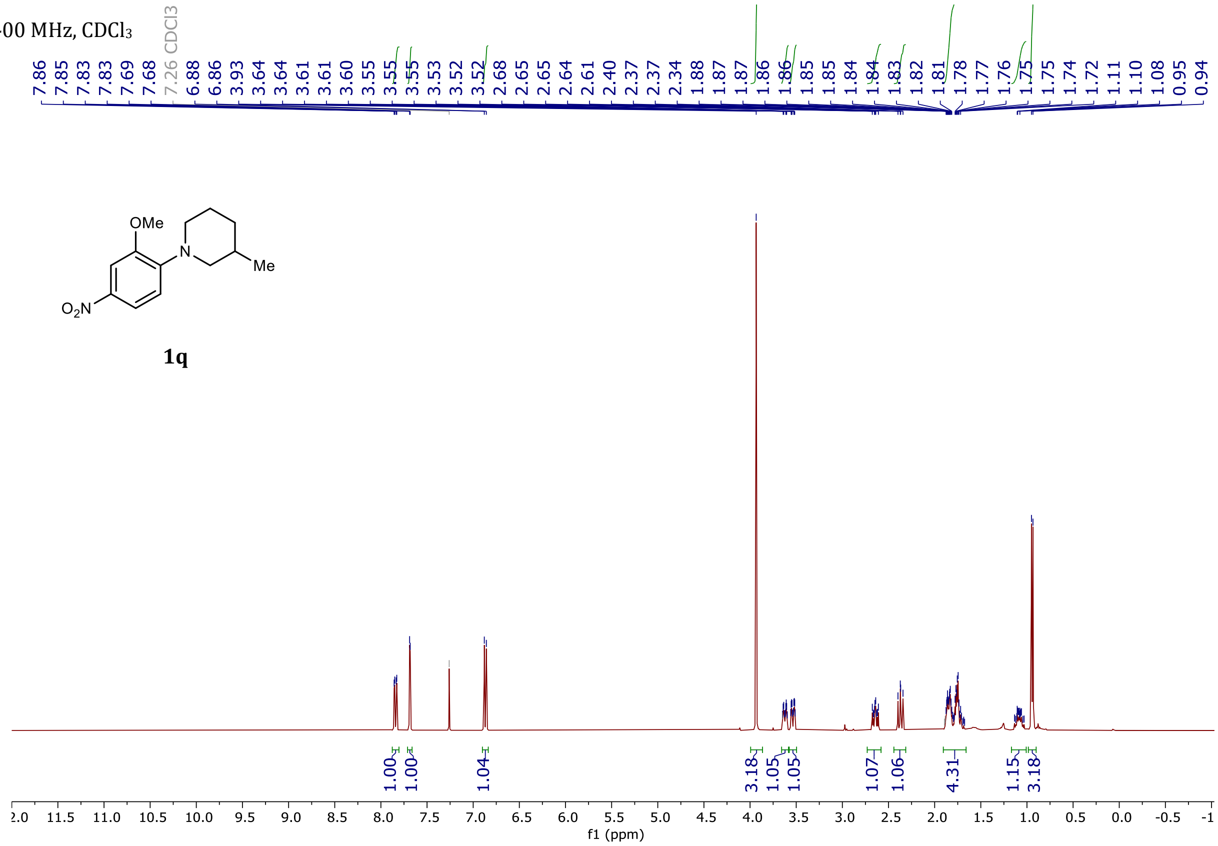

1q

101 MHz, CDCl3

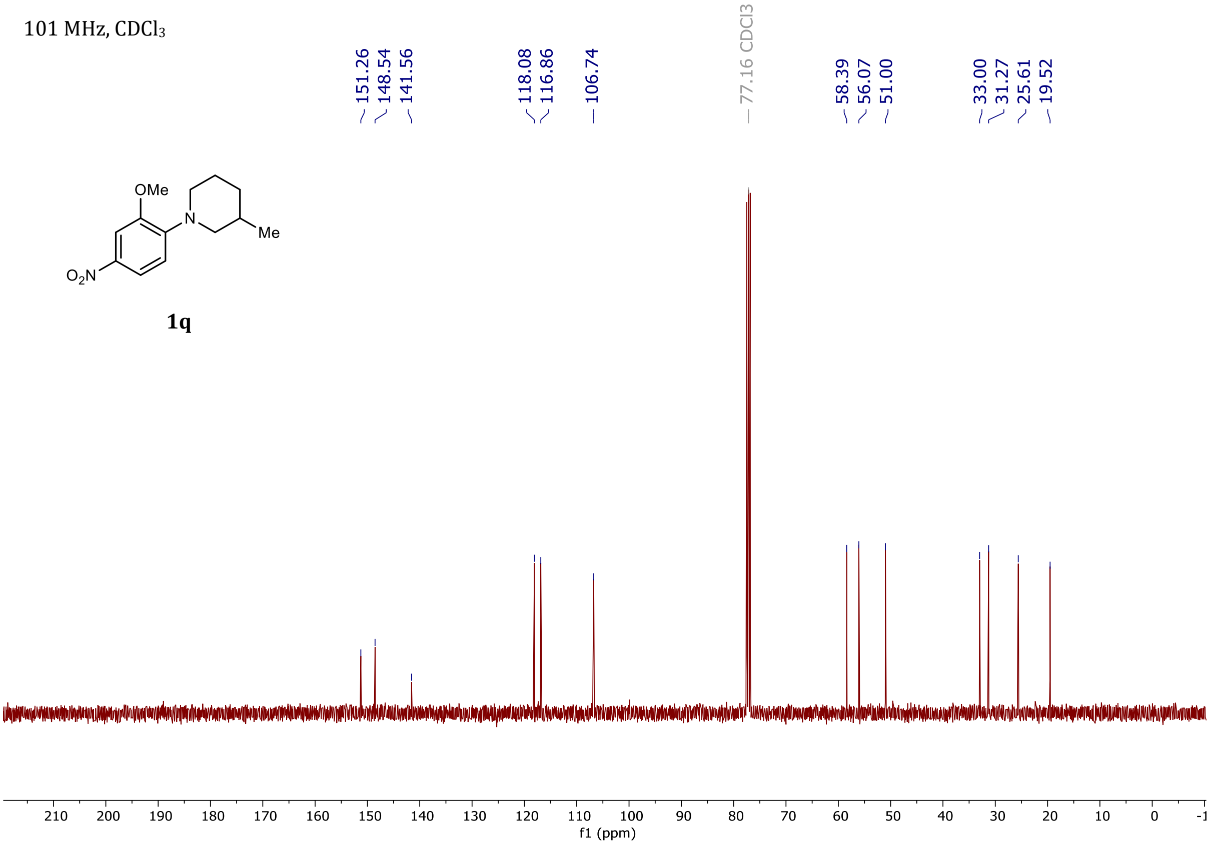

1q

400 MHz, CDCl3

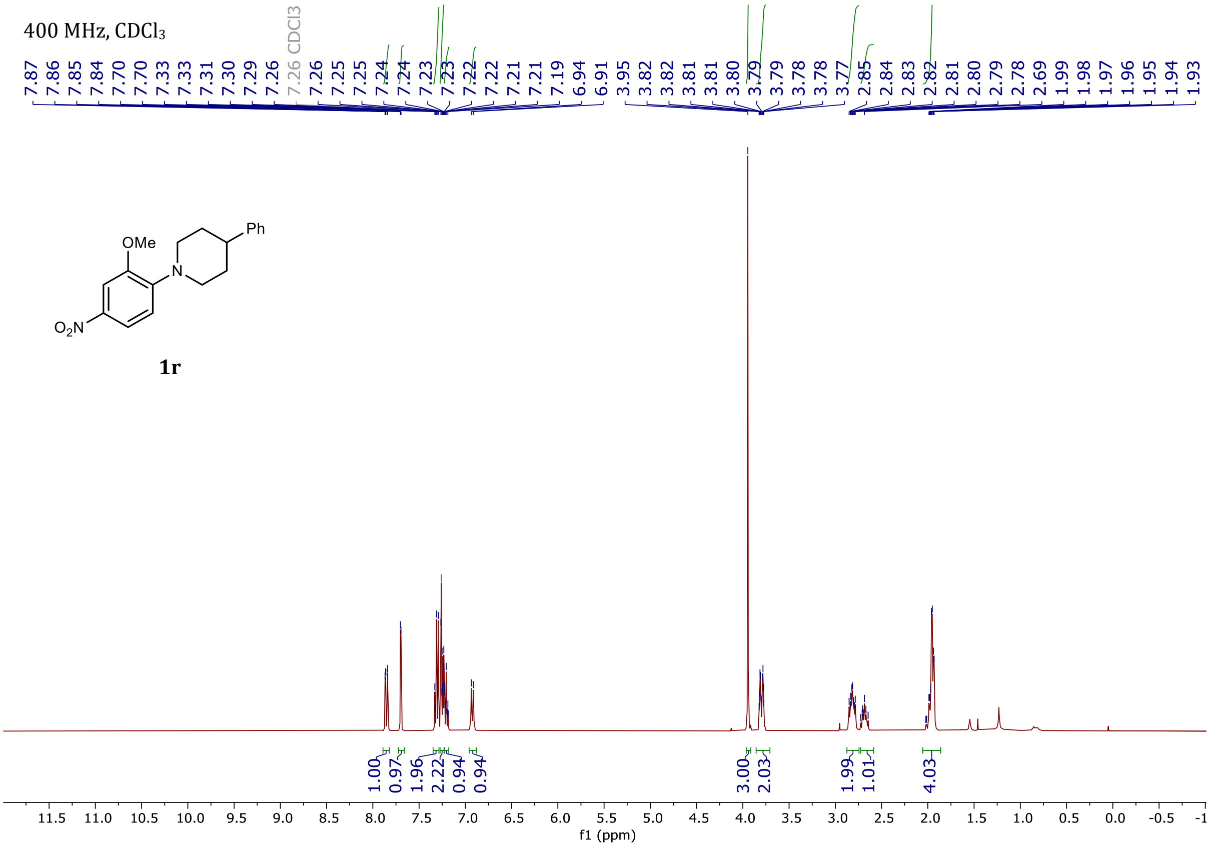

1r

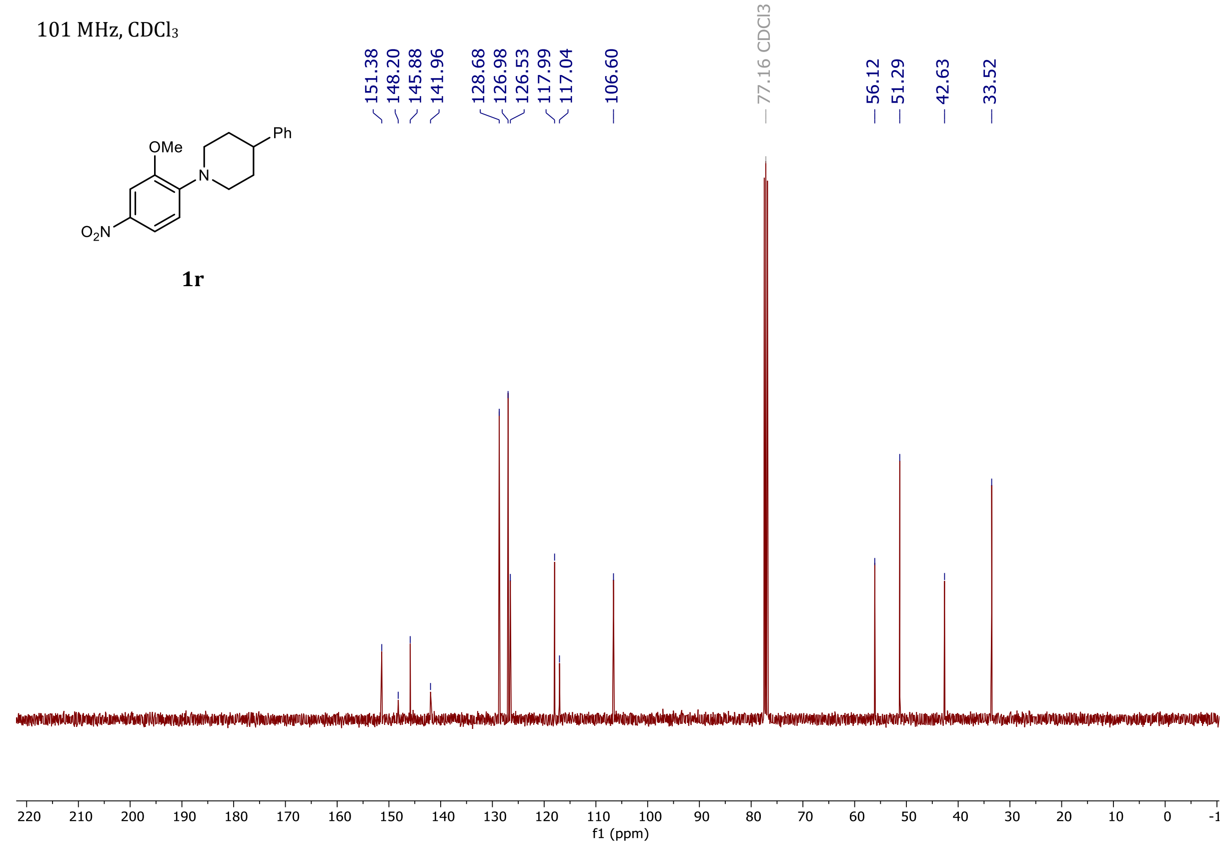
101 MHz, CDCl3
151.38
148.20
145.88
141.96
128.68
126.98
126.53
117.99
117.04
106.60
77.16 CDCl3
56.12
51.29
42.63
33.52
OMe
Ph
N
O2N
1r
f1 (ppm)

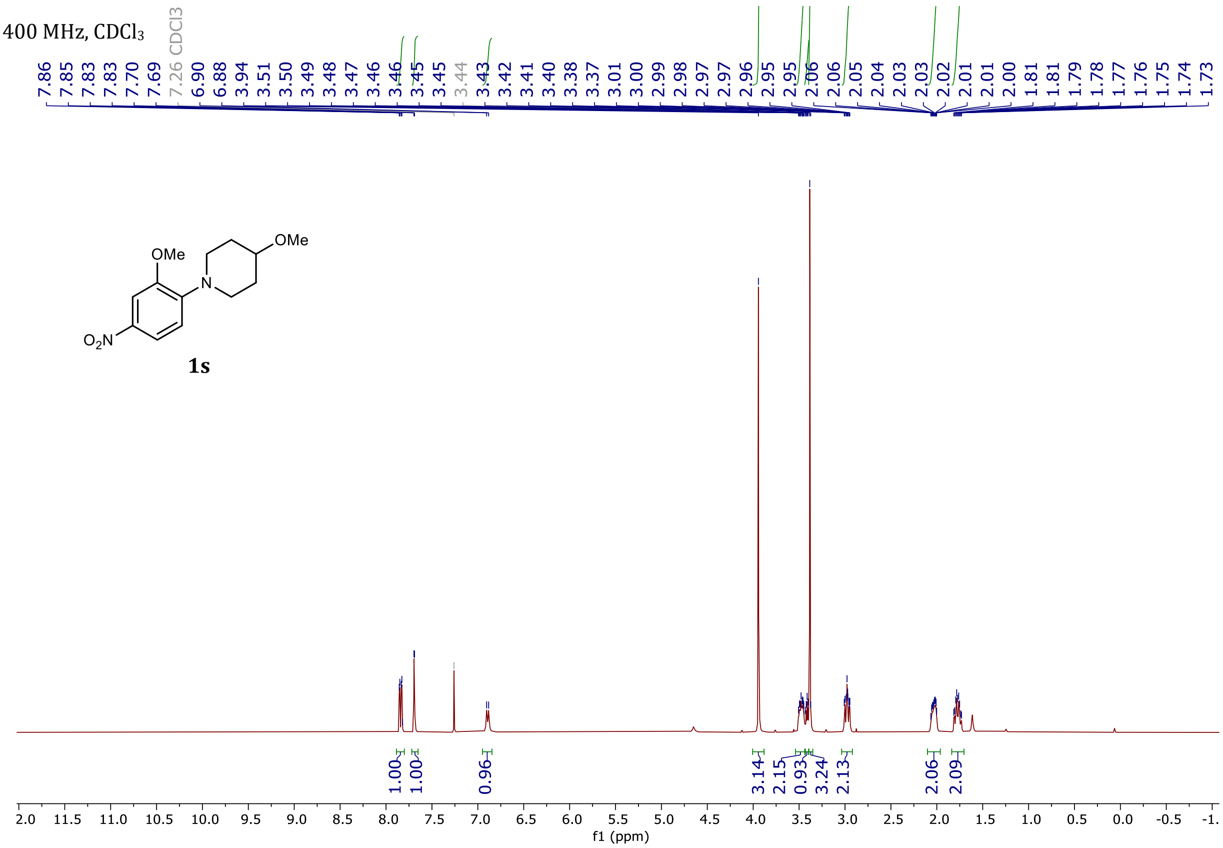
400 MHz, CDCl3
7.86 7.85 7.83 7.83 7.70 7.69 7.26 CDCl3 6.90 6.88 3.94 3.51 3.50 3.49 3.48 3.47 3.46 3.45 3.45 3.44 3.43 3.42 3.41 3.40 3.38 3.37 3.01 3.00 2.99 2.98 2.97 2.97 2.96 2.95 2.95 2.06 2.05 2.04 2.03 2.02 2.02 2.01 2.00 1.81 1.81 1.79 1.78 1.77 1.76 1.75 1.74 1.73
OMe
OMe
N
O2N
1s
1.00 1.00 0.96 3.14 2.15 0.93 3.24 2.13 2.06 2.09
f1 (ppm)

101 MHz, $CDCl_3$

1s

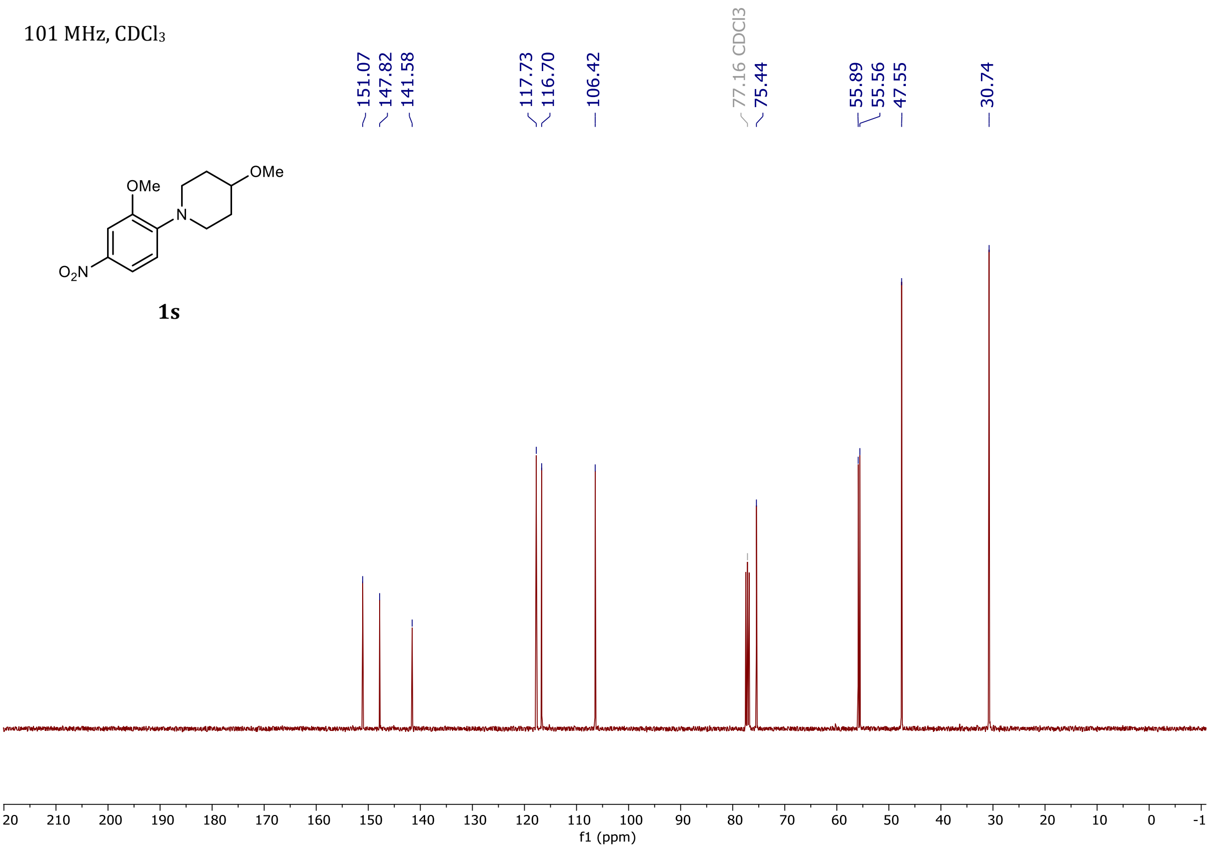

400 MHz, $CDCl_3$

1t

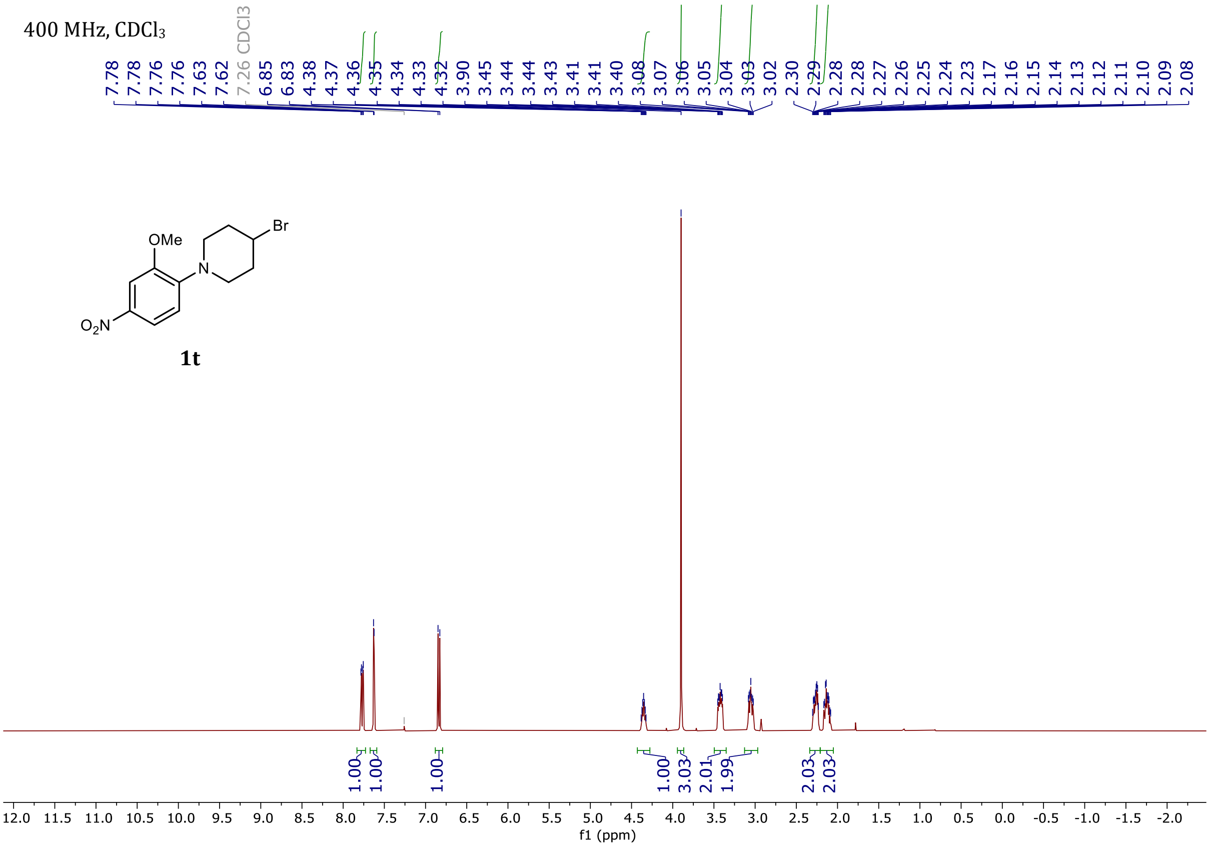

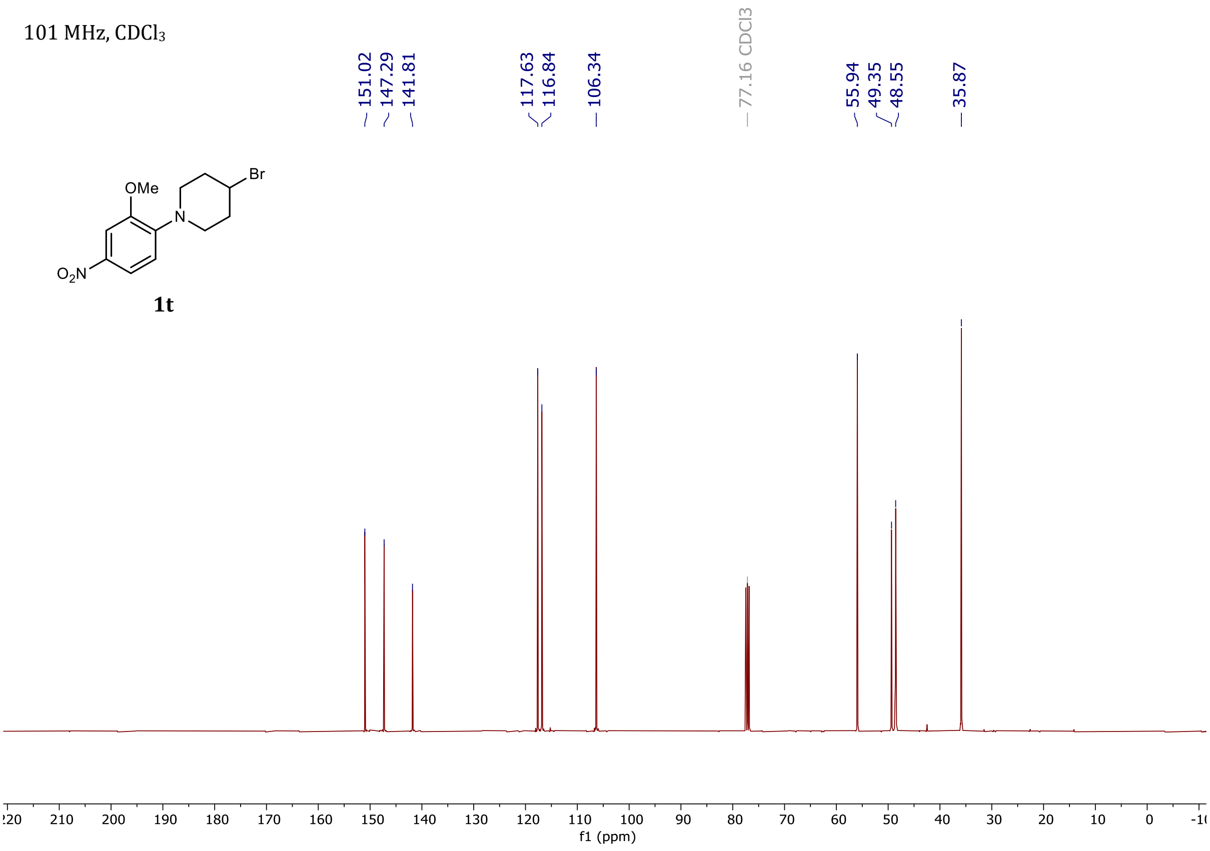
101 MHz, CDCl3
151.02
147.29
141.81
117.63
116.84
106.34
77.16 CDCl3
55.94
49.35
48.55
35.87
OMe
Br
N
O2N
1t
f1 (ppm)

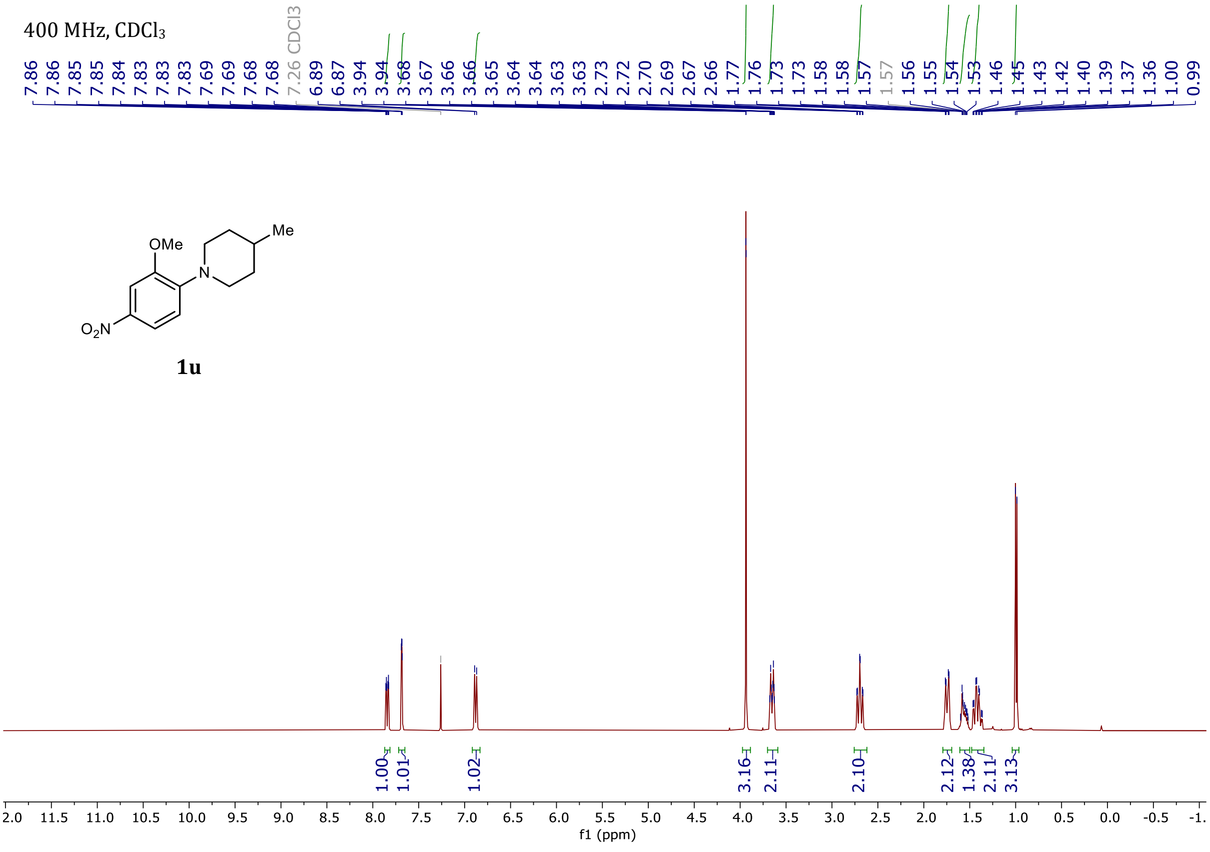
400 MHz, CDCl3
7.86 7.86 7.85 7.85 7.84 7.83 7.83 7.83 7.69 7.69 7.68 7.68 7.26 CDCl3 6.89 6.87 3.94 3.68 3.67 3.66 3.66 3.65 3.64 3.64 3.63 3.63 2.73 2.72 2.70 2.69 2.67 2.66 1.77 1.76 1.73 1.73 1.58 1.58 1.57 1.57 1.56 1.55 1.54 1.53 1.46 1.45 1.43 1.42 1.40 1.39 1.37 1.36 1.00 0.99
OMe
Me
N
O2N
1u
1.00
1.01
1.02
3.16
2.11
2.10
2.12
1.38
2.11
3.13
f1 (ppm)

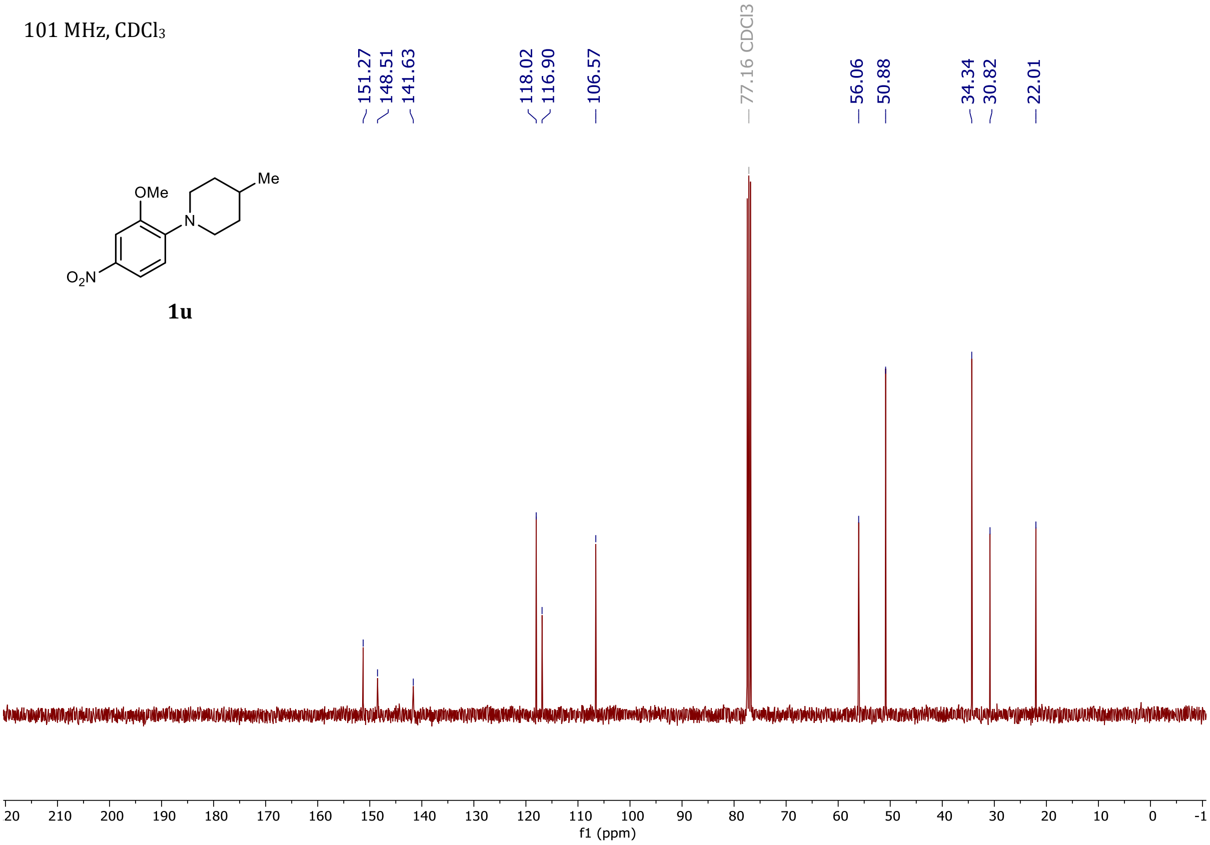
101 MHz, CDCl3
151.27
148.51
141.63
118.02
116.90
106.57
77.16 CDCl3
56.06
50.88
34.34
30.82
22.01
OMe
Me
N
O2N
1u
f1 (ppm)

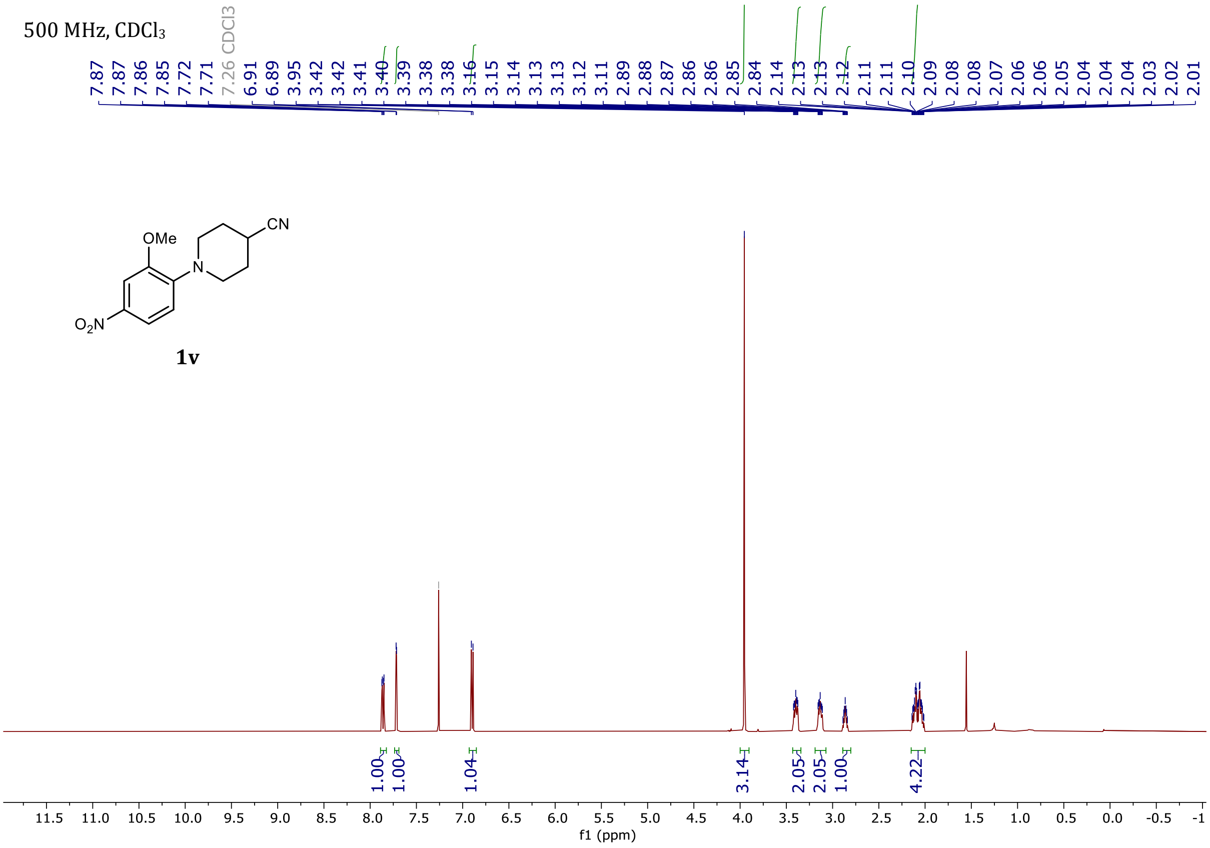
500 MHz, CDCl3
7.87
7.87
7.86
7.85
7.72
7.71
7.26 CDCl3
6.91
6.89
3.95
3.42
3.42
3.41
3.40
3.39
3.38
3.38
3.16
3.15
3.14
3.13
3.13
3.12
3.11
2.89
2.88
2.87
2.86
2.85
2.84
2.14
2.13
2.13
2.12
2.11
2.11
2.10
2.09
2.08
2.08
2.07
2.06
2.06
2.05
2.04
2.04
2.04
2.03
2.02
2.01
OMe
CN
N
O2N
1v
1.00
1.00
1.04
3.14
2.05
2.05
1.00
4.22
f1 (ppm)

126 MHz, $CDCl_3$

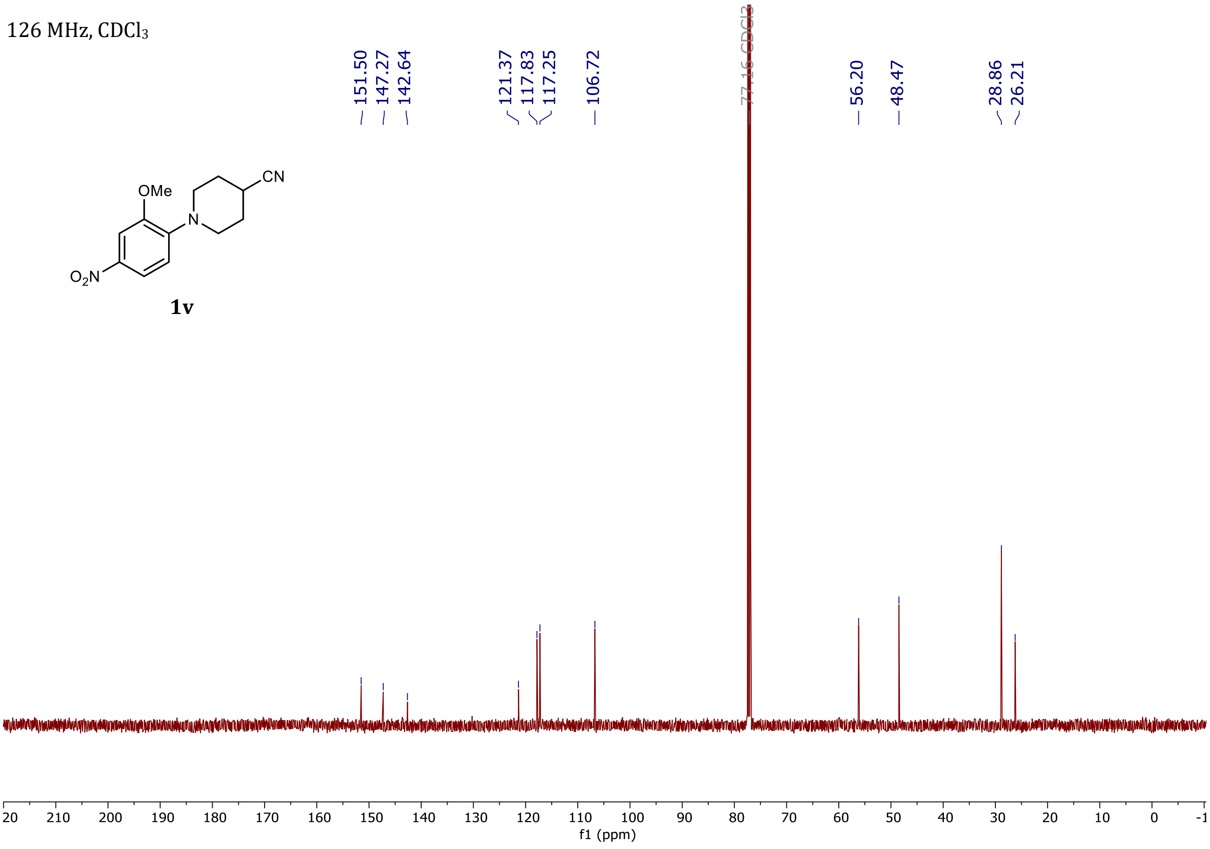

1v

500 MHz, $CDCl_3$

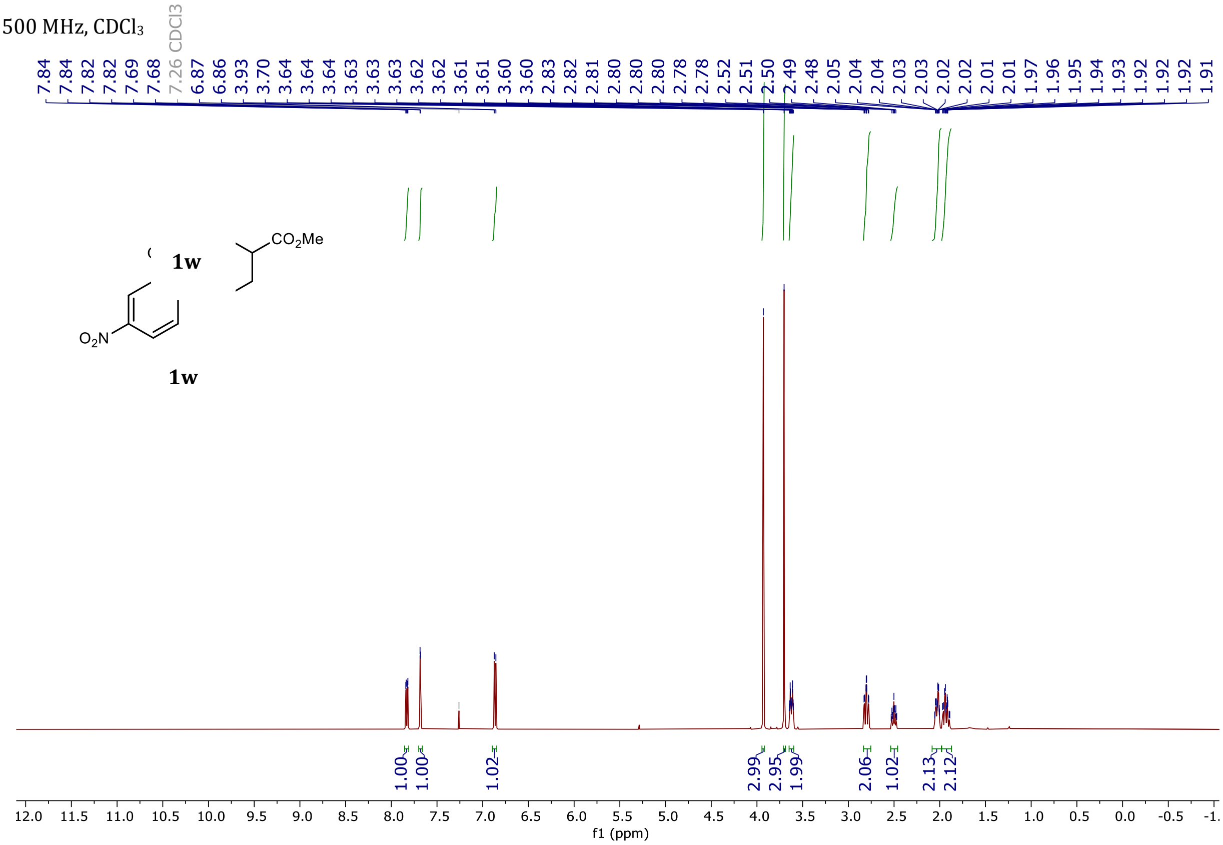

1w

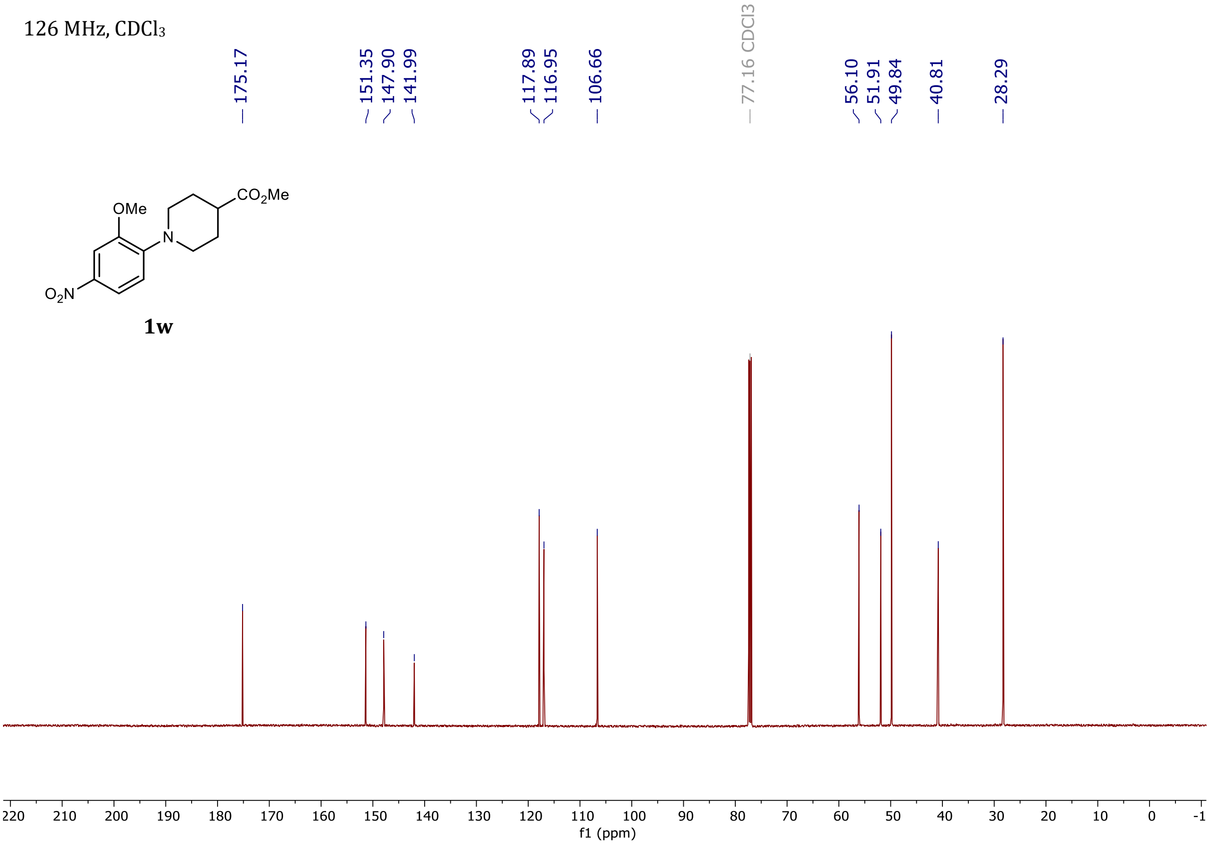
126 MHz, CDCl3
175.17
151.35
147.90
141.99
117.89
116.95
106.66
77.16 CDCl3
56.10
51.91
49.84
40.81
28.29
OMe
CO2Me
N
O2N
1w
f1 (ppm)

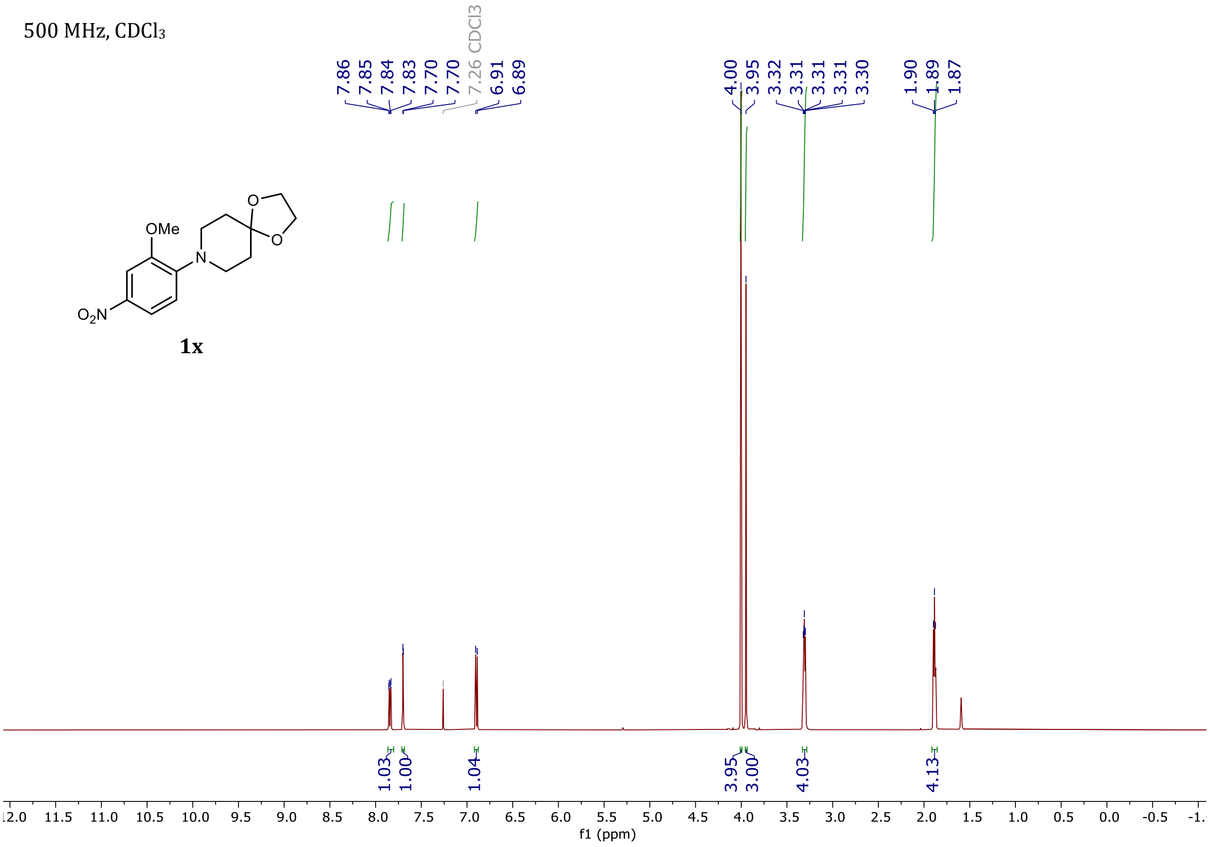
500 MHz, CDCl3
7.86
7.85
7.84
7.83
7.70
7.70
7.26 CDCl3
6.91
6.89
4.00
3.95
3.32
3.31
3.31
3.30
1.90
1.89
1.87
OMe
O
O
N
O2N
1x
1.03
1.00
1.04
3.95
3.00
4.03
4.13
f1 (ppm)

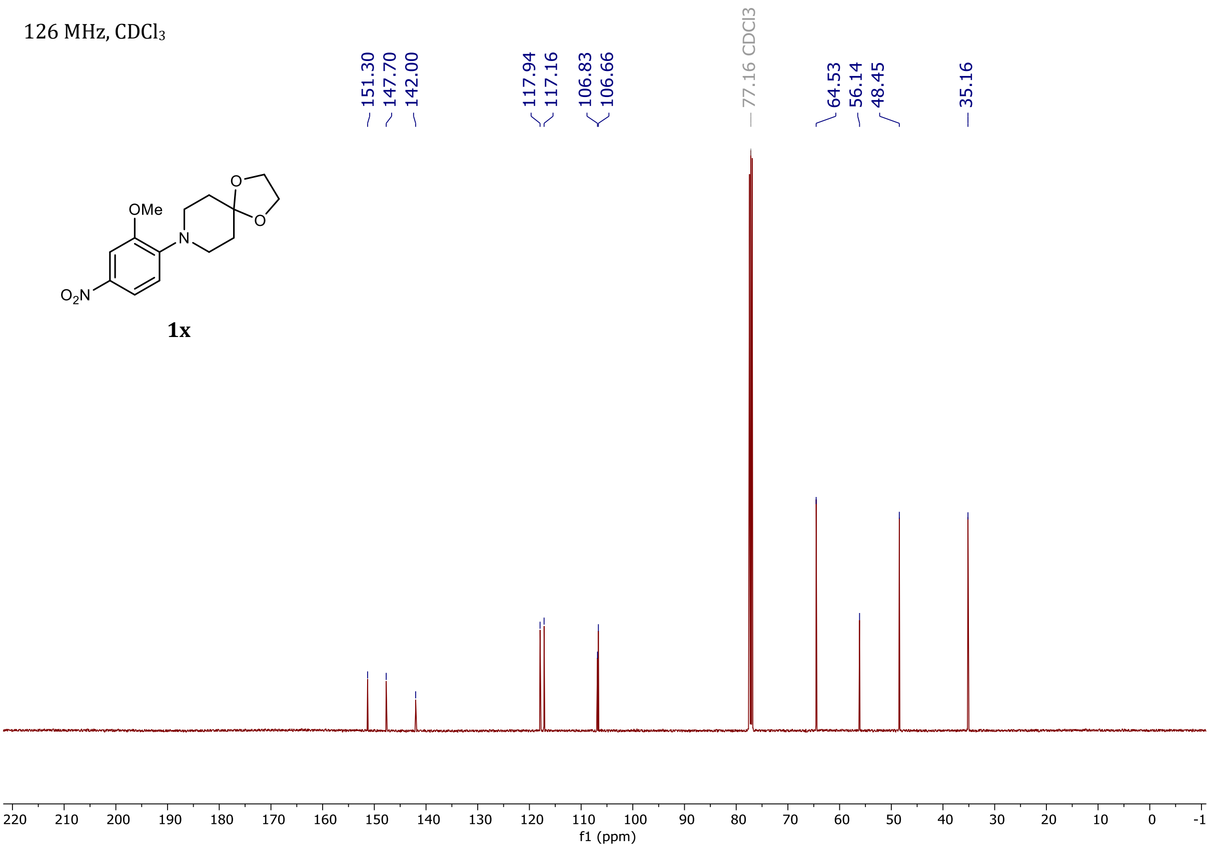
126 MHz, CDCl3
151.30
147.70
142.00
117.94
117.16
106.83
106.66
77.16 CDCl3
64.53
56.14
48.45
35.16
OMe
N
O
O
O2N
1x
220 210 200 190 180 170 160 150 140 130 120 110 100 90 80 70 60 50 40 30 20 10 0 -1
f1 (ppm)

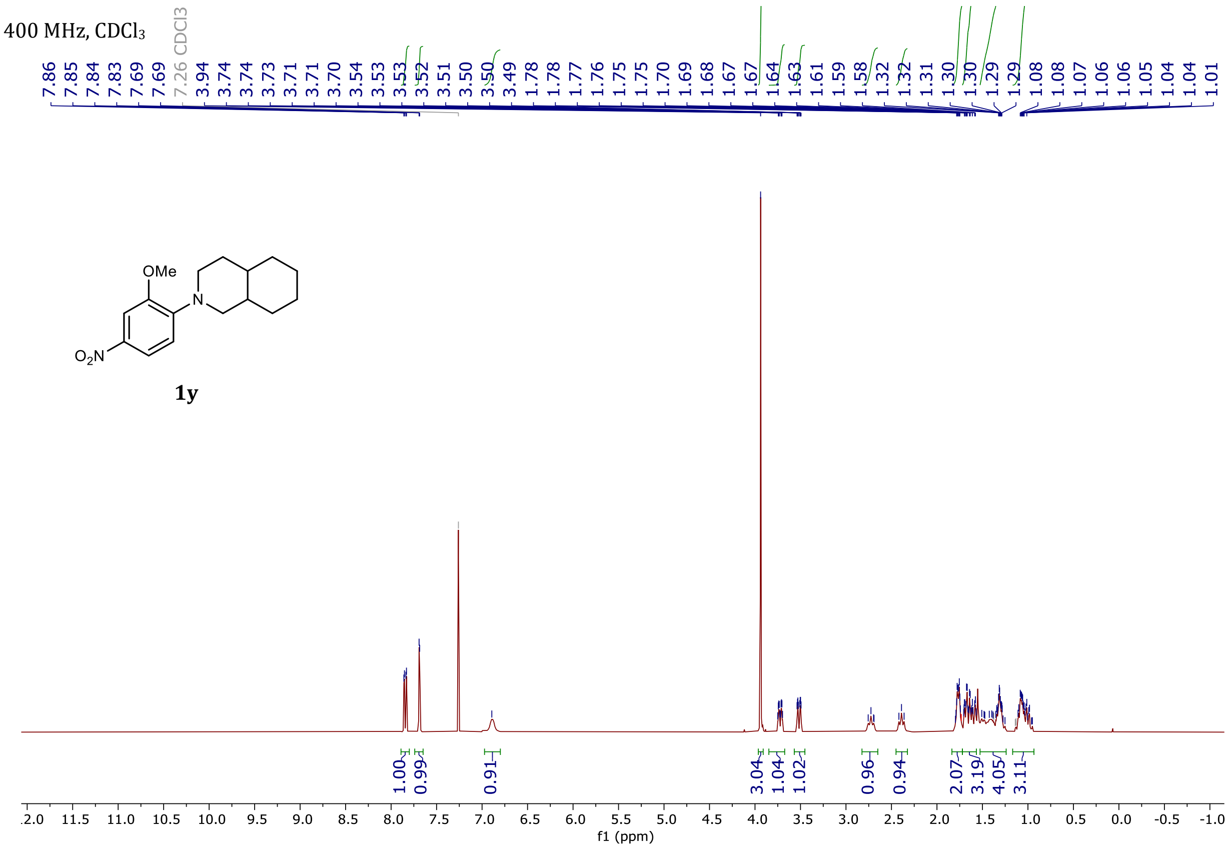
400 MHz, CDCl3
7.86 7.85 7.84 7.83 7.69 7.69 7.26 CDCl3 3.94 3.74 3.74 3.73 3.71 3.71 3.70 3.54 3.53 3.52 3.51 3.50 3.49 1.78 1.78 1.77 1.76 1.75 1.75 1.70 1.69 1.68 1.67 1.67 1.64 1.63 1.61 1.59 1.58 1.32 1.32 1.31 1.30 1.30 1.29 1.29 1.08 1.07 1.06 1.06 1.05 1.04 1.04 1.01
OMe
N
O2N
1y
1.00
0.99
0.91
3.04
1.04
1.02
0.96
0.94
2.07
3.19
4.05
3.11
.2.0 11.5 11.0 10.5 10.0 9.5 9.0 8.5 8.0 7.5 7.0 6.5 6.0 5.5 5.0 4.5 4.0 3.5 3.0 2.5 2.0 1.5 1.0 0.5 0.0 -0.5 -1.0
f1 (ppm)

101 MHz, CDCl3

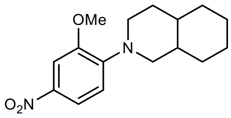

1y

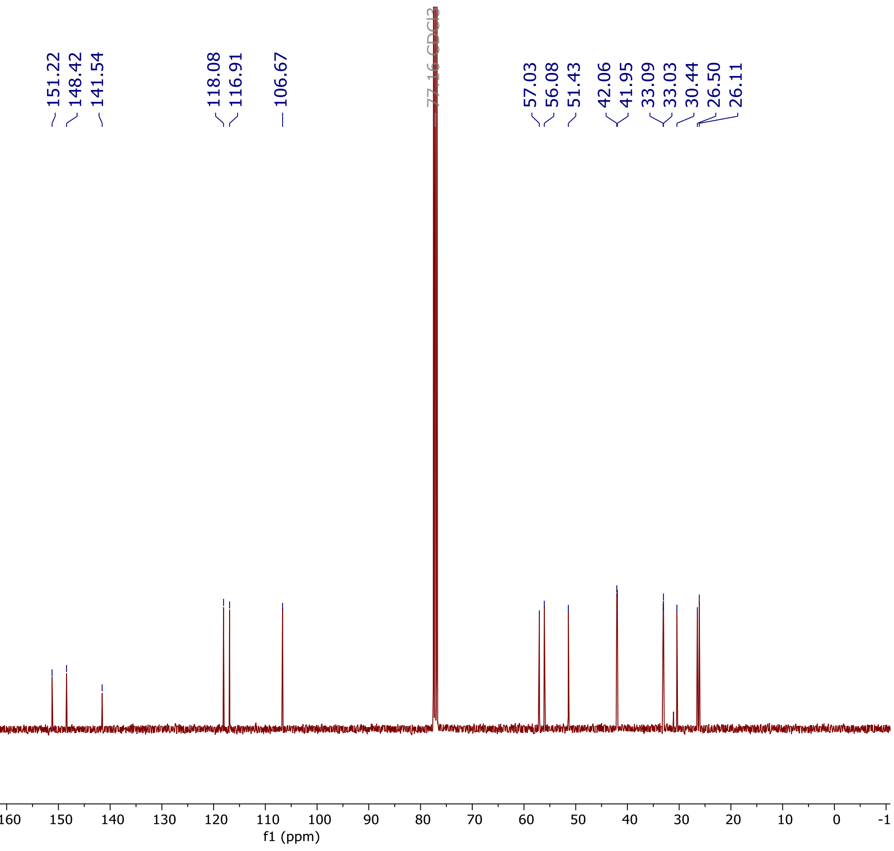

400 MHz, CDCl3

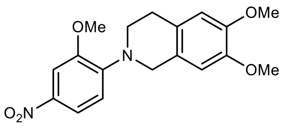

1z

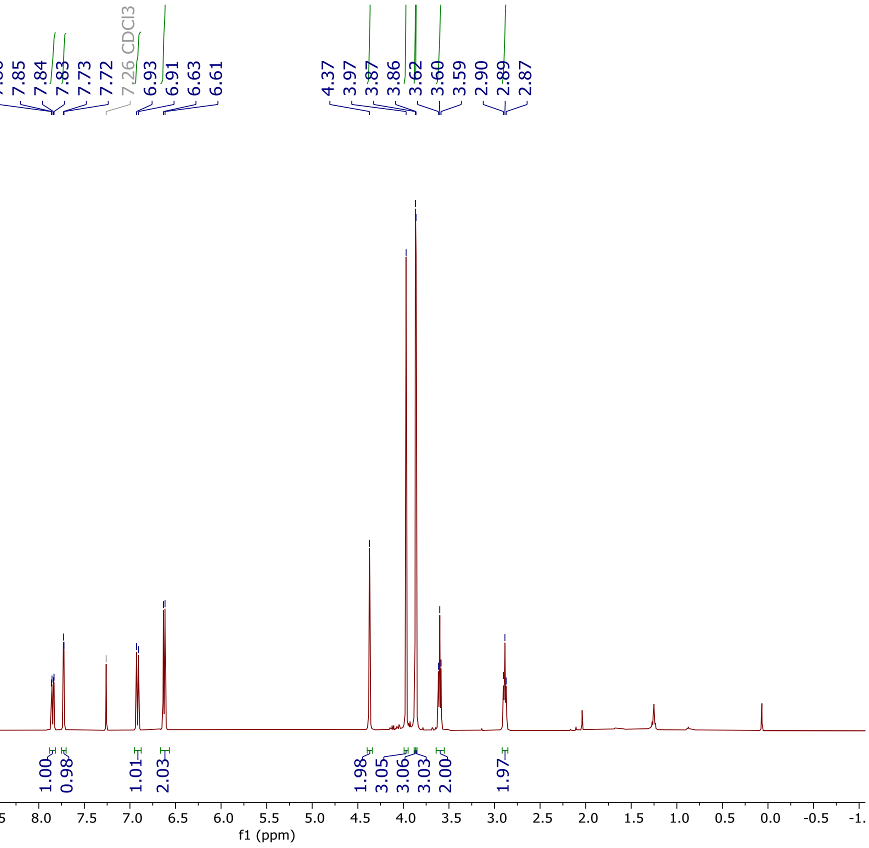

101 MHz, CDCl3z

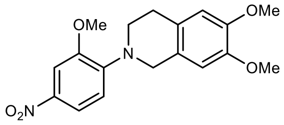

1z

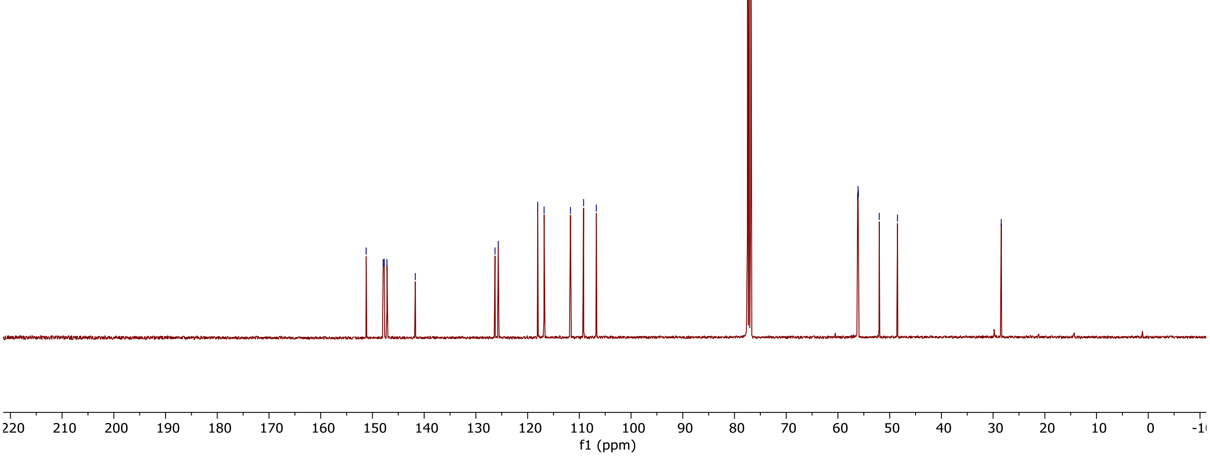

400 MHz, $CDCl_3$

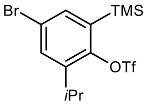

2ab

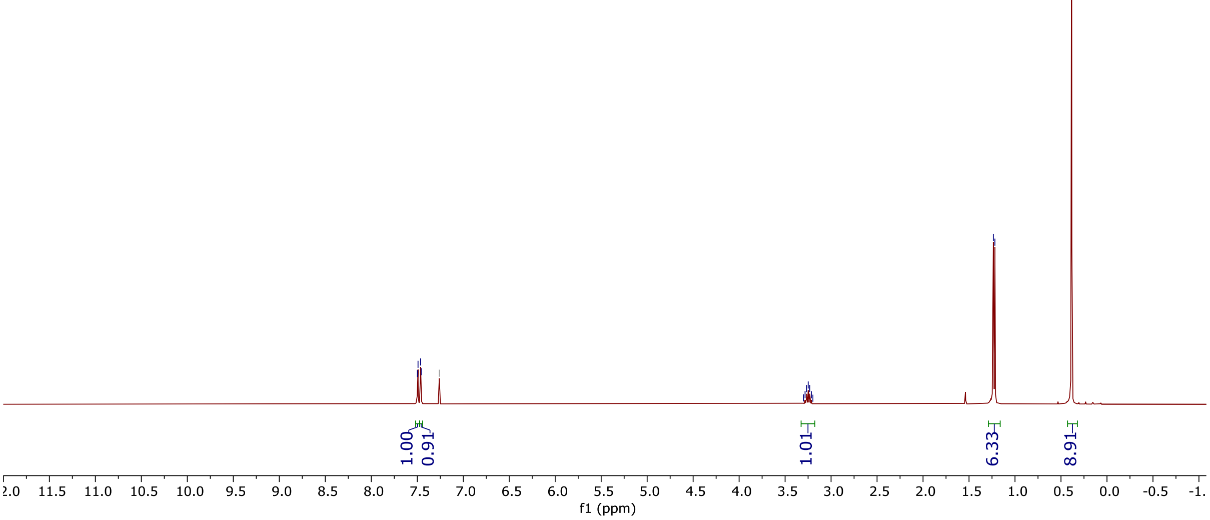

101 MHz, CDCl$_3$

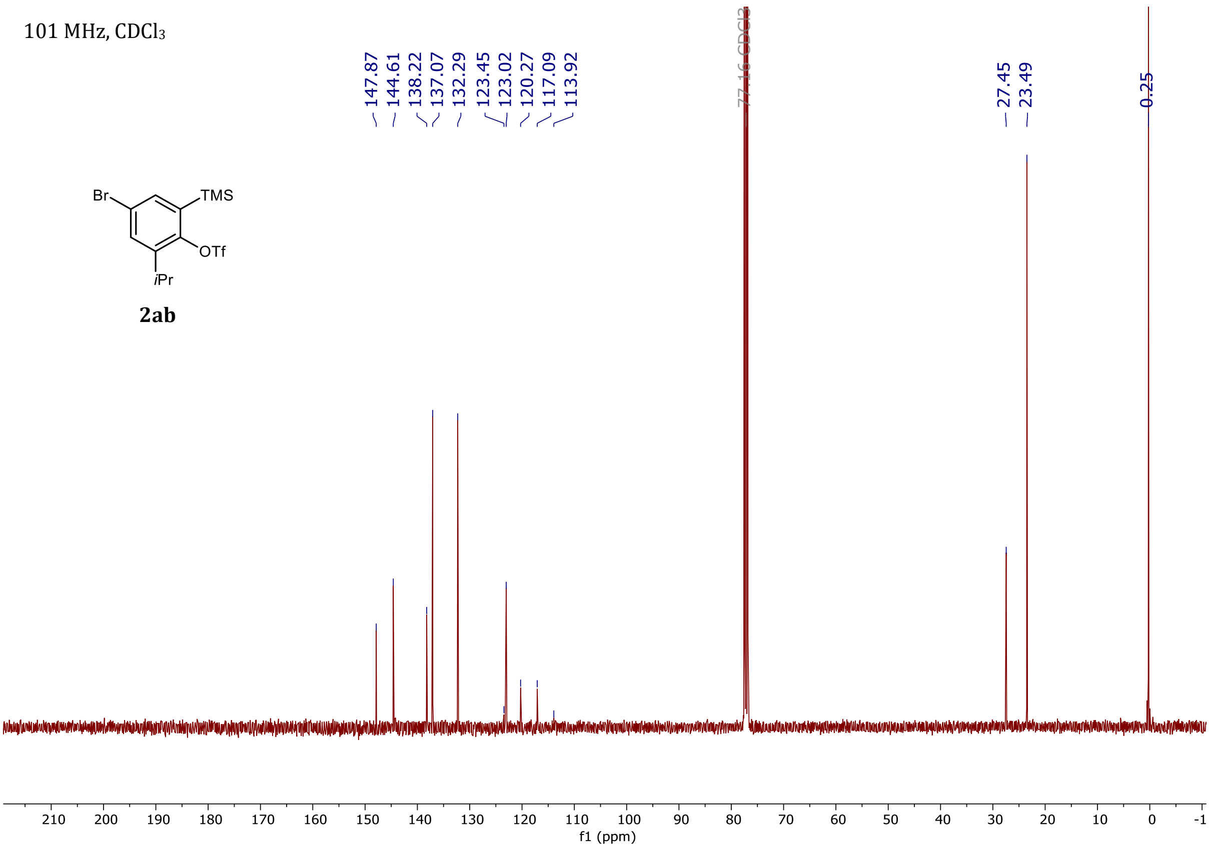

2ab

376 MHz, CDCl$_3$

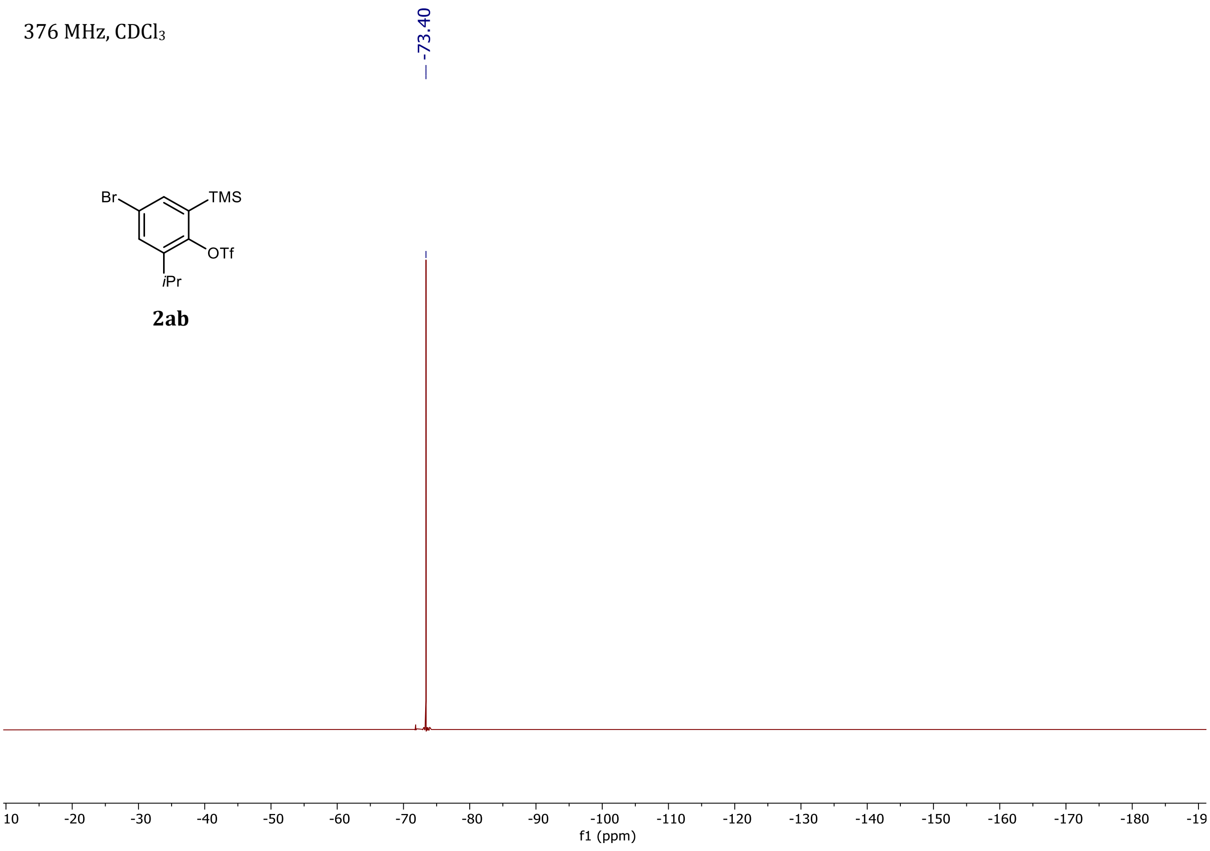

2ab

400 MHz, CDCl3

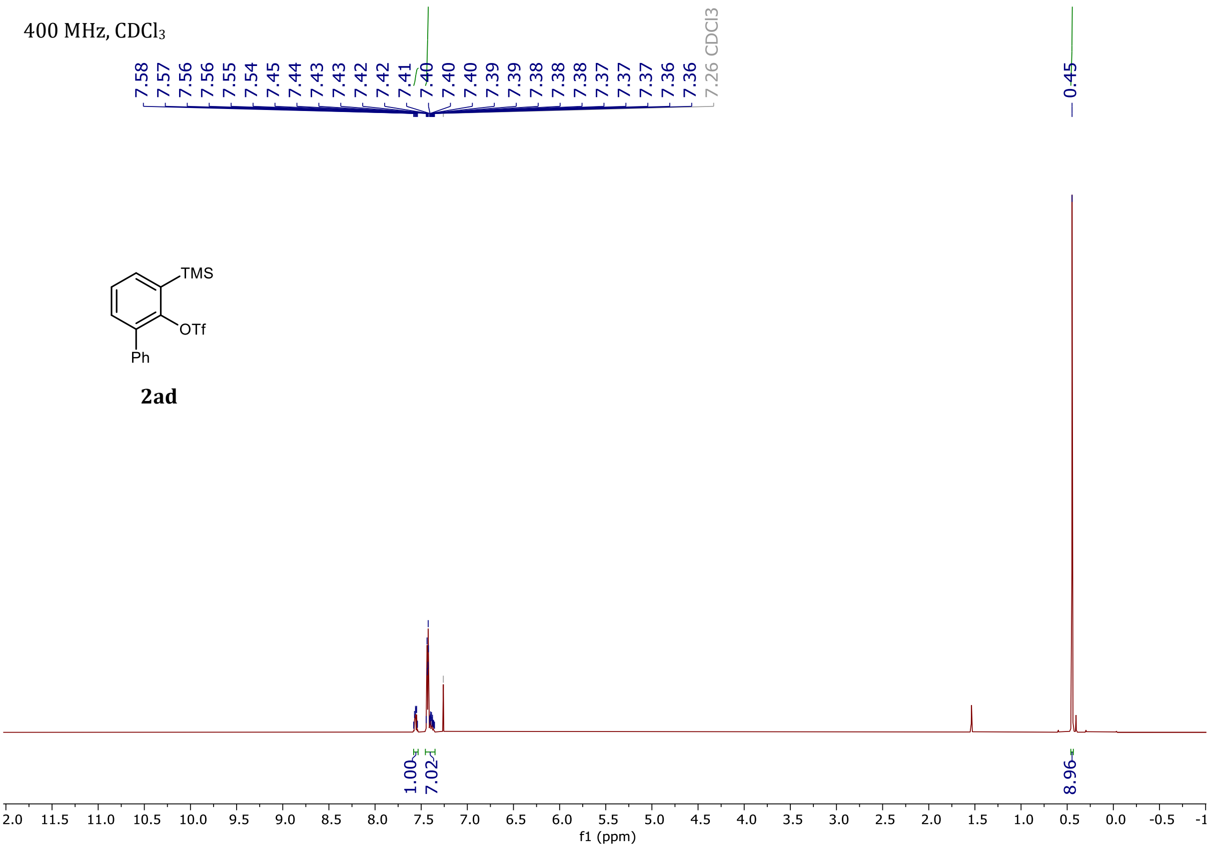

2ad

101 MHz, CDCl3

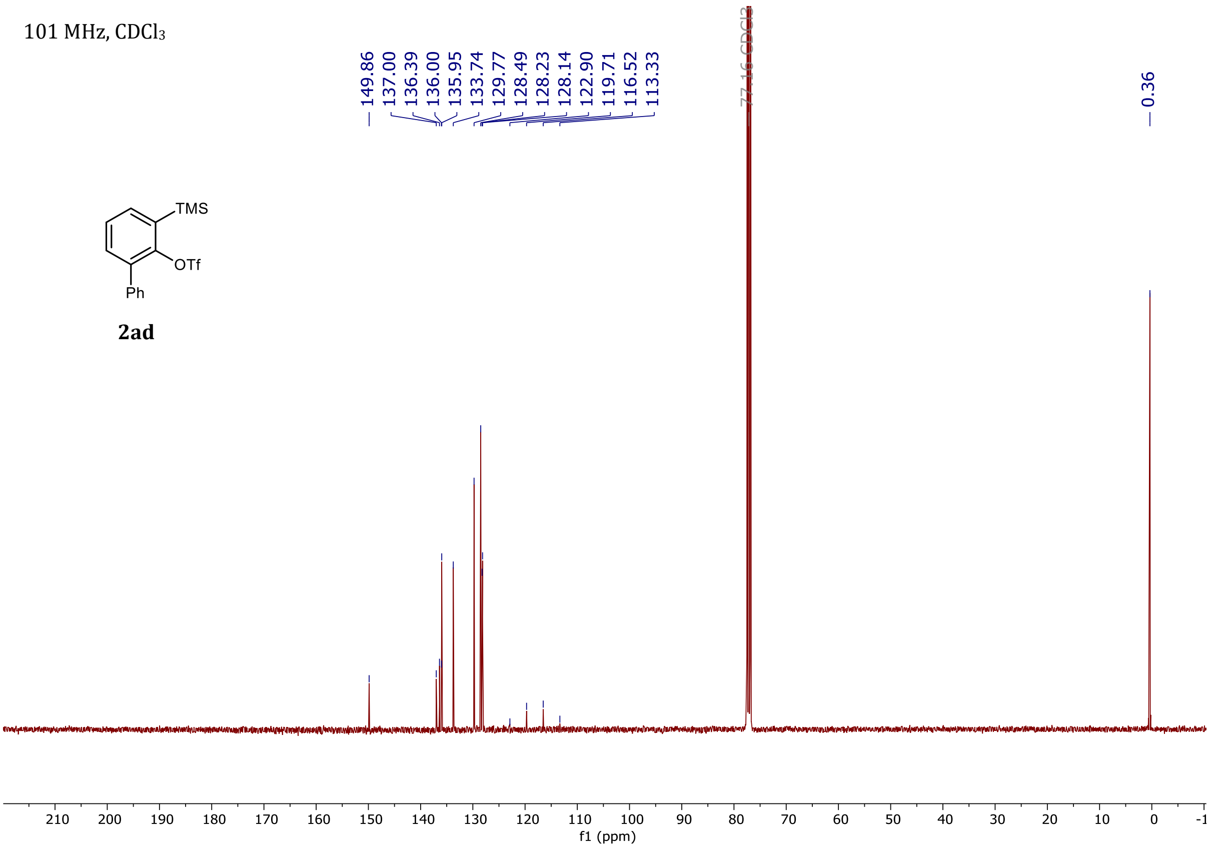

2ad

376 MHz, $CDCl_3$

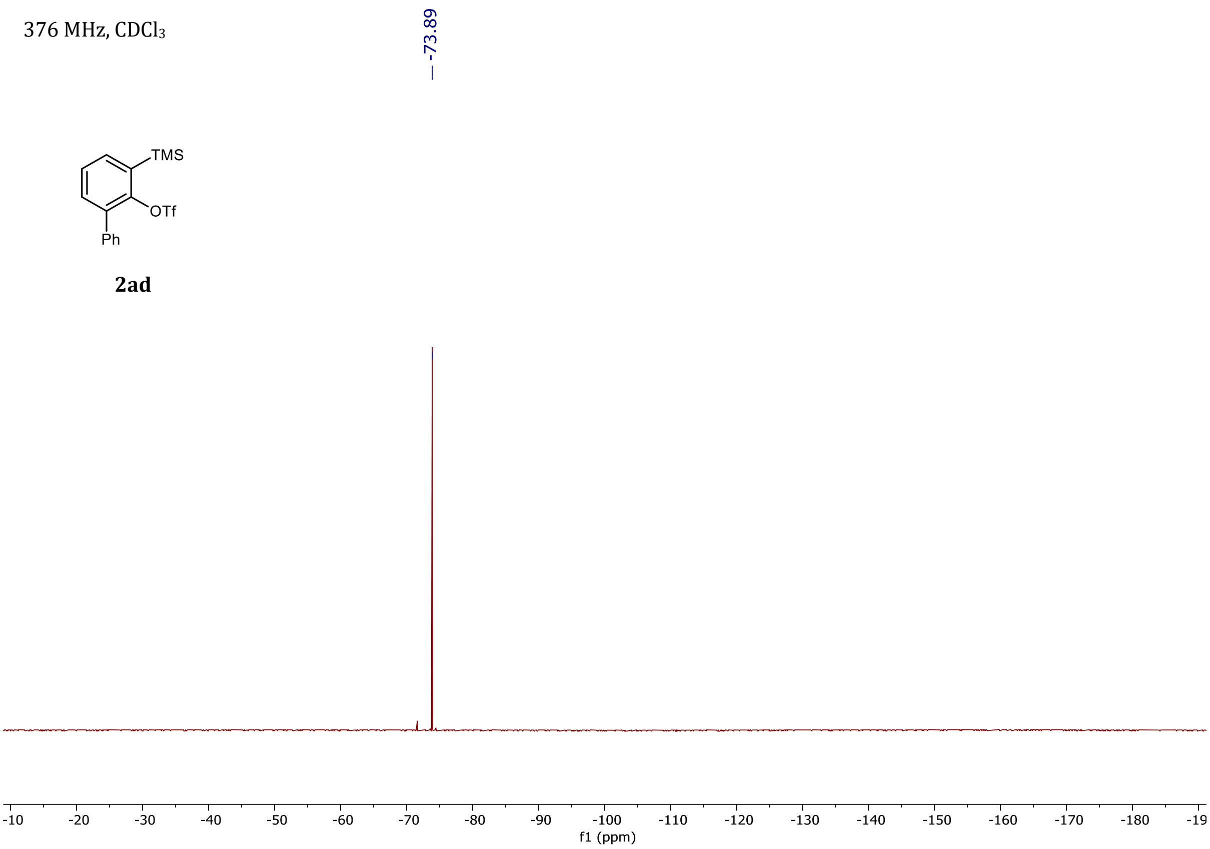

2ad

400 MHz, $CDCl_3$

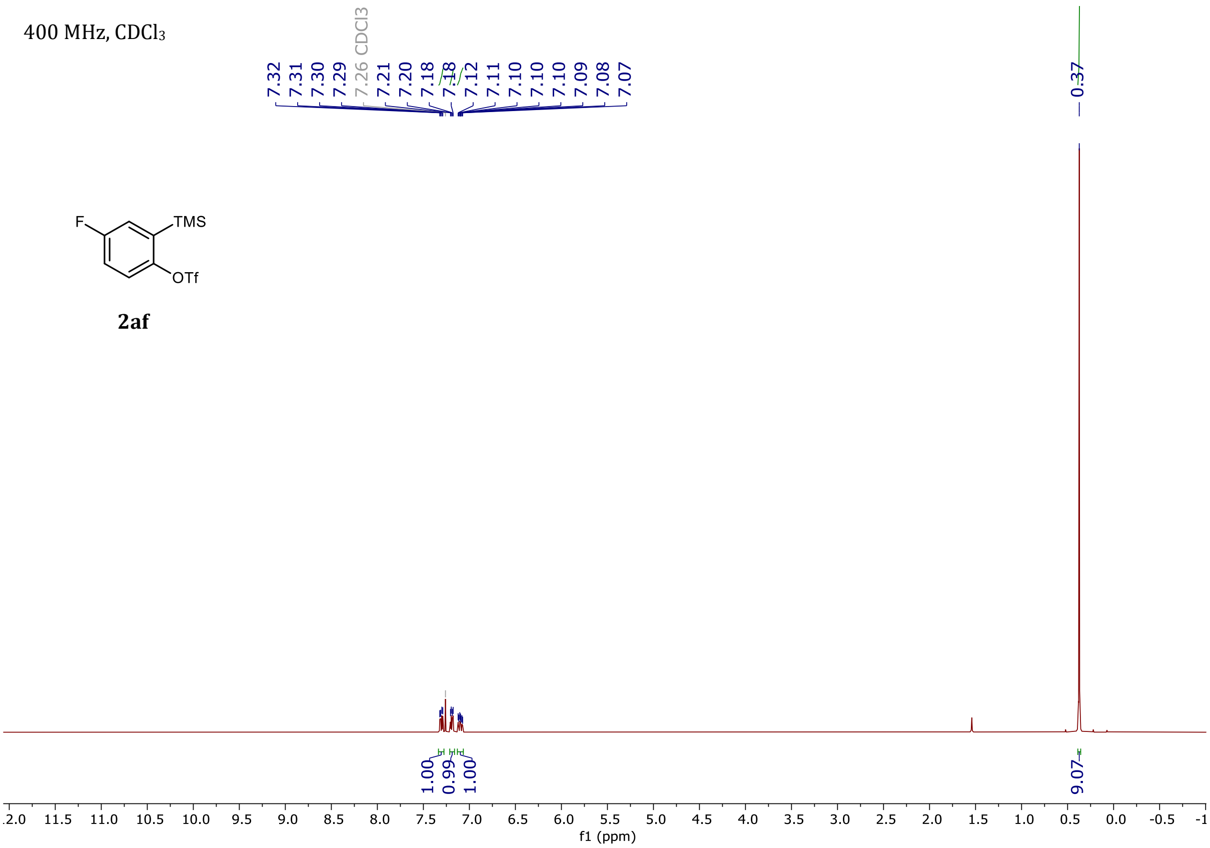

2af

101 MHz, $CDCl_3$

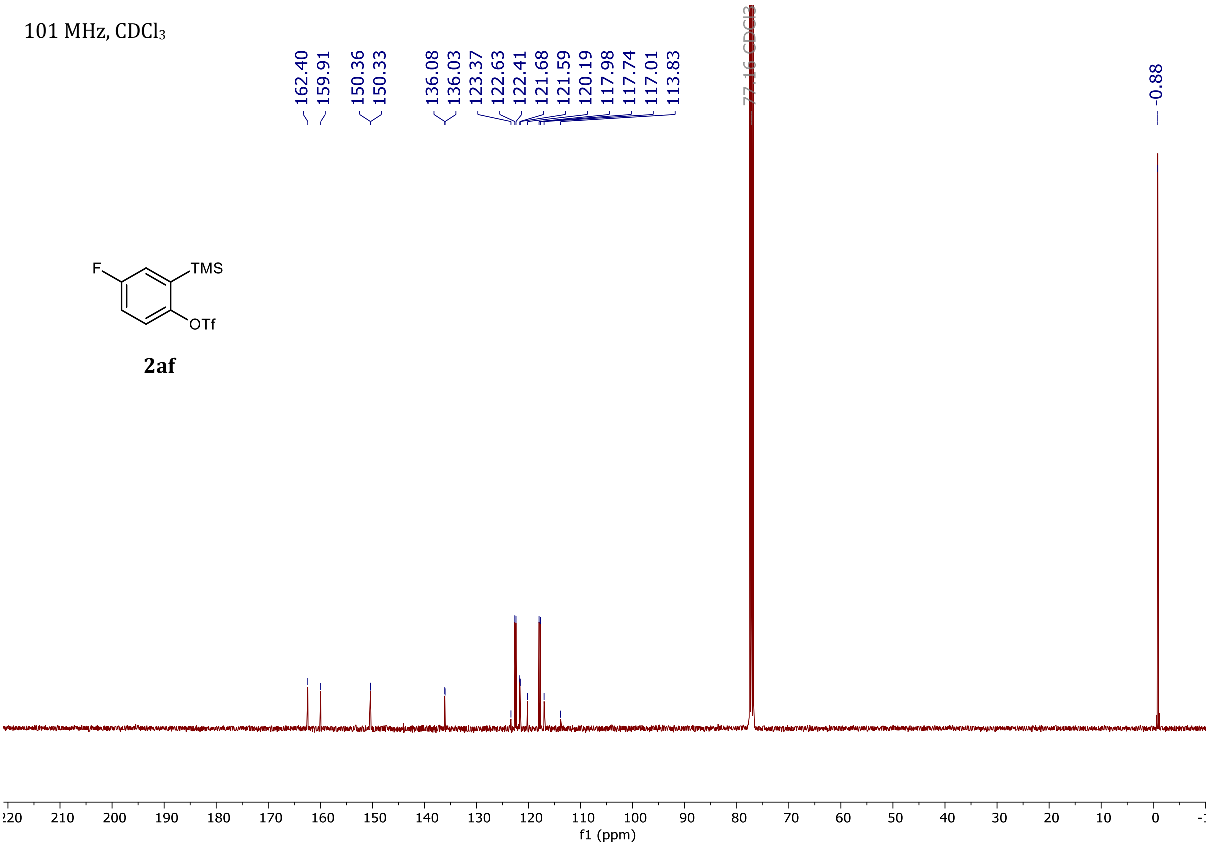

2af

376 MHz, $CDCl_3$

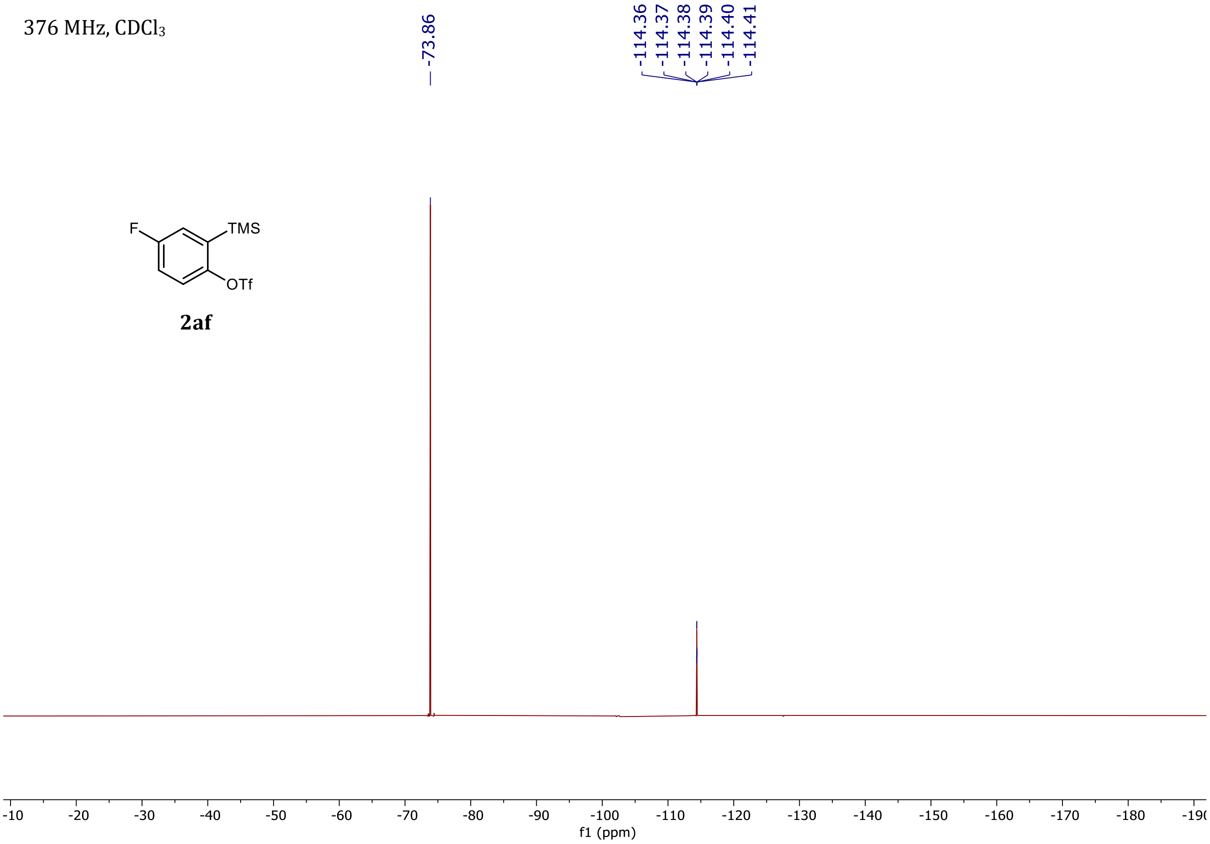

2af

500 MHz, $CDCl_3$

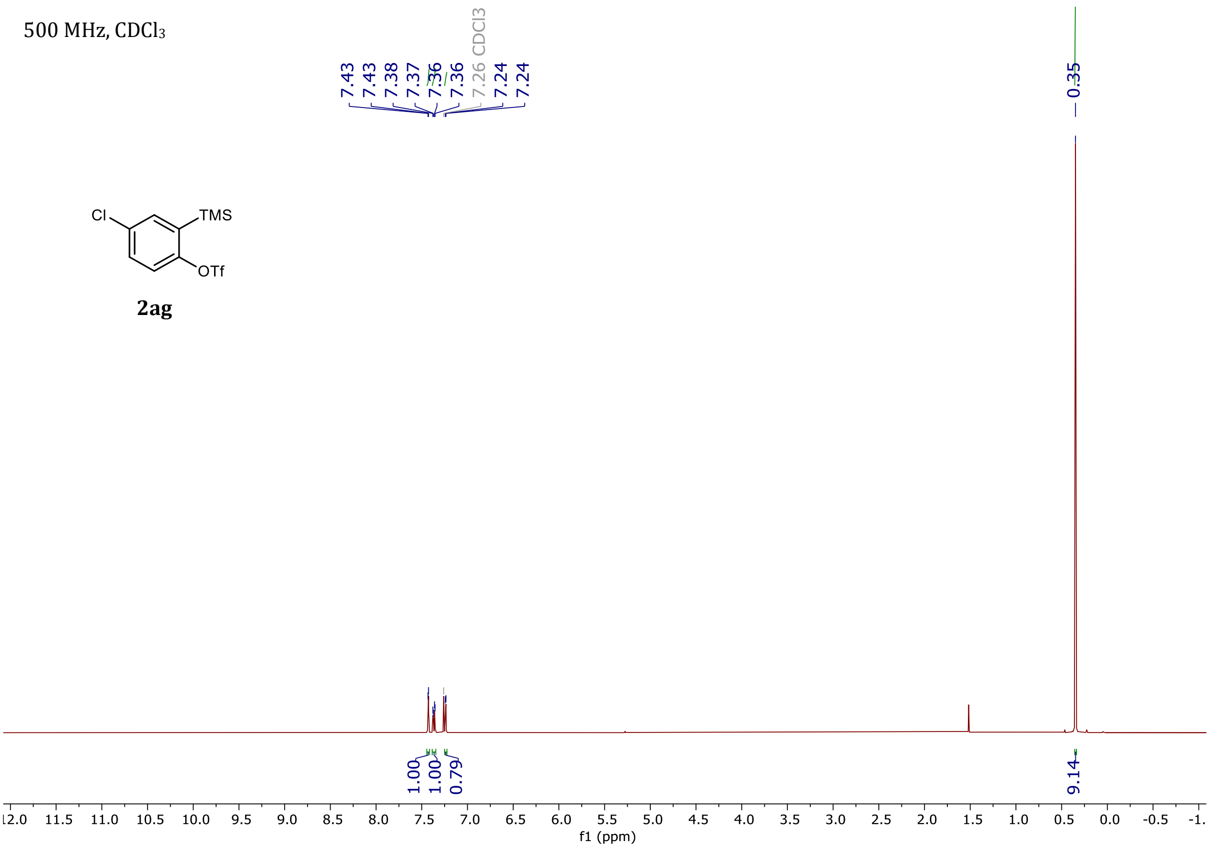

2ag

101 MHz, $CDCl_3$

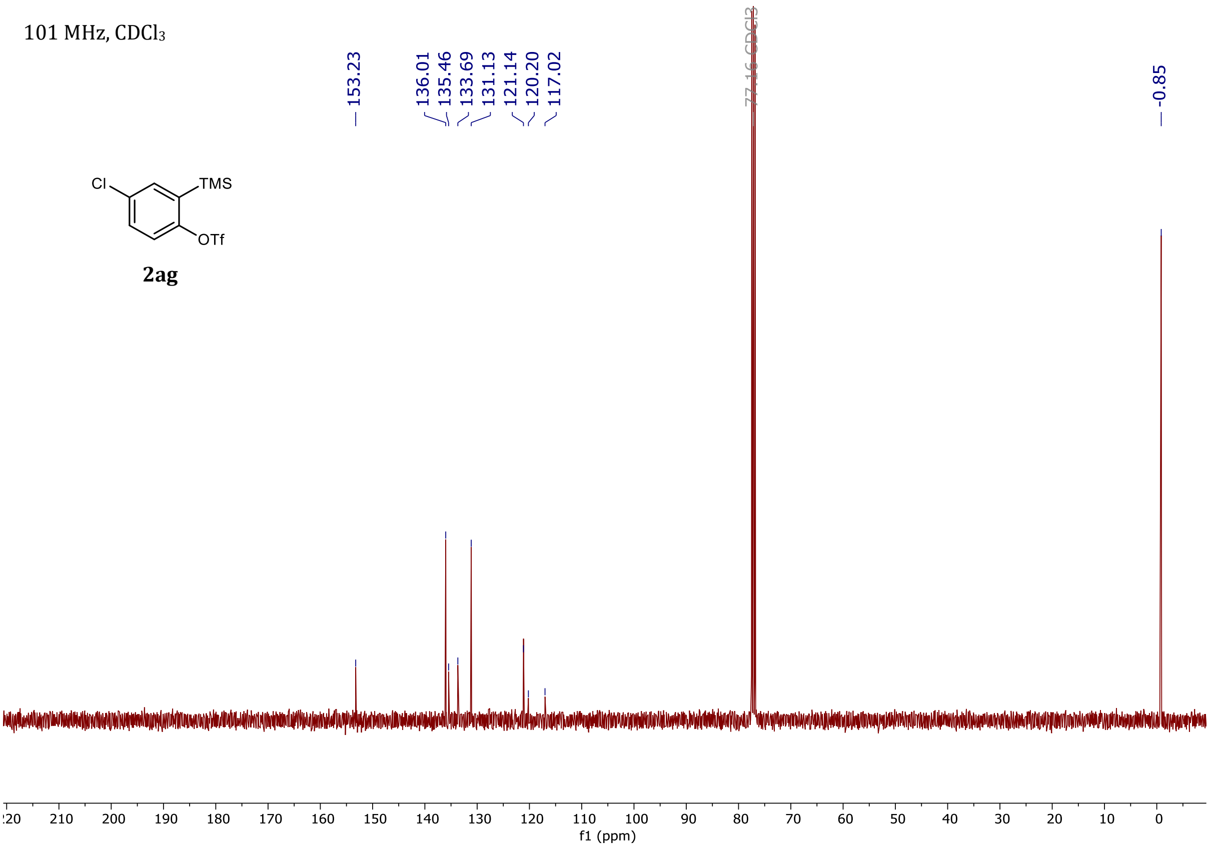

2ag

376 MHz, CDCl₃

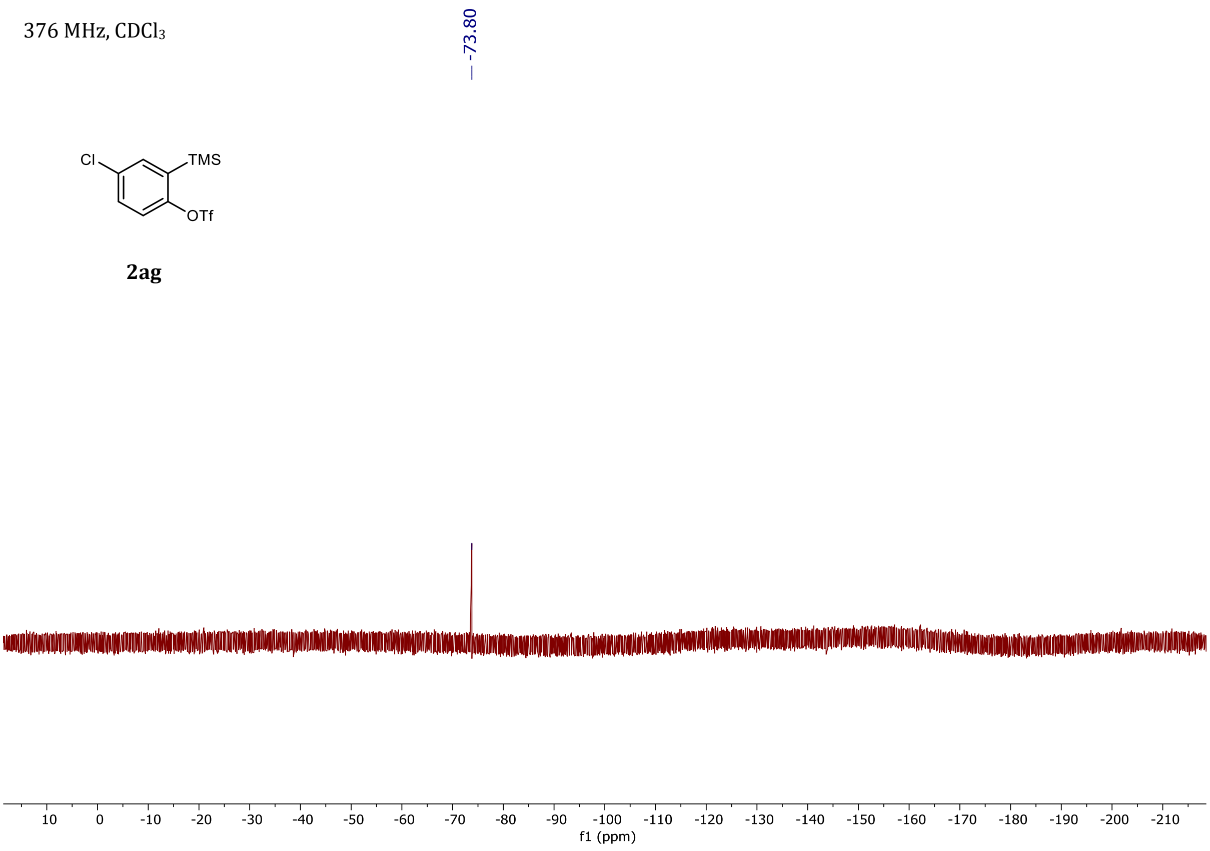

2ag

400 MHz, CDCl₃

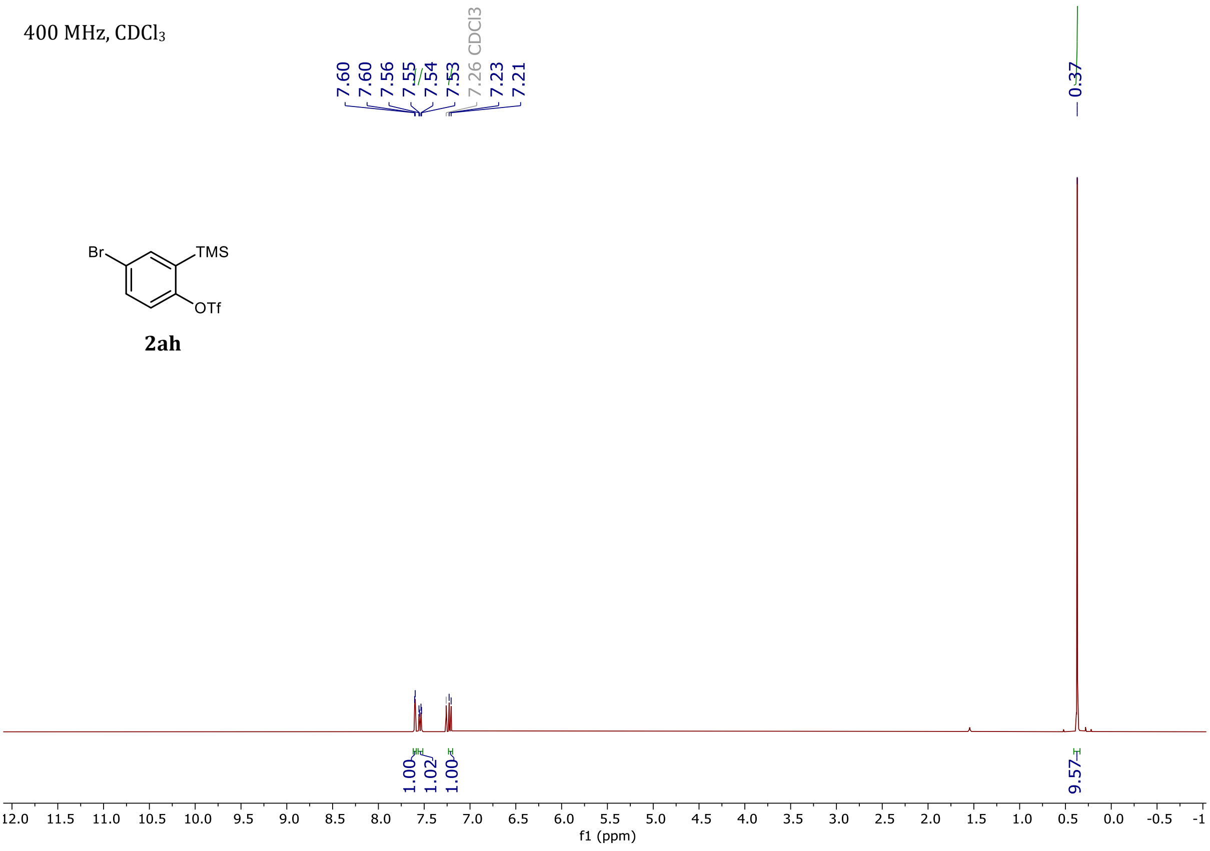

2ah

101 MHz, CDCl$_3$

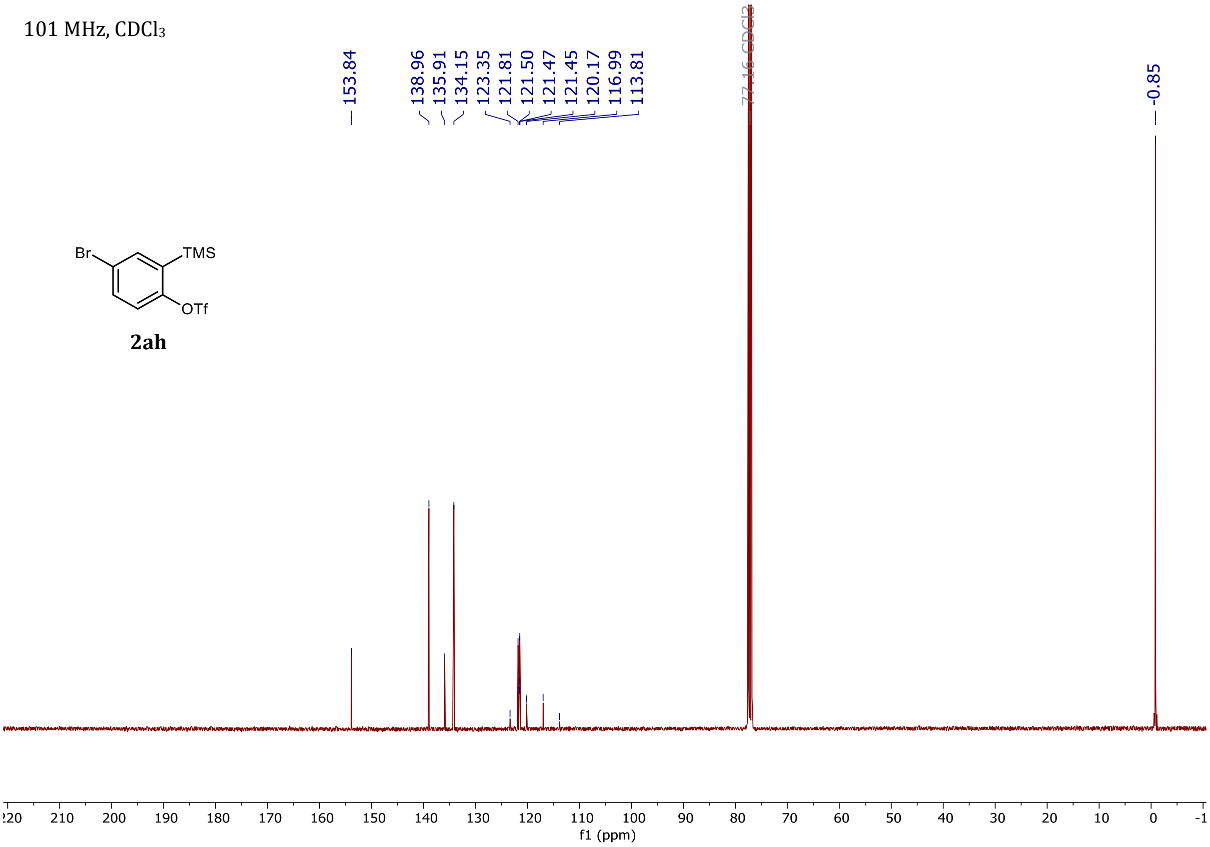

2ah

376 MHz, CDCl$_3$

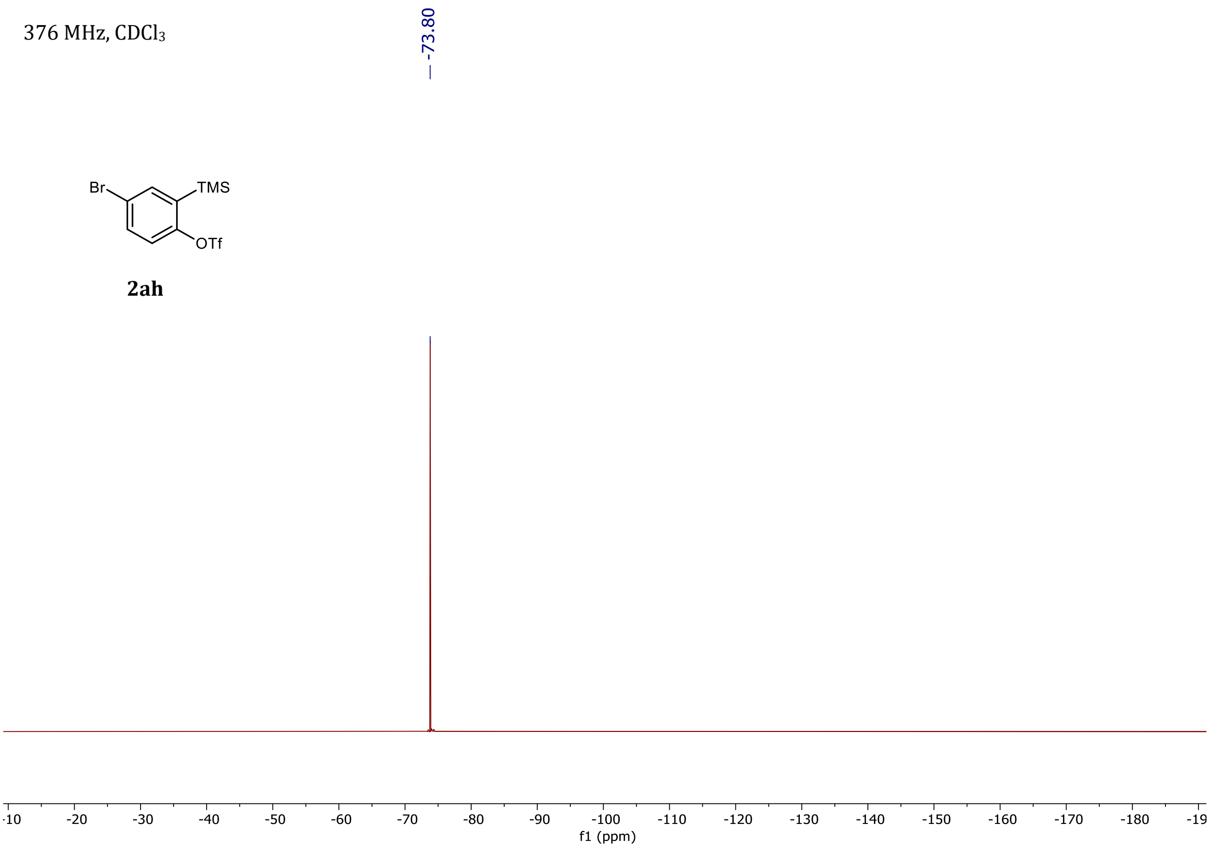

2ah

400 MHz, $CDCl_3$

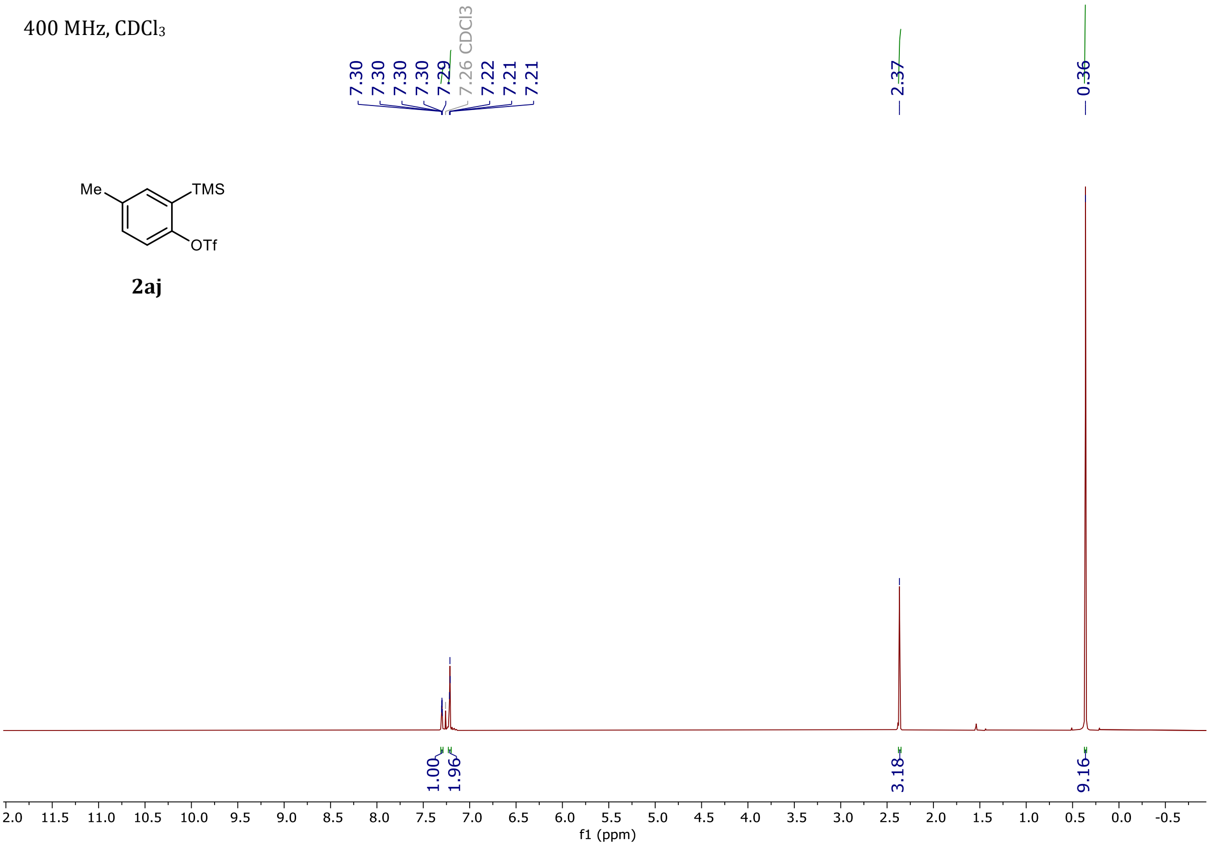

101 MHz, $CDCl_3$

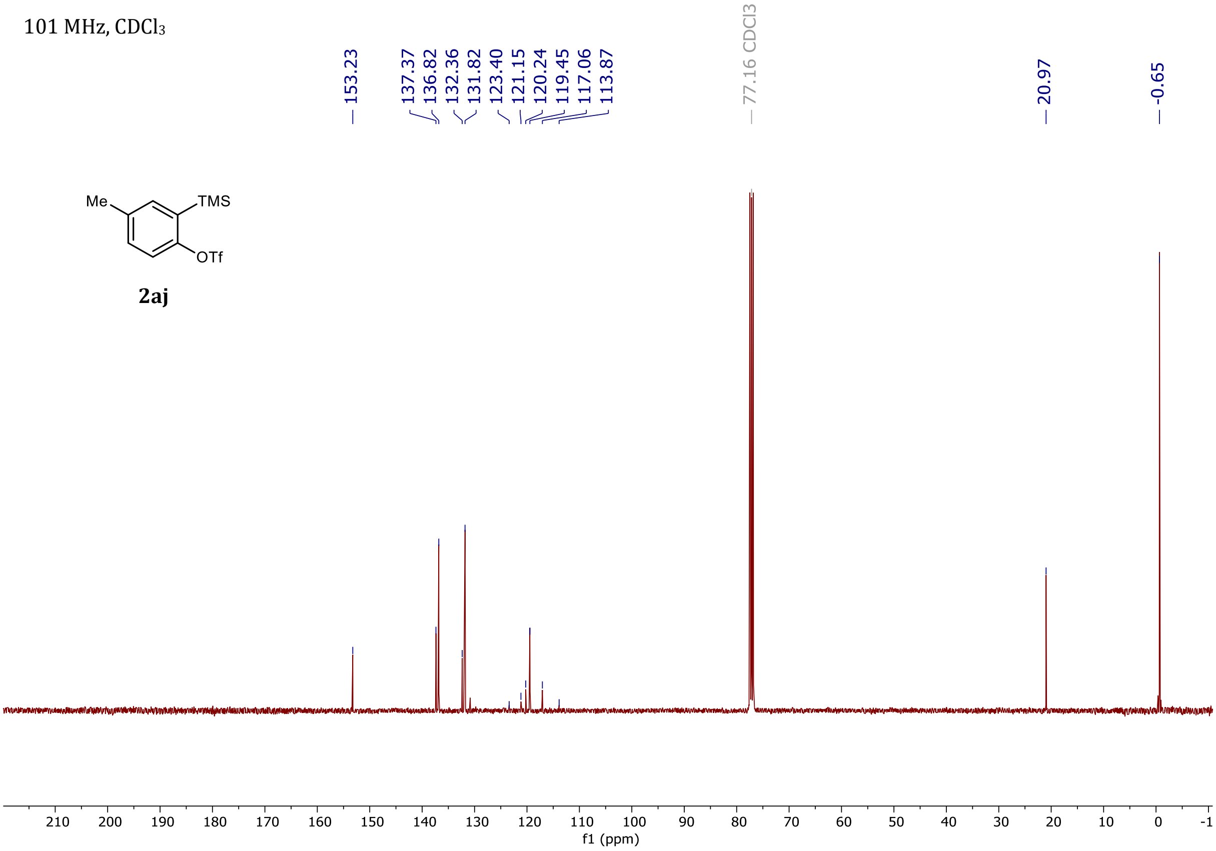

376 MHz, $CDCl_3$

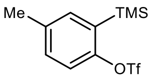

2aj

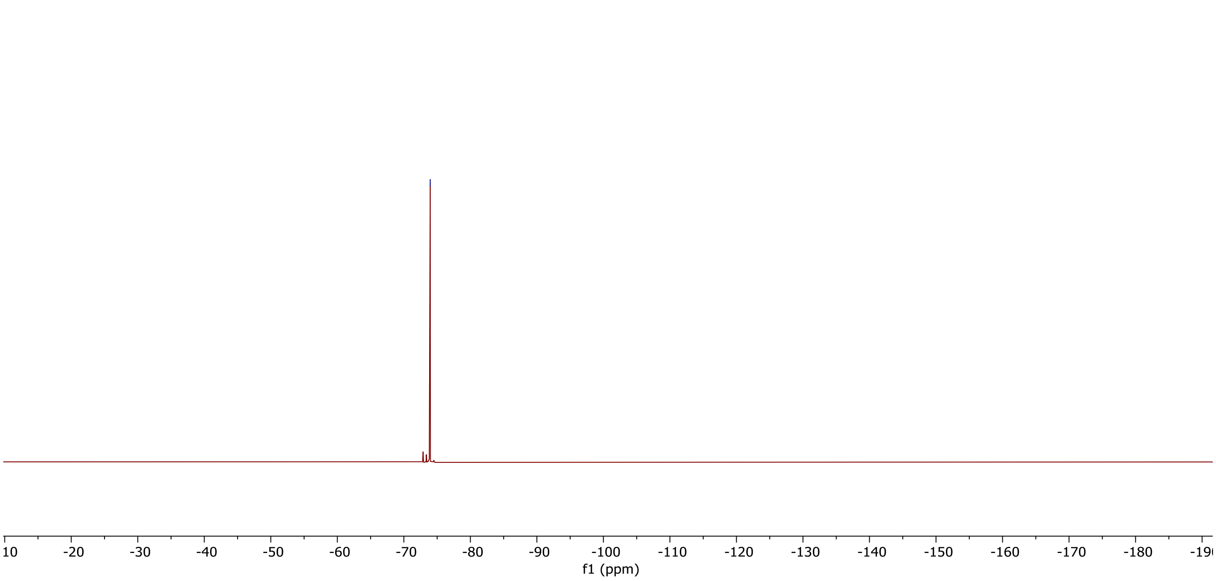

400 MHz, $CDCl_3$

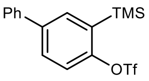

2ak

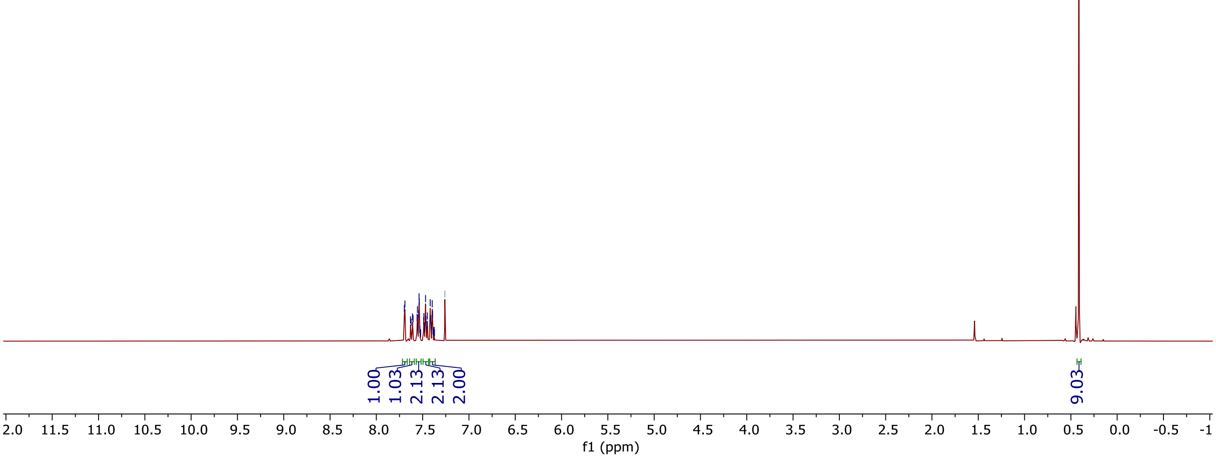

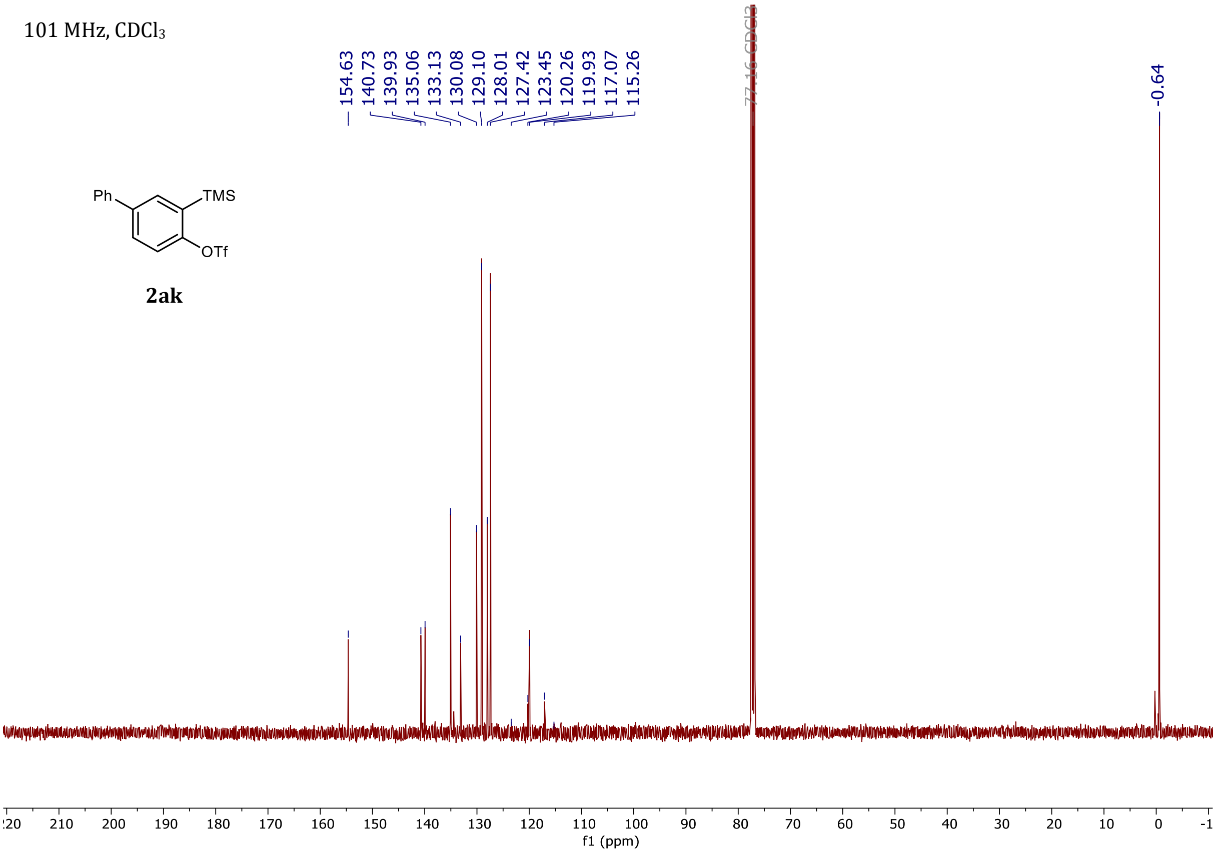
101 MHz, CDCl$_3$
154.63
140.73
139.93
135.06
133.13
130.08
129.10
128.01
127.42
123.45
120.26
119.93
117.07
115.26
77.16 CDCl3
-0.64
Ph
TMS
OTf
2ak
f1 (ppm)

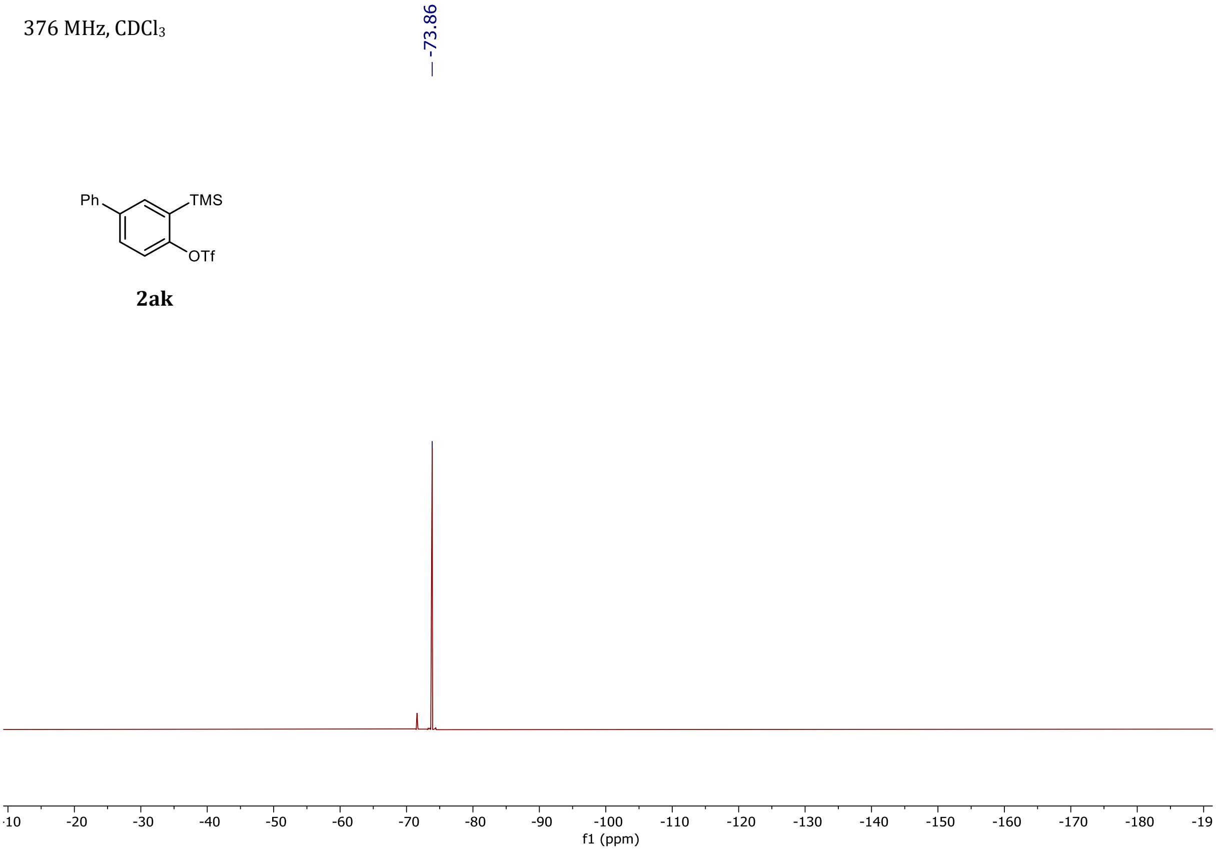
376 MHz, CDCl$_3$
-73.86
Ph
TMS
OTf
2ak
f1 (ppm)

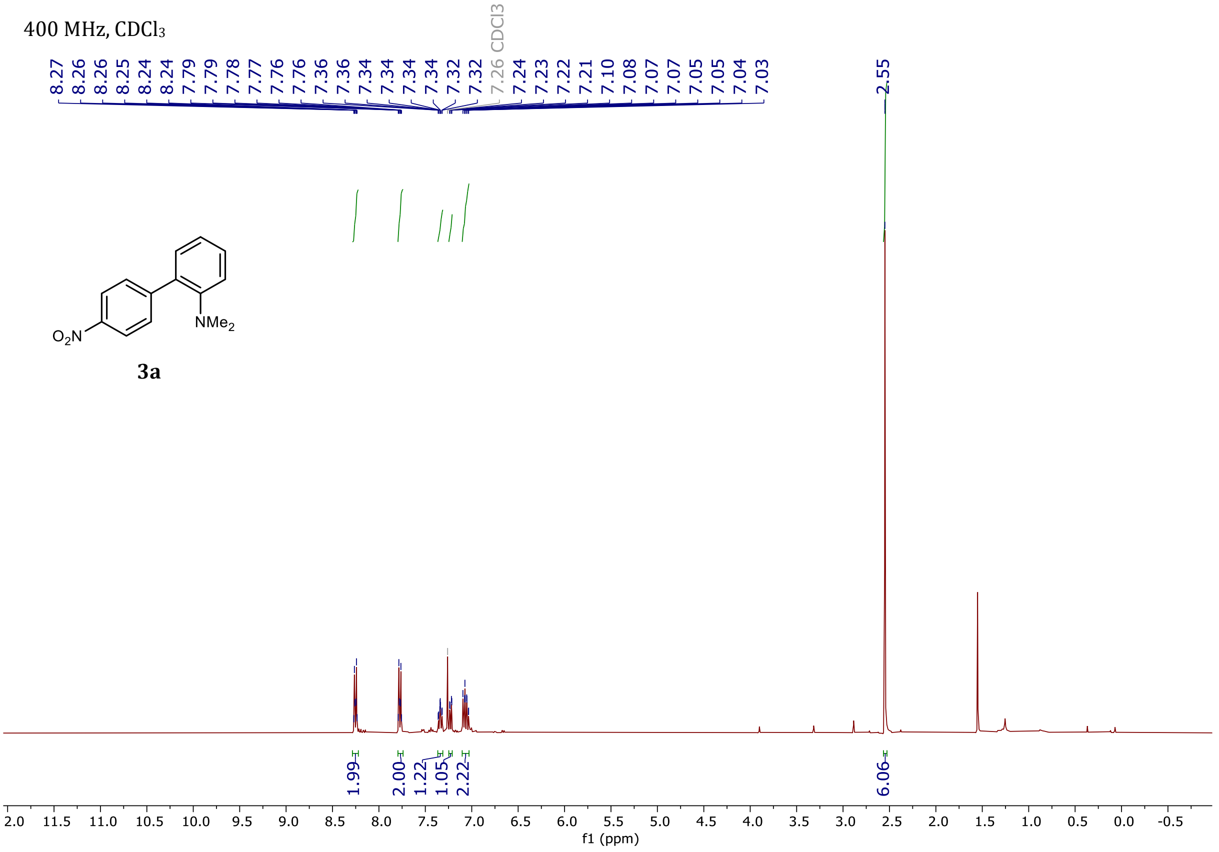

400 MHz, CDCl3
8.27 8.26 8.26 8.25 8.24 8.24 7.79 7.79 7.78 7.77 7.76 7.36 7.36 7.34 7.34 7.34 7.32 7.32 7.26 CDCl3 7.24 7.23 7.22 7.21 7.10 7.08 7.07 7.07 7.05 7.05 7.04 7.03
2.55
NMe2
O2N
3a
1.99 2.00 1.22 1.05 2.22
6.06
f1 (ppm)

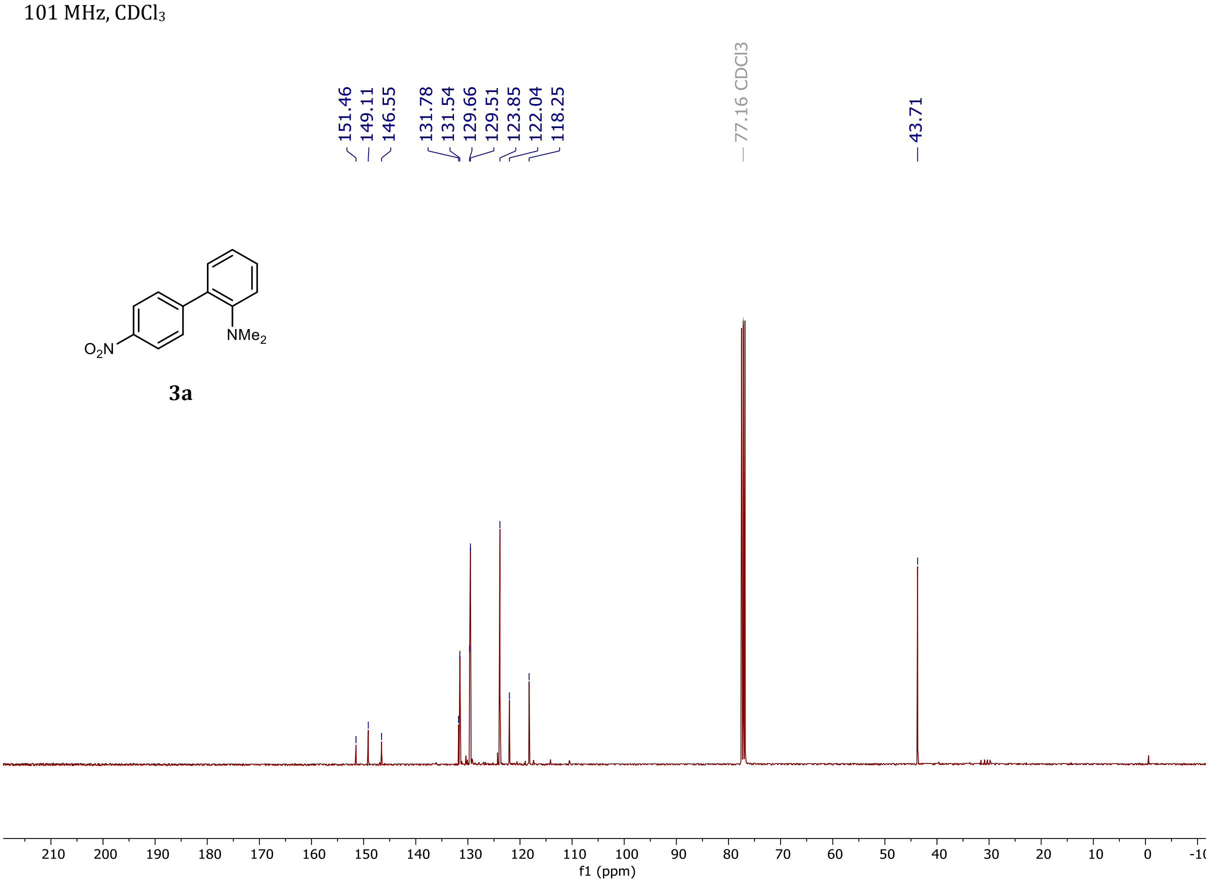

101 MHz, CDCl3
151.46 149.11 146.55 131.78 131.54 129.66 129.51 123.85 122.04 118.25
77.16 CDCl3
43.71
NMe2
O2N
3a
f1 (ppm)

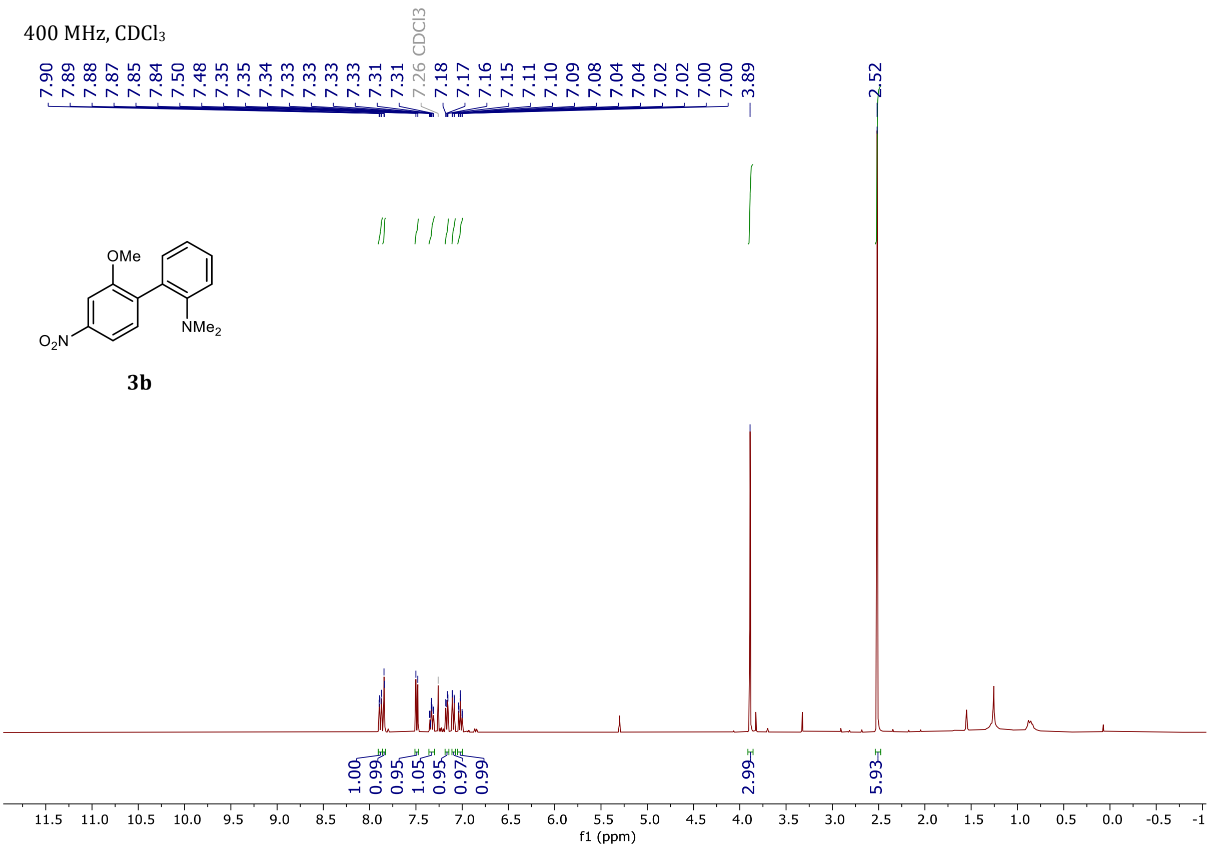
400 MHz, CDCl3
7.90 7.89 7.88 7.87 7.85 7.84 7.50 7.48 7.35 7.35 7.34 7.33 7.33 7.33 7.31 7.31 7.26 CDCl3 7.18 7.17 7.16 7.15 7.11 7.10 7.09 7.08 7.04 7.04 7.02 7.00 7.00 3.89 2.52
OMe
O2N
NMe2
3b
1.00 0.99 0.95 1.05 0.95 0.97 0.99 2.99 5.93
f1 (ppm)

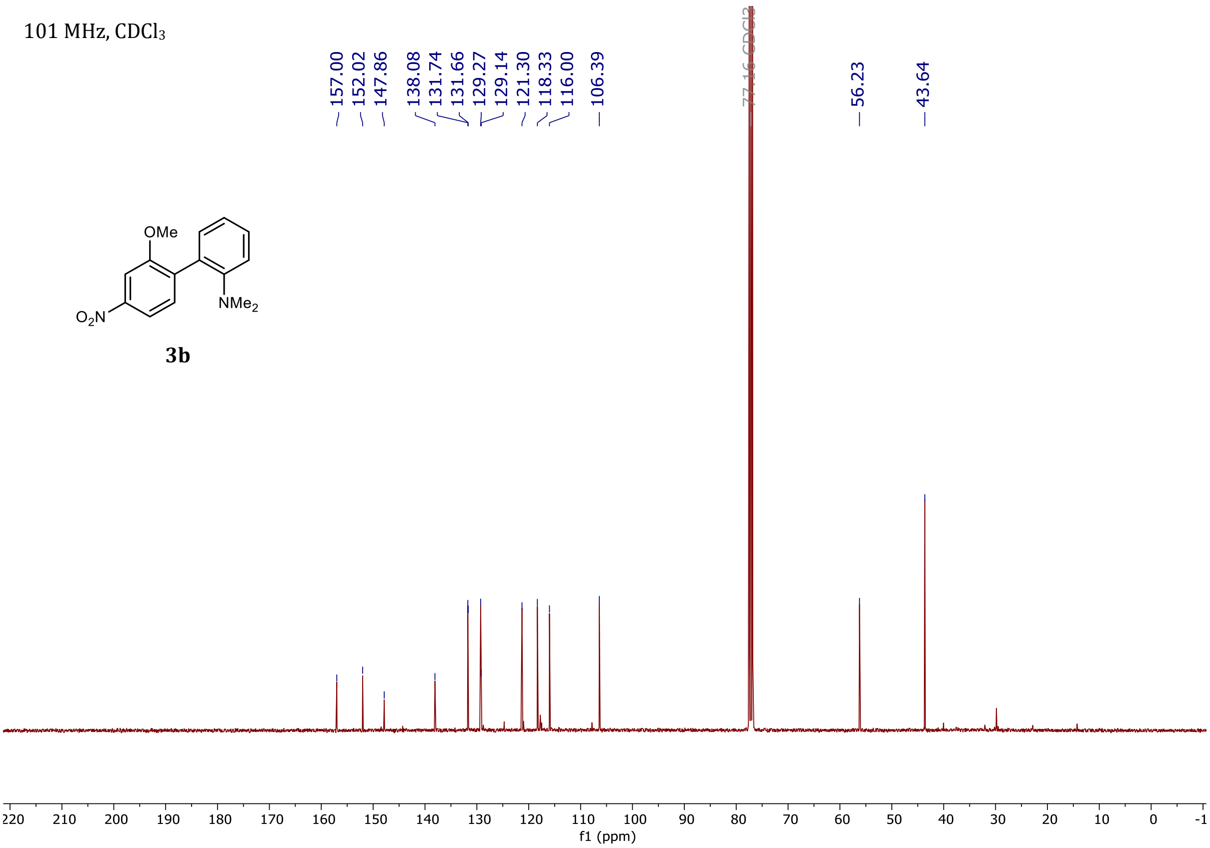
101 MHz, CDCl3
157.00 152.02 147.86 138.08 131.74 131.66 129.27 129.14 121.30 118.33 116.00 106.39 77.16 CDCl3 56.23 43.64
OMe
O2N
NMe2
3b
f1 (ppm)

400 MHz, CDCl3

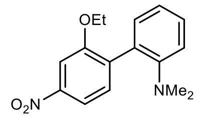

3c

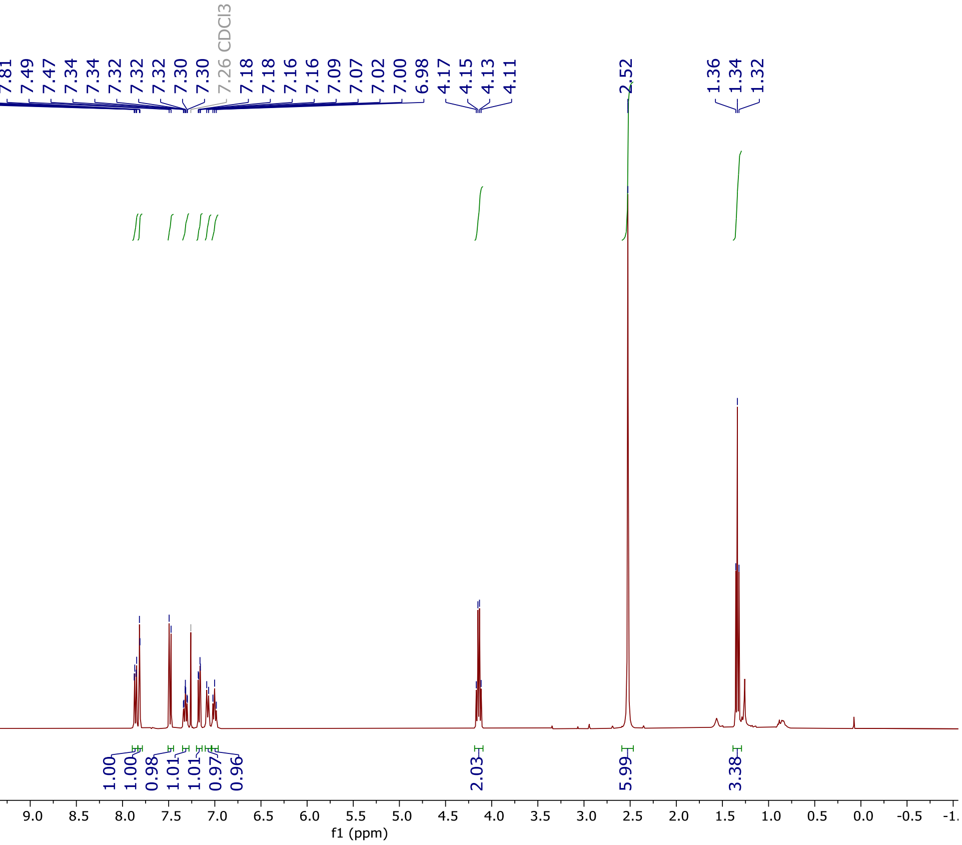

101 MHz, CDCl3

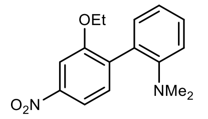

3c

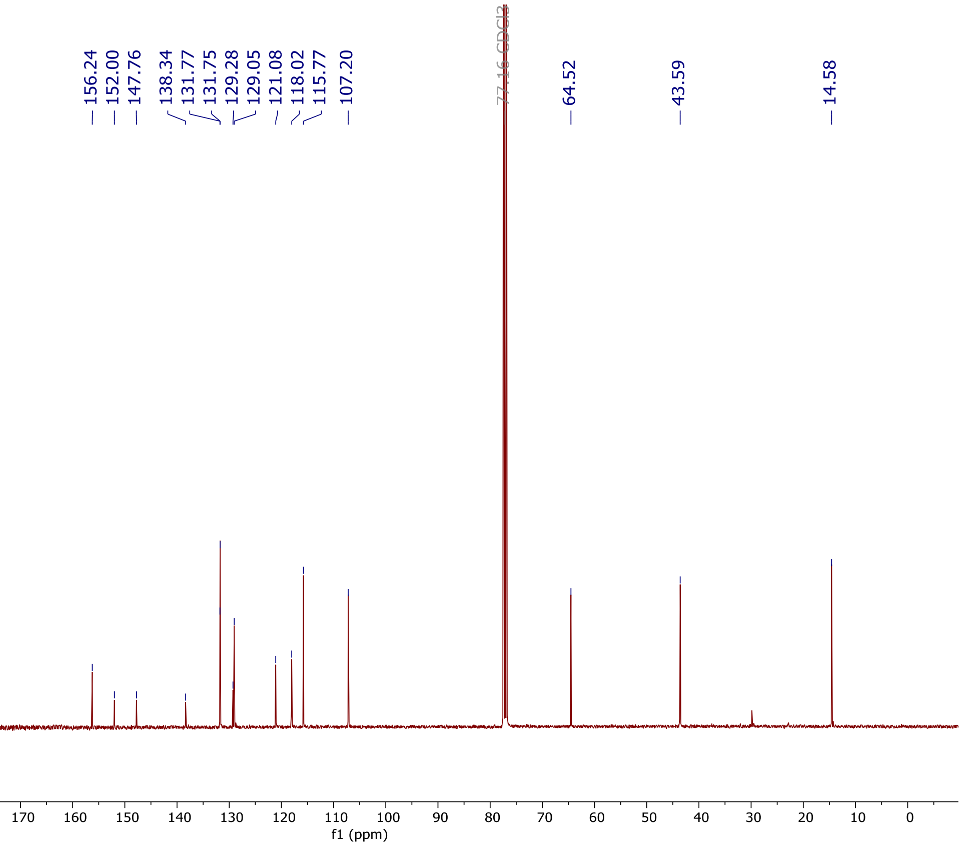

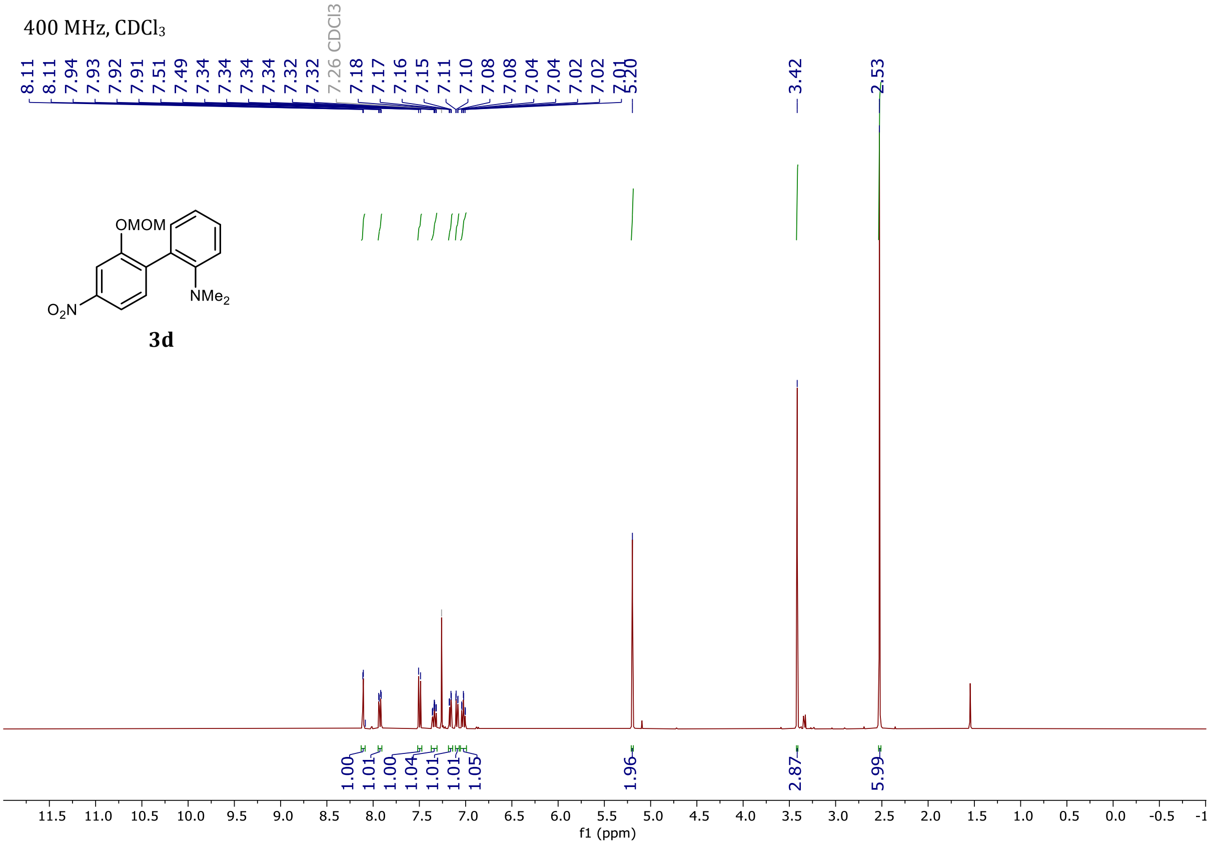
400 MHz, CDCl3
8.11
8.11
7.94
7.93
7.92
7.91
7.51
7.49
7.34
7.34
7.34
7.34
7.32
7.32
7.26 CDCl3
7.18
7.17
7.16
7.15
7.11
7.10
7.08
7.08
7.04
7.04
7.02
7.01
5.20
3.42
2.53
OMOM
O2N
NMe2
3d
1.00
1.01
1.00
1.04
1.01
1.01
1.05
1.96
2.87
5.99
f1 (ppm)

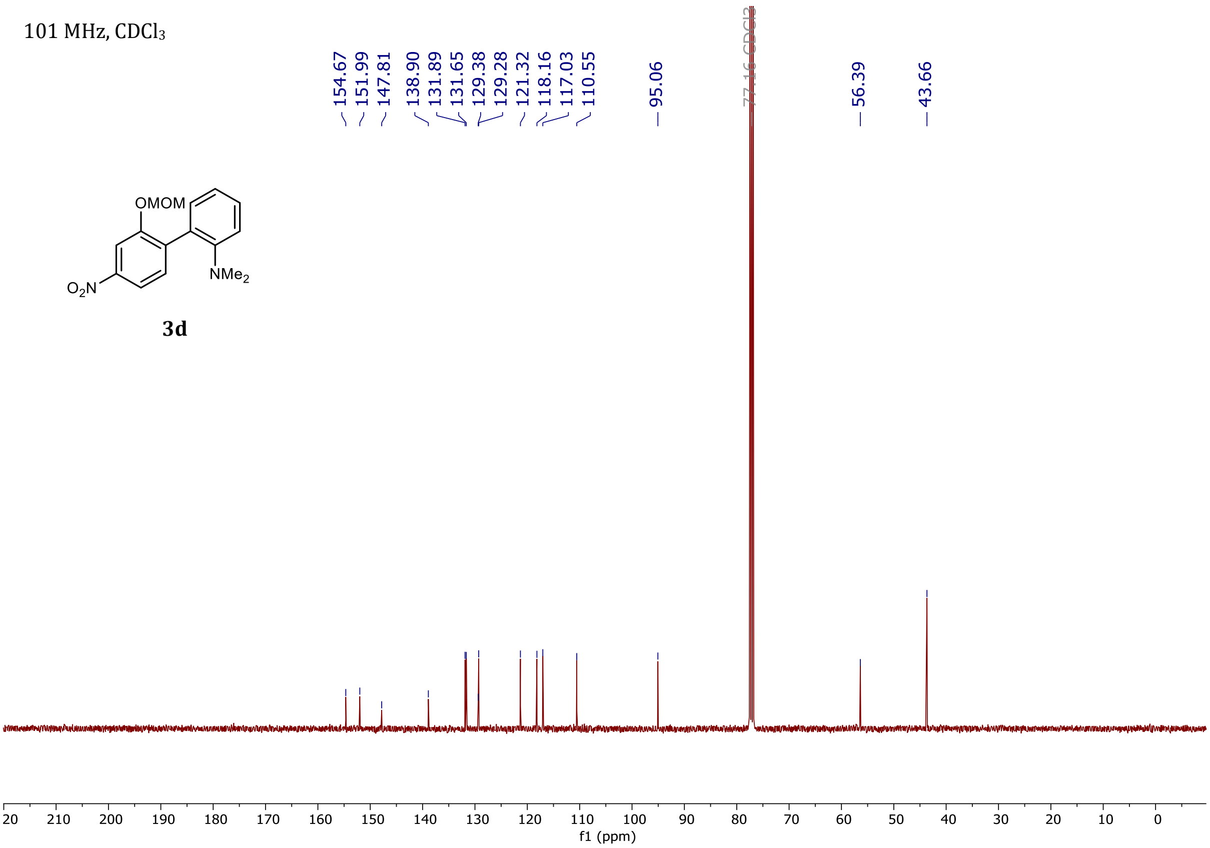
101 MHz, CDCl3
154.67
151.99
147.81
138.90
131.89
131.65
129.38
129.28
121.32
118.16
117.03
110.55
95.06
77.16 CDCl3
56.39
43.66
OMOM
O2N
NMe2
3d
f1 (ppm)

400 MHz, CDCl3

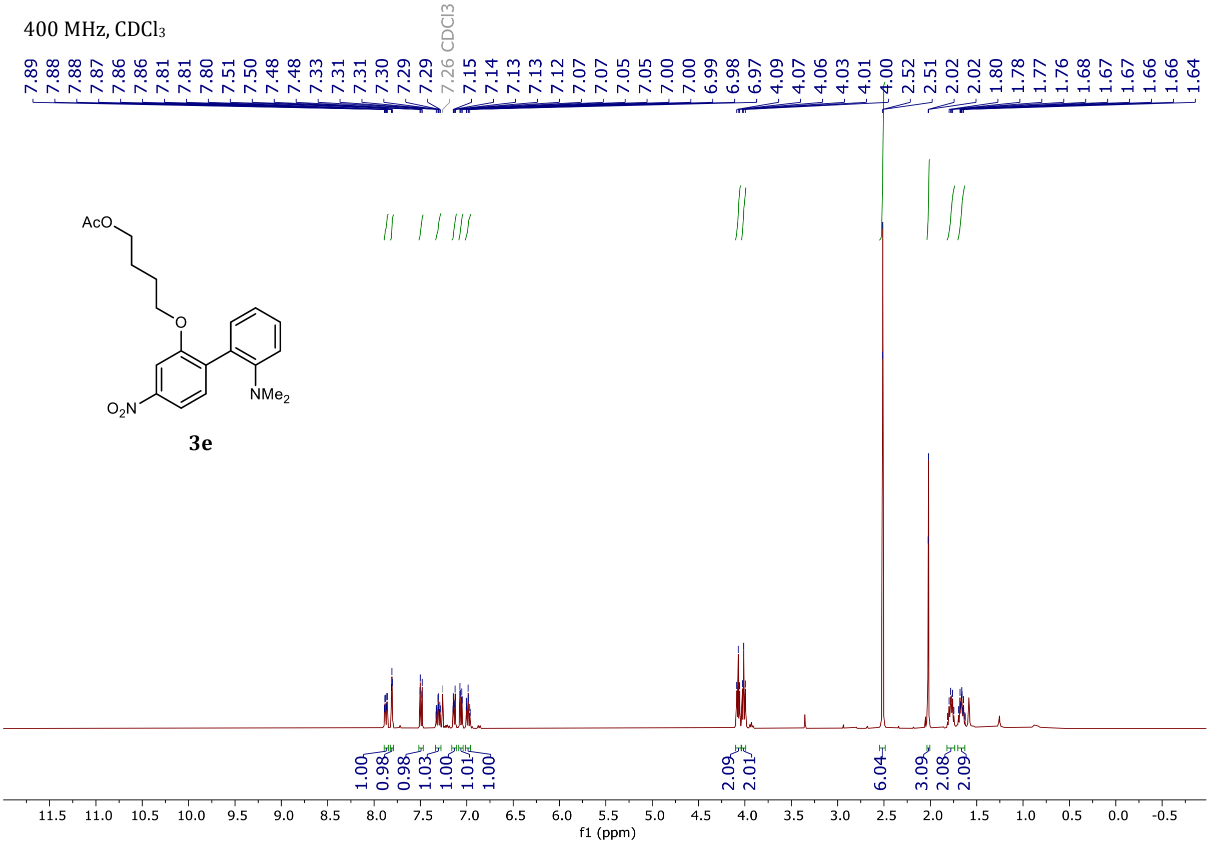

3e

101 MHz, CDCl3

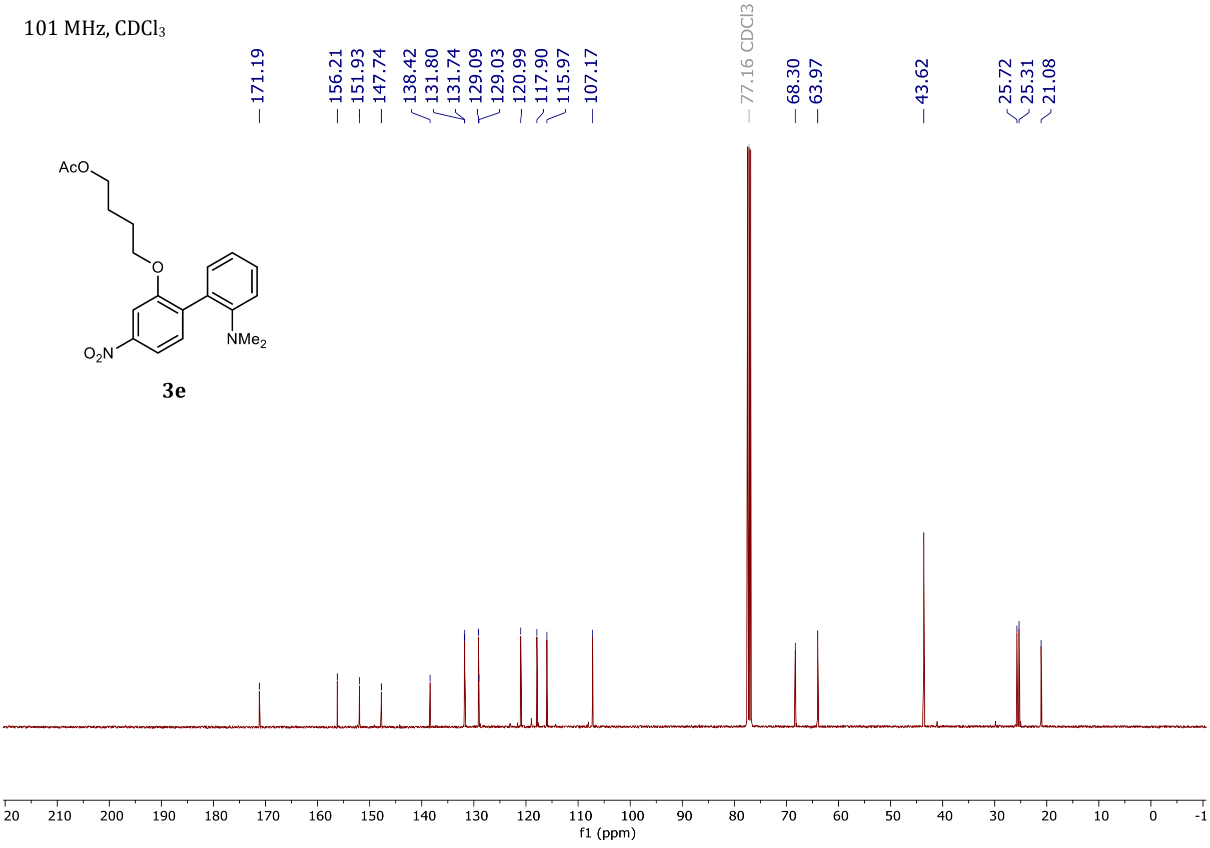

3e

400 MHz, CDCl₃

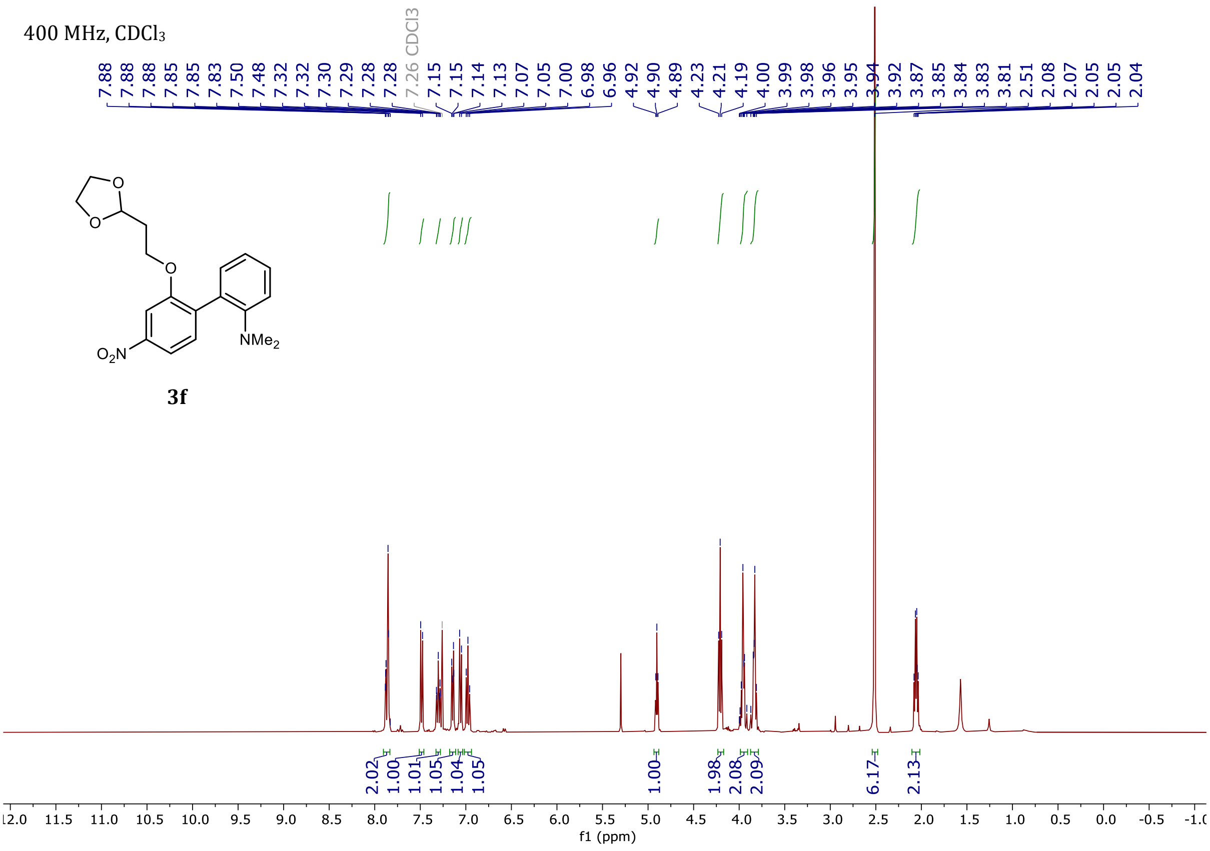

3f

101 MHz, CDCl₃

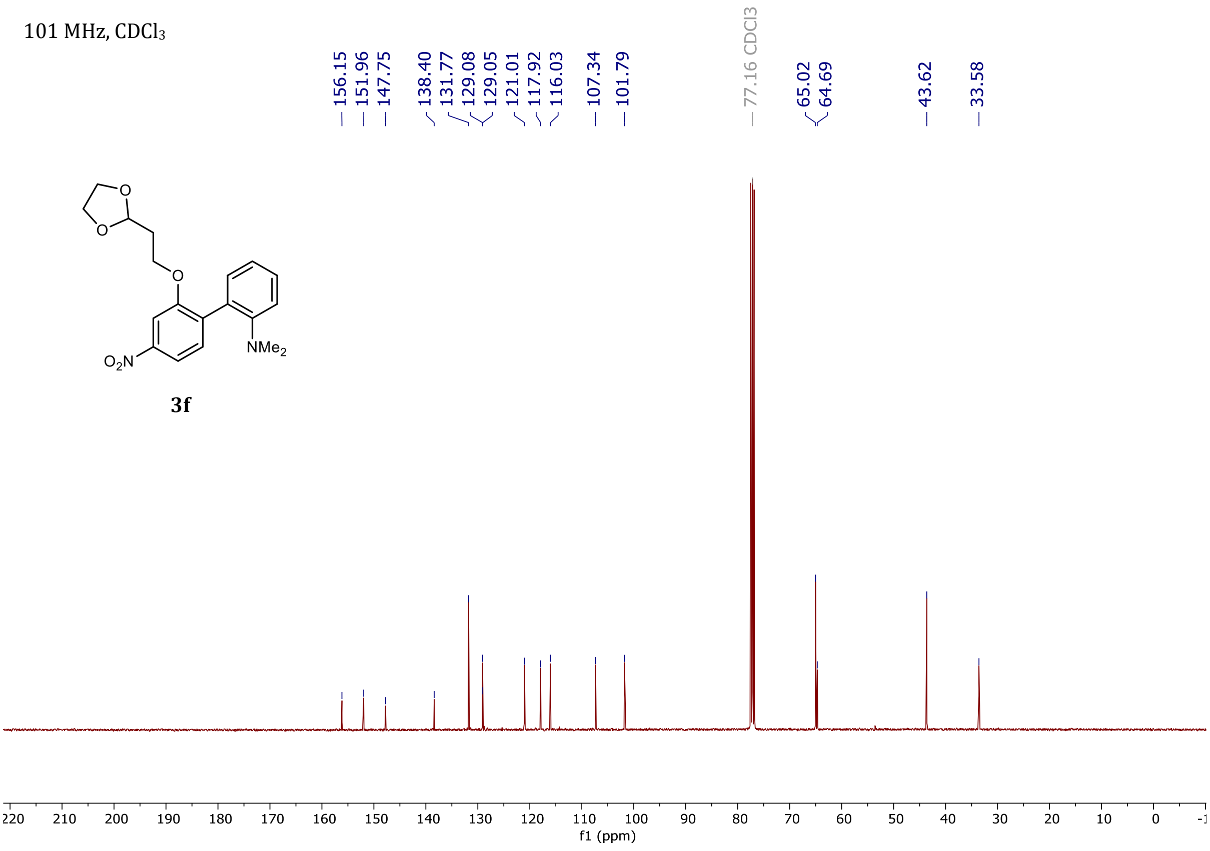

3f

500 MHz, $CDCl_3$

3g

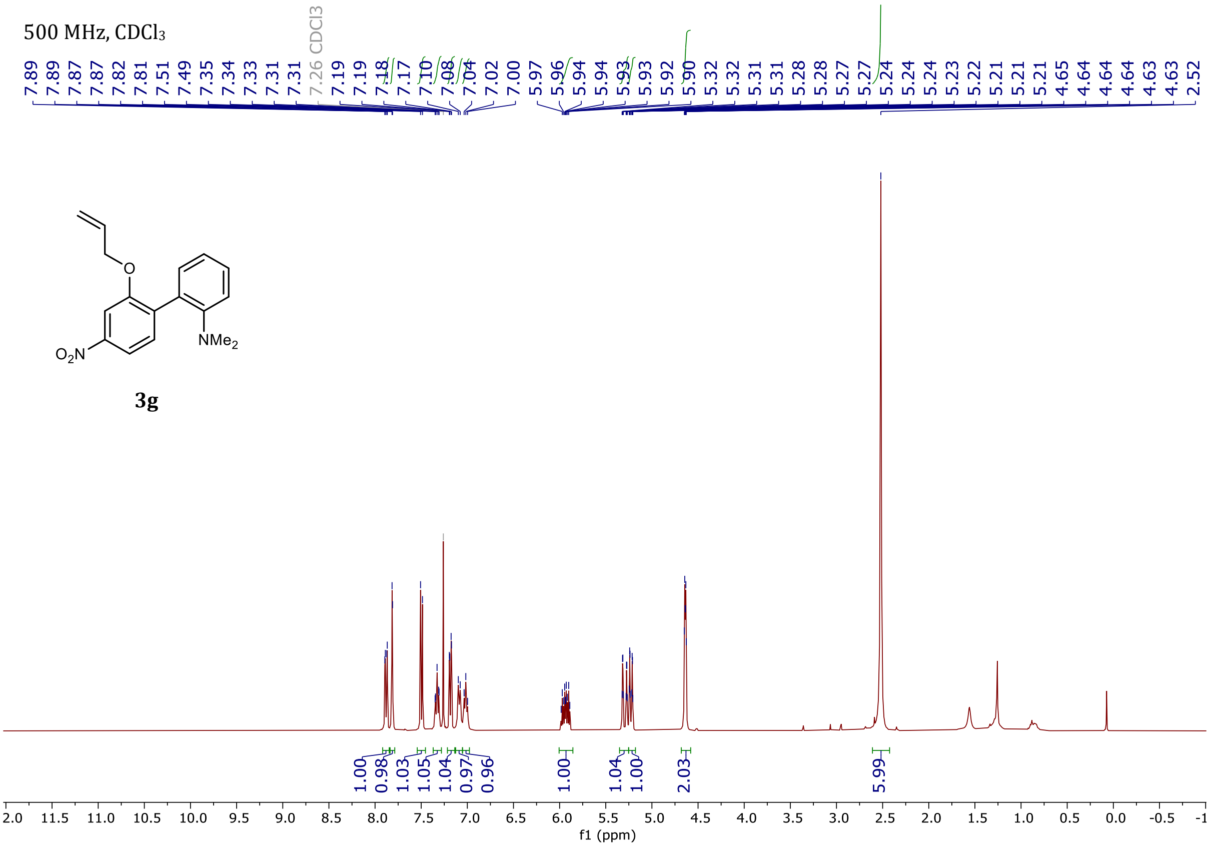

126 MHz, $CDCl_3$

3g

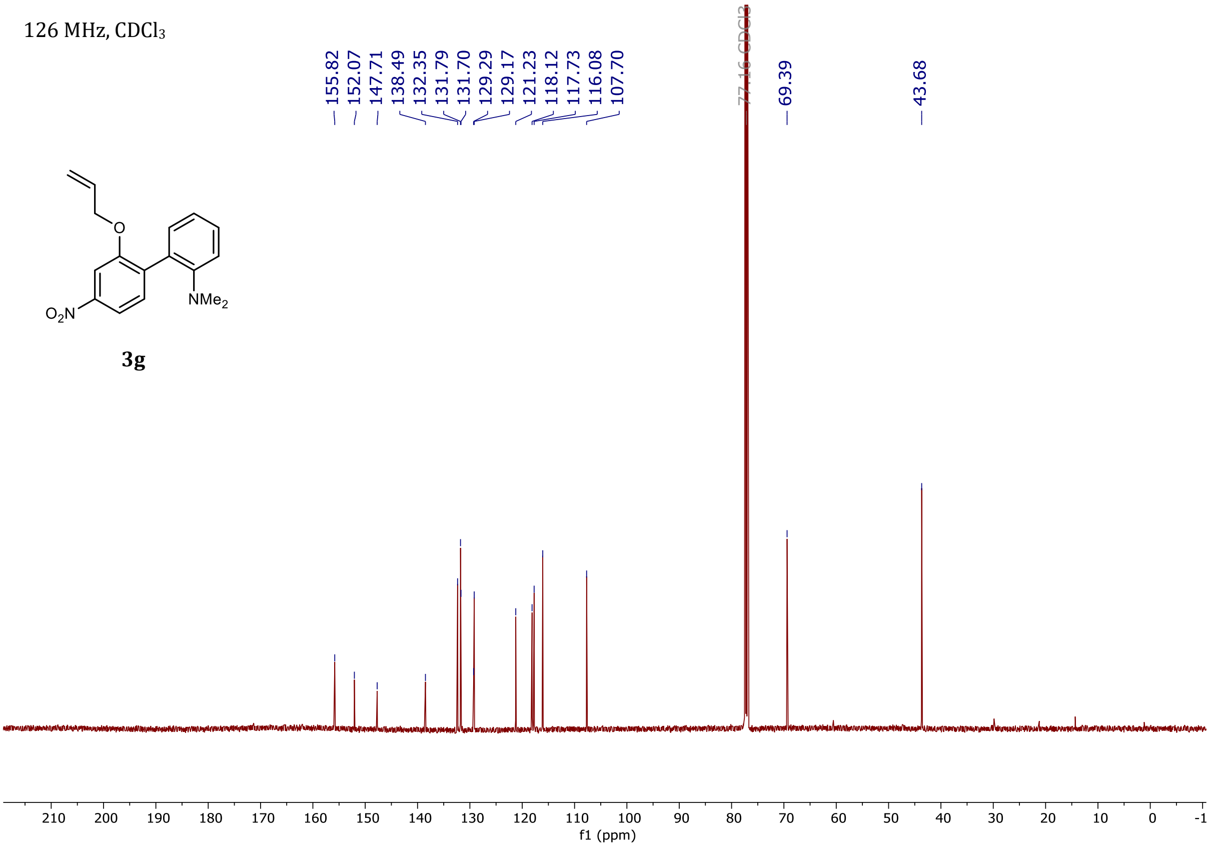

400 MHz, $CDCl_3$

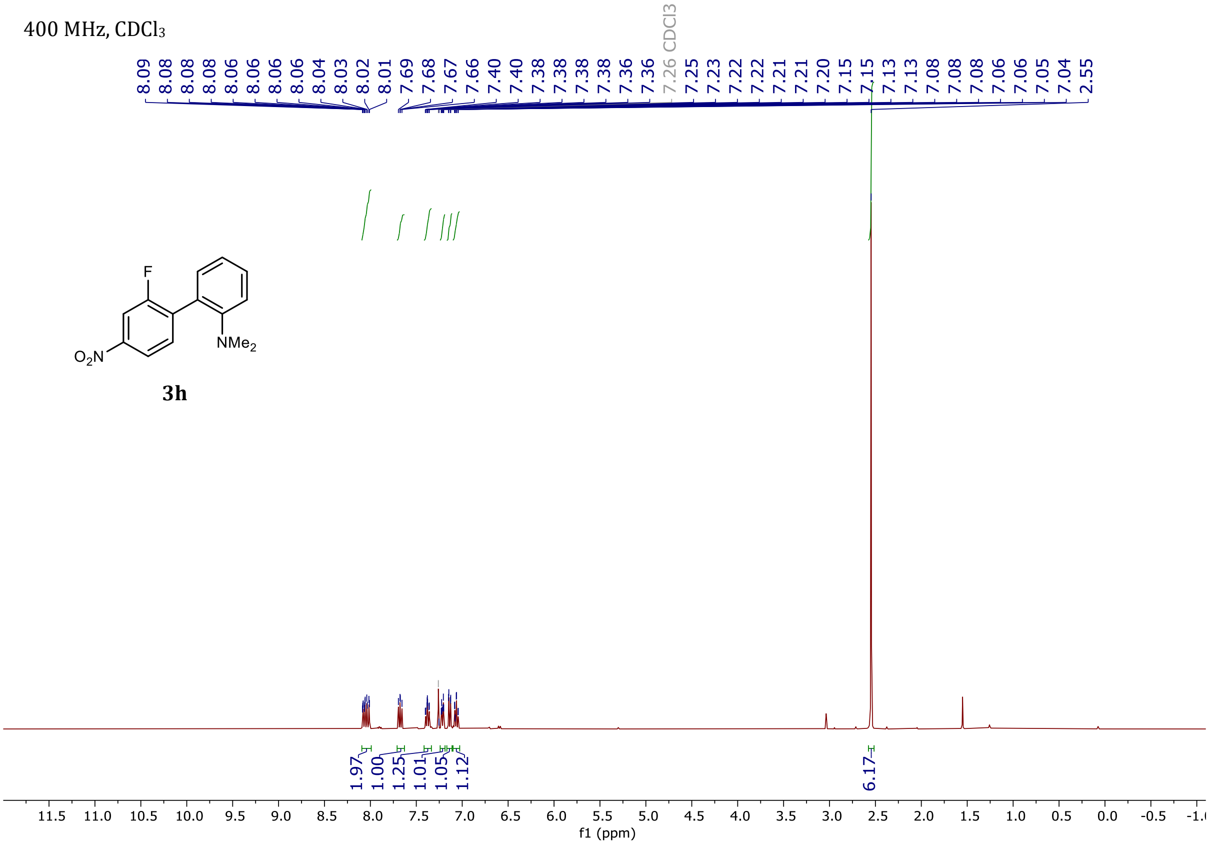

3h

101 MHz, $CDCl_3$

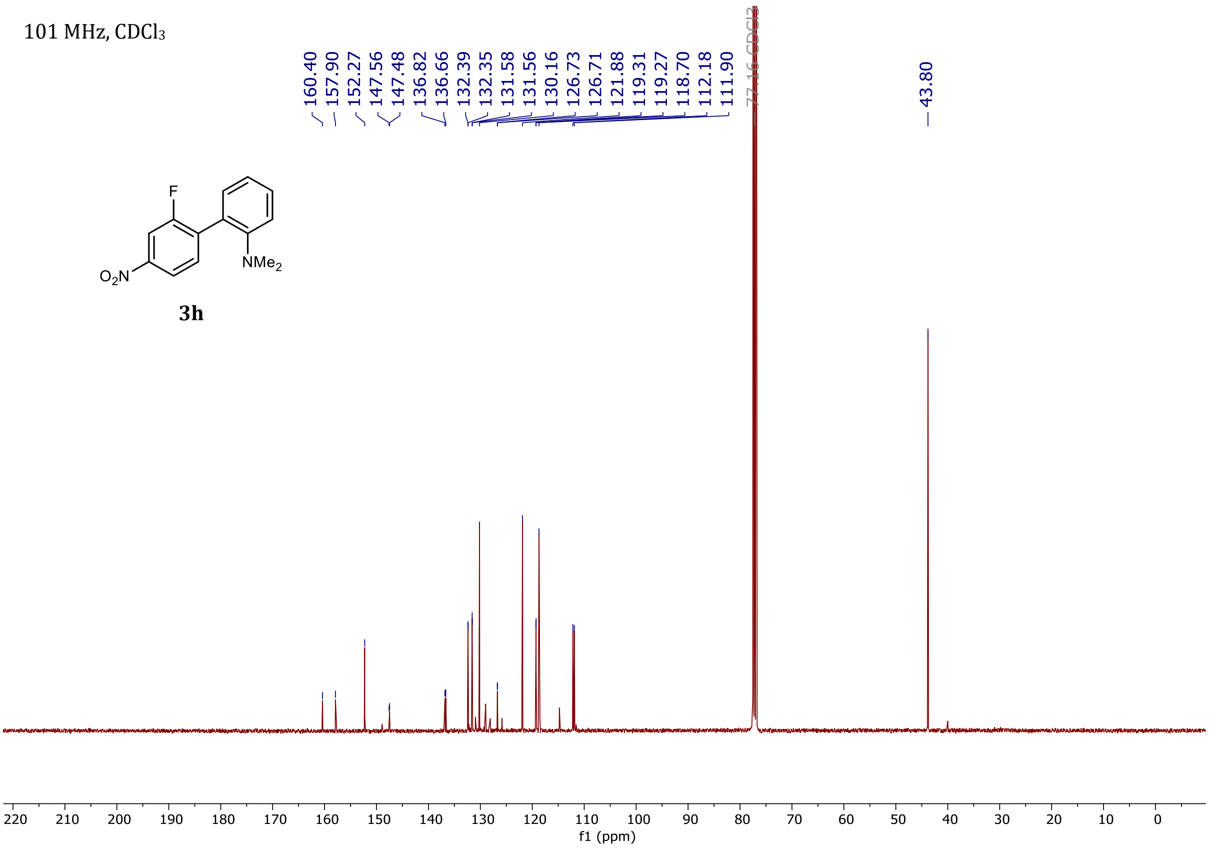

3h

376 MHz, CDCl3

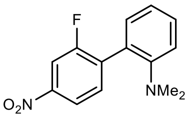

3h

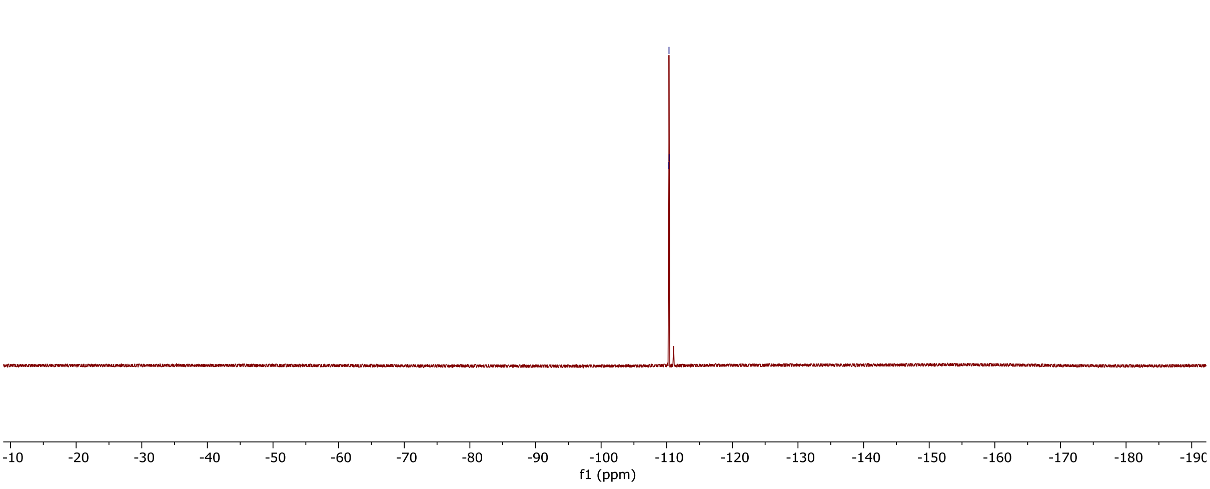

400 MHz, CDCl3

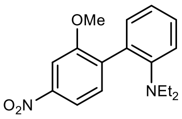

3i

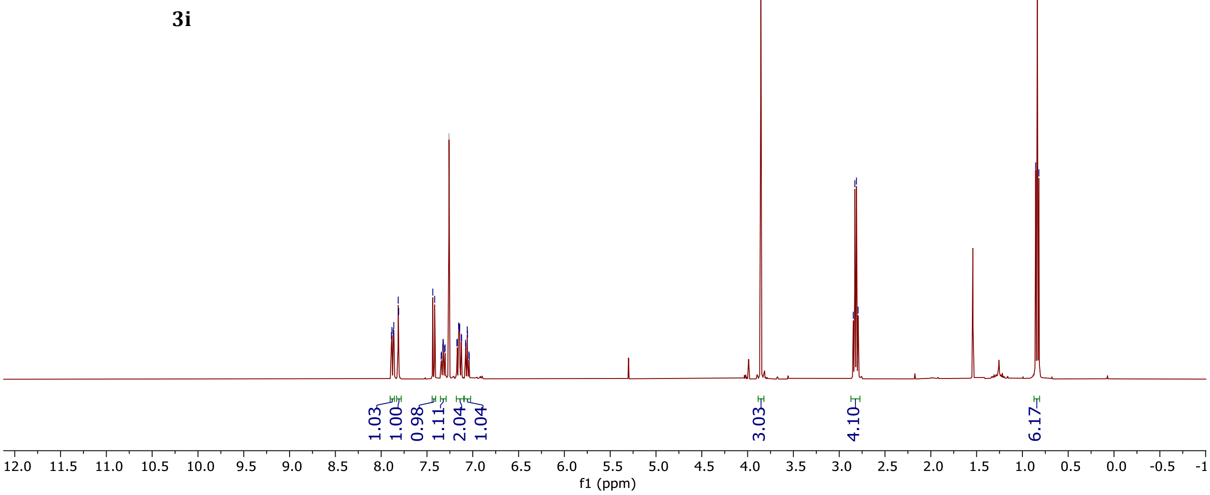

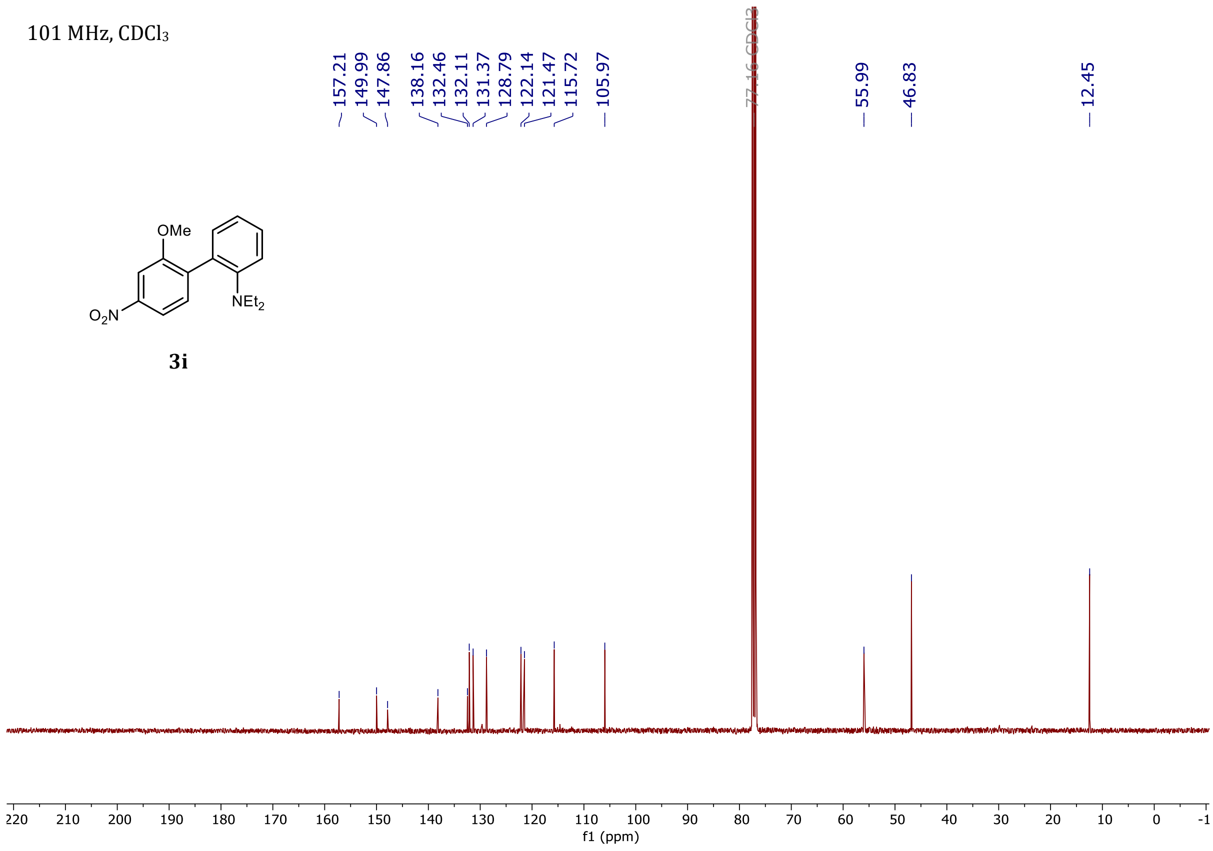
101 MHz, CDCl3
157.21
149.99
147.86
138.16
132.46
132.11
131.37
128.79
122.14
121.47
115.72
105.97
77.16 CDCl3
55.99
46.83
12.45
OMe
NEt2
O2N
3i
f1 (ppm)

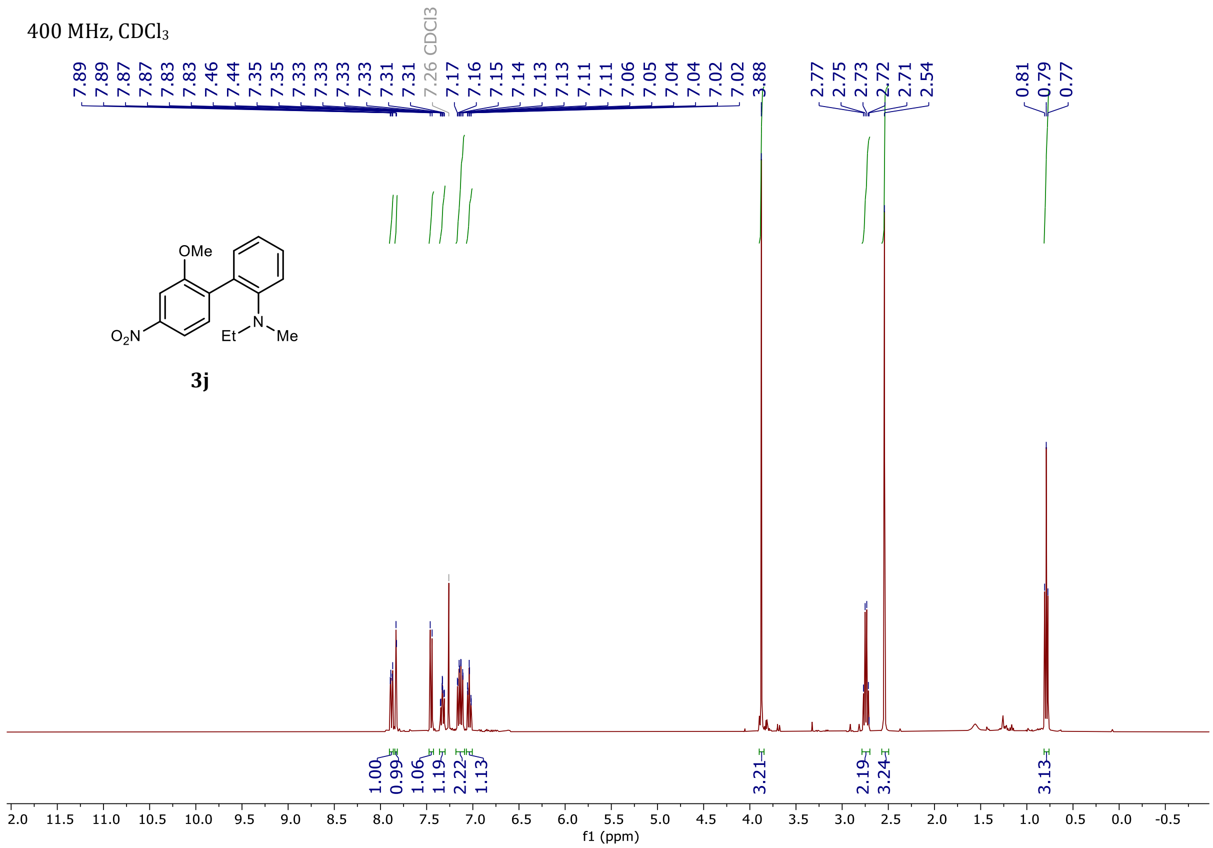
400 MHz, CDCl3
7.89
7.89
7.87
7.87
7.83
7.83
7.46
7.44
7.35
7.35
7.33
7.33
7.33
7.33
7.31
7.31
7.26 CDCl3
7.17
7.16
7.15
7.14
7.13
7.13
7.11
7.11
7.06
7.05
7.04
7.04
7.02
7.02
3.88
2.77
2.75
2.73
2.72
2.71
2.54
0.81
0.79
0.77
OMe
O2N
Et
N
Me
3j
1.00
0.99
1.06
1.19
2.22
1.13
3.21
2.19
3.24
3.13
f1 (ppm)

101 MHz, $CDCl_3$

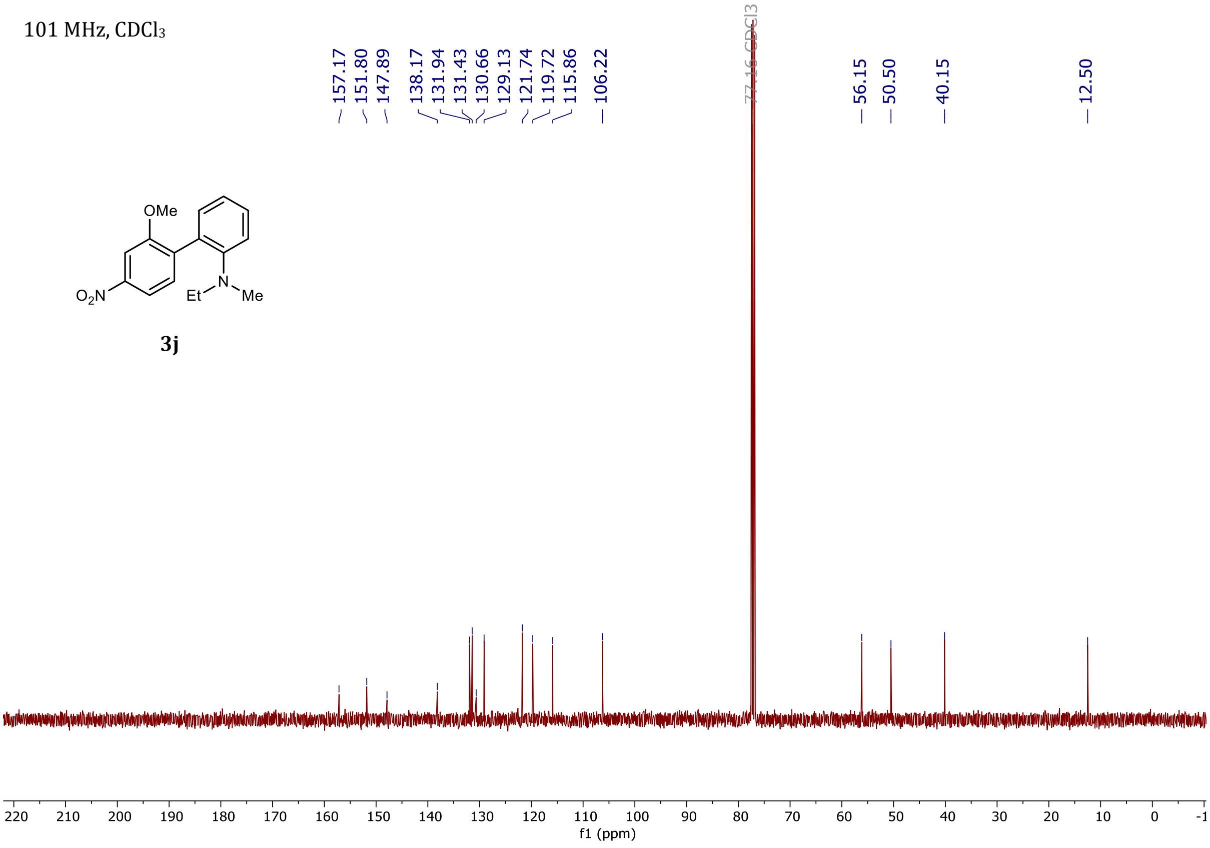

400 MHz, $CDCl_3$

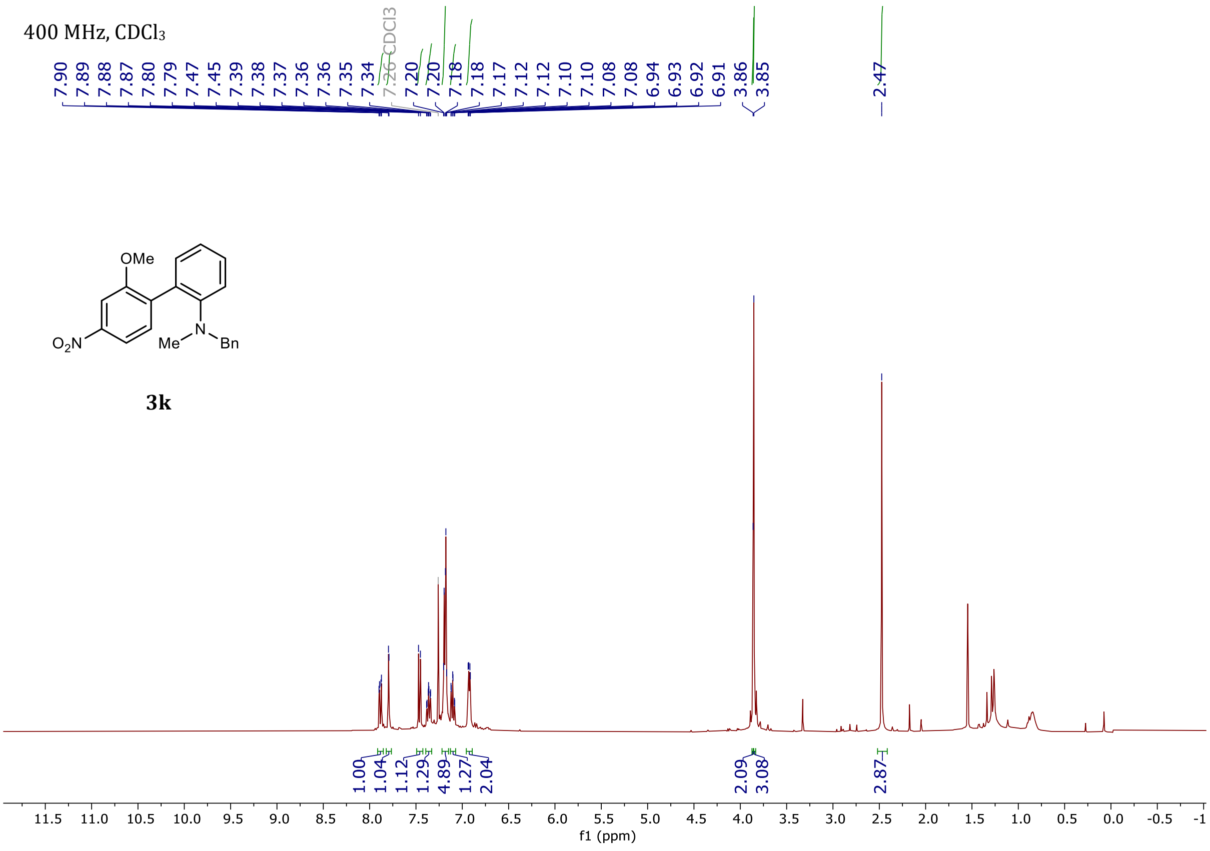

101 MHz, $CDCl_3$

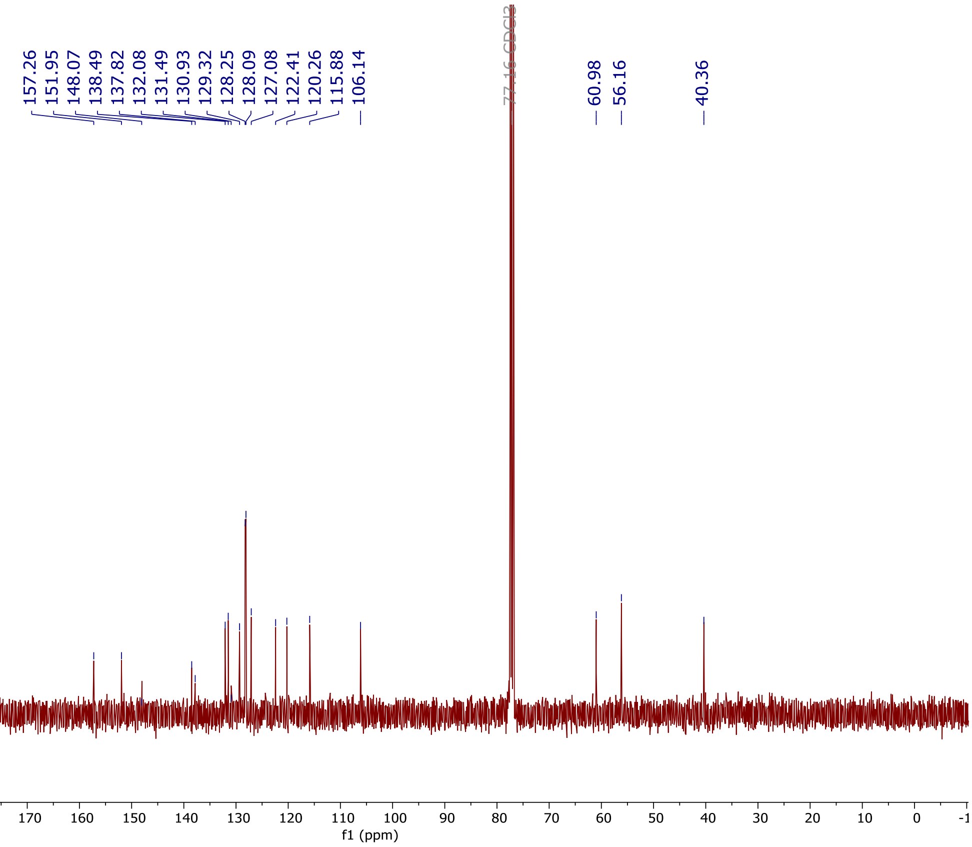

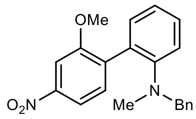

3k

500 MHz, $CDCl_3$

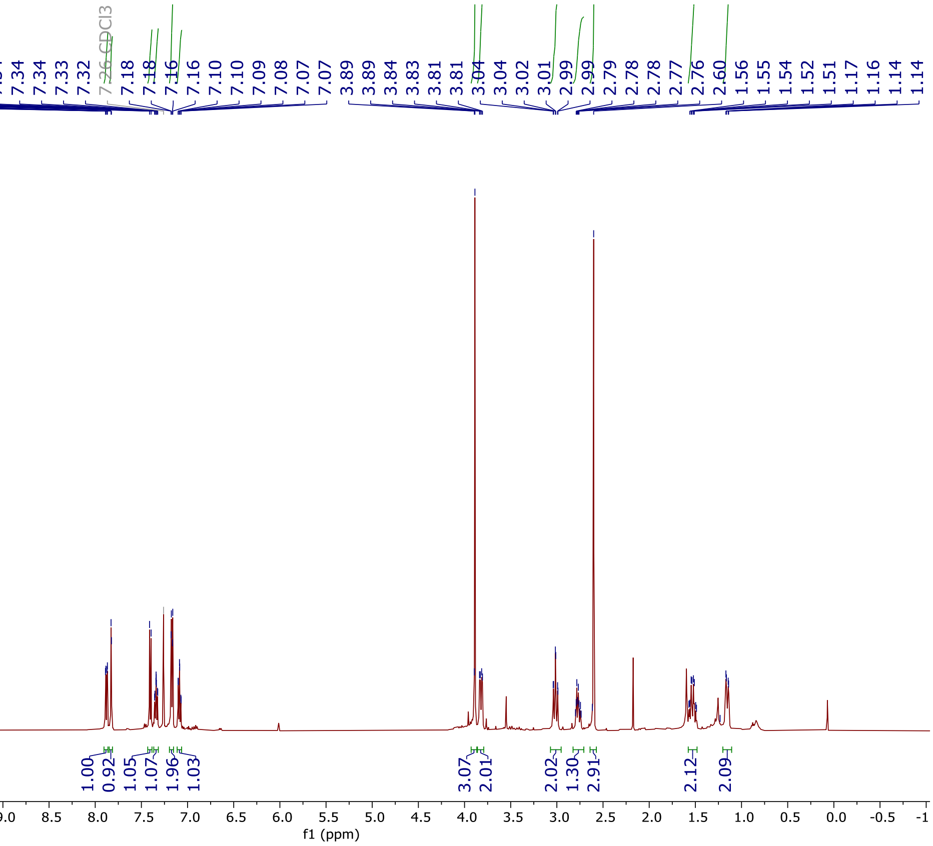

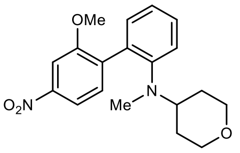

3l

126 MHz, CDCl3

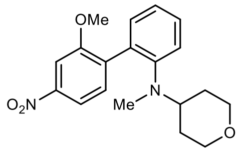

3l

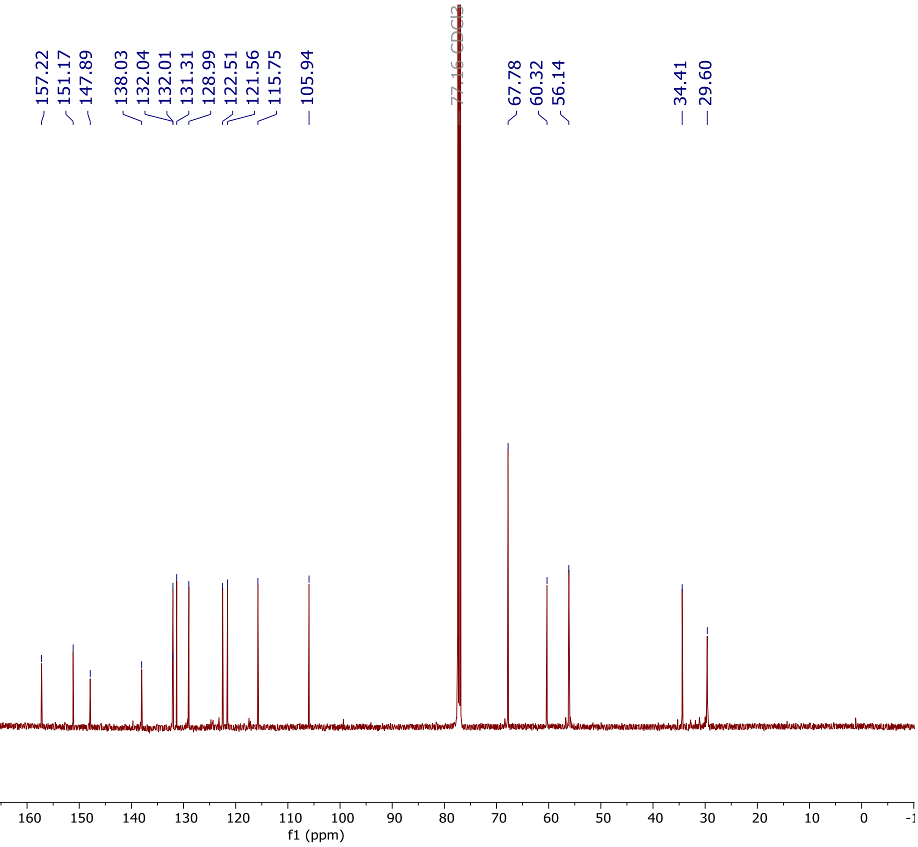

500 MHz, CDCl3

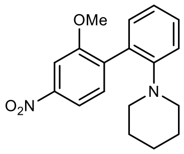

3m

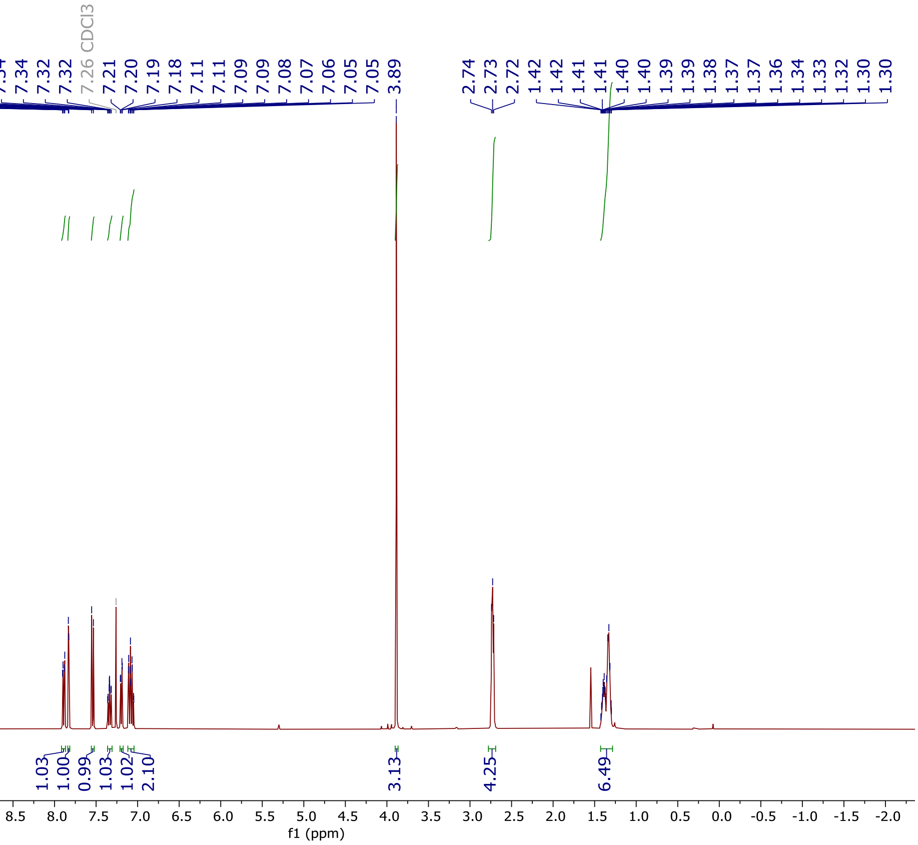

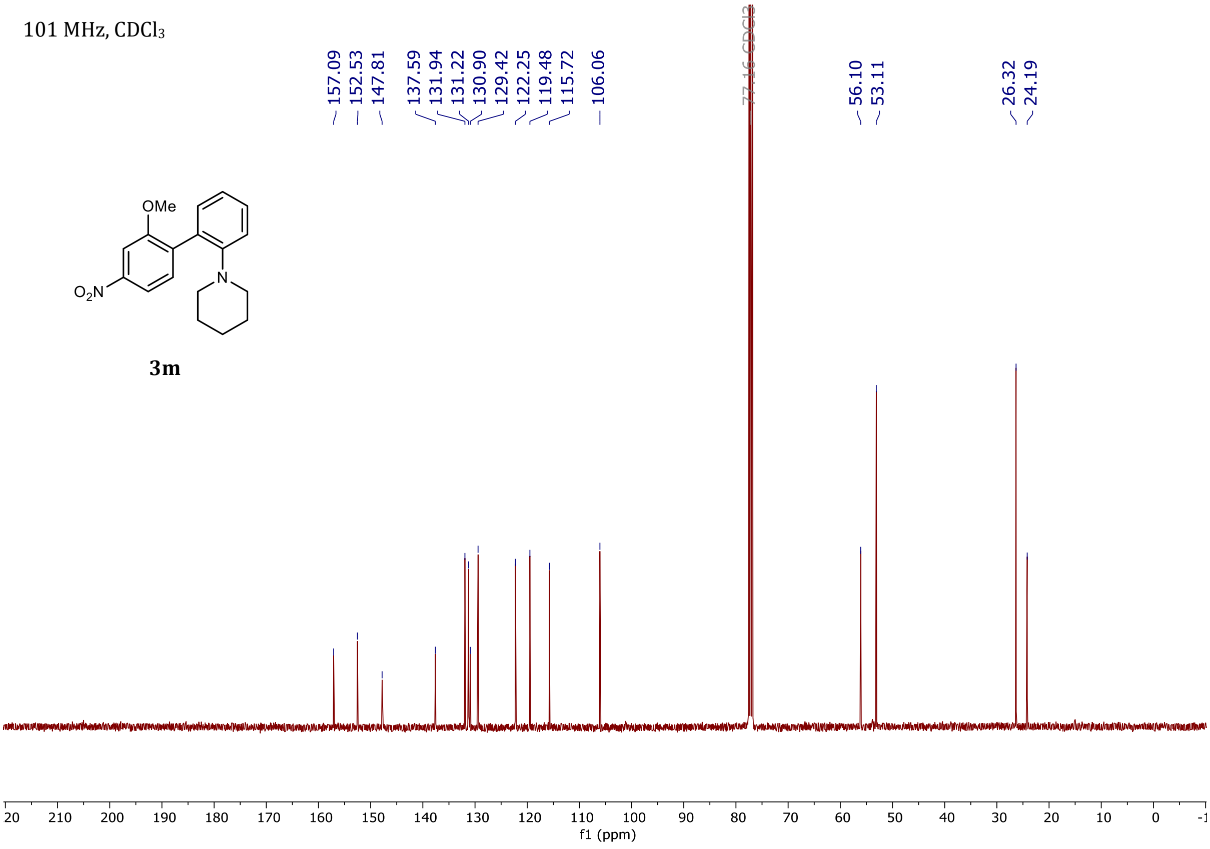
101 MHz, CDCl3
157.09
152.53
147.81
137.59
131.94
131.22
130.90
129.42
122.25
119.48
115.72
106.06
77.16 CDCl3
56.10
53.11
26.32
24.19
OMe
O2N
N
f1 (ppm)

3m

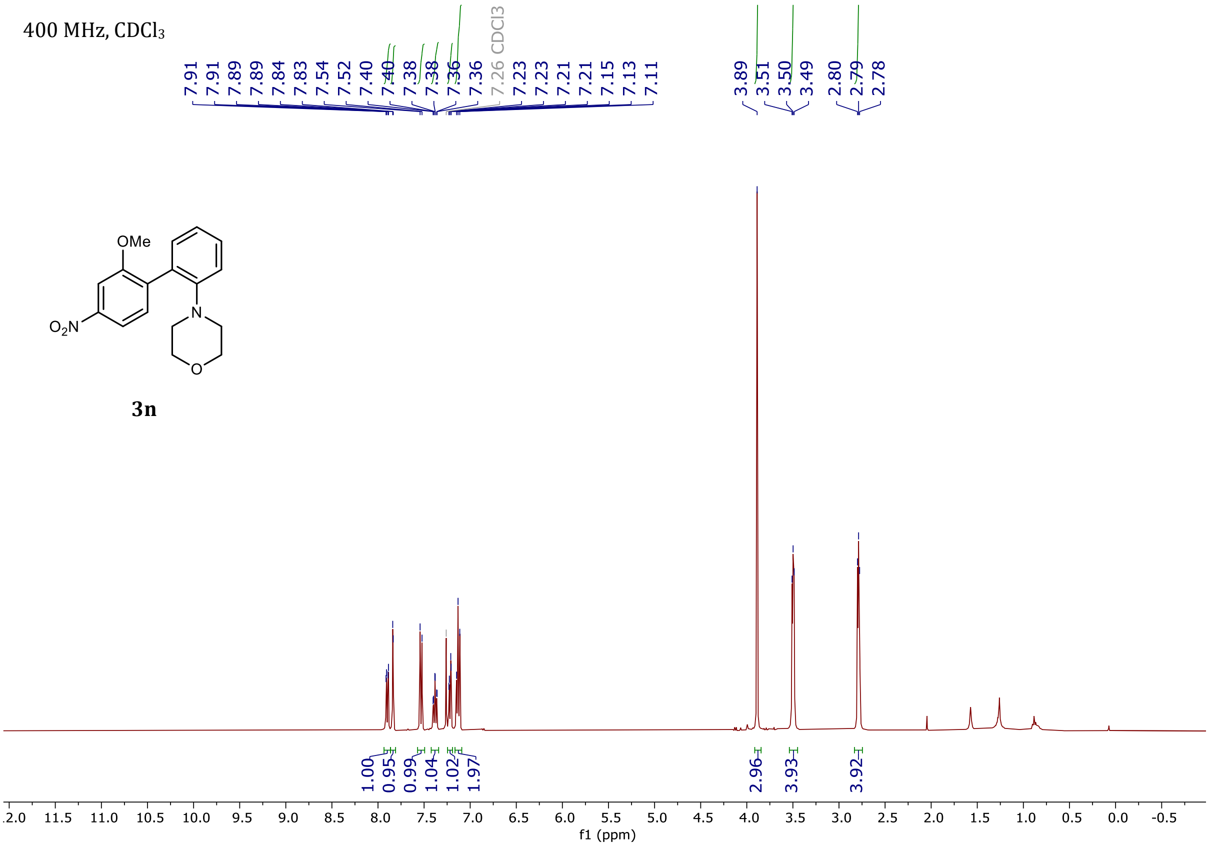
400 MHz, CDCl3
7.91
7.91
7.89
7.89
7.84
7.83
7.54
7.52
7.40
7.40
7.38
7.38
7.36
7.36
7.26 CDCl3
7.23
7.23
7.21
7.21
7.15
7.13
7.11
3.89
3.51
3.50
3.49
2.80
2.79
2.78
OMe
O2N
N
O
1.00
0.95
0.99
1.04
1.02
1.97
2.96
3.93
3.92
f1 (ppm)

3n

101 MHz, $CDCl_3$

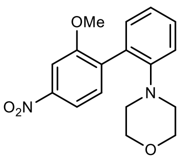

3n

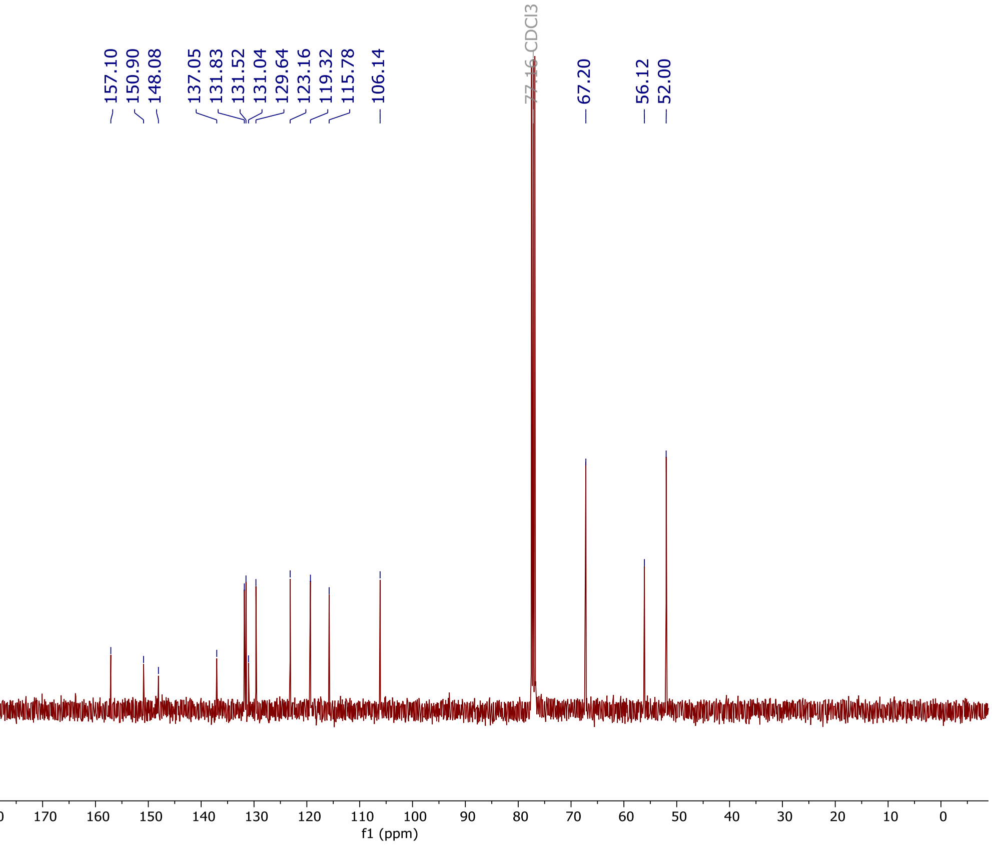

400 MHz, $CDCl_3$

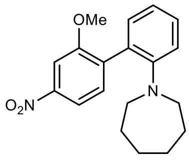

3o

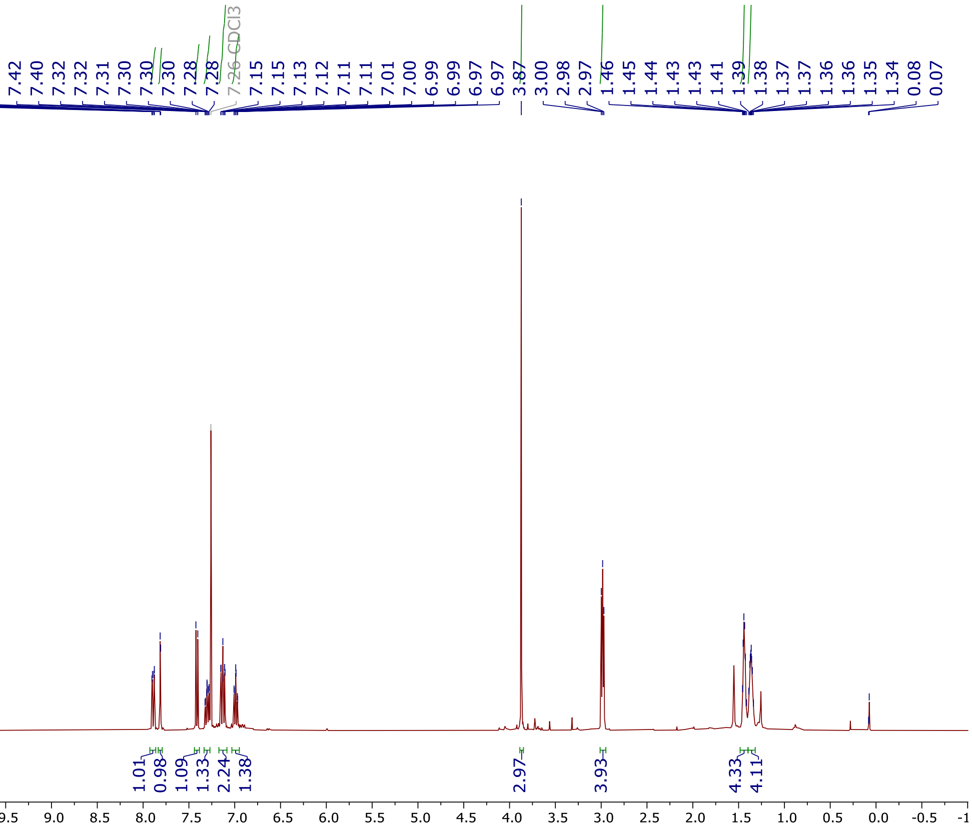

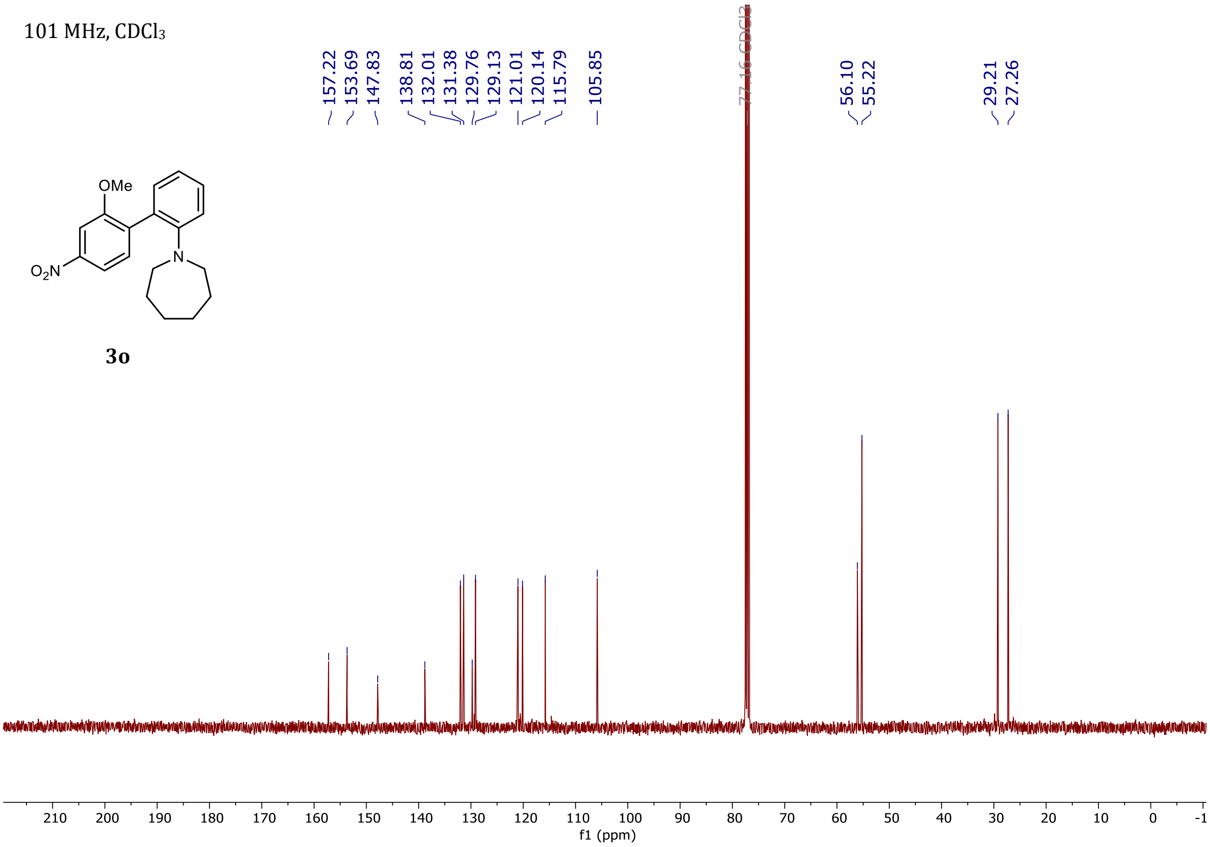
101 MHz, CDCl3
157.22
153.69
147.83
138.81
132.01
131.38
129.76
129.13
121.01
120.14
115.79
105.85
77.16 CDCl3
56.10
55.22
29.21
27.26
OMe
O2N
N
210 200 190 180 170 160 150 140 130 120 110 100 90 80 70 60 50 40 30 20 10 0 -1
f1 (ppm)

3o

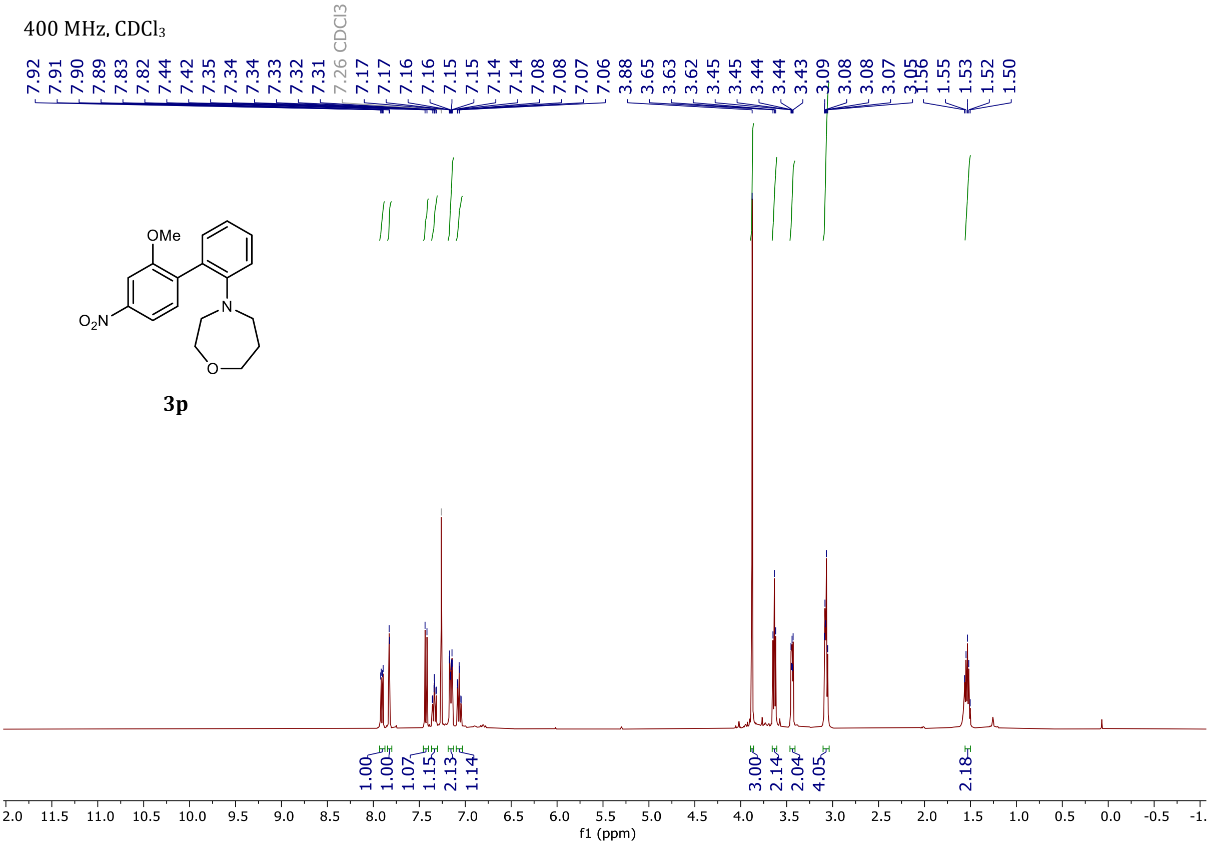
400 MHz, CDCl3
7.92
7.91
7.90
7.89
7.83
7.82
7.44
7.42
7.35
7.34
7.34
7.33
7.32
7.31
7.26 CDCl3
7.17
7.17
7.16
7.16
7.15
7.15
7.14
7.08
7.08
7.07
7.06
3.88
3.65
3.63
3.62
3.45
3.45
3.44
3.44
3.43
3.09
3.08
3.08
3.07
3.05
1.56
1.55
1.53
1.52
1.50
OMe
O2N
N
O
1.00
1.00
1.07
1.15
2.13
1.14
3.00
2.14
2.04
4.05
2.18
2.0 11.5 11.0 10.5 10.0 9.5 9.0 8.5 8.0 7.5 7.0 6.5 6.0 5.5 5.0 4.5 4.0 3.5 3.0 2.5 2.0 1.5 1.0 0.5 0.0 -0.5 -1.
f1 (ppm)

3p

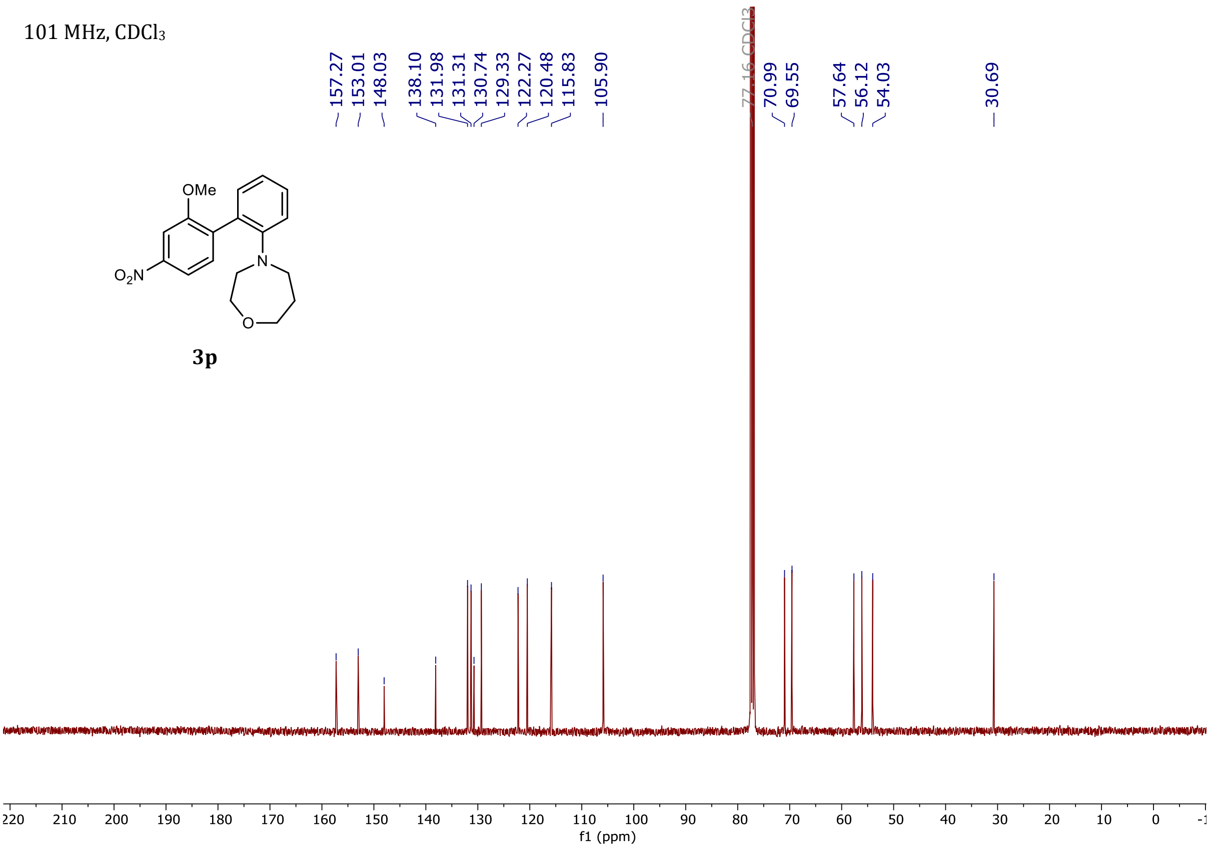
101 MHz, CDCl3
157.27
153.01
148.03
138.10
131.98
131.31
130.74
129.33
122.27
120.48
115.83
105.90
77.16 CDCl3
70.99
69.55
57.64
56.12
54.03
30.69
OMe
O2N
N
O
3p
f1 (ppm)

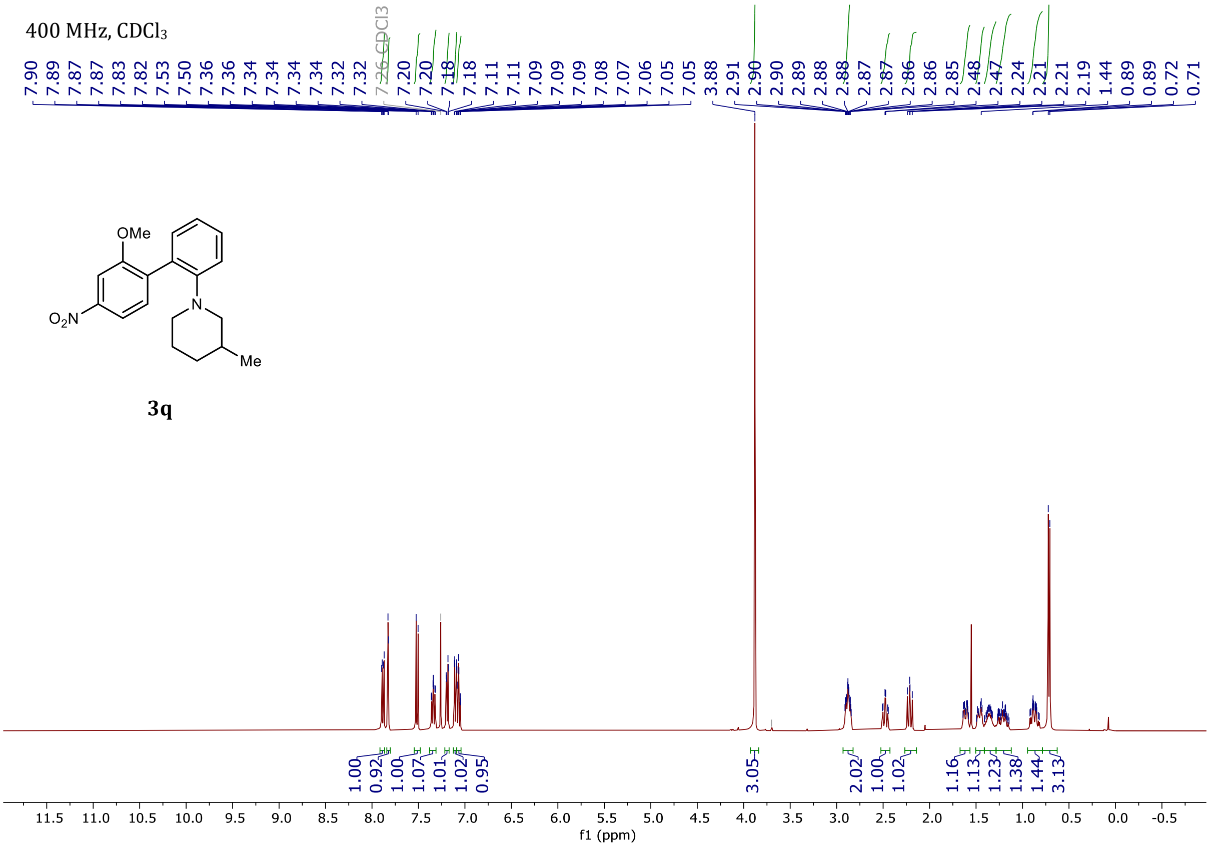
400 MHz, CDCl3
7.90 7.89 7.87 7.87 7.83 7.82 7.53 7.50 7.36 7.36 7.34 7.34 7.34 7.34 7.32 7.32 7.26 CDCl3 7.20 7.20 7.18 7.18 7.11 7.11 7.09 7.09 7.09 7.08 7.07 7.06 7.05 7.05 3.88 2.91 2.90 2.90 2.89 2.88 2.88 2.87 2.87 2.86 2.86 2.85 2.48 2.47 2.24 2.21 2.21 2.19 1.44 0.89 0.72 0.71
OMe
O2N
N
Me
3q
1.00 0.92 1.00 1.07 1.01 1.02 0.95 3.05 2.02 1.00 1.02 1.16 1.13 1.23 1.38 1.44 3.13
f1 (ppm)

101 MHz, $CDCl_3$

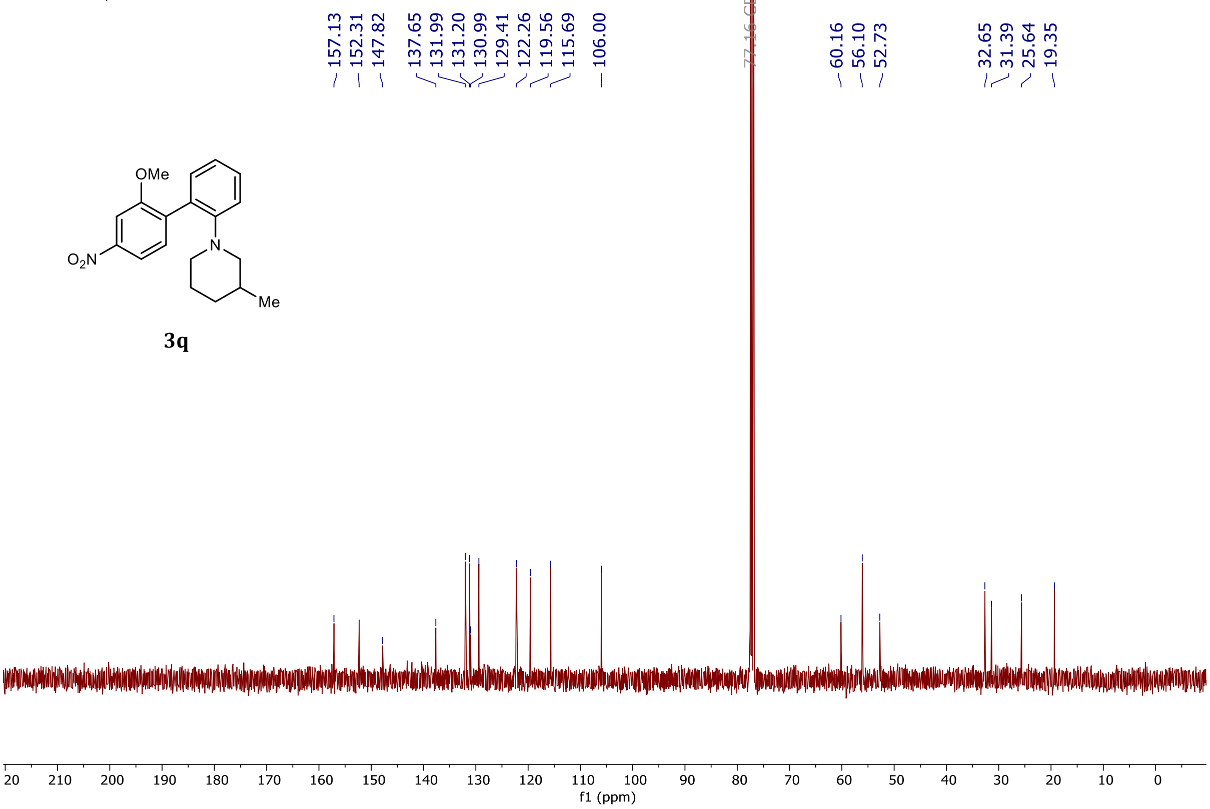

400 MHz, $CDCl_3$

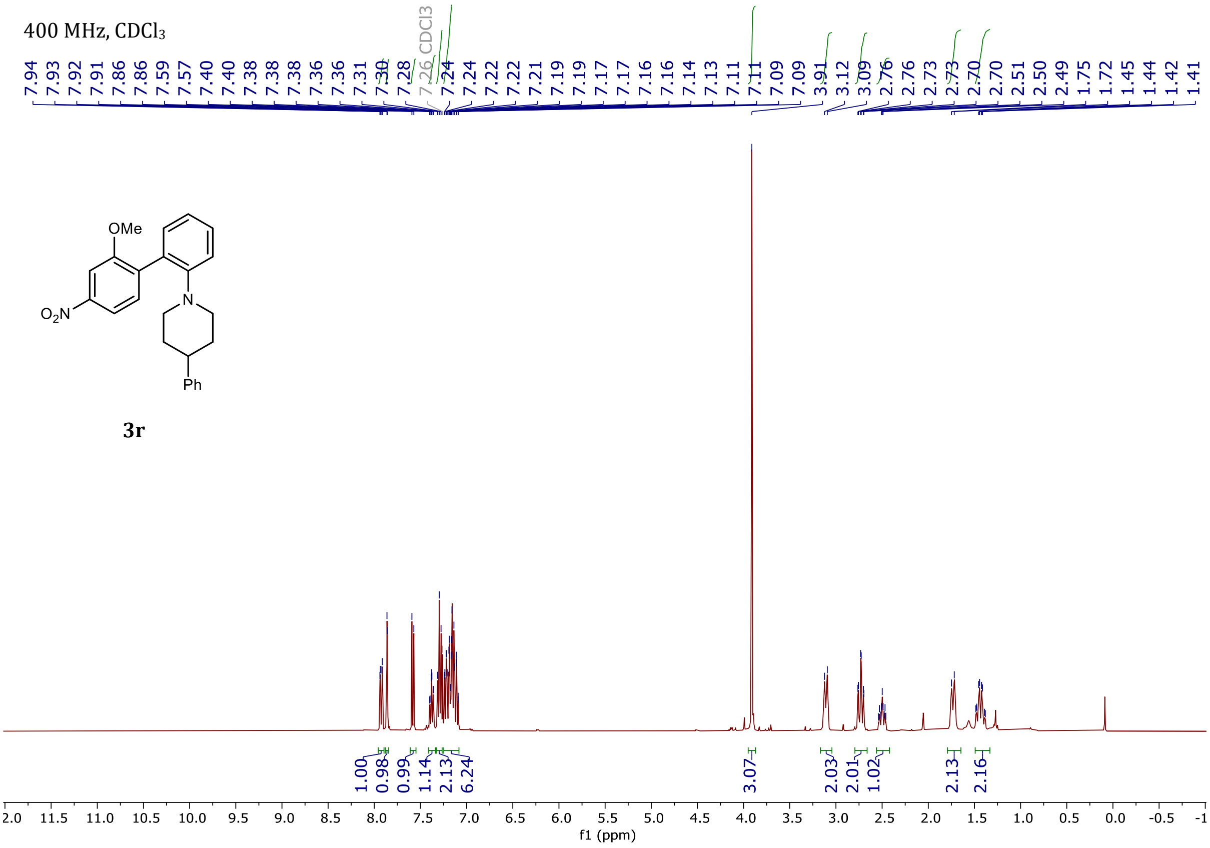

101 MHz, $CDCl_3$

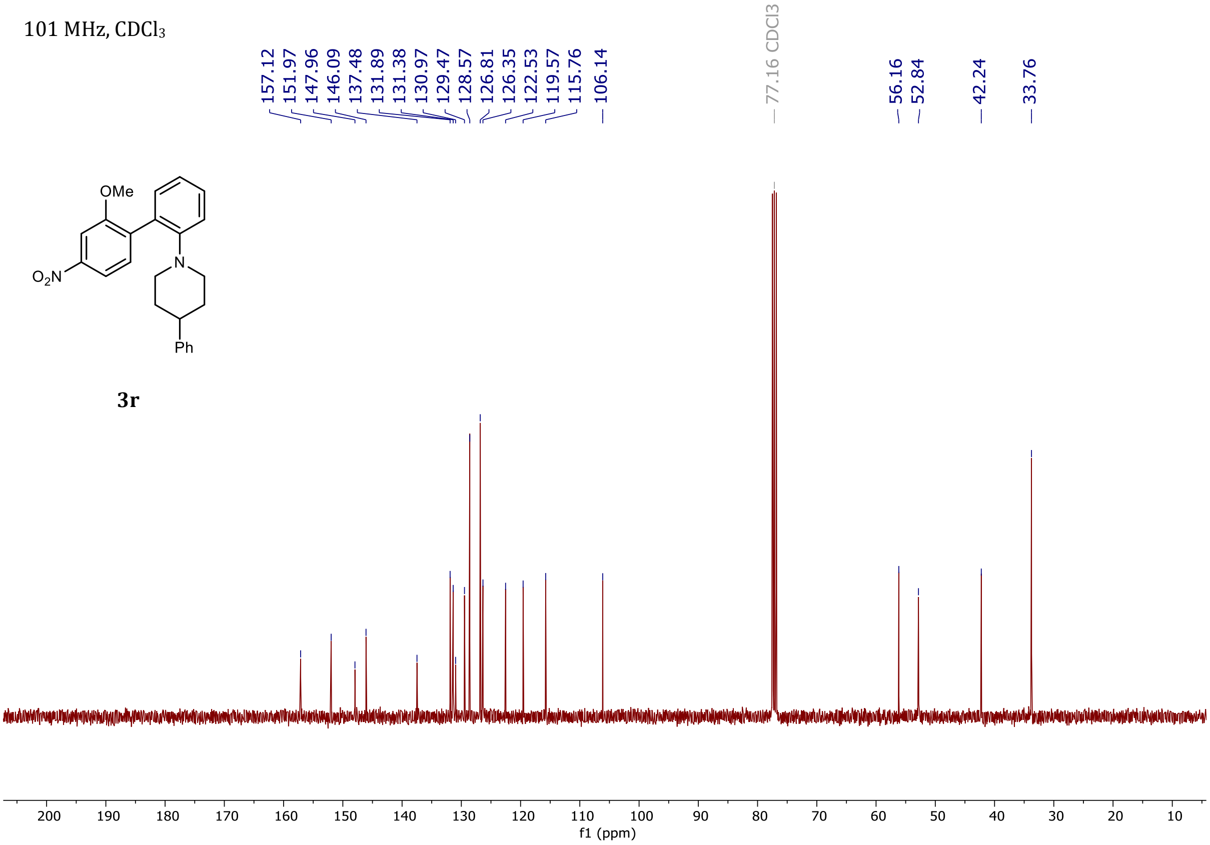

3r

500 MHz, $CDCl_3$

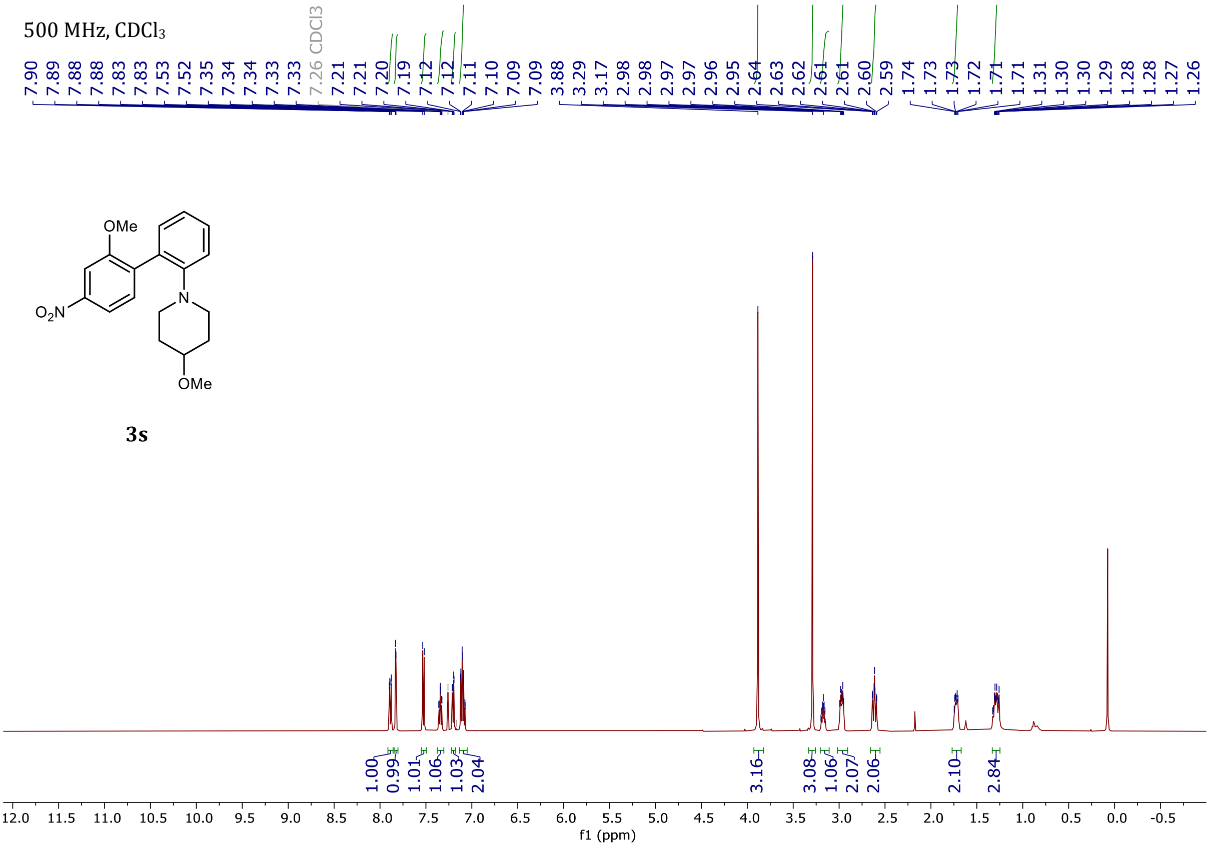

3s

156 MHz, CDCl₃

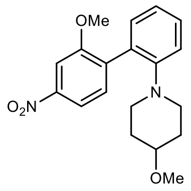

3s

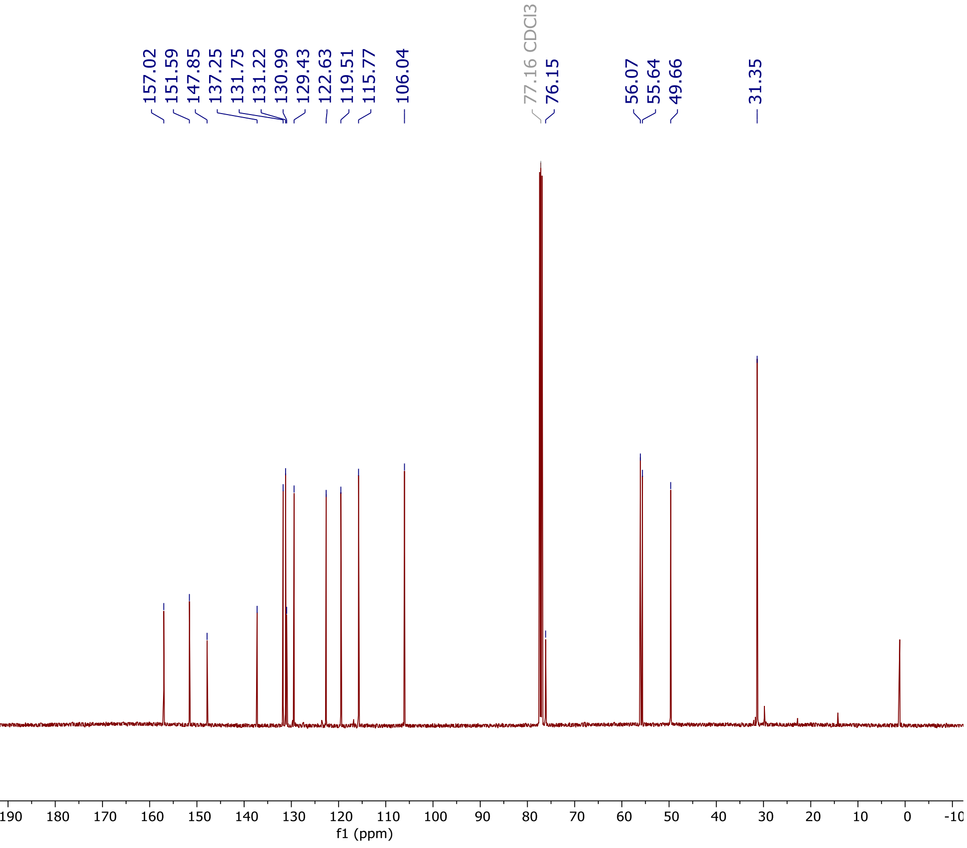

400 MHz, CDCl₃

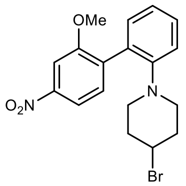

3t

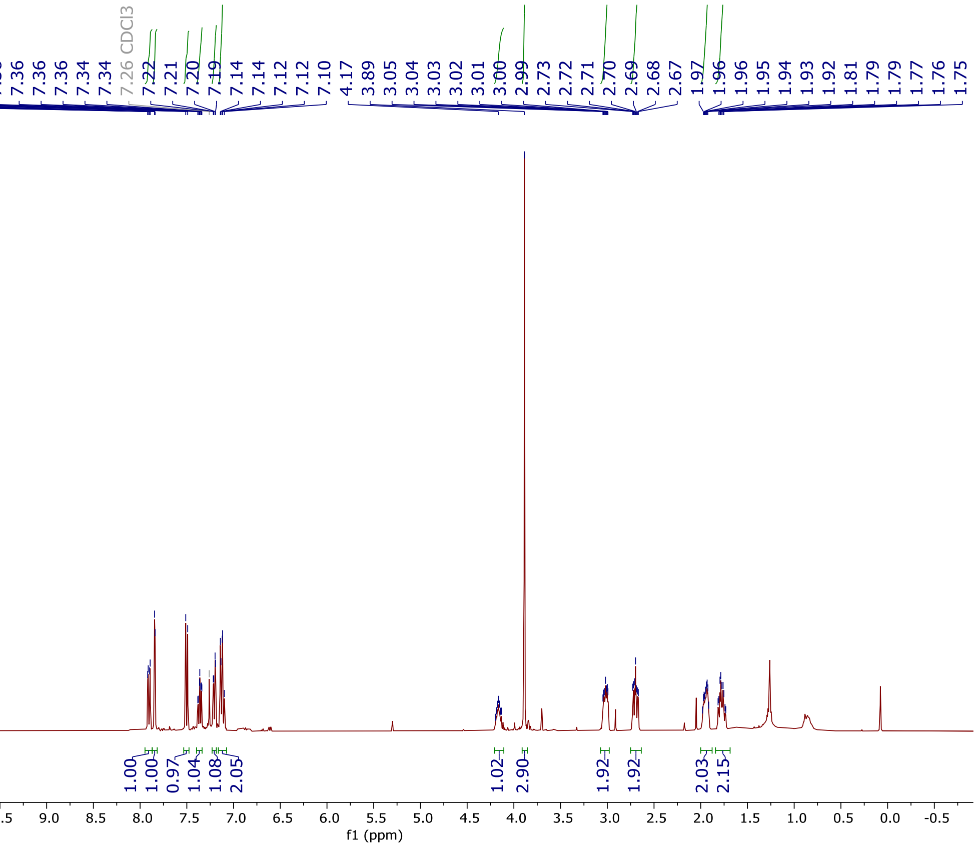

101 MHz, $CDCl_3$

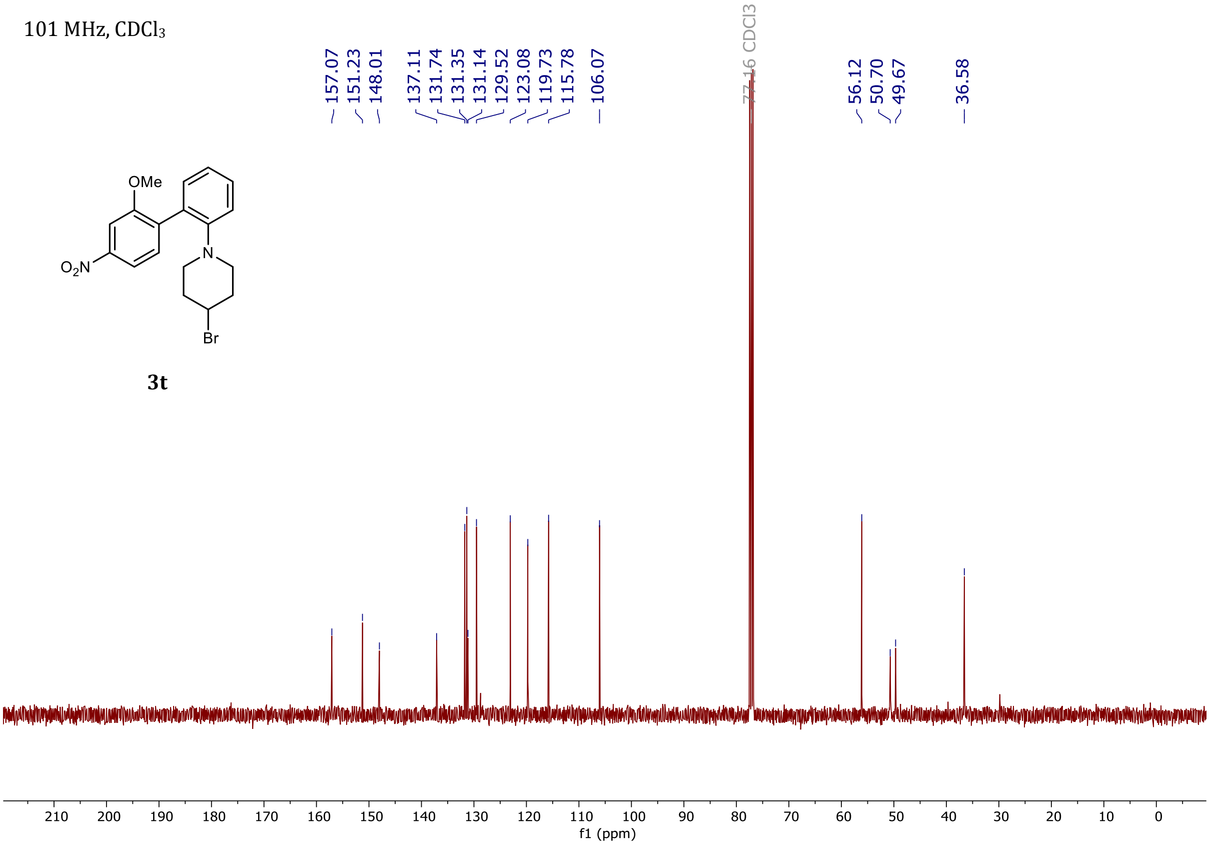

3t

400 MHz, $CDCl_3$

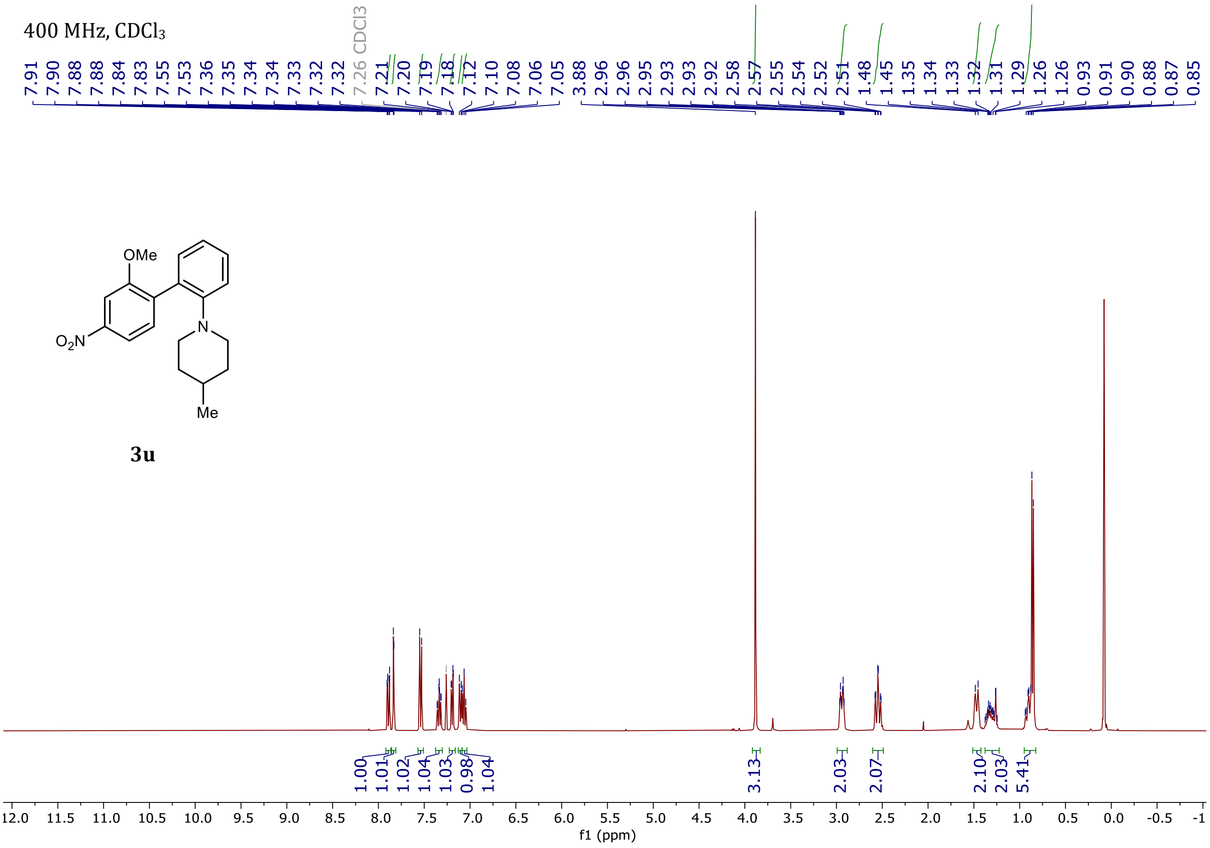

3u

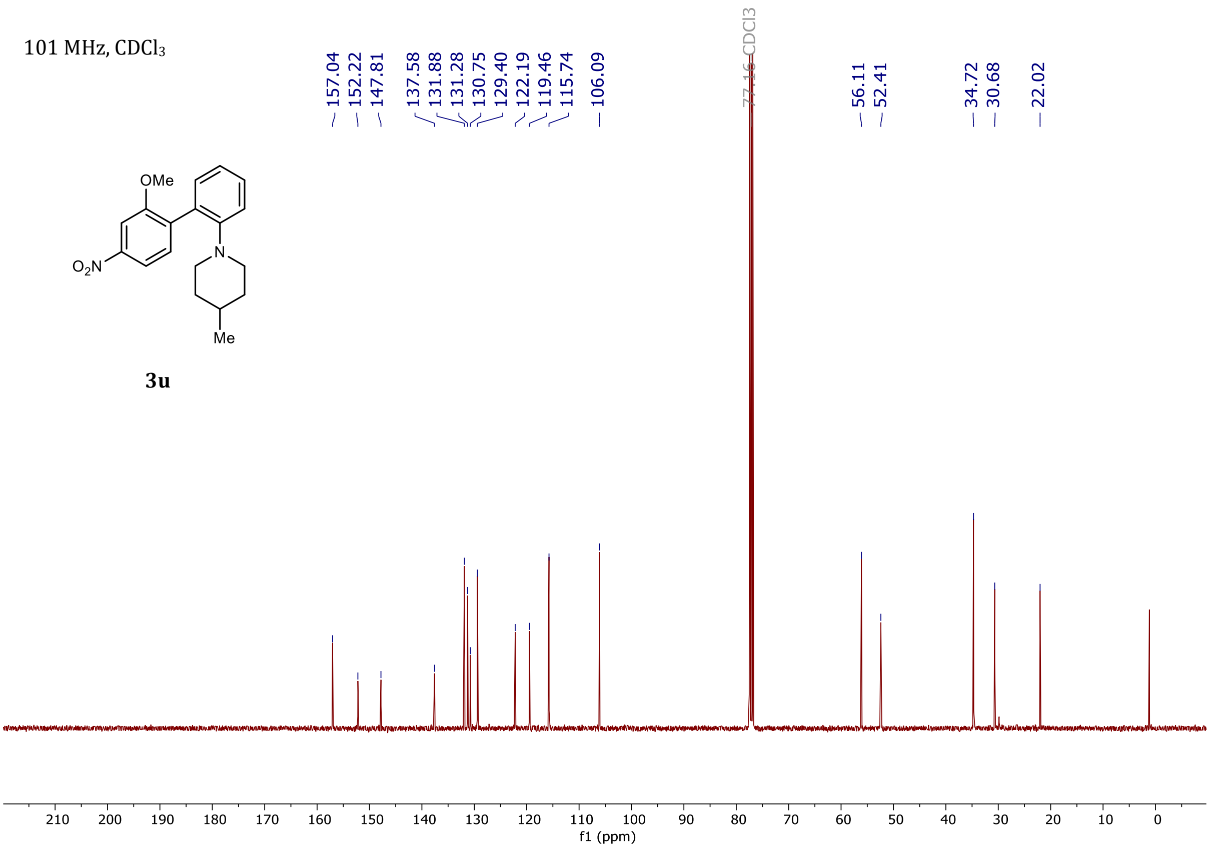
101 MHz, CDCl3
157.04
152.22
147.81
137.58
131.88
131.28
130.75
129.40
122.19
119.46
115.74
106.09
77.16 CDCl3
56.11
52.41
34.72
30.68
22.02
OMe
O2N
N
Me
3u
f1 (ppm)

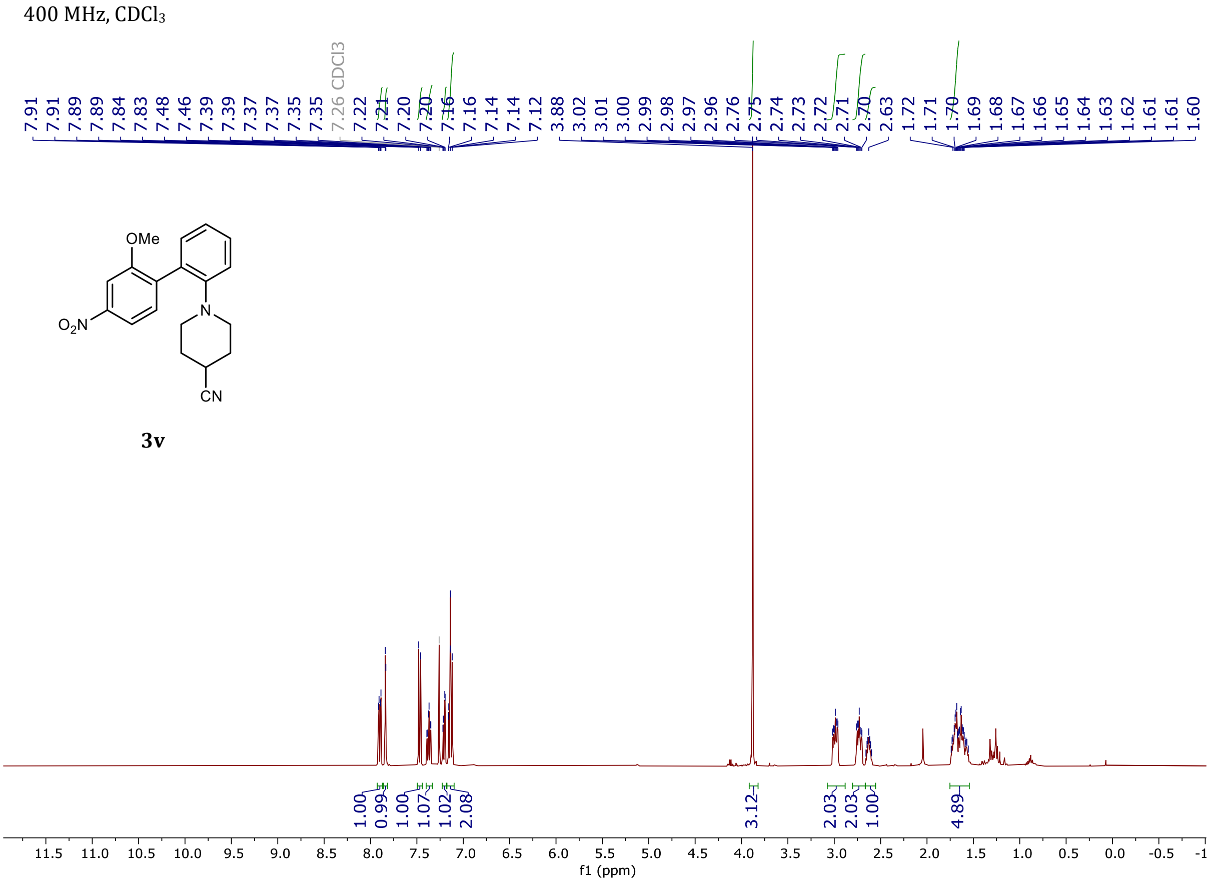
400 MHz, CDCl3
7.91
7.91
7.89
7.89
7.84
7.83
7.48
7.46
7.39
7.39
7.37
7.37
7.35
7.35
7.26 CDCl3
7.22
7.21
7.20
7.20
7.16
7.16
7.14
7.12
3.88
3.02
3.01
3.00
2.99
2.98
2.97
2.96
2.76
2.75
2.74
2.73
2.72
2.71
2.70
2.63
1.72
1.71
1.70
1.69
1.68
1.67
1.66
1.65
1.64
1.63
1.62
1.61
1.60
1.00
0.99
1.00
1.07
1.02
2.08
3.12
2.03
2.03
1.00
4.89
OMe
O2N
N
CN
3v
f1 (ppm)

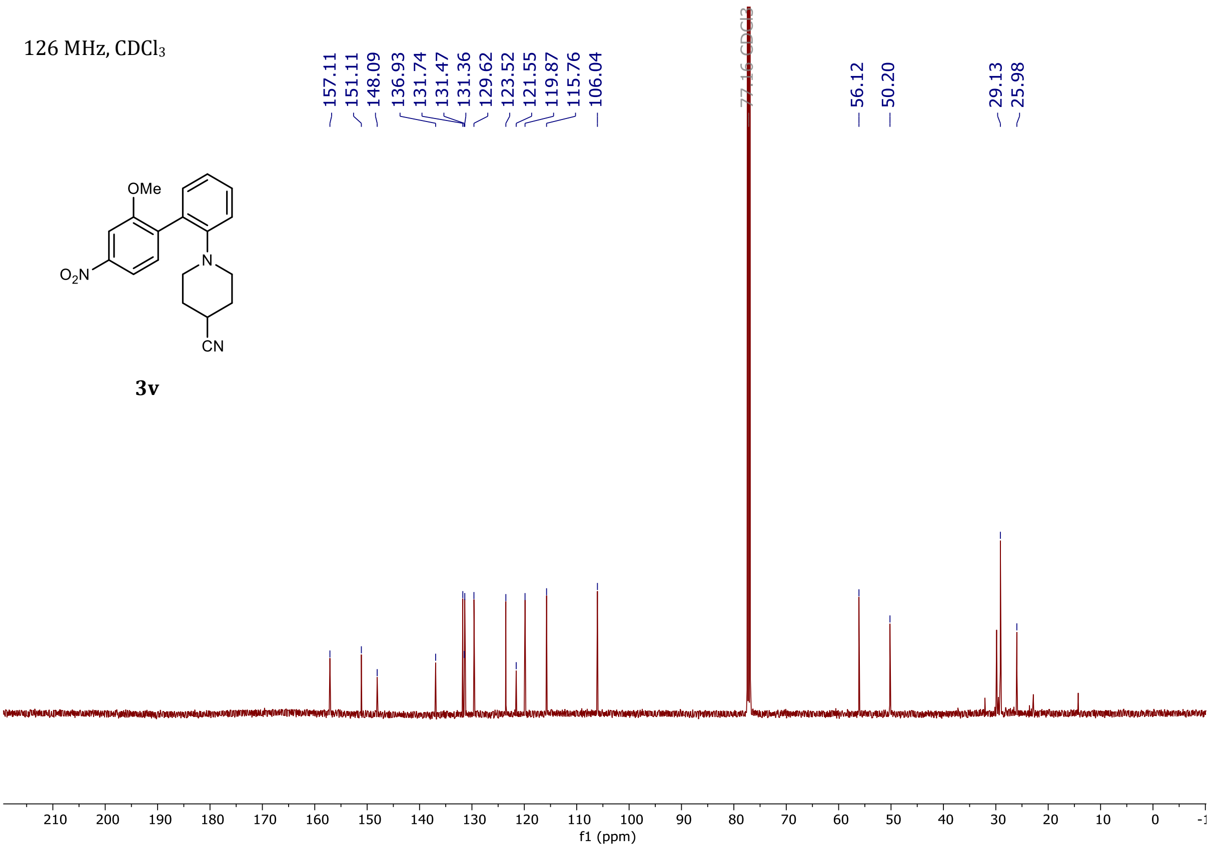
126 MHz, $CDCl_3$
157.11
151.11
148.09
136.93
131.74
131.47
131.36
129.62
123.52
121.55
119.87
115.76
106.04
77.16 CDCl3
56.12
50.20
29.13
25.98
OMe
O_2N
N
CN
3v
f1 (ppm)

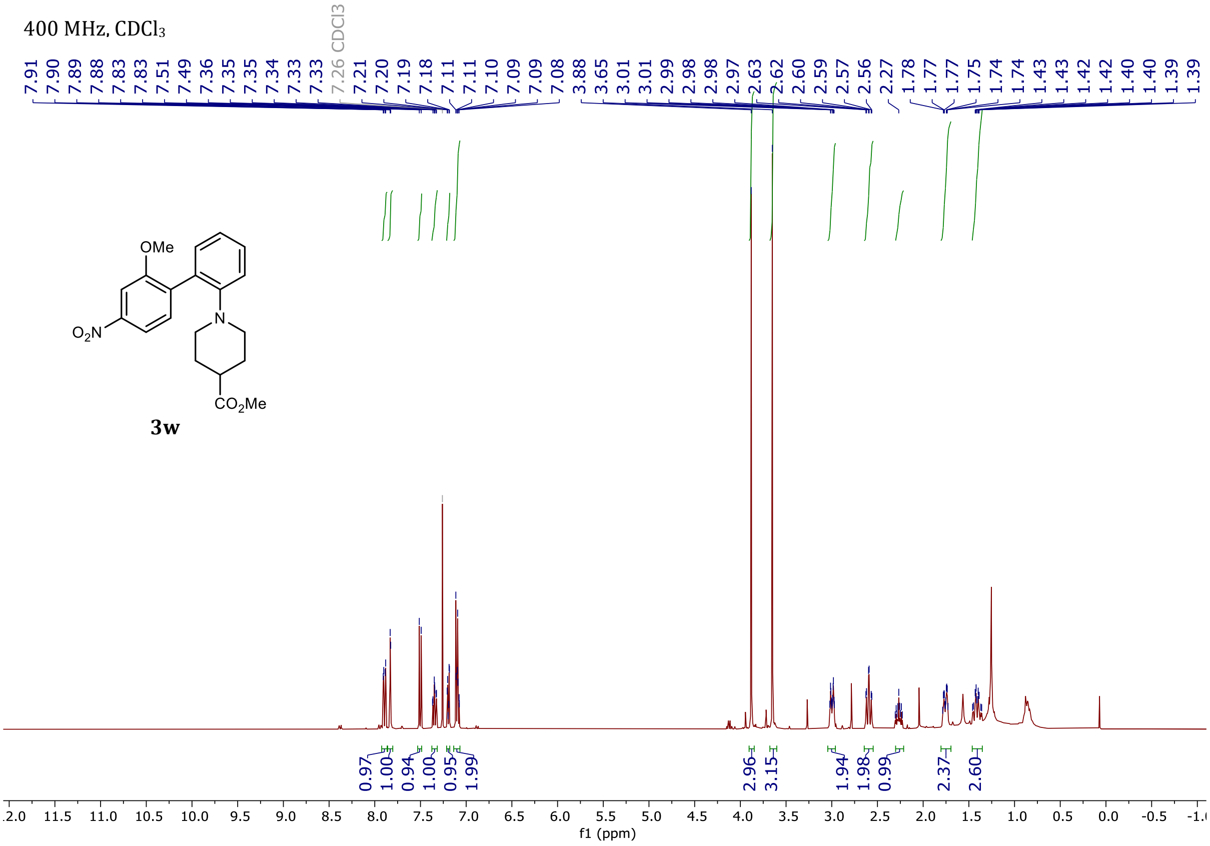
400 MHz, $CDCl_3$
7.91 7.90 7.89 7.88 7.83 7.83 7.51 7.49 7.36 7.35 7.35 7.34 7.33 7.33 7.26 CDCl3 7.21 7.20 7.19 7.18 7.11 7.11 7.10 7.09 7.09 7.08 3.88 3.65 3.01 2.99 2.98 2.98 2.97 2.63 2.62 2.60 2.59 2.57 2.56 2.27 1.78 1.77 1.77 1.75 1.74 1.74 1.43 1.43 1.42 1.42 1.40 1.39 1.39
OMe
O_2N
N
CO_2Me
3w
0.97 1.00 0.94 1.00 0.95 1.99 2.96 3.15 1.94 1.98 0.99 2.37 2.60
f1 (ppm)

101 MHz, $CDCl_3$

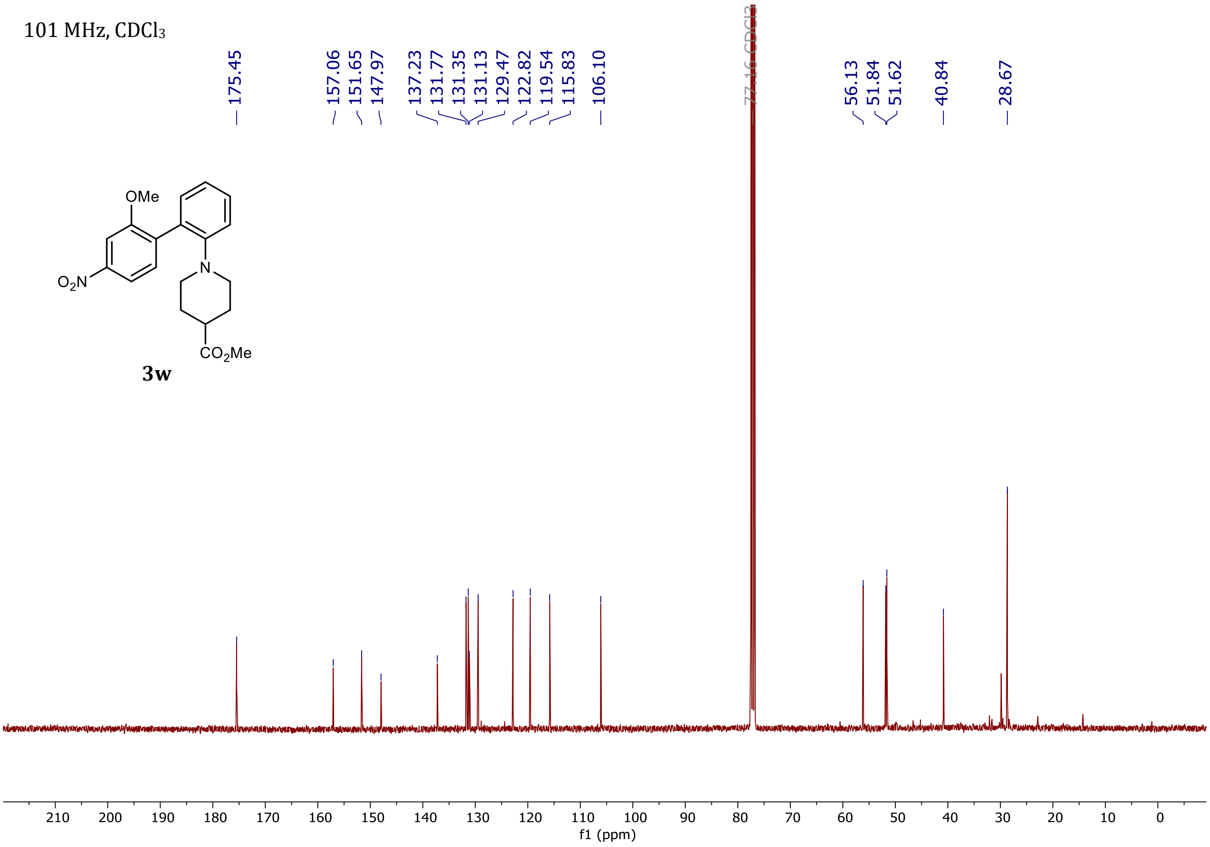

400 MHz, $CDCl_3$

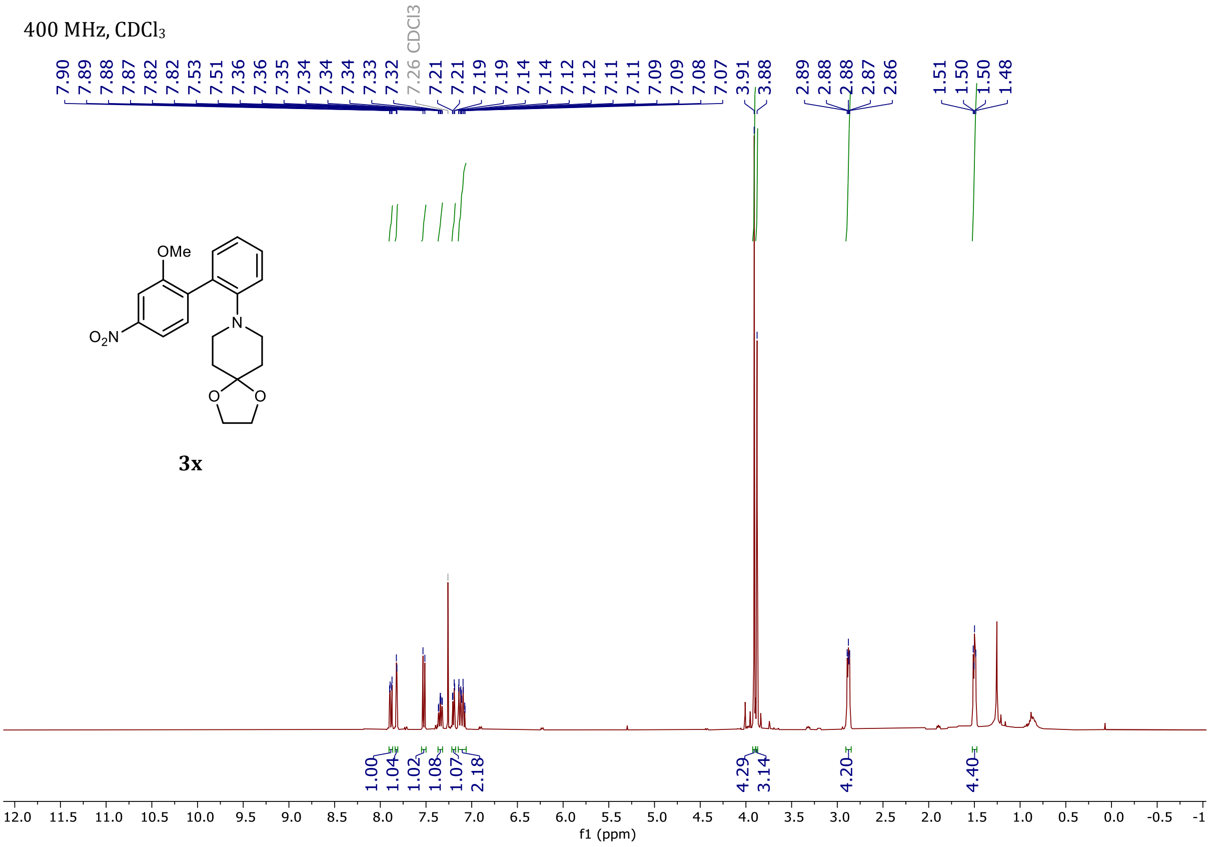

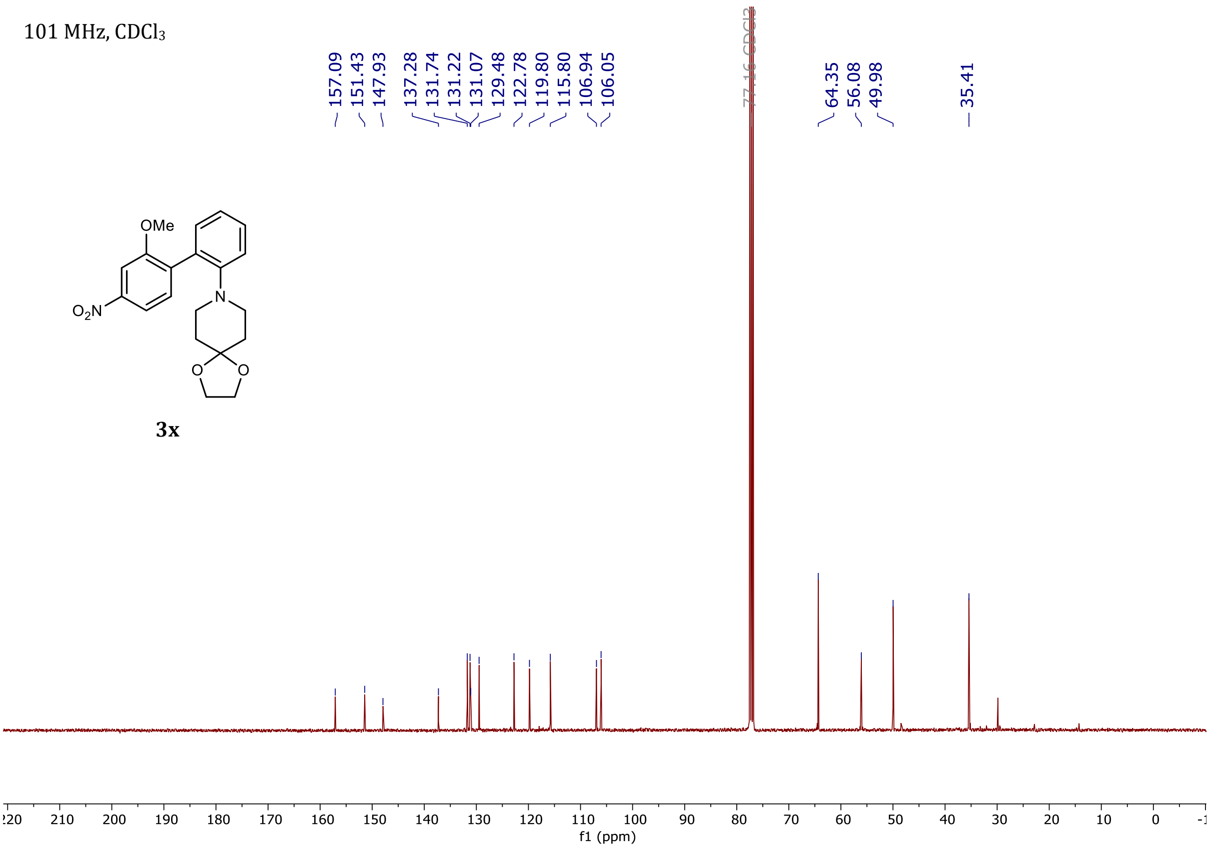
101 MHz, CDCl3
157.09
151.43
147.93
137.28
131.74
131.22
131.07
129.48
122.78
119.80
115.80
106.94
106.05
77.16 CDCl3
64.35
56.08
49.98
35.41
OMe
O2N
N
O
O
3x
f1 (ppm)

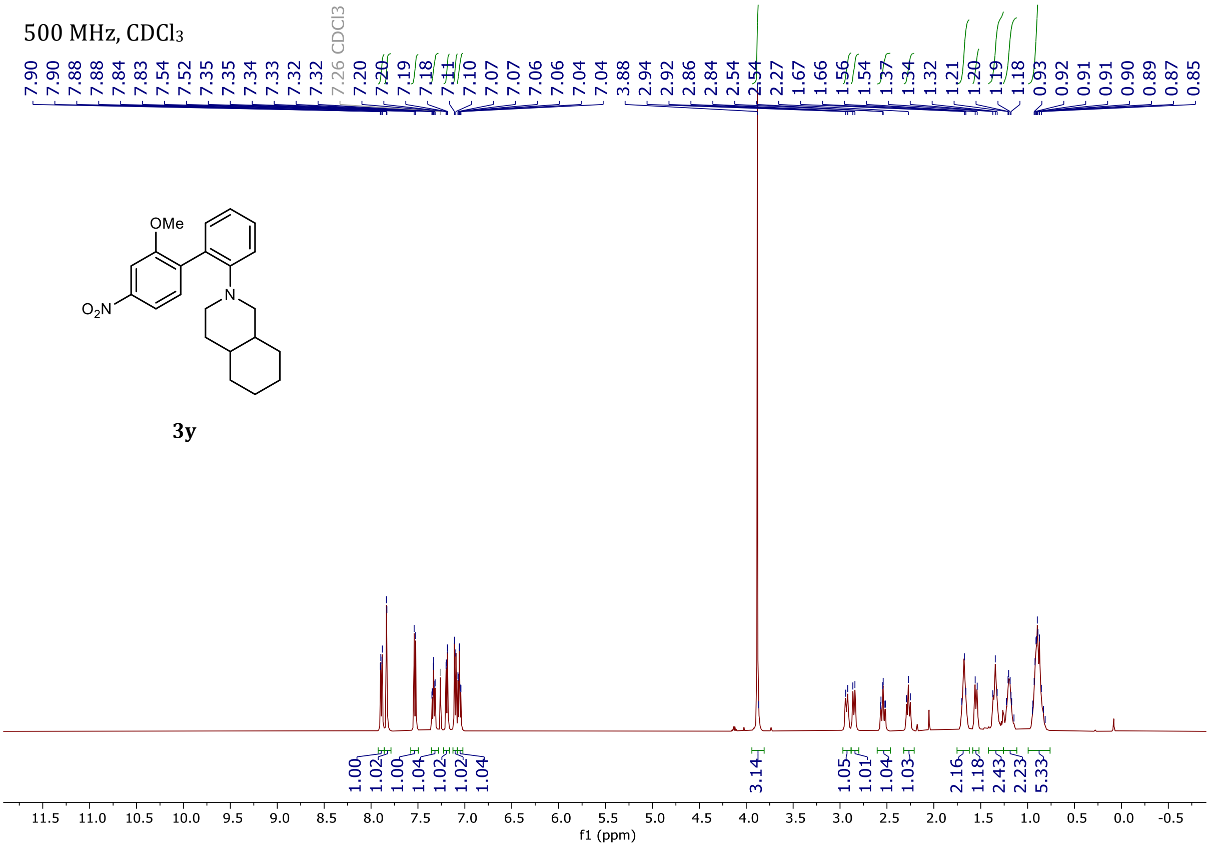
500 MHz, CDCl3
7.90
7.90
7.88
7.88
7.84
7.83
7.54
7.52
7.35
7.35
7.34
7.33
7.32
7.32
7.26 CDCl3
7.20
7.19
7.18
7.11
7.10
7.07
7.06
7.06
7.04
3.88
2.94
2.92
2.86
2.84
2.54
2.54
2.27
1.67
1.66
1.56
1.54
1.37
1.34
1.32
1.21
1.20
1.19
1.18
0.93
0.92
0.91
0.91
0.90
0.89
0.87
0.85
OMe
O2N
N
3y
1.00
1.02
1.00
1.04
1.02
1.02
1.04
3.14
1.05
1.01
1.04
1.03
2.16
1.18
2.43
2.23
5.33
f1 (ppm)

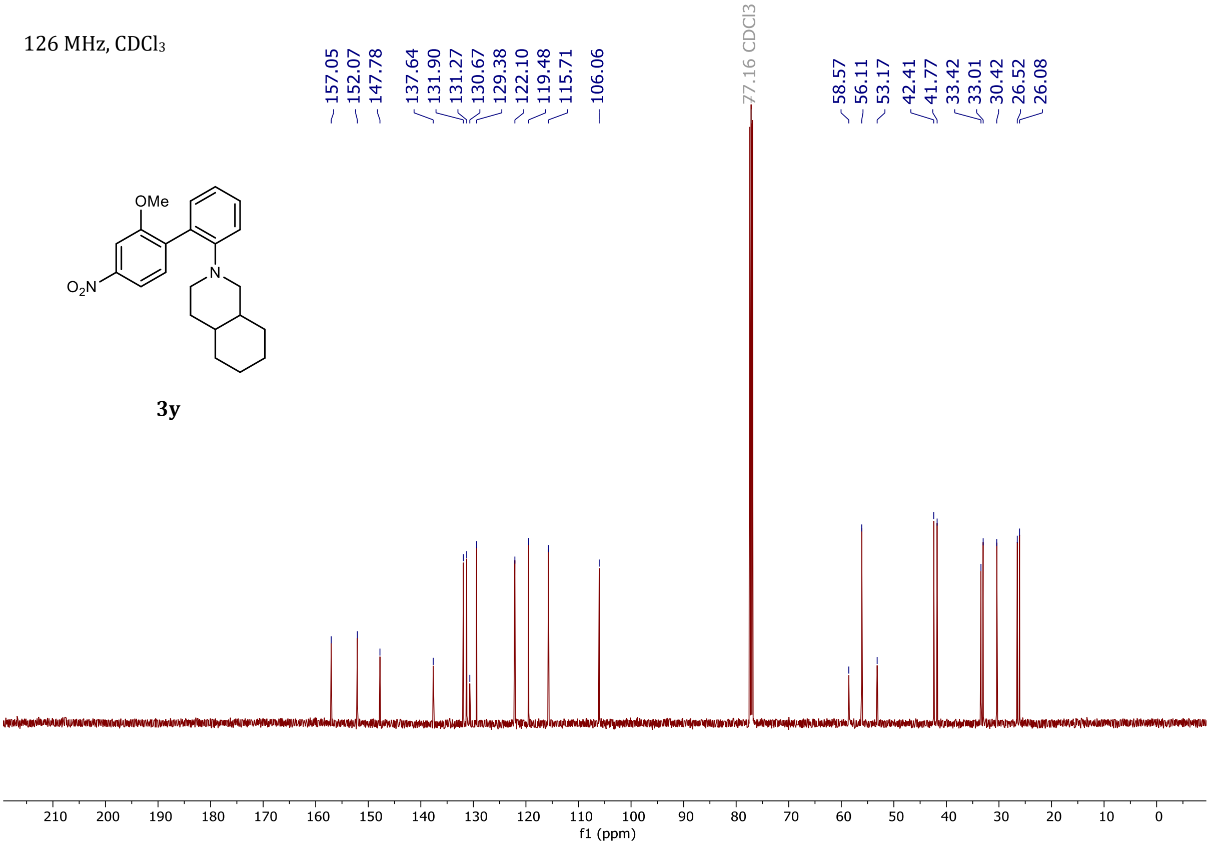
126 MHz, CDCl3
157.05
152.07
147.78
137.64
131.90
131.27
130.67
129.38
122.10
119.48
115.71
106.06
77.16 CDCl3
58.57
56.11
53.17
42.41
41.77
33.42
33.01
30.42
26.52
26.08
OMe
O2N
N
3y
f1 (ppm)

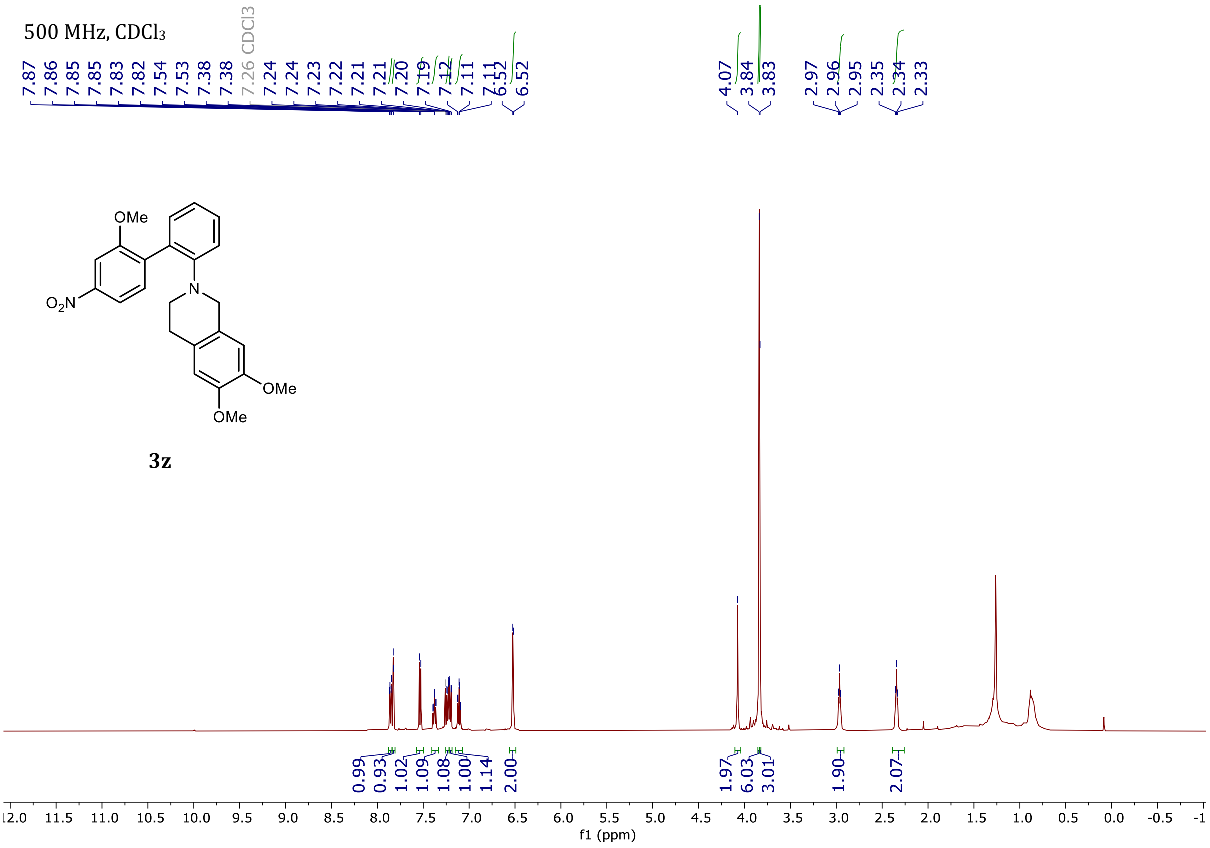
500 MHz, CDCl3
7.87
7.86
7.85
7.85
7.83
7.82
7.54
7.53
7.38
7.38
7.26 CDCl3
7.24
7.24
7.23
7.22
7.21
7.21
7.20
7.19
7.12
7.11
6.52
6.52
4.07
3.84
3.83
2.97
2.96
2.95
2.35
2.34
2.33
OMe
O2N
N
OMe
OMe
3z
0.99
0.93
1.02
1.09
1.08
1.00
1.14
2.00
1.97
6.03
3.01
1.90
2.07
f1 (ppm)

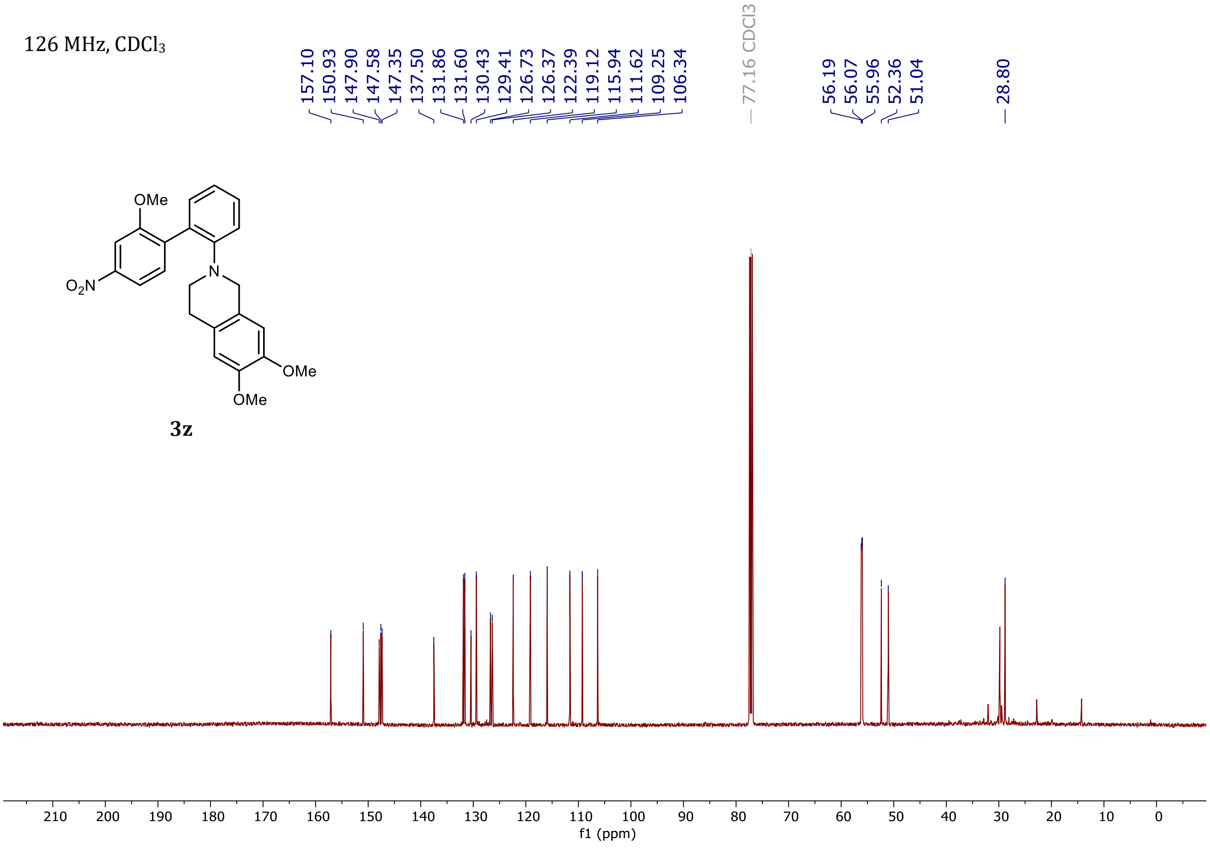
126 MHz, CDCl3
157.10
150.93
147.90
147.58
147.35
137.50
131.86
131.60
130.43
129.41
126.73
126.37
122.39
119.12
115.94
111.62
109.25
106.34
77.16 CDCl3
56.19
56.07
55.96
52.36
51.04
28.80
OMe
O2N
N
OMe
OMe
3z
210 200 190 180 170 160 150 140 130 120 110 100 90 80 70 60 50 40 30 20 10 0
f1 (ppm)

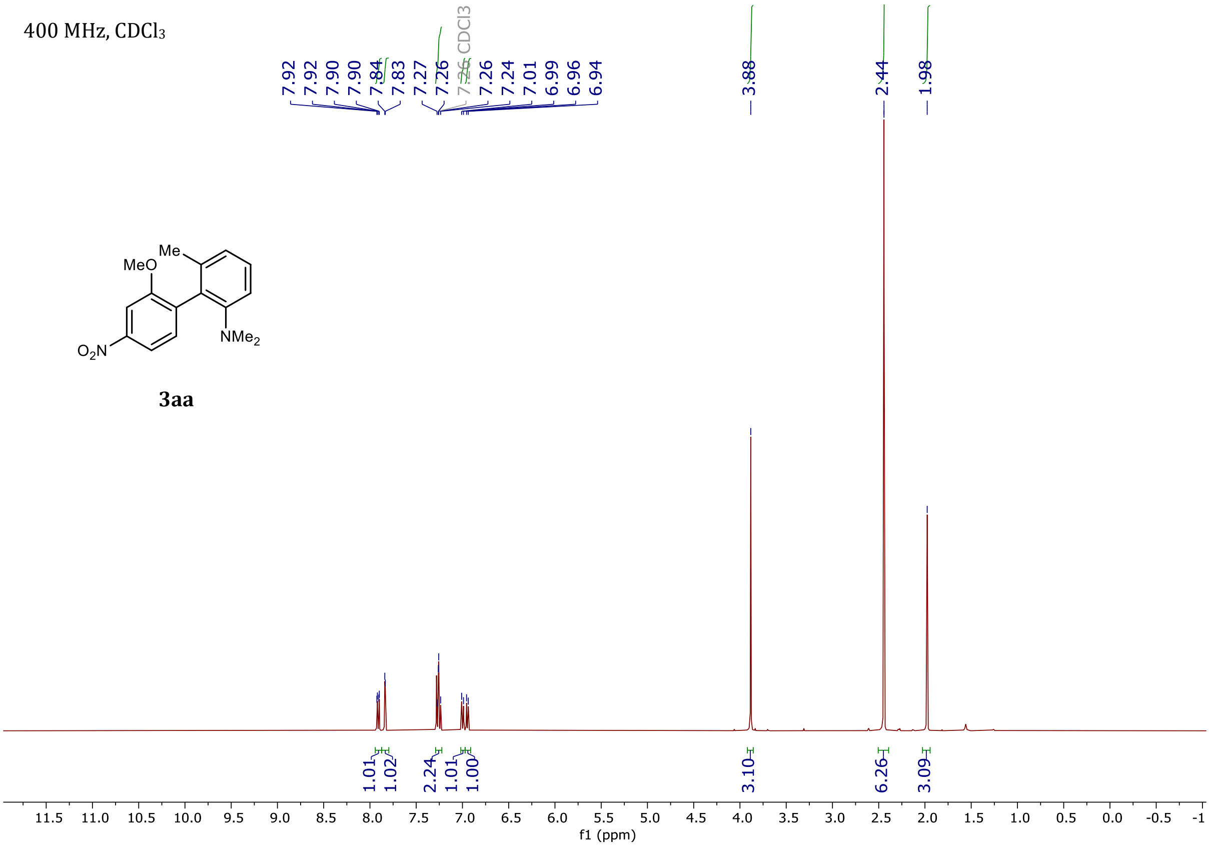
400 MHz, CDCl3
7.92
7.92
7.90
7.90
7.84
7.83
7.27
7.26
7.26 CDCl3
7.26
7.24
7.01
6.99
6.96
6.94
3.88
2.44
1.98
Me
MeO
O2N
NMe2
3aa
1.01
1.02
2.24
1.01
1.00
3.10
6.26
3.09
11.5 11.0 10.5 10.0 9.5 9.0 8.5 8.0 7.5 7.0 6.5 6.0 5.5 5.0 4.5 4.0 3.5 3.0 2.5 2.0 1.5 1.0 0.5 0.0 -0.5 -1
f1 (ppm)

101 MHz, CDCl₃

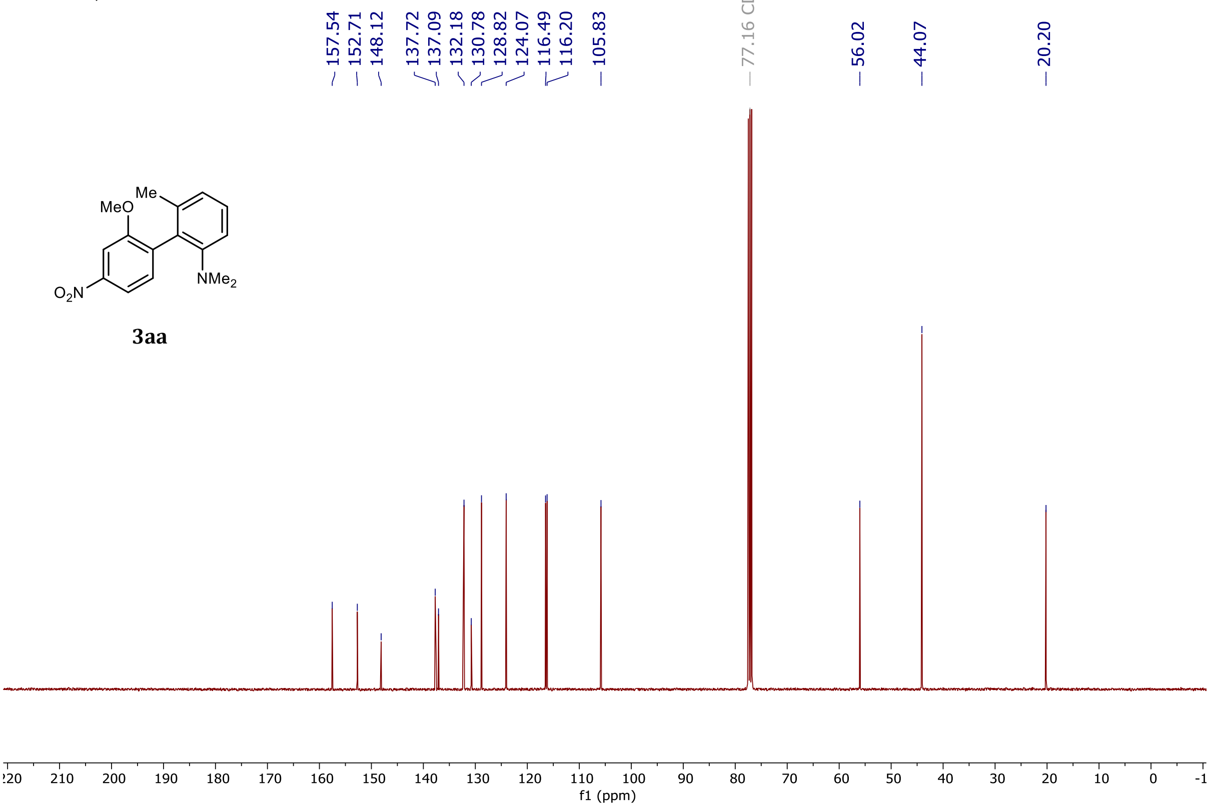

400 MHz, CDCl₃

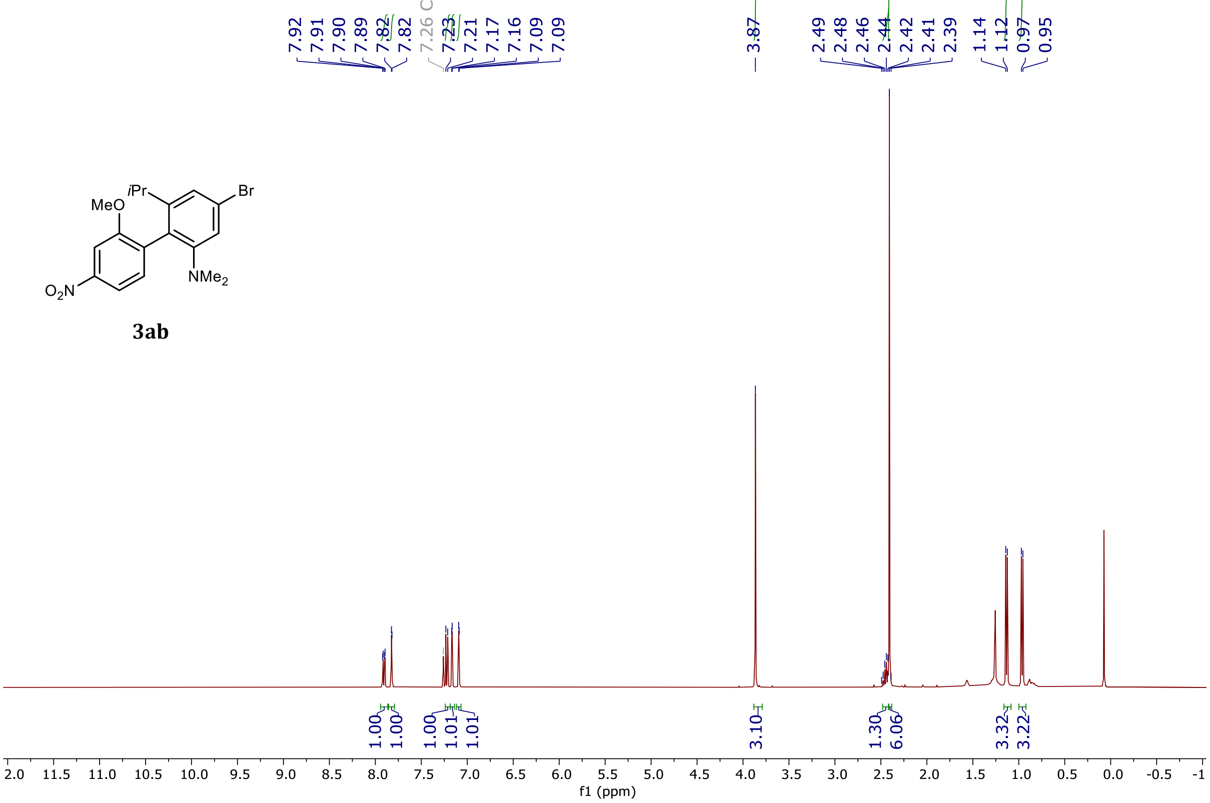

101 MHz, $CDCl_3$

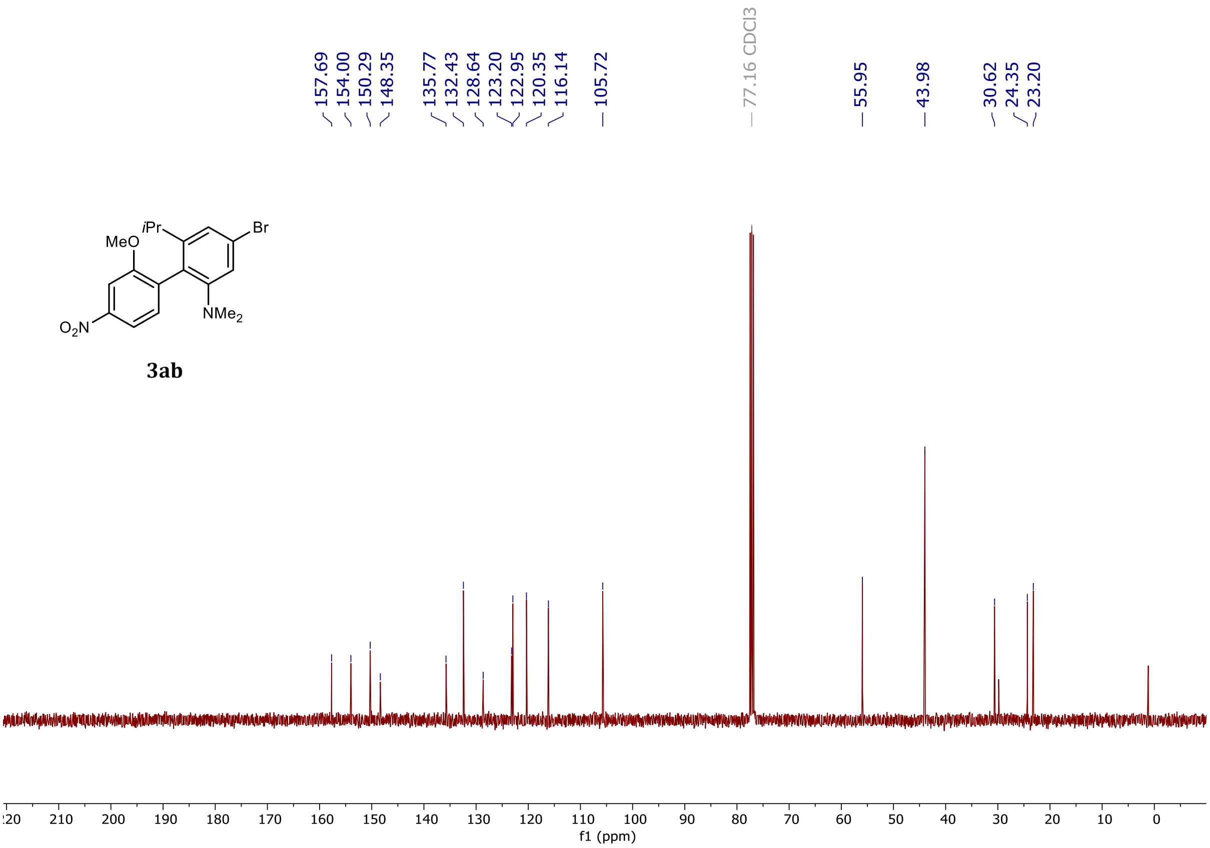

3ab

400 MHz, $CDCl_3$

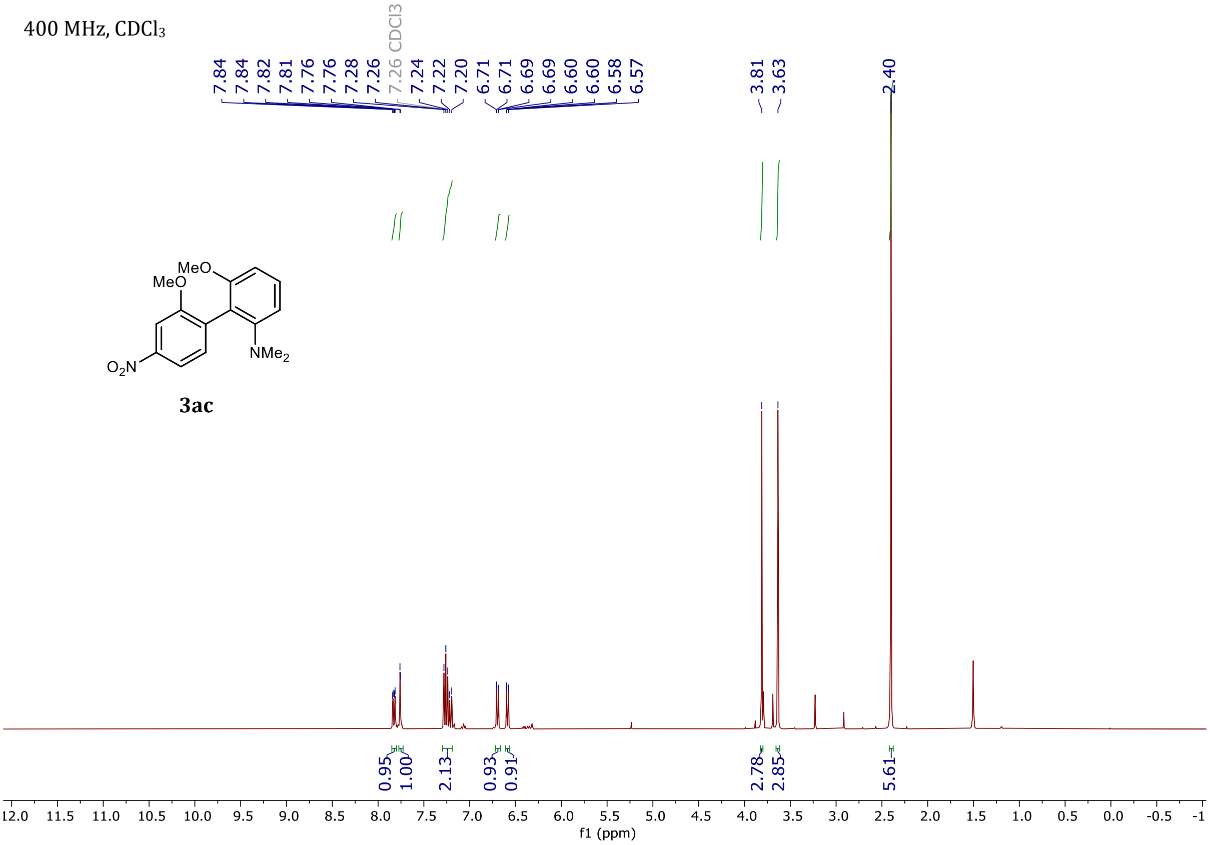

3ac

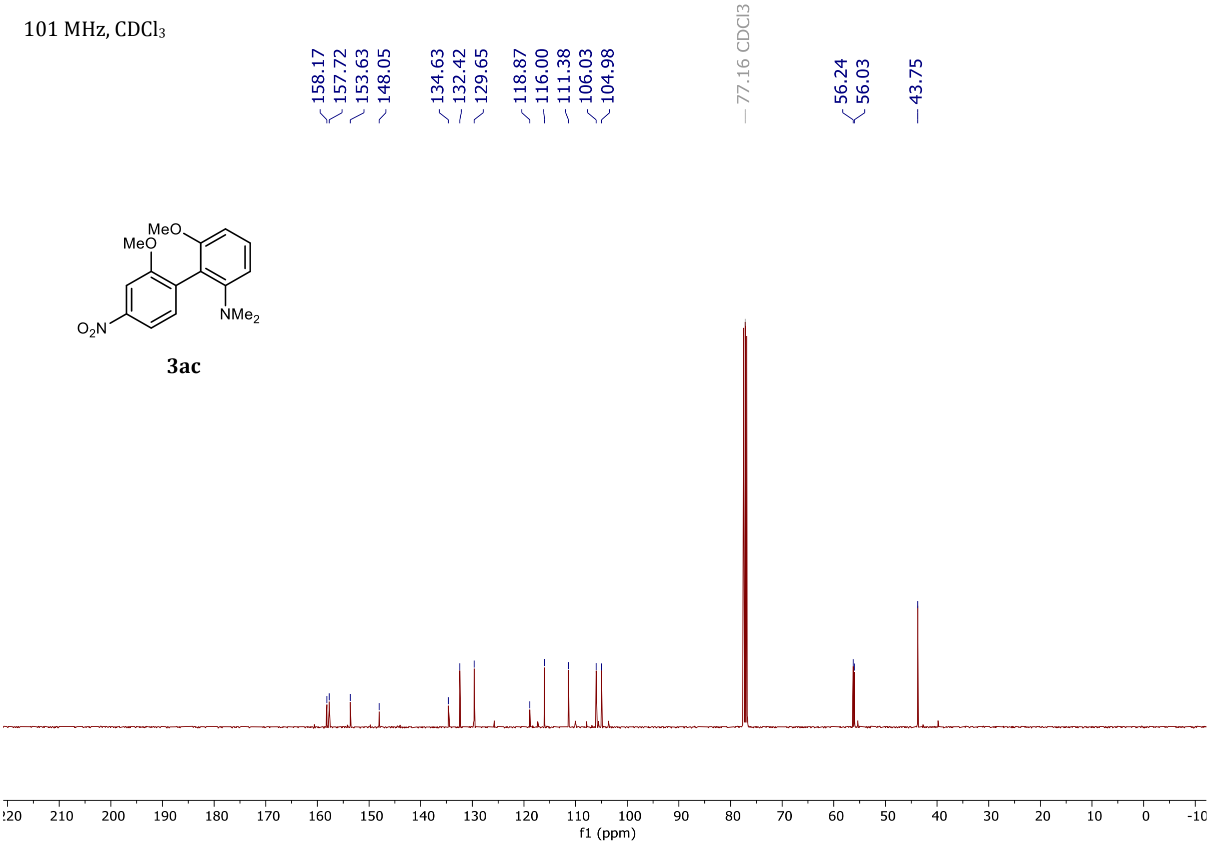
101 MHz, CDCl3
158.17
157.72
153.63
148.05
134.63
132.42
129.65
118.87
116.00
111.38
106.03
104.98
77.16 CDCl3
56.24
56.03
43.75
MeO
MeO
O2N
NMe2
3ac
f1 (ppm)

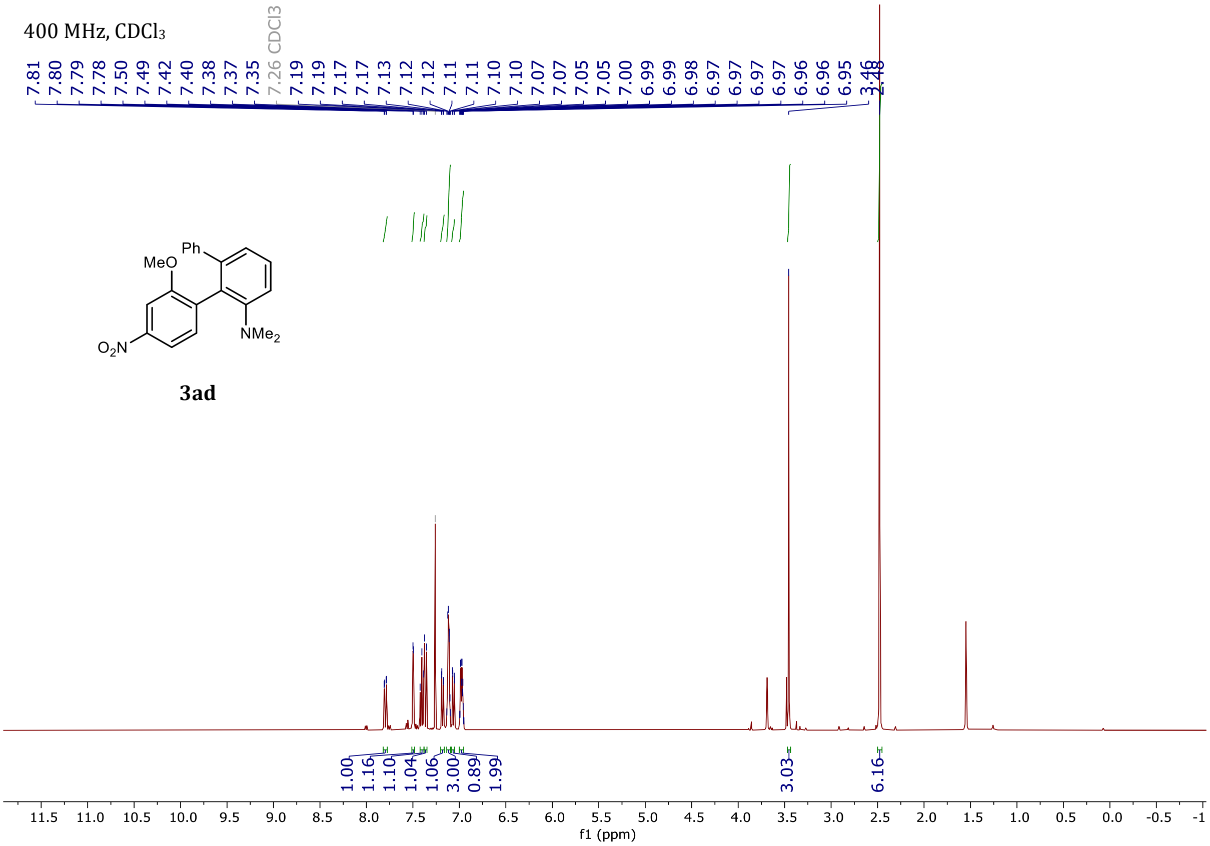
400 MHz, CDCl3
7.81
7.80
7.79
7.78
7.50
7.49
7.42
7.40
7.38
7.37
7.35
7.26 CDCl3
7.19
7.19
7.17
7.17
7.13
7.12
7.12
7.11
7.11
7.10
7.07
7.07
7.05
7.05
7.00
6.99
6.99
6.98
6.97
6.97
6.97
6.97
6.96
6.96
6.95
3.48
2.48
Ph
MeO
O2N
NMe2
3ad
1.00
1.16
1.10
1.04
1.06
3.00
0.89
1.99
3.03
6.16
f1 (ppm)

101 MHz, $CDCl_3$

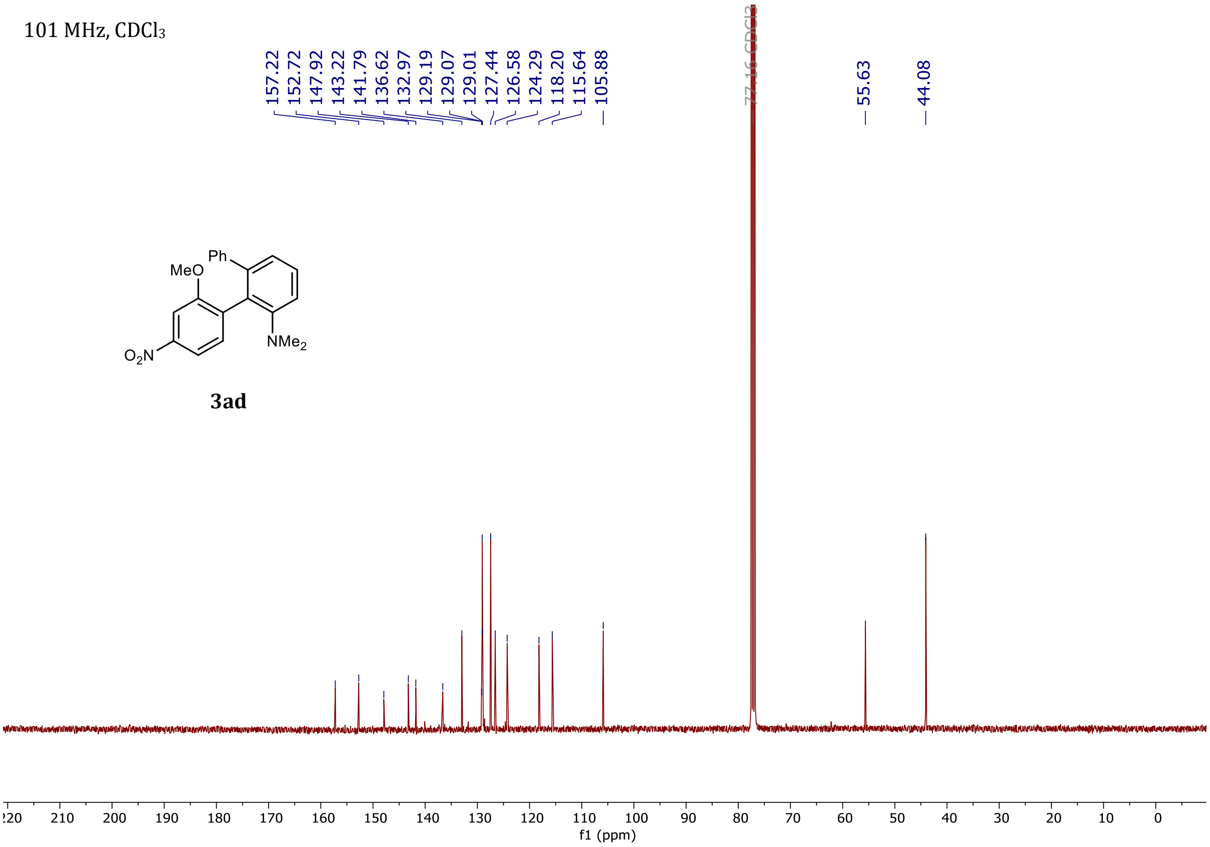

3ad

400 MHz, $CDCl_3$

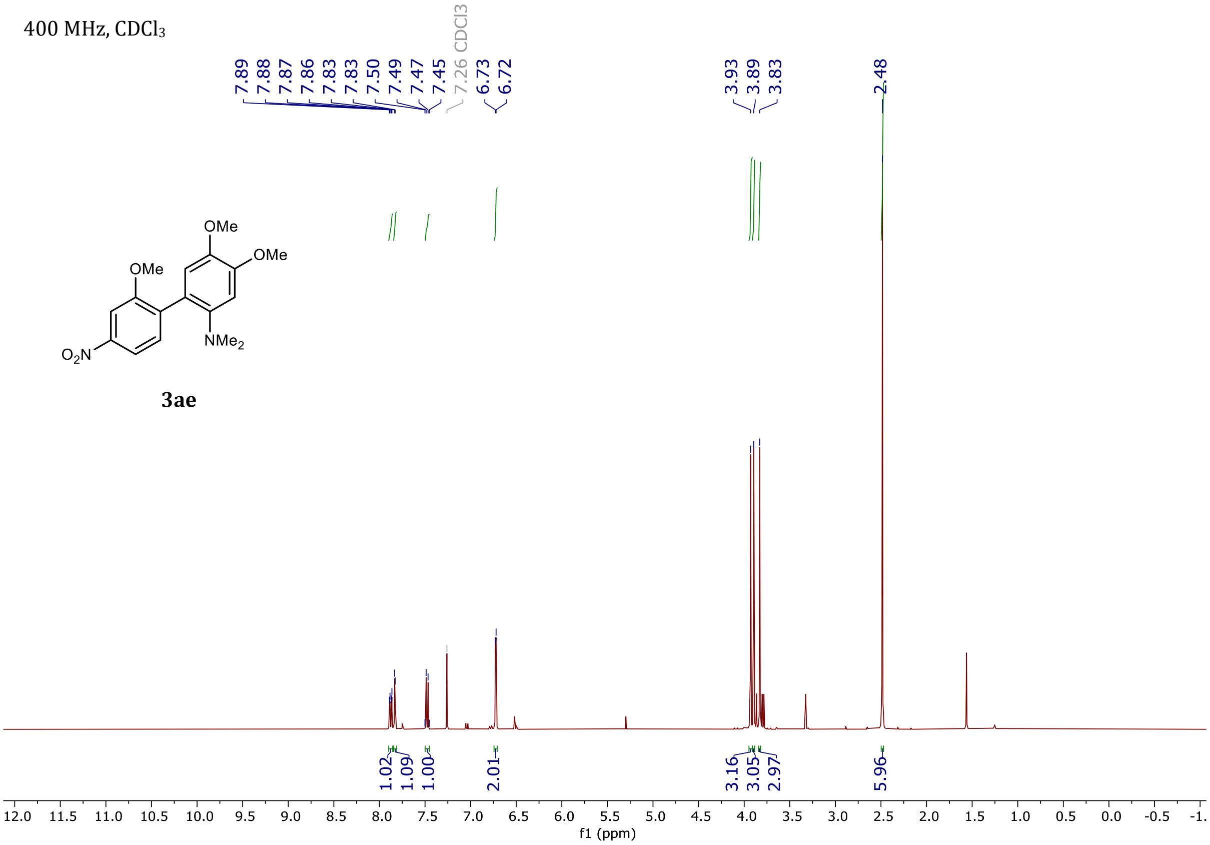

3ae

101 MHz, $CDCl_3$

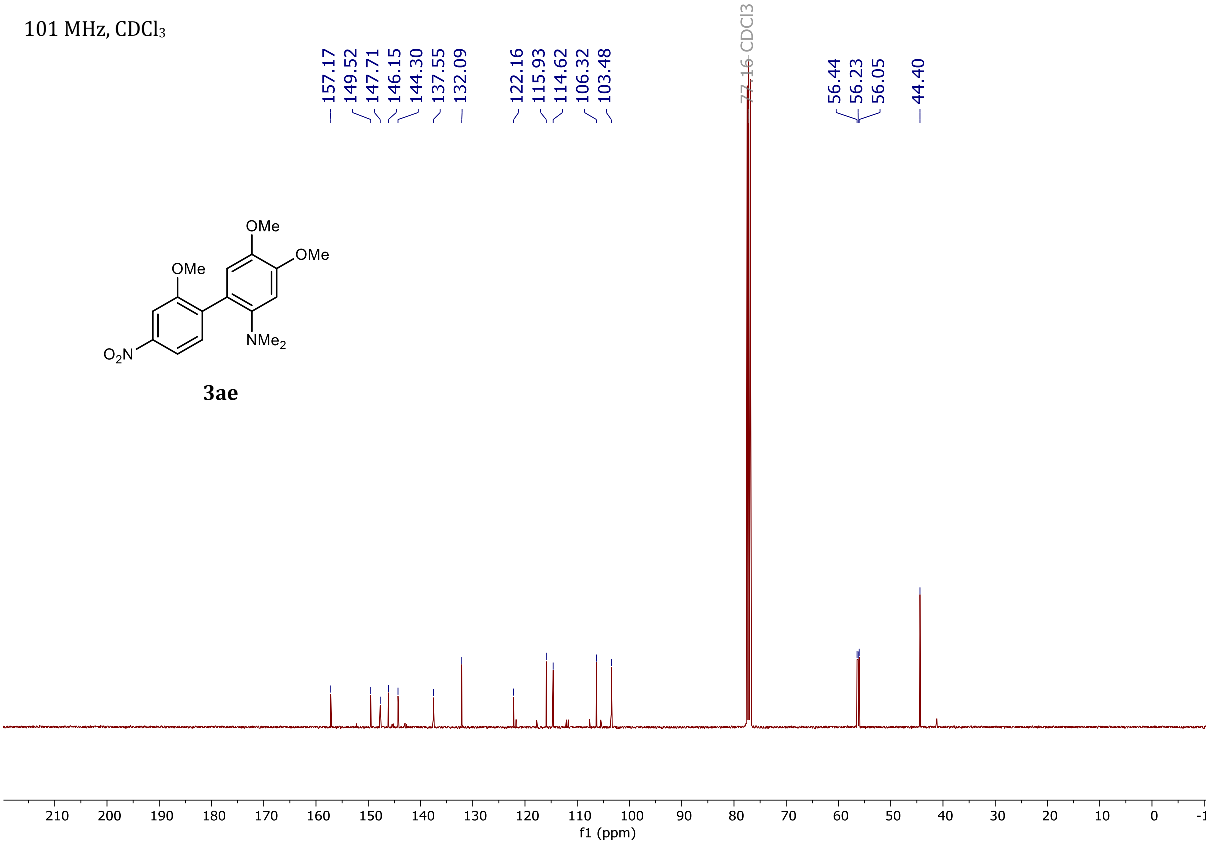

500 MHz, $CDCl_3$

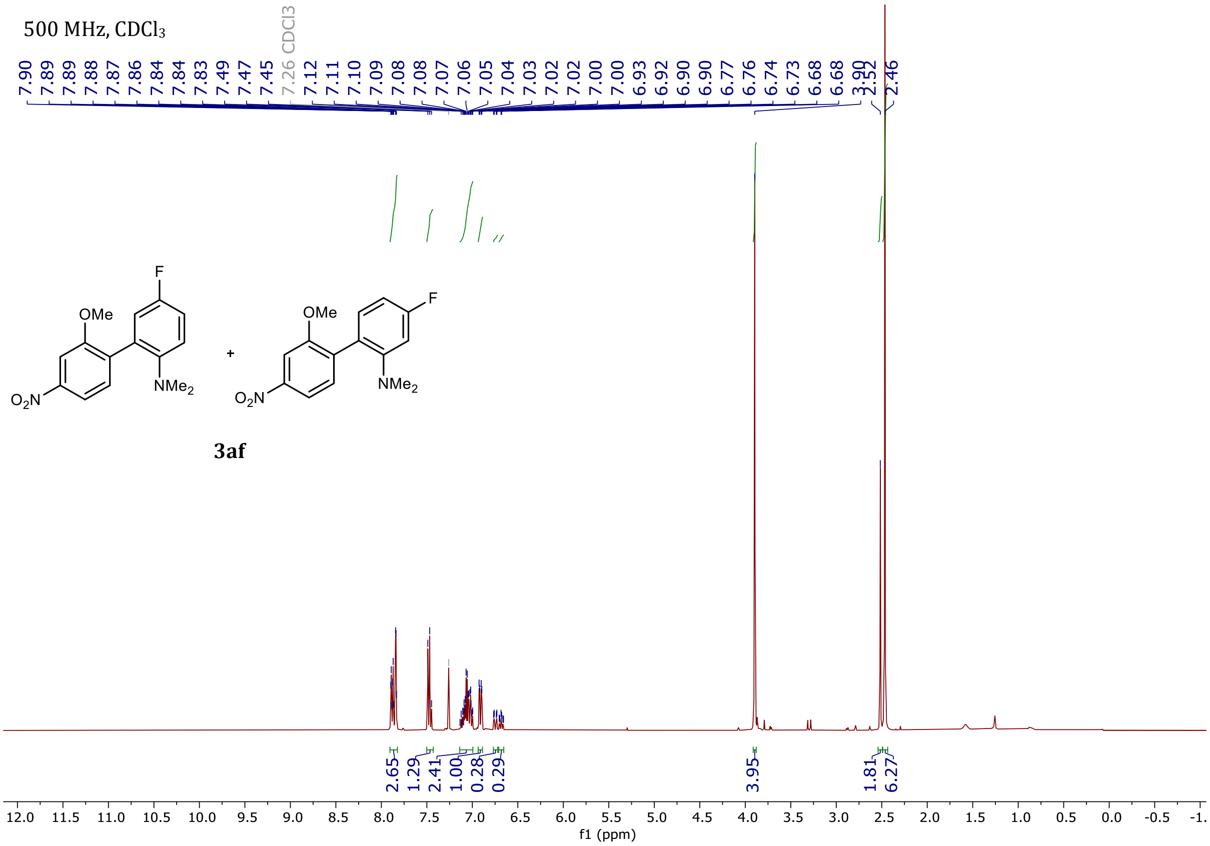

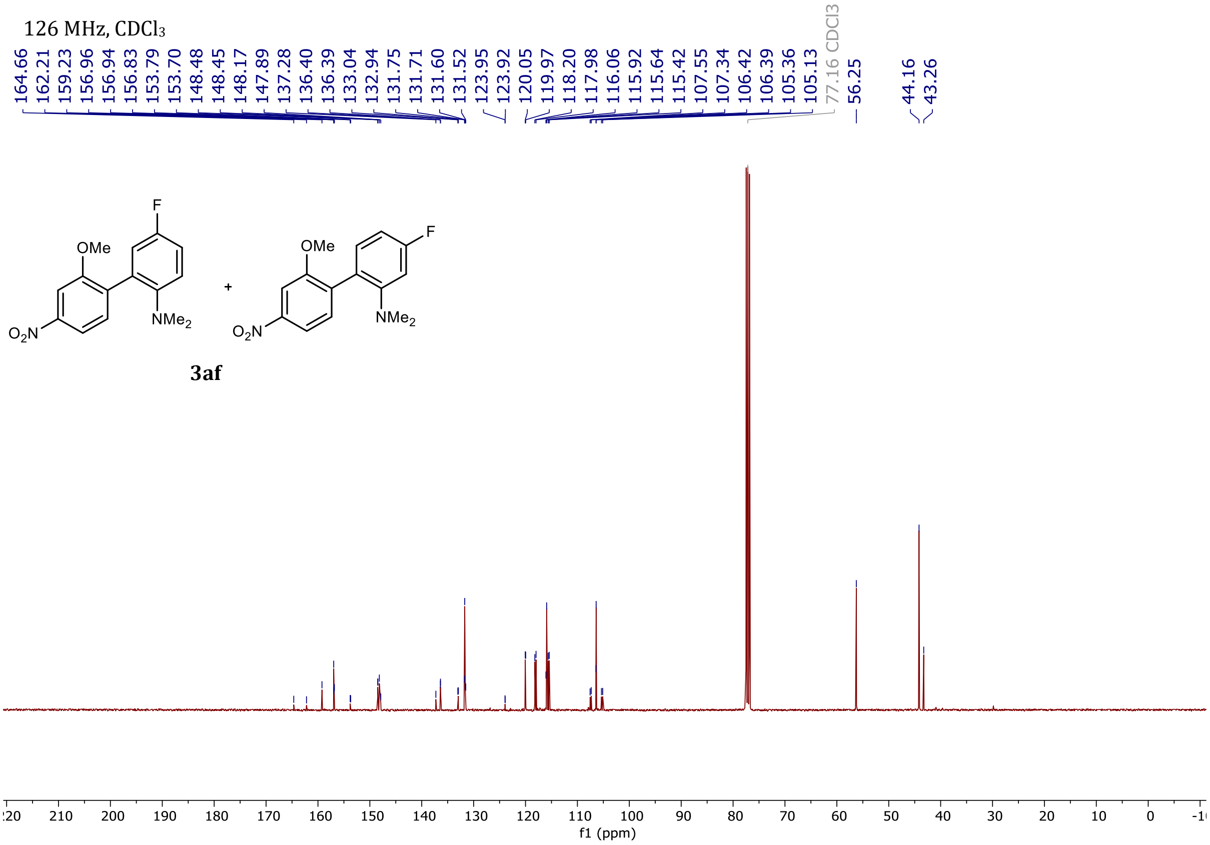
126 MHz, CDCl3
164.66
162.21
159.23
156.96
156.94
156.83
153.79
153.70
148.48
148.45
148.17
147.89
137.28
136.40
136.39
133.04
132.94
131.75
131.71
131.60
131.52
123.95
123.92
120.05
119.97
118.20
117.98
116.06
115.92
115.64
115.42
107.55
107.34
106.42
106.39
105.36
105.13
77.16 CDCl3
56.25
44.16
43.26
F
OMe
NMe2
O2N
+
3af
f1 (ppm)

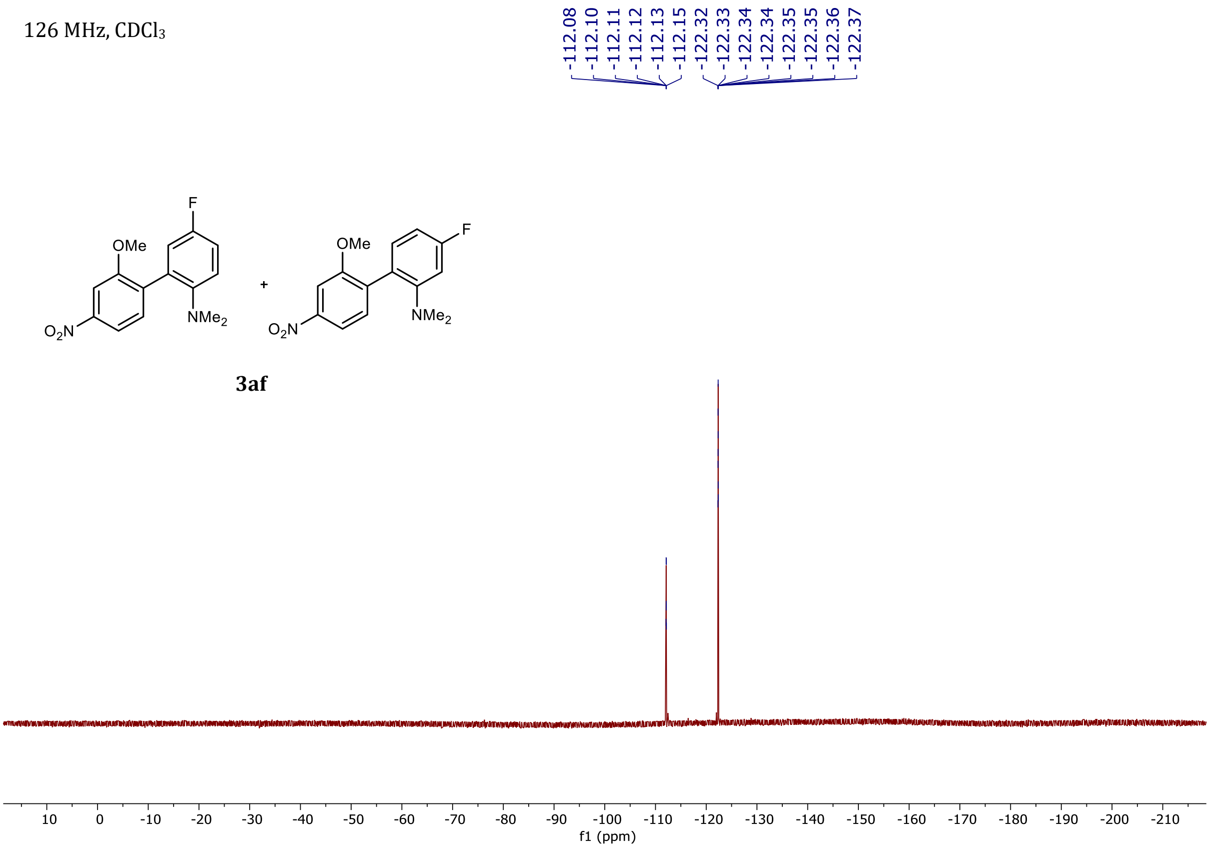
126 MHz, CDCl3
-112.08
-112.10
-112.11
-112.12
-112.13
-112.15
-122.32
-122.33
-122.34
-122.34
-122.35
-122.35
-122.36
-122.37
F
OMe
NMe2
O2N
+
3af
f1 (ppm)

400 MHz, $CDCl_3$

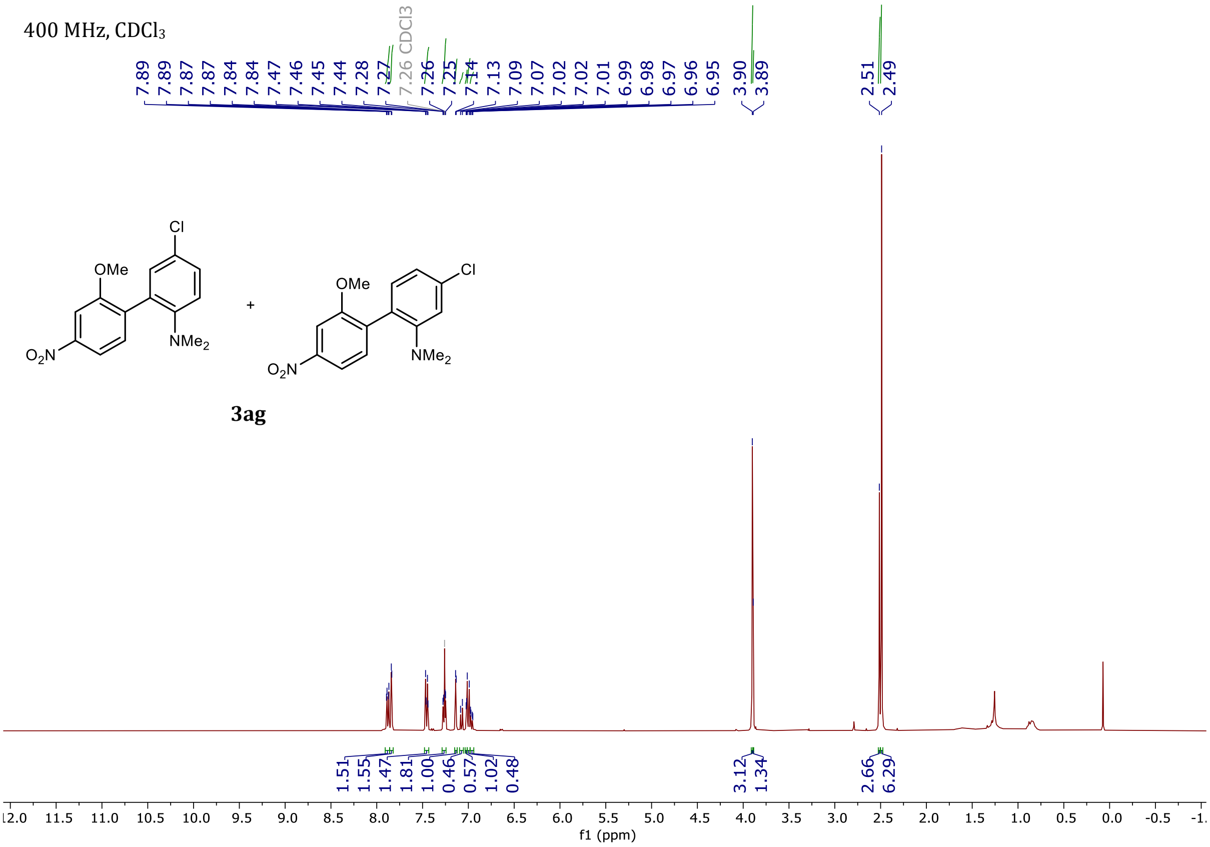

3ag

101 MHz, $CDCl_3$

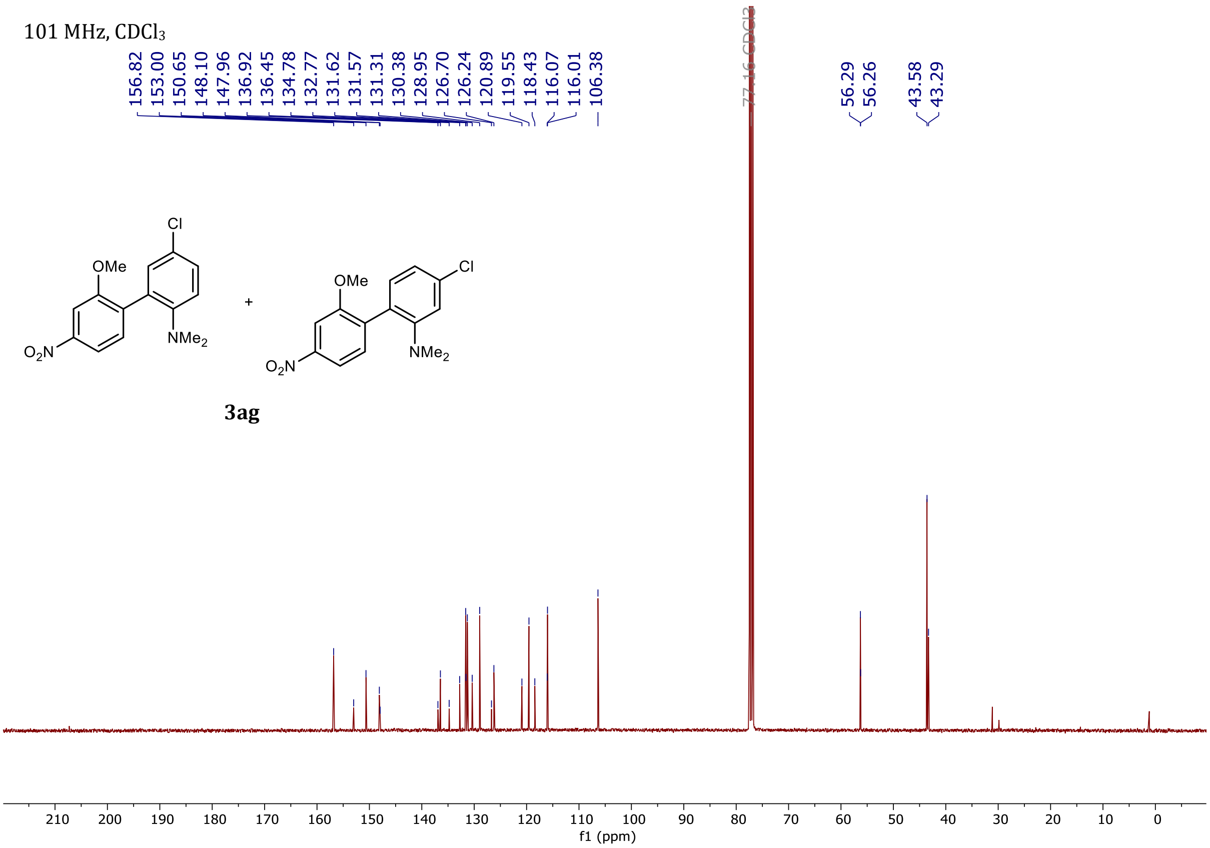

3ag

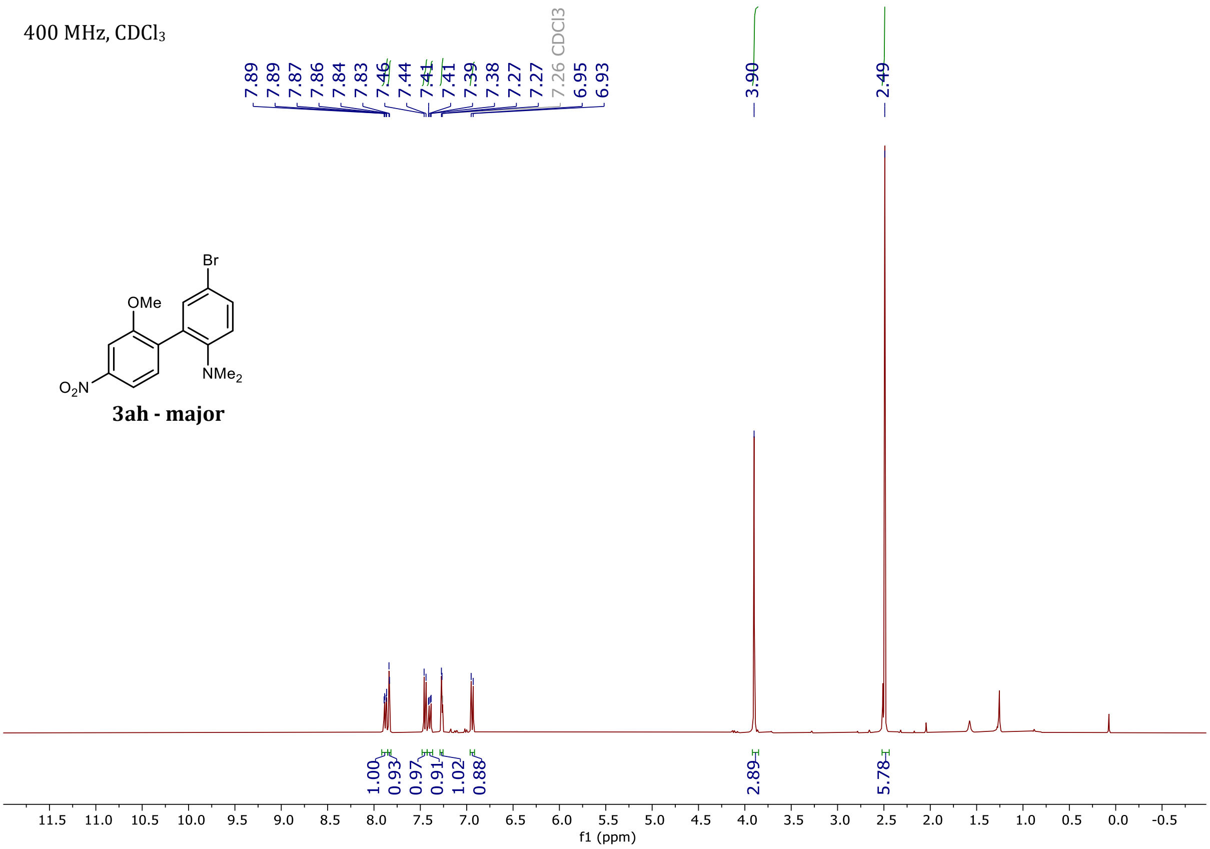
400 MHz, CDCl$_3$
7.89 7.89 7.87 7.86 7.84 7.83 7.46 7.44 7.41 7.41 7.39 7.38 7.27 7.27 7.26 CDCl3 6.95 6.93
3.90
2.49
Br
OMe
NMe$_2$
O$_2$N
3ah - major
1.00 0.93 0.97 0.91 1.02 0.88
2.89
5.78
11.5 11.0 10.5 10.0 9.5 9.0 8.5 8.0 7.5 7.0 6.5 6.0 5.5 5.0 4.5 4.0 3.5 3.0 2.5 2.0 1.5 1.0 0.5 0.0 -0.5
f1 (ppm)

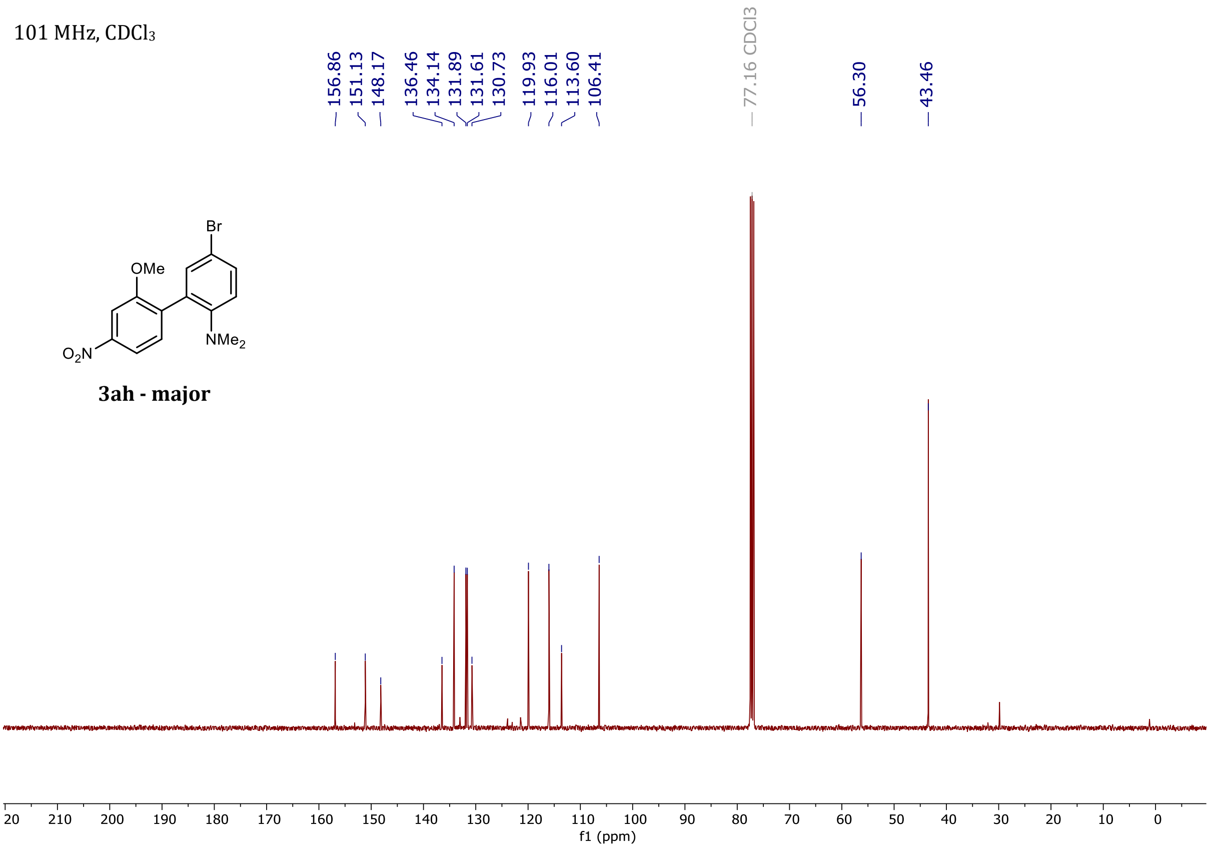
101 MHz, CDCl$_3$
156.86 151.13 148.17 136.46 134.14 131.89 131.61 130.73 119.93 116.01 113.60 106.41
77.16 CDCl3
56.30
43.46
Br
OMe
NMe$_2$
O$_2$N
3ah - major
20 210 200 190 180 170 160 150 140 130 120 110 100 90 80 70 60 50 40 30 20 10 0
f1 (ppm)

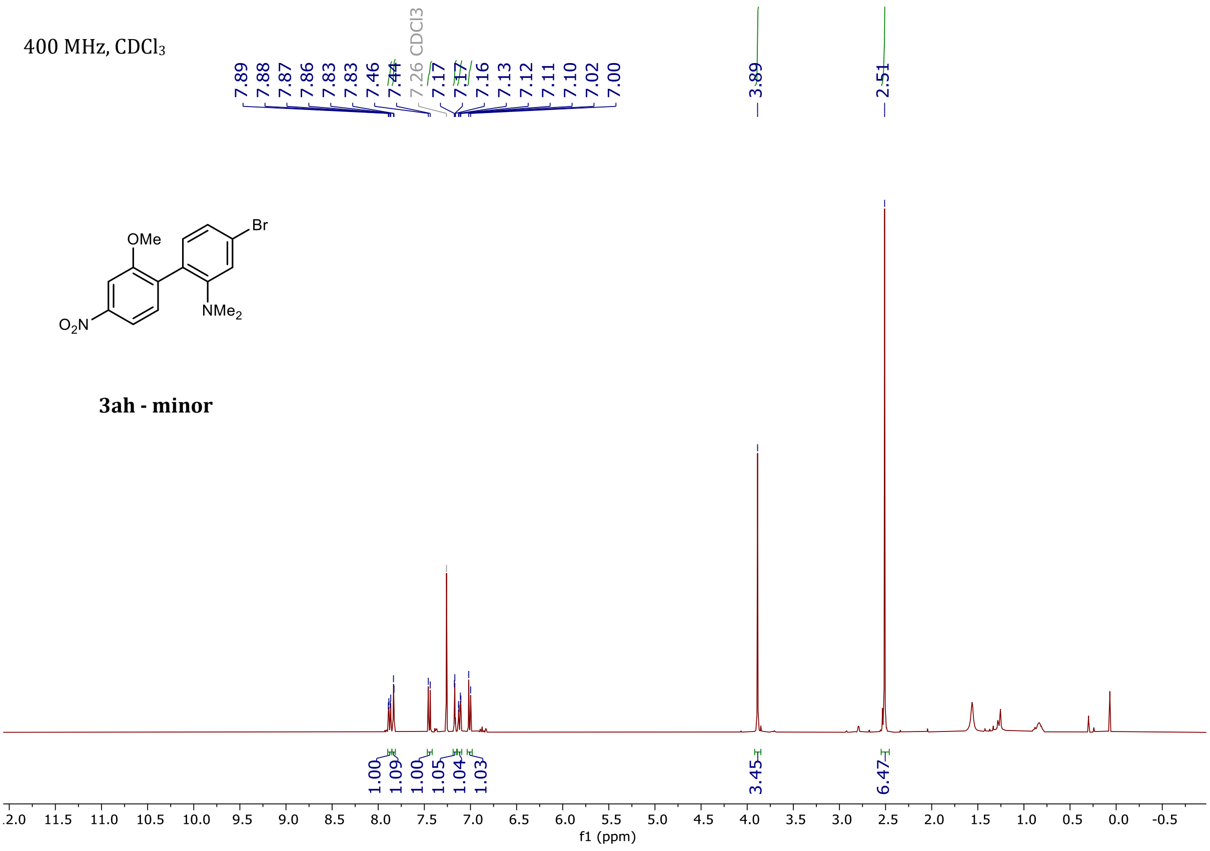
400 MHz, CDCl3
7.89
7.88
7.87
7.86
7.83
7.83
7.46
7.44
7.26 CDCl3
7.17
7.17
7.16
7.13
7.12
7.11
7.10
7.02
7.00
3.89
2.51
OMe
Br
O2N
NMe2
3ah - minor
1.00
1.09
1.00
1.05
1.04
1.03
3.45
6.47
f1 (ppm)

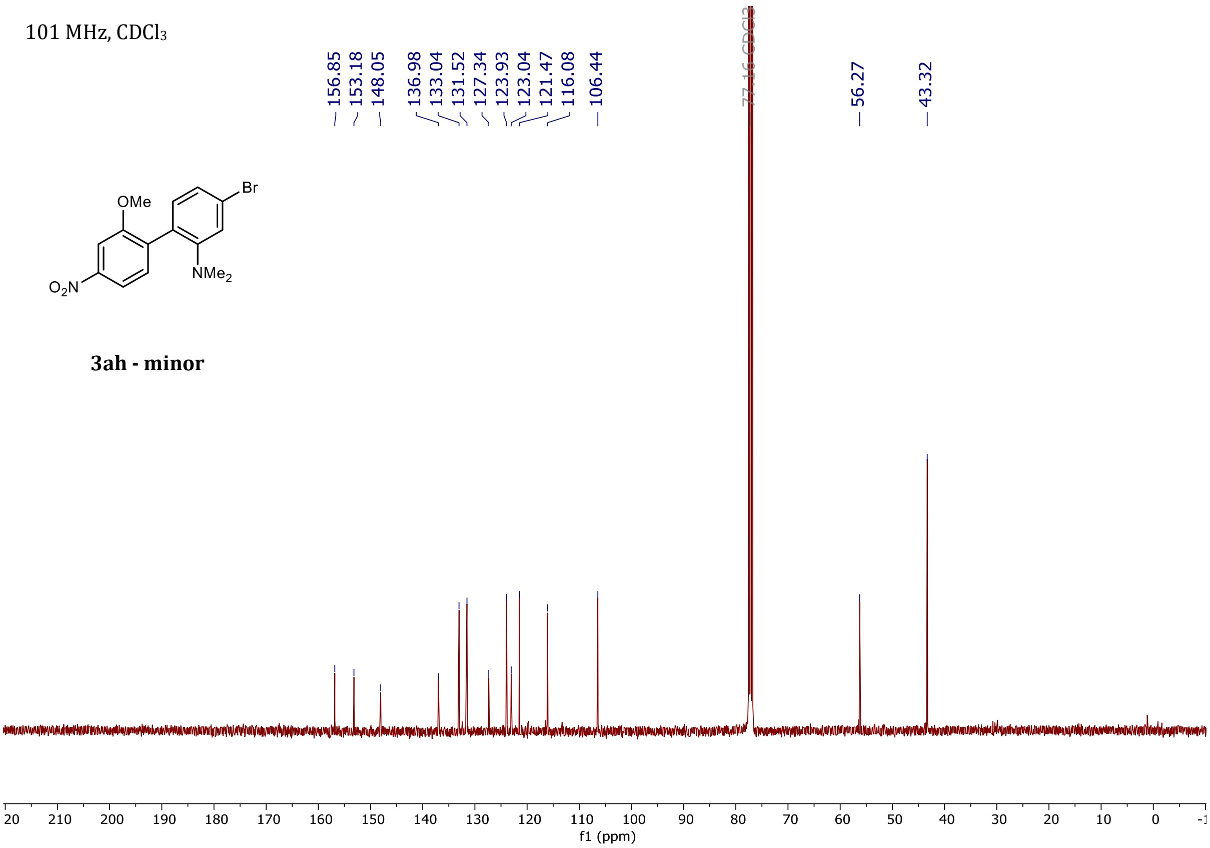
101 MHz, CDCl3
156.85
153.18
148.05
136.98
133.04
131.52
127.34
123.93
123.04
121.47
116.08
106.44
77.16 CDCl3
56.27
43.32
OMe
Br
O2N
NMe2
3ah - minor
f1 (ppm)

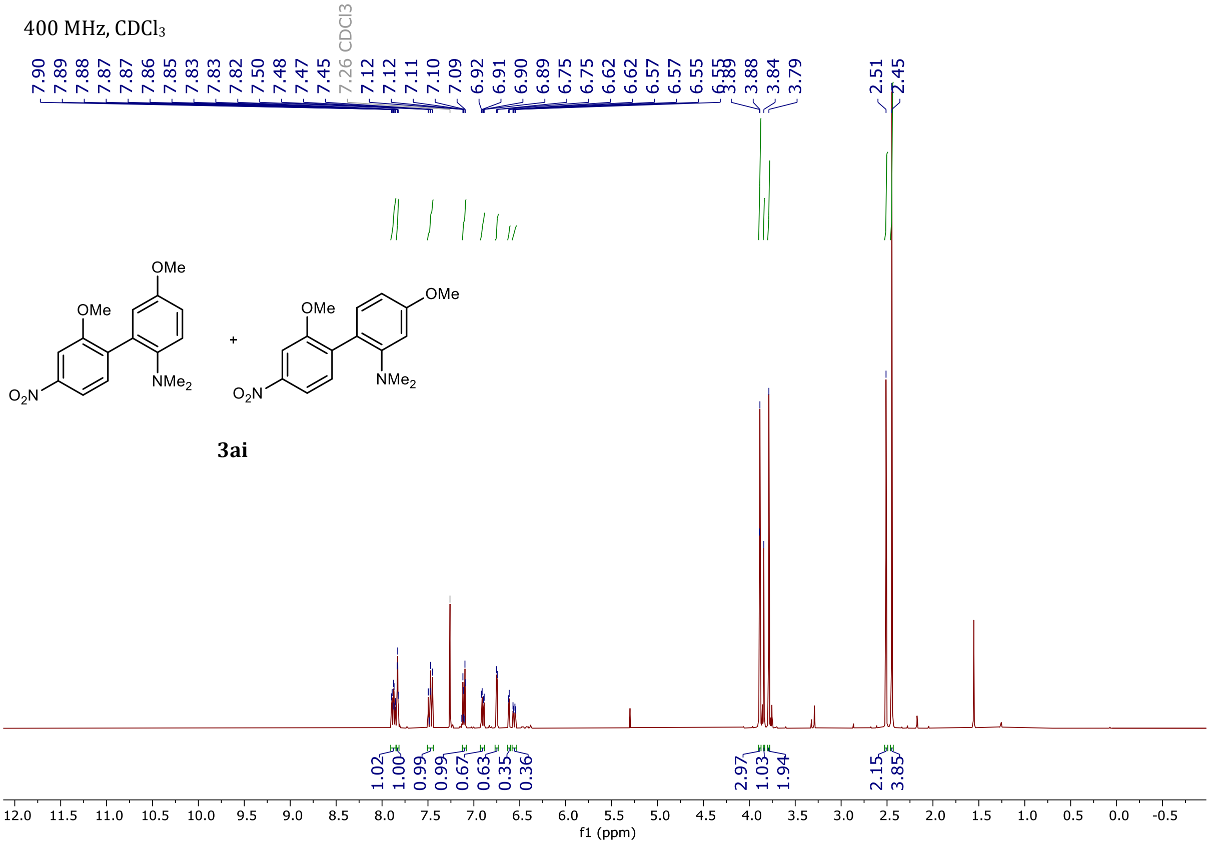
400 MHz, CDCl3
7.90 7.89 7.88 7.87 7.87 7.86 7.85 7.83 7.83 7.82 7.50 7.48 7.47 7.45 7.26 CDCl3 7.12 7.12 7.11 7.10 7.09 6.92 6.91 6.90 6.89 6.75 6.75 6.62 6.62 6.62 6.57 6.57 6.55 6.55 3.89 3.88 3.84 3.79 2.51 2.45
OMe
OMe
NMe2
O2N
+
OMe
OMe
NMe2
O2N
3ai
1.02 1.00 0.99 0.99 0.67 0.63 0.35 0.36 2.97 1.03 1.94 2.15 3.85
f1 (ppm)

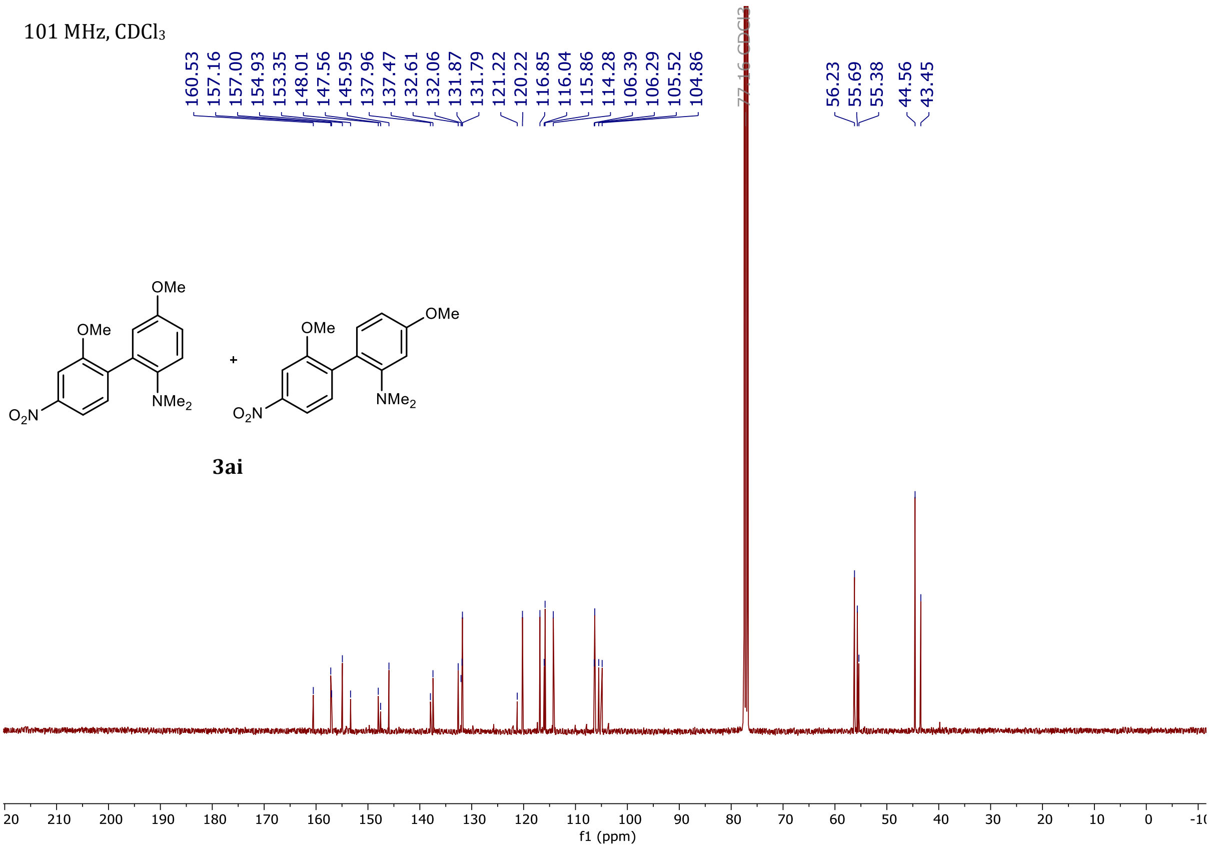
101 MHz, CDCl3
160.53 157.16 157.00 154.93 153.35 148.01 147.56 145.95 137.96 137.47 132.61 132.06 131.87 131.79 121.22 120.22 116.85 116.04 115.86 114.28 106.39 106.29 105.52 104.86 77.16 CDCl3 56.23 55.69 55.38 44.56 43.45
OMe
OMe
NMe2
O2N
+
OMe
OMe
NMe2
O2N
3ai
f1 (ppm)

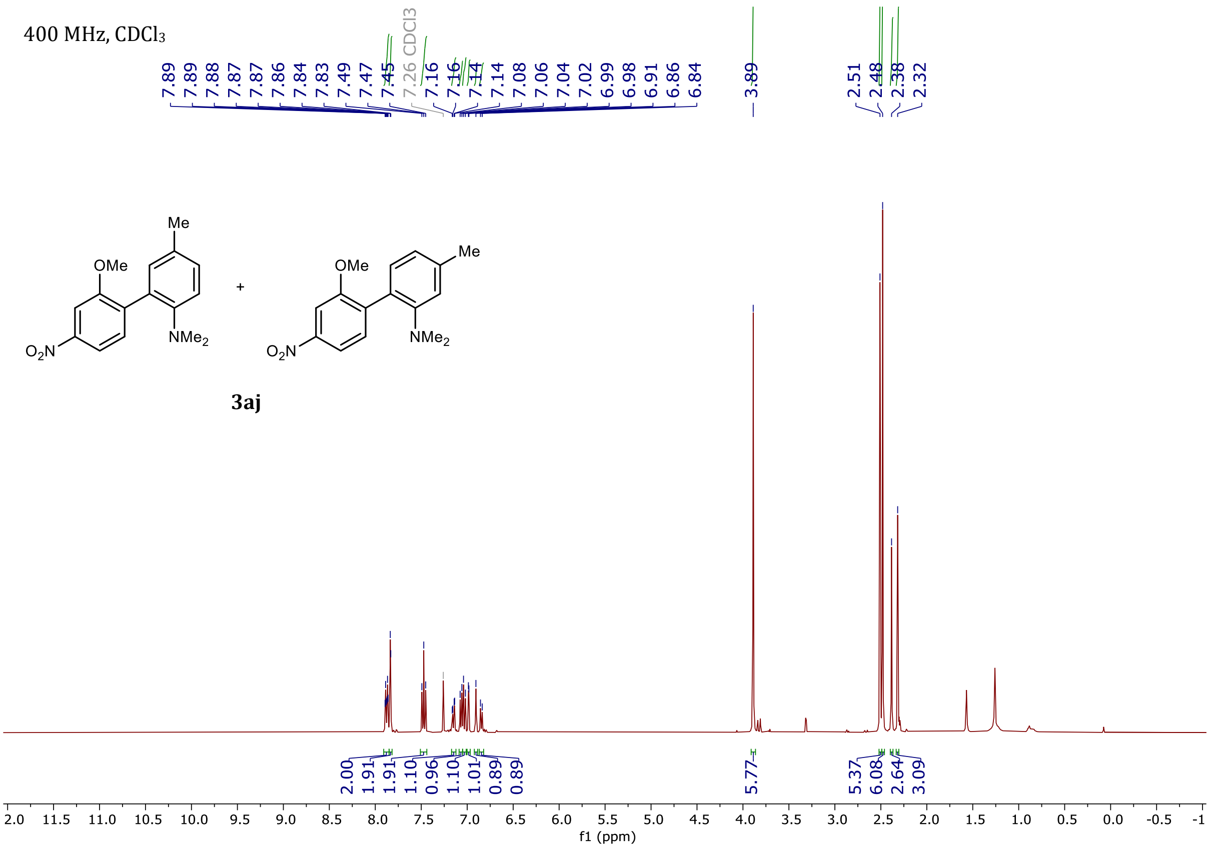
400 MHz, CDCl3
7.89 7.89 7.88 7.87 7.87 7.86 7.84 7.83 7.49 7.47 7.45 7.26 CDCl3 7.16 7.15 7.14 7.14 7.08 7.06 7.04 7.02 6.99 6.98 6.91 6.86 6.84
3.89
2.51 2.48 2.38 2.32
OMe
Me
O2N
NMe2
+
3aj
2.00 1.91 1.91 1.10 0.96 1.10 1.01 0.89 0.89
5.77
5.37 6.08 2.64 3.09
f1 (ppm)

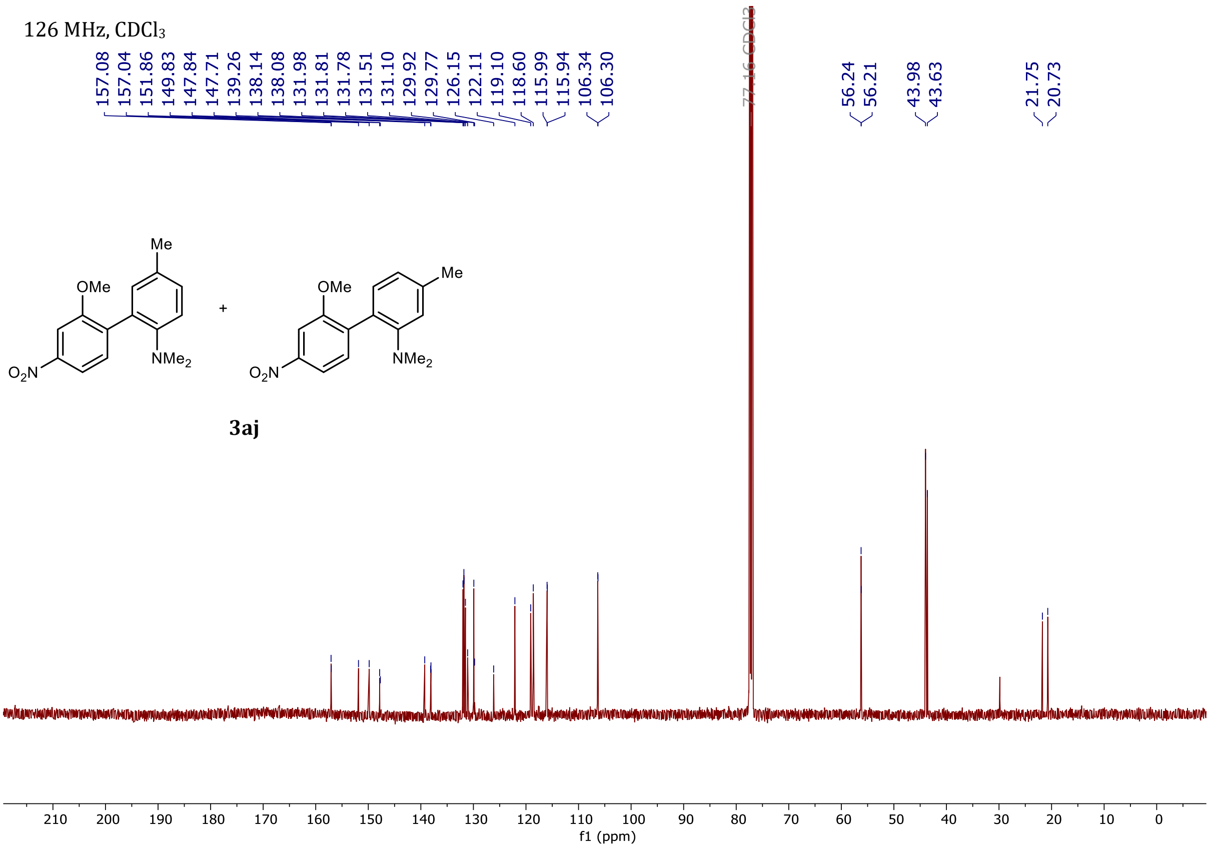
126 MHz, CDCl3
157.08 157.04 151.86 149.83 147.84 147.71 139.26 138.14 138.08 131.98 131.81 131.78 131.51 131.10 129.92 129.77 126.15 122.11 119.10 118.60 115.99 115.94 106.34 106.30
77.16 CDCl3
56.24 56.21
43.98 43.63
21.75 20.73
OMe
Me
O2N
NMe2
+
3aj
f1 (ppm)

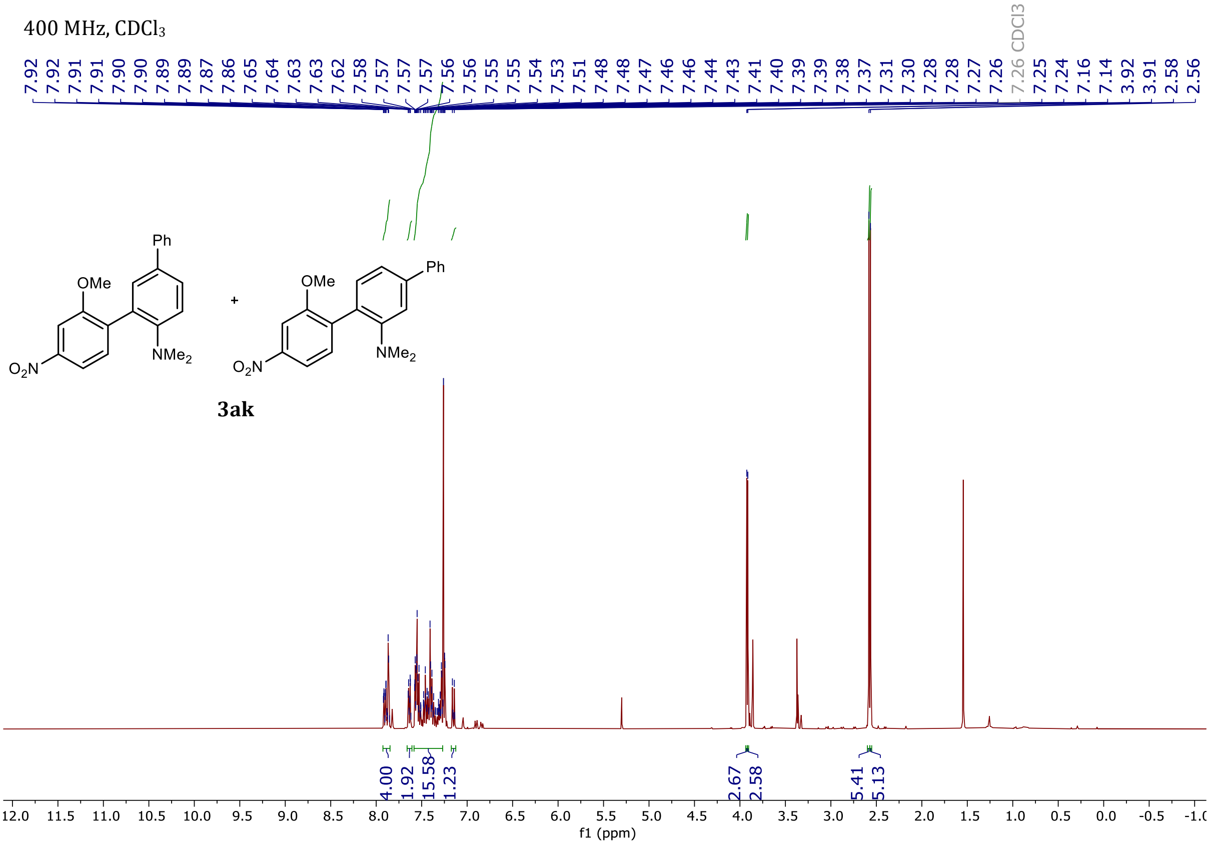
400 MHz, CDCl$_3$
7.92 7.92 7.91 7.91 7.90 7.90 7.89 7.89 7.87 7.86 7.65 7.64 7.63 7.63 7.62 7.58 7.57 7.57 7.57 7.56 7.56 7.55 7.55 7.54 7.53 7.51 7.48 7.48 7.47 7.46 7.46 7.44 7.43 7.41 7.40 7.39 7.39 7.38 7.37 7.31 7.30 7.28 7.28 7.27 7.26 7.26 CDCl3 7.25 7.24 7.16 7.14 3.92 3.91 2.58 2.56
Ph
OMe
O$_2$N
NMe$_2$
+
3ak
4.00 1.92 15.58 1.23 2.67 2.58 5.41 5.13
f1 (ppm)

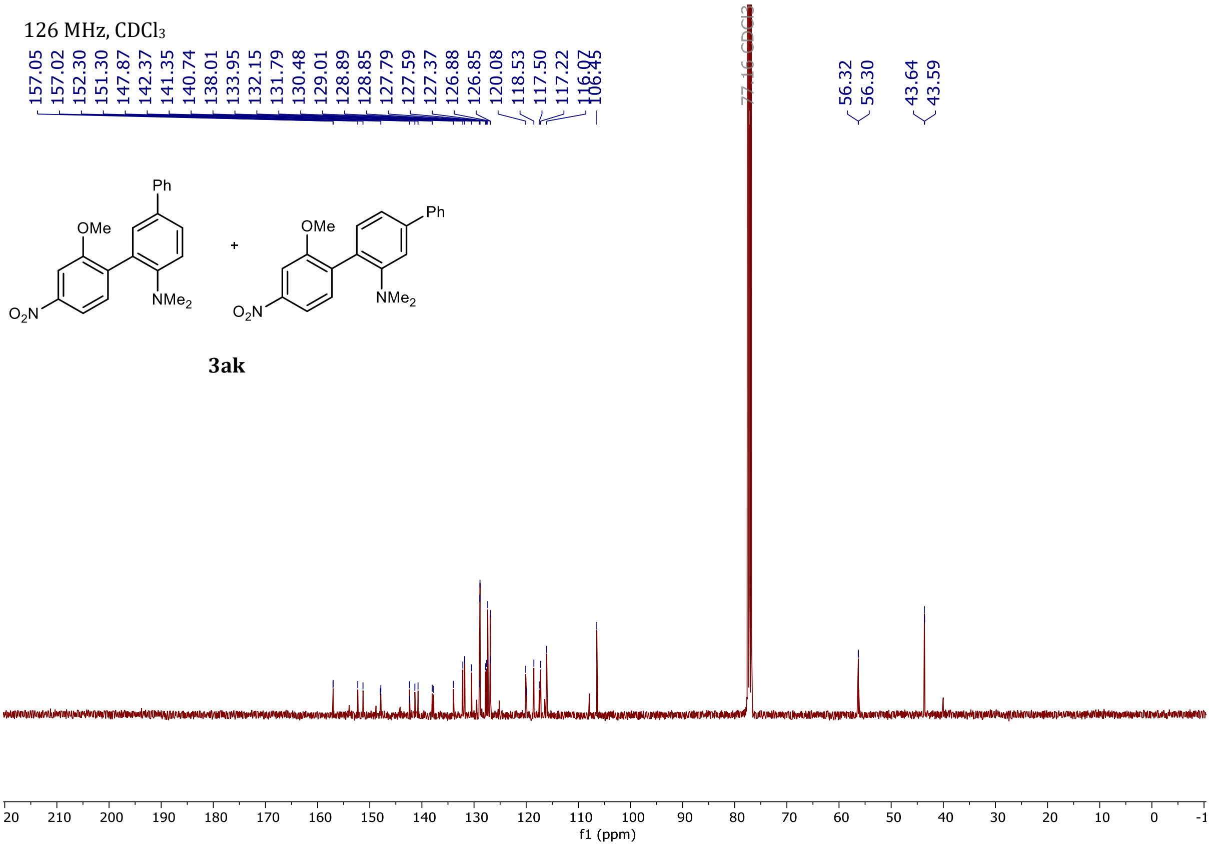
126 MHz, CDCl$_3$
157.05 157.02 152.30 151.30 147.87 142.37 141.35 140.74 138.01 133.95 132.15 131.79 130.48 129.01 128.89 128.85 127.79 127.59 127.37 126.88 126.85 120.08 118.53 117.50 117.22 116.97 106.45
77.16 CDCl3
56.32 56.30 43.64 43.59
Ph
OMe
O$_2$N
NMe$_2$
+
3ak
f1 (ppm)

400 MHz, $CDCl_3$

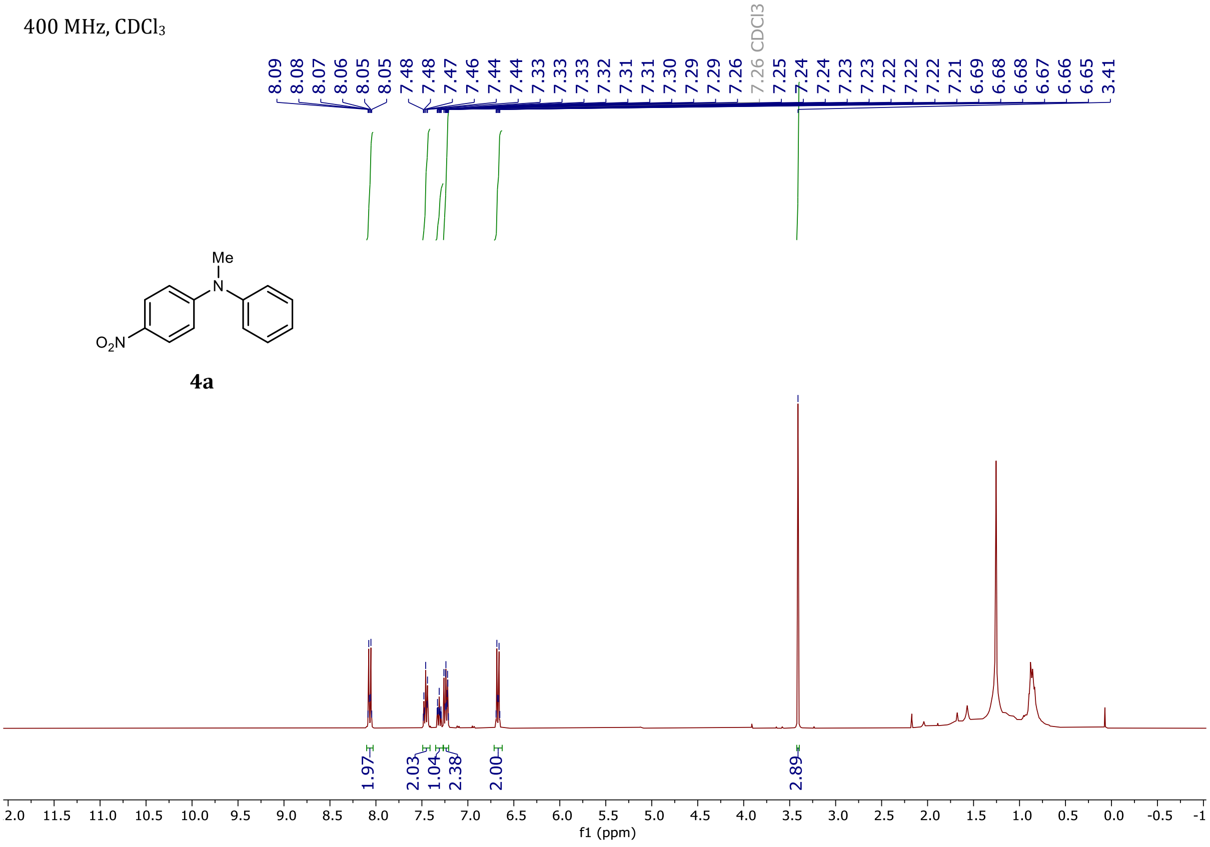

4a

101 MHz, $CDCl_3$

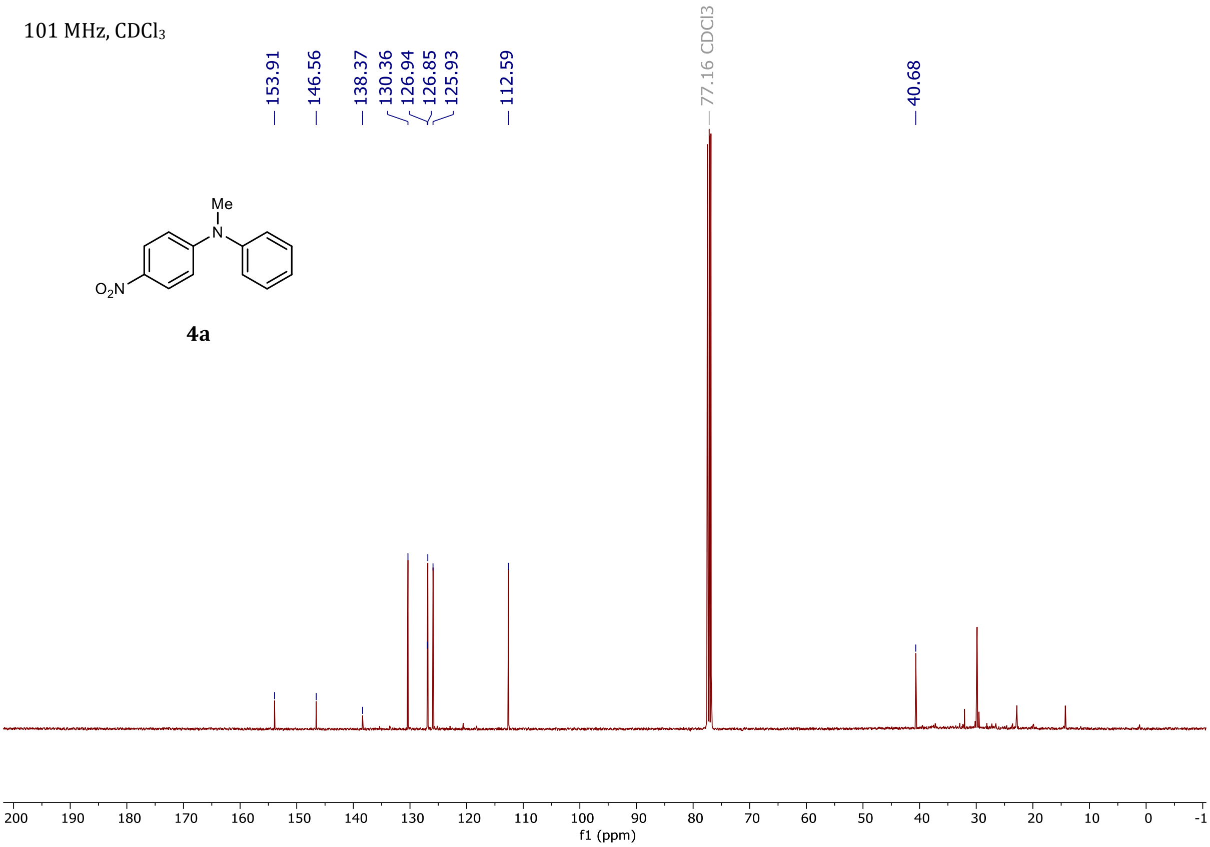

4a

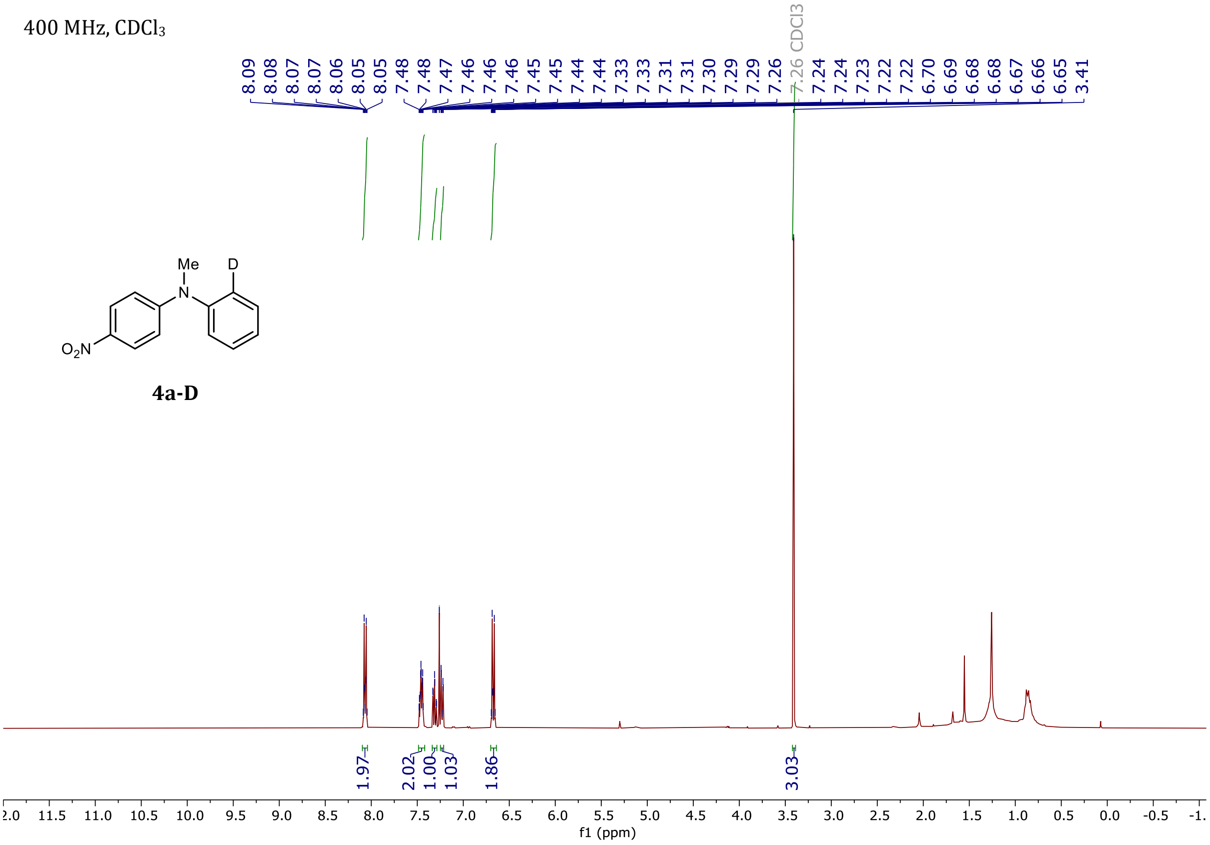
400 MHz, CDCl3
8.09 8.08 8.07 8.07 8.06 8.05 8.05 7.48 7.48 7.47 7.46 7.46 7.46 7.45 7.45 7.44 7.44 7.33 7.33 7.31 7.31 7.30 7.29 7.29 7.26 7.26 CDCl3 7.24 7.24 7.23 7.22 7.22 6.70 6.69 6.68 6.68 6.67 6.66 6.65 3.41
Me
D
N
O2N
4a-D
1.97
2.02
1.00
1.03
1.86
3.03
2.0 11.5 11.0 10.5 10.0 9.5 9.0 8.5 8.0 7.5 7.0 6.5 6.0 5.5 5.0 4.5 4.0 3.5 3.0 2.5 2.0 1.5 1.0 0.5 0.0 -0.5 -1.
f1 (ppm)

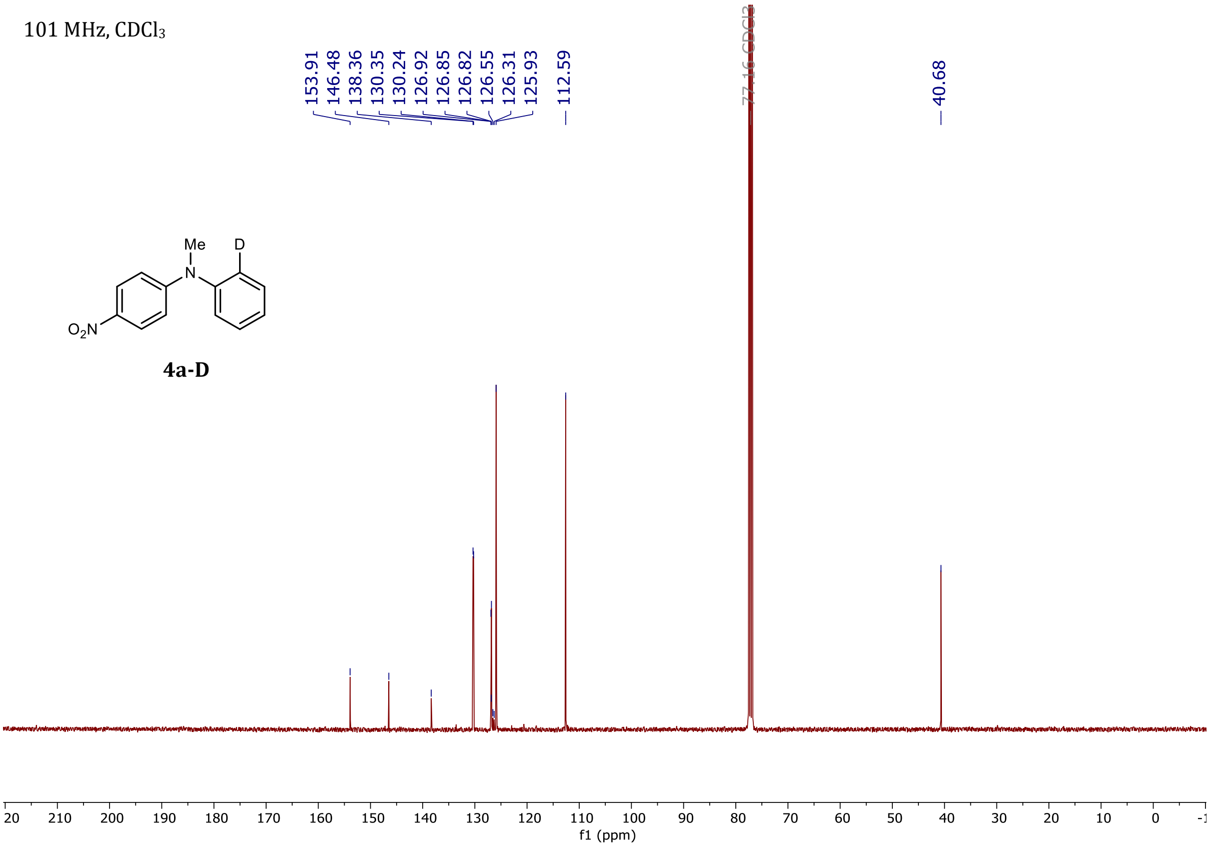
101 MHz, CDCl3
153.91 146.48 138.36 130.35 130.24 126.92 126.85 126.82 126.55 126.31 125.93
112.59
77.16 CDCl3
40.68
Me
D
N
O2N
4a-D
20 210 200 190 180 170 160 150 140 130 120 110 100 90 80 70 60 50 40 30 20 10 0 -1
f1 (ppm)

500 MHz, CDCl₃

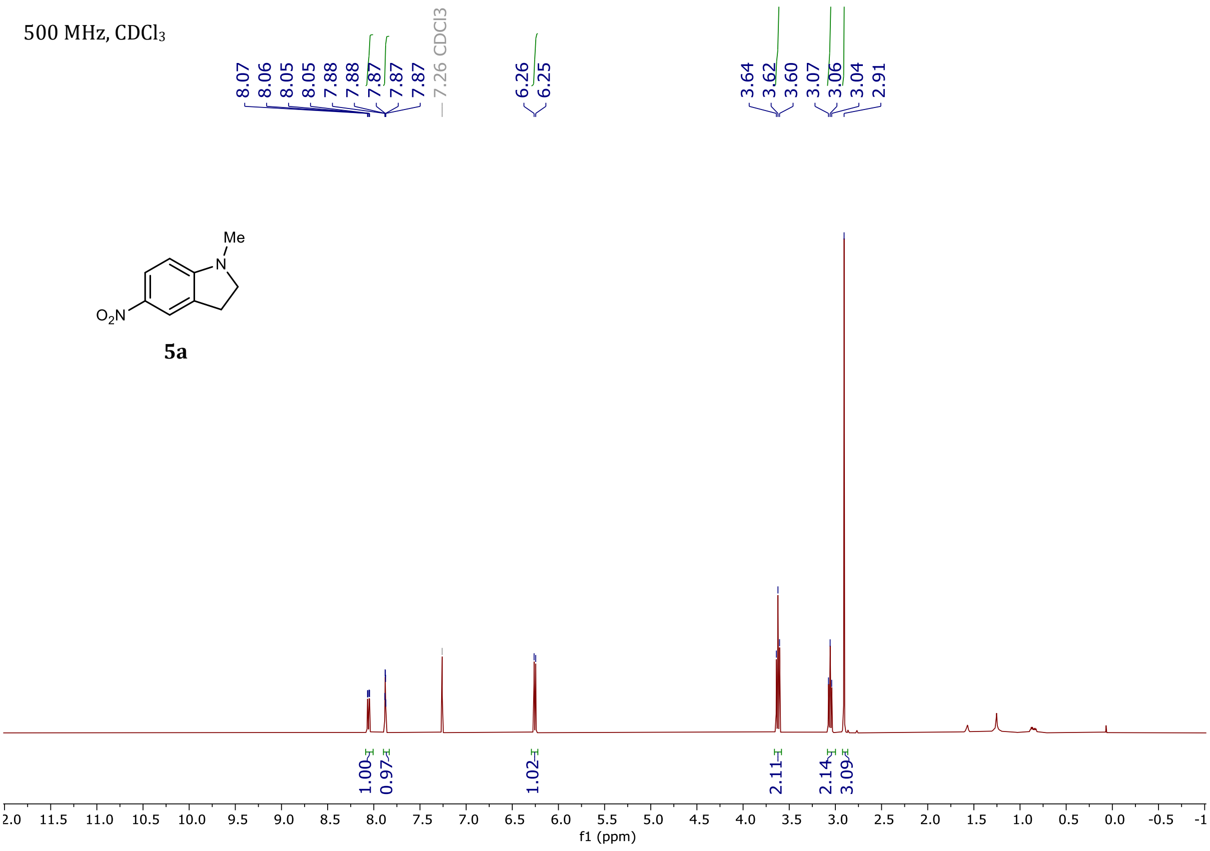

5a

126 MHz, CDCl₃

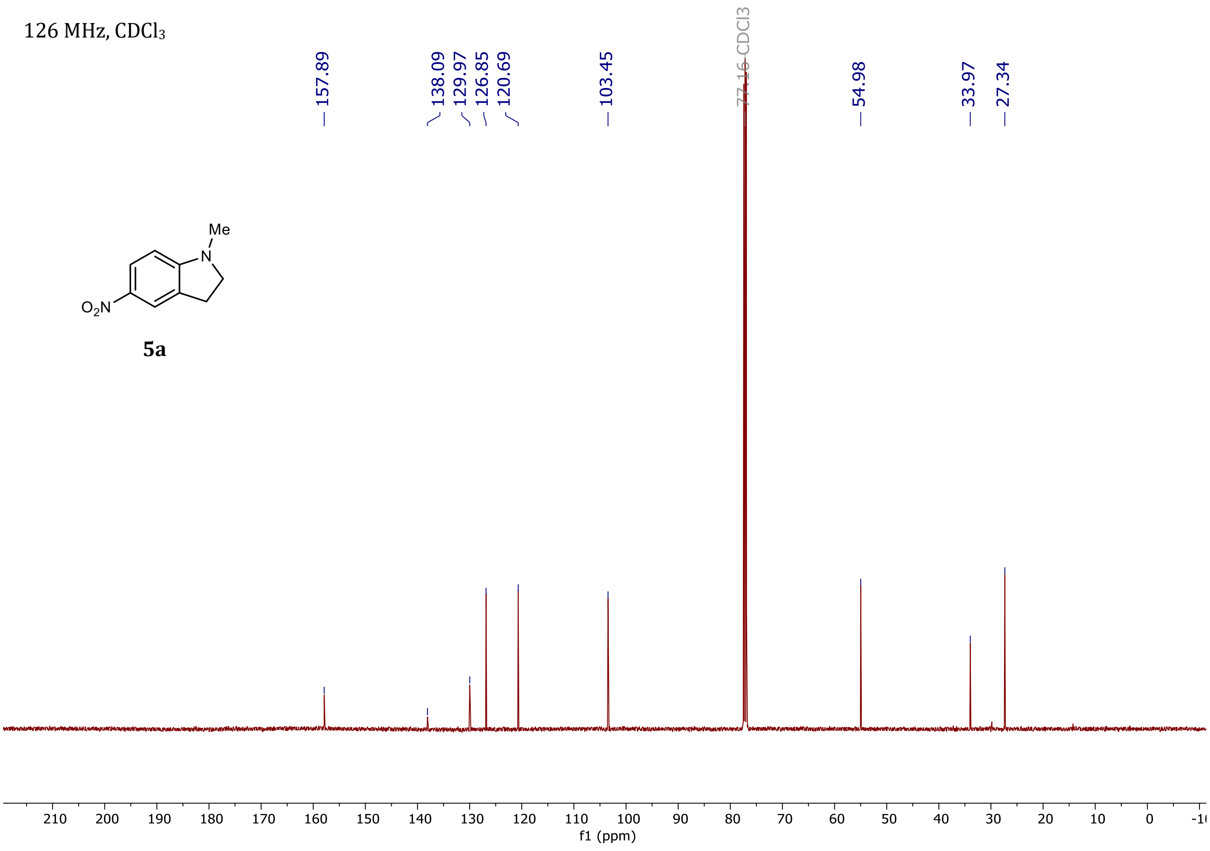

5a

400 MHz, $CDCl_3$

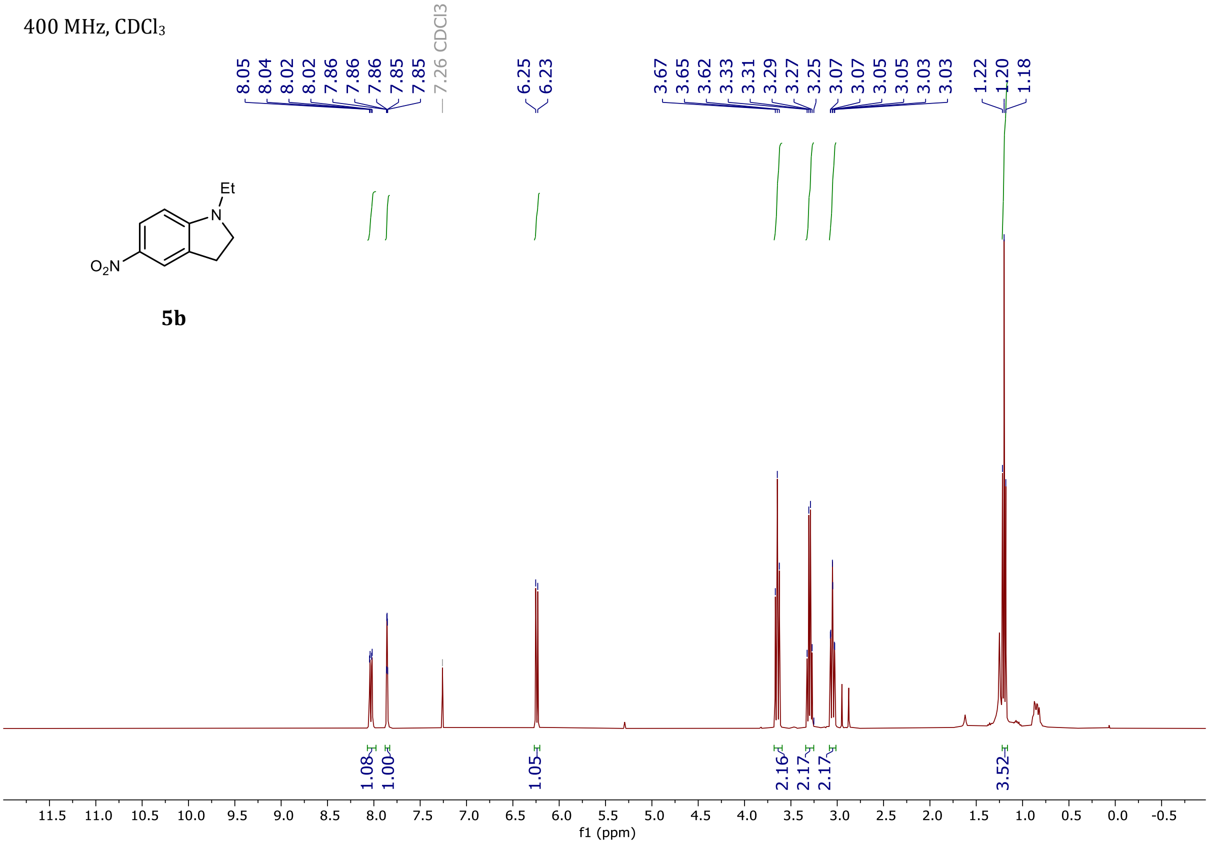

5b

126 MHz, $CDCl_3$

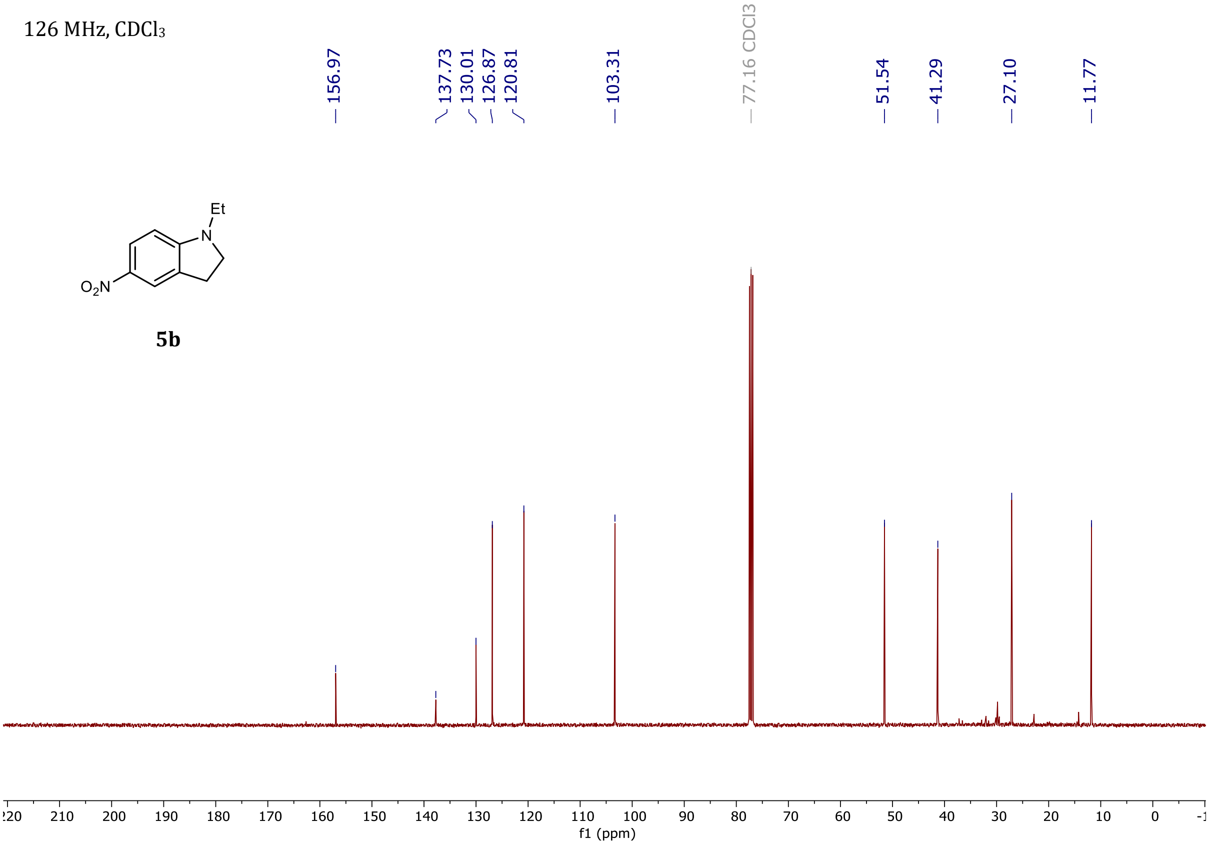

5b

500 MHz, $CDCl_3$

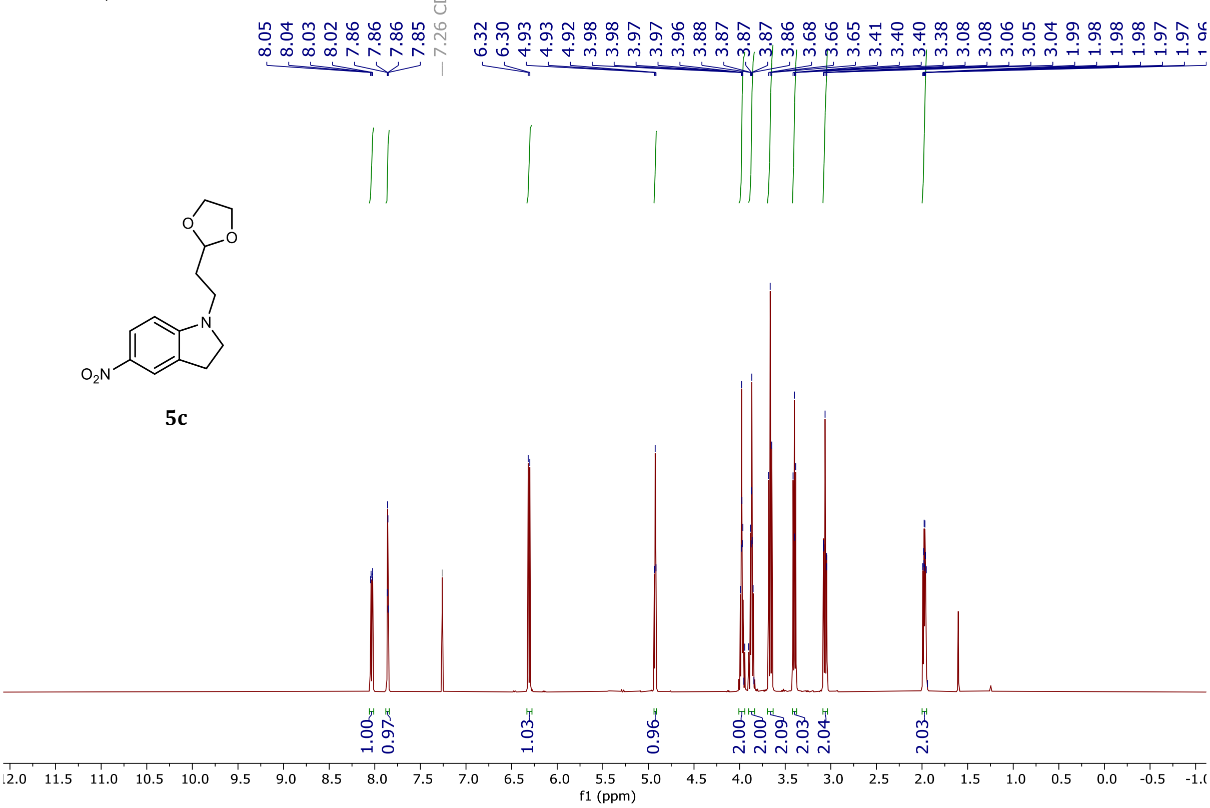

126 MHz, $CDCl_3$

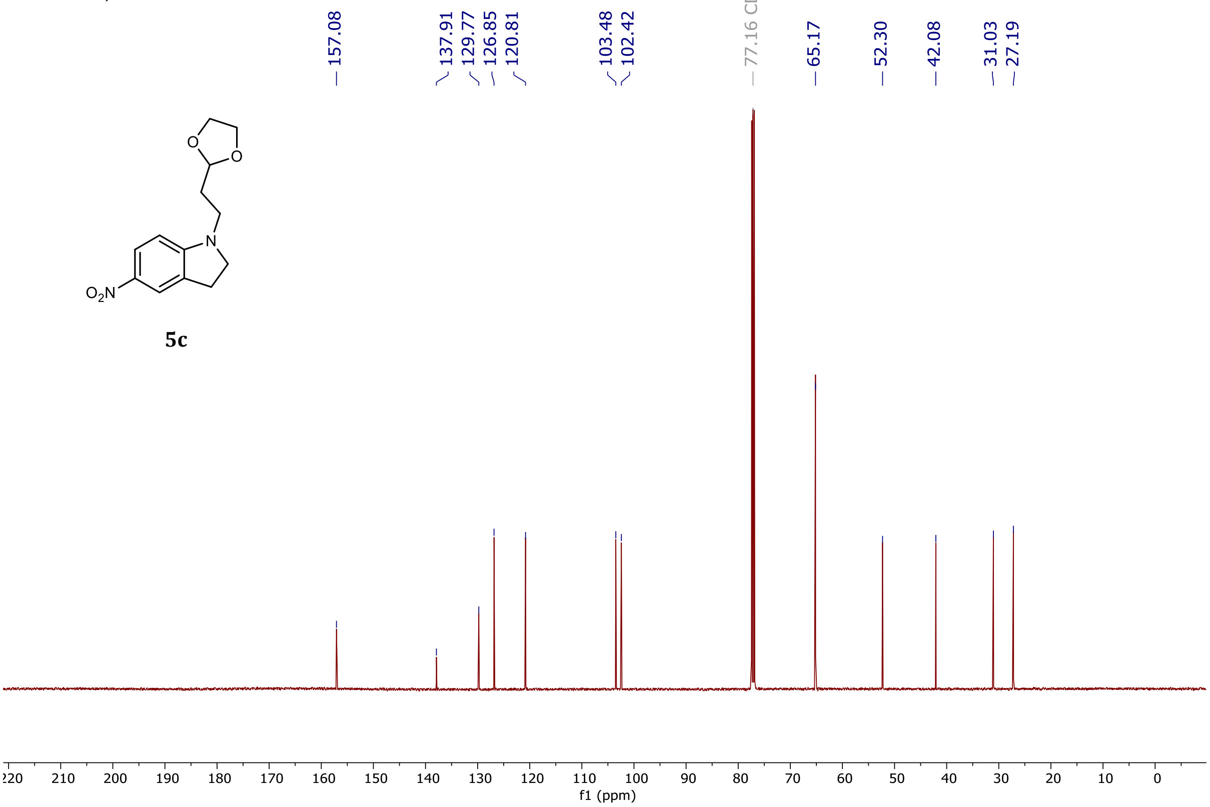

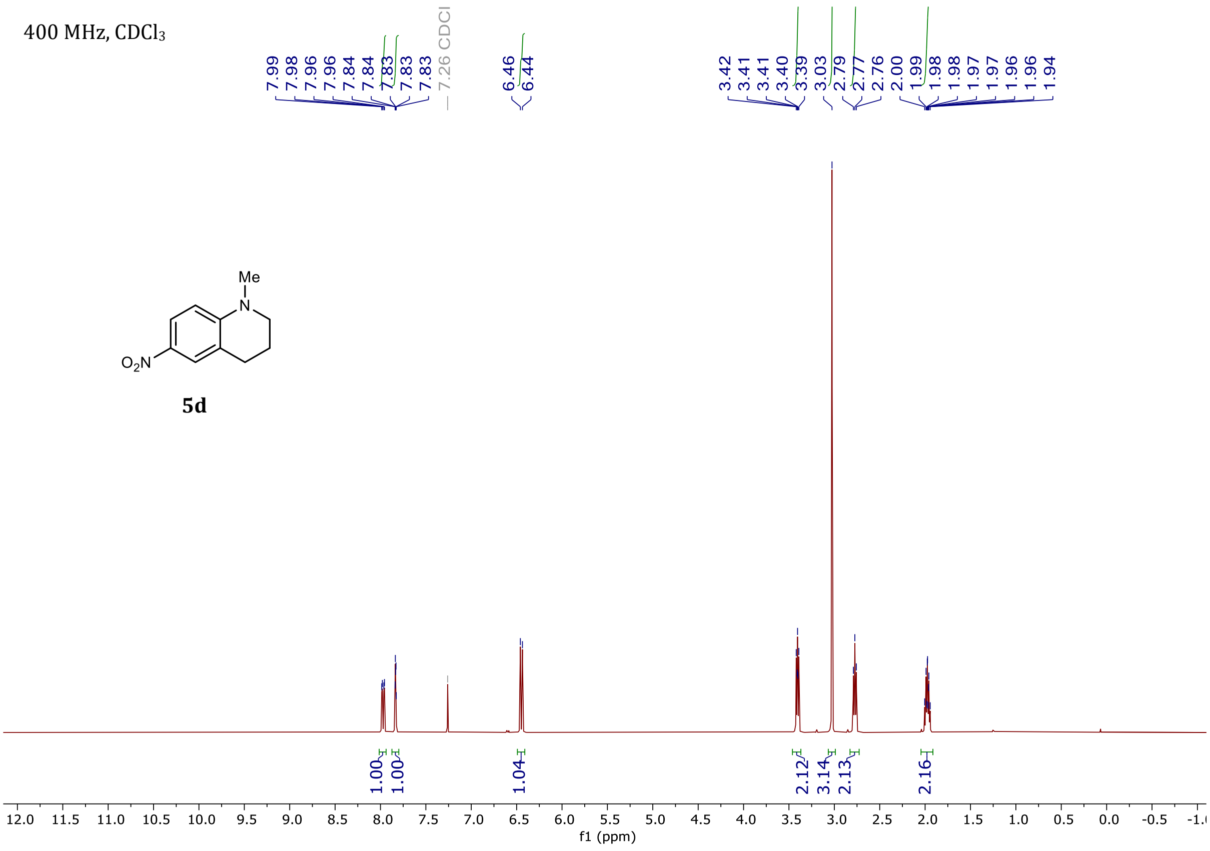

400 MHz, CDCl3
7.99
7.98
7.96
7.84
7.84
7.83
7.83
7.83
7.26 CDCl
6.46
6.44
3.42
3.41
3.41
3.40
3.39
3.03
2.79
2.77
2.76
2.00
1.99
1.98
1.98
1.97
1.97
1.96
1.96
1.94
Me
N
O2N
1.00
1.00
1.04
2.12
3.14
2.13
2.16
f1 (ppm)

5d

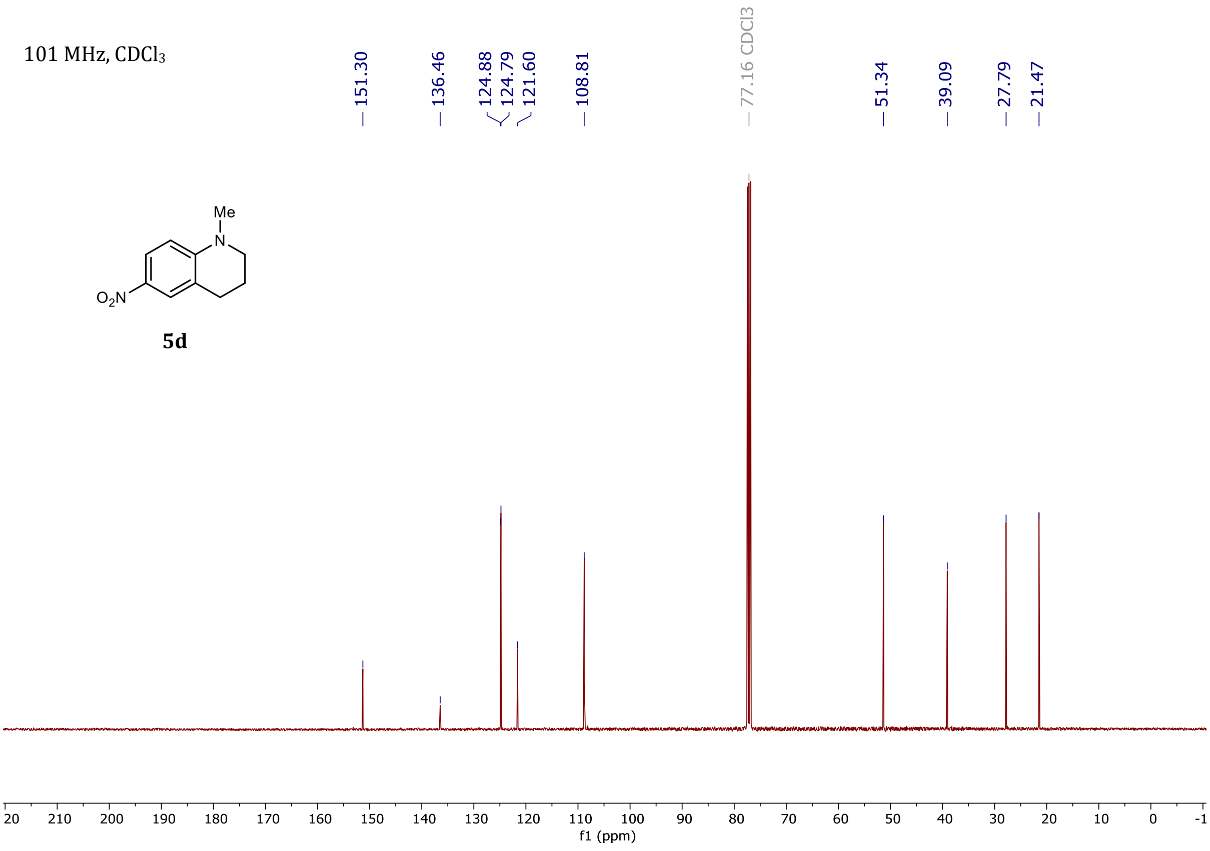

101 MHz, CDCl3
151.30
136.46
124.88
124.79
121.60
108.81
77.16 CDCl3
51.34
39.09
27.79
21.47
Me
N
O2N
f1 (ppm)

5d

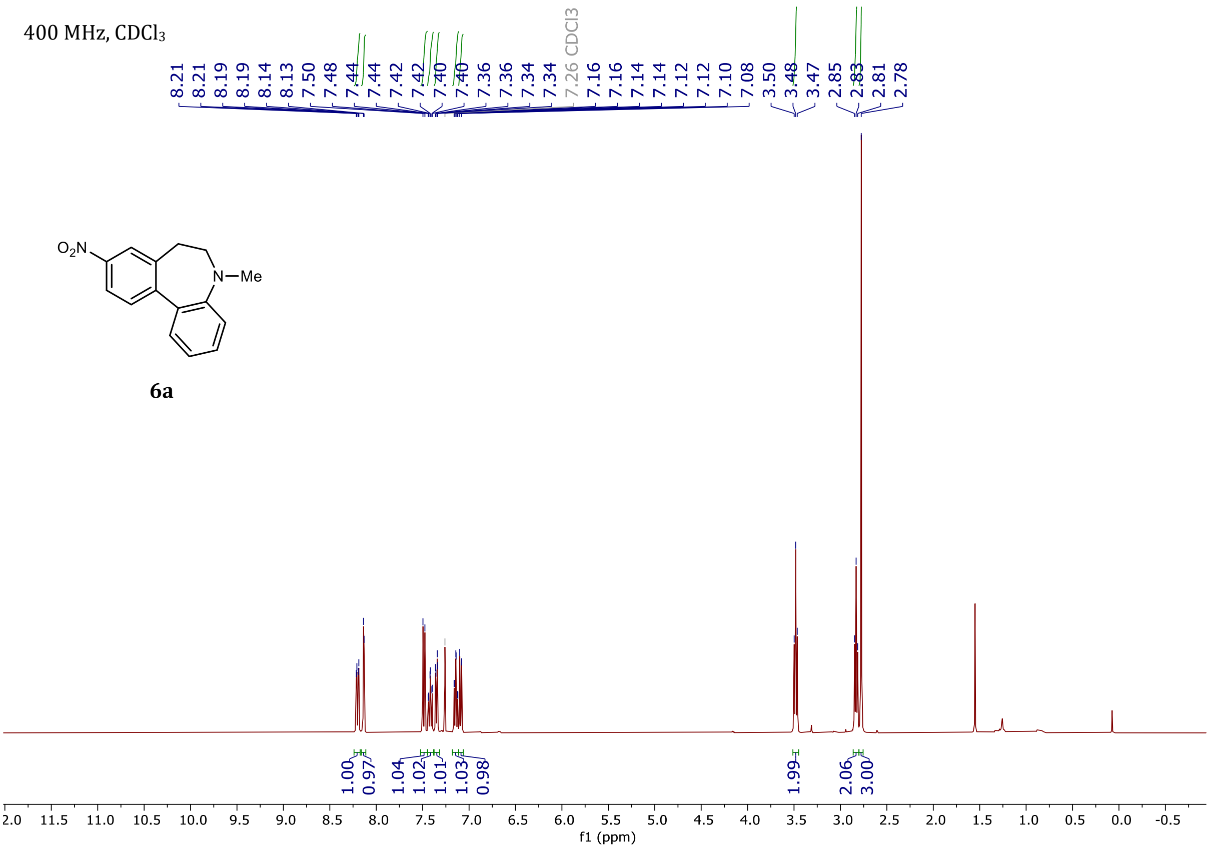
400 MHz, CDCl$_3$
8.21 8.21 8.19 8.19 8.14 8.13 7.50 7.48 7.44 7.44 7.42 7.42 7.40 7.40 7.36 7.36 7.34 7.34 7.26 CDCl3 7.16 7.16 7.14 7.14 7.12 7.12 7.10 7.08 3.50 3.48 3.47 2.85 2.83 2.81 2.78
O$_2$N
N–Me
6a
1.00 0.97 1.04 1.02 1.01 1.03 0.98 1.99 2.06 3.00
f1 (ppm)

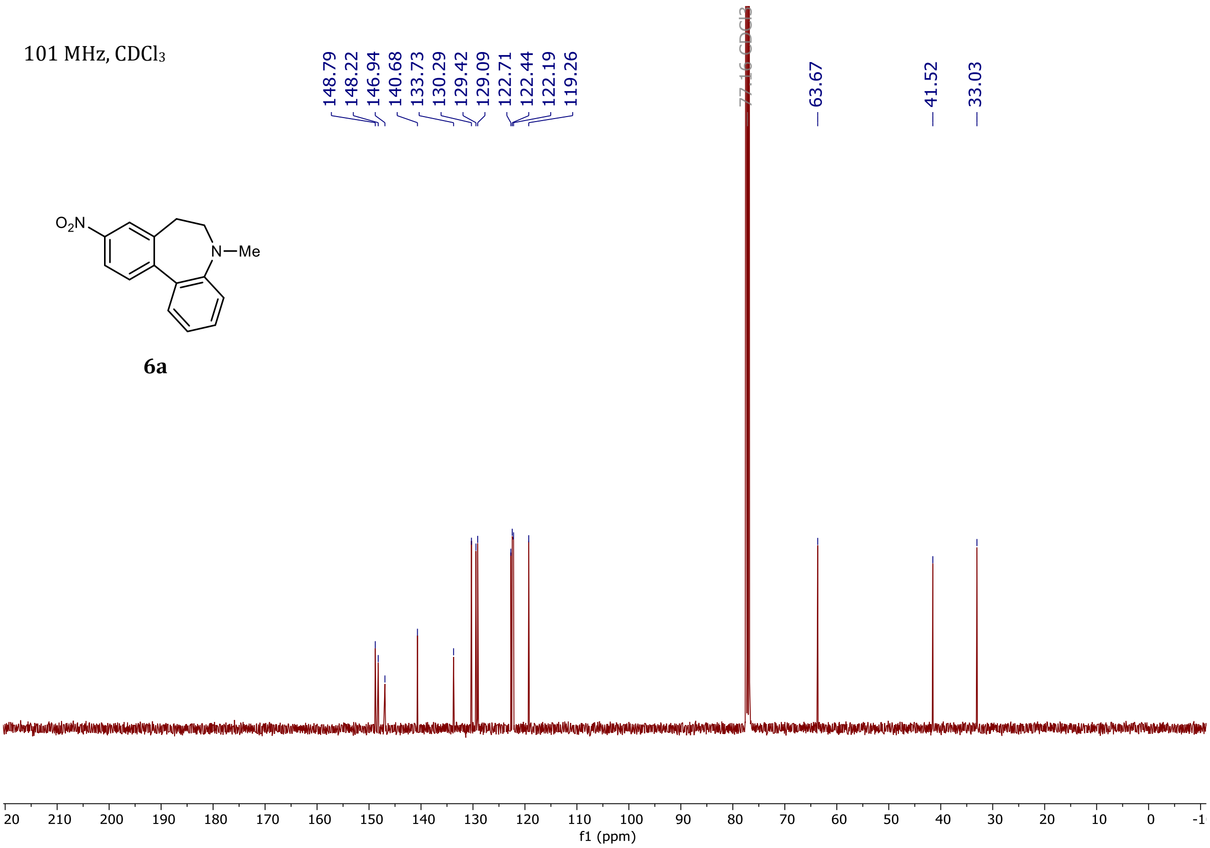
101 MHz, CDCl$_3$
148.79 148.22 146.94 140.68 133.73 130.29 129.42 129.09 122.71 122.44 122.19 119.26
77.16 CDCl3
63.67
41.52
33.03
O$_2$N
N–Me
6a
f1 (ppm)

400 MHz, $CDCl_3$

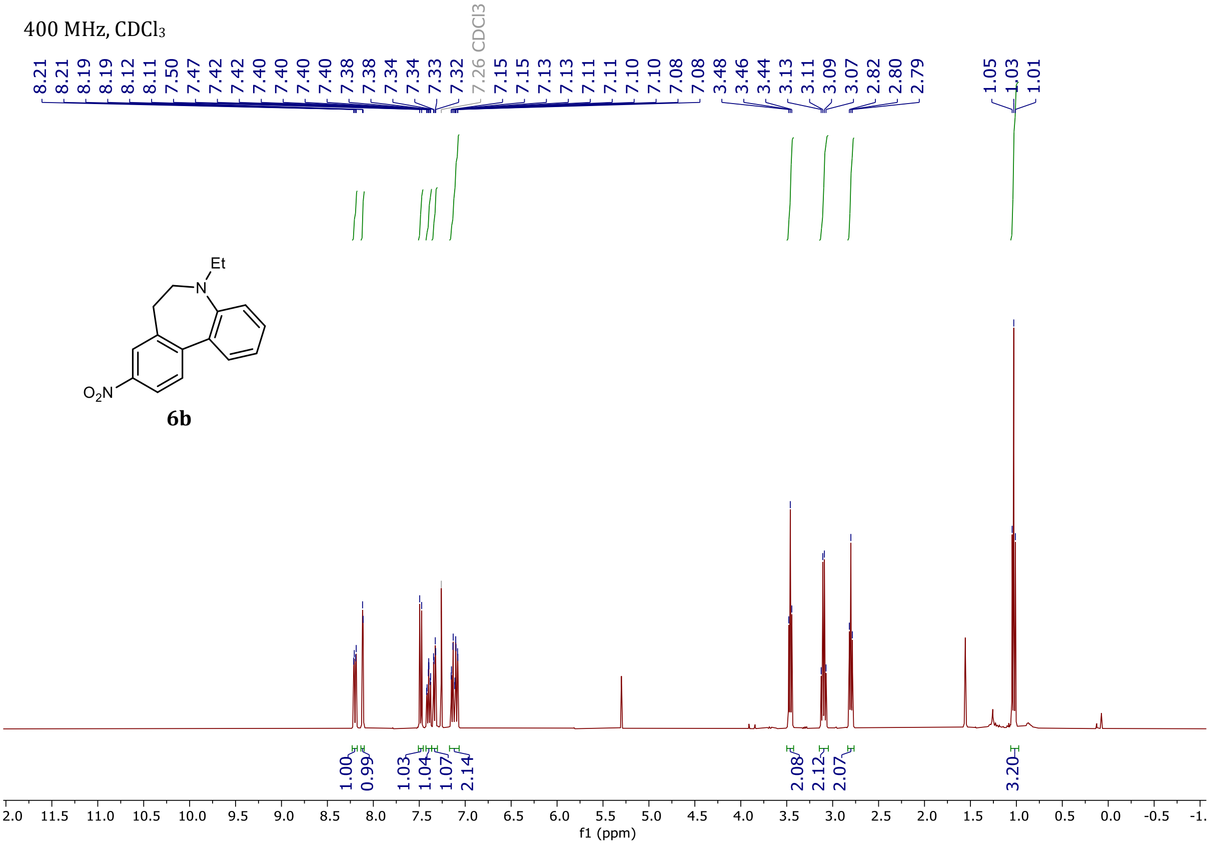

6b

101 MHz, $CDCl_3$

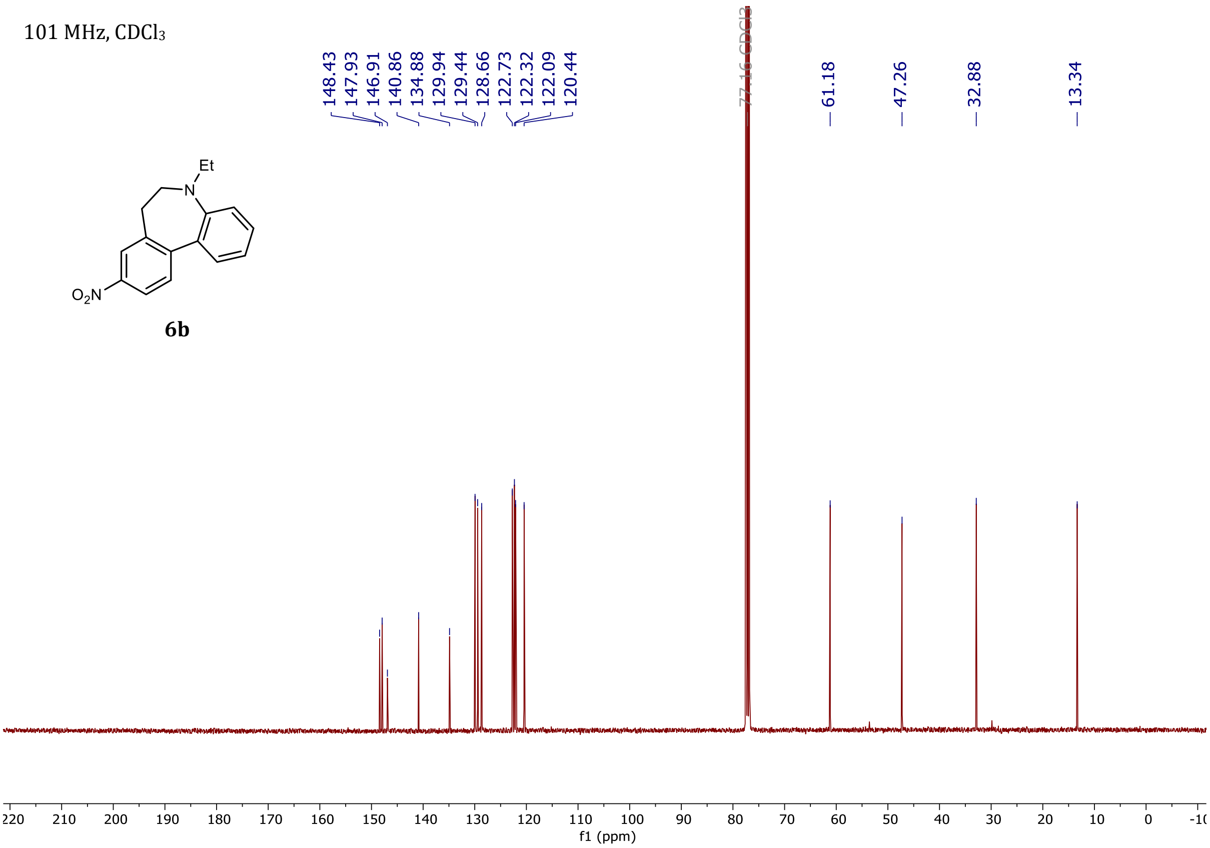

6b

400 MHz, $CDCl_3$

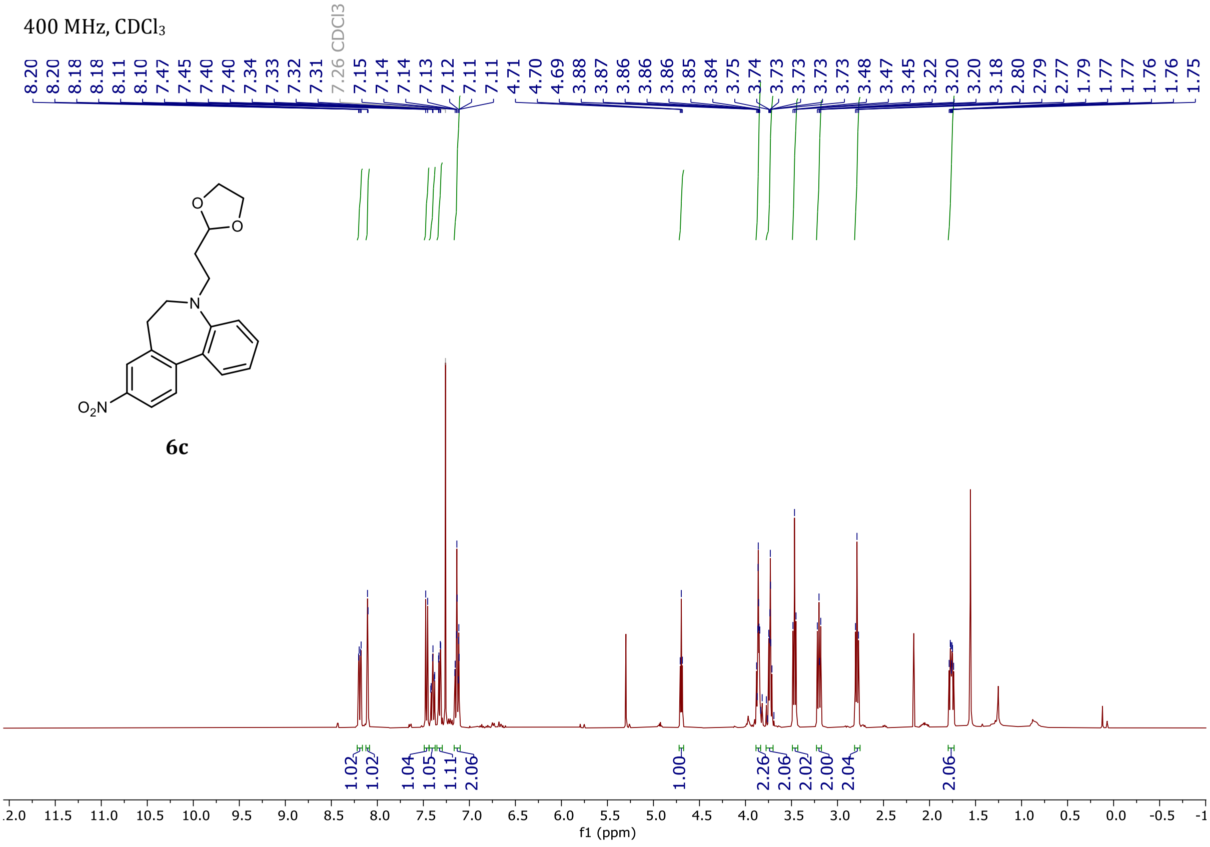

6c

101 MHz, $CDCl_3$

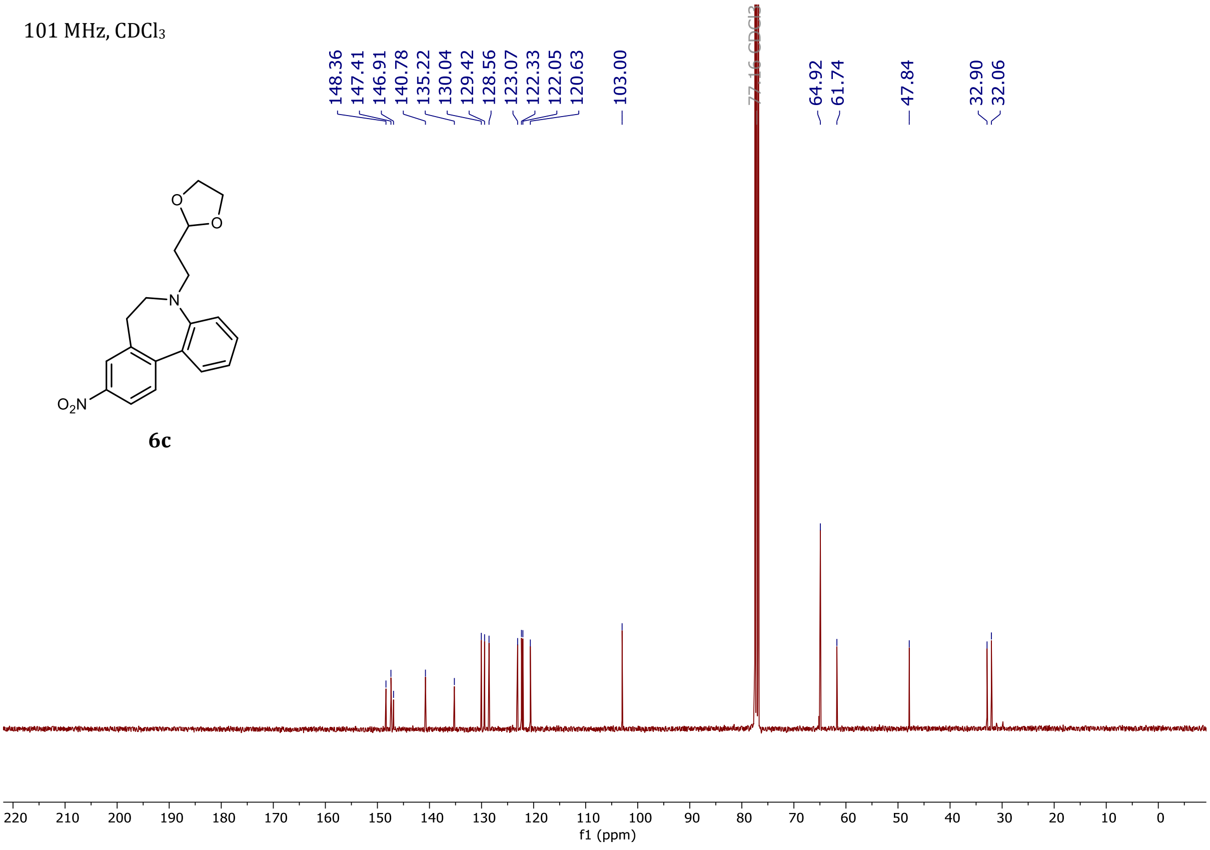

6c

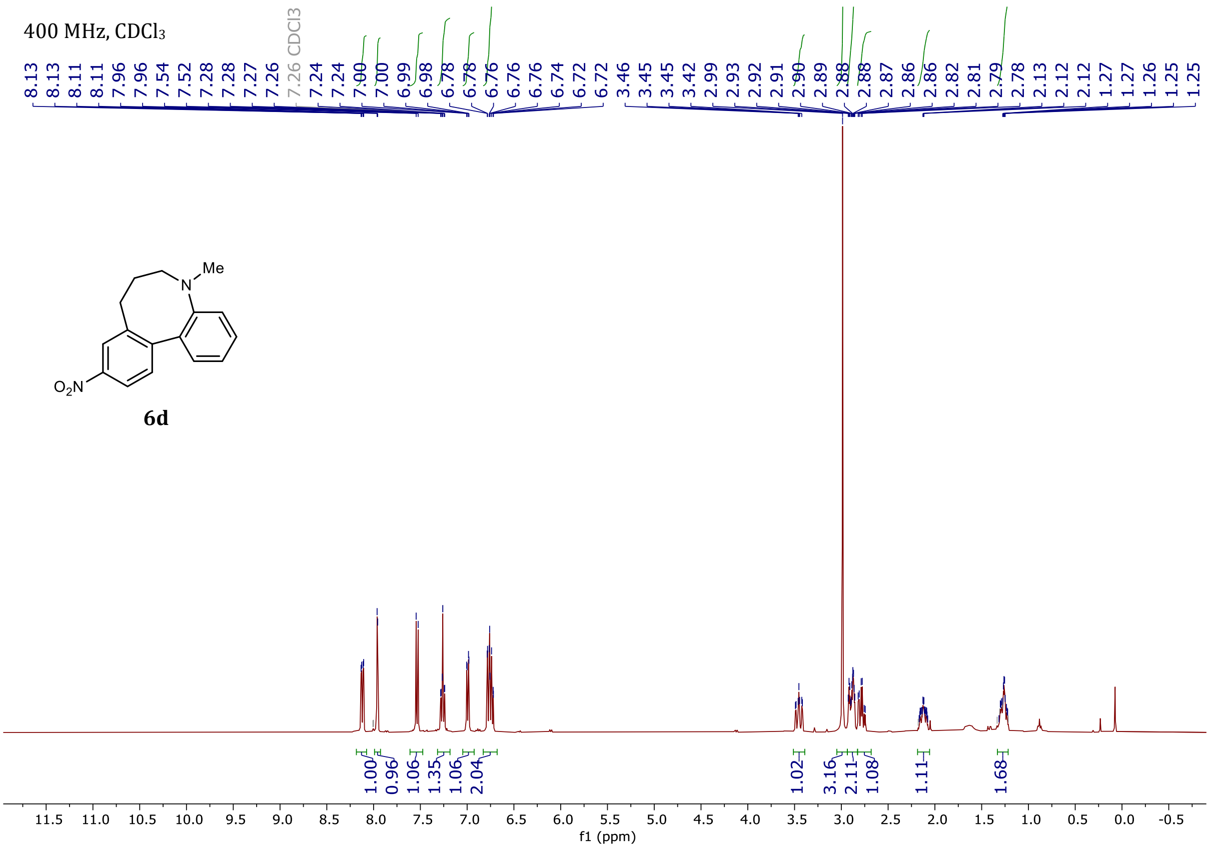
400 MHz, CDCl3
8.13 8.13 8.11 8.11 7.96 7.96 7.54 7.52 7.28 7.28 7.27 7.26 7.26 CDCl3 7.24 7.24 7.00 7.00 6.99 6.98 6.78 6.78 6.76 6.76 6.76 6.74 6.72 6.72
3.46 3.45 3.45 3.42 2.99 2.93 2.92 2.91 2.90 2.89 2.88 2.88 2.87 2.86 2.86 2.82 2.81 2.79 2.78 2.13 2.12 2.12 1.27 1.27 1.26 1.25 1.25
Me
N
O2N
6d
1.00 0.96 1.06 1.35 1.06 2.04 1.02 3.16 2.11 1.08 1.11 1.68
11.5 11.0 10.5 10.0 9.5 9.0 8.5 8.0 7.5 7.0 6.5 6.0 5.5 5.0 4.5 4.0 3.5 3.0 2.5 2.0 1.5 1.0 0.5 0.0 -0.5
f1 (ppm)

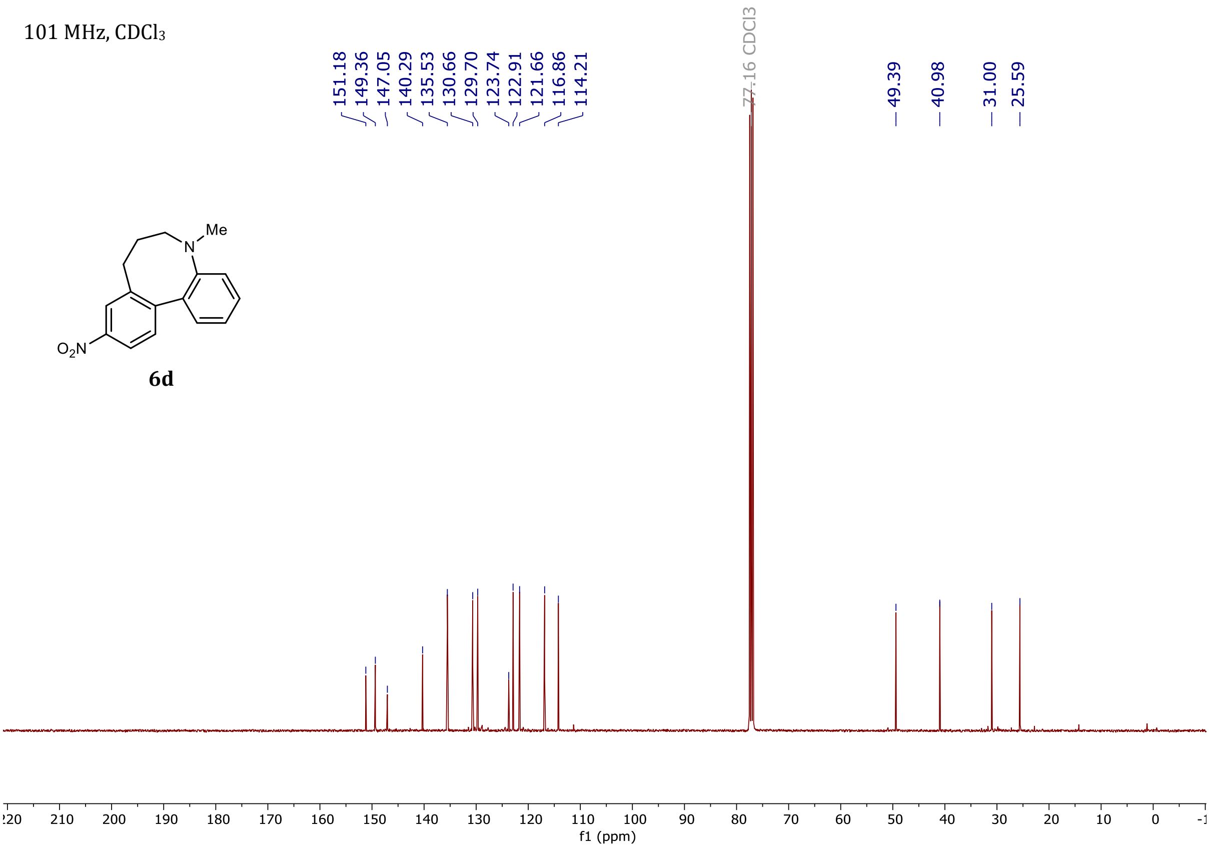
101 MHz, CDCl3
151.18 149.36 147.05 140.29 135.53 130.66 129.70 123.74 122.91 121.66 116.86 114.21
77.16 CDCl3
49.39 40.98 31.00 25.59
Me
N
O2N
6d
220 210 200 190 180 170 160 150 140 130 120 110 100 90 80 70 60 50 40 30 20 10 0
f1 (ppm)

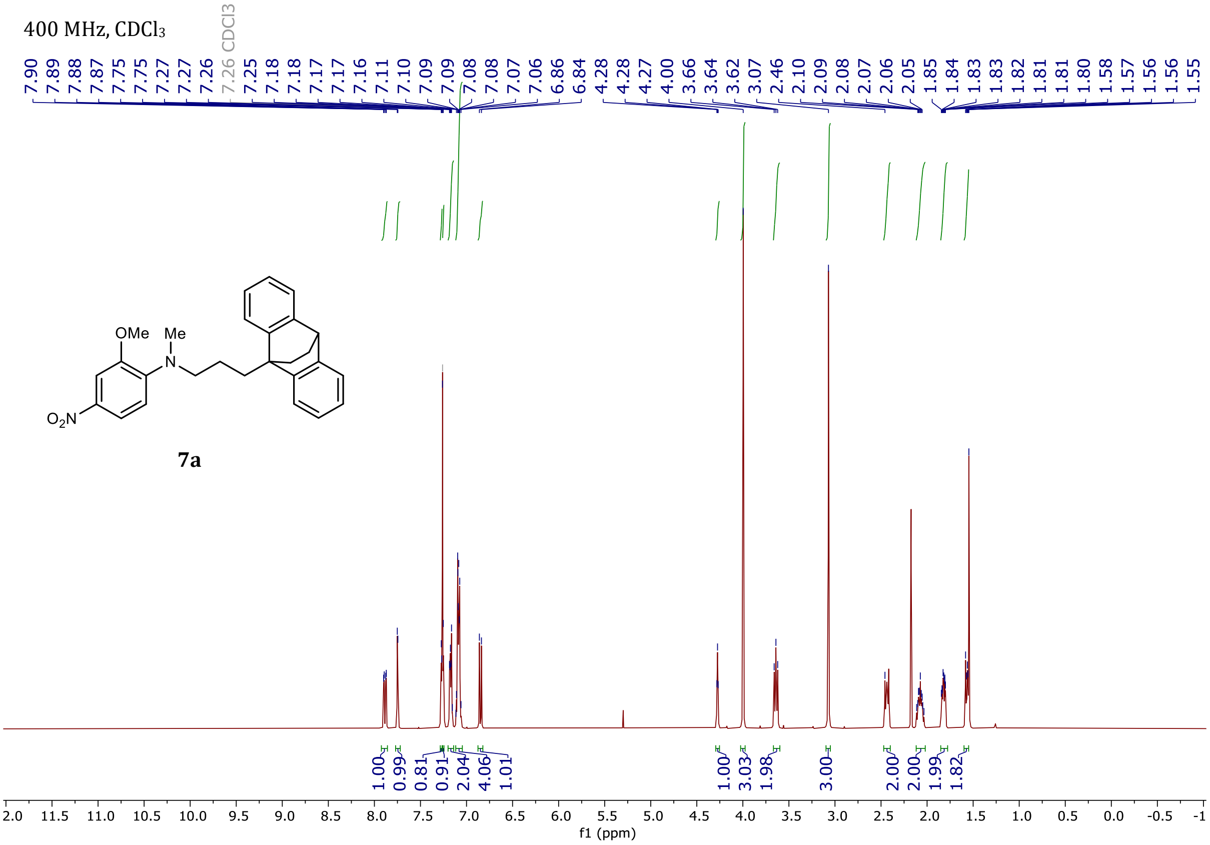
400 MHz, CDCl3
7.90 7.89 7.88 7.87 7.75 7.75 7.27 7.27 7.26 7.26 CDCl3 7.25 7.18 7.18 7.17 7.17 7.16 7.11 7.10 7.09 7.09 7.08 7.08 7.07 7.06 6.86 6.84 4.28 4.28 4.27 4.00 3.66 3.64 3.62 3.07 2.46 2.10 2.09 2.08 2.07 2.06 2.05 1.85 1.84 1.83 1.83 1.82 1.81 1.81 1.80 1.58 1.57 1.56 1.56 1.55
OMe
Me
N
O2N
7a
1.00 0.99 0.81 0.91 2.04 4.06 1.01 1.00 3.03 1.98 3.00 2.00 2.00 1.99 1.82
f1 (ppm)

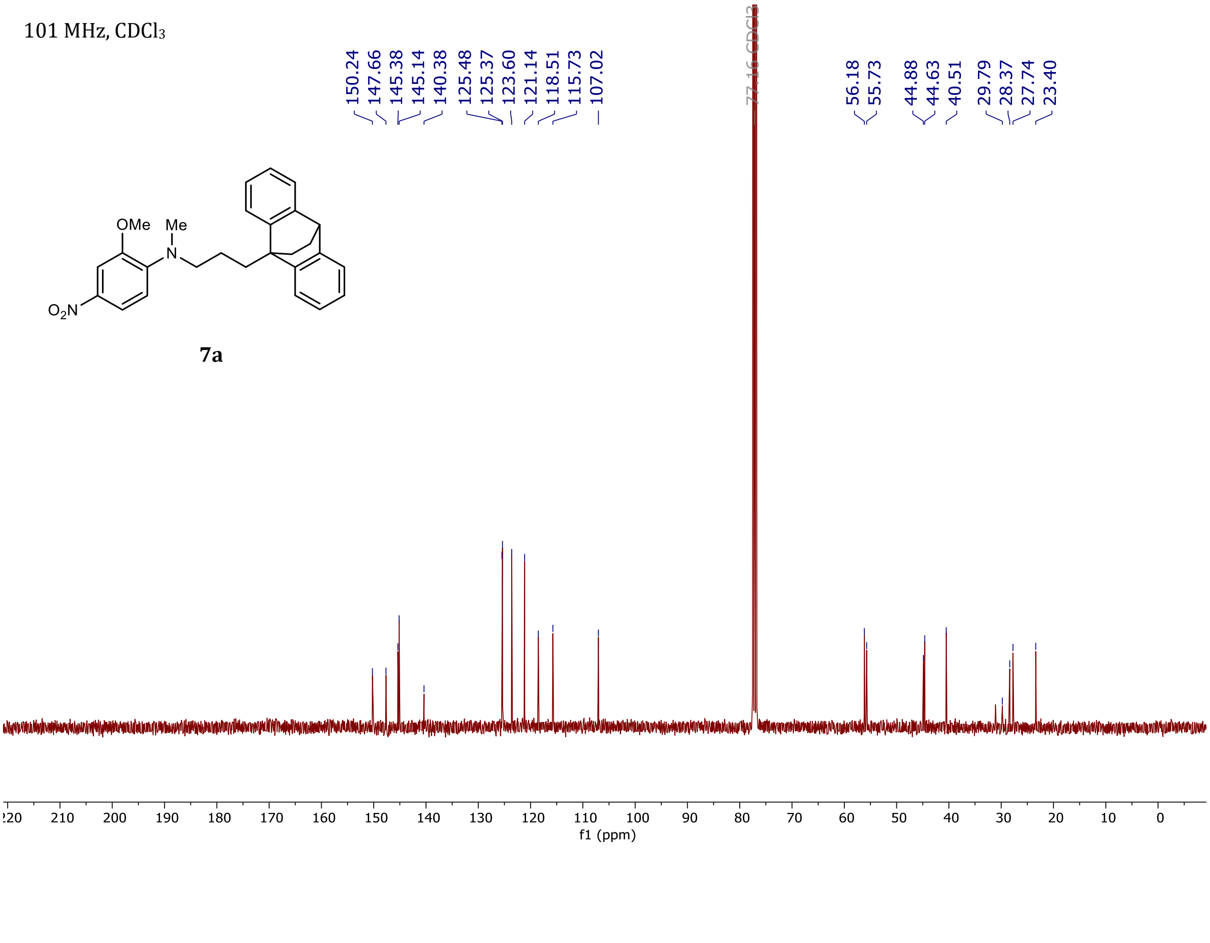
101 MHz, CDCl3
150.24 147.66 145.38 145.14 140.38 125.48 125.37 123.60 121.14 118.51 115.73 107.02
77.16 CDCl3
56.18 55.73 44.88 44.63 40.51 29.79 28.37 27.74 23.40
OMe
Me
N
O2N
7a
f1 (ppm)

400 MHz, $CDCl_3$

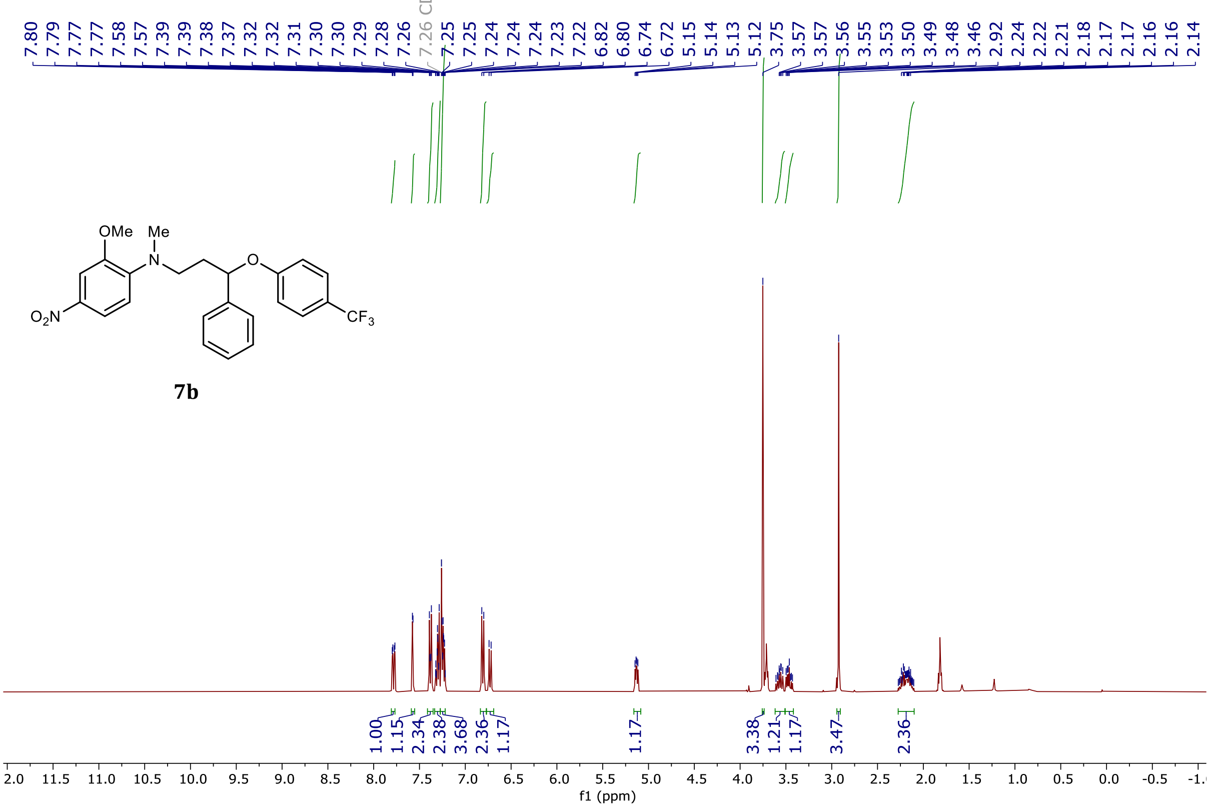

101 MHz, $CDCl_3$

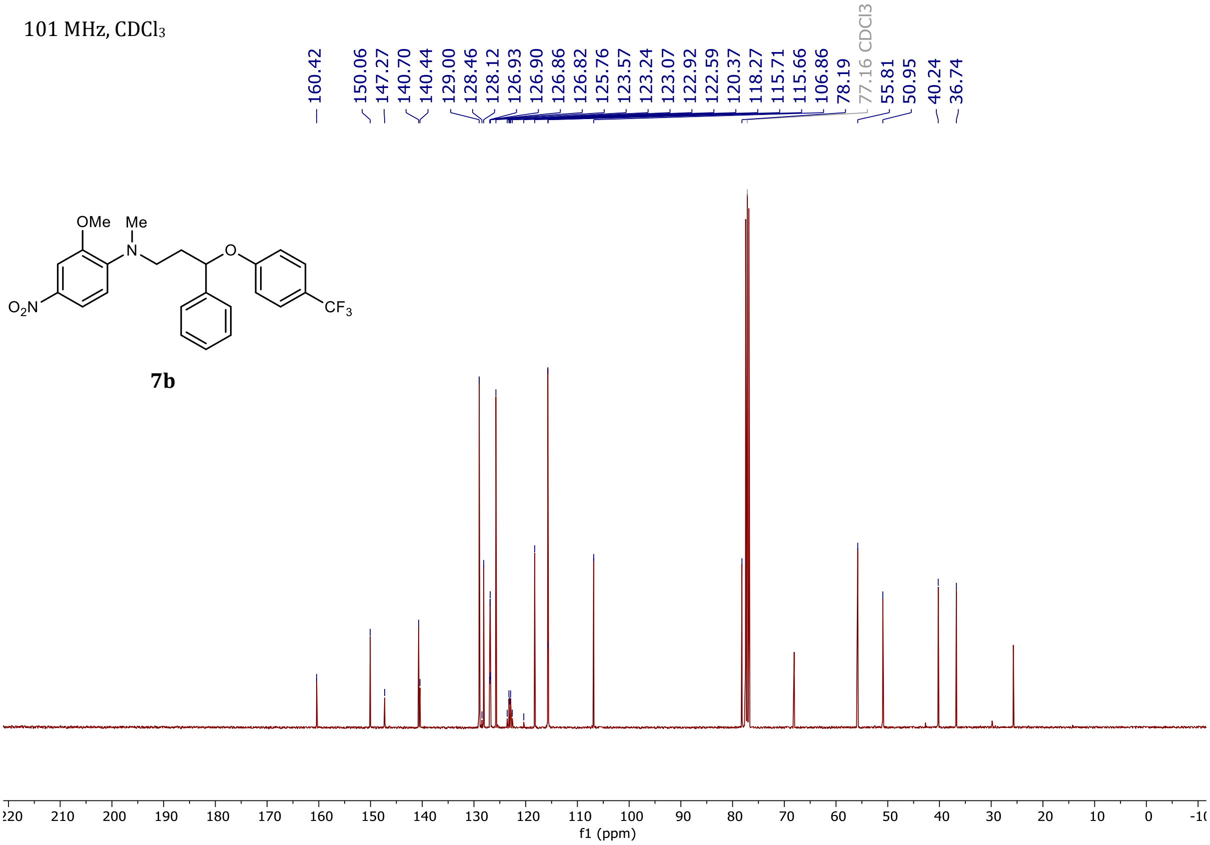

376 MHz, $CDCl_3$

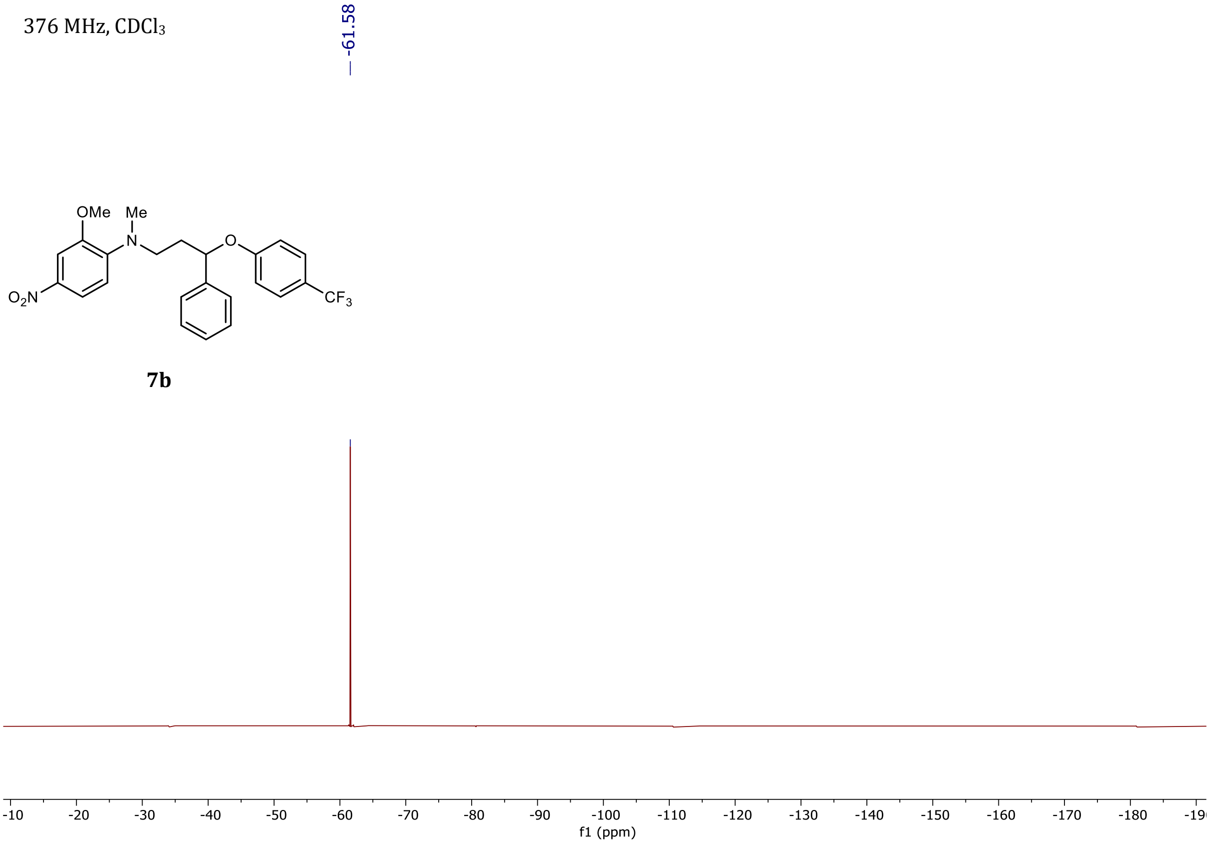

7b

400 MHz, $CDCl_3$

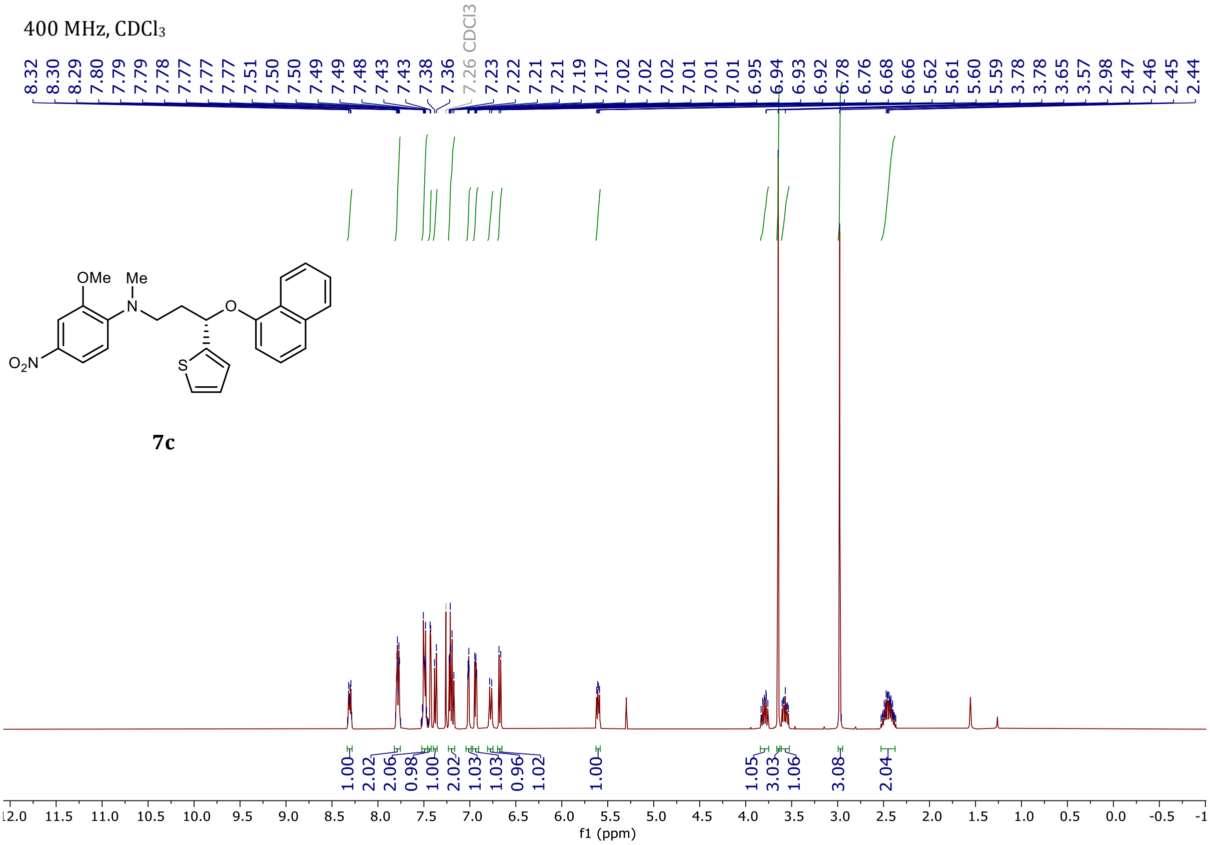

7c

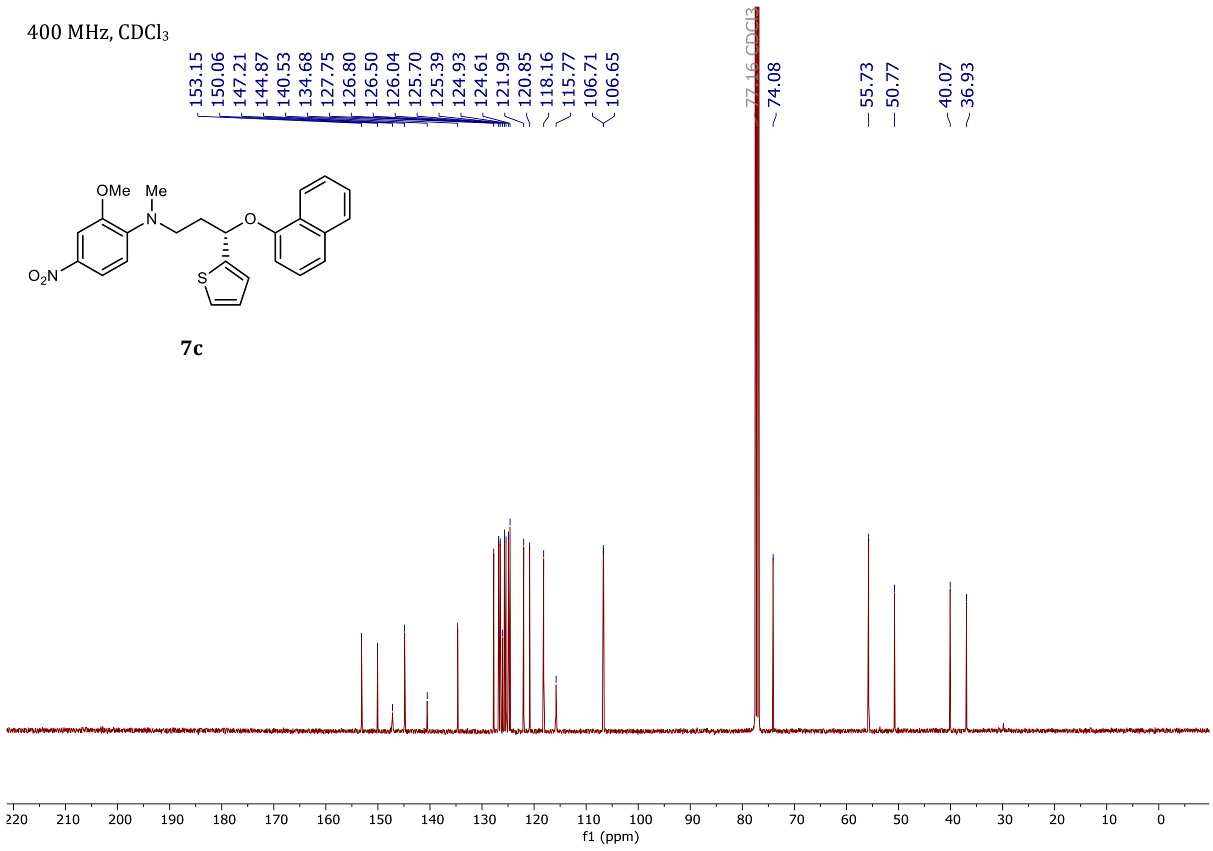
400 MHz, CDCl3
153.15
150.06
147.21
144.87
140.53
134.68
127.75
126.80
126.50
126.04
125.70
125.39
124.93
124.61
121.99
120.85
118.16
115.77
106.71
106.65
77.16 CDCl3
74.08
55.73
50.77
40.07
36.93
OMe
Me
N
O
O_2N
S
f1 (ppm)

7c

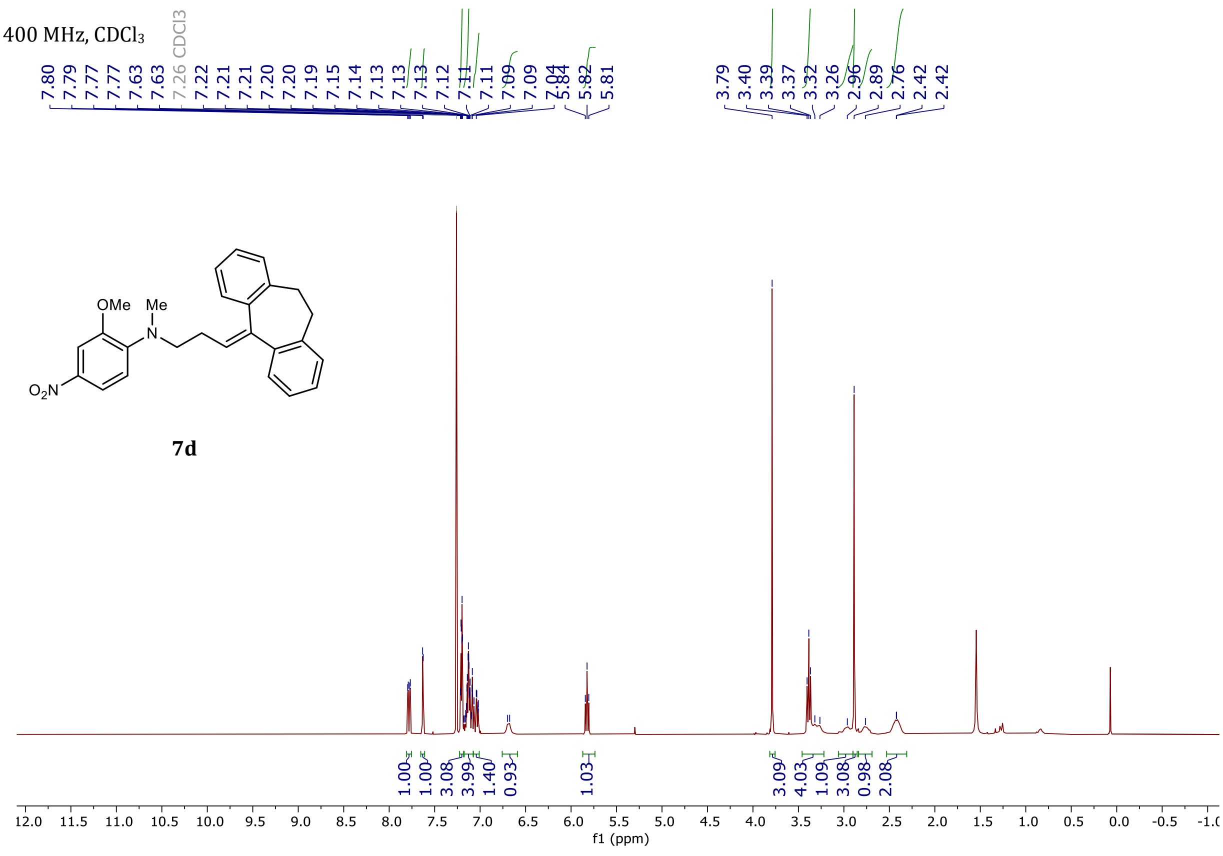
400 MHz, CDCl3
7.80
7.79
7.77
7.63
7.63
7.26 CDCl3
7.22
7.21
7.21
7.20
7.20
7.19
7.15
7.14
7.13
7.13
7.13
7.12
7.11
7.11
7.09
7.04
5.84
5.82
5.81
3.79
3.40
3.39
3.37
3.32
3.26
2.96
2.89
2.76
2.42
2.42
OMe
Me
N
O_2N
1.00
1.00
3.08
3.99
1.40
0.93
1.03
3.09
4.03
1.09
3.08
0.98
2.08
f1 (ppm)

7d

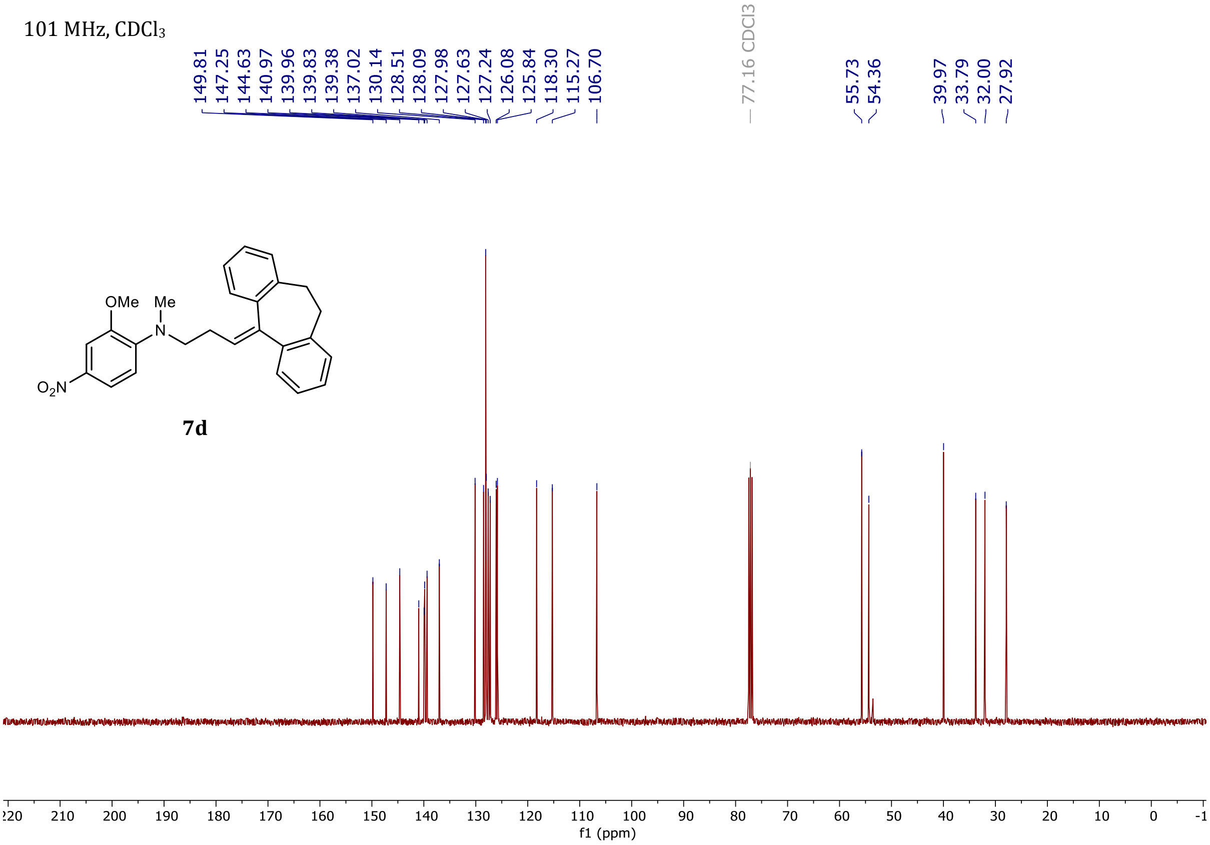
101 MHz, CDCl$_3$
149.81
147.25
144.63
140.97
139.96
139.83
139.38
137.02
130.14
128.51
128.09
127.98
127.63
127.24
126.08
125.84
118.30
115.27
106.70
77.16 CDCl3
55.73
54.36
39.97
33.79
32.00
27.92
OMe
Me
N
O_2N
f1 (ppm)

7d

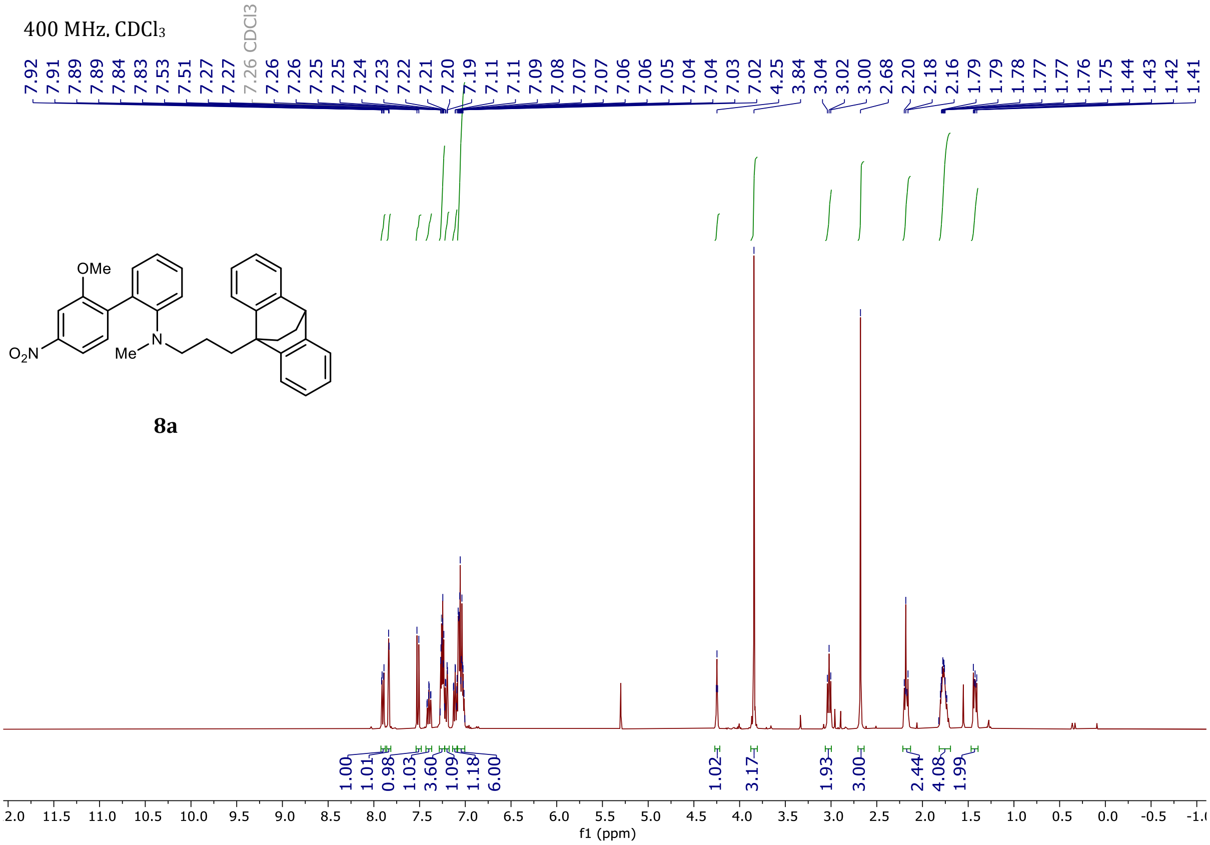
400 MHz, CDCl$_3$
7.92
7.91
7.89
7.89
7.84
7.83
7.53
7.51
7.27
7.27
7.26 CDCl3
7.26
7.25
7.25
7.24
7.23
7.22
7.21
7.20
7.19
7.11
7.09
7.08
7.07
7.07
7.06
7.06
7.05
7.04
7.03
7.02
4.25
3.84
3.04
3.02
3.00
2.68
2.20
2.18
2.16
1.79
1.79
1.78
1.77
1.76
1.75
1.44
1.43
1.42
1.41
1.00
1.01
0.98
1.03
3.60
1.09
1.18
6.00
1.02
3.17
1.93
3.00
2.44
4.08
1.99
OMe
O_2N
Me
N
f1 (ppm)

8a

101 MHz, $CDCl_3$

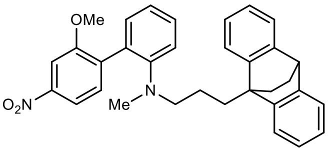

8a

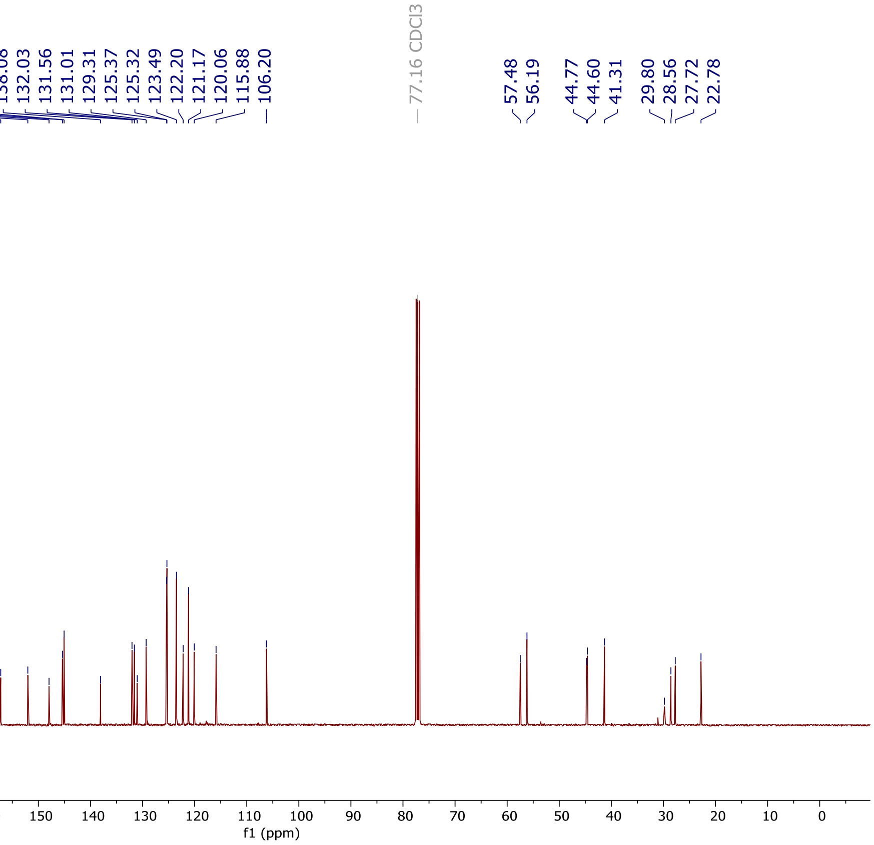

400 MHz, $CDCl_3$

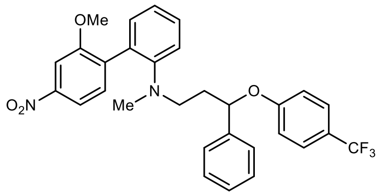

8b

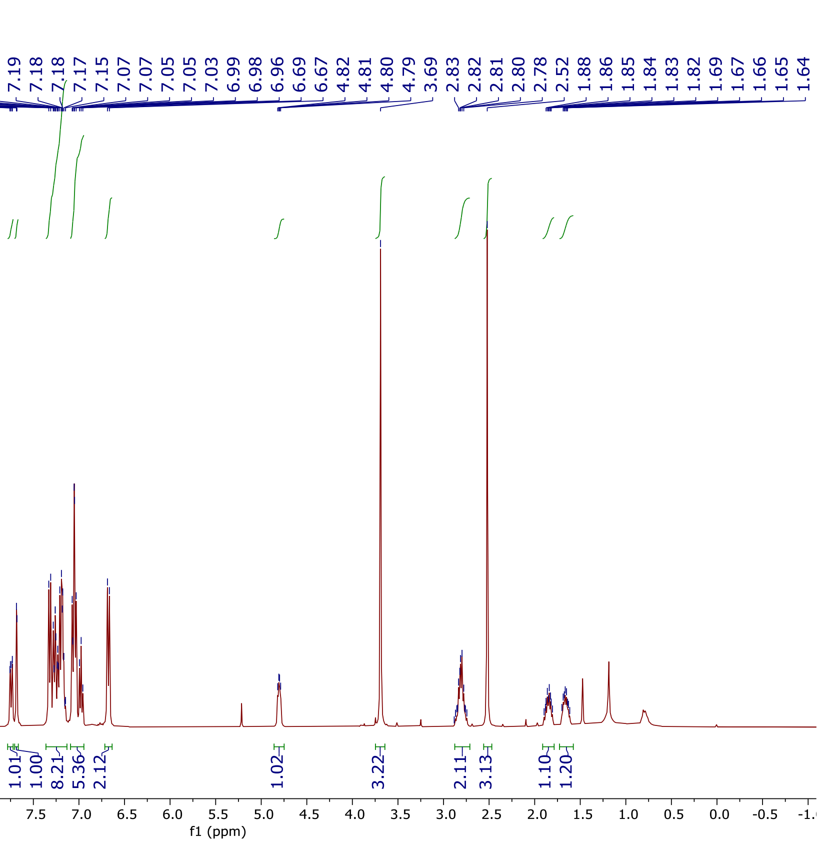

101 MHz, CDCl3

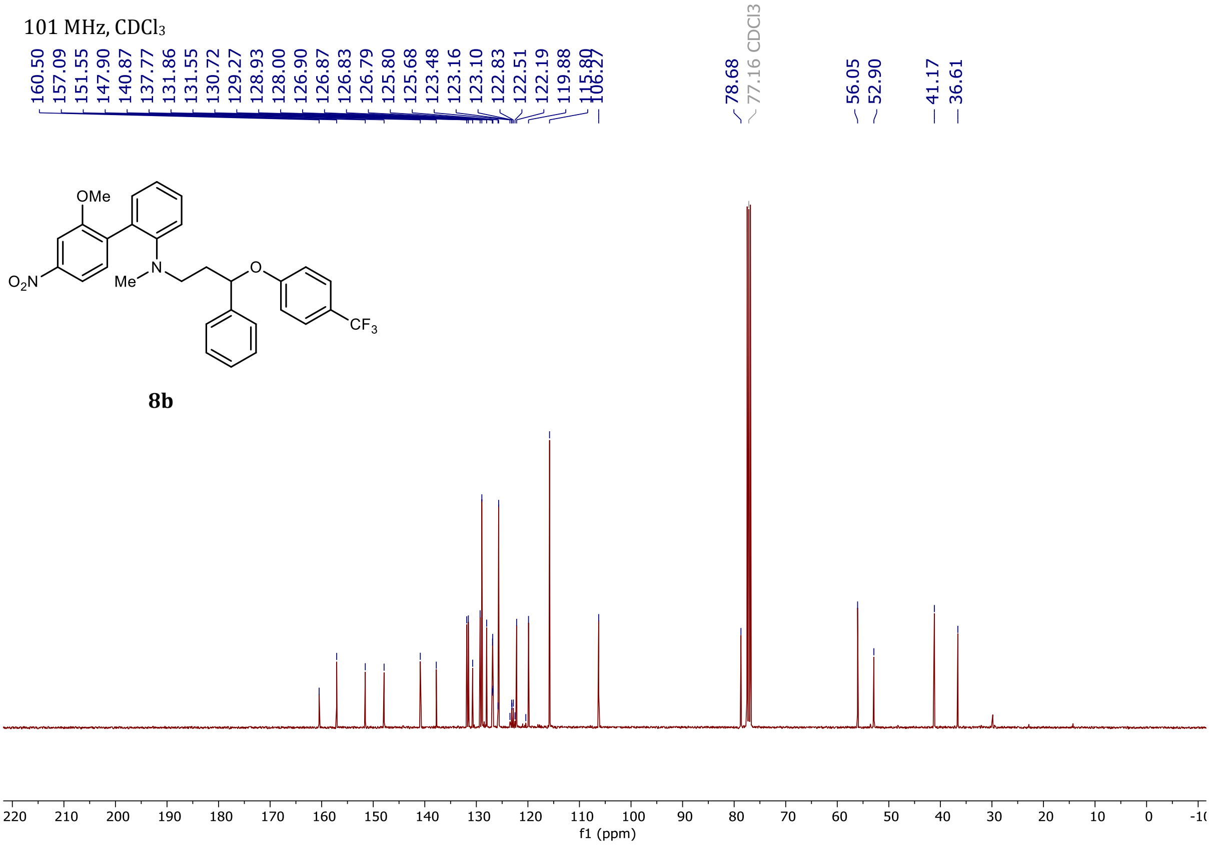

8b

400 MHz, CDCl3

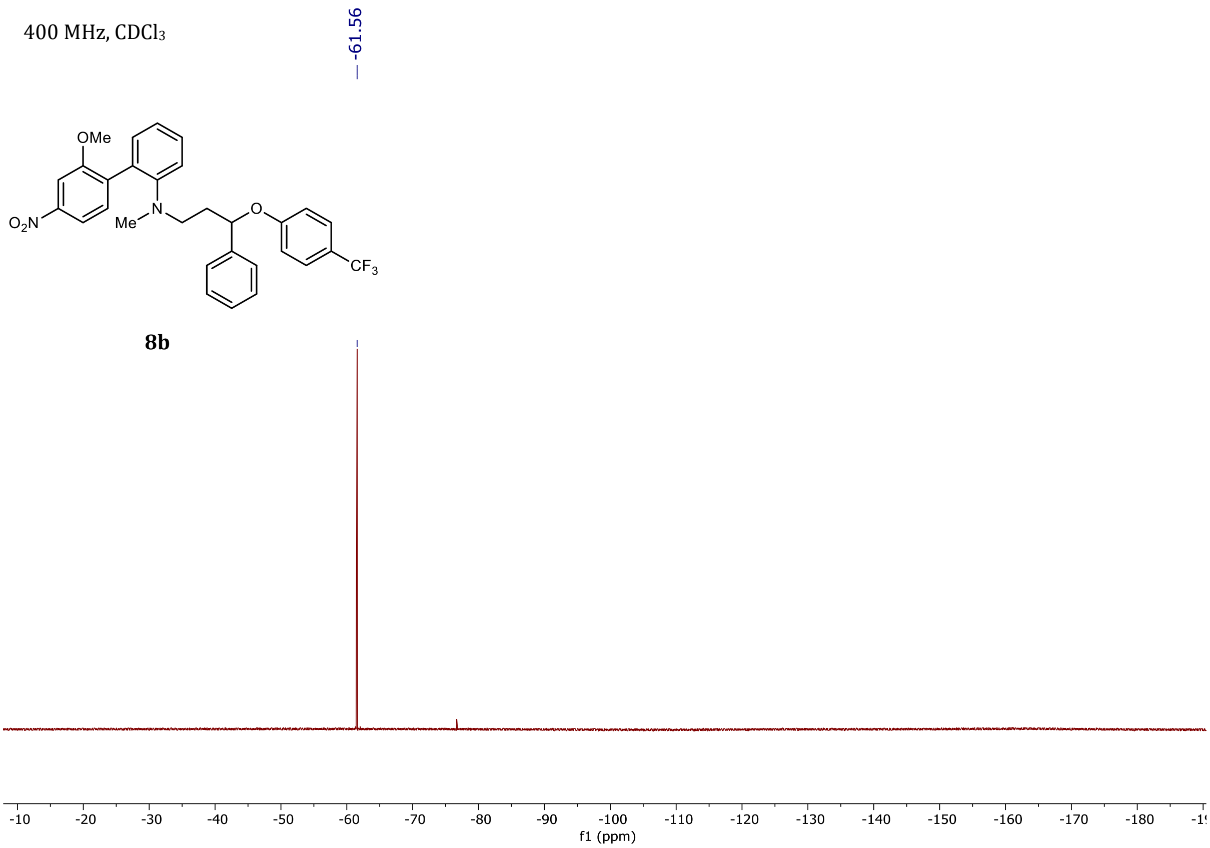

8b

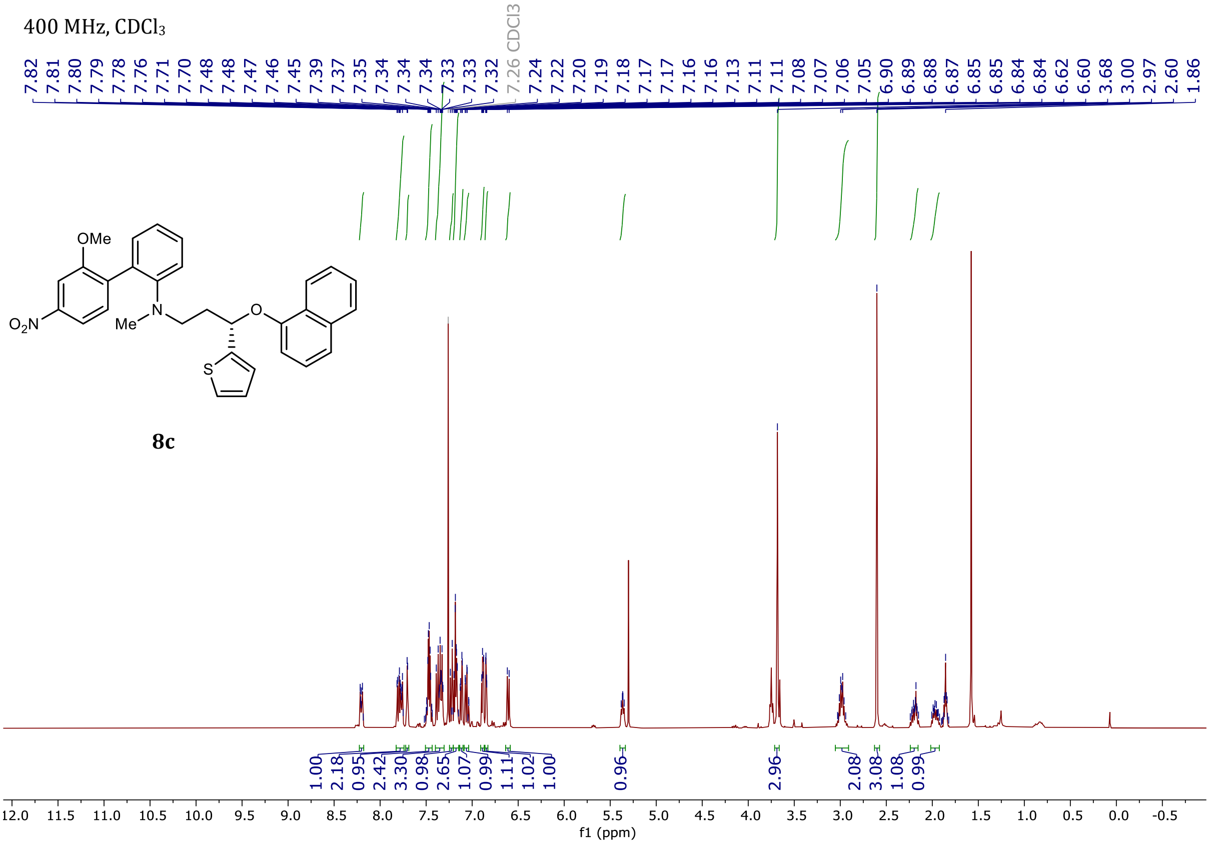
400 MHz, CDCl$_3$
7.82 7.81 7.80 7.79 7.78 7.76 7.71 7.70 7.48 7.48 7.47 7.46 7.45 7.39 7.37 7.35 7.34 7.34 7.33 7.33 7.32 7.26 CDCl3 7.24 7.22 7.20 7.19 7.18 7.17 7.17 7.16 7.13 7.11 7.11 7.08 7.07 7.06 7.05 6.90 6.89 6.88 6.87 6.85 6.85 6.84 6.84 6.62 6.60 3.68 3.00 2.97 2.60 1.86
OMe
O_2N
Me
N
O
S
8c
1.00 2.18 0.95 2.42 3.30 0.98 2.65 1.07 0.99 1.11 1.02 1.00 0.96 2.96 2.08 3.08 1.08 0.99
12.0 11.5 11.0 10.5 10.0 9.5 9.0 8.5 8.0 7.5 7.0 6.5 6.0 5.5 5.0 4.5 4.0 3.5 3.0 2.5 2.0 1.5 1.0 0.5 0.0 -0.5
f1 (ppm)

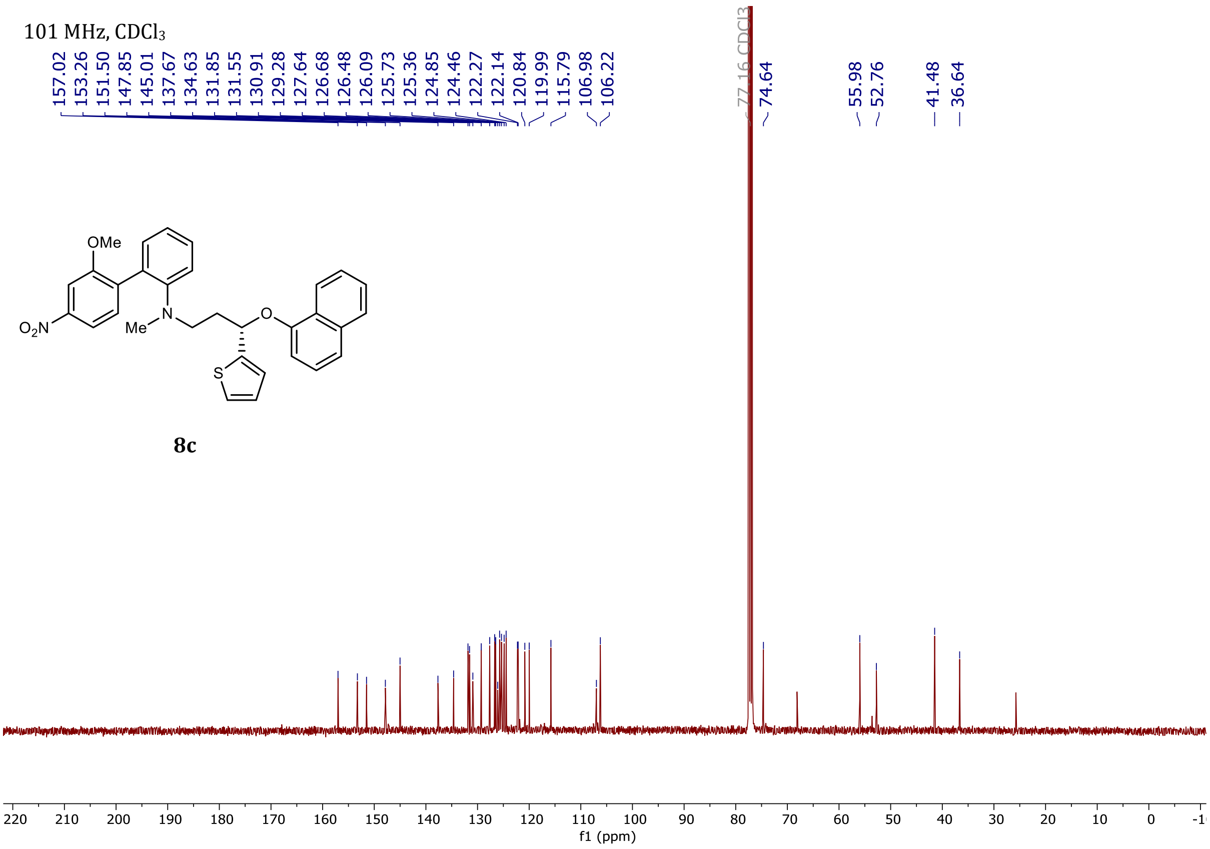
101 MHz, CDCl$_3$
157.02 153.26 151.50 147.85 145.01 137.67 134.63 131.85 131.55 130.91 129.28 127.64 126.68 126.48 126.09 125.73 125.36 124.85 124.46 122.27 122.14 120.84 119.99 115.79 106.98 106.22
77.16 CDCl3 74.64 55.98 52.76 41.48 36.64
OMe
O_2N
Me
N
O
S
8c
220 210 200 190 180 170 160 150 140 130 120 110 100 90 80 70 60 50 40 30 20 10 0 -1
f1 (ppm)

400 MHz, CDCl3

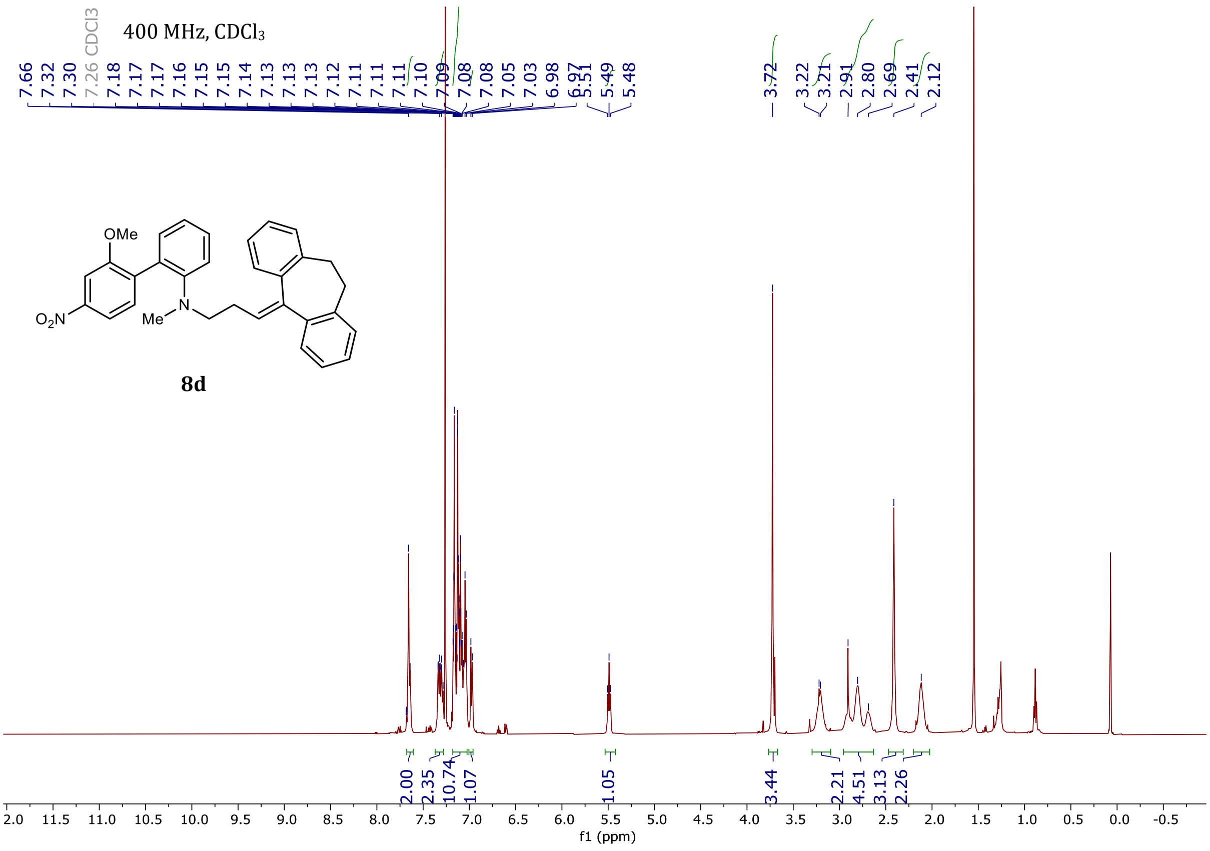

8d

101 MHz, CDCl3

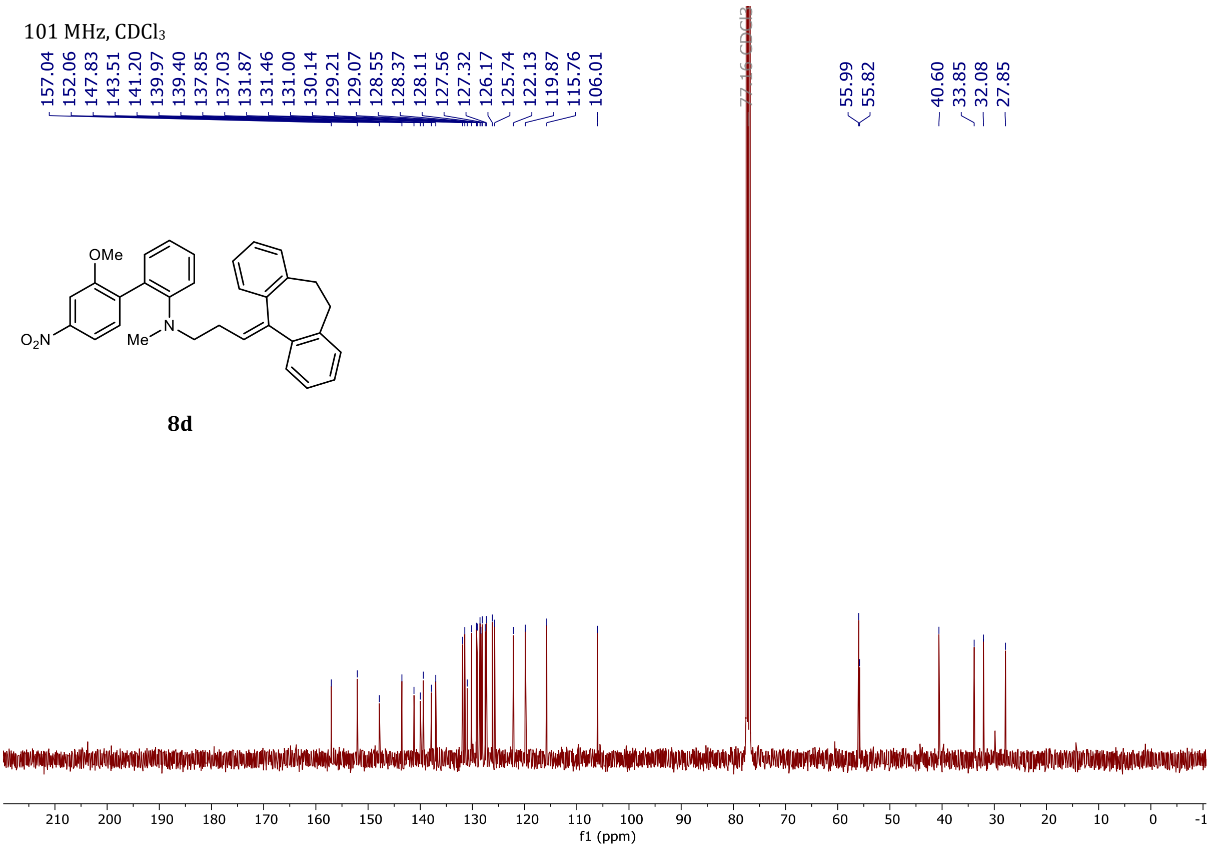

8d

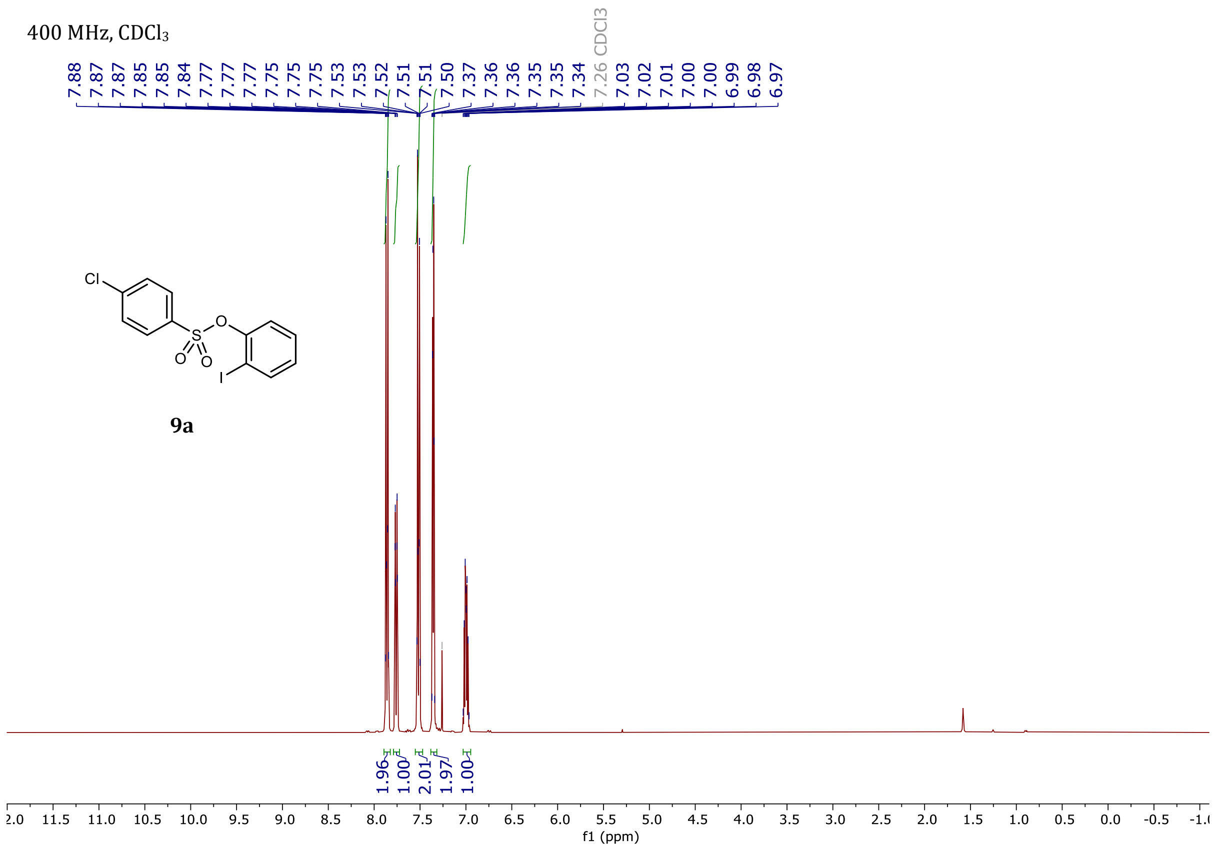
400 MHz, CDCl3
7.88 7.87 7.87 7.85 7.85 7.84 7.77 7.77 7.77 7.75 7.75 7.75 7.53 7.53 7.52 7.51 7.51 7.50 7.37 7.36 7.36 7.35 7.35 7.34 7.26 CDCl3 7.03 7.02 7.01 7.00 7.00 6.99 6.98 6.97
Cl
O
S
O O
I
9a
1.96 1.00 2.01 1.97 1.00
f1 (ppm)

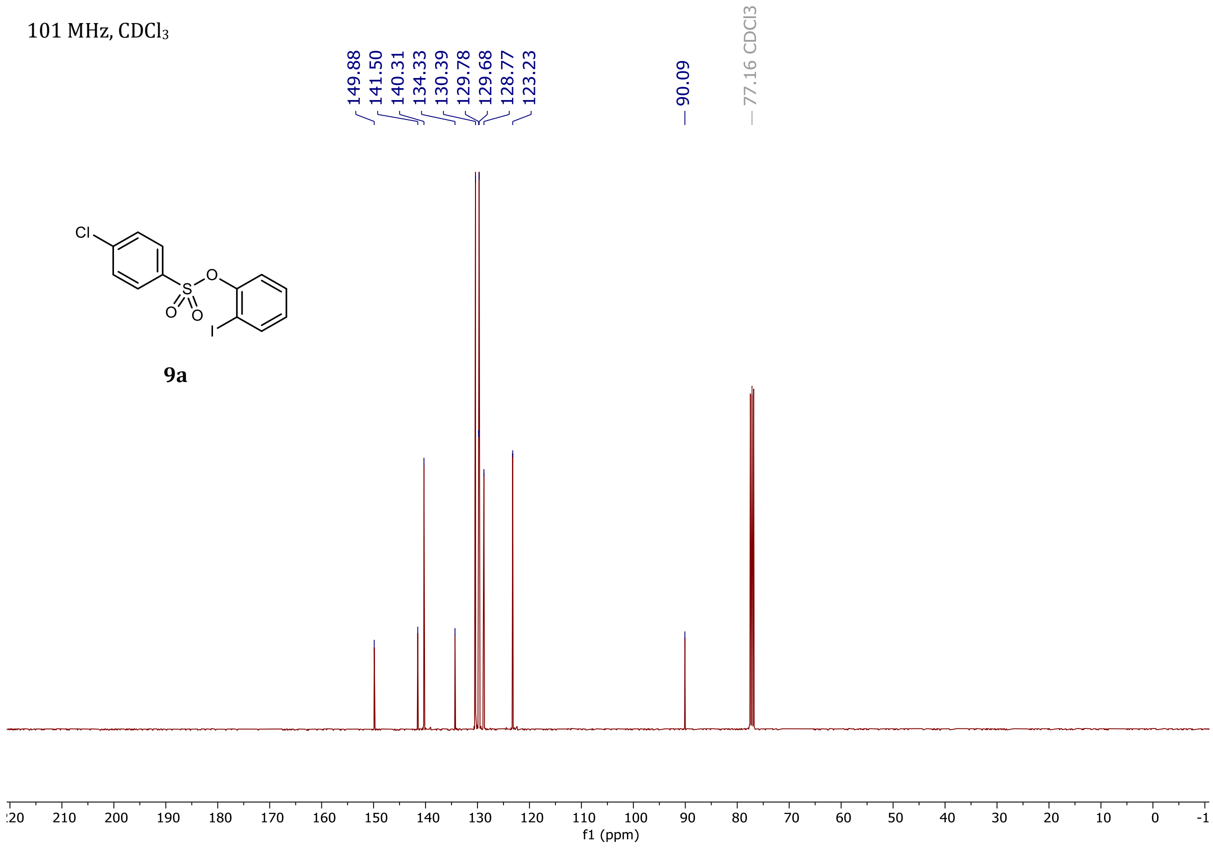
101 MHz, CDCl3
149.88 141.50 140.31 134.33 130.39 129.78 129.68 128.77 123.23
90.09
77.16 CDCl3
Cl
O
S
O O
I
9a
f1 (ppm)

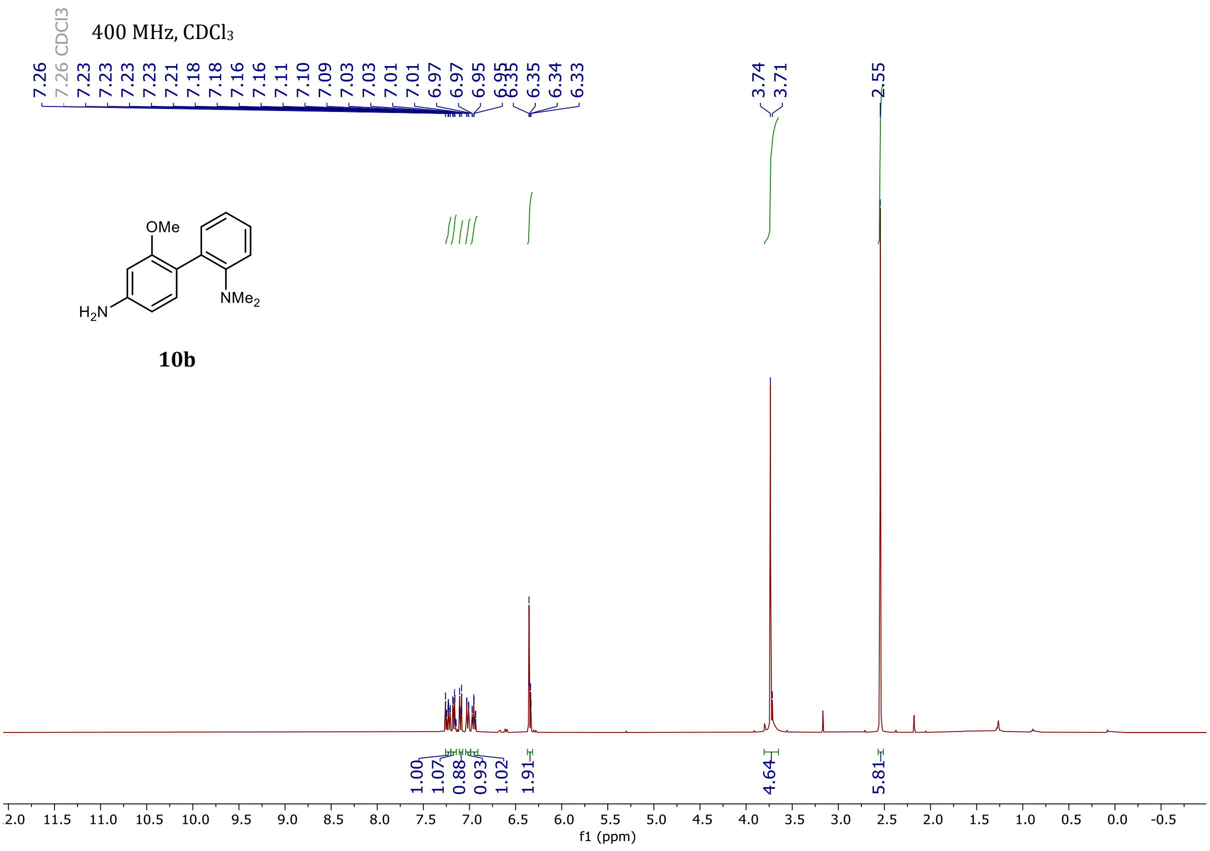
400 MHz, CDCl3
7.26 CDCl3
7.26 7.23 7.23 7.23 7.23 7.21 7.18 7.18 7.16 7.16 7.11 7.10 7.09 7.03 7.03 7.01 7.01 6.97 6.95 6.95 6.35 6.35 6.34 6.33
3.74 3.71
2.55
OMe
H2N
NMe2
10b
1.00 1.07 0.88 0.93 1.02 1.91
4.64
5.81
f1 (ppm)

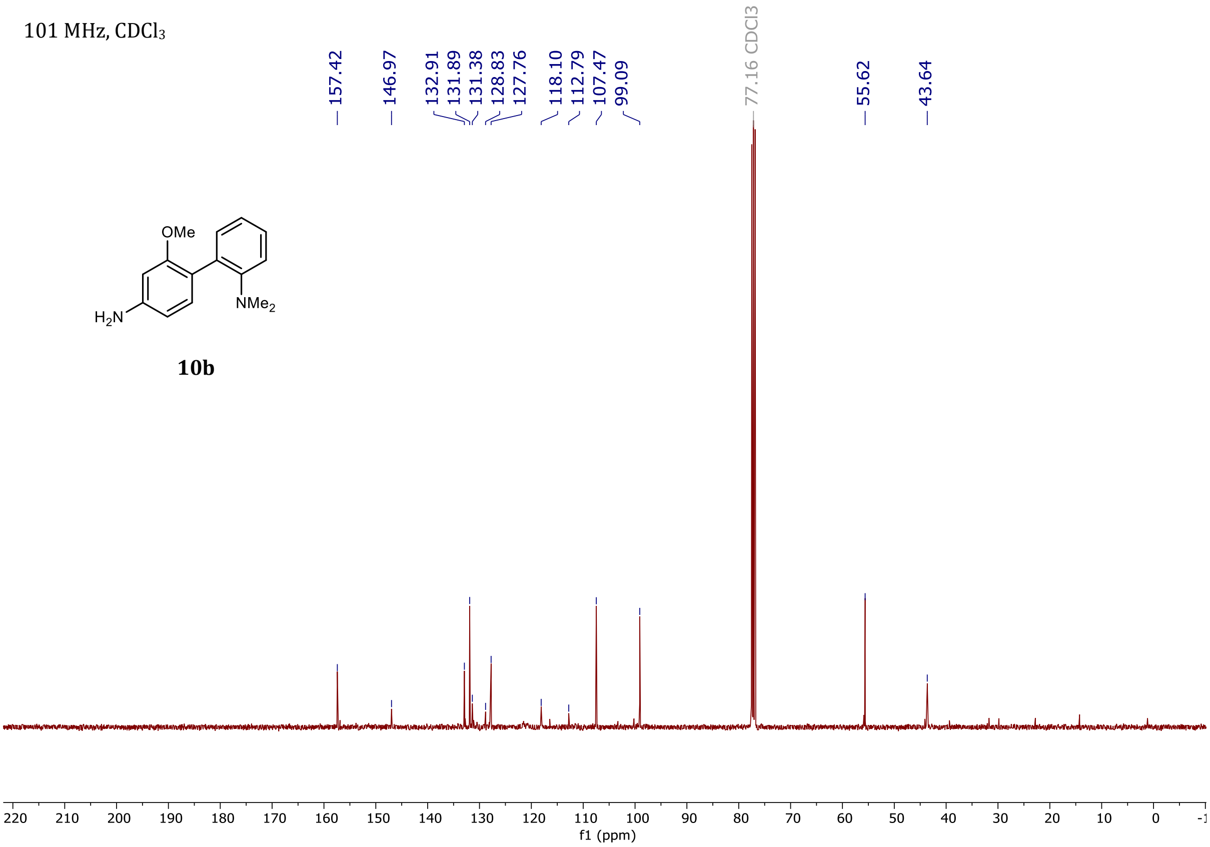
101 MHz, CDCl3
157.42
146.97
132.91 131.89 131.38 128.83 127.76
118.10 112.79 107.47 99.09
77.16 CDCl3
55.62
43.64
OMe
H2N
NMe2
10b
f1 (ppm)

400 MHz, $CDCl_3$

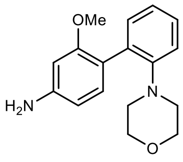

10n

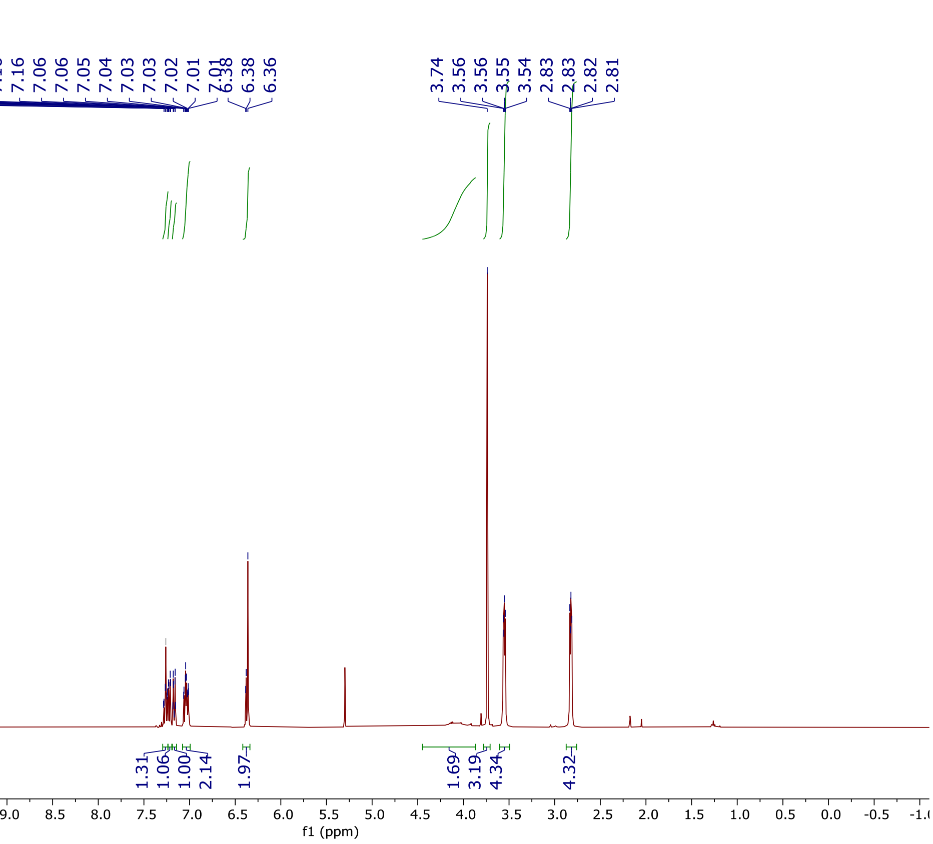

101 MHz, $CDCl_3$

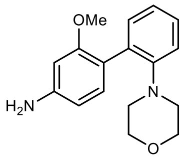

10n

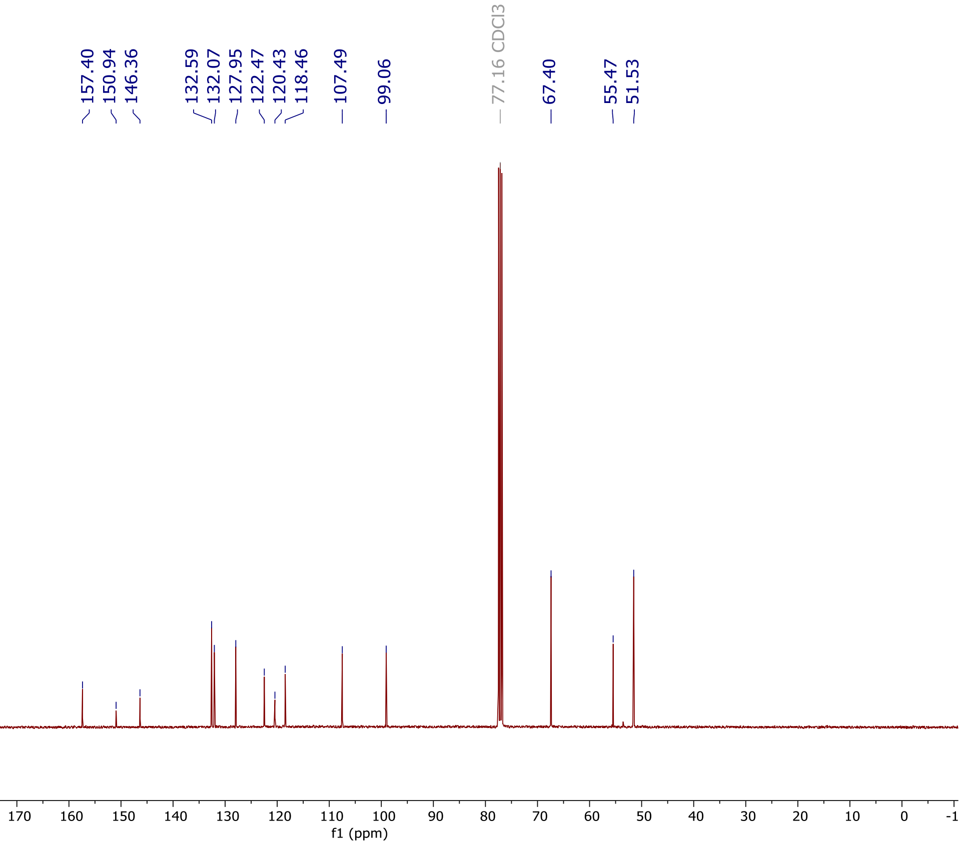

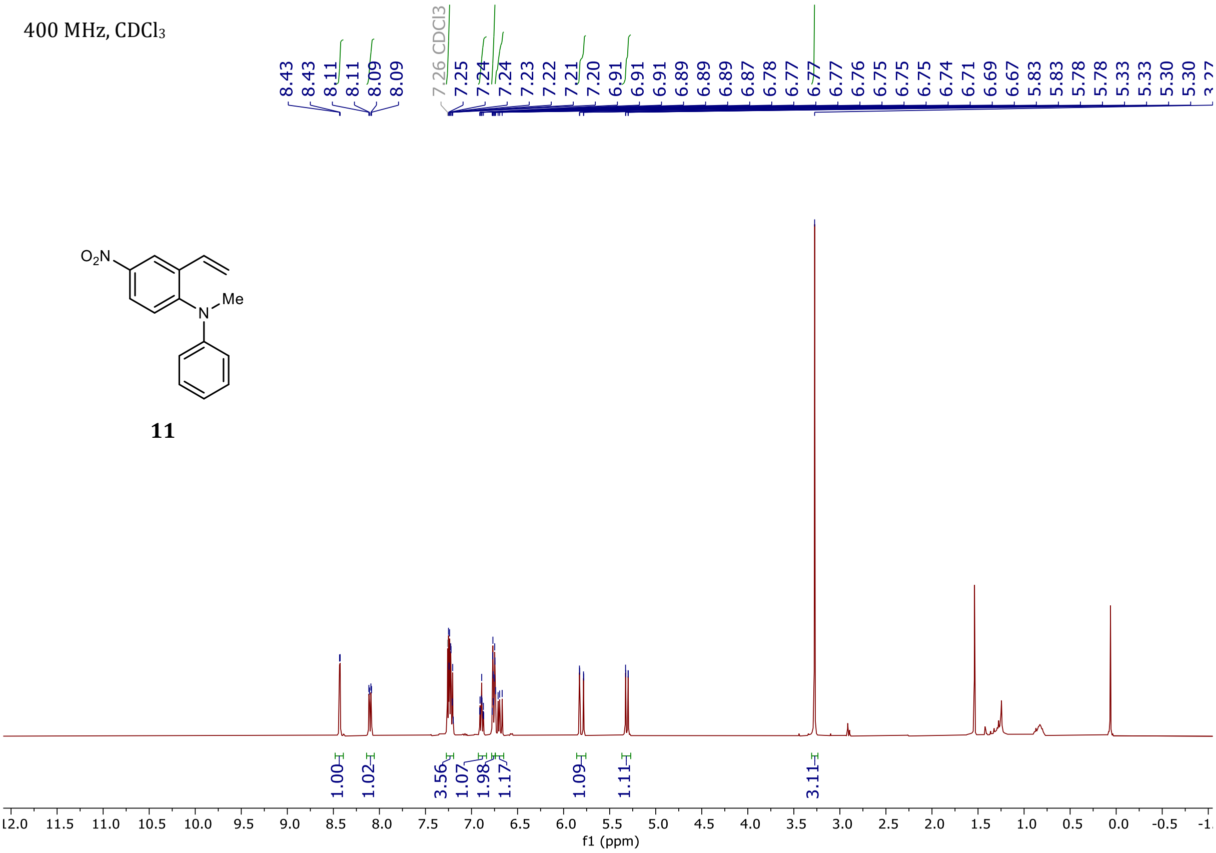
400 MHz, CDCl3
8.43
8.43
8.11
8.11
8.09
8.09
7.26 CDCl3
7.25
7.24
7.24
7.23
7.22
7.21
7.20
6.91
6.91
6.91
6.89
6.89
6.87
6.78
6.77
6.77
6.76
6.75
6.75
6.74
6.71
6.69
6.67
5.83
5.83
5.78
5.78
5.33
5.33
5.30
5.30
O2N
Me
N
11
1.00
1.02
3.56
1.07
1.98
1.17
1.09
1.11
3.11
f1 (ppm)

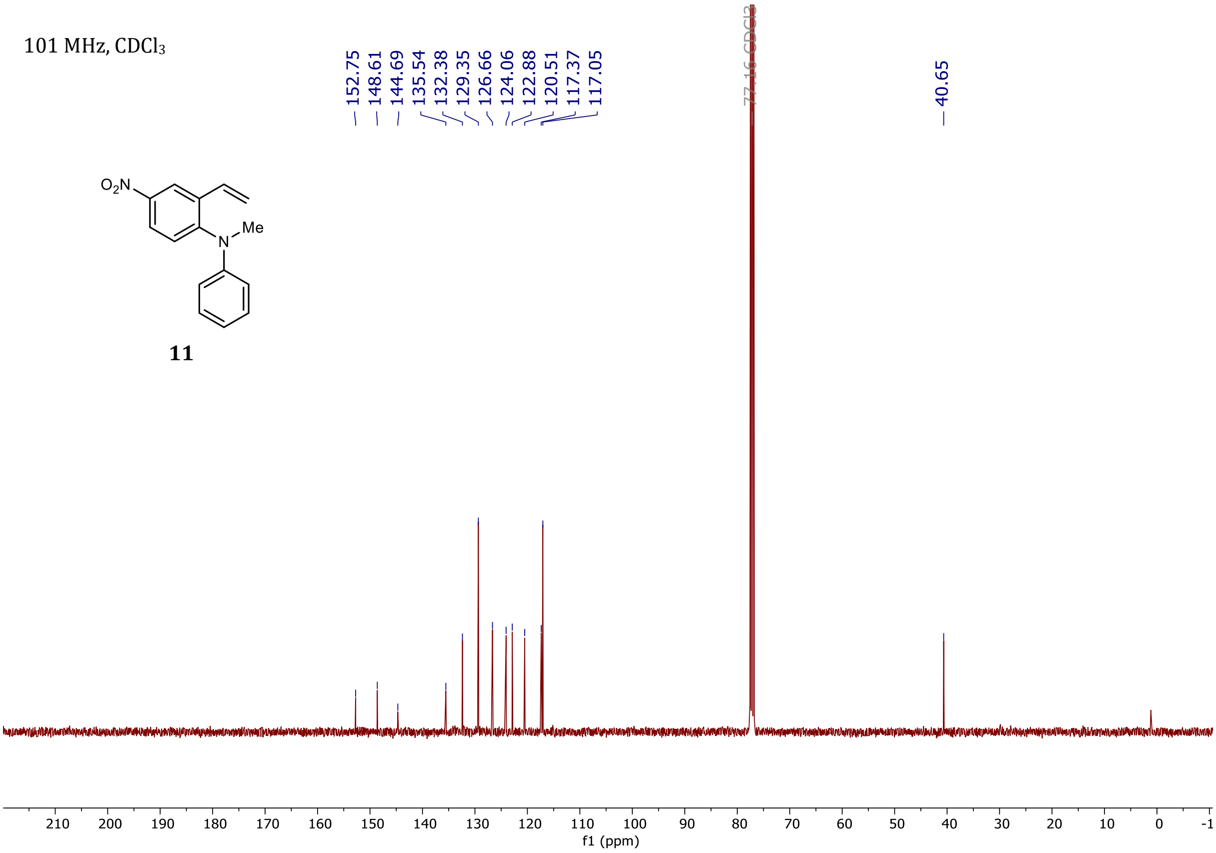
101 MHz, CDCl3
152.75
148.61
144.69
135.54
132.38
129.35
126.66
124.06
122.88
120.51
117.37
117.05
77.16 CDCl3
40.65
O2N
Me
N
11
f1 (ppm)
